# Supplementary material for: Assessing the added value of linking electronic health records to improve the prediction of self-reported COVID-19 testing and diagnosis
Source: PLoS One. 2022 Jul 25;17(7):e0269017. doi: 10.1371/journal.pone.0269017 (PMC9312965; doi:10.1371/journal.pone.0269017)
Supplement: S1 Table — Each variable is named after the survey question it is derived from but does not always represent the syntax or nature of that question exactly. Certain survey questions were redefined for simplicity or interpretation. (PDF) [file pone.0269017.s001.pdf]

S1 Table. Complete Variable Descriptions

Name	Content*	Details
**Outcome Variables**		
Tested	Was the respondent/patient tested for COVID-19?	Binary. For survey-based analysis, derived from survey Question 2. For Michigan Medicine case-control analysis, cases include anyone who was tested for COVID-19 at Michigan Medicine from March 10th to June 30th, 2020.
Diagnosed	Was the respondent/patient diagnosed with COVID-19?	Binary. For survey-based analysis, derived directly from survey Question 1. Includes those diagnosed by a physician or by a test. Those who tested negative or self-diagnosed due to symptoms are excluded from controls. For Michigan Medicine case-control analysis, cases include anyone who was diagnosed with COVID-19 at Michigan Medicine by a physician or test from March 10th to June 30th, 2020.
Self-Diagnosed	Did the respondent diagnosis *themselves* with COVID-19 due to symptoms?	Binary. 1 if reported to have "self-diagnosed due to symptoms" and 0 otherwise. For survey-based analysis, derived directly from survey Question 1. Self-diagnosis data is not available in Michigan Medicine EHR, so this outcome was excluded from the EHR-based case control analysis.
**Covariates**		
Age	Age of the respondent/patient.	Numeric. Known from EHR. In modeling, used per ten years.
Sex (Q25)	Sex of the respondent/patient.	Binary. 1 if female, 0 if male. Derived from survey responses.
BMI	Body Mass Index of the respondent/patient.	Numeric. Computed from survey-reported height and weight as kg/m^2 for recency purposes. Where missing, obtained from EHR.
Race/ Ethnicity	Race/Ethnicity of the respondent/patient	Nominal. Three levels: Non-Hispanic White, Non-Hispanic African American, Other. Derived from EHR, which had less missingness. Where still missing, obtained from survey.
Essential Worker (Q94)	Is the respondent currently working as an essential worker?	Binary. Derived from survey responses.
Education (Q67)	Highest level of school the respondent has completed.	Nominal. Four Levels: High School or Less, Associate Degree, Bachelor's Degree (reference), Advanced Degree
**Survey Variables**		
Q130	Is someone in the respondent's household high risk, other than the respondent?	Binary. 1 if yes, 0 if no.
Q38	Is a medical professional living in the respondent's household, other than the respondent?	Binary. 1 if yes, 0 if no.
Q59	Is a first responder living in the respondent's household, other than the respondent?	Binary. 1 if yes, 0 if no.
Q13	How many times has the respondent gotten the flu in the past year?	Numeric. Integer 0-3. ("3 or more" treated as 3)

S1 Table (continued)

Name	Content*	Details
Q46	Did the respondent get a flu shot in the past year?	Binary. 1 if yes, 0 if no.
Q16	How many times has the respondent had the common cold in the past year?	Numeric. Values 0, 1.5 (representing “1-2” response), 3.5 (“3-4”), and 5.5 (“5+”)
Q17	Has the respondent ever been hospitalized for a viral infection, excluding COVID-19?	Binary. 1 if yes, 0 if no.
Q18	How concerned has the respondent been about COVD-19 in the past 7 days?	Numeric. Integer values 0-10.
Q23.1	How concerned is the respondent about: Someone close to them contracting COVID-19?	Binary. (Not or Slightly Concerned treated as 0, Very or Extremely Concerned as 1)
Q23.2	How concerned is the respondent about: Getting into serious financial trouble due to COVID-19?	Binary. (Not or Slightly Concerned treated as 0, Very or Extremely Concerned as 1)
Q23.3	How concerned is the respondent about: Losing their job due to COVID-19?	Binary. (Not or Slightly Concerned treated as 0, Very or Extremely Concerned as 1)
Q23.4	How concerned is the respondent about: How long it will be before life returns to normal?	Binary. (Not or Slightly Concerned treated as 0, Very or Extremely Concerned as 1)
Q23.5	How concerned is the respondent about: Not seeing friends and family?	Binary. (Not or Slightly Concerned treated as 0, Very or Extremely Concerned as 1)
Q24	Respondent’s level of agreement: “Since the outbreak, I see others in my community as a threat”	Nominal. Three levels: Disagree (totally or slightly), Neutral, Agree (totally or slightly). Neutral used as reference.
Q27	Respondent’s level of agreement: “I have had more negative thoughts toward Asian Americans”	Nominal. Three levels: Disagree (totally or slightly), Neutral, Agree (totally or slightly) Neutral used as reference.
Q45	Respondent’s level of agreement: “I have had more negative thoughts toward Asian Americans”	Nominal. Three levels: Disagree (totally or slightly), Neutral, Agree (totally or slightly) Neutral used as reference.
Q81	Have any of the respondent’s blood relatives been diagnosed with COVID-19?	Binary. 1 if yes, 0 if no.
Q85	Have any of the respondent’s blood relatives died of COVID-19?	Binary. 1 if yes, 0 if no.
Q133	Is the respondent pregnant?	Binary. 1 if yes, 0 if no.
Q66	What is the respondent’s average annual household income?	Nominal. Three levels: Low (≤40,000), Medium, High (> 100,000). Medium used as reference.
Q150	How many automobiles does the respondent have?	Numeric. Integer 0-10. (10+ response option treated as 10)
Q151	What is the respondent’s primary mode of transportation?	Nominal. Two levels: Car, and Other
Q152	What is the respondent’s current living situation?	Nominal. Four levels: Own a house, rent an apartment, live in family-owned property, and other. Own a house used as reference.

S1 Table (continued)

Name	Content*	Details
Q68.1	The respondent’s fatigue over the past week.	Binary. 1 if Moderate or Severe, 0 if Mild or None.
Q68.2	The respondent’s trouble thinking or remembering in the past week.	Binary. 1 if Moderate or Severe, 0 if Mild or None.
Q68.3	The respondent’s waking up tired or unrefreshed in the past week.	Binary. 1 if Moderate or Severe, 0 if Mild or None.
Q70.1	During the past six months, has the respondent had pain or cramps in lower abdomen?	Binary. 1 if yes, 0 if no.
Q70.2	During the past six months, has the respondent had depression?	Binary. 1 if yes, 0 if no.
Q70.3	During the past six months, has the respondent had headaches?	Binary. 1 if yes, 0 if no.
Q71.1	How easily is the respondent able to do chores such as vacuuming or yardwork?	Nominal. Three levels: No difficulty, Some Difficulty, Much difficulty or Unable. No difficulty used as reference.
Q71.2	How easily is the respondent able to go own stairs at a normal pace?	Nominal. Three levels: No difficulty, Some Difficulty, Much difficulty or Unable. No difficulty used as reference.
Q71.3	How easily is the respondent able to go for a walk of at least 15 minutes?	Nominal. Three levels: No difficulty, Some Difficulty, Much difficulty or Unable. No difficulty used as reference.
Q71.4	How easily is the respondent able to run errands and shop?	Nominal. Three levels: No difficulty, Some Difficulty, Much difficulty or Unable. No difficulty used as reference.
Q72.1	In the past seven days, how often has the respondent felt worthless?	Nominal. Three levels: Rarely or Never, Sometimes, Often or Always. Rarely or Never as reference.
Q72.2	In the past seven days, how often has the respondent felt helpless?	Nominal. Three levels: Rarely or Never, Sometimes, Often or Always. Rarely or Never as reference.
Q72.3	In the past seven days, how often has the respondent felt depressed?	Nominal. Three levels: Rarely or Never, Sometimes, Often or Always. Rarely or Never as reference.
Q72.4	In the past seven days, how often has the respondent felt depressed?	Nominal. Three levels: Rarely or Never, Sometimes, Often or Always. Rarely or Never as reference.
Q74.1	In the past seven days, how often has the respondent felt fearful?	Nominal. Three levels: Rarely or Never, Sometimes, Often or Always. Rarely or Never as reference.
Q74.2	In the past seven days, how often has the respondent struggled to focus on anything but anxiety?	Nominal. Three levels: Rarely or Never, Sometimes, Often or Always. Rarely or Never as reference.
Q74.3	In the past seven days, how often did the respondent’s worries overwhelm them?	Nominal. Three levels: Rarely or Never, Sometimes, Often or Always. Rarely or Never as reference.
Q74.4	In the past seven days, how often has the respondent felt uneasy?	Nominal. Three levels: Rarely or Never, Sometimes, Often or Always. Rarely or Never as reference.
Q77	In the past seven days, how was the respondent’s sleep quality?	Binary. 1 if poor or very poor , 0 if fair or better.
Q80.1	How often does the respondent have someone to help them if they are confined in bed?	Nominal. Three levels: Rarely or Never, Sometimes, Usually or Always. Rarely or Never as reference.

S1 Table (continued)

Name	Content*	Details
Q80.2	How often does the respondent have someone to take them to the doctor if necessary?	Nominal. Three levels: Rarely or Never, Sometimes, Usually or Always. Rarely or Never as reference.
Q80.3	How often does the respondent have someone to help them with their daily chores if they are sick?	Nominal. Three levels: Rarely or Never, Sometimes, Usually or Always. Rarely or Never as reference.
Q80.4	How often does the respondent have someone to run errands for them?	Nominal. Three levels: Rarely or Never, Sometimes, Usually or Always Rarely or Never as reference.
Q141	Does the respondent have a primary care physician?	Binary. 1 if yes, 0 if no.
Q145	Does the respondent have an immune system condition?	Binary. 1 if yes, 0 if no.
Q146	Does the respondent have a respiratory condition?	Binary. 1 if yes, 0 if no.
Q147	Does the respondent have a genitourinary/metabolic condition?	Binary. 1 if yes, 0 if no.
Q125	Does the respondent have a cardiovascular condition?	Binary. 1 if yes, 0 if no.
Q127	Do any of these conditions/treatments apply to the respondent: Leukemia, Lymphoma, Malignant solid tumor, Chemotherapy, Radiation therapy	Binary. 1 if yes, 0 if no.
Q40	The respondent's tobacco/nicotine use status	Nominal. Four Levels: Current User, Never Used (reference), Former user
Q114.1	The respondent's overall body pain, at worst, in the past week.	Numeric. 0 – 10
Q114.2	The respondent's overall body pain, on average, in the past week.	Numeric. 0 – 10
Q56.1	Respondent's height.	Numeric. In inches.
Q56.2	Respondent's weight.	Numeric. In pounds.
Q88	In the past 12 months, how often has the respondent had 5 or more drinks if male or 4 or more drinks if female, in a single day?	Nominal. Three levels: Weekly or more, Monthly, Less than Monthly (reference)
Q38.1	Is there a physician currently living in the respondent's household?	Binary. 1 if yes, 0 if no.
Q38.2	Is there a nurse currently living in the respondent's household?	Binary. 1 if yes, 0 if no.
Q59.1	Is there a police officer currently living in the respondent's home?	Binary. 1 if yes, 0 if no.
Q59.2	Is there a firefighter currently living in the respondent's home?	Binary. 1 if yes, 0 if no.
Q59.3	Is there an EMT currently living in the respondent's home?	Binary. 1 if yes, 0 if no.
Q59.4	Is there military personnel currently living in the respondent's home?	Binary. 1 if yes, 0 if no.
Q145.1	Does the respondent have HIV?	Binary. 1 if yes, 0 if no.

S1 Table (continued)

Name	Content*	Details
Q145.2	Is the respondent immunocompromised?	Binary. 1 if yes, 0 if no.
Q145.3	Has the respondent had an organ transplant?	Binary. 1 if yes, 0 if no.
Q145.4	Has the respondent had a bone marrow transplant?	Binary. 1 if yes, 0 if no.
Q145.5	Does the respondent have Type I Diabetes?	Binary. 1 if yes, 0 if no.
Q145.6	Does the respondent have Type II Diabetes?	Binary. 1 if yes, 0 if no.
Q146.1	Does the respondent have asthma?	Binary. 1 if yes, 0 if no.
Q146.2	Does the respondent have COPD?	Binary. 1 if yes, 0 if no.
Q146.3	Does the respondent have Cystic Fibrosis?	Binary. 1 if yes, 0 if no.
Q146.4	Does the respondent have Emphysema?	Binary. 1 if yes, 0 if no.
Q146.5	Does the respondent have sleep apnea?	Binary. 1 if yes, 0 if no.
Q146.6	Does the respondent use a home CPAP?	Binary. 1 if yes, 0 if no.
Q147.1	Does the respondent have chronic kidney disease?	Binary. 1 if yes, 0 if no.
Q147.2	Does the respondent have liver disease?	Binary. 1 if yes, 0 if no.
Q147.3	Does the respondent have gallbladder disease?	Binary. 1 if yes, 0 if no.
Q147.4	Does the respondent have pancreas disease?	Binary. 1 if yes, 0 if no.
Q125.1	Has the respondent had a balloon angioplasty or percutaneous coronary intervention?	Binary. 1 if yes, 0 if no.
Q125.2	Has the respondent had a coronary artery bypass?	Binary. 1 if yes, 0 if no.
Q125.3	Has the respondent had congestive heart failure?	Binary. 1 if yes, 0 if no.
Q125.4	Does the respondent have hypertension?	Binary. 1 if yes, 0 if no.
Q125.5	Has the respondent had a myocardial infarction?	Binary. 1 if yes, 0 if no.
Q125.6	Does the respondent have peripheral vascular disease?	Binary. 1 if yes, 0 if no.
Q125.7	Has the respondent had a blood clot or blood clotting disorder?	Binary. 1 if yes, 0 if no.
Q125.8	Has the respondent had a stroke?	Binary. 1 if yes, 0 if no.
Q125.9	Does the respondent have arrythmia?	Binary. 1 if yes, 0 if no.
Q127.1	Does the respondent have leukemia?	Binary. 1 if yes, 0 if no.
Q127.2	Does the respondent have lymphoma?	Binary. 1 if yes, 0 if no.
Q127.3	Does the respondent have a malignant solid tumor?	Binary. 1 if yes, 0 if no.

S1 Table (continued)

Name	Content*	Details
Q127.4	Has the respondent had chemotherapy?	Binary. 1 if yes, 0 if no.
Q127.5	Has the respondent had radiation therapy?	Binary. 1 if yes, 0 if no.
Q36.live.alone	Does anyone else live with the respondent?	Binary. 1 if yes, 0 if no.
Q36.house.diagnose	Has anyone in the respondent’s household been diagnosed with COVID-19	Binary. 1 if yes, 0 if no.
Q18.G	How concerned has the respondent been about COVD-19 in the past 7 days?	Binary. 1 if yes, 0 if no.
Q126.1	Does the respondent have dementia?	Binary. 1 if yes, 0 if no.
Q126.2	Does the respondent have a neurological disease?	Binary. 1 if yes, 0 if no.
Q118.1	During lockdown, has the respondent’s moderate to strenuous exercise increased?	Binary. 1 if yes, 0 if no.
Q118.2	During lockdown, has the respondent’s alcohol consumption increased?	Binary. 1 if yes, 0 if no.
Q118.3	During lockdown, has the respondent’s drug us increased?	Binary. 1 if yes, 0 if no.
Q118.4	During lockdown, has the respondent’s tobacco use increased?	Binary. 1 if yes, 0 if no.
Q118.5	During lockdown, has the respondent improved the respondent’s sleep habits?	Binary. 1 if yes, 0 if no.
Q118.6	During lockdown, has the respondent improved their nutrition?	Binary. 1 if yes, 0 if no.
Q118.7	During lockdown, has the respondent gained weight?	Binary. 1 if yes, 0 if no.
Q133.1	During lockdown, has the respondent’s opioid pain medication use increased?	Binary. 1 if yes, 0 if no.
Q133.2	During lockdown, has the respondent’s benzodiazepine use increased?	Binary. 1 if yes, 0 if no.
Q133.3	During lockdown, has the respondent’s marijuana / cannabis us increased?	Binary. 1 if yes, 0 if no.
Q28.1	Is anyone in the respondent’s immediate family deceased?	Binary. 1 if yes, 0 if no.
Q28.2	Does anyone in the respondent’s immediate family have cancer?	Binary. 1 if yes, 0 if no.
Q28.3	Does anyone in the respondent’s immediate family have chronic kidney disease?	Binary. 1 if yes, 0 if no.
Q28.4	Does anyone in the respondent’s immediate family have COPD?	Binary. 1 if yes, 0 if no.
Q28.5	Does anyone in the respondent’s immediate family have coronary artery disease?	Binary. 1 if yes, 0 if no.
Q28.6	Does anyone in the respondent’s immediate family have Crohn’s Disease?	Binary. 1 if yes, 0 if no.
Q28.7	Does anyone in the respondent’s immediate family have depression or anxiety?	Binary. 1 if yes, 0 if no.

S1 Table (continued)

Name	Content*	Details
Q28.8	Has anyone in the respondent's immediate family ever had a heart attack?	Binary. 1 if yes, 0 if no.
Q28.9	Does anyone in the respondent's family have high cholesterol?	Binary. 1 if yes, 0 if no.
Q28.10	Does anyone in the respondent's immediate family have hypertension?	Binary. 1 if yes, 0 if no.
Q28.11	Does anyone in the respondent's immediate family have liver disease?	Binary. 1 if yes, 0 if no.
Q28.12	Does anyone in the respondent's immediate family have asthma?	Binary. 1 if yes, 0 if no.
Q28.13	Has anyone in the respondent's immediate family had a stroke?	Binary. 1 if yes, 0 if no.
Q28.14	Does anyone in the respondent's immediate family have diabetes?	Binary. 1 if yes, 0 if no.
Q28.15	Does anyone in the respondent's immediate family have heart disease?	Binary. 1 if yes, 0 if no.
Q28.16	Does anyone in the respondent's immediate family have an irregular heart rhythm?	Binary. 1 if yes, 0 if no.
Q28.17	Does anyone in the respondent's immediate family have an autoimmune disease?	Binary. 1 if yes, 0 if no.
Q28.18	Has anyone in the respondent's immediate family had an organ transplant?	Binary. 1 if yes, 0 if no.
Q117.face	Has the respondent felt persistent or recurrent pain? – Face	Binary. 1 if yes, 0 if no.
Q117.jaw	Has the respondent felt persistent or recurrent pain? – Right or Left Jaw	Binary. 1 if yes, 0 if no.
Q117.breast	Has the respondent felt persistent or recurrent pain? – Right or Left Breast	Binary. 1 if yes, 0 if no.
Q117.arm	Has the respondent felt persistent or recurrent pain? – Right or Left Arm or Elbow	Binary. 1 if yes, 0 if no.
Q117.hand	Has the respondent felt persistent or recurrent pain? – Right or Left Hand or Wrist	Binary. 1 if yes, 0 if no.
Q117.abdomen	Has the respondent felt persistent or recurrent pain? – Abdomen or Pelvis	Binary. 1 if yes, 0 if no.
Q117.groin	Has the respondent felt persistent or recurrent pain? – Right or Left Groin	Binary. 1 if yes, 0 if no.
Q117.leg	Has the respondent felt persistent or recurrent pain? – Right or Left Leg or Knee	Binary. 1 if yes, 0 if no.
Q117.foot	Has the respondent felt persistent or recurrent pain? – Right or Left Ankle or Foot	Binary. 1 if yes, 0 if no.
Q117.head	Has the respondent felt persistent or recurrent pain? – Head	Binary. 1 if yes, 0 if no.
Q117.neck	Has the respondent felt persistent or recurrent pain? – Neck	Binary. 1 if yes, 0 if no.

S1 Table (continued)

Name	Content*	Details
Q117.shoulder	Has the respondent felt persistent or recurrent pain? – Right or Left Shoulder	Binary. 1 if yes, 0 if no.
Q117.back	Has the respondent felt persistent or recurrent pain? – Back	Binary. 1 if yes, 0 if no.
Q117.hip	Has the respondent felt persistent or recurrent pain? – Right or Left Hip	Binary. 1 if yes, 0 if no.
Q117.buttocks	Has the respondent felt persistent or recurrent pain? – Right or Left Buttocks	Binary. 1 if yes, 0 if no.
EHR Variables -		
Any Cancer	At least one of the following phecodes observed: 145, 145.2, 145.3, 145.4, 149, 149.1, 149.2, 149.3, 149.4, 149.5, 149.9, 150, 151, 153, 153.2, 153.3, 155, 155.1, 157, 158, 159, 159.2, 159.3, 159.4, 164, 165, 165.1, 170, 170.1, 170.2, 172, 172.1, 172.11, 172.2, 172.21, 172.22, 172.3, 174, 174.1, 174.11, 175, 180, 180.1, 180.3, 182, 184, 184.1, 184.11, 184.2, 185, 187, 187.1, 187.2, 189, 189.1, 189.11, 189.12, 189.2, 189.21, 189.4, 190, 191, 191.1, 191.11, 193, 194, 195, 195.1, 195.3, 196, 197, 198, 198.1, 198.2, 198.3, 198.4, 198.5, 198.6, 198.7, 199.4, 200, 200.1, 201, 202, 202.2, 202.21, 202.22, 202.23, 202.24, 204, 204.1, 204.11, 204.12, 204.2, 204.21, 204.22, 204.3, 204.4, 209	Binary. Phenomes are truncated differently depending on analysis.<br><br>Survey-based analysis: Codes updated up to March 23rd, 2020, prior to the survey's start. Derived from Michigan Genomics Initiative biorepository, the subset of Michigan Medicine on which the survey data were collected.<br><br>Michigan Medicine EHR Analysis (restricted): Codes updated up to June 22nd, 2020. For the Tested and Diagnosed cohorts, codes truncated to at least 14 days prior to the time of the first test or diagnosis.<br><br>Michigan Medicine EHR Analysis (unrestricted): Codes updated up to June 22nd, 2020. For the Tested and Diagnosed cohorts, codes truncated to at least 0 days prior to the time of the first test or diagnosis.
Respiratory conditions	At least one of the following phecodes observed: 470, 471, 472, 473.1, 474.2, 475, 475.9, 495, 495.1, 495.11, 495.2, 496, 496.1, 496.2, 496.21, 496.3, 499, 500, 500.1, 500.2, 502, 503, 504, 504.1, 505, 506, 507, 508, 509, 509.1, 509.2, 509.3, 509.5, 509.8, 510.2, 513.3, 513.31, 513.32, 513.4, 513.8, 514.1, 514.2, 516, 516.1, 519.1, 519.2, 473.3, 473.4, 478, 512, 513, 514, 519.8, 519.9	
Circulatory conditions	At least one of the following phecodes observed: 94, 394.1, 394.2, 394.3, 394.4, 394.7, 395, 395.1, 395.2, 395.3, 395.4, 395.6, 401, 401.1, 401.2, 401.21, 401.22, 401.3, 411, 411.1, 411.2, 411.3, 411.4, 411.41, 411.8, 411.9, 414, 414.2, 415.2, 415.21, 416, 420.1, 420.22, 420.3, 425, 425.1, 425.11, 425.12, 425.2, 425.8, 426, 426.2, 426.21, 426.22, 426.23, 426.24, 426.25, 426.3, 426.31, 426.32, 426.4, 426.7, 426.8, 426.9, 426.91, 426.92, 427, 427.1, 427.11, 427.12, 427.2, 427.21, 427.22, 427.3, 427.4, 427.41, 427.42, 427.5, 427.6, 427.61, 427.7, 427.8, 427.9, 428, 428.1, 428.2, 428.3, 428.4, 429.1, 430, 433.1, 433.11, 433.12, 433.2, 433.21, 433.3, 433.31, 433.32, 433.5, 433.8, 440, 440.1, 440.2, 440.21, 440.22, 440.9, 441.2, 442, 442.1, 442.11, 442.2, 442.3, 442.4, 442.8, 443, 443.1, 443.7, 443.8, 443.9, 444, 444.1, 444.2, 444.5, 446.1, 446.3, 446.4, 446.5, 446.6, 446.7, 446.8, 446.9, 447, 447.1, 447.7, 448, 450, 451, 451.2, 452, 452.1, 452.2, 452.8, 453, 454, 454.1,454.11, 455, 456, 457, 457.2, 457.3, 458, 458.1, 458.2, 458.9, 459, 459.1, 459.7, 459.9, 396, 418, 429, 430	
Type II Diabetes	At least one of the following phecodes observed: 250.2	
Kidney diseases	At least one of the following phecodes observed: 585	
Liver diseases	At least one of the following phecodes observed: 571	

S1 Table (continued)

Name	Content*	Details
Autoimmune diseases	At least one of the following phecodes observed: 242.1, 250.1, 335, 557.1, 694.1, 695.4, 696.4, 697, 704.1, 714.1, 717	
Comorbidity Score	-	Numeric. Sum of the previous 7 ICD-code derived variables.
Neighborhood Education	Proportion of adults with less than high school diploma, 2010	Numeric. Defined by US census tract based on EHR. Standardized for analysis.
Neighborhood Unemployment	Proportion of 16+ civilian labor force unemployed, 2010	Numeric. Defined by US census tract based on EHR. Standardized for analysis.
Neighborhood Poverty	Proportion of people with income past 12 months below poverty level, 2010	Numeric. Defined by US census tract based on EHR. Standardized for analysis.
Population Density	Persons per square mile, 2010	Numeric. Defined by US census tract based on EHR. Standardized for analysis.
Neighborhood Disadvantage Index	Mean of proportion of Population in Poverty; Unemployed; with Public Assistance Income; and Female-Headed Families with children, 2010	Numeric. Defined by US census tract based on EHR. Standardized for analysis.
Smoking Status	Respondent’s smoking status	Categorical with 3 levels: Never Smoked (reference), Former Smoker, and Current Smoker.
Alcohol	Respondent’s alcohol status.	Binary. 1 if drinker, 0 if non-drinker.

*Each variable is named after the survey question it is derived from, but the variable does not always represent the syntax or nature of that question exactly. Certain survey questions were redefined for simplicity or interpretation.
