# Supplementary material for: Assessing the added value of linking electronic health records to improve the prediction of self-reported COVID-19 testing and diagnosis
Source: PLoS One. 2022 Jul 25;17(7):e0269017. doi: 10.1371/journal.pone.0269017 (PMC9312965; doi:10.1371/journal.pone.0269017)
Supplement: S2 Table — *Controls consisted of randomly selected patients who were alive at the time of extraction, who had an encounter with Michigan Medicine between April 23, 2012, and June 21, 2020, and who were not part of the other cohorts. **Tested cohort includes all patients who were tested for SARS-CoV-2 between March 10th and June 30th of 2020. Diagnosed cohort includes those who tested positive as well as those who were diagnosed by a physician during that span. (PDF) [file pone.0269017.s002.pdf]

S3 Table. Description of Michigan Medicine EHR COVID-19 Cohorts

Variables	Controls* (n = 30,000)	Tested for SARS-CoV-2** (n = 15,929)	Diagnosed with COVID-19** (n = 1,483)
**Numeric, Mean (SD)**			
Age	43.22 (24.36) *n = 29,989*	47.02 (22.84) *n = 15.929*	52.24 (18.69) *n = 1,483*
Body Mass Index	28.48 (7.31) *n = 16,607*	29.66 (7.69) *n = 13,297*	31.86 (9.06) *n = 1,303*
**Categoric, No. (%)**			
Sex			
Male	13,840 (46.13%)	6,769 (42.09%)	678 (45.72%)
Female	16,141 (53.84%)	9,160 (57.51%)	805 (54.28%)
Missing	19 (0.06%)	0 (0.00%)	0 (0.00%)
Race / Ethnicity			
Non-Hispanic Black	2,215 (7.38%)	2,140 (13.43%)	500 (33.72%)
Non-Hispanic White	18,992 (63.31%)	11,404 (71.59%)	692 (46.66%)
Other	2,899 (9.66%)	1,439 (9.03%)	150 (10.11%)
Missing	13,840 (46.13%)	6,769 (42.09%)	678 (45.72%)

*Controls consisted of randomly selected patients who were alive at the time of the extraction, who had an encounter with Michigan Medicine between April 23, 2012 and June 21, 2020, and who were not part of the other cohorts.

**Tested cohort includes all patients who were tested for SARS-CoV-2 between March 10th and June 30th of 2020. Diagnosed cohort includes those who tested positive as well as those who were diagnosed by a physician during that span.
