# Supplementary material for: Assessing the added value of linking electronic health records to improve the prediction of self-reported COVID-19 testing and diagnosis
Source: PLoS One. 2022 Jul 25;17(7):e0269017. doi: 10.1371/journal.pone.0269017 (PMC9312965; doi:10.1371/journal.pone.0269017)
Supplement: S3 Table — All odds ratios are Firth bias-corrected and combined from 30 multiply imputed datasets using Rubin’s Rule’s. †Adjustment 1: Models adjust for Age, Race/Ethnicity, Sex, BMI, Essential Worker Status, and Education as covariates. ‡Adjustment 2: Models additionally adjust for Neighborhood Disadvantage Index. *p Value statistically significant at 1 –α level. **For covariates, α = 0.05. For other variables, α = 0.05 / k, where k = 184 for Adjustment 1 models and k = 183 for Adjustment 2 models. (PDF) [file pone.0269017.s003.pdf]

S4 Table. Single-Predictor Model Odds Ratios for COVID-19 Testing

	Adjustment 1† (Main Analysis)			Adjustment 2‡ (Sensitivity Analysis)		
Variable	OR	1 – α CI**	PV	OR	1 – α CI**	PV
**Covariates**						
Age (per 10 years)	0.99	(0.93, 1.04)	0.291	0.99	(0.94, 1.04)	0.323
Race/Ethnicity – NHB	2.12	(1.45, 3.1)	<0.001*	2.04	(1.38, 3)	<0.001*
Race/Ethnicity – Other	1.4	(0.99, 1.98)	0.029	1.4	(0.99, 1.98)	0.028
Essential Worker	1.65	(1.39, 1.96)	<0.001*	1.64	(1.39, 1.95)	<0.001*
Education – Advanced	1.06	(0.88, 1.27)	0.277	1.06	(0.88, 1.28)	0.259
Education – Associate	1.22	(0.98, 1.52)	0.036	1.21	(0.97, 1.51)	0.042
Education – HS or Less	1.37	(1.11, 1.7)	0.002*	1.35	(1.1, 1.68)	0.003*
Sex	1.2	(1.03, 1.4)	0.011*	1.2	(1.03, 1.4)	0.011*
BMI	1.01	(1, 1.02)	0.094	1.01	(1, 1.02)	0.112
Neighborhood disadvantage	-	-	-	1.04	(0.97, 1.13)	0.131
**Survey Variables**						
Q130	0.88	(0.76, 1.02)	0.049	0.88	(0.76, 1.02)	0.05
Q38	1.04	(0.82, 1.31)	0.379	1.05	(0.83, 1.32)	0.355
Q51	1.17	(0.79, 1.74)	0.222	1.18	(0.79, 1.75)	0.208
Q59	1.4	(0.89, 2.2)	0.072	1.41	(0.9, 2.21)	0.069
Q13	1.38	(1.2, 1.59)	<0.001*	1.38	(1.2, 1.59)	<0.001*
Q46	1.36	(1.09, 1.68)	0.003*	1.36	(1.1, 1.69)	0.002*
Q16	1.08	(1, 1.16)	0.028	1.08	(1, 1.16)	0.028
Q17	1.99	(1.6, 2.48)	<0.001*	1.99	(1.6, 2.47)	<0.001*
Q18	1.02	(1, 1.05)	0.044	1.02	(1, 1.05)	0.046
Q23.1	1.06	(0.91, 1.23)	0.242	1.06	(0.91, 1.23)	0.243
Q23.2	1.27	(1.06, 1.52)	0.004*	1.27	(1.06, 1.51)	0.005*
Q23.3	1.48	(1.2, 1.82)	<0.001*	1.48	(1.2, 1.82)	<0.001*
Q23.4	1.17	(1.01, 1.35)	0.019*	1.17	(1.01, 1.35)	0.019*
Q23.5	1.14	(0.99, 1.33)	0.036	1.14	(0.99, 1.33)	0.036
Q24 – Agree	0.99	(0.82, 1.2)	0.456	0.99	(0.82, 1.19)	0.449
Q24 – Disagree	0.93	(0.75, 1.15)	0.243	0.93	(0.75, 1.15)	0.243
Q27 – Agree	1.21	(0.62, 2.36)	0.29	1.22	(0.62, 2.38)	0.281
Q27 – Disagree	1.25	(0.88, 1.77)	0.11	1.25	(0.88, 1.77)	0.108
Q45 – Agree	1.05	(0.83, 1.31)	0.348	1.05	(0.83, 1.31)	0.351
Q45 – Disagree	0.89	(0.73, 1.07)	0.107	0.89	(0.73, 1.07)	0.108
Q81	1.62	(1.19, 2.21)	0.001*	1.63	(1.2, 2.22)	0.001*
Q85	1.57	(1.13, 2.19)	0.004*	1.58	(1.13, 2.2)	0.003*
Q133	0.88	(0.28, 2.77)	0.414	0.88	(0.28, 2.77)	0.411
Q66 – High	0.94	(0.79, 1.11)	0.233	0.95	(0.8, 1.13)	0.273
Q66 – Low	1.15	(0.92, 1.43)	0.108	1.14	(0.91, 1.41)	0.129
Q150	0.99	(0.93, 1.07)	0.423	1	(0.93, 1.07)	0.49
Q151	0.94	(0.63, 1.4)	0.383	0.92	(0.62, 1.37)	0.347
Q152 – Family-Owned	1.38	(1.03, 1.84)	0.016*	1.37	(1.02, 1.83)	0.017*
Q152 – Other	1.52	(1.03, 2.26)	0.018*	1.5	(1.01, 2.23)	0.022*
Q152 – Rent	1.15	(0.91, 1.46)	0.126	1.13	(0.88, 1.44)	0.168
Q68.1	1.84	(1.57, 2.15)	<0.001*	1.83	(1.56, 2.14)	<0.001*
Q68.2	1.7	(1.4, 2.06)	<0.001*	1.69	(1.39, 2.06)	<0.001*
Q68.3	1.68	(1.43, 1.98)	<0.001*	1.68	(1.43, 1.97)	<0.001*
Q70.1	1.67	(1.42, 1.97)	<0.001*	1.67	(1.42, 1.96)	<0.001*
Q70.2	1.33	(1.14, 1.56)	<0.001*	1.32	(1.13, 1.55)	<0.001*
Q70.3	1.56	(1.34, 1.82)	<0.001*	1.56	(1.33, 1.82)	<0.001*
Q71.1 – Much	2.62	(2.03, 3.38)	<0.001*	2.61	(2.02, 3.37)	<0.001*
Q71.1 – Some	1.7	(1.44, 2)	<0.001*	1.69	(1.43, 2)	<0.001*
Q71.2 – Much	2.43	(1.87, 3.15)	<0.001*	2.42	(1.86, 3.14)	<0.001*
Q71.2 – Some	1.62	(1.37, 1.92)	<0.001*	1.61	(1.36, 1.91)	<0.001*
Q71.3 – Much	2.09	(1.6, 2.72)	<0.001*	2.07	(1.59, 2.71)	<0.001*
Q71.3 – Some	1.65	(1.37, 1.98)	<0.001*	1.64	(1.36, 1.97)	<0.001*
Q71.4 – Much	2.33	(1.72, 3.16)	<0.001*	2.32	(1.71, 3.15)	<0.001*
Q71.4 – Some	1.72	(1.43, 2.06)	<0.001*	1.71	(1.42, 2.05)	<0.001*
Q72.1 – Often	1.2	(0.77, 1.88)	0.208	1.19	(0.76, 1.86)	0.219
Q72.1 – Sometimes	1.36	(1.08, 1.71)	0.004*	1.35	(1.07, 1.7)	0.005*
Q72.2 – Often	1.2	(0.88, 1.65)	0.128	1.19	(0.87, 1.64)	0.14

S4 Table (continued)

	Adjustment 1† (Main Analysis)			Adjustment 2‡ (Sensitivity Analysis)		
Variable	OR	1 – α CI**	PV	OR	1 – α CI**	PV
Q72.2 – Sometimes	1.3	(1.06, 1.59)	0.005*	1.29	(1.06, 1.58)	0.006*
Q72.3 – Often	1.48	(1.14, 1.91)	0.001*	1.47	(1.14, 1.89)	0.002*
Q72.3 – Sometimes	1.28	(1.07, 1.53)	0.003*	1.28	(1.07, 1.53)	0.003*
Q72.4 – Often	1.08	(0.76, 1.56)	0.33	1.08	(0.75, 1.54)	0.347
Q72.4 – Sometimes	1.22	(0.98, 1.52)	0.036	1.21	(0.98, 1.51)	0.041
Q74.1 – Often	1.47	(1.09, 1.99)	0.006*	1.46	(1.08, 1.98)	0.007*
Q74.1 – Sometimes	1.16	(0.95, 1.4)	0.068	1.16	(0.95, 1.4)	0.071
Q74.2 – Often	1.31	(0.94, 1.83)	0.054	1.3	(0.94, 1.82)	0.059
Q74.2 – Sometimes	1.37	(1.11, 1.69)	0.002*	1.36	(1.1, 1.68)	0.002*
Q74.3 – Often	1.52	(1.09, 2.1)	0.006*	1.51	(1.09, 2.09)	0.007*
Q74.3 – Sometimes	1.44	(1.17, 1.78)	<0.001*	1.44	(1.17, 1.77)	<0.001*
Q74.4 – Often	1.26	(0.97, 1.64)	0.043	1.25	(0.96, 1.63)	0.047
Q74.4 – Sometimes	1.18	(0.98, 1.41)	0.038	1.17	(0.98, 1.4)	0.039
Q77	1.6	(1.34, 1.92)	<0.001*	1.59	(1.33, 1.91)	<0.001*
Q80.1 – Often	0.77	(0.64, 0.93)	0.003*	0.77	(0.64, 0.94)	0.004*
Q80.1 – Sometimes	0.99	(0.73, 1.34)	0.473	0.99	(0.73, 1.34)	0.475
Q80.2 – Often	0.9	(0.71, 1.14)	0.193	0.91	(0.71, 1.15)	0.212
Q80.2 – Sometimes	1.15	(0.81, 1.64)	0.221	1.15	(0.81, 1.64)	0.213
Q80.3 – Often	0.89	(0.72, 1.1)	0.139	0.9	(0.72, 1.11)	0.155
Q80.3 – Sometimes	1.29	(0.96, 1.73)	0.045	1.29	(0.96, 1.73)	0.044
Q80.4 – Often	0.95	(0.74, 1.21)	0.331	0.95	(0.75, 1.22)	0.357
Q80.4 – Sometimes	1.28	(0.93, 1.78)	0.067	1.29	(0.93, 1.78)	0.064
Q141	1.31	(0.8, 2.15)	0.142	1.32	(0.8, 2.17)	0.136
Q145	1.28	(1.07, 1.53)	0.004*	1.27	(1.07, 1.53)	0.004*
Q146	1.42	(1.22, 1.66)	<0.001*	1.42	(1.22, 1.66)	<0.001*
Q147	1.96	(1.6, 2.41)	<0.001*	1.95	(1.59, 2.4)	<0.001*
Q125	1.51	(1.29, 1.77)	<0.001*	1.51	(1.29, 1.77)	<0.001*
Q127	1.17	(0.95, 1.44)	0.07	1.17	(0.95, 1.44)	0.072
Q40 – Current User	1.34	(1.01, 1.78)	0.021*	1.33	(1, 1.77)	0.025
Q40 – Former User	1.1	(0.94, 1.3)	0.118	1.1	(0.93, 1.3)	0.126
Q114.1	1.09	(1.06, 1.12)	<0.001*	1.09	(1.06, 1.12)	<0.001*
Q114.2	1.11	(1.08, 1.15)	<0.001*	1.11	(1.07, 1.15)	<0.001*
Q56.1	0.99	(0.97, 1.02)	0.297	0.99	(0.97, 1.02)	0.303
Q56.2	1	(0.99, 1)	0.346	1	(0.99, 1)	0.352
Q88 – Monthly	1.05	(0.78, 1.42)	0.38	1.05	(0.78, 1.42)	0.38
Alcohol – Weekly or More	0.88	(0.69, 1.12)	0.15	0.88	(0.69, 1.12)	0.149
Q38.1	1.17	(0.7, 1.95)	0.28	1.18	(0.7, 1.98)	0.265
Q38.2	1.04	(0.68, 1.59)	0.43	1.05	(0.68, 1.6)	0.416
Q59.1	4.02	(1.67, 9.68)	0.001*	4.02	(1.67, 9.67)	0.001*
Q59.2	1.78	(0.67, 4.68)	0.123	1.78	(0.67, 4.69)	0.123
Q59.3	1.71	(0.7, 4.18)	0.118	1.74	(0.71, 4.24)	0.112
Q59.4	1.3	(0.32, 5.3)	0.356	1.33	(0.33, 5.4)	0.345
Q145.1	1.81	(0.63, 5.25)	0.136	1.75	(0.6, 5.07)	0.151
Q145.2	1.49	(1.13, 1.96)	0.003*	1.48	(1.12, 1.96)	0.003*
Q145.3	1.9	(1.23, 2.95)	0.002*	1.9	(1.22, 2.94)	0.002*
Q145.4	0.89	(0.16, 5.09)	0.447	0.89	(0.15, 5.07)	0.446
Q145.5	1.15	(0.93, 1.42)	0.095	1.15	(0.93, 1.42)	0.098
Q145.6	1.2	(0.95, 1.52)	0.063	1.2	(0.95, 1.52)	0.066
Q146.1	1.25	(1.03, 1.51)	0.013*	1.24	(1.02, 1.51)	0.014*
Q146.2	2.14	(1.56, 2.96)	<0.001*	2.13	(1.54, 2.93)	<0.001*
Q146.3	3.22	(0.42, 24.53)	0.129	3.26	(0.43, 24.85)	0.127
Q146.4	2.3	(1.29, 4.11)	0.002*	2.28	(1.28, 4.07)	0.003*
Q146.5	1.34	(1.12, 1.61)	0.001*	1.34	(1.12, 1.61)	0.001*
Q146.6	1.35	(1.11, 1.64)	0.001*	1.35	(1.11, 1.63)	0.001*
Q147.1	2.24	(1.76, 2.86)	<0.001*	2.22	(1.74, 2.84)	<0.001*
Q147.2	1.97	(1.35, 2.87)	<0.001*	1.95	(1.34, 2.84)	<0.001*
Q147.3	0.97	(0.54, 1.74)	0.463	0.97	(0.54, 1.74)	0.464
Q147.4	0.72	(0.3, 1.73)	0.229	0.71	(0.29, 1.72)	0.226
Q125.1	1.53	(1.01, 2.33)	0.022*	1.53	(1.01, 2.32)	0.023*

S4 Table (continued)

	Adjustment 1† (Main Analysis)			Adjustment 2‡ (Sensitivity Analysis)		
Variable	OR	1 – α CI**	PV	OR	1 – α CI**	PV
Q125.2	1.68	(1.02, 2.78)	0.021*	1.67	(1.01, 2.75)	0.023*
Q125.3	1.61	(1.09, 2.36)	0.008*	1.61	(1.09, 2.36)	0.008*
Q125.4	1.13	(0.96, 1.34)	0.074	1.13	(0.96, 1.34)	0.075
Q125.5	1.27	(0.77, 2.1)	0.176	1.27	(0.77, 2.09)	0.178
Q125.6	1.16	(0.65, 2.07)	0.311	1.16	(0.65, 2.07)	0.312
Q125.7	2.07	(1.52, 2.82)	<0.001*	2.06	(1.51, 2.81)	<0.001*
Q125.8	1.28	(0.83, 1.97)	0.132	1.27	(0.83, 1.96)	0.137
Q125.9	1.36	(1.04, 1.76)	0.011*	1.36	(1.04, 1.76)	0.011*
Q127.1	0.88	(0.33, 2.35)	0.396	0.88	(0.33, 2.36)	0.399
Q127.2	1.06	(0.55, 2.04)	0.43	1.06	(0.55, 2.03)	0.435
Q127.3	1.1	(0.79, 1.54)	0.282	1.1	(0.79, 1.54)	0.283
Q127.4	1.06	(0.79, 1.41)	0.353	1.05	(0.79, 1.41)	0.366
Q127.5	1	(0.76, 1.3)	0.495	1	(0.76, 1.3)	0.489
Q36.live.alone	1.33	(1.08, 1.64)	0.004*	1.32	(1.07, 1.63)	0.005*
Q36.house.diagnose	3.38	(2.36, 4.85)	<0.001*	3.38	(2.35, 4.84)	<0.001*
Q18.G – Detractor	0.92	(0.78, 1.09)	0.176	0.92	(0.78, 1.09)	0.177
Q18.G – Promoter	1.11	(0.89, 1.39)	0.176	1.11	(0.89, 1.39)	0.18
Q126.1	2.17	(0.75, 6.28)	0.077	2.14	(0.74, 6.19)	0.081
Q126.2	1.63	(1.14, 2.32)	0.004*	1.62	(1.13, 2.3)	0.004*
Q118.1	0.82	(0.68, 0.99)	0.018*	0.82	(0.68, 0.99)	0.02*
Q118.2	0.92	(0.75, 1.13)	0.215	0.92	(0.75, 1.13)	0.216
Q118.3	0.9	(0.55, 1.48)	0.341	0.89	(0.54, 1.47)	0.33
Q118.4	1.5	(0.99, 2.25)	0.026	1.48	(0.98, 2.23)	0.03
Q118.5	0.96	(0.78, 1.18)	0.341	0.96	(0.78, 1.18)	0.345
Q118.6	1.08	(0.91, 1.27)	0.184	1.08	(0.92, 1.28)	0.177
Q118.7	1.08	(0.92, 1.26)	0.181	1.08	(0.92, 1.26)	0.181
Q133.1	3	(1.72, 5.24)	<0.001*	2.96	(1.7, 5.17)	<0.001*
Q133.2	2.05	(1.37, 3.08)	<0.001*	2.04	(1.36, 3.06)	<0.001*
Q133.3	1.2	(0.83, 1.74)	0.163	1.19	(0.83, 1.72)	0.174
Q28.1	0.93	(0.7, 1.23)	0.301	0.93	(0.7, 1.22)	0.294
Q28.2	1.05	(0.89, 1.23)	0.284	1.05	(0.89, 1.23)	0.282
Q28.3	1.08	(0.86, 1.34)	0.26	1.07	(0.86, 1.34)	0.267
Q28.4	1.19	(1.01, 1.42)	0.021*	1.19	(1, 1.41)	0.023*
Q28.5	1.07	(0.92, 1.25)	0.189	1.07	(0.92, 1.25)	0.19
Q28.6	1.4	(1, 1.97)	0.025	1.4	(1, 1.97)	0.025
Q28.7	1.19	(1.02, 1.38)	0.012*	1.19	(1.02, 1.38)	0.012*
Q28.8	1.14	(0.99, 1.32)	0.039	1.14	(0.99, 1.32)	0.039
Q28.9	1.09	(0.94, 1.26)	0.137	1.09	(0.94, 1.26)	0.135
Q28.10	1.08	(0.93, 1.26)	0.148	1.08	(0.93, 1.26)	0.152
Q28.11	1.19	(0.9, 1.56)	0.108	1.19	(0.9, 1.56)	0.112
Q28.12	1.09	(0.92, 1.3)	0.156	1.09	(0.92, 1.29)	0.164
Q28.13	1.07	(0.92, 1.25)	0.183	1.07	(0.92, 1.25)	0.18
Q28.14	1.02	(0.88, 1.18)	0.417	1.01	(0.87, 1.17)	0.432
Q28.15	1.24	(1.07, 1.43)	0.002*	1.24	(1.07, 1.43)	0.002*
Q28.16	1.2	(1.01, 1.43)	0.019*	1.2	(1.01, 1.43)	0.019*
Q28.17	1	(0.79, 1.26)	0.487	0.99	(0.79, 1.26)	0.483
Q28.18	0.76	(0.45, 1.29)	0.156	0.76	(0.44, 1.29)	0.152
Q117.face	1.34	(0.88, 2.05)	0.087	1.33	(0.87, 2.03)	0.093
Q117.jaw	1.59	(1.19, 2.13)	0.001*	1.57	(1.17, 2.11)	0.001*
Q117.breast	1.62	(1.16, 2.25)	0.002*	1.6	(1.15, 2.23)	0.003*
Q117.arm	1.27	(1.02, 1.58)	0.016*	1.26	(1.01, 1.57)	0.02*
Q117.hand	1.2	(0.99, 1.45)	0.03	1.19	(0.99, 1.44)	0.034
Q117.abdomen	1.74	(1.39, 2.18)	<0.001*	1.73	(1.39, 2.17)	<0.001*
Q117.groin	0.95	(0.68, 1.34)	0.393	0.95	(0.67, 1.34)	0.385
Q117.leg	1.22	(1.04, 1.43)	0.006*	1.22	(1.04, 1.42)	0.007*
Q117.foot	1.23	(1.03, 1.47)	0.01*	1.23	(1.03, 1.47)	0.011*
Q117.head	1.43	(1.13, 1.8)	0.001*	1.42	(1.13, 1.79)	0.002*
Q117.neck	1.26	(1.05, 1.5)	0.006*	1.25	(1.05, 1.49)	0.007*
Q117.shoulder	1.22	(1.03, 1.44)	0.012*	1.21	(1.02, 1.44)	0.014*

S4 Table (continued)

	Adjustment 1† (Main Analysis)			Adjustment 2‡ (Sensitivity Analysis)		
Variable	OR	1 – α CI**	PV	OR	1 – α CI**	PV
Q117.back	1.19	(1.02, 1.38)	0.012*	1.19	(1.02, 1.38)	0.013*
Q117.hip	1.15	(0.97, 1.36)	0.056	1.14	(0.96, 1.35)	0.061
Q117.buttocks	1.12	(0.87, 1.44)	0.196	1.11	(0.86, 1.43)	0.206
**EHR Variables**						
Respiratory Condition	1.33	(1.14, 1.56)	<0.001*	1.34	(1.14, 1.57)	<0.001*
Circulatory Condition	1.26	(1.03, 1.56)	0.013*	1.27	(1.03, 1.56)	0.013*
Any Cancer	0.95	(0.81, 1.1)	0.236	0.94	(0.81, 1.1)	0.23
Type II Diabetes	1.21	(1.01, 1.45)	0.02*	1.21	(1.01, 1.45)	0.021*
Kidney Disease	1.54	(1.27, 1.86)	<0.001*	1.53	(1.26, 1.86)	<0.001*
Liver Disease	1.43	(1.15, 1.77)	0.001*	1.42	(1.15, 1.77)	0.001*
Autoimmune Disease	1.22	(1.03, 1.45)	0.011*	1.23	(1.03, 1.46)	0.009*
Comorbidity Score	1.14	(1.08, 1.2)	<0.001*	1.14	(1.08, 1.2)	<0.001*
Smoker – Past	1.2	(1.02, 1.42)	0.015*	1.2	(1.02, 1.42)	0.016*
Smoker – Current	1.29	(1.01, 1.66)	0.022*	1.28	(1, 1.65)	0.026
Drinker	0.95	(0.8, 1.14)	0.29	0.96	(0.8, 1.14)	0.315
Neighborhood Education	1.04	(0.96, 1.13)	0.174	1.02	(0.92, 1.13)	0.381
Neighborhood Unemployment	1.05	(0.97, 1.13)	0.104	1.04	(0.94, 1.14)	0.231
Neighborhood Disadvantage	1.04	(0.97, 1.13)	0.131	-	-	-
Population Density	1.05	(0.98, 1.14)	0.089	1.04	(0.96, 1.13)	0.146
Neighborhood Poverty	1.01	(0.94, 1.09)	0.388	0.9	(0.77, 1.05)	0.095

All odds ratios are Firth bias-corrected and combined from 30 multiply imputed datasets using Rubin's Rule's. Complete variable descriptions are available in the supplement (S1 Table).

†Adjustment 1: Models adjust for Age, Race/Ethnicity, Sex, BMI, Essential Worker Status, and Education as covariates.

‡Adjustment 2: Models additionally adjust for Neighborhood Disadvantage Index.

*p Value statistically significant at 1 – α level.

**For covariates, α = 0.05. For other variables, α = 0.05 / k, where k = 184 for Adjustment 1 models and k = 183 for Adjustment 2 models
