# Supplementary material for: Assessing the added value of linking electronic health records to improve the prediction of self-reported COVID-19 testing and diagnosis
Source: PLoS One. 2022 Jul 25;17(7):e0269017. doi: 10.1371/journal.pone.0269017 (PMC9312965; doi:10.1371/journal.pone.0269017)
Supplement: S4 Table — All odds ratios are Firth bias-corrected and combined from 30 multiply imputed datasets using Rubin’s Rule’s. †Adjustment 1: Models adjust for Age, Race/Ethnicity, Sex, BMI, Essential Worker Status, and Education as covariates. ‡Adjustment 2: Models additionally adjust for Neighborhood Disadvantage Index. *p value statistically significant at 1 –α level. **For covariates, α = 0.05. For other variables, α = 0.05 / k, where k = 184 for Adjustment 1 models and k = 183 for Adjustment 2 models. (PDF) [file pone.0269017.s004.pdf]

S5 Table. Single-Predictor Model Odds Ratios for COVID-19 Diagnosis

	Adjustment 1† (Main Analysis)			Adjustment 2‡ (Sensitivity Analysis)		
Variable	OR	1 – α CI**	PV	OR	1 – α CI**	PV
**Covariates**						
Age (per 10 years)	0.75	(0.64, 0.87)	<0.001*	0.75	(0.64, 0.87)	<0.001*
Race/Ethnicity – NHB	3.27	(1.4, 7.62)	0.003*	3.27	(1.38, 7.76)	0.004*
Race/Ethnicity – Other	1.87	(0.82, 4.26)	0.068	1.87	(0.82, 4.25)	0.068
Essential Worker	2.7	(1.7, 4.29)	<0.001*	2.7	(1.7, 4.28)	<0.001*
Education – Advanced	1.19	(0.67, 2.11)	0.273	1.19	(0.67, 2.1)	0.275
Education – Associate	1.25	(0.64, 2.42)	0.257	1.24	(0.64, 2.41)	0.259
Education – HS or Less	1.43	(0.75, 2.7)	0.139	1.42	(0.75, 2.7)	0.141
Sex	0.97	(0.61, 1.55)	0.45	0.97	(0.61, 1.55)	0.451
BMI	1.01	(0.98, 1.04)	0.32	1.01	(0.98, 1.04)	0.322
Neighborhood disadvantage	-	-	-	1.01	(0.81, 1.26)	0.479
**Survey Variables**						
Q130	0.66	(0.42, 1.05)	0.041	0.66	(0.42, 1.05)	0.041
Q38	1.33	(0.72, 2.44)	0.179	1.33	(0.72, 2.44)	0.179
Q51	1.78	(0.74, 4.31)	0.101	1.78	(0.74, 4.31)	0.1
Q59	2.83	(1.16, 6.93)	0.011*	2.83	(1.16, 6.92)	0.011*
Q13	1.16	(0.75, 1.79)	0.253	1.16	(0.75, 1.79)	0.252
Q46	1	(0.57, 1.75)	0.497	1	(0.57, 1.74)	0.498
Q16	0.91	(0.72, 1.15)	0.223	0.91	(0.72, 1.15)	0.223
Q17	2.18	(1.16, 4.09)	0.008*	2.18	(1.16, 4.08)	0.008*
Q18	1.03	(0.96, 1.12)	0.205	1.03	(0.96, 1.12)	0.205
Q23.1	0.85	(0.54, 1.33)	0.24	0.85	(0.55, 1.33)	0.239
Q23.2	1.23	(0.74, 2.04)	0.211	1.23	(0.74, 2.04)	0.21
Q23.3	1.5	(0.86, 2.61)	0.078	1.5	(0.86, 2.6)	0.077
Q23.4	1.72	(1.08, 2.72)	0.011*	1.72	(1.08, 2.72)	0.011*
Q23.5	1.22	(0.78, 1.9)	0.192	1.22	(0.78, 1.89)	0.192
Q24 – Agree	0.71	(0.41, 1.21)	0.105	0.71	(0.41, 1.21)	0.104
Q24 – Disagree	0.73	(0.4, 1.31)	0.144	0.73	(0.4, 1.31)	0.143
Q27 – Agree	5.02	(0.99, 25.58)	0.026	5.02	(0.99, 25.54)	0.026
Q27 – Disagree	1.47	(0.42, 5.17)	0.274	1.47	(0.42, 5.16)	0.273
Q45 – Agree	0.84	(0.43, 1.62)	0.298	0.84	(0.43, 1.61)	0.296
Q45 – Disagree	0.79	(0.45, 1.38)	0.202	0.79	(0.45, 1.38)	0.201
Q81	8.7	(5.07, 14.93)	<0.001*	8.68	(5.06, 14.9)	<0.001*
Q85	9.38	(5.39, 16.35)	<0.001*	9.37	(5.38, 16.3)	<0.001*
Q133	0.87	(0.05, 15.18)	0.463	0.88	(0.05, 15.01)	0.464
Q66 – High	1.56	(0.95, 2.55)	0.039	1.59	(0.97, 2.61)	0.034
Q66 – Low	0.66	(0.3, 1.48)	0.157	0.65	(0.29, 1.45)	0.146
Q150	1.08	(0.89, 1.32)	0.215	1.08	(0.89, 1.32)	0.212
Q151	2.03	(0.88, 4.65)	0.047	2.04	(0.89, 4.69)	0.047
Q152 – Family-Owned	1.49	(0.71, 3.14)	0.145	1.49	(0.71, 3.13)	0.146
Q152 – Other	3.62	(1.56, 8.4)	0.001*	3.61	(1.55, 8.37)	0.001*
Q152 – Rent	0.64	(0.3, 1.38)	0.129	0.64	(0.3, 1.38)	0.126
Q68.1	1.33	(0.82, 2.18)	0.125	1.34	(0.82, 2.18)	0.124
Q68.2	1.71	(0.99, 2.97)	0.028	1.71	(0.99, 2.97)	0.028
Q68.3	1.42	(0.88, 2.28)	0.076	1.42	(0.88, 2.28)	0.075
Q70.1	2.69	(1.71, 4.23)	<0.001*	2.7	(1.72, 4.24)	<0.001*
Q70.2	1.02	(0.63, 1.65)	0.463	1.02	(0.63, 1.65)	0.463
Q70.3	2.45	(1.48, 4.06)	<0.001*	2.45	(1.49, 4.05)	<0.001*
Q71.1 – Much	3.65	(1.67, 7.99)	0.001*	3.66	(1.67, 8)	0.001*
Q71.1 – Some	1.99	(1.21, 3.25)	0.003*	2	(1.22, 3.27)	0.003*
Q71.2 – Much	3.52	(1.53, 8.08)	0.002*	3.52	(1.54, 8.08)	0.001*
Q71.2 – Some	2.29	(1.39, 3.77)	0.001*	2.3	(1.39, 3.79)	0.001*
Q71.3 – Much	4.31	(2.05, 9.07)	<0.001*	4.35	(2.06, 9.16)	<0.001*
Q71.3 – Some	2.47	(1.44, 4.24)	<0.001*	2.48	(1.45, 4.26)	<0.001*
Q71.4 – Much	2.52	(0.94, 6.73)	0.033	2.52	(0.95, 6.73)	0.032
Q71.4 – Some	2.05	(1.19, 3.51)	0.005*	2.06	(1.2, 3.52)	0.004*
Q72.1 – Often	1.11	(0.31, 3.98)	0.439	1.11	(0.31, 3.97)	0.438
Q72.1 – Sometimes	1.89	(1.05, 3.4)	0.017*	1.89	(1.05, 3.4)	0.017*
Q72.2 – Often	1.11	(0.46, 2.71)	0.408	1.11	(0.46, 2.71)	0.407

S5 Table (continued)

	Adjustment 1† (Main Analysis)			Adjustment 2‡ (Sensitivity Analysis)		
Variable	OR	1 – α CI**	PV	OR	1 – α CI**	PV
Q72.2 – Sometimes	1.46	(0.83, 2.58)	0.096	1.46	(0.83, 2.58)	0.096
Q72.3 – Often	1.15	(0.55, 2.43)	0.356	1.15	(0.55, 2.42)	0.356
Q72.3 – Sometimes	1.03	(0.59, 1.78)	0.462	1.03	(0.59, 1.77)	0.462
Q72.4 – Often	0.76	(0.26, 2.25)	0.311	0.76	(0.26, 2.24)	0.31
Q72.4 – Sometimes	0.85	(0.43, 1.7)	0.326	0.85	(0.43, 1.7)	0.324
Q74.1 – Often	0.91	(0.35, 2.35)	0.422	0.91	(0.35, 2.35)	0.422
Q74.1 – Sometimes	0.92	(0.51, 1.67)	0.394	0.92	(0.51, 1.67)	0.393
Q74.2 – Often	1.03	(0.42, 2.58)	0.471	1.03	(0.42, 2.57)	0.471
Q74.2 – Sometimes	1.23	(0.67, 2.26)	0.25	1.23	(0.67, 2.26)	0.25
Q74.3 – Often	1.42	(0.6, 3.37)	0.213	1.42	(0.6, 3.36)	0.214
Q74.3 – Sometimes	1.85	(1.07, 3.22)	0.014*	1.85	(1.07, 3.22)	0.014*
Q74.4 – Often	0.94	(0.41, 2.18)	0.443	0.94	(0.41, 2.18)	0.443
Q74.4 – Sometimes	1.71	(1.05, 2.79)	0.016*	1.71	(1.05, 2.79)	0.016*
Q77	1.28	(0.74, 2.21)	0.187	1.28	(0.74, 2.21)	0.186
Q80.1 – Often	1.01	(0.55, 1.85)	0.484	1.01	(0.55, 1.85)	0.483
Q80.1 – Sometimes	0.78	(0.27, 2.26)	0.324	0.78	(0.27, 2.26)	0.323
Q80.2 – Often	0.7	(0.37, 1.32)	0.136	0.7	(0.37, 1.32)	0.136
Q80.2 – Sometimes	0.37	(0.1, 1.42)	0.073	0.37	(0.1, 1.41)	0.073
Q80.3 – Often	0.84	(0.46, 1.55)	0.292	0.84	(0.46, 1.55)	0.292
Q80.3 – Sometimes	0.7	(0.26, 1.88)	0.237	0.69	(0.26, 1.87)	0.235
Q80.4 – Often	0.88	(0.44, 1.74)	0.353	0.88	(0.44, 1.74)	0.353
Q80.4 – Sometimes	0.56	(0.18, 1.7)	0.152	0.56	(0.18, 1.69)	0.152
Q141	1.03	(0.34, 3.08)	0.479	1.03	(0.35, 3.07)	0.479
Q145	0.8	(0.43, 1.5)	0.247	0.8	(0.43, 1.5)	0.247
Q146	1.79	(1.13, 2.84)	0.007*	1.79	(1.13, 2.84)	0.007*
Q147	1.72	(0.89, 3.33)	0.053	1.72	(0.89, 3.33)	0.052
Q125	1.02	(0.61, 1.7)	0.468	1.02	(0.61, 1.7)	0.468
Q127	0.77	(0.34, 1.71)	0.259	0.77	(0.35, 1.71)	0.258
Q40 – Current User	0.72	(0.27, 1.9)	0.255	0.72	(0.27, 1.9)	0.254
Q40 – Former User	1.06	(0.63, 1.77)	0.417	1.06	(0.63, 1.77)	0.417
Q114.1	1.02	(0.94, 1.1)	0.338	1.02	(0.94, 1.1)	0.338
Q114.2	1.04	(0.94, 1.14)	0.244	1.04	(0.94, 1.14)	0.243
Q56.1	1.08	(1, 1.16)	0.026	1.08	(1, 1.16)	0.026
Q56.2	1.01	(1, 1.03)	0.015*	1.01	(1, 1.03)	0.014*
Q88 – Monthly	0.68	(0.26, 1.76)	0.213	0.68	(0.26, 1.76)	0.212
Alcohol – Weekly or More	0.89	(0.45, 1.76)	0.368	0.89	(0.45, 1.76)	0.369
Q38.1	2.27	(0.75, 6.86)	0.074	2.27	(0.75, 6.86)	0.074
Q38.2	1.02	(0.29, 3.59)	0.489	1.02	(0.29, 3.59)	0.489
Q59.1	12.82	(3.03, 54.3)	<0.001*	12.8	(3.02, 54.14)	<0.001*
Q59.2	7.91	(1.94, 32.2)	0.002*	7.9	(1.94, 32.11)	0.002*
Q59.3	5.7	(1.44, 22.54)	0.007*	5.7	(1.44, 22.58)	0.007*
Q59.4	2.67	(0.14, 50.09)	0.256	2.67	(0.14, 50.06)	0.256
Q145.1	5.67	(0.95, 33.87)	0.029	5.76	(0.96, 34.38)	0.027
Q145.2	0.88	(0.3, 2.53)	0.404	0.88	(0.3, 2.53)	0.404
Q145.3	0.93	(0.19, 4.72)	0.467	0.93	(0.19, 4.7)	0.467
Q145.4	4.34	(0.23, 80.97)	0.163	4.33	(0.23, 80.34)	0.163
Q145.5	0.98	(0.49, 1.94)	0.472	0.98	(0.49, 1.93)	0.472
Q145.6	1.24	(0.59, 2.6)	0.285	1.24	(0.59, 2.59)	0.285
Q146.1	1.56	(0.91, 2.67)	0.052	1.56	(0.91, 2.66)	0.052
Q146.2	2.02	(0.68, 6.04)	0.103	2.03	(0.68, 6.04)	0.102
Q146.3	10.54	(0.37, 296.74)	0.083	10.49	(0.37, 295.39)	0.084
Q146.4	6.12	(1.63, 22.93)	0.004*	6.12	(1.64, 22.9)	0.004*
Q146.5	1.66	(0.96, 2.9)	0.036	1.66	(0.96, 2.89)	0.036
Q146.6	1.68	(0.93, 3.03)	0.042	1.68	(0.93, 3.02)	0.042
Q147.1	1.5	(0.63, 3.61)	0.181	1.5	(0.63, 3.61)	0.181
Q147.2	4.26	(1.83, 9.89)	<0.001*	4.26	(1.84, 9.89)	<0.001*
Q147.3	0.43	(0.03, 6.78)	0.274	0.43	(0.03, 6.73)	0.273
Q147.4	0.81	(0.05, 13.18)	0.44	0.81	(0.05, 13.09)	0.439
Q125.1	0.37	(0.02, 5.89)	0.241	0.37	(0.02, 5.85)	0.24

S5 Table (continued)

	Adjustment 1† (Main Analysis)			Adjustment 2‡ (Sensitivity Analysis)		
Variable	OR	1 – α CI**	PV	OR	1 – α CI**	PV
Q125.2	0.72	(0.04, 11.74)	0.41	0.72	(0.04, 11.64)	0.41
Q125.3	1.66	(0.47, 5.91)	0.217	1.66	(0.47, 5.89)	0.216
Q125.4	0.73	(0.4, 1.31)	0.144	0.73	(0.41, 1.31)	0.143
Q125.5	1.5	(0.3, 7.65)	0.312	1.5	(0.3, 7.62)	0.312
Q125.6	2.26	(0.44, 11.54)	0.164	2.25	(0.44, 11.48)	0.164
Q125.7	2.88	(1.27, 6.54)	0.006*	2.88	(1.27, 6.53)	0.006*
Q125.8	2.38	(0.79, 7.1)	0.061	2.37	(0.79, 7.08)	0.061
Q125.9	2.14	(1.03, 4.42)	0.02*	2.14	(1.04, 4.41)	0.02*
Q127.1	1.47	(0.09, 24.42)	0.394	1.47	(0.09, 24.27)	0.393
Q127.2	0.78	(0.05, 12.37)	0.429	0.78	(0.05, 12.26)	0.429
Q127.3	1.28	(0.44, 3.74)	0.325	1.28	(0.44, 3.73)	0.325
Q127.4	0.89	(0.31, 2.59)	0.417	0.89	(0.31, 2.59)	0.417
Q127.5	0.65	(0.22, 1.87)	0.21	0.65	(0.22, 1.86)	0.209
Q36.live.alone	1.07	(0.53, 2.17)	0.426	1.07	(0.53, 2.17)	0.425
Q36.house.diagnose	38.61	(22.75, 65.52)	<0.001*	38.38	(22.63, 65.11)	<0.001*
Q18.G – Detractor	1.26	(0.72, 2.21)	0.21	1.26	(0.72, 2.21)	0.209
Q18.G – Promoter	1.87	(0.93, 3.78)	0.04	1.87	(0.93, 3.78)	0.04
Q126.1	13.34	(2.3, 77.18)	0.002*	13.32	(2.31, 76.86)	0.002*
Q126.2	0.75	(0.15, 3.74)	0.365	0.76	(0.15, 3.73)	0.365
Q118.1	0.54	(0.29, 1.01)	0.027	0.54	(0.29, 1.01)	0.027
Q118.2	0.85	(0.47, 1.54)	0.3	0.85	(0.47, 1.54)	0.3
Q118.3	1.75	(0.66, 4.66)	0.132	1.75	(0.66, 4.65)	0.132
Q118.4	1.02	(0.29, 3.64)	0.487	1.02	(0.29, 3.63)	0.487
Q118.5	0.45	(0.21, 1)	0.025	0.45	(0.21, 1)	0.025*
Q118.6	1.15	(0.7, 1.87)	0.289	1.15	(0.71, 1.87)	0.29
Q118.7	1.1	(0.7, 1.75)	0.337	1.1	(0.7, 1.75)	0.338
Q133.1	4.37	(1.13, 16.97)	0.016*	4.41	(1.13, 17.15)	0.016*
Q133.2	1.4	(0.39, 5.01)	0.305	1.39	(0.39, 5)	0.305
Q133.3	1.77	(0.77, 4.05)	0.089	1.77	(0.77, 4.05)	0.088
Q28.1	0.65	(0.34, 1.25)	0.098	0.65	(0.34, 1.25)	0.098
Q28.2	1.14	(0.7, 1.83)	0.303	1.14	(0.7, 1.83)	0.302
Q28.3	0.77	(0.37, 1.63)	0.248	0.77	(0.37, 1.63)	0.248
Q28.4	0.77	(0.43, 1.39)	0.193	0.77	(0.43, 1.38)	0.192
Q28.5	0.77	(0.46, 1.3)	0.165	0.77	(0.46, 1.3)	0.164
Q28.6	0.17	(0.01, 2.6)	0.101	0.17	(0.01, 2.58)	0.101
Q28.7	0.62	(0.39, 0.99)	0.022*	0.62	(0.39, 0.99)	0.022*
Q28.8	0.72	(0.45, 1.14)	0.079	0.72	(0.45, 1.14)	0.078
Q28.9	0.85	(0.55, 1.32)	0.236	0.85	(0.55, 1.32)	0.235
Q28.10	0.78	(0.5, 1.21)	0.134	0.78	(0.5, 1.21)	0.134
Q28.11	0.85	(0.33, 2.19)	0.368	0.85	(0.33, 2.18)	0.367
Q28.12	0.87	(0.52, 1.47)	0.305	0.87	(0.52, 1.47)	0.305
Q28.13	0.97	(0.61, 1.55)	0.45	0.97	(0.61, 1.54)	0.449
Q28.14	1	(0.64, 1.56)	0.497	1	(0.64, 1.56)	0.498
Q28.15	1	(0.64, 1.57)	0.498	1	(0.64, 1.56)	0.497
Q28.16	1.52	(0.92, 2.52)	0.051	1.52	(0.92, 2.52)	0.051
Q28.17	0.85	(0.4, 1.79)	0.332	0.85	(0.4, 1.79)	0.331
Q28.18	1.24	(0.35, 4.37)	0.368	1.24	(0.35, 4.36)	0.368
Q117.face	2.02	(0.76, 5.42)	0.081	2.02	(0.75, 5.41)	0.081
Q117.jaw	1.2	(0.46, 3.13)	0.352	1.2	(0.46, 3.12)	0.352
Q117.breast	3.12	(1.45, 6.73)	0.002*	3.12	(1.45, 6.72)	0.002*
Q117.arm	1.57	(0.81, 3.02)	0.09	1.57	(0.81, 3.02)	0.089
Q117.hand	1.4	(0.77, 2.52)	0.135	1.4	(0.77, 2.53)	0.134
Q117.abdomen	1.97	(1.06, 3.66)	0.015*	1.97	(1.07, 3.66)	0.015*
Q117.groin	0.65	(0.19, 2.26)	0.249	0.65	(0.19, 2.25)	0.249
Q117.leg	1.08	(0.66, 1.78)	0.374	1.09	(0.66, 1.78)	0.373
Q117.foot	1.52	(0.91, 2.55)	0.056	1.52	(0.91, 2.55)	0.056
Q117.head	0.92	(0.43, 1.96)	0.413	0.92	(0.43, 1.96)	0.412
Q117.neck	0.86	(0.48, 1.54)	0.304	0.86	(0.48, 1.54)	0.304
Q117.shoulder	1.73	(1.06, 2.83)	0.015*	1.73	(1.06, 2.82)	0.015*

S5 Table (continued)

	Adjustment 1† (Main Analysis)			Adjustment 2‡ (Sensitivity Analysis)		
Variable	OR	1 – α CI**	PV	OR	1 – α CI**	PV
Q117.back	0.97	(0.61, 1.55)	0.446	0.97	(0.61, 1.55)	0.446
Q117.hip	1.36	(0.82, 2.27)	0.117	1.36	(0.82, 2.27)	0.117
Q117.buttocks	0.73	(0.28, 1.88)	0.257	0.73	(0.29, 1.87)	0.257
**EHR Variables**						
Respiratory Condition	2.07	(1.24, 3.48)	0.003*	2.08	(1.24, 3.48)	0.003*
Circulatory Condition	1.45	(0.81, 2.59)	0.107	1.45	(0.81, 2.59)	0.107
Any Cancer	0.88	(0.55, 1.42)	0.301	0.88	(0.55, 1.42)	0.301
Type II Diabetes	1.15	(0.64, 2.04)	0.322	1.15	(0.65, 2.03)	0.322
Kidney Disease	1.39	(0.73, 2.61)	0.157	1.39	(0.74, 2.61)	0.156
Liver Disease	2.57	(1.47, 4.49)	<0.001*	2.57	(1.47, 4.48)	<0.001*
Autoimmune Disease	1.57	(0.96, 2.57)	0.035	1.57	(0.96, 2.57)	0.035
Comorbidity Score	1.26	(1.08, 1.47)	0.002*	1.26	(1.08, 1.47)	0.002*
Smoker – Past	0.72	(0.39, 1.34)	0.151	0.72	(0.39, 1.34)	0.151
Smoker – Current	1.81	(0.98, 3.33)	0.028	1.81	(0.99, 3.34)	0.028
Drinker	1.67	(0.9, 3.09)	0.053	1.67	(0.9, 3.1)	0.052
Neighborhood Education	1.01	(0.8, 1.28)	0.463	1.01	(0.76, 1.36)	0.468
Neighborhood Unemployment	1.13	(0.91, 1.41)	0.137	1.21	(0.92, 1.6)	0.09
Neighborhood Disadvantage	1.01	(0.81, 1.26)	0.479	-	-	-
Population Density	1.22	(1.02, 1.46)	0.016*	1.25	(1.03, 1.52)	0.013*
Neighborhood Poverty	0.92	(0.59, 1.45)	0.513	0.58	(0.19, 1.82)	0.085

All odds ratios are Firth bias-corrected and combined from 30 multiply imputed datasets using Rubin's Rule's. †Adjustment 1: Models adjust for Age, Race/Ethnicity, Sex, BMI, Essential Worker Status, and Education as covariates. ‡Adjustment 2: Models additionally adjust for Neighborhood Disadvantage Index. *p Value statistically significant at 1 – α level. **For covariates, α = 0.05. For other variables, α = 0.05 / k, where k = 184 for Adjustment 1 models and k = 183 for Adjustment 2 models.
