# Supplementary material for: Assessing the added value of linking electronic health records to improve the prediction of self-reported COVID-19 testing and diagnosis
Source: PLoS One. 2022 Jul 25;17(7):e0269017. doi: 10.1371/journal.pone.0269017 (PMC9312965; doi:10.1371/journal.pone.0269017)
Supplement: S5 Table — All odds ratios are Firth bias-corrected and combined from 30 multiply imputed datasets using Rubin’s Rule’s. †Adjustment 1: Models adjust for Age, Race/Ethnicity, Sex, BMI, Essential Worker Status, and Education as covariates. ‡Adjustment 2: Models additionally adjust for Neighborhood Disadvantage Index. *p Value statistically significant at 1 –α level. **For covariates, α = 0.05. For other variables, α = 0.05 / k, where k = 184 for Adjustment 1 models and k = 183 for Adjustment 2 models. (PDF) [file pone.0269017.s005.pdf]

S6 Table. Single-Predictor Model Odds Ratios for COVID-19 Self-Diagnosis

	Adjustment 1† (Main Analysis)			Adjustment 2‡ (Sensitivity Analysis)		
Variable	OR	1 – α CI**	PV	OR	1 – α CI**	PV
**Covariates**						
Age (per 10 years)	0.89	(0.79, 1)	0.028	0.88	(0.78, 1)	0.021*
Race/Ethnicity – NHB	1.39	(0.53, 3.62)	0.249	1.56	(0.6, 4.11)	0.181
Race/Ethnicity – Other	0.8	(0.31, 2.07)	0.326	0.8	(0.31, 2.05)	0.321
Essential Worker	1.37	(0.92, 2.04)	0.061	1.38	(0.93, 2.06)	0.057
Education – Advanced	0.99	(0.65, 1.5)	0.473	0.97	(0.64, 1.48)	0.446
Education – Associate	0.93	(0.55, 1.57)	0.386	0.95	(0.56, 1.61)	0.42
Education – HS or Less	1.03	(0.62, 1.71)	0.451	1.08	(0.65, 1.79)	0.39
Sex	1.28	(0.88, 1.85)	0.098	1.28	(0.89, 1.85)	0.094
BMI	1	(0.97, 1.02)	0.362	1	(0.97, 1.02)	0.413
Neighborhood disadvantage	-	-	-	0.87	(0.71, 1.06)	0.083
**Survey Variables**						
Q130	1.1	(0.77, 1.56)	0.3	1.1	(0.77, 1.55)	0.303
Q38	1.73	(1.09, 2.74)	0.01*	1.69	(1.07, 2.69)	0.013*
Q51	1.74	(0.82, 3.68)	0.075	1.69	(0.8, 3.58)	0.085
Q59	0.85	(0.24, 2.97)	0.397	0.83	(0.24, 2.91)	0.386
Q13	1.47	(1.08, 2)	0.007*	1.47	(1.09, 2)	0.006*
Q46	0.84	(0.54, 1.3)	0.214	0.83	(0.53, 1.28)	0.197
Q16	0.94	(0.79, 1.13)	0.268	0.94	(0.79, 1.13)	0.263
Q17	1.18	(0.66, 2.1)	0.293	1.18	(0.66, 2.11)	0.286
Q18	0.93	(0.88, 0.99)	0.008*	0.93	(0.88, 0.99)	0.008*
Q23.1	0.63	(0.45, 0.89)	0.004*	0.63	(0.45, 0.89)	0.004*
Q23.2	1.4	(0.93, 2.09)	0.051	1.43	(0.95, 2.13)	0.042
Q23.3	1.35	(0.84, 2.18)	0.105	1.37	(0.85, 2.2)	0.099
Q23.4	1.15	(0.82, 1.63)	0.206	1.16	(0.82, 1.63)	0.202
Q23.5	0.98	(0.69, 1.37)	0.443	0.98	(0.69, 1.37)	0.446
Q24 – Agree	0.83	(0.54, 1.29)	0.204	0.84	(0.54, 1.29)	0.212
Q24 – Disagree	0.87	(0.54, 1.41)	0.287	0.87	(0.54, 1.41)	0.288
Q27 – Agree	2.24	(0.67, 7.52)	0.096	2.2	(0.65, 7.37)	0.102
Q27 – Disagree	0.94	(0.43, 2.1)	0.444	0.94	(0.43, 2.09)	0.441
Q45 – Agree	1.3	(0.75, 2.24)	0.172	1.3	(0.76, 2.24)	0.171
Q45 – Disagree	1.12	(0.69, 1.8)	0.322	1.11	(0.69, 1.79)	0.327
Q81	3.53	(2.08, 5.98)	<0.001*	3.48	(2.05, 5.9)	<0.001*
Q85	3.49	(2, 6.11)	<0.001*	3.45	(1.97, 6.03)	<0.001*
Q133	1.89	(0.35, 10.22)	0.229	1.88	(0.35, 10.15)	0.231
Q66 – High	1.05	(0.72, 1.53)	0.392	1.02	(0.7, 1.49)	0.467
Q66 – Low	0.58	(0.31, 1.1)	0.049	0.6	(0.32, 1.15)	0.062
Q150	1.2	(1.03, 1.39)	0.009*	1.18	(1.02, 1.37)	0.015*
Q151	0.88	(0.34, 2.26)	0.398	0.93	(0.36, 2.39)	0.443
Q152 – Family-Owned	0.66	(0.29, 1.49)	0.16	0.68	(0.3, 1.53)	0.174
Q152 – Other	0.69	(0.2, 2.39)	0.278	0.72	(0.21, 2.49)	0.299
Q152 – Rent	0.83	(0.47, 1.48)	0.267	0.9	(0.5, 1.62)	0.366
Q68.1	1.15	(0.78, 1.7)	0.236	1.18	(0.8, 1.74)	0.206
Q68.2	1.59	(1.02, 2.46)	0.02*	1.6	(1.03, 2.49)	0.018*
Q68.3	1.35	(0.93, 1.96)	0.06	1.37	(0.94, 1.99)	0.052
Q70.1	1.77	(1.23, 2.55)	0.001*	1.8	(1.25, 2.6)	0.001*
Q70.2	1.75	(1.23, 2.51)	0.001*	1.79	(1.25, 2.56)	0.001*
Q70.3	2.21	(1.51, 3.22)	<0.001*	2.22	(1.52, 3.25)	<0.001*
Q71.1 – Much	2.03	(1.13, 3.68)	0.009*	2.07	(1.15, 3.75)	0.008*
Q71.1 – Some	1.25	(0.84, 1.85)	0.135	1.29	(0.87, 1.91)	0.106
Q71.2 – Much	1.21	(0.6, 2.44)	0.299	1.24	(0.61, 2.5)	0.277
Q71.2 – Some	1.17	(0.78, 1.75)	0.227	1.2	(0.8, 1.8)	0.188
Q71.3 – Much	1.2	(0.58, 2.46)	0.312	1.24	(0.6, 2.55)	0.278
Q71.3 – Some	1.16	(0.73, 1.82)	0.265	1.19	(0.75, 1.87)	0.23
Q71.4 – Much	0.92	(0.36, 2.37)	0.43	0.94	(0.36, 2.42)	0.446
Q71.4 – Some	1.34	(0.87, 2.07)	0.093	1.38	(0.89, 2.13)	0.074
Q72.1 – Often	1.72	(0.72, 4.11)	0.112	1.77	(0.74, 4.25)	0.099
Q72.1 – Sometimes	1.53	(0.93, 2.53)	0.049	1.57	(0.95, 2.59)	0.04
Q72.2 – Often	1.37	(0.69, 2.7)	0.185	1.41	(0.71, 2.8)	0.16

S6 Table (continued)

Variable	Adjustment 1† (Main Analysis) OR	1 – α CI**	PV	Adjustment 2‡ (Sensitivity Analysis) OR	1 – α CI**	PV
Q72.2 – Sometimes	1.37	(0.87, 2.15)	0.087	1.39	(0.88, 2.18)	0.076
Q72.3 – Often	1.28	(0.69, 2.36)	0.214	1.32	(0.71, 2.43)	0.188
Q72.3 – Sometimes	1.54	(1.04, 2.28)	0.016*	1.56	(1.05, 2.31)	0.014*
Q72.4 – Often	1.16	(0.52, 2.6)	0.361	1.19	(0.53, 2.68)	0.334
Q72.4 – Sometimes	1.34	(0.83, 2.15)	0.114	1.37	(0.85, 2.19)	0.099
Q74.1 – Often	1.52	(0.79, 2.94)	0.105	1.55	(0.8, 2.99)	0.095
Q74.1 – Sometimes	1.31	(0.85, 2.01)	0.108	1.32	(0.86, 2.02)	0.102
Q74.2 – Often	0.97	(0.43, 2.18)	0.469	0.99	(0.44, 2.23)	0.491
Q74.2 – Sometimes	1.32	(0.82, 2.12)	0.126	1.34	(0.84, 2.15)	0.112
Q74.3 – Often	1.28	(0.62, 2.66)	0.252	1.31	(0.63, 2.71)	0.233
Q74.3 – Sometimes	1.08	(0.65, 1.78)	0.383	1.09	(0.66, 1.79)	0.368
Q74.4 – Often	1.26	(0.69, 2.28)	0.227	1.28	(0.7, 2.32)	0.212
Q74.4 – Sometimes	1.37	(0.92, 2.04)	0.061	1.38	(0.93, 2.05)	0.057
Q77	1.63	(1.09, 2.45)	0.009*	1.67	(1.11, 2.51)	0.007*
Q80.1 – Often	1.74	(0.97, 3.1)	0.032	1.69	(0.95, 3.02)	0.038
Q80.1 – Sometimes	2.5	(1.18, 5.33)	0.009*	2.5	(1.18, 5.31)	0.009*
Q80.2 – Often	1.39	(0.72, 2.68)	0.164	1.35	(0.7, 2.61)	0.184
Q80.2 – Sometimes	1.23	(0.47, 3.17)	0.337	1.21	(0.47, 3.13)	0.347
Q80.3 – Often	1.49	(0.83, 2.67)	0.088	1.45	(0.81, 2.6)	0.104
Q80.3 – Sometimes	1.26	(0.56, 2.85)	0.289	1.26	(0.56, 2.84)	0.291
Q80.4 – Often	1.36	(0.7, 2.62)	0.181	1.32	(0.68, 2.55)	0.203
Q80.4 – Sometimes	1.33	(0.57, 3.12)	0.257	1.32	(0.56, 3.09)	0.263
Q141	0.77	(0.32, 1.85)	0.28	0.75	(0.31, 1.8)	0.262
Q145	1.22	(0.79, 1.86)	0.185	1.23	(0.8, 1.88)	0.176
Q146	1.36	(0.95, 1.96)	0.047	1.37	(0.95, 1.97)	0.045
Q147	0.97	(0.54, 1.74)	0.456	1	(0.56, 1.79)	0.497
Q125	0.87	(0.59, 1.28)	0.241	0.87	(0.59, 1.28)	0.244
Q127	0.98	(0.58, 1.65)	0.468	0.99	(0.59, 1.66)	0.479
Q40 – Current User	0.49	(0.19, 1.26)	0.069	0.5	(0.2, 1.3)	0.079
Q40 – Former User	1.02	(0.69, 1.5)	0.46	1.03	(0.7, 1.52)	0.436
Q114.1	1.1	(1.03, 1.17)	0.001*	1.1	(1.04, 1.17)	0.001*
Q114.2	1.11	(1.03, 1.2)	0.002*	1.12	(1.04, 1.2)	0.001*
Q56.1	0.96	(0.91, 1.02)	0.117	0.96	(0.91, 1.02)	0.111
Q56.2	0.99	(0.98, 1.01)	0.168	0.99	(0.98, 1.01)	0.161
Q88 – Monthly	1.01	(0.52, 1.99)	0.485	1.01	(0.52, 1.99)	0.484
Alcohol – Weekly or More	0.8	(0.45, 1.45)	0.233	0.81	(0.45, 1.45)	0.235
Q38.1	1.3	(0.44, 3.83)	0.316	1.26	(0.43, 3.7)	0.339
Q38.2	2.18	(1.03, 4.62)	0.021*	2.14	(1.01, 4.53)	0.024*
Q59.1	3.27	(0.59, 18.02)	0.087	3.33	(0.6, 18.34)	0.083
Q59.2	0.85	(0.05, 14.78)	0.457	0.84	(0.05, 14.51)	0.452
Q59.3	2.42	(0.45, 12.91)	0.15	2.3	(0.43, 12.31)	0.165
Q59.4	1.47	(0.08, 26.43)	0.397	1.38	(0.08, 24.86)	0.414
Q145.1	1.26	(0.07, 21.88)	0.437	1.42	(0.08, 24.34)	0.404
Q145.2	1.14	(0.57, 2.28)	0.358	1.15	(0.57, 2.29)	0.35
Q145.3	0.61	(0.12, 3.03)	0.274	0.62	(0.13, 3.08)	0.28
Q145.4	2.01	(0.11, 36.19)	0.319	2.02	(0.11, 36.3)	0.317
Q145.5	1.51	(0.94, 2.41)	0.043	1.52	(0.95, 2.42)	0.041
Q145.6	1.48	(0.86, 2.55)	0.079	1.5	(0.87, 2.58)	0.073
Q146.1	1.42	(0.92, 2.18)	0.057	1.43	(0.93, 2.19)	0.053
Q146.2	0.87	(0.3, 2.54)	0.401	0.9	(0.31, 2.62)	0.425
Q146.3	4.6	(0.19, 111.27)	0.174	4.51	(0.19, 108.13)	0.176
Q146.4	0.45	(0.03, 7.23)	0.286	0.46	(0.03, 7.44)	0.294
Q146.5	1.32	(0.85, 2.03)	0.107	1.33	(0.86, 2.05)	0.101
Q146.6	1.18	(0.73, 1.91)	0.244	1.19	(0.74, 1.91)	0.242
Q147.1	1.11	(0.55, 2.24)	0.384	1.16	(0.57, 2.33)	0.341
Q147.2	0.48	(0.1, 2.36)	0.181	0.49	(0.1, 2.42)	0.19
Q147.3	1.69	(0.58, 4.96)	0.169	1.69	(0.58, 4.96)	0.168
Q147.4	1.29	(0.25, 6.53)	0.381	1.3	(0.26, 6.6)	0.375
Q125.1	0.88	(0.25, 3.11)	0.421	0.89	(0.25, 3.14)	0.427

S6 Table (continued)

Variable	Adjustment 1† (Main Analysis)			Adjustment 2‡ (Sensitivity Analysis)		
	OR	1 – α CI**	PV	OR	1 – α CI**	PV
Q125.2	0.86	(0.17, 4.37)	0.427	0.88	(0.17, 4.48)	0.44
Q125.3	0.45	(0.09, 2.24)	0.166	0.45	(0.09, 2.25)	0.167
Q125.4	0.83	(0.54, 1.27)	0.193	0.83	(0.55, 1.27)	0.195
Q125.5	1.17	(0.33, 4.12)	0.406	1.17	(0.33, 4.13)	0.402
Q125.6	0.88	(0.17, 4.41)	0.437	0.88	(0.18, 4.42)	0.439
Q125.7	0.95	(0.37, 2.42)	0.455	0.96	(0.38, 2.46)	0.469
Q125.8	0.16	(0.01, 2.51)	0.096	0.16	(0.01, 2.54)	0.098
Q125.9	1.51	(0.84, 2.73)	0.084	1.51	(0.84, 2.73)	0.083
Q127.1	3.43	(0.94, 12.54)	0.031	3.39	(0.93, 12.36)	0.032
Q127.2	1.73	(0.49, 6.15)	0.198	1.75	(0.49, 6.21)	0.193
Q127.3	0.76	(0.3, 1.94)	0.283	0.76	(0.3, 1.94)	0.284
Q127.4	0.91	(0.44, 1.9)	0.402	0.93	(0.44, 1.94)	0.419
Q127.5	0.92	(0.47, 1.78)	0.399	0.92	(0.48, 1.79)	0.408
Q36.live.alone	0.93	(0.54, 1.61)	0.403	0.96	(0.56, 1.65)	0.44
Q36.house.diagnose	10.23	(5.99, 17.47)	<0.001*	10.3	(6.03, 17.6)	<0.001*
Q18.G – Detractor	1.15	(0.77, 1.71)	0.248	1.15	(0.77, 1.71)	0.251
Q18.G – Promoter	1	(0.56, 1.76)	0.495	1	(0.57, 1.77)	0.499
Q126.1	1.6	(0.09, 27.83)	0.373	1.66	(0.1, 28.86)	0.363
Q126.2	1.32	(0.56, 3.11)	0.263	1.34	(0.57, 3.16)	0.25
Q118.1	0.75	(0.48, 1.17)	0.102	0.74	(0.47, 1.15)	0.089
Q118.2	1.12	(0.72, 1.74)	0.315	1.11	(0.72, 1.73)	0.316
Q118.3	0.42	(0.08, 2.08)	0.144	0.43	(0.09, 2.12)	0.149
Q118.4	0.41	(0.08, 2.04)	0.139	0.43	(0.09, 2.1)	0.147
Q118.5	0.55	(0.31, 0.99)	0.023*	0.55	(0.31, 0.98)	0.022*
Q118.6	0.97	(0.65, 1.43)	0.431	0.96	(0.65, 1.42)	0.418
Q118.7	1.26	(0.88, 1.81)	0.104	1.26	(0.88, 1.81)	0.103
Q133.1	0.41	(0.03, 6.49)	0.262	0.43	(0.03, 6.89)	0.276
Q133.2	1.14	(0.39, 3.31)	0.408	1.16	(0.4, 3.38)	0.39
Q133.3	0.64	(0.22, 1.86)	0.206	0.66	(0.23, 1.9)	0.219
Q28.1	1.13	(0.58, 2.2)	0.364	1.13	(0.58, 2.21)	0.356
Q28.2	1.08	(0.74, 1.57)	0.344	1.08	(0.74, 1.57)	0.35
Q28.3	0.78	(0.44, 1.4)	0.203	0.79	(0.44, 1.4)	0.208
Q28.4	0.91	(0.59, 1.39)	0.329	0.92	(0.6, 1.41)	0.345
Q28.5	1.39	(0.97, 1.97)	0.035	1.39	(0.97, 1.97)	0.035
Q28.6	1.48	(0.71, 3.11)	0.149	1.48	(0.71, 3.1)	0.15
Q28.7	1.39	(0.97, 1.97)	0.034	1.4	(0.98, 1.99)	0.032
Q28.8	1.11	(0.79, 1.56)	0.277	1.11	(0.79, 1.56)	0.275
Q28.9	1.24	(0.88, 1.76)	0.106	1.24	(0.88, 1.75)	0.109
Q28.10	1.21	(0.84, 1.74)	0.149	1.22	(0.85, 1.74)	0.144
Q28.11	0.96	(0.48, 1.92)	0.451	0.96	(0.48, 1.93)	0.458
Q28.12	1.09	(0.73, 1.61)	0.338	1.1	(0.74, 1.62)	0.325
Q28.13	1.22	(0.86, 1.72)	0.135	1.21	(0.86, 1.72)	0.137
Q28.14	1.03	(0.73, 1.45)	0.439	1.04	(0.73, 1.46)	0.419
Q28.15	0.98	(0.69, 1.38)	0.446	0.98	(0.69, 1.38)	0.446
Q28.16	1.67	(1.15, 2.44)	0.004*	1.67	(1.15, 2.44)	0.004*
Q28.17	1.46	(0.91, 2.36)	0.059	1.47	(0.91, 2.36)	0.057
Q28.18	1.15	(0.4, 3.33)	0.398	1.17	(0.4, 3.37)	0.387
Q117.face	0.41	(0.08, 2.02)	0.136	0.42	(0.09, 2.07)	0.143
Q117.jaw	1.42	(0.73, 2.77)	0.15	1.48	(0.76, 2.87)	0.126
Q117.breast	1.14	(0.49, 2.68)	0.382	1.17	(0.5, 2.74)	0.361
Q117.arm	1.75	(1.11, 2.78)	0.008*	1.8	(1.14, 2.85)	0.006*
Q117.hand	1.36	(0.88, 2.09)	0.081	1.39	(0.9, 2.14)	0.068
Q117.abdomen	1.6	(0.96, 2.66)	0.035	1.64	(0.98, 2.72)	0.029
Q117.groin	1.39	(0.69, 2.79)	0.175	1.41	(0.7, 2.82)	0.167
Q117.leg	1.31	(0.91, 1.89)	0.071	1.33	(0.92, 1.92)	0.062
Q117.foot	1.11	(0.72, 1.69)	0.321	1.12	(0.73, 1.71)	0.301
Q117.head	1.01	(0.57, 1.79)	0.485	1.04	(0.59, 1.84)	0.451
Q117.neck	1.8	(1.23, 2.61)	0.001*	1.83	(1.26, 2.66)	0.001*
Q117.shoulder	1.52	(1.04, 2.22)	0.016*	1.54	(1.05, 2.25)	0.013*

S6 Table (continued)

	Adjustment 1† (Main Analysis)			Adjustment 2‡ (Sensitivity Analysis)		
Variable	OR	1 – α CI**	PV	OR	1 – α CI**	PV
Q117.back	1.6	(1.14, 2.26)	0.004*	1.62	(1.15, 2.29)	0.003*
Q117.hip	1.3	(0.88, 1.91)	0.092	1.32	(0.9, 1.94)	0.08
Q117.buttocks	0.86	(0.45, 1.67)	0.331	0.87	(0.45, 1.69)	0.345
**EHR Variables**						
Respiratory Condition	1.03	(0.72, 1.48)	0.426	1.02	(0.72, 1.46)	0.452
Circulatory Condition	0.98	(0.63, 1.53)	0.461	0.97	(0.62, 1.51)	0.449
Any Cancer	0.77	(0.54, 1.11)	0.083	0.78	(0.54, 1.12)	0.088
Type II Diabetes	1.28	(0.84, 1.96)	0.127	1.29	(0.84, 1.96)	0.123
Kidney Disease	1.16	(0.71, 1.89)	0.277	1.17	(0.72, 1.92)	0.26
Liver Disease	0.79	(0.42, 1.49)	0.232	0.79	(0.42, 1.49)	0.234
Autoimmune Disease	1.36	(0.92, 2)	0.06	1.34	(0.91, 1.97)	0.068
Comorbidity Score	1.02	(0.9, 1.16)	0.382	1.02	(0.9, 1.16)	0.391
Smoker – Past	1.1	(0.74, 1.62)	0.318	1.11	(0.75, 1.64)	0.303
Smoker – Current	0.51	(0.23, 1.15)	0.052	0.53	(0.24, 1.18)	0.06
Drinker	1	(0.66, 1.54)	0.492	0.99	(0.64, 1.51)	0.472
Neighborhood Education	0.92	(0.75, 1.12)	0.207	1.01	(0.78, 1.3)	0.476
Neighborhood Unemployment	0.91	(0.75, 1.11)	0.174	0.98	(0.78, 1.24)	0.432
Neighborhood Disadvantage	0.87	(0.71, 1.06)	0.083	-	-	-
Population Density	0.97	(0.81, 1.17)	0.391	1.02	(0.83, 1.24)	0.436
Neighborhood Poverty	0.82	(0.65, 1.02)	0.039	0.74	(0.46, 1.21)	0.115

All odds ratios are Firth bias-corrected and combined from 30 multiply imputed datasets using Rubin's Rule's. †Adjustment 1: Models adjust for Age, Race/Ethnicity, Sex, BMI, Essential Worker Status, and Education as covariates. ‡Adjustment 2: Models additionally adjust for Neighborhood Disadvantage Index. *p Value statistically significant at 1 – α level. **For covariates, α = 0.05. For other variables, α = 0.05 / k, where k = 184 for Adjustment 1 models and k = 183 for Adjustment 2 models.
