# Supplementary material for: Assessing the added value of linking electronic health records to improve the prediction of self-reported COVID-19 testing and diagnosis
Source: PLoS One. 2022 Jul 25;17(7):e0269017. doi: 10.1371/journal.pone.0269017 (PMC9312965; doi:10.1371/journal.pone.0269017)
Supplement: S6 Table — †Adjustment 1: Models adjust for Age, Race/Ethnicity, Sex, BMI, Essential Worker Status, and Education as covariates. ‡Adjustment 2: Models additionally adjust for Neighborhood Disadvantage Index. *Detailed descriptions of each outcome are available in the methods sections as well as the supplement (S1 Table). (PDF) [file pone.0269017.s006.pdf]

S1 Table. Complete Variable Descriptions

Name	Content*	Details
**Outcome Variables**		
Tested	Was the respondent/patient tested for COVID-19?	Binary. For survey-based analysis, derived from survey Question 2. For Michigan Medicine case-control analysis, cases include anyone who was tested for COVID-19 at Michigan Medicine from March 10th to June 30th, 2020.
Diagnosed	Was the respondent/patient diagnosed with COVID-19?	Binary. For survey-based analysis, derived directly from survey Question 1. Includes those diagnosed by a physician or by a test. Those who tested negative or self-diagnosed due to symptoms are excluded from controls. For Michigan Medicine case-control analysis, cases include anyone who was diagnosed with COVID-19 at Michigan Medicine by a physician or test from March 10th to June 30th, 2020.
Self-Diagnosed	Did the respondent diagnosis *themselves* with COVID-19 due to symptoms?	Binary. 1 if reported to have "self-diagnosed due to symptoms" and 0 otherwise. For survey-based analysis, derived directly from survey Question 1. Self-diagnosis data is not available in Michigan Medicine EHR, so this outcome was excluded from the EHR-based case control analysis.
**Covariates**		
Age	Age of the respondent/patient.	Numeric. Known from EHR. In modeling, used per ten years.
Sex (Q25)	Sex of the respondent/patient.	Binary. 1 if female, 0 if male. Derived from survey responses.
BMI	Body Mass Index of the respondent/patient.	Numeric. Computed from survey-reported height and weight as kg/m^2 for recency purposes. Where missing, obtained from EHR.
Race/ Ethnicity	Race/Ethnicity of the respondent/patient	Nominal. Three levels: Non-Hispanic White, Non-Hispanic African American, Other. Derived from EHR, which had less missingness. Where still missing, obtained from survey.
Essential Worker (Q94)	Is the respondent currently working as an essential worker?	Binary. Derived from survey responses.
Education (Q67)	Highest level of school the respondent has completed.	Nominal. Four Levels: High School or Less, Associate Degree, Bachelor's Degree (reference), Advanced Degree
**Survey Variables**		
Q130	Is someone in the respondent's household high risk, other than the respondent?	Binary. 1 if yes, 0 if no.
Q38	Is a medical professional living in the respondent's household, other than the respondent?	Binary. 1 if yes, 0 if no.
Q59	Is a first responder living in the respondent's household, other than the respondent?	Binary. 1 if yes, 0 if no.
Q13	How many times has the respondent gotten the flu in the past year?	Numeric. Integer 0-3. ("3 or more" treated as 3)

S1 Table (continued)

Name	Content*	Details
Q46	Did the respondent get a flu shot in the past year?	Binary. 1 if yes, 0 if no.
Q16	How many times has the respondent had the common cold in the past year?	Numeric. Values 0, 1.5 (representing “1-2” response), 3.5 (“3-4”), and 5.5 (“5+”)
Q17	Has the respondent ever been hospitalized for a viral infection, excluding COVID-19?	Binary. 1 if yes, 0 if no.
Q18	How concerned has the respondent been about COVD-19 in the past 7 days?	Numeric. Integer values 0-10.
Q23.1	How concerned is the respondent about: Someone close to them contracting COVID-19?	Binary. (Not or Slightly Concerned treated as 0, Very or Extremely Concerned as 1)
Q23.2	How concerned is the respondent about: Getting into serious financial trouble due to COVID-19?	Binary. (Not or Slightly Concerned treated as 0, Very or Extremely Concerned as 1)
Q23.3	How concerned is the respondent about: Losing their job due to COVID-19?	Binary. (Not or Slightly Concerned treated as 0, Very or Extremely Concerned as 1)
Q23.4	How concerned is the respondent about: How long it will be before life returns to normal?	Binary. (Not or Slightly Concerned treated as 0, Very or Extremely Concerned as 1)
Q23.5	How concerned is the respondent about: Not seeing friends and family?	Binary. (Not or Slightly Concerned treated as 0, Very or Extremely Concerned as 1)
Q24	Respondent’s level of agreement: “Since the outbreak, I see others in my community as a threat”	Nominal. Three levels: Disagree (totally or slightly), Neutral, Agree (totally or slightly). Neutral used as reference.
Q27	Respondent’s level of agreement: “I have had more negative thoughts toward Asian Americans”	Nominal. Three levels: Disagree (totally or slightly), Neutral, Agree (totally or slightly) Neutral used as reference.
Q45	Respondent’s level of agreement: “I have had more negative thoughts toward Asian Americans”	Nominal. Three levels: Disagree (totally or slightly), Neutral, Agree (totally or slightly) Neutral used as reference.
Q81	Have any of the respondent’s blood relatives been diagnosed with COVID-19?	Binary. 1 if yes, 0 if no.
Q85	Have any of the respondent’s blood relatives died of COVID-19?	Binary. 1 if yes, 0 if no.
Q133	Is the respondent pregnant?	Binary. 1 if yes, 0 if no.
Q66	What is the respondent’s average annual household income?	Nominal. Three levels: Low (≤40,000), Medium, High (> 100,000). Medium used as reference.
Q150	How many automobiles does the respondent have?	Numeric. Integer 0-10. (10+ response option treated as 10)
Q151	What is the respondent’s primary mode of transportation?	Nominal. Two levels: Car, and Other
Q152	What is the respondent’s current living situation?	Nominal. Four levels: Own a house, rent an apartment, live in family-owned property, and other. Own a house used as reference.

S1 Table (continued)

Name	Content*	Details
Q68.1	The respondent's fatigue over the past week.	Binary. 1 if Moderate or Severe, 0 if Mild or None.
Q68.2	The respondent's trouble thinking or remembering in the past week.	Binary. 1 if Moderate or Severe, 0 if Mild or None.
Q68.3	The respondent's waking up tired or unrefreshed in the past week.	Binary. 1 if Moderate or Severe, 0 if Mild or None.
Q70.1	During the past six months, has the respondent had pain or cramps in lower abdomen?	Binary. 1 if yes, 0 if no.
Q70.2	During the past six months, has the respondent had depression?	Binary. 1 if yes, 0 if no.
Q70.3	During the past six months, has the respondent had headaches?	Binary. 1 if yes, 0 if no.
Q71.1	How easily is the respondent able to do chores such as vacuuming or yardwork?	Nominal. Three levels: No difficulty, Some Difficulty, Much difficulty or Unable. No difficulty used as reference.
Q71.2	How easily is the respondent able to go own stairs at a normal pace?	Nominal. Three levels: No difficulty, Some Difficulty, Much difficulty or Unable. No difficulty used as reference.
Q71.3	How easily is the respondent able to go for a walk of at least 15 minutes?	Nominal. Three levels: No difficulty, Some Difficulty, Much difficulty or Unable. No difficulty used as reference.
Q71.4	How easily is the respondent able to run errands and shop?	Nominal. Three levels: No difficulty, Some Difficulty, Much difficulty or Unable. No difficulty used as reference.
Q72.1	In the past seven days, how often has the respondent felt worthless?	Nominal. Three levels: Rarely or Never, Sometimes, Often or Always. Rarely or Never as reference.
Q72.2	In the past seven days, how often has the respondent felt helpless?	Nominal. Three levels: Rarely or Never, Sometimes, Often or Always. Rarely or Never as reference.
Q72.3	In the past seven days, how often has the respondent felt depressed?	Nominal. Three levels: Rarely or Never, Sometimes, Often or Always. Rarely or Never as reference.
Q72.4	In the past seven days, how often has the respondent felt depressed?	Nominal. Three levels: Rarely or Never, Sometimes, Often or Always. Rarely or Never as reference.
Q74.1	In the past seven days, how often has the respondent felt fearful?	Nominal. Three levels: Rarely or Never, Sometimes, Often or Always. Rarely or Never as reference.
Q74.2	In the past seven days, how often has the respondent struggled to focus on anything but anxiety?	Nominal. Three levels: Rarely or Never, Sometimes, Often or Always. Rarely or Never as reference.
Q74.3	In the past seven days, how often did the respondent's worries overwhelm them?	Nominal. Three levels: Rarely or Never, Sometimes, Often or Always. Rarely or Never as reference.
Q74.4	In the past seven days, how often has the respondent felt uneasy?	Nominal. Three levels: Rarely or Never, Sometimes, Often or Always. Rarely or Never as reference.
Q77	In the past seven days, how was the respondent's sleep quality?	Binary. 1 if poor or very poor , 0 if fair or better.
Q80.1	How often does the respondent have someone to help them if they are confined in bed?	Nominal. Three levels: Rarely or Never, Sometimes, Usually or Always. Rarely or Never as reference.

S1 Table (continued)

Name	Content*	Details
Q80.2	How often does the respondent have someone to take them to the doctor if necessary?	Nominal. Three levels: Rarely or Never, Sometimes, Usually or Always. Rarely or Never as reference.
Q80.3	How often does the respondent have someone to help them with their daily chores if they are sick?	Nominal. Three levels: Rarely or Never, Sometimes, Usually or Always. Rarely or Never as reference.
Q80.4	How often does the respondent have someone to run errands for them?	Nominal. Three levels: Rarely or Never, Sometimes, Usually or Always Rarely or Never as reference.
Q141	Does the respondent have a primary care physician?	Binary. 1 if yes, 0 if no.
Q145	Does the respondent have an immune system condition?	Binary. 1 if yes, 0 if no.
Q146	Does the respondent have a respiratory condition?	Binary. 1 if yes, 0 if no.
Q147	Does the respondent have a genitourinary/metabolic condition?	Binary. 1 if yes, 0 if no.
Q125	Does the respondent have a cardiovascular condition?	Binary. 1 if yes, 0 if no.
Q127	Do any of these conditions/treatments apply to the respondent: Leukemia, Lymphoma, Malignant solid tumor, Chemotherapy, Radiation therapy	Binary. 1 if yes, 0 if no.
Q40	The respondent's tobacco/nicotine use status	Nominal. Four Levels: Current User, Never Used (reference), Former user
Q114.1	The respondent's overall body pain, at worst, in the past week.	Numeric. 0 – 10
Q114.2	The respondent's overall body pain, on average, in the past week.	Numeric. 0 – 10
Q56.1	Respondent's height.	Numeric. In inches.
Q56.2	Respondent's weight.	Numeric. In pounds.
Q88	In the past 12 months, how often has the respondent had 5 or more drinks if male or 4 or more drinks if female, in a single day?	Nominal. Three levels: Weekly or more, Monthly, Less than Monthly (reference)
Q38.1	Is there a physician currently living in the respondent's household?	Binary. 1 if yes, 0 if no.
Q38.2	Is there a nurse currently living in the respondent's household?	Binary. 1 if yes, 0 if no.
Q59.1	Is there a police officer currently living in the respondent's home?	Binary. 1 if yes, 0 if no.
Q59.2	Is there a firefighter currently living in the respondent's home?	Binary. 1 if yes, 0 if no.
Q59.3	Is there an EMT currently living in the respondent's home?	Binary. 1 if yes, 0 if no.
Q59.4	Is there military personnel currently living in the respondent's home?	Binary. 1 if yes, 0 if no.
Q145.1	Does the respondent have HIV?	Binary. 1 if yes, 0 if no.

S1 Table (continued)

Name	Content*	Details
Q145.2	Is the respondent immunocompromised?	Binary. 1 if yes, 0 if no.
Q145.3	Has the respondent had an organ transplant?	Binary. 1 if yes, 0 if no.
Q145.4	Has the respondent had a bone marrow transplant?	Binary. 1 if yes, 0 if no.
Q145.5	Does the respondent have Type I Diabetes?	Binary. 1 if yes, 0 if no.
Q145.6	Does the respondent have Type II Diabetes?	Binary. 1 if yes, 0 if no.
Q146.1	Does the respondent have asthma?	Binary. 1 if yes, 0 if no.
Q146.2	Does the respondent have COPD?	Binary. 1 if yes, 0 if no.
Q146.3	Does the respondent have Cystic Fibrosis?	Binary. 1 if yes, 0 if no.
Q146.4	Does the respondent have Emphysema?	Binary. 1 if yes, 0 if no.
Q146.5	Does the respondent have sleep apnea?	Binary. 1 if yes, 0 if no.
Q146.6	Does the respondent use a home CPAP?	Binary. 1 if yes, 0 if no.
Q147.1	Does the respondent have chronic kidney disease?	Binary. 1 if yes, 0 if no.
Q147.2	Does the respondent have liver disease?	Binary. 1 if yes, 0 if no.
Q147.3	Does the respondent have gallbladder disease?	Binary. 1 if yes, 0 if no.
Q147.4	Does the respondent have pancreas disease?	Binary. 1 if yes, 0 if no.
Q125.1	Has the respondent had a balloon angioplasty or percutaneous coronary intervention?	Binary. 1 if yes, 0 if no.
Q125.2	Has the respondent had a coronary artery bypass?	Binary. 1 if yes, 0 if no.
Q125.3	Has the respondent had congestive heart failure?	Binary. 1 if yes, 0 if no.
Q125.4	Does the respondent have hypertension?	Binary. 1 if yes, 0 if no.
Q125.5	Has the respondent had a myocardial infarction?	Binary. 1 if yes, 0 if no.
Q125.6	Does the respondent have peripheral vascular disease?	Binary. 1 if yes, 0 if no.
Q125.7	Has the respondent had a blood clot or blood clotting disorder?	Binary. 1 if yes, 0 if no.
Q125.8	Has the respondent had a stroke?	Binary. 1 if yes, 0 if no.
Q125.9	Does the respondent have arrythmia?	Binary. 1 if yes, 0 if no.
Q127.1	Does the respondent have leukemia?	Binary. 1 if yes, 0 if no.
Q127.2	Does the respondent have lymphoma?	Binary. 1 if yes, 0 if no.
Q127.3	Does the respondent have a malignant solid tumor?	Binary. 1 if yes, 0 if no.

S1 Table (continued)

Name	Content*	Details
Q127.4	Has the respondent had chemotherapy?	Binary. 1 if yes, 0 if no.
Q127.5	Has the respondent had radiation therapy?	Binary. 1 if yes, 0 if no.
Q36.live.alone	Does anyone else live with the respondent?	Binary. 1 if yes, 0 if no.
Q36.house.diagnose	Has anyone in the respondent's household been diagnosed with COVID-19	Binary. 1 if yes, 0 if no.
Q18.G	How concerned has the respondent been about COVD-19 in the past 7 days?	Binary. 1 if yes, 0 if no.
Q126.1	Does the respondent have dementia?	Binary. 1 if yes, 0 if no.
Q126.2	Does the respondent have a neurological disease?	Binary. 1 if yes, 0 if no.
Q118.1	During lockdown, has the respondent's moderate to strenuous exercise increased?	Binary. 1 if yes, 0 if no.
Q118.2	During lockdown, has the respondent's alcohol consumption increased?	Binary. 1 if yes, 0 if no.
Q118.3	During lockdown, has the respondent's drug us increased?	Binary. 1 if yes, 0 if no.
Q118.4	During lockdown, has the respondent's tobacco use increased?	Binary. 1 if yes, 0 if no.
Q118.5	During lockdown, has the respondent improved the respondent's sleep habits?	Binary. 1 if yes, 0 if no.
Q118.6	During lockdown, has the respondent improved their nutrition?	Binary. 1 if yes, 0 if no.
Q118.7	During lockdown, has the respondent gained weight?	Binary. 1 if yes, 0 if no.
Q133.1	During lockdown, has the respondent's opioid pain medication use increased?	Binary. 1 if yes, 0 if no.
Q133.2	During lockdown, has the respondent's benzodiazepine use increased?	Binary. 1 if yes, 0 if no.
Q133.3	During lockdown, has the respondent's marijuana / cannabis us increased?	Binary. 1 if yes, 0 if no.
Q28.1	Is anyone in the respondent's immediate family deceased?	Binary. 1 if yes, 0 if no.
Q28.2	Does anyone in the respondent's immediate family have cancer?	Binary. 1 if yes, 0 if no.
Q28.3	Does anyone in the respondent's immediate family have chronic kidney disease?	Binary. 1 if yes, 0 if no.
Q28.4	Does anyone in the respondent's immediate family have COPD?	Binary. 1 if yes, 0 if no.
Q28.5	Does anyone in the respondent's immediate family have coronary artery disease?	Binary. 1 if yes, 0 if no.
Q28.6	Does anyone in the respondent's immediate family have Crohn's Disease?	Binary. 1 if yes, 0 if no.
Q28.7	Does anyone in the respondent's immediate family have depression or anxiety?	Binary. 1 if yes, 0 if no.

S1 Table (continued)

Name	Content*	Details
Q28.8	Has anyone in the respondent’s immediate family ever had a heart attack?	Binary. 1 if yes, 0 if no.
Q28.9	Does anyone in the respondent’s family have high cholesterol?	Binary. 1 if yes, 0 if no.
Q28.10	Does anyone in the respondent’s immediate family have hypertension?	Binary. 1 if yes, 0 if no.
Q28.11	Does anyone in the respondent’s immediate family have liver disease?	Binary. 1 if yes, 0 if no.
Q28.12	Does anyone in the respondent’s immediate family have asthma?	Binary. 1 if yes, 0 if no.
Q28.13	Has anyone in the respondent’s immediate family had a stroke?	Binary. 1 if yes, 0 if no.
Q28.14	Does anyone in the respondent’s immediate family have diabetes?	Binary. 1 if yes, 0 if no.
Q28.15	Does anyone in the respondent’s immediate family have heart disease?	Binary. 1 if yes, 0 if no.
Q28.16	Does anyone in the respondent’s immediate family have an irregular heart rhythm?	Binary. 1 if yes, 0 if no.
Q28.17	Does anyone in the respondent’s immediate family have an autoimmune disease?	Binary. 1 if yes, 0 if no.
Q28.18	Has anyone in the respondent’s immediate family had an organ transplant?	Binary. 1 if yes, 0 if no.
Q117.face	Has the respondent felt persistent or recurrent pain? – Face	Binary. 1 if yes, 0 if no.
Q117.jaw	Has the respondent felt persistent or recurrent pain? – Right or Left Jaw	Binary. 1 if yes, 0 if no.
Q117.breast	Has the respondent felt persistent or recurrent pain? – Right or Left Breast	Binary. 1 if yes, 0 if no.
Q117.arm	Has the respondent felt persistent or recurrent pain? – Right or Left Arm or Elbow	Binary. 1 if yes, 0 if no.
Q117.hand	Has the respondent felt persistent or recurrent pain? – Right or Left Hand or Wrist	Binary. 1 if yes, 0 if no.
Q117.abdomen	Has the respondent felt persistent or recurrent pain? – Abdomen or Pelvis	Binary. 1 if yes, 0 if no.
Q117.groin	Has the respondent felt persistent or recurrent pain? – Right or Left Groin	Binary. 1 if yes, 0 if no.
Q117.leg	Has the respondent felt persistent or recurrent pain? – Right or Left Leg or Knee	Binary. 1 if yes, 0 if no.
Q117.foot	Has the respondent felt persistent or recurrent pain? – Right or Left Ankle or Foot	Binary. 1 if yes, 0 if no.
Q117.head	Has the respondent felt persistent or recurrent pain? – Head	Binary. 1 if yes, 0 if no.
Q117.neck	Has the respondent felt persistent or recurrent pain? – Neck	Binary. 1 if yes, 0 if no.

S1 Table (continued)

Name	Content*	Details
Q117.shoulder	Has the respondent felt persistent or recurrent pain? – Right or Left Shoulder	Binary. 1 if yes, 0 if no.
Q117.back	Has the respondent felt persistent or recurrent pain? – Back	Binary. 1 if yes, 0 if no.
Q117.hip	Has the respondent felt persistent or recurrent pain? – Right or Left Hip	Binary. 1 if yes, 0 if no.
Q117.buttocks	Has the respondent felt persistent or recurrent pain? – Right or Left Buttocks	Binary. 1 if yes, 0 if no.
EHR Variables -		
Any Cancer	At least one of the following phecodes observed: 145, 145.2, 145.3, 145.4, 149, 149.1, 149.2, 149.3, 149.4, 149.5, 149.9, 150, 151, 153, 153.2, 153.3, 155, 155.1, 157, 158, 159, 159.2, 159.3, 159.4, 164, 165, 165.1, 170, 170.1, 170.2, 172, 172.1, 172.11, 172.2, 172.21, 172.22, 172.3, 174, 174.1, 174.11, 175, 180, 180.1, 180.3, 182, 184, 184.1, 184.11, 184.2, 185, 187, 187.1, 187.2, 189, 189.1, 189.11, 189.12, 189.2, 189.21, 189.4, 190, 191, 191.1, 191.11, 193, 194, 195, 195.1, 195.3, 196, 197, 198, 198.1, 198.2, 198.3, 198.4, 198.5, 198.6, 198.7, 199.4, 200, 200.1, 201, 202, 202.2, 202.21, 202.22, 202.23, 202.24, 204, 204.1, 204.11, 204.12, 204.2, 204.21, 204.22, 204.3, 204.4, 209	Binary. Phenomes are truncated differently depending on analysis.<br><br>Survey-based analysis: Codes updated up to March 23rd, 2020, prior to the survey's start. Derived from Michigan Genomics Initiative biorepository, the subset of Michigan Medicine on which the survey data were collected.<br><br>Michigan Medicine EHR Analysis (restricted): Codes updated up to June 22nd, 2020. For the Tested and Diagnosed cohorts, codes truncated to at least 14 days prior to the time of the first test or diagnosis.<br><br>Michigan Medicine EHR Analysis (unrestricted): Codes updated up to June 22nd, 2020. For the Tested and Diagnosed cohorts, codes truncated to at least 0 days prior to the time of the first test or diagnosis.
Respiratory conditions	At least one of the following phecodes observed: 470, 471, 472, 473.1, 474.2, 475, 475.9, 495, 495.1, 495.11, 495.2, 496, 496.1, 496.2, 496.21, 496.3, 499, 500, 500.1, 500.2, 502, 503, 504, 504.1, 505, 506, 507, 508, 509, 509.1, 509.2, 509.3, 509.5, 509.8, 510.2, 513.3, 513.31, 513.32, 513.4, 513.8, 514.1, 514.2, 516, 516.1, 519.1, 519.2, 473.3, 473.4, 478, 512, 513, 514, 519.8, 519.9	
Circulatory conditions	At least one of the following phecodes observed: 94, 394.1, 394.2, 394.3, 394.4, 394.7, 395, 395.1, 395.2, 395.3, 395.4, 395.6, 401, 401.1, 401.2, 401.21, 401.22, 401.3, 411, 411.1, 411.2, 411.3, 411.4, 411.41, 411.8, 411.9, 414, 414.2, 415.2, 415.21, 416, 420.1, 420.22, 420.3, 425, 425.1, 425.11, 425.12, 425.2, 425.8, 426, 426.2, 426.21, 426.22, 426.23, 426.24, 426.25, 426.3, 426.31, 426.32, 426.4, 426.7, 426.8, 426.9, 426.91, 426.92, 427, 427.1, 427.11, 427.12, 427.2, 427.21, 427.22, 427.3, 427.4, 427.41, 427.42, 427.5, 427.6, 427.61, 427.7, 427.8, 427.9, 428, 428.1, 428.2, 428.3, 428.4, 429.1, 430, 433.1, 433.11, 433.12, 433.2, 433.21, 433.3, 433.31, 433.32, 433.5, 433.8, 440, 440.1, 440.2, 440.21, 440.22, 440.9, 441.2, 442, 442.1, 442.11, 442.2, 442.3, 442.4, 442.8, 443, 443.1, 443.7, 443.8, 443.9, 444, 444.1, 444.2, 444.5, 446.1, 446.3, 446.4, 446.5, 446.6, 446.7, 446.8, 446.9, 447, 447.1, 447.7, 448, 450, 451, 451.2, 452, 452.1, 452.2, 452.8, 453, 454, 454.1,454.11, 455, 456, 457, 457.2, 457.3, 458, 458.1, 458.2, 458.9, 459, 459.1, 459.7, 459.9, 396, 418, 429, 430	
Type II Diabetes	At least one of the following phecodes observed: 250.2	
Kidney diseases	At least one of the following phecodes observed: 585	
Liver diseases	At least one of the following phecodes observed: 571	

S1 Table (continued)

Name	Content*	Details
Autoimmune diseases	At least one of the following phecodes observed: 242.1, 250.1, 335, 557.1, 694.1, 695.4, 696.4, 697, 704.1, 714.1, 717	
Comorbidity Score	-	Numeric. Sum of the previous 7 ICD-code derived variables.
Neighborhood Education	Proportion of adults with less than high school diploma, 2010	Numeric. Defined by US census tract based on EHR. Standardized for analysis.
Neighborhood Unemployment	Proportion of 16+ civilian labor force unemployed, 2010	Numeric. Defined by US census tract based on EHR. Standardized for analysis.
Neighborhood Poverty	Proportion of people with income past 12 months below poverty level, 2010	Numeric. Defined by US census tract based on EHR. Standardized for analysis.
Population Density	Persons per square mile, 2010	Numeric. Defined by US census tract based on EHR. Standardized for analysis.
Neighborhood Disadvantage Index	Mean of proportion of Population in Poverty; Unemployed; with Public Assistance Income; and Female-Headed Families with children, 2010	Numeric. Defined by US census tract based on EHR. Standardized for analysis.
Smoking Status	Respondent’s smoking status	Categorical with 3 levels: Never Smoked (reference), Former Smoker, and Current Smoker.
Alcohol	Respondent’s alcohol status.	Binary. 1 if drinker, 0 if non-drinker.

*Each variable is named after the survey question it is derived from, but the variable does not always represent the syntax or nature of that question exactly. Certain survey questions were redefined for simplicity or interpretation.

S3 Table. Description of Michigan Medicine EHR COVID-19 Cohorts

Variables	Controls*<br>(n = 30,000)	Tested for SARS-CoV-2**<br>(n = 15,929)	Diagnosed with COVID-19**<br>(n = 1,483)
**Numeric, Mean (SD)**			
Age	43.22 (24.36)<br>*n = 29,989*	47.02 (22.84)<br>*n = 15.929*	52.24 (18.69)<br>*n = 1,483*
Body Mass Index	28.48 (7.31)<br>*n = 16,607*	29.66 (7.69)<br>*n = 13,297*	31.86 (9.06)<br>*n = 1,303*
**Categoric, No. (%)**			
Sex			
Male	13,840 (46.13%)	6,769 (42.09%)	678 (45.72%)
Female	16,141 (53.84%)	9,160 (57.51%)	805 (54.28%)
Missing	19 (0.06%)	0 (0.00%)	0 (0.00%)
Race / Ethnicity			
Non-Hispanic Black	2,215 (7.38%)	2,140 (13.43%)	500 (33.72%)
Non-Hispanic White	18,992 (63.31%)	11,404 (71.59%)	692 (46.66%)
Other	2,899 (9.66%)	1,439 (9.03%)	150 (10.11%)
Missing	13,840 (46.13%)	6,769 (42.09%)	678 (45.72%)

*Controls consisted of randomly selected patients who were alive at the time of the extraction, who had an encounter with Michigan Medicine between April 23, 2012 and June 21, 2020, and who were not part of the other cohorts.

**Tested cohort includes all patients who were tested for SARS-CoV-2 between March 10th and June 30th of 2020. Diagnosed cohort includes those who tested positive as well as those who were diagnosed by a physician during that span.

S4 Table. Single-Predictor Model Odds Ratios for COVID-19 Testing

	Adjustment 1† (Main Analysis)			Adjustment 2‡ (Sensitivity Analysis)		
Variable	OR	1 – α CI**	PV	OR	1 – α CI**	PV
**Covariates**						
Age (per 10 years)	0.99	(0.93, 1.04)	0.291	0.99	(0.94, 1.04)	0.323
Race/Ethnicity – NHB	2.12	(1.45, 3.1)	<0.001*	2.04	(1.38, 3)	<0.001*
Race/Ethnicity – Other	1.4	(0.99, 1.98)	0.029	1.4	(0.99, 1.98)	0.028
Essential Worker	1.65	(1.39, 1.96)	<0.001*	1.64	(1.39, 1.95)	<0.001*
Education – Advanced	1.06	(0.88, 1.27)	0.277	1.06	(0.88, 1.28)	0.259
Education – Associate	1.22	(0.98, 1.52)	0.036	1.21	(0.97, 1.51)	0.042
Education – HS or Less	1.37	(1.11, 1.7)	0.002*	1.35	(1.1, 1.68)	0.003*
Sex	1.2	(1.03, 1.4)	0.011*	1.2	(1.03, 1.4)	0.011*
BMI	1.01	(1, 1.02)	0.094	1.01	(1, 1.02)	0.112
Neighborhood disadvantage	-	-	-	1.04	(0.97, 1.13)	0.131
**Survey Variables**						
Q130	0.88	(0.76, 1.02)	0.049	0.88	(0.76, 1.02)	0.05
Q38	1.04	(0.82, 1.31)	0.379	1.05	(0.83, 1.32)	0.355
Q51	1.17	(0.79, 1.74)	0.222	1.18	(0.79, 1.75)	0.208
Q59	1.4	(0.89, 2.2)	0.072	1.41	(0.9, 2.21)	0.069
Q13	1.38	(1.2, 1.59)	<0.001*	1.38	(1.2, 1.59)	<0.001*
Q46	1.36	(1.09, 1.68)	0.003*	1.36	(1.1, 1.69)	0.002*
Q16	1.08	(1, 1.16)	0.028	1.08	(1, 1.16)	0.028
Q17	1.99	(1.6, 2.48)	<0.001*	1.99	(1.6, 2.47)	<0.001*
Q18	1.02	(1, 1.05)	0.044	1.02	(1, 1.05)	0.046
Q23.1	1.06	(0.91, 1.23)	0.242	1.06	(0.91, 1.23)	0.243
Q23.2	1.27	(1.06, 1.52)	0.004*	1.27	(1.06, 1.51)	0.005*
Q23.3	1.48	(1.2, 1.82)	<0.001*	1.48	(1.2, 1.82)	<0.001*
Q23.4	1.17	(1.01, 1.35)	0.019*	1.17	(1.01, 1.35)	0.019*
Q23.5	1.14	(0.99, 1.33)	0.036	1.14	(0.99, 1.33)	0.036
Q24 – Agree	0.99	(0.82, 1.2)	0.456	0.99	(0.82, 1.19)	0.449
Q24 – Disagree	0.93	(0.75, 1.15)	0.243	0.93	(0.75, 1.15)	0.243
Q27 – Agree	1.21	(0.62, 2.36)	0.29	1.22	(0.62, 2.38)	0.281
Q27 – Disagree	1.25	(0.88, 1.77)	0.11	1.25	(0.88, 1.77)	0.108
Q45 – Agree	1.05	(0.83, 1.31)	0.348	1.05	(0.83, 1.31)	0.351
Q45 – Disagree	0.89	(0.73, 1.07)	0.107	0.89	(0.73, 1.07)	0.108
Q81	1.62	(1.19, 2.21)	0.001*	1.63	(1.2, 2.22)	0.001*
Q85	1.57	(1.13, 2.19)	0.004*	1.58	(1.13, 2.2)	0.003*
Q133	0.88	(0.28, 2.77)	0.414	0.88	(0.28, 2.77)	0.411
Q66 – High	0.94	(0.79, 1.11)	0.233	0.95	(0.8, 1.13)	0.273
Q66 – Low	1.15	(0.92, 1.43)	0.108	1.14	(0.91, 1.41)	0.129
Q150	0.99	(0.93, 1.07)	0.423	1	(0.93, 1.07)	0.49
Q151	0.94	(0.63, 1.4)	0.383	0.92	(0.62, 1.37)	0.347
Q152 – Family-Owned	1.38	(1.03, 1.84)	0.016*	1.37	(1.02, 1.83)	0.017*
Q152 – Other	1.52	(1.03, 2.26)	0.018*	1.5	(1.01, 2.23)	0.022*
Q152 – Rent	1.15	(0.91, 1.46)	0.126	1.13	(0.88, 1.44)	0.168
Q68.1	1.84	(1.57, 2.15)	<0.001*	1.83	(1.56, 2.14)	<0.001*
Q68.2	1.7	(1.4, 2.06)	<0.001*	1.69	(1.39, 2.06)	<0.001*
Q68.3	1.68	(1.43, 1.98)	<0.001*	1.68	(1.43, 1.97)	<0.001*
Q70.1	1.67	(1.42, 1.97)	<0.001*	1.67	(1.42, 1.96)	<0.001*
Q70.2	1.33	(1.14, 1.56)	<0.001*	1.32	(1.13, 1.55)	<0.001*
Q70.3	1.56	(1.34, 1.82)	<0.001*	1.56	(1.33, 1.82)	<0.001*
Q71.1 – Much	2.62	(2.03, 3.38)	<0.001*	2.61	(2.02, 3.37)	<0.001*
Q71.1 – Some	1.7	(1.44, 2)	<0.001*	1.69	(1.43, 2)	<0.001*
Q71.2 – Much	2.43	(1.87, 3.15)	<0.001*	2.42	(1.86, 3.14)	<0.001*
Q71.2 – Some	1.62	(1.37, 1.92)	<0.001*	1.61	(1.36, 1.91)	<0.001*
Q71.3 – Much	2.09	(1.6, 2.72)	<0.001*	2.07	(1.59, 2.71)	<0.001*
Q71.3 – Some	1.65	(1.37, 1.98)	<0.001*	1.64	(1.36, 1.97)	<0.001*
Q71.4 – Much	2.33	(1.72, 3.16)	<0.001*	2.32	(1.71, 3.15)	<0.001*
Q71.4 – Some	1.72	(1.43, 2.06)	<0.001*	1.71	(1.42, 2.05)	<0.001*
Q72.1 – Often	1.2	(0.77, 1.88)	0.208	1.19	(0.76, 1.86)	0.219
Q72.1 – Sometimes	1.36	(1.08, 1.71)	0.004*	1.35	(1.07, 1.7)	0.005*
Q72.2 – Often	1.2	(0.88, 1.65)	0.128	1.19	(0.87, 1.64)	0.14

S4 Table (continued)

Variable	Adjustment 1† (Main Analysis) OR	1 – α CI**	PV	Adjustment 2‡ (Sensitivity Analysis) OR	1 – α CI**	PV
Q72.2 – Sometimes	1.3	(1.06, 1.59)	0.005*	1.29	(1.06, 1.58)	0.006*
Q72.3 – Often	1.48	(1.14, 1.91)	0.001*	1.47	(1.14, 1.89)	0.002*
Q72.3 – Sometimes	1.28	(1.07, 1.53)	0.003*	1.28	(1.07, 1.53)	0.003*
Q72.4 – Often	1.08	(0.76, 1.56)	0.33	1.08	(0.75, 1.54)	0.347
Q72.4 – Sometimes	1.22	(0.98, 1.52)	0.036	1.21	(0.98, 1.51)	0.041
Q74.1 – Often	1.47	(1.09, 1.99)	0.006*	1.46	(1.08, 1.98)	0.007*
Q74.1 – Sometimes	1.16	(0.95, 1.4)	0.068	1.16	(0.95, 1.4)	0.071
Q74.2 – Often	1.31	(0.94, 1.83)	0.054	1.3	(0.94, 1.82)	0.059
Q74.2 – Sometimes	1.37	(1.11, 1.69)	0.002*	1.36	(1.1, 1.68)	0.002*
Q74.3 – Often	1.52	(1.09, 2.1)	0.006*	1.51	(1.09, 2.09)	0.007*
Q74.3 – Sometimes	1.44	(1.17, 1.78)	<0.001*	1.44	(1.17, 1.77)	<0.001*
Q74.4 – Often	1.26	(0.97, 1.64)	0.043	1.25	(0.96, 1.63)	0.047
Q74.4 – Sometimes	1.18	(0.98, 1.41)	0.038	1.17	(0.98, 1.4)	0.039
Q77	1.6	(1.34, 1.92)	<0.001*	1.59	(1.33, 1.91)	<0.001*
Q80.1 – Often	0.77	(0.64, 0.93)	0.003*	0.77	(0.64, 0.94)	0.004*
Q80.1 – Sometimes	0.99	(0.73, 1.34)	0.473	0.99	(0.73, 1.34)	0.475
Q80.2 – Often	0.9	(0.71, 1.14)	0.193	0.91	(0.71, 1.15)	0.212
Q80.2 – Sometimes	1.15	(0.81, 1.64)	0.221	1.15	(0.81, 1.64)	0.213
Q80.3 – Often	0.89	(0.72, 1.1)	0.139	0.9	(0.72, 1.11)	0.155
Q80.3 – Sometimes	1.29	(0.96, 1.73)	0.045	1.29	(0.96, 1.73)	0.044
Q80.4 – Often	0.95	(0.74, 1.21)	0.331	0.95	(0.75, 1.22)	0.357
Q80.4 – Sometimes	1.28	(0.93, 1.78)	0.067	1.29	(0.93, 1.78)	0.064
Q141	1.31	(0.8, 2.15)	0.142	1.32	(0.8, 2.17)	0.136
Q145	1.28	(1.07, 1.53)	0.004*	1.27	(1.07, 1.53)	0.004*
Q146	1.42	(1.22, 1.66)	<0.001*	1.42	(1.22, 1.66)	<0.001*
Q147	1.96	(1.6, 2.41)	<0.001*	1.95	(1.59, 2.4)	<0.001*
Q125	1.51	(1.29, 1.77)	<0.001*	1.51	(1.29, 1.77)	<0.001*
Q127	1.17	(0.95, 1.44)	0.07	1.17	(0.95, 1.44)	0.072
Q40 – Current User	1.34	(1.01, 1.78)	0.021*	1.33	(1, 1.77)	0.025
Q40 – Former User	1.1	(0.94, 1.3)	0.118	1.1	(0.93, 1.3)	0.126
Q114.1	1.09	(1.06, 1.12)	<0.001*	1.09	(1.06, 1.12)	<0.001*
Q114.2	1.11	(1.08, 1.15)	<0.001*	1.11	(1.07, 1.15)	<0.001*
Q56.1	0.99	(0.97, 1.02)	0.297	0.99	(0.97, 1.02)	0.303
Q56.2	1	(0.99, 1)	0.346	1	(0.99, 1)	0.352
Q88 – Monthly	1.05	(0.78, 1.42)	0.38	1.05	(0.78, 1.42)	0.38
Alcohol – Weekly or More	0.88	(0.69, 1.12)	0.15	0.88	(0.69, 1.12)	0.149
Q38.1	1.17	(0.7, 1.95)	0.28	1.18	(0.7, 1.98)	0.265
Q38.2	1.04	(0.68, 1.59)	0.43	1.05	(0.68, 1.6)	0.416
Q59.1	4.02	(1.67, 9.68)	0.001*	4.02	(1.67, 9.67)	0.001*
Q59.2	1.78	(0.67, 4.68)	0.123	1.78	(0.67, 4.69)	0.123
Q59.3	1.71	(0.7, 4.18)	0.118	1.74	(0.71, 4.24)	0.112
Q59.4	1.3	(0.32, 5.3)	0.356	1.33	(0.33, 5.4)	0.345
Q145.1	1.81	(0.63, 5.25)	0.136	1.75	(0.6, 5.07)	0.151
Q145.2	1.49	(1.13, 1.96)	0.003*	1.48	(1.12, 1.96)	0.003*
Q145.3	1.9	(1.23, 2.95)	0.002*	1.9	(1.22, 2.94)	0.002*
Q145.4	0.89	(0.16, 5.09)	0.447	0.89	(0.15, 5.07)	0.446
Q145.5	1.15	(0.93, 1.42)	0.095	1.15	(0.93, 1.42)	0.098
Q145.6	1.2	(0.95, 1.52)	0.063	1.2	(0.95, 1.52)	0.066
Q146.1	1.25	(1.03, 1.51)	0.013*	1.24	(1.02, 1.51)	0.014*
Q146.2	2.14	(1.56, 2.96)	<0.001*	2.13	(1.54, 2.93)	<0.001*
Q146.3	3.22	(0.42, 24.53)	0.129	3.26	(0.43, 24.85)	0.127
Q146.4	2.3	(1.29, 4.11)	0.002*	2.28	(1.28, 4.07)	0.003*
Q146.5	1.34	(1.12, 1.61)	0.001*	1.34	(1.12, 1.61)	0.001*
Q146.6	1.35	(1.11, 1.64)	0.001*	1.35	(1.11, 1.63)	0.001*
Q147.1	2.24	(1.76, 2.86)	<0.001*	2.22	(1.74, 2.84)	<0.001*
Q147.2	1.97	(1.35, 2.87)	<0.001*	1.95	(1.34, 2.84)	<0.001*
Q147.3	0.97	(0.54, 1.74)	0.463	0.97	(0.54, 1.74)	0.464
Q147.4	0.72	(0.3, 1.73)	0.229	0.71	(0.29, 1.72)	0.226
Q125.1	1.53	(1.01, 2.33)	0.022*	1.53	(1.01, 2.32)	0.023*

S4 Table (continued)

	Adjustment 1† (Main Analysis)			Adjustment 2‡ (Sensitivity Analysis)		
Variable	OR	1 – α CI**	PV	OR	1 – α CI**	PV
Q125.2	1.68	(1.02, 2.78)	0.021*	1.67	(1.01, 2.75)	0.023*
Q125.3	1.61	(1.09, 2.36)	0.008*	1.61	(1.09, 2.36)	0.008*
Q125.4	1.13	(0.96, 1.34)	0.074	1.13	(0.96, 1.34)	0.075
Q125.5	1.27	(0.77, 2.1)	0.176	1.27	(0.77, 2.09)	0.178
Q125.6	1.16	(0.65, 2.07)	0.311	1.16	(0.65, 2.07)	0.312
Q125.7	2.07	(1.52, 2.82)	<0.001*	2.06	(1.51, 2.81)	<0.001*
Q125.8	1.28	(0.83, 1.97)	0.132	1.27	(0.83, 1.96)	0.137
Q125.9	1.36	(1.04, 1.76)	0.011*	1.36	(1.04, 1.76)	0.011*
Q127.1	0.88	(0.33, 2.35)	0.396	0.88	(0.33, 2.36)	0.399
Q127.2	1.06	(0.55, 2.04)	0.43	1.06	(0.55, 2.03)	0.435
Q127.3	1.1	(0.79, 1.54)	0.282	1.1	(0.79, 1.54)	0.283
Q127.4	1.06	(0.79, 1.41)	0.353	1.05	(0.79, 1.41)	0.366
Q127.5	1	(0.76, 1.3)	0.495	1	(0.76, 1.3)	0.489
Q36.live.alone	1.33	(1.08, 1.64)	0.004*	1.32	(1.07, 1.63)	0.005*
Q36.house.diagnose	3.38	(2.36, 4.85)	<0.001*	3.38	(2.35, 4.84)	<0.001*
Q18.G – Detractor	0.92	(0.78, 1.09)	0.176	0.92	(0.78, 1.09)	0.177
Q18.G – Promoter	1.11	(0.89, 1.39)	0.176	1.11	(0.89, 1.39)	0.18
Q126.1	2.17	(0.75, 6.28)	0.077	2.14	(0.74, 6.19)	0.081
Q126.2	1.63	(1.14, 2.32)	0.004*	1.62	(1.13, 2.3)	0.004*
Q118.1	0.82	(0.68, 0.99)	0.018*	0.82	(0.68, 0.99)	0.02*
Q118.2	0.92	(0.75, 1.13)	0.215	0.92	(0.75, 1.13)	0.216
Q118.3	0.9	(0.55, 1.48)	0.341	0.89	(0.54, 1.47)	0.33
Q118.4	1.5	(0.99, 2.25)	0.026	1.48	(0.98, 2.23)	0.03
Q118.5	0.96	(0.78, 1.18)	0.341	0.96	(0.78, 1.18)	0.345
Q118.6	1.08	(0.91, 1.27)	0.184	1.08	(0.92, 1.28)	0.177
Q118.7	1.08	(0.92, 1.26)	0.181	1.08	(0.92, 1.26)	0.181
Q133.1	3	(1.72, 5.24)	<0.001*	2.96	(1.7, 5.17)	<0.001*
Q133.2	2.05	(1.37, 3.08)	<0.001*	2.04	(1.36, 3.06)	<0.001*
Q133.3	1.2	(0.83, 1.74)	0.163	1.19	(0.83, 1.72)	0.174
Q28.1	0.93	(0.7, 1.23)	0.301	0.93	(0.7, 1.22)	0.294
Q28.2	1.05	(0.89, 1.23)	0.284	1.05	(0.89, 1.23)	0.282
Q28.3	1.08	(0.86, 1.34)	0.26	1.07	(0.86, 1.34)	0.267
Q28.4	1.19	(1.01, 1.42)	0.021*	1.19	(1, 1.41)	0.023*
Q28.5	1.07	(0.92, 1.25)	0.189	1.07	(0.92, 1.25)	0.19
Q28.6	1.4	(1, 1.97)	0.025	1.4	(1, 1.97)	0.025
Q28.7	1.19	(1.02, 1.38)	0.012*	1.19	(1.02, 1.38)	0.012*
Q28.8	1.14	(0.99, 1.32)	0.039	1.14	(0.99, 1.32)	0.039
Q28.9	1.09	(0.94, 1.26)	0.137	1.09	(0.94, 1.26)	0.135
Q28.10	1.08	(0.93, 1.26)	0.148	1.08	(0.93, 1.26)	0.152
Q28.11	1.19	(0.9, 1.56)	0.108	1.19	(0.9, 1.56)	0.112
Q28.12	1.09	(0.92, 1.3)	0.156	1.09	(0.92, 1.29)	0.164
Q28.13	1.07	(0.92, 1.25)	0.183	1.07	(0.92, 1.25)	0.18
Q28.14	1.02	(0.88, 1.18)	0.417	1.01	(0.87, 1.17)	0.432
Q28.15	1.24	(1.07, 1.43)	0.002*	1.24	(1.07, 1.43)	0.002*
Q28.16	1.2	(1.01, 1.43)	0.019*	1.2	(1.01, 1.43)	0.019*
Q28.17	1	(0.79, 1.26)	0.487	0.99	(0.79, 1.26)	0.483
Q28.18	0.76	(0.45, 1.29)	0.156	0.76	(0.44, 1.29)	0.152
Q117.face	1.34	(0.88, 2.05)	0.087	1.33	(0.87, 2.03)	0.093
Q117.jaw	1.59	(1.19, 2.13)	0.001*	1.57	(1.17, 2.11)	0.001*
Q117.breast	1.62	(1.16, 2.25)	0.002*	1.6	(1.15, 2.23)	0.003*
Q117.arm	1.27	(1.02, 1.58)	0.016*	1.26	(1.01, 1.57)	0.02*
Q117.hand	1.2	(0.99, 1.45)	0.03	1.19	(0.99, 1.44)	0.034
Q117.abdomen	1.74	(1.39, 2.18)	<0.001*	1.73	(1.39, 2.17)	<0.001*
Q117.groin	0.95	(0.68, 1.34)	0.393	0.95	(0.67, 1.34)	0.385
Q117.leg	1.22	(1.04, 1.43)	0.006*	1.22	(1.04, 1.42)	0.007*
Q117.foot	1.23	(1.03, 1.47)	0.01*	1.23	(1.03, 1.47)	0.011*
Q117.head	1.43	(1.13, 1.8)	0.001*	1.42	(1.13, 1.79)	0.002*
Q117.neck	1.26	(1.05, 1.5)	0.006*	1.25	(1.05, 1.49)	0.007*
Q117.shoulder	1.22	(1.03, 1.44)	0.012*	1.21	(1.02, 1.44)	0.014*

S4 Table (continued)

	Adjustment 1† (Main Analysis)			Adjustment 2‡ (Sensitivity Analysis)		
Variable	OR	1 – α CI**	PV	OR	1 – α CI**	PV
Q117.back	1.19	(1.02, 1.38)	0.012*	1.19	(1.02, 1.38)	0.013*
Q117.hip	1.15	(0.97, 1.36)	0.056	1.14	(0.96, 1.35)	0.061
Q117.buttocks	1.12	(0.87, 1.44)	0.196	1.11	(0.86, 1.43)	0.206
**EHR Variables**						
Respiratory Condition	1.33	(1.14, 1.56)	<0.001*	1.34	(1.14, 1.57)	<0.001*
Circulatory Condition	1.26	(1.03, 1.56)	0.013*	1.27	(1.03, 1.56)	0.013*
Any Cancer	0.95	(0.81, 1.1)	0.236	0.94	(0.81, 1.1)	0.23
Type II Diabetes	1.21	(1.01, 1.45)	0.02*	1.21	(1.01, 1.45)	0.021*
Kidney Disease	1.54	(1.27, 1.86)	<0.001*	1.53	(1.26, 1.86)	<0.001*
Liver Disease	1.43	(1.15, 1.77)	0.001*	1.42	(1.15, 1.77)	0.001*
Autoimmune Disease	1.22	(1.03, 1.45)	0.011*	1.23	(1.03, 1.46)	0.009*
Comorbidity Score	1.14	(1.08, 1.2)	<0.001*	1.14	(1.08, 1.2)	<0.001*
Smoker – Past	1.2	(1.02, 1.42)	0.015*	1.2	(1.02, 1.42)	0.016*
Smoker – Current	1.29	(1.01, 1.66)	0.022*	1.28	(1, 1.65)	0.026
Drinker	0.95	(0.8, 1.14)	0.29	0.96	(0.8, 1.14)	0.315
Neighborhood Education	1.04	(0.96, 1.13)	0.174	1.02	(0.92, 1.13)	0.381
Neighborhood Unemployment	1.05	(0.97, 1.13)	0.104	1.04	(0.94, 1.14)	0.231
Neighborhood Disadvantage	1.04	(0.97, 1.13)	0.131	-	-	-
Population Density	1.05	(0.98, 1.14)	0.089	1.04	(0.96, 1.13)	0.146
Neighborhood Poverty	1.01	(0.94, 1.09)	0.388	0.9	(0.77, 1.05)	0.095

All odds ratios are Firth bias-corrected and combined from 30 multiply imputed datasets using Rubin's Rule's. Complete variable descriptions are available in the supplement (S1 Table).

†Adjustment 1: Models adjust for Age, Race/Ethnicity, Sex, BMI, Essential Worker Status, and Education as covariates.

‡Adjustment 2: Models additionally adjust for Neighborhood Disadvantage Index.

*p Value statistically significant at 1 – α level.

**For covariates, α = 0.05. For other variables, α = 0.05 / k, where k = 184 for Adjustment 1 models and k = 183 for Adjustment 2 models

S5 Table. Single-Predictor Model Odds Ratios for COVID-19 Diagnosis

	Adjustment 1† (Main Analysis)			Adjustment 2‡ (Sensitivity Analysis)		
Variable	OR	1 – α CI**	PV	OR	1 – α CI**	PV
**Covariates**						
Age (per 10 years)	0.75	(0.64, 0.87)	<0.001*	0.75	(0.64, 0.87)	<0.001*
Race/Ethnicity – NHB	3.27	(1.4, 7.62)	0.003*	3.27	(1.38, 7.76)	0.004*
Race/Ethnicity – Other	1.87	(0.82, 4.26)	0.068	1.87	(0.82, 4.25)	0.068
Essential Worker	2.7	(1.7, 4.29)	<0.001*	2.7	(1.7, 4.28)	<0.001*
Education – Advanced	1.19	(0.67, 2.11)	0.273	1.19	(0.67, 2.1)	0.275
Education – Associate	1.25	(0.64, 2.42)	0.257	1.24	(0.64, 2.41)	0.259
Education – HS or Less	1.43	(0.75, 2.7)	0.139	1.42	(0.75, 2.7)	0.141
Sex	0.97	(0.61, 1.55)	0.45	0.97	(0.61, 1.55)	0.451
BMI	1.01	(0.98, 1.04)	0.32	1.01	(0.98, 1.04)	0.322
Neighborhood disadvantage	-	-	-	1.01	(0.81, 1.26)	0.479
**Survey Variables**						
Q130	0.66	(0.42, 1.05)	0.041	0.66	(0.42, 1.05)	0.041
Q38	1.33	(0.72, 2.44)	0.179	1.33	(0.72, 2.44)	0.179
Q51	1.78	(0.74, 4.31)	0.101	1.78	(0.74, 4.31)	0.1
Q59	2.83	(1.16, 6.93)	0.011*	2.83	(1.16, 6.92)	0.011*
Q13	1.16	(0.75, 1.79)	0.253	1.16	(0.75, 1.79)	0.252
Q46	1	(0.57, 1.75)	0.497	1	(0.57, 1.74)	0.498
Q16	0.91	(0.72, 1.15)	0.223	0.91	(0.72, 1.15)	0.223
Q17	2.18	(1.16, 4.09)	0.008*	2.18	(1.16, 4.08)	0.008*
Q18	1.03	(0.96, 1.12)	0.205	1.03	(0.96, 1.12)	0.205
Q23.1	0.85	(0.54, 1.33)	0.24	0.85	(0.55, 1.33)	0.239
Q23.2	1.23	(0.74, 2.04)	0.211	1.23	(0.74, 2.04)	0.21
Q23.3	1.5	(0.86, 2.61)	0.078	1.5	(0.86, 2.6)	0.077
Q23.4	1.72	(1.08, 2.72)	0.011*	1.72	(1.08, 2.72)	0.011*
Q23.5	1.22	(0.78, 1.9)	0.192	1.22	(0.78, 1.89)	0.192
Q24 – Agree	0.71	(0.41, 1.21)	0.105	0.71	(0.41, 1.21)	0.104
Q24 – Disagree	0.73	(0.4, 1.31)	0.144	0.73	(0.4, 1.31)	0.143
Q27 – Agree	5.02	(0.99, 25.58)	0.026	5.02	(0.99, 25.54)	0.026
Q27 – Disagree	1.47	(0.42, 5.17)	0.274	1.47	(0.42, 5.16)	0.273
Q45 – Agree	0.84	(0.43, 1.62)	0.298	0.84	(0.43, 1.61)	0.296
Q45 – Disagree	0.79	(0.45, 1.38)	0.202	0.79	(0.45, 1.38)	0.201
Q81	8.7	(5.07, 14.93)	<0.001*	8.68	(5.06, 14.9)	<0.001*
Q85	9.38	(5.39, 16.35)	<0.001*	9.37	(5.38, 16.3)	<0.001*
Q133	0.87	(0.05, 15.18)	0.463	0.88	(0.05, 15.01)	0.464
Q66 – High	1.56	(0.95, 2.55)	0.039	1.59	(0.97, 2.61)	0.034
Q66 – Low	0.66	(0.3, 1.48)	0.157	0.65	(0.29, 1.45)	0.146
Q150	1.08	(0.89, 1.32)	0.215	1.08	(0.89, 1.32)	0.212
Q151	2.03	(0.88, 4.65)	0.047	2.04	(0.89, 4.69)	0.047
Q152 – Family-Owned	1.49	(0.71, 3.14)	0.145	1.49	(0.71, 3.13)	0.146
Q152 – Other	3.62	(1.56, 8.4)	0.001*	3.61	(1.55, 8.37)	0.001*
Q152 – Rent	0.64	(0.3, 1.38)	0.129	0.64	(0.3, 1.38)	0.126
Q68.1	1.33	(0.82, 2.18)	0.125	1.34	(0.82, 2.18)	0.124
Q68.2	1.71	(0.99, 2.97)	0.028	1.71	(0.99, 2.97)	0.028
Q68.3	1.42	(0.88, 2.28)	0.076	1.42	(0.88, 2.28)	0.075
Q70.1	2.69	(1.71, 4.23)	<0.001*	2.7	(1.72, 4.24)	<0.001*
Q70.2	1.02	(0.63, 1.65)	0.463	1.02	(0.63, 1.65)	0.463
Q70.3	2.45	(1.48, 4.06)	<0.001*	2.45	(1.49, 4.05)	<0.001*
Q71.1 – Much	3.65	(1.67, 7.99)	0.001*	3.66	(1.67, 8)	0.001*
Q71.1 – Some	1.99	(1.21, 3.25)	0.003*	2	(1.22, 3.27)	0.003*
Q71.2 – Much	3.52	(1.53, 8.08)	0.002*	3.52	(1.54, 8.08)	0.001*
Q71.2 – Some	2.29	(1.39, 3.77)	0.001*	2.3	(1.39, 3.79)	0.001*
Q71.3 – Much	4.31	(2.05, 9.07)	<0.001*	4.35	(2.06, 9.16)	<0.001*
Q71.3 – Some	2.47	(1.44, 4.24)	<0.001*	2.48	(1.45, 4.26)	<0.001*
Q71.4 – Much	2.52	(0.94, 6.73)	0.033	2.52	(0.95, 6.73)	0.032
Q71.4 – Some	2.05	(1.19, 3.51)	0.005*	2.06	(1.2, 3.52)	0.004*
Q72.1 – Often	1.11	(0.31, 3.98)	0.439	1.11	(0.31, 3.97)	0.438
Q72.1 – Sometimes	1.89	(1.05, 3.4)	0.017*	1.89	(1.05, 3.4)	0.017*
Q72.2 – Often	1.11	(0.46, 2.71)	0.408	1.11	(0.46, 2.71)	0.407

S5 Table (continued)

	Adjustment 1† (Main Analysis)			Adjustment 2‡ (Sensitivity Analysis)		
Variable	OR	1 – α CI**	PV	OR	1 – α CI**	PV
Q72.2 – Sometimes	1.46	(0.83, 2.58)	0.096	1.46	(0.83, 2.58)	0.096
Q72.3 – Often	1.15	(0.55, 2.43)	0.356	1.15	(0.55, 2.42)	0.356
Q72.3 – Sometimes	1.03	(0.59, 1.78)	0.462	1.03	(0.59, 1.77)	0.462
Q72.4 – Often	0.76	(0.26, 2.25)	0.311	0.76	(0.26, 2.24)	0.31
Q72.4 – Sometimes	0.85	(0.43, 1.7)	0.326	0.85	(0.43, 1.7)	0.324
Q74.1 – Often	0.91	(0.35, 2.35)	0.422	0.91	(0.35, 2.35)	0.422
Q74.1 – Sometimes	0.92	(0.51, 1.67)	0.394	0.92	(0.51, 1.67)	0.393
Q74.2 – Often	1.03	(0.42, 2.58)	0.471	1.03	(0.42, 2.57)	0.471
Q74.2 – Sometimes	1.23	(0.67, 2.26)	0.25	1.23	(0.67, 2.26)	0.25
Q74.3 – Often	1.42	(0.6, 3.37)	0.213	1.42	(0.6, 3.36)	0.214
Q74.3 – Sometimes	1.85	(1.07, 3.22)	0.014*	1.85	(1.07, 3.22)	0.014*
Q74.4 – Often	0.94	(0.41, 2.18)	0.443	0.94	(0.41, 2.18)	0.443
Q74.4 – Sometimes	1.71	(1.05, 2.79)	0.016*	1.71	(1.05, 2.79)	0.016*
Q77	1.28	(0.74, 2.21)	0.187	1.28	(0.74, 2.21)	0.186
Q80.1 – Often	1.01	(0.55, 1.85)	0.484	1.01	(0.55, 1.85)	0.483
Q80.1 – Sometimes	0.78	(0.27, 2.26)	0.324	0.78	(0.27, 2.26)	0.323
Q80.2 – Often	0.7	(0.37, 1.32)	0.136	0.7	(0.37, 1.32)	0.136
Q80.2 – Sometimes	0.37	(0.1, 1.42)	0.073	0.37	(0.1, 1.41)	0.073
Q80.3 – Often	0.84	(0.46, 1.55)	0.292	0.84	(0.46, 1.55)	0.292
Q80.3 – Sometimes	0.7	(0.26, 1.88)	0.237	0.69	(0.26, 1.87)	0.235
Q80.4 – Often	0.88	(0.44, 1.74)	0.353	0.88	(0.44, 1.74)	0.353
Q80.4 – Sometimes	0.56	(0.18, 1.7)	0.152	0.56	(0.18, 1.69)	0.152
Q141	1.03	(0.34, 3.08)	0.479	1.03	(0.35, 3.07)	0.479
Q145	0.8	(0.43, 1.5)	0.247	0.8	(0.43, 1.5)	0.247
Q146	1.79	(1.13, 2.84)	0.007*	1.79	(1.13, 2.84)	0.007*
Q147	1.72	(0.89, 3.33)	0.053	1.72	(0.89, 3.33)	0.052
Q125	1.02	(0.61, 1.7)	0.468	1.02	(0.61, 1.7)	0.468
Q127	0.77	(0.34, 1.71)	0.259	0.77	(0.35, 1.71)	0.258
Q40 – Current User	0.72	(0.27, 1.9)	0.255	0.72	(0.27, 1.9)	0.254
Q40 – Former User	1.06	(0.63, 1.77)	0.417	1.06	(0.63, 1.77)	0.417
Q114.1	1.02	(0.94, 1.1)	0.338	1.02	(0.94, 1.1)	0.338
Q114.2	1.04	(0.94, 1.14)	0.244	1.04	(0.94, 1.14)	0.243
Q56.1	1.08	(1, 1.16)	0.026	1.08	(1, 1.16)	0.026
Q56.2	1.01	(1, 1.03)	0.015*	1.01	(1, 1.03)	0.014*
Q88 – Monthly	0.68	(0.26, 1.76)	0.213	0.68	(0.26, 1.76)	0.212
Alcohol – Weekly or More	0.89	(0.45, 1.76)	0.368	0.89	(0.45, 1.76)	0.369
Q38.1	2.27	(0.75, 6.86)	0.074	2.27	(0.75, 6.86)	0.074
Q38.2	1.02	(0.29, 3.59)	0.489	1.02	(0.29, 3.59)	0.489
Q59.1	12.82	(3.03, 54.3)	<0.001*	12.8	(3.02, 54.14)	<0.001*
Q59.2	7.91	(1.94, 32.2)	0.002*	7.9	(1.94, 32.11)	0.002*
Q59.3	5.7	(1.44, 22.54)	0.007*	5.7	(1.44, 22.58)	0.007*
Q59.4	2.67	(0.14, 50.09)	0.256	2.67	(0.14, 50.06)	0.256
Q145.1	5.67	(0.95, 33.87)	0.029	5.76	(0.96, 34.38)	0.027
Q145.2	0.88	(0.3, 2.53)	0.404	0.88	(0.3, 2.53)	0.404
Q145.3	0.93	(0.19, 4.72)	0.467	0.93	(0.19, 4.7)	0.467
Q145.4	4.34	(0.23, 80.97)	0.163	4.33	(0.23, 80.34)	0.163
Q145.5	0.98	(0.49, 1.94)	0.472	0.98	(0.49, 1.93)	0.472
Q145.6	1.24	(0.59, 2.6)	0.285	1.24	(0.59, 2.59)	0.285
Q146.1	1.56	(0.91, 2.67)	0.052	1.56	(0.91, 2.66)	0.052
Q146.2	2.02	(0.68, 6.04)	0.103	2.03	(0.68, 6.04)	0.102
Q146.3	10.54	(0.37, 296.74)	0.083	10.49	(0.37, 295.39)	0.084
Q146.4	6.12	(1.63, 22.93)	0.004*	6.12	(1.64, 22.9)	0.004*
Q146.5	1.66	(0.96, 2.9)	0.036	1.66	(0.96, 2.89)	0.036
Q146.6	1.68	(0.93, 3.03)	0.042	1.68	(0.93, 3.02)	0.042
Q147.1	1.5	(0.63, 3.61)	0.181	1.5	(0.63, 3.61)	0.181
Q147.2	4.26	(1.83, 9.89)	<0.001*	4.26	(1.84, 9.89)	<0.001*
Q147.3	0.43	(0.03, 6.78)	0.274	0.43	(0.03, 6.73)	0.273
Q147.4	0.81	(0.05, 13.18)	0.44	0.81	(0.05, 13.09)	0.439
Q125.1	0.37	(0.02, 5.89)	0.241	0.37	(0.02, 5.85)	0.24

S5 Table (continued)

	Adjustment 1† (Main Analysis)			Adjustment 2‡ (Sensitivity Analysis)		
Variable	OR	1 – α CI**	PV	OR	1 – α CI**	PV
Q125.2	0.72	(0.04, 11.74)	0.41	0.72	(0.04, 11.64)	0.41
Q125.3	1.66	(0.47, 5.91)	0.217	1.66	(0.47, 5.89)	0.216
Q125.4	0.73	(0.4, 1.31)	0.144	0.73	(0.41, 1.31)	0.143
Q125.5	1.5	(0.3, 7.65)	0.312	1.5	(0.3, 7.62)	0.312
Q125.6	2.26	(0.44, 11.54)	0.164	2.25	(0.44, 11.48)	0.164
Q125.7	2.88	(1.27, 6.54)	0.006*	2.88	(1.27, 6.53)	0.006*
Q125.8	2.38	(0.79, 7.1)	0.061	2.37	(0.79, 7.08)	0.061
Q125.9	2.14	(1.03, 4.42)	0.02*	2.14	(1.04, 4.41)	0.02*
Q127.1	1.47	(0.09, 24.42)	0.394	1.47	(0.09, 24.27)	0.393
Q127.2	0.78	(0.05, 12.37)	0.429	0.78	(0.05, 12.26)	0.429
Q127.3	1.28	(0.44, 3.74)	0.325	1.28	(0.44, 3.73)	0.325
Q127.4	0.89	(0.31, 2.59)	0.417	0.89	(0.31, 2.59)	0.417
Q127.5	0.65	(0.22, 1.87)	0.21	0.65	(0.22, 1.86)	0.209
Q36.live.alone	1.07	(0.53, 2.17)	0.426	1.07	(0.53, 2.17)	0.425
Q36.house.diagnose	38.61	(22.75, 65.52)	<0.001*	38.38	(22.63, 65.11)	<0.001*
Q18.G – Detractor	1.26	(0.72, 2.21)	0.21	1.26	(0.72, 2.21)	0.209
Q18.G – Promoter	1.87	(0.93, 3.78)	0.04	1.87	(0.93, 3.78)	0.04
Q126.1	13.34	(2.3, 77.18)	0.002*	13.32	(2.31, 76.86)	0.002*
Q126.2	0.75	(0.15, 3.74)	0.365	0.76	(0.15, 3.73)	0.365
Q118.1	0.54	(0.29, 1.01)	0.027	0.54	(0.29, 1.01)	0.027
Q118.2	0.85	(0.47, 1.54)	0.3	0.85	(0.47, 1.54)	0.3
Q118.3	1.75	(0.66, 4.66)	0.132	1.75	(0.66, 4.65)	0.132
Q118.4	1.02	(0.29, 3.64)	0.487	1.02	(0.29, 3.63)	0.487
Q118.5	0.45	(0.21, 1)	0.025	0.45	(0.21, 1)	0.025*
Q118.6	1.15	(0.7, 1.87)	0.289	1.15	(0.71, 1.87)	0.29
Q118.7	1.1	(0.7, 1.75)	0.337	1.1	(0.7, 1.75)	0.338
Q133.1	4.37	(1.13, 16.97)	0.016*	4.41	(1.13, 17.15)	0.016*
Q133.2	1.4	(0.39, 5.01)	0.305	1.39	(0.39, 5)	0.305
Q133.3	1.77	(0.77, 4.05)	0.089	1.77	(0.77, 4.05)	0.088
Q28.1	0.65	(0.34, 1.25)	0.098	0.65	(0.34, 1.25)	0.098
Q28.2	1.14	(0.7, 1.83)	0.303	1.14	(0.7, 1.83)	0.302
Q28.3	0.77	(0.37, 1.63)	0.248	0.77	(0.37, 1.63)	0.248
Q28.4	0.77	(0.43, 1.39)	0.193	0.77	(0.43, 1.38)	0.192
Q28.5	0.77	(0.46, 1.3)	0.165	0.77	(0.46, 1.3)	0.164
Q28.6	0.17	(0.01, 2.6)	0.101	0.17	(0.01, 2.58)	0.101
Q28.7	0.62	(0.39, 0.99)	0.022*	0.62	(0.39, 0.99)	0.022*
Q28.8	0.72	(0.45, 1.14)	0.079	0.72	(0.45, 1.14)	0.078
Q28.9	0.85	(0.55, 1.32)	0.236	0.85	(0.55, 1.32)	0.235
Q28.10	0.78	(0.5, 1.21)	0.134	0.78	(0.5, 1.21)	0.134
Q28.11	0.85	(0.33, 2.19)	0.368	0.85	(0.33, 2.18)	0.367
Q28.12	0.87	(0.52, 1.47)	0.305	0.87	(0.52, 1.47)	0.305
Q28.13	0.97	(0.61, 1.55)	0.45	0.97	(0.61, 1.54)	0.449
Q28.14	1	(0.64, 1.56)	0.497	1	(0.64, 1.56)	0.498
Q28.15	1	(0.64, 1.57)	0.498	1	(0.64, 1.56)	0.497
Q28.16	1.52	(0.92, 2.52)	0.051	1.52	(0.92, 2.52)	0.051
Q28.17	0.85	(0.4, 1.79)	0.332	0.85	(0.4, 1.79)	0.331
Q28.18	1.24	(0.35, 4.37)	0.368	1.24	(0.35, 4.36)	0.368
Q117.face	2.02	(0.76, 5.42)	0.081	2.02	(0.75, 5.41)	0.081
Q117.jaw	1.2	(0.46, 3.13)	0.352	1.2	(0.46, 3.12)	0.352
Q117.breast	3.12	(1.45, 6.73)	0.002*	3.12	(1.45, 6.72)	0.002*
Q117.arm	1.57	(0.81, 3.02)	0.09	1.57	(0.81, 3.02)	0.089
Q117.hand	1.4	(0.77, 2.52)	0.135	1.4	(0.77, 2.53)	0.134
Q117.abdomen	1.97	(1.06, 3.66)	0.015*	1.97	(1.07, 3.66)	0.015*
Q117.groin	0.65	(0.19, 2.26)	0.249	0.65	(0.19, 2.25)	0.249
Q117.leg	1.08	(0.66, 1.78)	0.374	1.09	(0.66, 1.78)	0.373
Q117.foot	1.52	(0.91, 2.55)	0.056	1.52	(0.91, 2.55)	0.056
Q117.head	0.92	(0.43, 1.96)	0.413	0.92	(0.43, 1.96)	0.412
Q117.neck	0.86	(0.48, 1.54)	0.304	0.86	(0.48, 1.54)	0.304
Q117.shoulder	1.73	(1.06, 2.83)	0.015*	1.73	(1.06, 2.82)	0.015*

S5 Table (continued)

	Adjustment 1† (Main Analysis)			Adjustment 2‡ (Sensitivity Analysis)		
Variable	OR	1 – α CI**	PV	OR	1 – α CI**	PV
Q117.back	0.97	(0.61, 1.55)	0.446	0.97	(0.61, 1.55)	0.446
Q117.hip	1.36	(0.82, 2.27)	0.117	1.36	(0.82, 2.27)	0.117
Q117.buttocks	0.73	(0.28, 1.88)	0.257	0.73	(0.29, 1.87)	0.257
**EHR Variables**						
Respiratory Condition	2.07	(1.24, 3.48)	0.003*	2.08	(1.24, 3.48)	0.003*
Circulatory Condition	1.45	(0.81, 2.59)	0.107	1.45	(0.81, 2.59)	0.107
Any Cancer	0.88	(0.55, 1.42)	0.301	0.88	(0.55, 1.42)	0.301
Type II Diabetes	1.15	(0.64, 2.04)	0.322	1.15	(0.65, 2.03)	0.322
Kidney Disease	1.39	(0.73, 2.61)	0.157	1.39	(0.74, 2.61)	0.156
Liver Disease	2.57	(1.47, 4.49)	<0.001*	2.57	(1.47, 4.48)	<0.001*
Autoimmune Disease	1.57	(0.96, 2.57)	0.035	1.57	(0.96, 2.57)	0.035
Comorbidity Score	1.26	(1.08, 1.47)	0.002*	1.26	(1.08, 1.47)	0.002*
Smoker – Past	0.72	(0.39, 1.34)	0.151	0.72	(0.39, 1.34)	0.151
Smoker – Current	1.81	(0.98, 3.33)	0.028	1.81	(0.99, 3.34)	0.028
Drinker	1.67	(0.9, 3.09)	0.053	1.67	(0.9, 3.1)	0.052
Neighborhood Education	1.01	(0.8, 1.28)	0.463	1.01	(0.76, 1.36)	0.468
Neighborhood Unemployment	1.13	(0.91, 1.41)	0.137	1.21	(0.92, 1.6)	0.09
Neighborhood Disadvantage	1.01	(0.81, 1.26)	0.479	-	-	-
Population Density	1.22	(1.02, 1.46)	0.016*	1.25	(1.03, 1.52)	0.013*
Neighborhood Poverty	0.92	(0.59, 1.45)	0.513	0.58	(0.19, 1.82)	0.085

All odds ratios are Firth bias-corrected and combined from 30 multiply imputed datasets using Rubin's Rule's. †Adjustment 1: Models adjust for Age, Race/Ethnicity, Sex, BMI, Essential Worker Status, and Education as covariates. ‡Adjustment 2: Models additionally adjust for Neighborhood Disadvantage Index. *p Value statistically significant at 1 – α level. **For covariates, α = 0.05. For other variables, α = 0.05 / k, where k = 184 for Adjustment 1 models and k = 183 for Adjustment 2 models.

S6 Table. Single-Predictor Model Odds Ratios for COVID-19 Self-Diagnosis

	Adjustment 1† (Main Analysis)			Adjustment 2‡ (Sensitivity Analysis)		
Variable	OR	1 – α CI**	PV	OR	1 – α CI**	PV
**Covariates**						
Age (per 10 years)	0.89	(0.79, 1)	0.028	0.88	(0.78, 1)	0.021*
Race/Ethnicity – NHB	1.39	(0.53, 3.62)	0.249	1.56	(0.6, 4.11)	0.181
Race/Ethnicity – Other	0.8	(0.31, 2.07)	0.326	0.8	(0.31, 2.05)	0.321
Essential Worker	1.37	(0.92, 2.04)	0.061	1.38	(0.93, 2.06)	0.057
Education – Advanced	0.99	(0.65, 1.5)	0.473	0.97	(0.64, 1.48)	0.446
Education – Associate	0.93	(0.55, 1.57)	0.386	0.95	(0.56, 1.61)	0.42
Education – HS or Less	1.03	(0.62, 1.71)	0.451	1.08	(0.65, 1.79)	0.39
Sex	1.28	(0.88, 1.85)	0.098	1.28	(0.89, 1.85)	0.094
BMI	1	(0.97, 1.02)	0.362	1	(0.97, 1.02)	0.413
Neighborhood disadvantage	-	-	-	0.87	(0.71, 1.06)	0.083
**Survey Variables**						
Q130	1.1	(0.77, 1.56)	0.3	1.1	(0.77, 1.55)	0.303
Q38	1.73	(1.09, 2.74)	0.01*	1.69	(1.07, 2.69)	0.013*
Q51	1.74	(0.82, 3.68)	0.075	1.69	(0.8, 3.58)	0.085
Q59	0.85	(0.24, 2.97)	0.397	0.83	(0.24, 2.91)	0.386
Q13	1.47	(1.08, 2)	0.007*	1.47	(1.09, 2)	0.006*
Q46	0.84	(0.54, 1.3)	0.214	0.83	(0.53, 1.28)	0.197
Q16	0.94	(0.79, 1.13)	0.268	0.94	(0.79, 1.13)	0.263
Q17	1.18	(0.66, 2.1)	0.293	1.18	(0.66, 2.11)	0.286
Q18	0.93	(0.88, 0.99)	0.008*	0.93	(0.88, 0.99)	0.008*
Q23.1	0.63	(0.45, 0.89)	0.004*	0.63	(0.45, 0.89)	0.004*
Q23.2	1.4	(0.93, 2.09)	0.051	1.43	(0.95, 2.13)	0.042
Q23.3	1.35	(0.84, 2.18)	0.105	1.37	(0.85, 2.2)	0.099
Q23.4	1.15	(0.82, 1.63)	0.206	1.16	(0.82, 1.63)	0.202
Q23.5	0.98	(0.69, 1.37)	0.443	0.98	(0.69, 1.37)	0.446
Q24 – Agree	0.83	(0.54, 1.29)	0.204	0.84	(0.54, 1.29)	0.212
Q24 – Disagree	0.87	(0.54, 1.41)	0.287	0.87	(0.54, 1.41)	0.288
Q27 – Agree	2.24	(0.67, 7.52)	0.096	2.2	(0.65, 7.37)	0.102
Q27 – Disagree	0.94	(0.43, 2.1)	0.444	0.94	(0.43, 2.09)	0.441
Q45 – Agree	1.3	(0.75, 2.24)	0.172	1.3	(0.76, 2.24)	0.171
Q45 – Disagree	1.12	(0.69, 1.8)	0.322	1.11	(0.69, 1.79)	0.327
Q81	3.53	(2.08, 5.98)	<0.001*	3.48	(2.05, 5.9)	<0.001*
Q85	3.49	(2, 6.11)	<0.001*	3.45	(1.97, 6.03)	<0.001*
Q133	1.89	(0.35, 10.22)	0.229	1.88	(0.35, 10.15)	0.231
Q66 – High	1.05	(0.72, 1.53)	0.392	1.02	(0.7, 1.49)	0.467
Q66 – Low	0.58	(0.31, 1.1)	0.049	0.6	(0.32, 1.15)	0.062
Q150	1.2	(1.03, 1.39)	0.009*	1.18	(1.02, 1.37)	0.015*
Q151	0.88	(0.34, 2.26)	0.398	0.93	(0.36, 2.39)	0.443
Q152 – Family-Owned	0.66	(0.29, 1.49)	0.16	0.68	(0.3, 1.53)	0.174
Q152 – Other	0.69	(0.2, 2.39)	0.278	0.72	(0.21, 2.49)	0.299
Q152 – Rent	0.83	(0.47, 1.48)	0.267	0.9	(0.5, 1.62)	0.366
Q68.1	1.15	(0.78, 1.7)	0.236	1.18	(0.8, 1.74)	0.206
Q68.2	1.59	(1.02, 2.46)	0.02*	1.6	(1.03, 2.49)	0.018*
Q68.3	1.35	(0.93, 1.96)	0.06	1.37	(0.94, 1.99)	0.052
Q70.1	1.77	(1.23, 2.55)	0.001*	1.8	(1.25, 2.6)	0.001*
Q70.2	1.75	(1.23, 2.51)	0.001*	1.79	(1.25, 2.56)	0.001*
Q70.3	2.21	(1.51, 3.22)	<0.001*	2.22	(1.52, 3.25)	<0.001*
Q71.1 – Much	2.03	(1.13, 3.68)	0.009*	2.07	(1.15, 3.75)	0.008*
Q71.1 – Some	1.25	(0.84, 1.85)	0.135	1.29	(0.87, 1.91)	0.106
Q71.2 – Much	1.21	(0.6, 2.44)	0.299	1.24	(0.61, 2.5)	0.277
Q71.2 – Some	1.17	(0.78, 1.75)	0.227	1.2	(0.8, 1.8)	0.188
Q71.3 – Much	1.2	(0.58, 2.46)	0.312	1.24	(0.6, 2.55)	0.278
Q71.3 – Some	1.16	(0.73, 1.82)	0.265	1.19	(0.75, 1.87)	0.23
Q71.4 – Much	0.92	(0.36, 2.37)	0.43	0.94	(0.36, 2.42)	0.446
Q71.4 – Some	1.34	(0.87, 2.07)	0.093	1.38	(0.89, 2.13)	0.074
Q72.1 – Often	1.72	(0.72, 4.11)	0.112	1.77	(0.74, 4.25)	0.099
Q72.1 – Sometimes	1.53	(0.93, 2.53)	0.049	1.57	(0.95, 2.59)	0.04
Q72.2 – Often	1.37	(0.69, 2.7)	0.185	1.41	(0.71, 2.8)	0.16

S6 Table (continued)

	Adjustment 1† (Main Analysis)			Adjustment 2‡ (Sensitivity Analysis)		
Variable	OR	1 – α CI**	PV	OR	1 – α CI**	PV
Q72.2 – Sometimes	1.37	(0.87, 2.15)	0.087	1.39	(0.88, 2.18)	0.076
Q72.3 – Often	1.28	(0.69, 2.36)	0.214	1.32	(0.71, 2.43)	0.188
Q72.3 – Sometimes	1.54	(1.04, 2.28)	0.016*	1.56	(1.05, 2.31)	0.014*
Q72.4 – Often	1.16	(0.52, 2.6)	0.361	1.19	(0.53, 2.68)	0.334
Q72.4 – Sometimes	1.34	(0.83, 2.15)	0.114	1.37	(0.85, 2.19)	0.099
Q74.1 – Often	1.52	(0.79, 2.94)	0.105	1.55	(0.8, 2.99)	0.095
Q74.1 – Sometimes	1.31	(0.85, 2.01)	0.108	1.32	(0.86, 2.02)	0.102
Q74.2 – Often	0.97	(0.43, 2.18)	0.469	0.99	(0.44, 2.23)	0.491
Q74.2 – Sometimes	1.32	(0.82, 2.12)	0.126	1.34	(0.84, 2.15)	0.112
Q74.3 – Often	1.28	(0.62, 2.66)	0.252	1.31	(0.63, 2.71)	0.233
Q74.3 – Sometimes	1.08	(0.65, 1.78)	0.383	1.09	(0.66, 1.79)	0.368
Q74.4 – Often	1.26	(0.69, 2.28)	0.227	1.28	(0.7, 2.32)	0.212
Q74.4 – Sometimes	1.37	(0.92, 2.04)	0.061	1.38	(0.93, 2.05)	0.057
Q77	1.63	(1.09, 2.45)	0.009*	1.67	(1.11, 2.51)	0.007*
Q80.1 – Often	1.74	(0.97, 3.1)	0.032	1.69	(0.95, 3.02)	0.038
Q80.1 – Sometimes	2.5	(1.18, 5.33)	0.009*	2.5	(1.18, 5.31)	0.009*
Q80.2 – Often	1.39	(0.72, 2.68)	0.164	1.35	(0.7, 2.61)	0.184
Q80.2 – Sometimes	1.23	(0.47, 3.17)	0.337	1.21	(0.47, 3.13)	0.347
Q80.3 – Often	1.49	(0.83, 2.67)	0.088	1.45	(0.81, 2.6)	0.104
Q80.3 – Sometimes	1.26	(0.56, 2.85)	0.289	1.26	(0.56, 2.84)	0.291
Q80.4 – Often	1.36	(0.7, 2.62)	0.181	1.32	(0.68, 2.55)	0.203
Q80.4 – Sometimes	1.33	(0.57, 3.12)	0.257	1.32	(0.56, 3.09)	0.263
Q141	0.77	(0.32, 1.85)	0.28	0.75	(0.31, 1.8)	0.262
Q145	1.22	(0.79, 1.86)	0.185	1.23	(0.8, 1.88)	0.176
Q146	1.36	(0.95, 1.96)	0.047	1.37	(0.95, 1.97)	0.045
Q147	0.97	(0.54, 1.74)	0.456	1	(0.56, 1.79)	0.497
Q125	0.87	(0.59, 1.28)	0.241	0.87	(0.59, 1.28)	0.244
Q127	0.98	(0.58, 1.65)	0.468	0.99	(0.59, 1.66)	0.479
Q40 – Current User	0.49	(0.19, 1.26)	0.069	0.5	(0.2, 1.3)	0.079
Q40 – Former User	1.02	(0.69, 1.5)	0.46	1.03	(0.7, 1.52)	0.436
Q114.1	1.1	(1.03, 1.17)	0.001*	1.1	(1.04, 1.17)	0.001*
Q114.2	1.11	(1.03, 1.2)	0.002*	1.12	(1.04, 1.2)	0.001*
Q56.1	0.96	(0.91, 1.02)	0.117	0.96	(0.91, 1.02)	0.111
Q56.2	0.99	(0.98, 1.01)	0.168	0.99	(0.98, 1.01)	0.161
Q88 – Monthly	1.01	(0.52, 1.99)	0.485	1.01	(0.52, 1.99)	0.484
Alcohol – Weekly or More	0.8	(0.45, 1.45)	0.233	0.81	(0.45, 1.45)	0.235
Q38.1	1.3	(0.44, 3.83)	0.316	1.26	(0.43, 3.7)	0.339
Q38.2	2.18	(1.03, 4.62)	0.021*	2.14	(1.01, 4.53)	0.024*
Q59.1	3.27	(0.59, 18.02)	0.087	3.33	(0.6, 18.34)	0.083
Q59.2	0.85	(0.05, 14.78)	0.457	0.84	(0.05, 14.51)	0.452
Q59.3	2.42	(0.45, 12.91)	0.15	2.3	(0.43, 12.31)	0.165
Q59.4	1.47	(0.08, 26.43)	0.397	1.38	(0.08, 24.86)	0.414
Q145.1	1.26	(0.07, 21.88)	0.437	1.42	(0.08, 24.34)	0.404
Q145.2	1.14	(0.57, 2.28)	0.358	1.15	(0.57, 2.29)	0.35
Q145.3	0.61	(0.12, 3.03)	0.274	0.62	(0.13, 3.08)	0.28
Q145.4	2.01	(0.11, 36.19)	0.319	2.02	(0.11, 36.3)	0.317
Q145.5	1.51	(0.94, 2.41)	0.043	1.52	(0.95, 2.42)	0.041
Q145.6	1.48	(0.86, 2.55)	0.079	1.5	(0.87, 2.58)	0.073
Q146.1	1.42	(0.92, 2.18)	0.057	1.43	(0.93, 2.19)	0.053
Q146.2	0.87	(0.3, 2.54)	0.401	0.9	(0.31, 2.62)	0.425
Q146.3	4.6	(0.19, 111.27)	0.174	4.51	(0.19, 108.13)	0.176
Q146.4	0.45	(0.03, 7.23)	0.286	0.46	(0.03, 7.44)	0.294
Q146.5	1.32	(0.85, 2.03)	0.107	1.33	(0.86, 2.05)	0.101
Q146.6	1.18	(0.73, 1.91)	0.244	1.19	(0.74, 1.91)	0.242
Q147.1	1.11	(0.55, 2.24)	0.384	1.16	(0.57, 2.33)	0.341
Q147.2	0.48	(0.1, 2.36)	0.181	0.49	(0.1, 2.42)	0.19
Q147.3	1.69	(0.58, 4.96)	0.169	1.69	(0.58, 4.96)	0.168
Q147.4	1.29	(0.25, 6.53)	0.381	1.3	(0.26, 6.6)	0.375
Q125.1	0.88	(0.25, 3.11)	0.421	0.89	(0.25, 3.14)	0.427

S6 Table (continued)

Variable	Adjustment 1† (Main Analysis) OR	1 – α CI**	PV	Adjustment 2‡ (Sensitivity Analysis) OR	1 – α CI**	PV
Q125.2	0.86	(0.17, 4.37)	0.427	0.88	(0.17, 4.48)	0.44
Q125.3	0.45	(0.09, 2.24)	0.166	0.45	(0.09, 2.25)	0.167
Q125.4	0.83	(0.54, 1.27)	0.193	0.83	(0.55, 1.27)	0.195
Q125.5	1.17	(0.33, 4.12)	0.406	1.17	(0.33, 4.13)	0.402
Q125.6	0.88	(0.17, 4.41)	0.437	0.88	(0.18, 4.42)	0.439
Q125.7	0.95	(0.37, 2.42)	0.455	0.96	(0.38, 2.46)	0.469
Q125.8	0.16	(0.01, 2.51)	0.096	0.16	(0.01, 2.54)	0.098
Q125.9	1.51	(0.84, 2.73)	0.084	1.51	(0.84, 2.73)	0.083
Q127.1	3.43	(0.94, 12.54)	0.031	3.39	(0.93, 12.36)	0.032
Q127.2	1.73	(0.49, 6.15)	0.198	1.75	(0.49, 6.21)	0.193
Q127.3	0.76	(0.3, 1.94)	0.283	0.76	(0.3, 1.94)	0.284
Q127.4	0.91	(0.44, 1.9)	0.402	0.93	(0.44, 1.94)	0.419
Q127.5	0.92	(0.47, 1.78)	0.399	0.92	(0.48, 1.79)	0.408
Q36.live.alone	0.93	(0.54, 1.61)	0.403	0.96	(0.56, 1.65)	0.44
Q36.house.diagnose	10.23	(5.99, 17.47)	<0.001*	10.3	(6.03, 17.6)	<0.001*
Q18.G – Detractor	1.15	(0.77, 1.71)	0.248	1.15	(0.77, 1.71)	0.251
Q18.G – Promoter	1	(0.56, 1.76)	0.495	1	(0.57, 1.77)	0.499
Q126.1	1.6	(0.09, 27.83)	0.373	1.66	(0.1, 28.86)	0.363
Q126.2	1.32	(0.56, 3.11)	0.263	1.34	(0.57, 3.16)	0.25
Q118.1	0.75	(0.48, 1.17)	0.102	0.74	(0.47, 1.15)	0.089
Q118.2	1.12	(0.72, 1.74)	0.315	1.11	(0.72, 1.73)	0.316
Q118.3	0.42	(0.08, 2.08)	0.144	0.43	(0.09, 2.12)	0.149
Q118.4	0.41	(0.08, 2.04)	0.139	0.43	(0.09, 2.1)	0.147
Q118.5	0.55	(0.31, 0.99)	0.023*	0.55	(0.31, 0.98)	0.022*
Q118.6	0.97	(0.65, 1.43)	0.431	0.96	(0.65, 1.42)	0.418
Q118.7	1.26	(0.88, 1.81)	0.104	1.26	(0.88, 1.81)	0.103
Q133.1	0.41	(0.03, 6.49)	0.262	0.43	(0.03, 6.89)	0.276
Q133.2	1.14	(0.39, 3.31)	0.408	1.16	(0.4, 3.38)	0.39
Q133.3	0.64	(0.22, 1.86)	0.206	0.66	(0.23, 1.9)	0.219
Q28.1	1.13	(0.58, 2.2)	0.364	1.13	(0.58, 2.21)	0.356
Q28.2	1.08	(0.74, 1.57)	0.344	1.08	(0.74, 1.57)	0.35
Q28.3	0.78	(0.44, 1.4)	0.203	0.79	(0.44, 1.4)	0.208
Q28.4	0.91	(0.59, 1.39)	0.329	0.92	(0.6, 1.41)	0.345
Q28.5	1.39	(0.97, 1.97)	0.035	1.39	(0.97, 1.97)	0.035
Q28.6	1.48	(0.71, 3.11)	0.149	1.48	(0.71, 3.1)	0.15
Q28.7	1.39	(0.97, 1.97)	0.034	1.4	(0.98, 1.99)	0.032
Q28.8	1.11	(0.79, 1.56)	0.277	1.11	(0.79, 1.56)	0.275
Q28.9	1.24	(0.88, 1.76)	0.106	1.24	(0.88, 1.75)	0.109
Q28.10	1.21	(0.84, 1.74)	0.149	1.22	(0.85, 1.74)	0.144
Q28.11	0.96	(0.48, 1.92)	0.451	0.96	(0.48, 1.93)	0.458
Q28.12	1.09	(0.73, 1.61)	0.338	1.1	(0.74, 1.62)	0.325
Q28.13	1.22	(0.86, 1.72)	0.135	1.21	(0.86, 1.72)	0.137
Q28.14	1.03	(0.73, 1.45)	0.439	1.04	(0.73, 1.46)	0.419
Q28.15	0.98	(0.69, 1.38)	0.446	0.98	(0.69, 1.38)	0.446
Q28.16	1.67	(1.15, 2.44)	0.004*	1.67	(1.15, 2.44)	0.004*
Q28.17	1.46	(0.91, 2.36)	0.059	1.47	(0.91, 2.36)	0.057
Q28.18	1.15	(0.4, 3.33)	0.398	1.17	(0.4, 3.37)	0.387
Q117.face	0.41	(0.08, 2.02)	0.136	0.42	(0.09, 2.07)	0.143
Q117.jaw	1.42	(0.73, 2.77)	0.15	1.48	(0.76, 2.87)	0.126
Q117.breast	1.14	(0.49, 2.68)	0.382	1.17	(0.5, 2.74)	0.361
Q117.arm	1.75	(1.11, 2.78)	0.008*	1.8	(1.14, 2.85)	0.006*
Q117.hand	1.36	(0.88, 2.09)	0.081	1.39	(0.9, 2.14)	0.068
Q117.abdomen	1.6	(0.96, 2.66)	0.035	1.64	(0.98, 2.72)	0.029
Q117.groin	1.39	(0.69, 2.79)	0.175	1.41	(0.7, 2.82)	0.167
Q117.leg	1.31	(0.91, 1.89)	0.071	1.33	(0.92, 1.92)	0.062
Q117.foot	1.11	(0.72, 1.69)	0.321	1.12	(0.73, 1.71)	0.301
Q117.head	1.01	(0.57, 1.79)	0.485	1.04	(0.59, 1.84)	0.451
Q117.neck	1.8	(1.23, 2.61)	0.001*	1.83	(1.26, 2.66)	0.001*
Q117.shoulder	1.52	(1.04, 2.22)	0.016*	1.54	(1.05, 2.25)	0.013*

S6 Table (continued)

	Adjustment 1† (Main Analysis)			Adjustment 2‡ (Sensitivity Analysis)		
Variable	OR	1 – α CI**	PV	OR	1 – α CI**	PV
Q117.back	1.6	(1.14, 2.26)	0.004*	1.62	(1.15, 2.29)	0.003*
Q117.hip	1.3	(0.88, 1.91)	0.092	1.32	(0.9, 1.94)	0.08
Q117.buttocks	0.86	(0.45, 1.67)	0.331	0.87	(0.45, 1.69)	0.345
**EHR Variables**						
Respiratory Condition	1.03	(0.72, 1.48)	0.426	1.02	(0.72, 1.46)	0.452
Circulatory Condition	0.98	(0.63, 1.53)	0.461	0.97	(0.62, 1.51)	0.449
Any Cancer	0.77	(0.54, 1.11)	0.083	0.78	(0.54, 1.12)	0.088
Type II Diabetes	1.28	(0.84, 1.96)	0.127	1.29	(0.84, 1.96)	0.123
Kidney Disease	1.16	(0.71, 1.89)	0.277	1.17	(0.72, 1.92)	0.26
Liver Disease	0.79	(0.42, 1.49)	0.232	0.79	(0.42, 1.49)	0.234
Autoimmune Disease	1.36	(0.92, 2)	0.06	1.34	(0.91, 1.97)	0.068
Comorbidity Score	1.02	(0.9, 1.16)	0.382	1.02	(0.9, 1.16)	0.391
Smoker – Past	1.1	(0.74, 1.62)	0.318	1.11	(0.75, 1.64)	0.303
Smoker – Current	0.51	(0.23, 1.15)	0.052	0.53	(0.24, 1.18)	0.06
Drinker	1	(0.66, 1.54)	0.492	0.99	(0.64, 1.51)	0.472
Neighborhood Education	0.92	(0.75, 1.12)	0.207	1.01	(0.78, 1.3)	0.476
Neighborhood Unemployment	0.91	(0.75, 1.11)	0.174	0.98	(0.78, 1.24)	0.432
Neighborhood Disadvantage	0.87	(0.71, 1.06)	0.083	-	-	-
Population Density	0.97	(0.81, 1.17)	0.391	1.02	(0.83, 1.24)	0.436
Neighborhood Poverty	0.82	(0.65, 1.02)	0.039	0.74	(0.46, 1.21)	0.115

All odds ratios are Firth bias-corrected and combined from 30 multiply imputed datasets using Rubin's Rule's. †Adjustment 1: Models adjust for Age, Race/Ethnicity, Sex, BMI, Essential Worker Status, and Education as covariates. ‡Adjustment 2: Models additionally adjust for Neighborhood Disadvantage Index. *p Value statistically significant at 1 – α level. **For covariates, α = 0.05. For other variables, α = 0.05 / k, where k = 184 for Adjustment 1 models and k = 183 for Adjustment 2 models.

S7 Table. Sensitivity of Survey-Based Model AUCs to Social Environment

		Mean AUC (95% Empirical CI)							
		Adjustment 1 (Main Analysis) †				Adjustment 2 (Sensitivity Analysis) ‡			
Outcome*	Model Type	Covariates Only	Covariates + EHR Variables	Covariates + Survey Vars.	All Variables	Covariates Only	Covariates + EHR Variables	Covariates + Survey Vars.	All Variables
Tested for COVID-19	Lasso	0.582 (0.552, 0.609)	0.593 (0.569, 0.617)	0.646 (0.621, 0.676)	0.645 (0.619, 0.674)	0.582 (0.554, 0.61)	0.591 (0.562, 0.616)	0.646 (0.617, 0.673)	0.644 (0.617, 0.673)
	Ridge	0.582 (0.552, 0.609)	0.597 (0.57, 0.623)	0.641 (0.618, 0.67)	0.639 (0.616, 0.668)	0.582 (0.554, 0.61)	0.597 (0.569, 0.623)	0.641 (0.618, 0.67)	0.639 (0.616, 0.668)
	Elastic net	0.582 (0.552, 0.609)	0.595 (0.569, 0.62)	0.649 (0.624, 0.678)	0.648 (0.624, 0.676)	0.582 (0.554, 0.61)	0.595 (0.567, 0.622)	0.648 (0.623, 0.677)	0.647 (0.622, 0.676)
Diagnosed with COVID-19	Lasso	0.694 (0.599, 0.774)	0.694 (0.599, 0.774)	0.798 (0.718, 0.885)	0.798 (0.718, 0.885)	0.691 (0.592, 0.774)	0.691 (0.592, 0.774)	0.798 (0.718, 0.885)	0.798 (0.718, 0.885)
	Ridge	0.694 (0.599, 0.774)	0.713 (0.615, 0.793)	0.812 (0.741, 0.885)	0.821 (0.743, 0.887)	0.691 (0.592, 0.774)	0.711 (0.614, 0.793)	0.81 (0.739, 0.885)	0.819 (0.744, 0.887)
	Elastic net	0.694 (0.599, 0.774)	0.709 (0.612, 0.788)	0.802 (0.728, 0.878)	0.804 (0.724, 0.88)	0.691 (0.592, 0.774)	0.707 (0.612, 0.79)	0.802 (0.728, 0.878)	0.804 (0.724, 0.88)
Self-Diagnosed with COVID-19	Lasso	0.537 (0.475, 0.609)	0.537 (0.475, 0.609)	0.585 (0.511, 0.665)	0.585 (0.514, 0.665)	0.544 (0.49, 0.616)	0.544 (0.49, 0.616)	0.595 (0.509, 0.674)	0.595 (0.509, 0.674)
	Ridge	0.537 (0.475, 0.609)	0.539 (0.494, 0.614)	0.605 (0.516, 0.697)	0.607 (0.528, 0.698)	0.544 (0.49, 0.616)	0.54 (0.486, 0.609)	0.609 (0.532, 0.694)	0.606 (0.522, 0.693)
	Elastic net	0.537 (0.475, 0.609)	0.536 (0.483, 0.611)	0.599 (0.501, 0.679)	0.598 (0.506, 0.677)	0.544 (0.49, 0.616)	0.541 (0.492, 0.614)	0.599 (0.501, 0.679)	0.598 (0.506, 0.677)

S8 Table. Comparison of Survey Respondents to Michigan Genomics Initiative and Michigan Medicine

	COVID-19 Survey n = 7,054		Michigan Genomics Initiative n = 82,372		Michigan Medicine n = 3,953,712	
	Number	Percent	Number	Percent	Number	Percent
**Age**						
18-30	379	5.37	8069	9.80	655185	16.57
31-40	689	9.77	8980	10.90	514851	13.02
41-50	907	12.86	11855	14.39	571074	14.44
51-60	1530	21.69	16699	20.27	635522	16.07
61-70	2066	29.29	19523	23.70	587345	14.86
71-80	1284	18.20	12880	15.64	393714	9.96
81+	199	2.83	4365	5.30	596021	15.08
**Sex**						
Male	2831	40.13	37891	45.00	1800540	45.54
Female	4223	59.87	44479	54.00	2102545	53.18
Other	0	0	0	0.00	6406	0.16
Unknown	0	0	2	0.002	44222	1.12
**Race/Ethnicity**						
NHAA	158	2.24	4994	6.06	106744	2.70
NHW	6545	92.78	68341	82.97	950123	24.03
Unknown	90	1.28	3590	4.36	2750156	69.56
Other	261	3.70	5447	6.61	146695	3.71

Michigan Medicine records include patients who received treatment at any point from 01/01/2000 to 07/27/2020 and were over 18 years of age. Note that many self-reported Caucasians did not report an ethnicity, only a race, and therefore the number of unknowns in the Michigan Medicine Race/Ethnicity variable is large. Acronyms: NHAA, Non-Hispanic African American, NHW, Non-Hispanic White

S9 Table. LASSO Model Most Selected Variables

Variable Selection Proportion. Outcome: Received a COVID-19 Test (Self-Reported)					
EHR-Variable Models		Survey-Variable Models		All-Variable Models	
Kidney disease	0.95	Q17. Ever Hospitalized with infection	1.00	Q17. Ever Hospitalized with infection	1.00
Comorbidity score	0.92	Q36. Household member diagnosed with COVID-19	1.00	Q36. Household member diagnosed with COVID-19	1.00
		Q68.1 Felt fatigued in past week	1.00	Q68.1 Felt fatigued in past week	1.00
		Q147.1 Kidney disease	0.99	Q147.1 Kidney disease	0.99
		Q70.1 Abdomen pain in past 6 months	0.98	Q70.1 Abdomen pain in past 6 months	0.98
		Q146.2 COPD	0.96	Q146.2 COPD	0.96
		Q70.3 Headaches in past 6 months	0.96	Q70.3 Headaches in past 6 months	0.95
		Q125. Cardiovascular condition	0.95	Q125. Cardiovascular condition	0.94
		Q13. Number of times gotten flu in past year	0.94	Q13. Number of times gotten flu in past year	0.94
		Q125.7 Blood clotting disorder	0.91	Q125.7 Blood clotting disorder	0.9
		Q59.1. Police officer lives in home	0.87	Q59.1. Police officer lives in home	0.84
		Q23.3 Concerned about losing job	0.82		
**Variable Selection Proportion. Outcome: Diagnosed with COVID-19 (Self-Reported)**					
EHR-Variable Models		Survey-Variable Models		All-Variable Models	
<No Variables Over 0.8>		Q36. Household member diagnosed with COVID-19	1.00	Q36. Household member diagnosed with COVID-19	1.00
**Variable Selection Proportion. Outcome: Self-Diagnosed with COVID-19 (Self-Reported)**					
EHR-Variable Models		Survey-Variable Models		All-Variable Models	
<No Variables Over 0.8>		Q36. Household member diagnosed with COVID-19	1.00	Q36. Household member diagnosed with COVID-19	1.00

The value shown is the proportion of times the variable was chosen in 3,000 fitted models, as models were fit on 1000 train/test splits of 30 multiply imputed datasets (100x30=3,000). Only variables with a selection rate over 80% are included. Variable descriptions are available in the supplement (S1 Table). The tested for COVID-19 outcome compares the tested population (1) to those not tested (0). The diagnosed with COVID-19 outcome compares those diagnosed with COVID-19 by a physician or test (1) to those not diagnosed, not tested, and not self-diagnosed (0). The self-diagnosed with COVID-19 outcome compares those who diagnosed themselves with COVID-19 without a test to those who were not self-diagnosed or formally diagnosed (0). All models included the six covariates age, sex, race/ethnicity, body mass index, education level, and essential worker status, which were not selected for or penalized. Data from Michigan Medicine COVID-19 Survey and Michigan Genomics Initiative. Sample size: 6,159 – 7,054

S10 Table. ENET Model Most-Selected Variables

**Variable Selection Proportion. Outcome: Received a COVID-19 Test (Self-Reported)**					
EHR-Variable Models		Survey-Variable Models		All-Variable Models	
Comorbidity score*	0.99	Q17. Ever Hospitalized with infection	1.00	Q17. Ever Hospitalized with infection	1.00
Kidney disease*	0.98	Q36. Household member diagnosed with COVID-19	1.00	Q36. Household member diagnosed with COVID-19	1.00
Respiratory disease*	0.94	Q147.1 Kidney disease	1.00	Q147.1 Kidney disease	1.00
Liver disease*	0.91	Q68.1 Felt fatigued in past week	1.00	Q68.1 Felt fatigued in past week	1.00
Former smoker*	0.80	Q70.1 Abdomen pain in past 6 months	1.00	Q70.1 Abdomen pain in past 6 months	1.00
		Q70.3 Headaches in past 6 months	1.00	Q70.3 Headaches in past 6 months	0.99
		Q13. No times gotten flu in past year	0.99	Q13. No times gotten flu in past year	0.99
		Q125. Cardiovascular condition	0.98	Q125. Cardiovascular condition	0.98
		Q146.2 COPD	0.98	Q146.2 COPD	0.97
		Q147. Metabolic Condition	0.96	Q125.7 Blood clotting disorder	0.95
		Q125.7 Has cardiovascular condition	0.95	Q147. Metabolic condition	0.94
		Q59.1 Police officer lives in home	0.95	Q59.1 Police officer lives in home	0.94
		Q23.3 Concerned about losing job	0.95	Q114.1 Overall body pain at worst	0.94
		Q71.1 Some difficult doing chores	0.94	Q23.3 Concerned about losing job	0.93
		Q71.1 Much difficulty doing chores	0.94	Q71.1 Much difficulty doing chores	0.93
		Q114.1 Overall body pain at worst	0.94	Q71.1 Some difficulty doing chores	0.93
		Q133.2 Benzodiazepine use has increased	0.88	Q133.2 Benzodiazepine use has increased	0.87
		Q114.2 Overall body pain on average	0.86	Q68.3 Trouble waking up refreshed	0.85
		Q46. Flu shot in past year	0.86	Q77. Poor sleep quality, past 7 days	0.85
		Q77. Poor sleep quality, past 7 days	0.86	Q114.2 Overall body pain, on average	0.85
		Q68.3 Trouble waking up refreshed	0.85	Q133.1 Opioid use has increased	0.83
		Q133.1 Opioid use has increase	0.85	Q46. Flu shot in past year	0.83
		Q36.1. Lives alone	0.83	Q36.1. Lives alone	0.81
		Q68.2 Memory trouble in past week	0.81		
**Variable Selection Proportion. Outcome: Diagnosed with COVID-19 (Self-Reported)**					
EHR-Variable Models		Survey-Variable Models		All-Variable Models	
Liver disease	0.92	Q36. Household member diagnosed with COVID-19	1.00	Q36. Household member diagnosed with COVID-19	1.00
Respiratory disease	0.87	Q85. Relative died from COVID-19	0.85	Q85. Relative died from COVID-19	0.82
		Q70.1 Abdomen pain in past 6 months	0.82	Q81. Relative diagnosed with COVID-19	0.81
		Q81. Relative diagnosed with COVID-19	0.82	Q70.1 Abdomen pain in past 6 months	0.80
**Variable Selection Proportion. Outcome: Self-Diagnosed with COVID-19 (Self-Reported)**					
EHR-Variable Models		Survey-Variable Models		All-Variable Models	
<No Variables Over 0.8>		Q36. Household member diagnosed with COVID-19	1	Q36. Household member diagnosed with COVID-19	1
		Q70.3 Headaches in past 6 months	0.95	Q70.3 Headaches in past 6 months	0.93
		Q81. Relative diagnosed with COVID-19	0.9	Q81. Relative diagnosed with COVID-19	0.89
		Q23.1 Concerned about someone close contracting COVID-19	0.87	Q23.1 Concerned about someone close contracting COVID-19	0.87

S11 Table (continued)

		Q118.5 Sleep habits have improved during pandemic	0.84	Q70.2 Depression in past 6 months	0.83
		Q28.16 Family member has irregular heartbeat	0.83	Q28.16 Family member has irregular heartbeat	0.81
		Q70.2 Depression in past 6 months	0.82	Q118.5 Sleep habits have improved during pandemic	0.81

The value shown is the proportion of times the variable was chosen in 3,000 fitted models, as models were fit on 1000 train/test splits of 30 multiply imputed datasets (100x30=3,000). Only variables with a selection rate over 80% are included. Variable descriptions are available in the supplement (S1 Table). The tested for COVID-19 outcome compares the tested population (1) to those not tested (0). The diagnosed with COVID-19 outcome compares those diagnosed with COVID-19 by a physician or test (1) to those not diagnosed, not tested, and not self-diagnosed (0). The self-diagnosed with COVID-19 outcome compares those who diagnosed themselves with COVID-19 without a test to those who were not self-diagnosed or formally diagnosed (0). All models included the six covariates age, sex, race/ethnicity, body mass index, education level, and essential worker status, which were not selected for or penalized. Data from Michigan Medicine COVID-19 Survey and Michigan Genomics Initiative. Sample size: 6,159 – 7,054

S11 Table. Mean Penalties Selected by Elastic Net Regression Models

**Elastic Net Regression Model Penalties – Outcome: Received a COVID-19 Test**						
	Covariates + EHR Variables		Covariates + Survey Variables		All Variables	
Split	Lambda	Alpha	Lambda	Alpha	Lambda	Alpha
1	0.020	0.247	0.051	0.100	0.057	0.107
2	0.013	0.267	0.052	0.107	0.054	0.140
3	0.017	0.100	0.054	0.160	0.052	0.180
4	0.019	0.213	0.052	0.100	0.055	0.100
5	0.022	0.107	0.049	0.160	0.044	0.213
6	0.012	0.273	0.048	0.200	0.047	0.187
7	0.018	0.120	0.054	0.113	0.056	0.107
8	0.020	0.120	0.039	0.300	0.034	0.360
9	0.021	0.120	0.045	0.187	0.045	0.227
10	0.012	0.407	0.042	0.260	0.039	0.313
**Elastic Net Regression Model Penalties – Outcome: Diagnosed with COVID-19**						
	Covariates + EHR Variables		Covariates + Survey Variables		All Variables	
Split	Lambda	Alpha	Lambda	Alpha	Lambda	Alpha
1	0.007	0.100	0.015	0.893	0.015	0.893
2	0.005	0.100	0.017	0.773	0.018	0.793
3	0.019	0.133	0.016	0.840	0.016	0.860
4	0.005	0.100	0.005	0.700	0.005	0.573
5	0.005	0.107	0.005	0.300	0.005	0.293
6	0.006	0.100	0.020	0.300	0.020	0.300
7	0.005	0.100	0.028	0.260	0.005	0.100
8	0.005	0.100	0.037	0.447	0.039	0.400
9	0.006	0.407	0.039	0.333	0.037	0.347
10	0.025	0.167	0.005	0.900	0.005	0.900
**Elastic Net Regression Model Penalties – Outcome: Self-Diagnosed with COVID-19**						
	Covariates + EHR Variables		Covariates + Survey Variables		All Variables	
Split	Lambda	Alpha	Lambda	Alpha	Lambda	Alpha
1	0.007	0.107	0.009	0.153	0.009	0.140
2	0.042	0.107	0.019	0.127	0.020	0.100
3	0.032	0.107	0.005	0.300	0.005	0.293
4	0.066	0.113	0.005	0.627	0.005	0.667
5	0.072	0.133	0.006	0.793	0.005	0.900
6	0.006	0.167	0.006	0.100	0.008	0.160
7	0.020	0.140	0.005	0.500	0.005	0.500
8	0.017	0.100	0.019	0.100	0.019	0.100
9	0.040	0.147	0.006	0.100	0.009	0.100
10	0.064	0.120	0.005	0.420	0.005	0.520

Lambda and alpha shown are the mean across 30 models fit on multiply imputed datasets. Lambda and alpha were selected by five-fold cross-validation on the training set of a single 70/30 train/test split.

S12 Table. Mean Penalties Selected by Ridge Regression Models

**Ridge Regression Model Penalties – Outcome: Received a COVID-19 Test**						
	Covariates + EHR Variables		Covariates + Survey Variables		All Variables	
Split	Lambda	Alpha	Lambda	Alpha	Lambda	Alpha
1	0.069	0.000	0.100	0.000	0.100	0.000
2	0.074	0.000	0.100	0.000	0.100	0.000
3	0.060	0.000	0.100	0.000	0.100	0.000
4	0.072	0.000	0.100	0.000	0.100	0.000
5	0.062	0.000	0.100	0.000	0.100	0.000
6	0.044	0.000	0.100	0.000	0.100	0.000
7	0.057	0.000	0.100	0.000	0.100	0.000
8	0.063	0.000	0.100	0.000	0.100	0.000
9	0.051	0.000	0.100	0.000	0.100	0.000
10	0.052	0.000	0.100	0.000	0.100	0.000
**Ridge Regression Model Penalties – Outcome: Diagnosed with COVID-19**						
	Covariates + EHR Variables		Covariates + Survey Variables		All Variables	
Split	Lambda	Alpha	Lambda	Alpha	Lambda	Alpha
1	0.019	0.000	0.061	0.000	0.056	0.000
2	0.006	0.000	0.100	0.000	0.099	0.000
3	0.061	0.000	0.098	0.000	0.100	0.000
4	0.005	0.000	0.075	0.000	0.068	0.000
5	0.010	0.000	0.036	0.000	0.035	0.000
6	0.013	0.000	0.100	0.000	0.099	0.000
7	0.005	0.000	0.023	0.000	0.015	0.000
8	0.016	0.000	0.099	0.000	0.089	0.000
9	0.034	0.000	0.098	0.000	0.100	0.000
10	0.064	0.000	0.039	0.000	0.044	0.000
**Ridge Regression Model Penalties – Outcome: Self-Diagnosed with COVID-19**						
	Covariates + EHR Variables		Covariates + Survey Variables		All Variables	
Split	Lambda	Alpha	Lambda	Alpha	Lambda	Alpha
1	0.019	0.000	0.042	0.000	0.049	0.000
2	0.049	0.000	0.066	0.000	0.071	0.000
3	0.061	0.000	0.031	0.000	0.039	0.000
4	0.037	0.000	0.064	0.000	0.066	0.000
5	0.069	0.000	0.033	0.000	0.040	0.000
6	0.023	0.000	0.024	0.000	0.025	0.000
7	0.050	0.000	0.055	0.000	0.061	0.000
8	0.021	0.000	0.057	0.000	0.053	0.000
9	0.068	0.000	0.023	0.000	0.026	0.000
10	0.084	0.000	0.082	0.000	0.083	0.000

Lambda and alpha shown are the mean across 30 models fit on multiply imputed datasets. Lambda and alpha were selected by five-fold cross-validation on the training set of a single 70/30 train/test split.

S13 Table. Mean Penalties Selected by Lasso Regression Models

**Lasso Regression Model Penalties – Outcome: Received a COVID-19 Test**						
	Covariates + EHR Variables		Covariates + Survey Variables		All Variables	
Split	Lambda	Alpha	Lambda	Alpha	Lambda	Alpha
1	0.005	1.000	0.007	1.000	0.007	1.000
2	0.006	1.000	0.007	1.000	0.008	1.000
3	0.008	1.000	0.008	1.000	0.008	1.000
4	0.008	1.000	0.006	1.000	0.006	1.000
5	0.008	1.000	0.007	1.000	0.007	1.000
6	0.005	1.000	0.007	1.000	0.007	1.000
7	0.008	1.000	0.006	1.000	0.006	1.000
8	0.005	1.000	0.008	1.000	0.008	1.000
9	0.005	1.000	0.006	1.000	0.007	1.000
10	0.009	1.000	0.009	1.000	0.009	1.000
**Lasso Regression Model Penalties – Outcome: Diagnosed with COVID-19**						
	Covariates + EHR Variables		Covariates + Survey Variables		All Variables	
Split	Lambda	Alpha	Lambda	Alpha	Lambda	Alpha
1	0.100	1.000	0.015	1.000	0.015	1.000
2	0.100	1.000	0.015	1.000	0.015	1.000
3	0.100	1.000	0.017	1.000	0.017	1.000
4	0.100	1.000	0.005	1.000	0.005	1.000
5	0.100	1.000	0.010	1.000	0.010	1.000
6	0.100	1.000	0.010	1.000	0.010	1.000
7	0.100	1.000	0.005	1.000	0.005	1.000
8	0.100	1.000	0.020	1.000	0.020	1.000
9	0.100	1.000	0.020	1.000	0.020	1.000
10	0.100	1.000	0.005	1.000	0.005	1.000
**Lasso Regression Model Penalties – Outcome: Self-Diagnosed with COVID-19**						
	Covariates + EHR Variables		Covariates + Survey Variables		All Variables	
Split	Lambda	Alpha	Lambda	Alpha	Lambda	Alpha
1	0.100	1.00	0.006	1.000	0.006	1.000
2	0.100	1.00	0.006	1.000	0.006	1.000
3	0.100	1.00	0.005	1.000	0.005	1.000
4	0.100	1.00	0.008	1.000	0.008	1.000
5	0.100	1.00	0.006	1.000	0.006	1.000
6	0.100	1.00	0.005	1.000	0.005	1.000
7	0.100	1.00	0.007	1.000	0.007	1.000
8	0.100	1.00	0.006	1.000	0.007	1.000
9	0.100	1.00	0.006	1.000	0.006	1.000
10	0.100	1.00	0.007	1.000	0.007	1.000

Lambda and alpha shown are the mean across 30 models fit on multiply imputed datasets. Lambda and alpha were selected by five-fold cross-validation on the training set of a single 70/30 train/test split.

EHR-Based Supplementary Analysis

To see how the 15 EHR-derived variables would perform in the larger cohort that was not surveyed, we ran similar models using COVID-19 testing and diagnosis case-control data from Michigan Medicine. To construct the COVID-19 tested cases, we retrieved data for all 15,929 patients who had obtained a reverse transcription polymerase chain reaction (RT-PCR) test for SARS-CoV-2 at Michigan Medicine between March 10th and June 30th, 2020 (Figure 1). For COVID-19 diagnosed cases, we used the 1,193 who had tested positive, along with another 290 patients who had had COVID-19 per their EHRs but had no test results (this latter group would have included, for example, patients who were treated for COVID-19 at Michigan Medicine but were not tested there). This resulted in a total of 1,483 diagnosed cases for the analysis. Lastly, for controls, we extracted data for 30,000 random patients who were alive, were not in the tested or diagnosed groups, and had an encounter in Michigan Medicine (Inpatient, Outpatient, or Emergency) between April 23, 2012, and June 21, 2020.

Figure 1. Extraction of Michigan Case-Control Records for COVID-19-related Outcomes

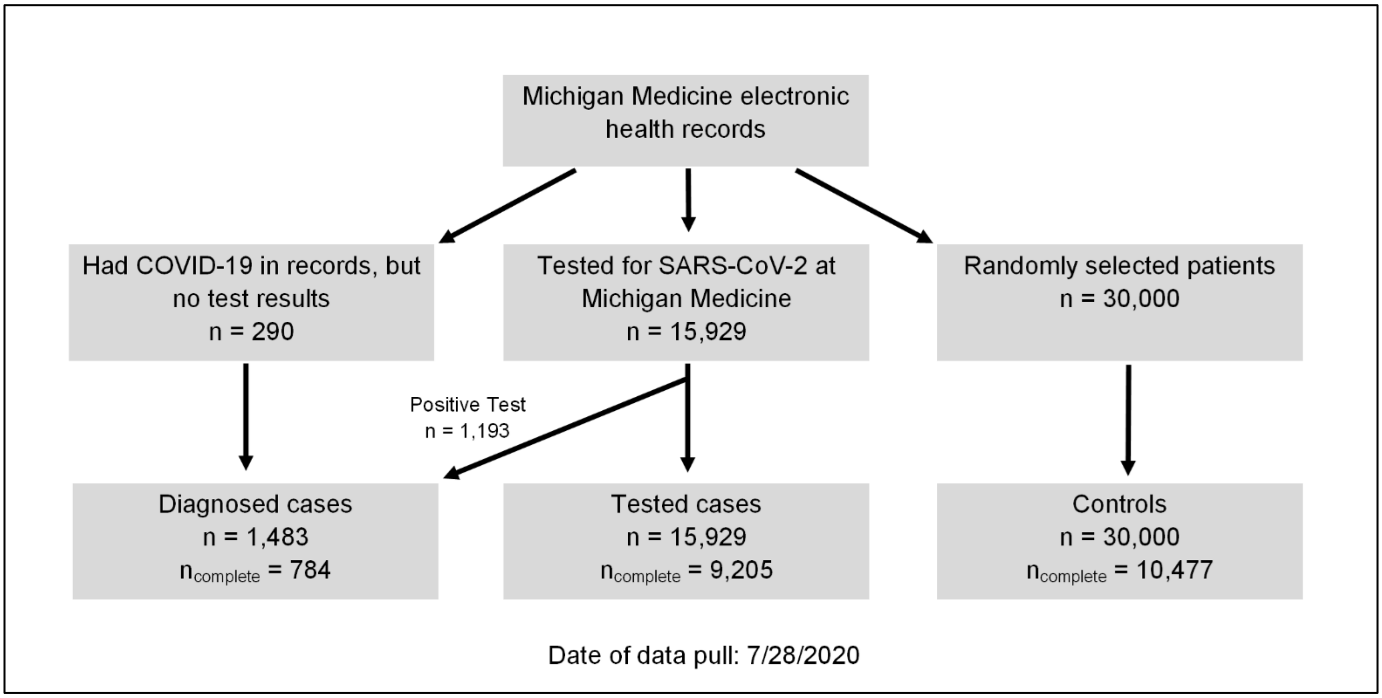

Patients were coded as “1” for the testing models if they were tested for COVID-19 at Michigan Medicine, and “1” for the diagnosis models if they tested positive or were diagnosed with COVID-19. Random controls

were coded as “0”. We used the same covariates as in the survey-based models except for education and essential worker status, which were not available. In addition, we attempted two different constructions of the health condition indicator variables: A *restricted* version, which limited health conditions to pre-existing conditions (those appearing at least 14 days prior to the patient’s first COVID-19 diagnosis or test, if they had one), and an *unrestricted* version, which included health conditions appearing up to *the day of* that patient’s first COVID-19 test or diagnosis (Figure 2A). This way, the EHR-derived variables in the unrestricted data would also be able to capture health conditions that occurred as a result of COVID-19 infection, rather than only those that existed beforehand, as symptoms usually appear within 14 days of infection [25]. Using both datasets separately, we applied similar models to our main analysis: a ridge regression, lasso regression, and elastic net regression (Figure 2B). All models adjusted for the four covariates age, sex, race/ethnicity, and body mass index, which were not selected for or penalized. Tuning parameters were selected using five-fold cross-validation. As in the main analysis, models were evaluated internally by computing the test set AUC of 100 random train/test splits and taking the average of those 100, along with the 2.5th and 97.5th percentiles for an empirical confidence interval. Note that, since the degree of missingness in the data was high, but sample size was large, we elected to conduct a complete case analysis rather than pursuing multiple imputation, our approach for the survey-based analysis described in the main text.

Figure 2. Analysis Overview of Michigan Medicine Case-Control Data

(A) Health condition predictor variables were defined differently for two separate analyses, a restricted version and an unrestricted version. (B) Multivariable logistic models were used to assess how well EHR-derived variables could discriminate COVID-19 cases from controls.

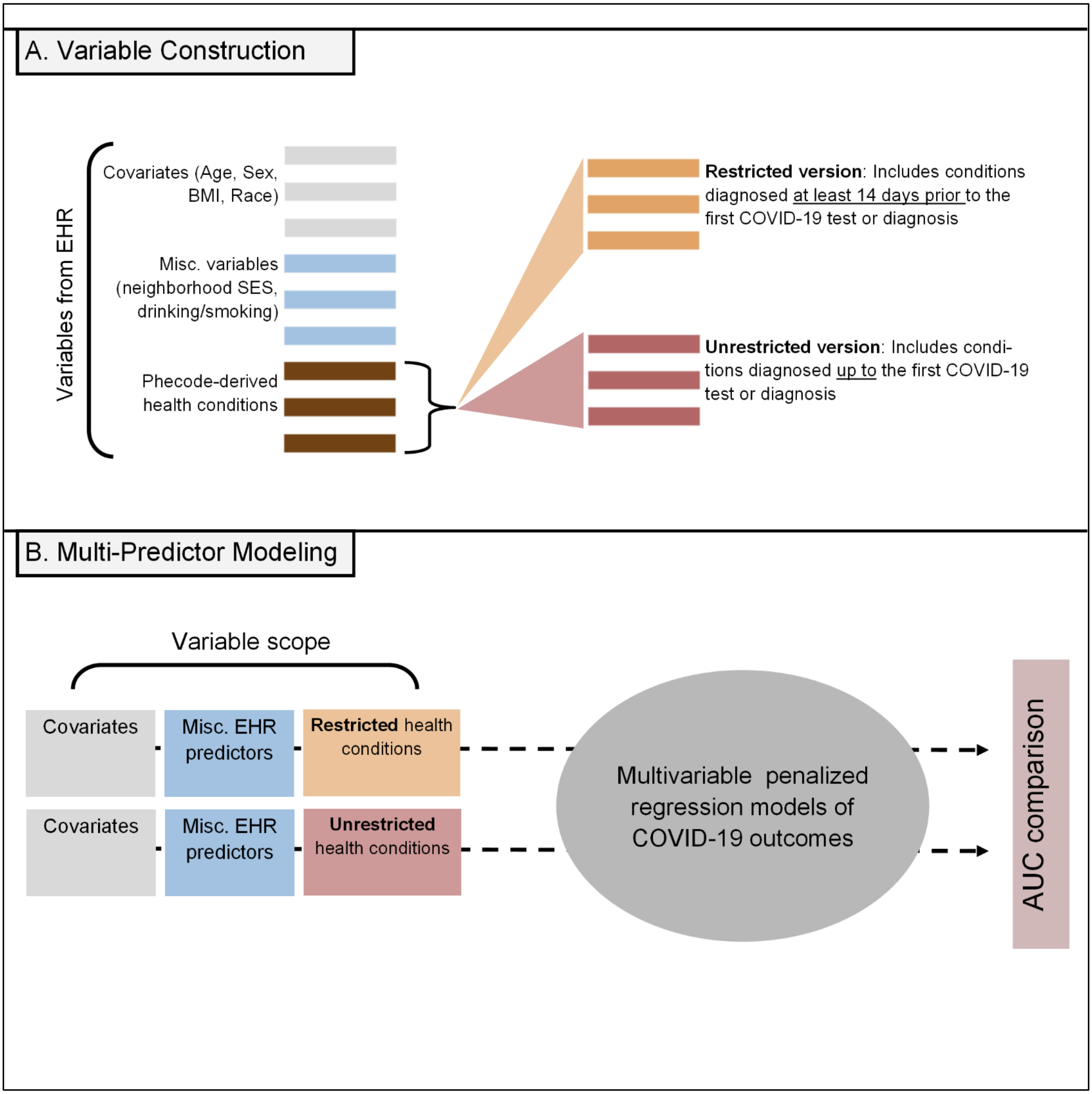

Results for the analysis are shown below in Table 1.

Table 1. Area under the Curve (AUC) for Michigan Medicine Case-Control Analysis of COVID-19 Outcomes

Mean AUC (95% Empirical CI)				
Outcome Variable	Model Type	Covariates Only	Covariates + EHR Variables (Restricted)	Covariates + EHR Variables (Unrestricted)
Tested for COVID-19	Lasso	0.563 (0.551, 0.574)	0.723 (0.712, 0.732)	0.747 (0.737, 0.756)
	Ridge Regression	0.563 (0.551, 0.574)	0.724 (0.713, 0.734)	0.748 (0.737, 0.757)
	Elastic Net	0.563 (0.551, 0.574)	0.724 (0.712, 0.734)	0.748 (0.737, 0.757)
Diagnosed with COVID-19	Lasso	0.671 (0.643, 0.698)	0.759 (0.735, 0.785)	0.79 (0.77, 0.812)
	Ridge Regression	0.671 (0.643, 0.698)	0.774 (0.749, 0.803)	0.803 (0.785, 0.823)
	Elastic net	0.671 (0.643, 0.698)	0.773 (0.748, 0.801)	0.802 (0.784, 0.823)

Mean AUC reflects the average of 100 random training test/splits, with a CI representing the 2.5th and 97.5th percentiles, respectively. Data from Michigan Medicine COVID-19 Cohort. Corresponding models include only EHR-derived predictors and covariates. Sample size: n = 19,682 for testing models, n = 11,261 for diagnosis models.

In the following tables and figures, we provide the selected tuning parameters for the models and plots describing their calibration. For tuning parameters, we present the λ and α selected for the first ten data splits of the procedure. Note that $\lambda_1 = \alpha \times \lambda$ and $\lambda_2 = (1 - \alpha)/2 \times \lambda$. For calibration, the first set of plots show the distribution of Hosmer-Lemeshow test p-values for all 100 training-test splits. A p-value less than 0.05 suggests poor calibration. The second set of plots using only the first training-test split and show the predicted probabilities of each outcome contrasted with the observed proportion of the outcome in a particular prediction range. Results for calibration appeared to vary. For the *Received a COVID-19* outcome, the models with only covariates had poor calibration, but the integration of other predictors, especially with elastic net and ridge regression, led to improvements. Similar trends were observed for the *Diagnosed with COVID-19* outcome, though calibration as a whole seemed to be superior.

S14 File (continued)

Elastic Net Regression Model Penalties – Outcome: Received a COVID-19 Test				
	Covariates + EHR Variables (Restricted)		Covariates + EHR Variables (Unrestricted)	
Split	Lambda	Alpha	Lambda	Alpha
1	0.005	0.100	0.005	0.100
2	0.030	0.100	0.03	0.100
3	0.005	0.100	0.005	0.100
4	0.005	0.100	0.005	0.100
5	0.005	0.100	0.005	0.100
6	0.005	0.500	0.005	0.300
7	0.005	0.100	0.005	0.100
8	0.005	0.100	0.005	0.100
9	0.005	0.100	0.005	0.100
10	0.005	0.100	0.005	0.100
**Elastic Net Regression Model Penalties – Outcome: Diagnosed with COVID-19**				
	Covariates + EHR Variables (Restricted)		Covariates + EHR Variables (Unrestricted)	
Split	Lambda	Alpha	Lambda	Alpha
1	0.005	0.100	0.005	0.100
2	0.005	0.100	0.005	0.100
3	0.005	0.100	0.005	0.100
4	0.005	0.100	0.005	0.100
5	0.005	0.100	0.005	0.100
6	0.005	0.100	0.005	0.100
7	0.005	0.100	0.005	0.100
8	0.005	0.100	0.005	0.100
9	0.005	0.100	0.005	0.100
10	0.005	0.100	0.005	0.100

Lambda and alpha were selected by five-fold cross-validation on the training set of a single 70/30 train/test split.

**Ridge Regression Model Penalties – Outcome: Received a COVID-19 Test**				
	Covariates + EHR Variables (Restricted)		Covariates + EHR Variables (Unrestricted)	
Split	Lambda	Alpha	Lambda	Alpha
1	0.01	0.000	0.010	0.000
2	0.04	0.000	0.030	0.000
3	0.01	0.000	0.010	0.000
4	0.01	0.000	0.010	0.000
5	0.01	0.000	0.010	0.000
6	0.015	0.000	0.015	0.000
7	0.01	0.000	0.010	0.000
8	0.01	0.000	0.010	0.000
9	0.01	0.000	0.010	0.000
10	0.01	0.000	0.010	0.000
**Ridge Regression Model Penalties – Outcome: Diagnosed with COVID-19**				
	Covariates + EHR Variables (Restricted)		Covariates + EHR Variables (Unrestricted)	
Split	Lambda	Alpha	Lambda	Alpha
1	0.005	0.000	0.005	0.000
2	0.005	0.000	0.005	0.000
3	0.005	0.000	0.005	0.000
4	0.005	0.000	0.005	0.000
5	0.005	0.000	0.005	0.000
6	0.005	0.000	0.005	0.000
7	0.005	0.000	0.005	0.000
8	0.005	0.000	0.005	0.000
9	0.005	0.000	0.005	0.000
10	0.005	0.000	0.005	0.000

Lambda and alpha were selected by five-fold cross-validation on the training set of a single 70/30 train/test split.

**Lasso Regression Model Penalties – Outcome: Received a COVID-19 Test**				
	Covariates + EHR Variables (Restricted)		Covariates + EHR Variables (Unrestricted)	
Split	Lambda	Alpha	Lambda	Alpha
1	0.005	1.000	0.005	1.000
2	0.005	1.000	0.005	1.000
3	0.005	1.000	0.005	1.000
4	0.005	1.000	0.005	1.000
5	0.005	1.000	0.005	1.000
6	0.005	1.000	0.005	1.000
7	0.005	1.000	0.005	1.000
8	0.005	1.000	0.005	1.000
9	0.005	1.000	0.005	1.000
10	0.005	1.000	0.005	1.000
**Lasso Regression Model Penalties – Outcome: Diagnosed with COVID-19**				
	Covariates + EHR Variables (Restricted)		Covariates + EHR Variables (Unrestricted)	
Split	Lambda	Alpha	Lambda	Alpha
1	0.005	1.000	0.005	1.000
2	0.005	1.000	0.005	1.000
3	0.005	1.000	0.005	1.000
4	0.005	1.000	0.005	1.000
5	0.005	1.000	0.005	1.000
6	0.005	1.000	0.005	1.000
7	0.005	1.000	0.005	1.000
8	0.005	1.000	0.005	1.000
9	0.005	1.000	0.005	1.000
10	0.005	1.000	0.005	1.000

Lambda and alpha were selected by five-fold cross-validation on the training set of a single 70/30 train/test split.

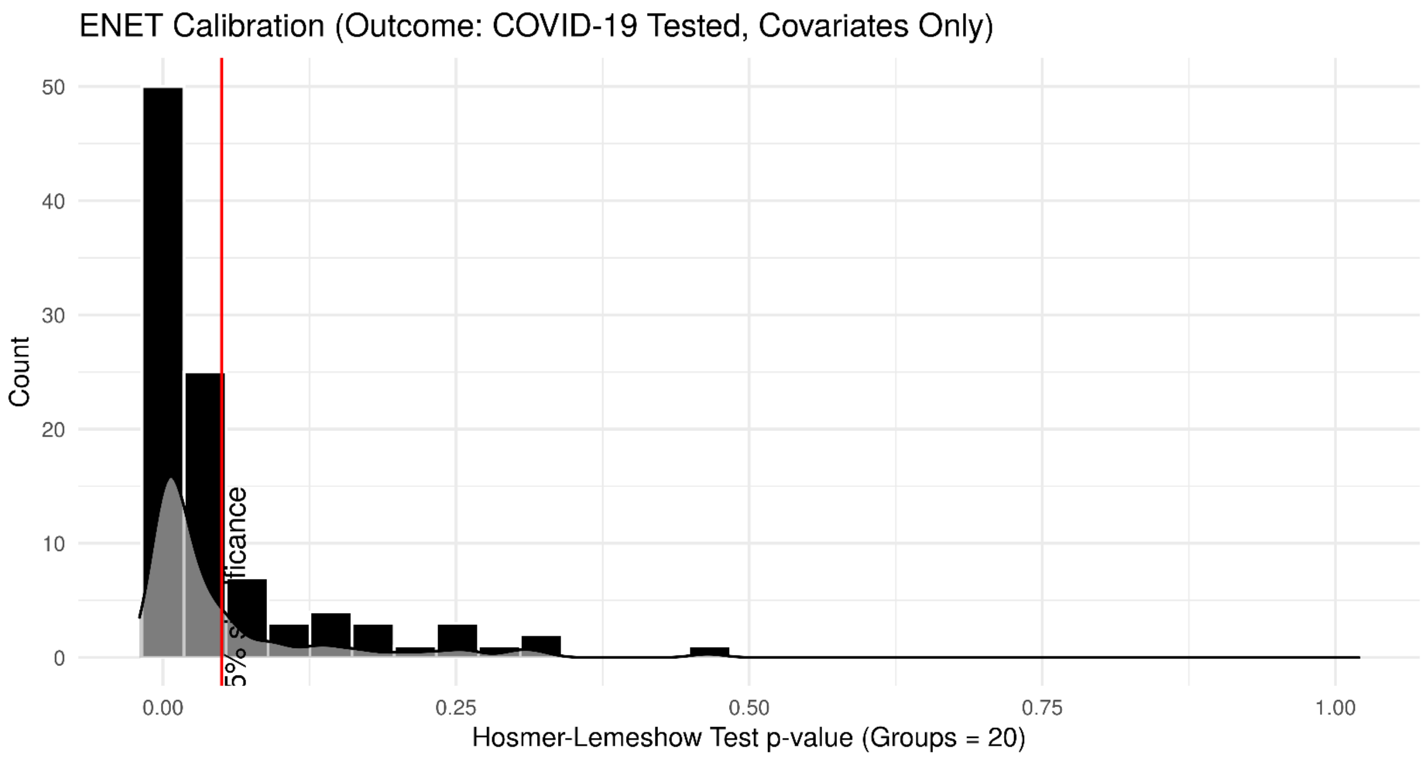

We plotted the p-values from conducting a Hosmer-Lemeshow goodness of fit test on all 100 train/test splits of the model evaluation procedure. Models tending to have poor calibration would show large numbers of p-values below the statistical significance threshold of 0.05.

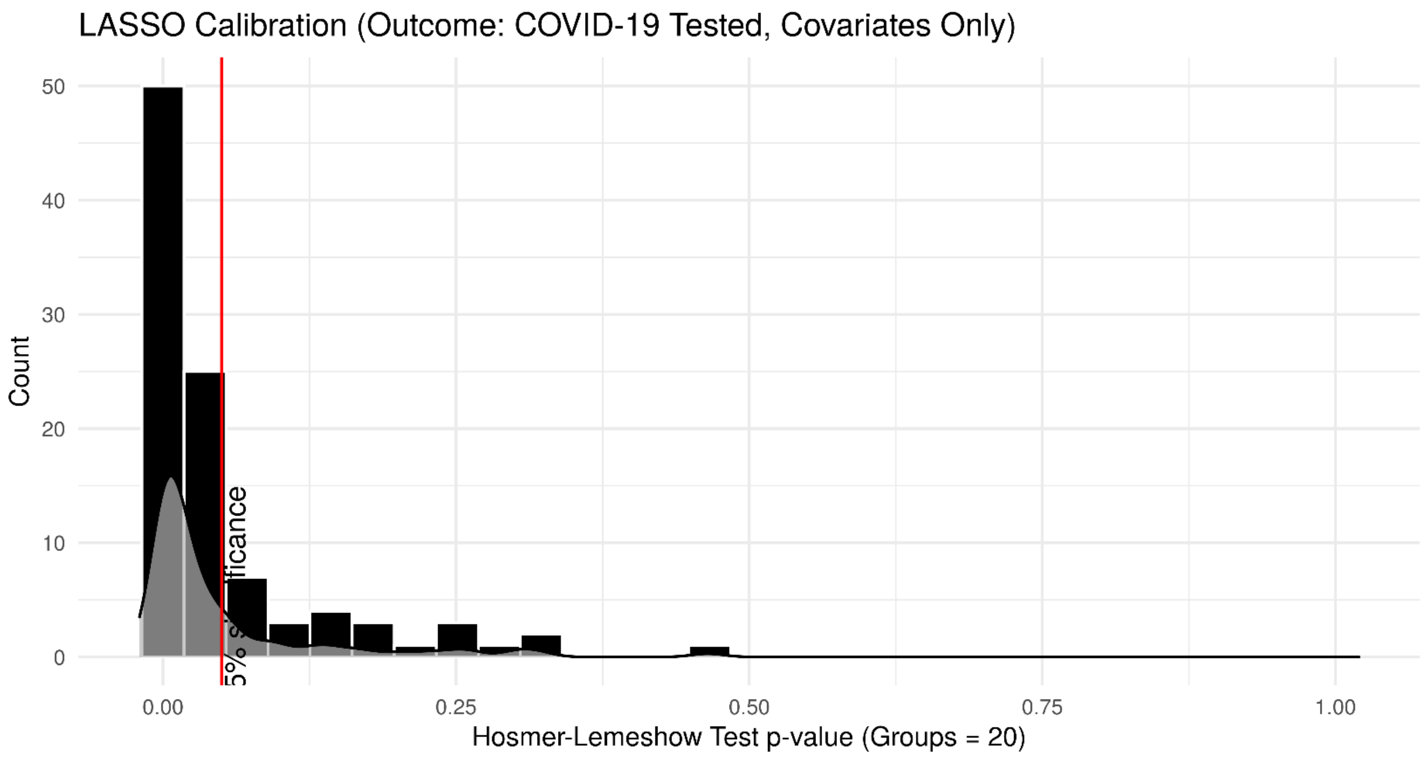

We plotted the p-values from conducting a Hosmer-Lemeshow goodness of fit test on all 100 train/test splits of the model evaluation procedure. Models tending to have poor calibration would show large numbers of p-values below the statistical significance threshold of 0.05.

S14 File (continued)

Ridge Calibration (Outcome: COVID-19 Tested, Covariates Only)

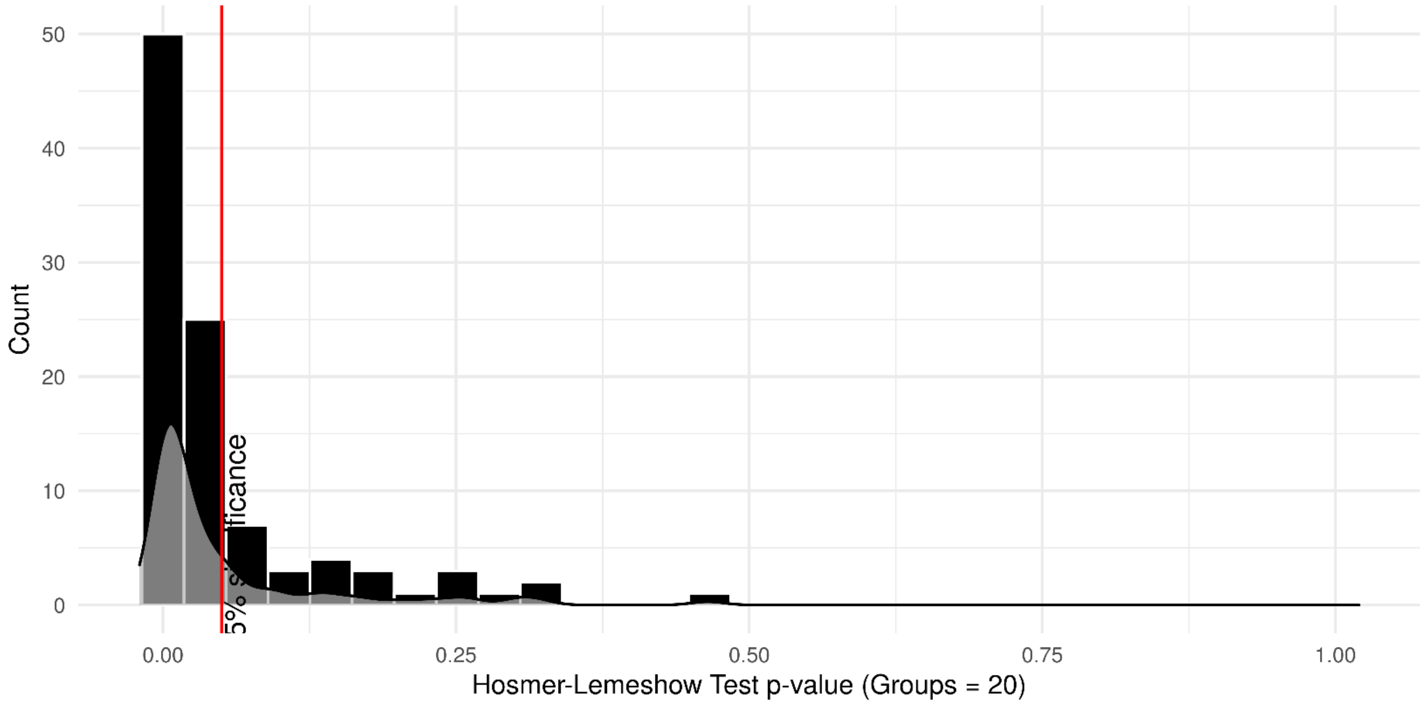

We plotted the p-values from conducting a Hosmer-Lemeshow goodness of fit test on all 100 train/test splits of the model evaluation procedure. Models tending to have poor calibration would show large numbers of p-values below the statistical significance threshold of 0.05.

ENET Calibration (Outcome: COVID-19 Tested, Restricted Comorbidities)

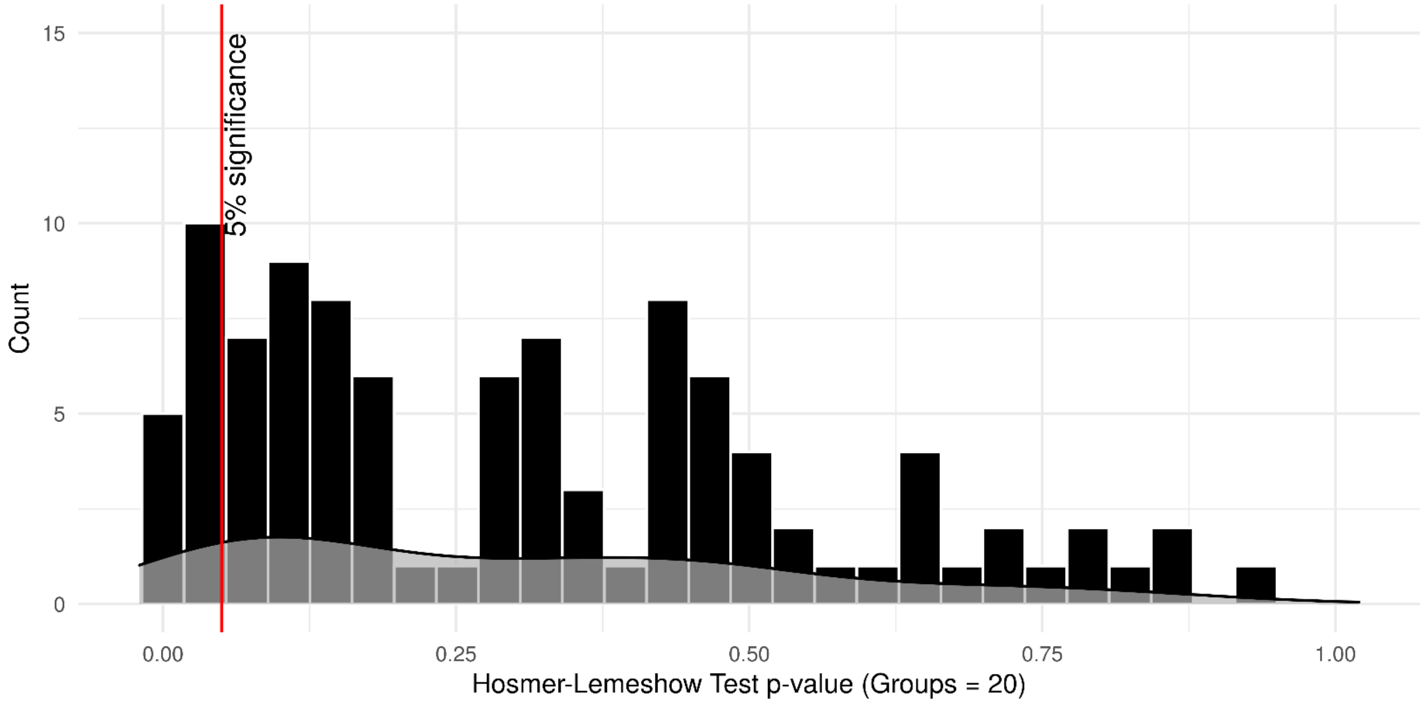

We plotted the p-values from conducting a Hosmer-Lemeshow goodness of fit test on all 100 train/test splits of the model evaluation procedure. Models tending to have poor calibration would show large numbers of p-values below the statistical significance threshold of 0.05.

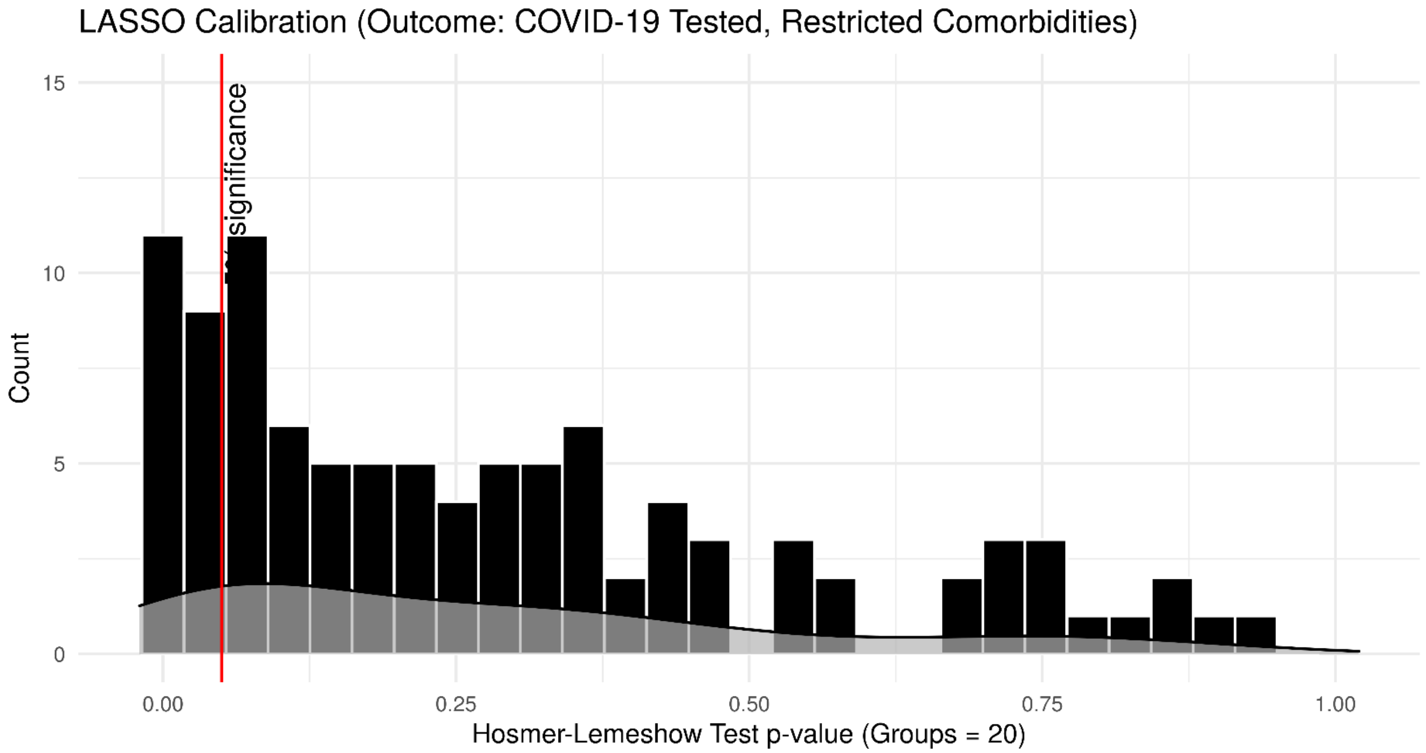

We plotted the p-values from conducting a Hosmer-Lemeshow goodness of fit test on all 100 train/test splits of the model evaluation procedure. Models tending to have poor calibration would show large numbers of p-values below the statistical significance threshold of 0.05.

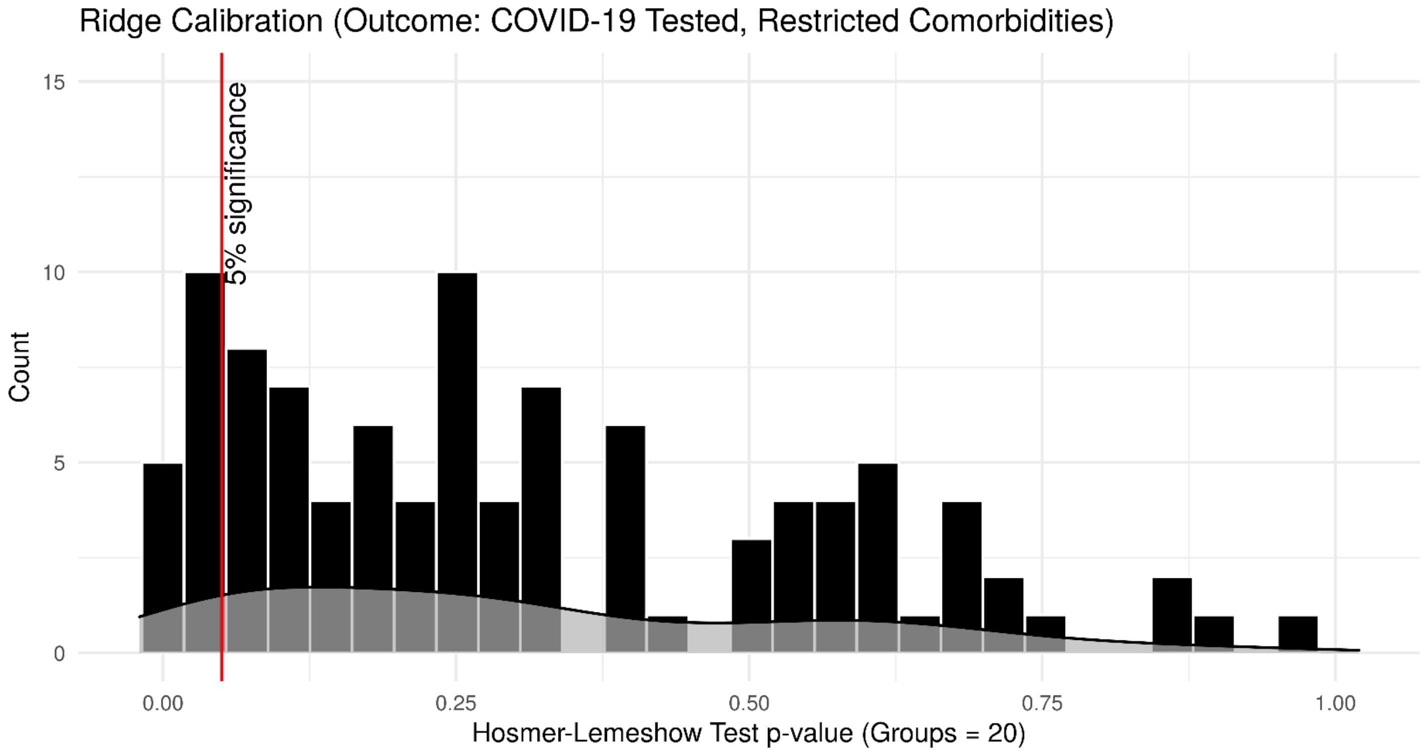

We plotted the p-values from conducting a Hosmer-Lemeshow goodness of fit test on all 100 train/test splits of the model evaluation procedure. Models tending to have poor calibration would show large numbers of p-values below the statistical significance threshold of 0.05.

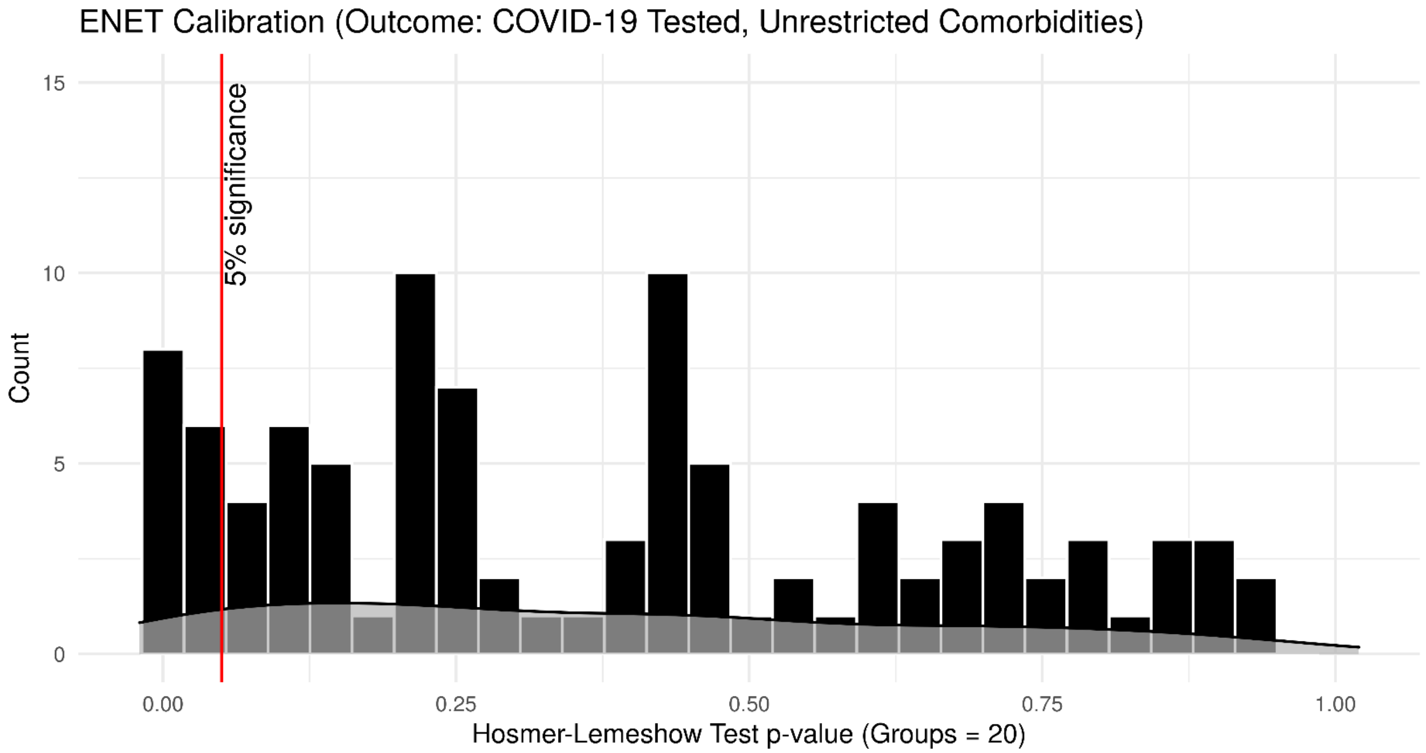

We plotted the p-values from conducting a Hosmer-Lemeshow goodness of fit test on all 100 train/test splits of the model evaluation procedure. Models tending to have poor calibration would show large numbers of p-values below the statistical significance threshold of 0.05.

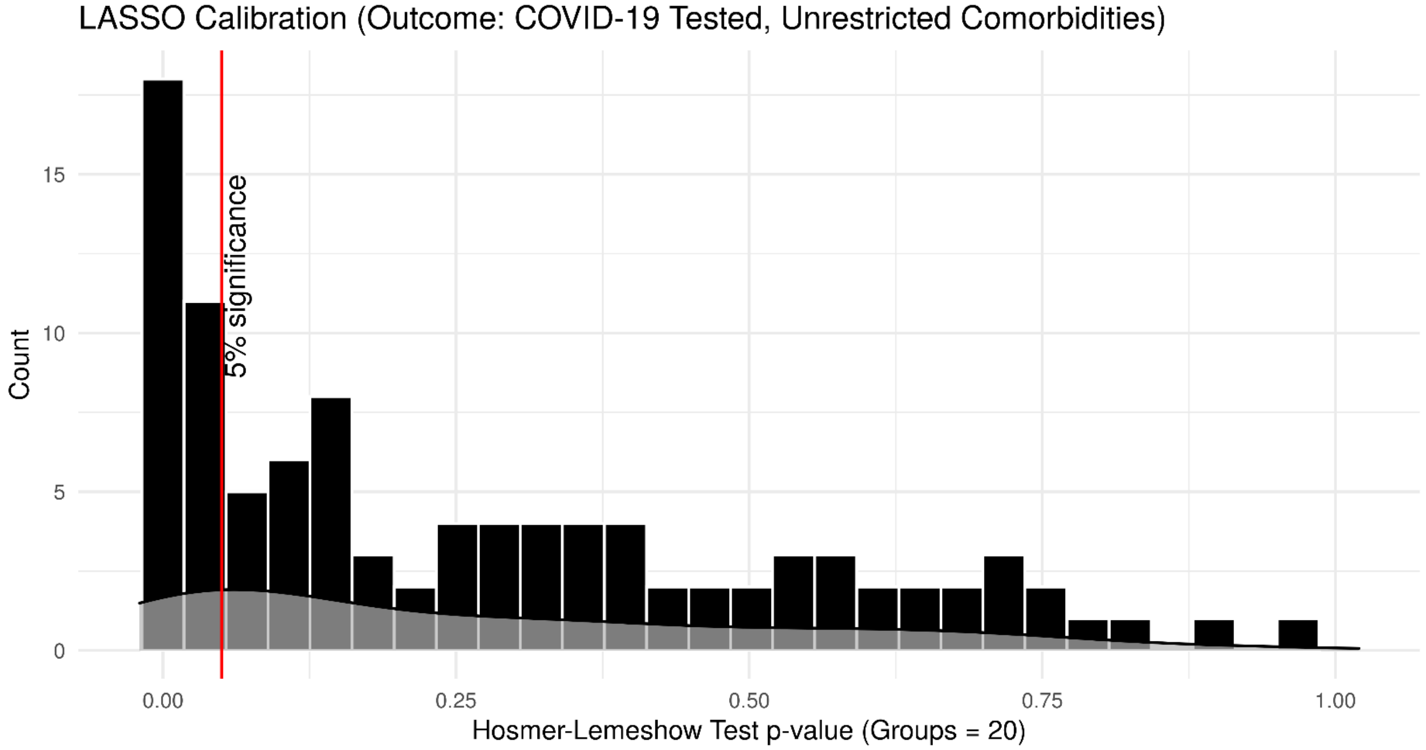

We plotted the p-values from conducting a Hosmer-Lemeshow goodness of fit test on all 100 train/test splits of the model evaluation procedure. Models tending to have poor calibration would show large numbers of p-values below the statistical significance threshold of 0.05.

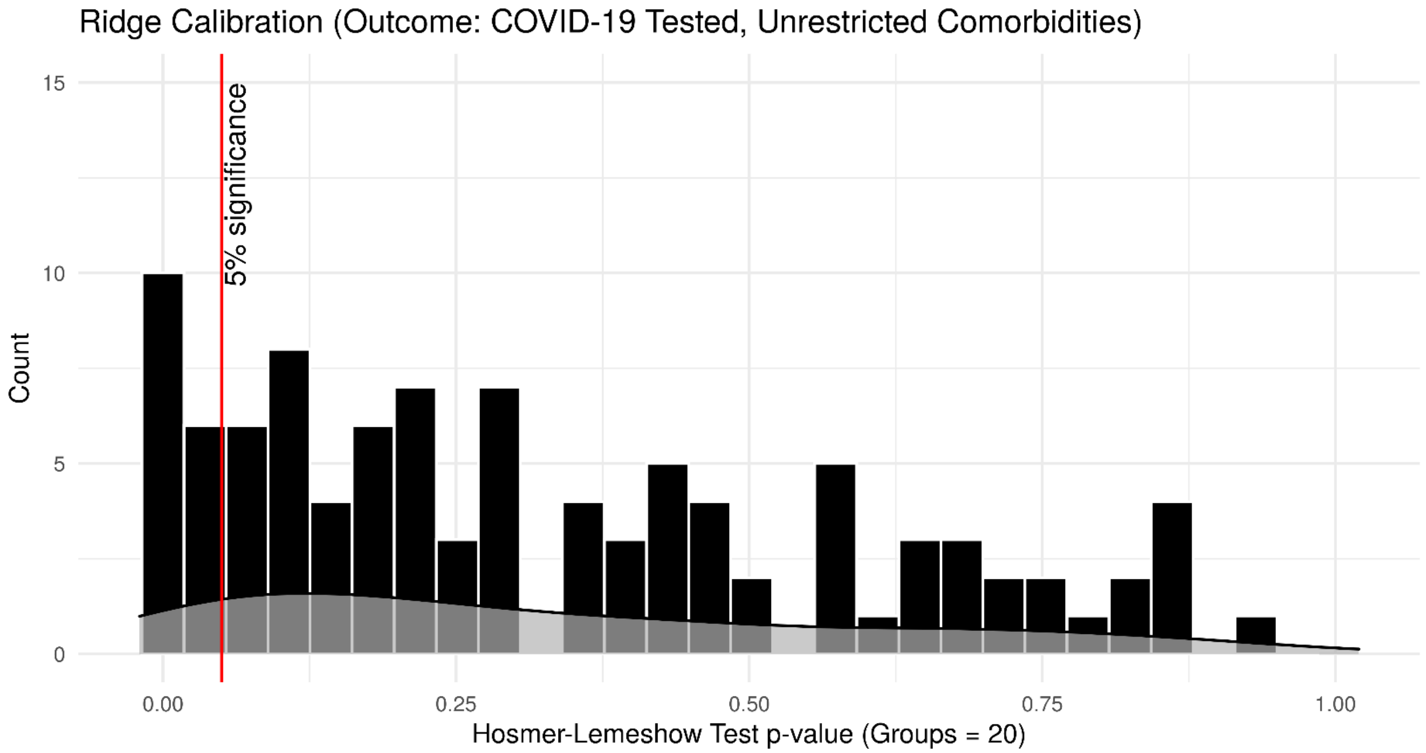

We plotted the p-values from conducting a Hosmer-Lemeshow goodness of fit test on all 100 train/test splits of the model evaluation procedure. Models tending to have poor calibration would show large numbers of p-values below the statistical significance threshold of 0.05.

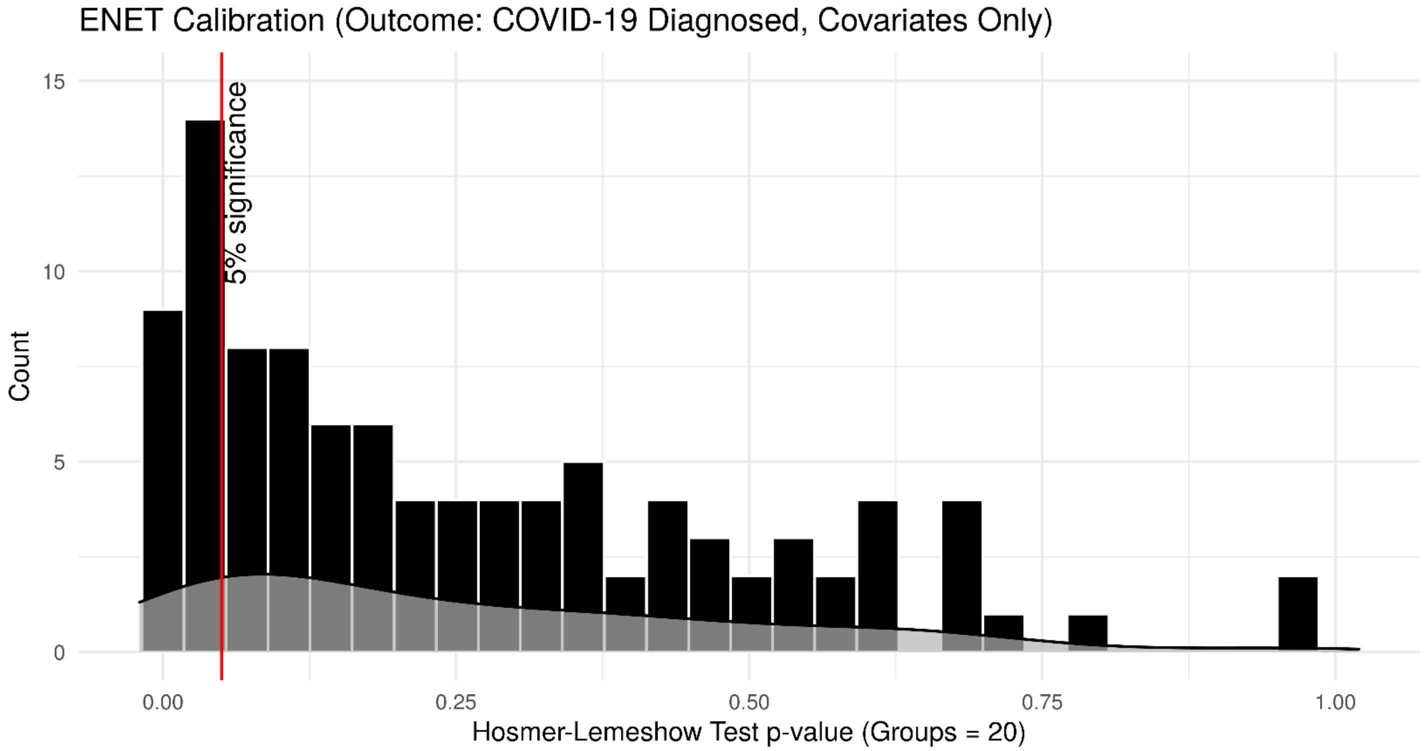

We plotted the p-values from conducting a Hosmer-Lemeshow goodness of fit test on all 100 train/test splits of the model evaluation procedure. Models tending to have poor calibration would show large numbers of p-values below the statistical significance threshold of 0.05.

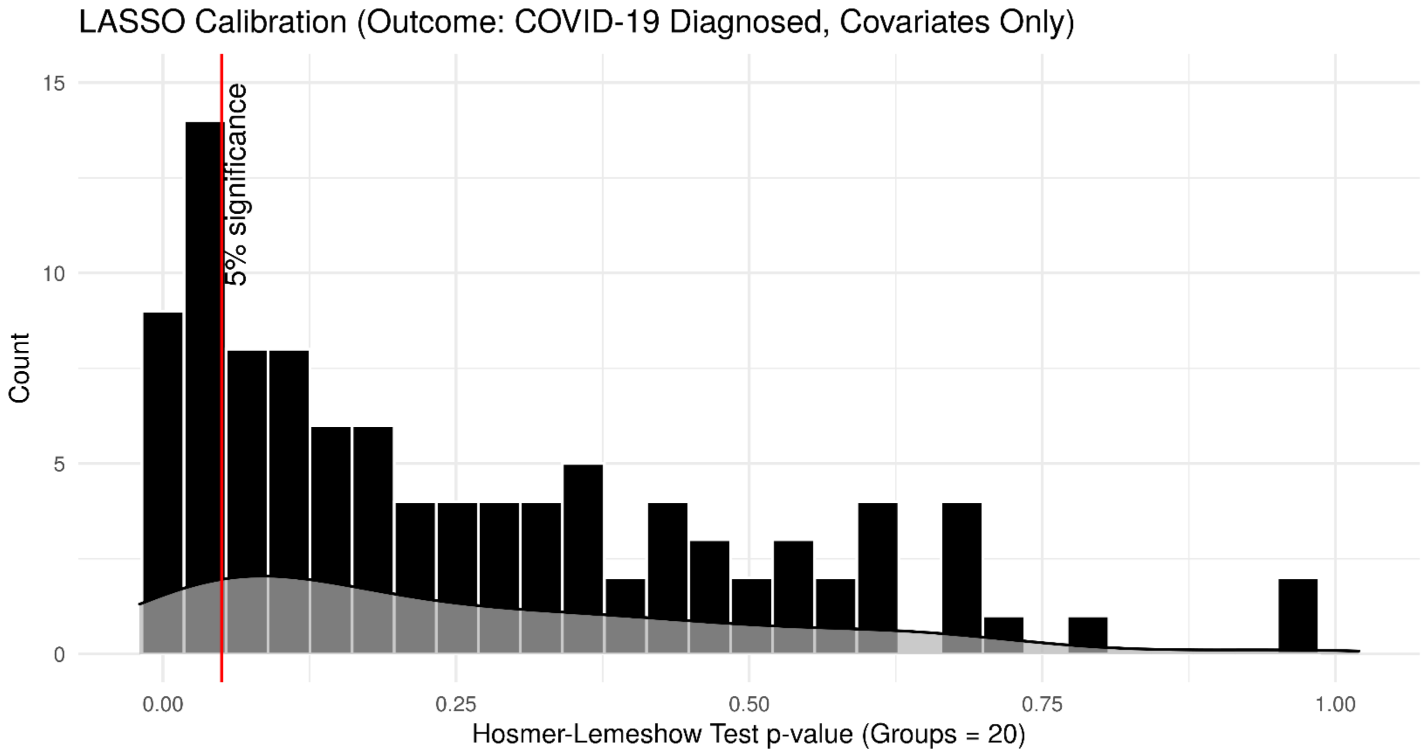

We plotted the p-values from conducting a Hosmer-Lemeshow goodness of fit test on all 100 train/test splits of the model evaluation procedure. Models tending to have poor calibration would show large numbers of p-values below the statistical significance threshold of 0.05.

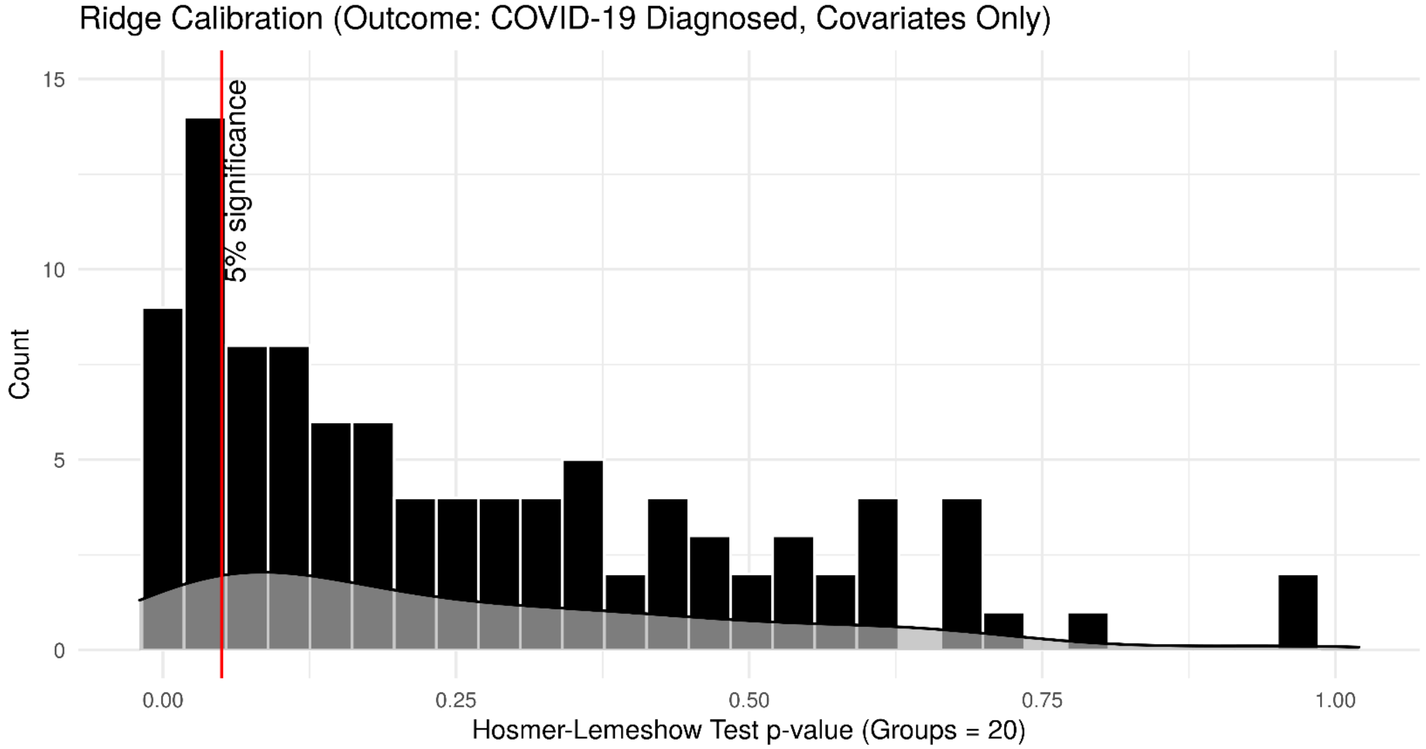

We plotted the p-values from conducting a Hosmer-Lemeshow goodness of fit test on all 100 train/test splits of the model evaluation procedure. Models tending to have poor calibration would show large numbers of p-values below the statistical significance threshold of 0.05.

ENET Calibration (Outcome: COVID-19 Diagnosed, Restricted Comorbidities)

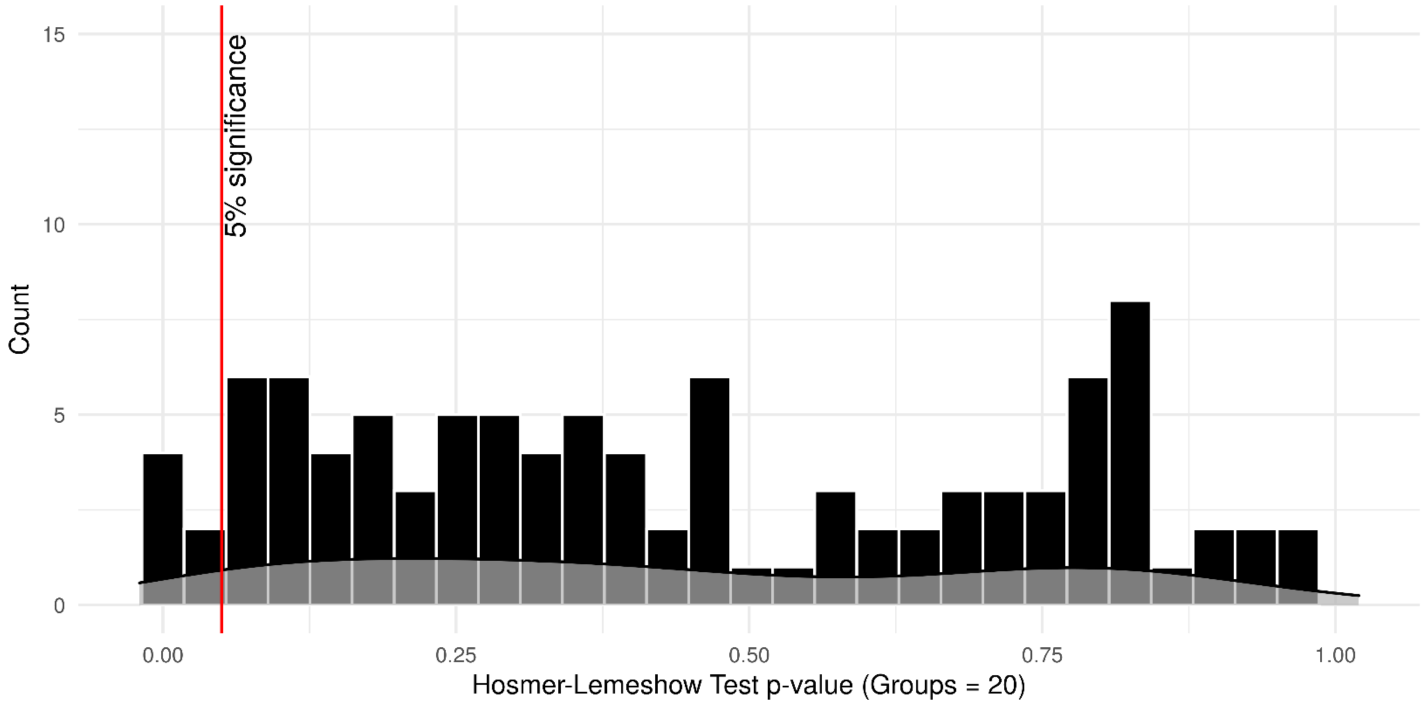

We plotted the p-values from conducting a Hosmer-Lemeshow goodness of fit test on all 100 train/test splits of the model evaluation procedure. Models tending to have poor calibration would show large numbers of p-values below the statistical significance threshold of 0.05.

LASSO Calibration (Outcome: COVID-19 Diagnosed, Restricted Comorbidities)

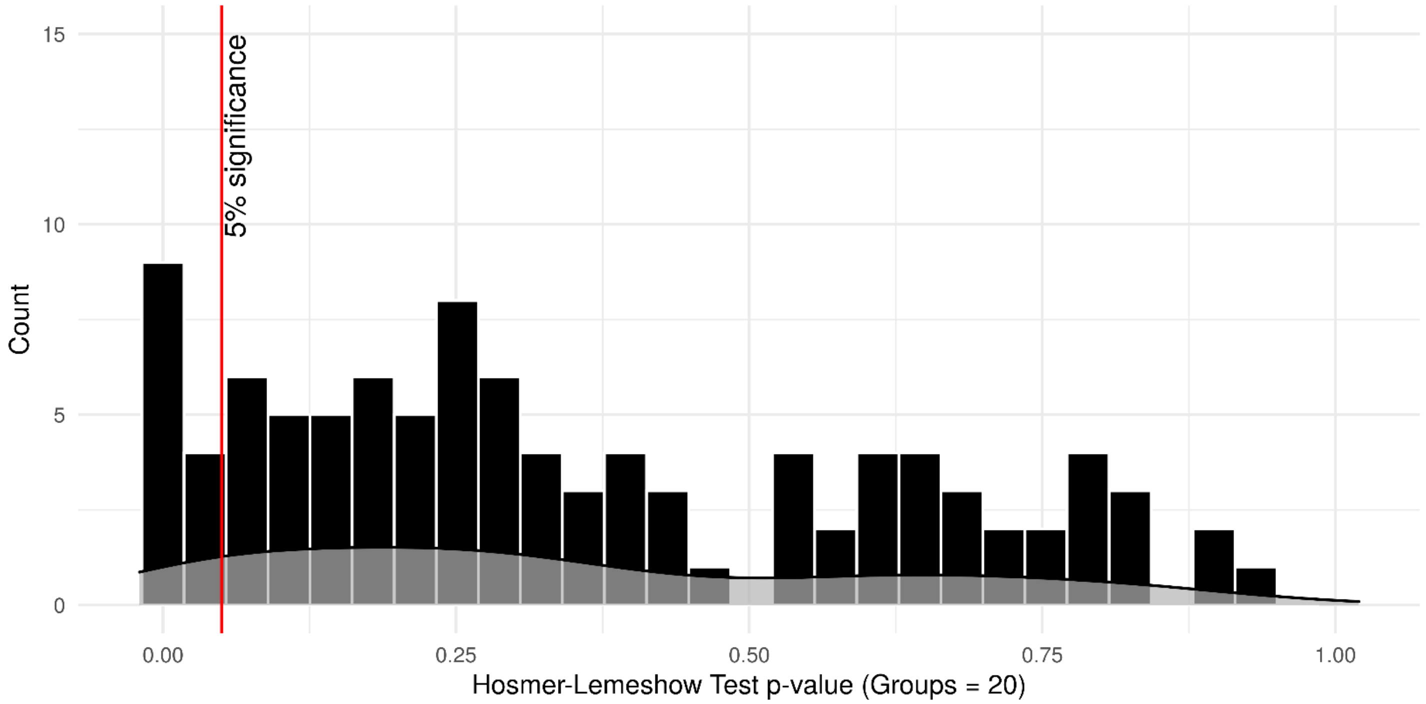

We plotted the p-values from conducting a Hosmer-Lemeshow goodness of fit test on all 100 train/test splits of the model evaluation procedure. Models tending to have poor calibration would show large numbers of p-values below the statistical significance threshold of 0.05.

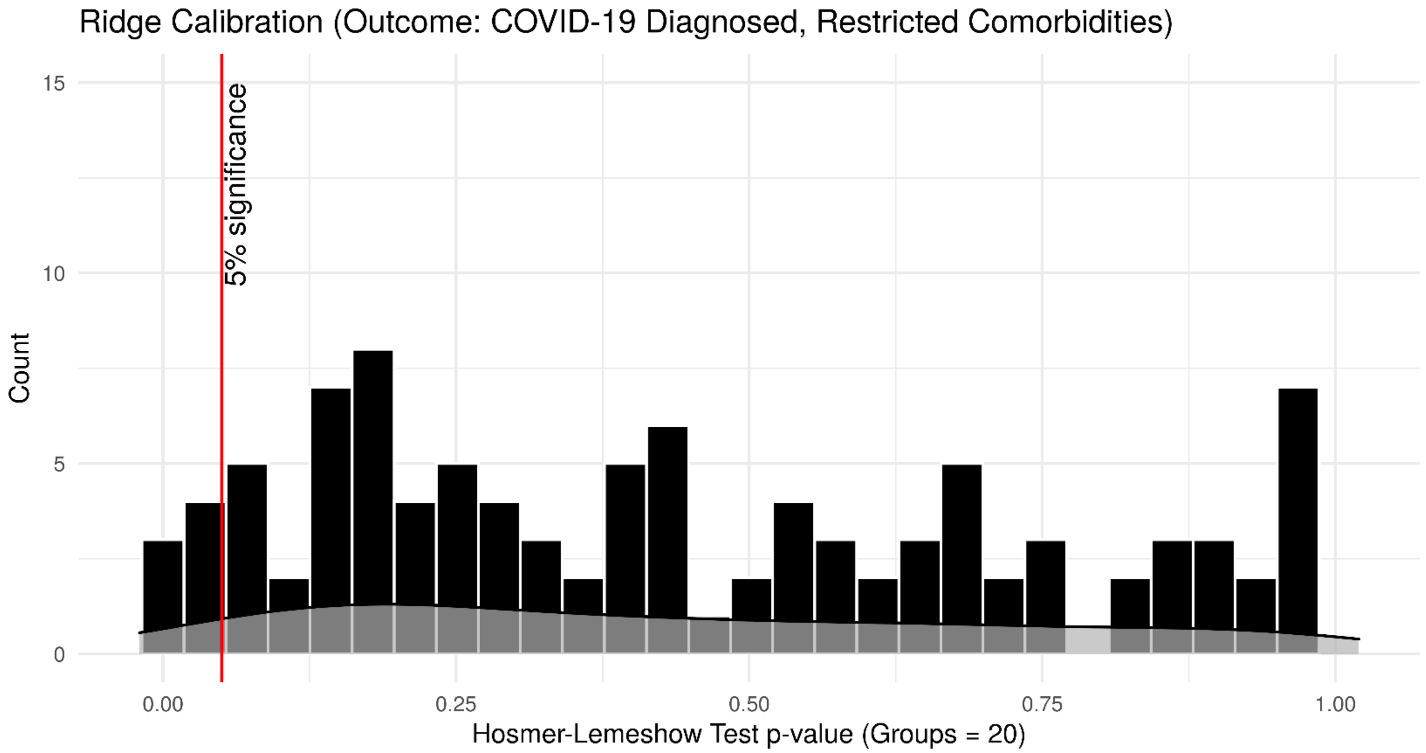

We plotted the p-values from conducting a Hosmer-Lemeshow goodness of fit test on all 100 train/test splits of the model evaluation procedure. Models tending to have poor calibration would show large numbers of p-values below the statistical significance threshold of 0.05.

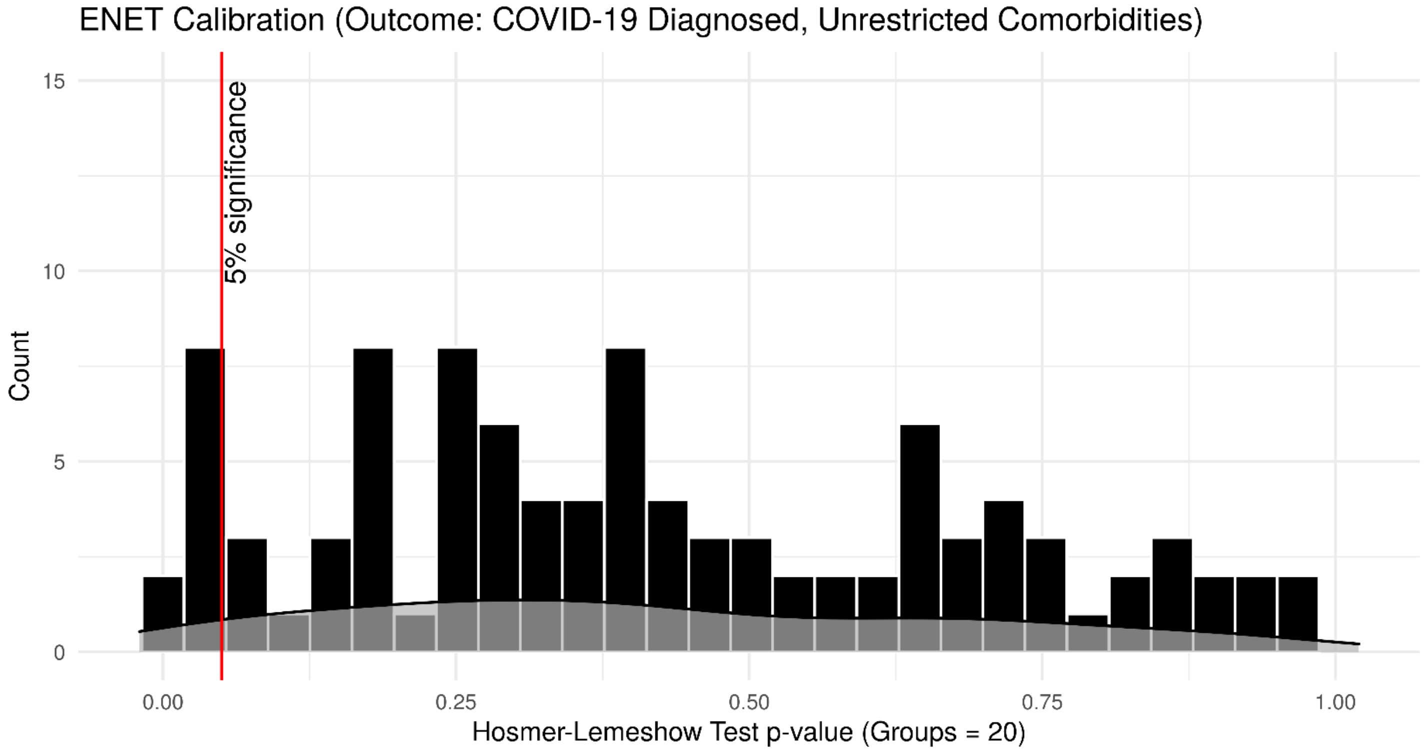

We plotted the p-values from conducting a Hosmer-Lemeshow goodness of fit test on all 100 train/test splits of the model evaluation procedure. Models tending to have poor calibration would show large numbers of p-values below the statistical significance threshold of 0.05.

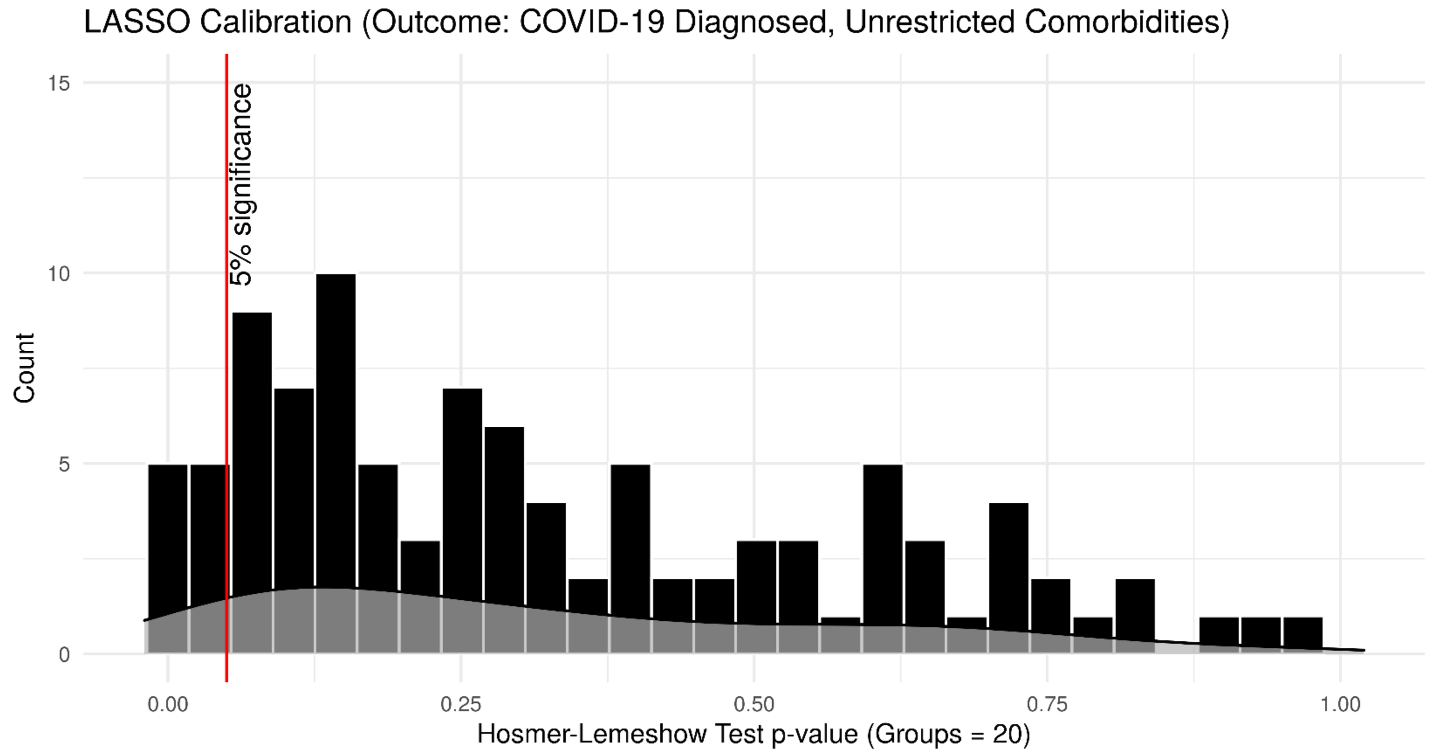

We plotted the p-values from conducting a Hosmer-Lemeshow goodness of fit test on all 100 train/test splits of the model evaluation procedure. Models tending to have poor calibration would show large numbers of p-values below the statistical significance threshold of 0.05.

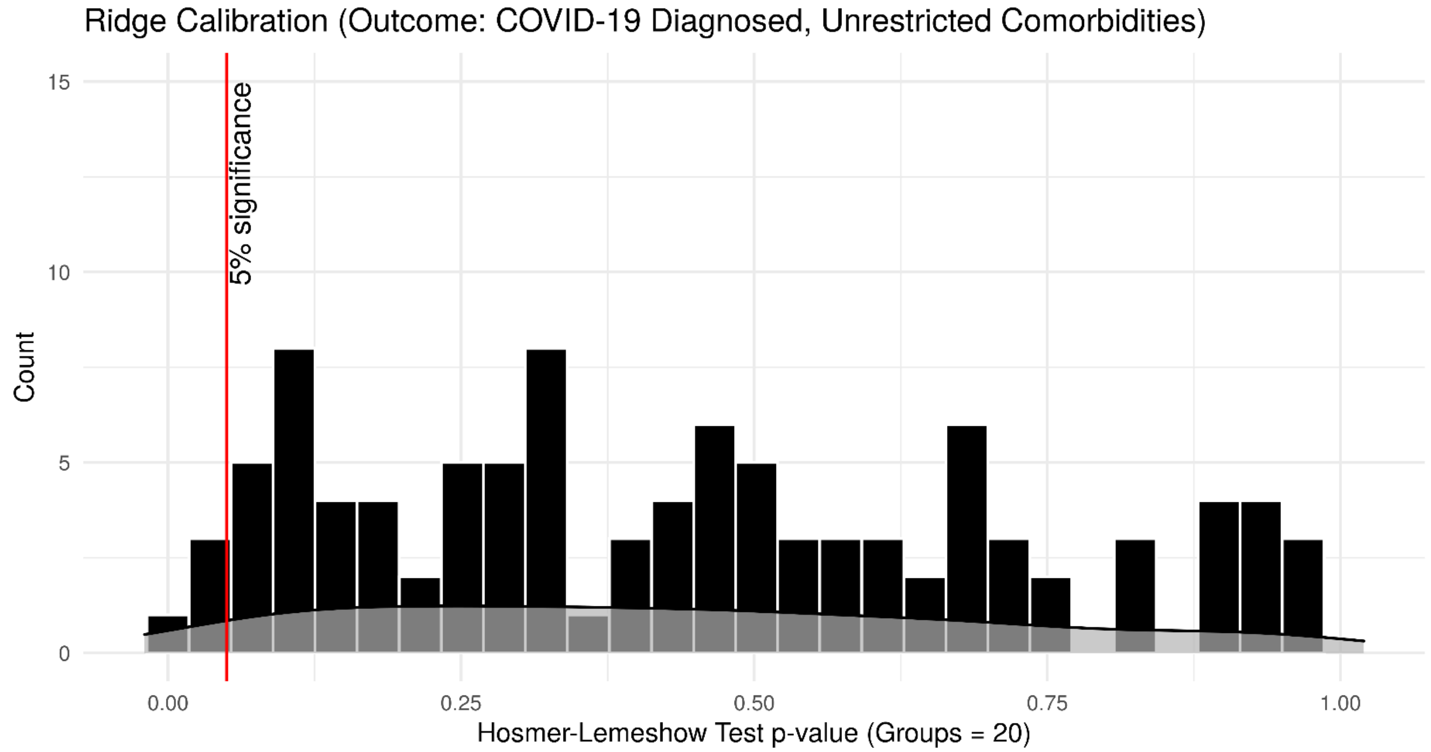

We plotted the p-values from conducting a Hosmer-Lemeshow goodness of fit test on all 100 train/test splits of the model evaluation procedure. Models tending to have poor calibration would show large numbers of p-values below the statistical significance threshold of 0.05.

S15 File. Calibration Plots for Survey-Based Models of COVID-19 Outcomes

Calibration

We assessed the calibration of our models with the construction of two plots. The first set of plots show the distribution of Hosmer-Lemeshow test p-values for all 100 training-test splits. A p-value less than 0.05 suggests poor calibration. The second set of plots using only the first training-test split and show the predicted probabilities of each outcome contrasted with the observed proportion of the outcome in a particular prediction range. Models for the outcome *Received a COVID-19 Test* in general appeared to be well-calibrated. The calibration plots from the first train/test split show that the observed risk for groups of respondents was highly similar to the predicted risk, and the Hosmer-Lemeshow p-values from all 100 splits were infrequently statistically significant. Calibration was weaker for the outcome *Diagnosed with COVID-19*, as the relationship between observed risk and predicted risk was less linear, and the distributions of Hosmer-Lemeshow test p-values tended to have spikes below 0.05. Models of the *Self-Diagnosed with COVID-19* outcome showed especially poor calibration per both sets of plots.

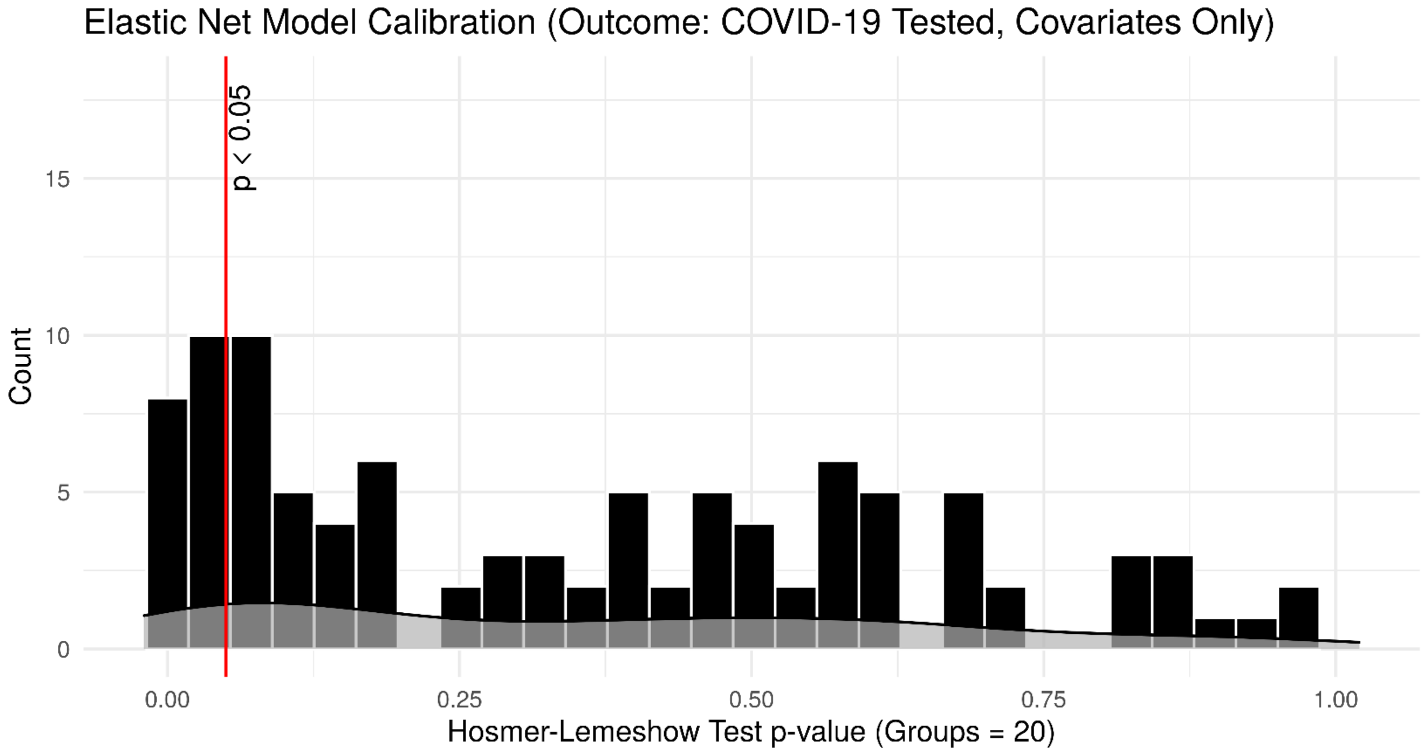

We plotted the p-values from conducting a Hosmer-Lemeshow goodness of fit test on all 100 train/test splits of the model evaluation procedure. Models tending to have poor calibration would show large numbers of p-values below the statistical

S15 File (continued)
significance threshold of 0.05.

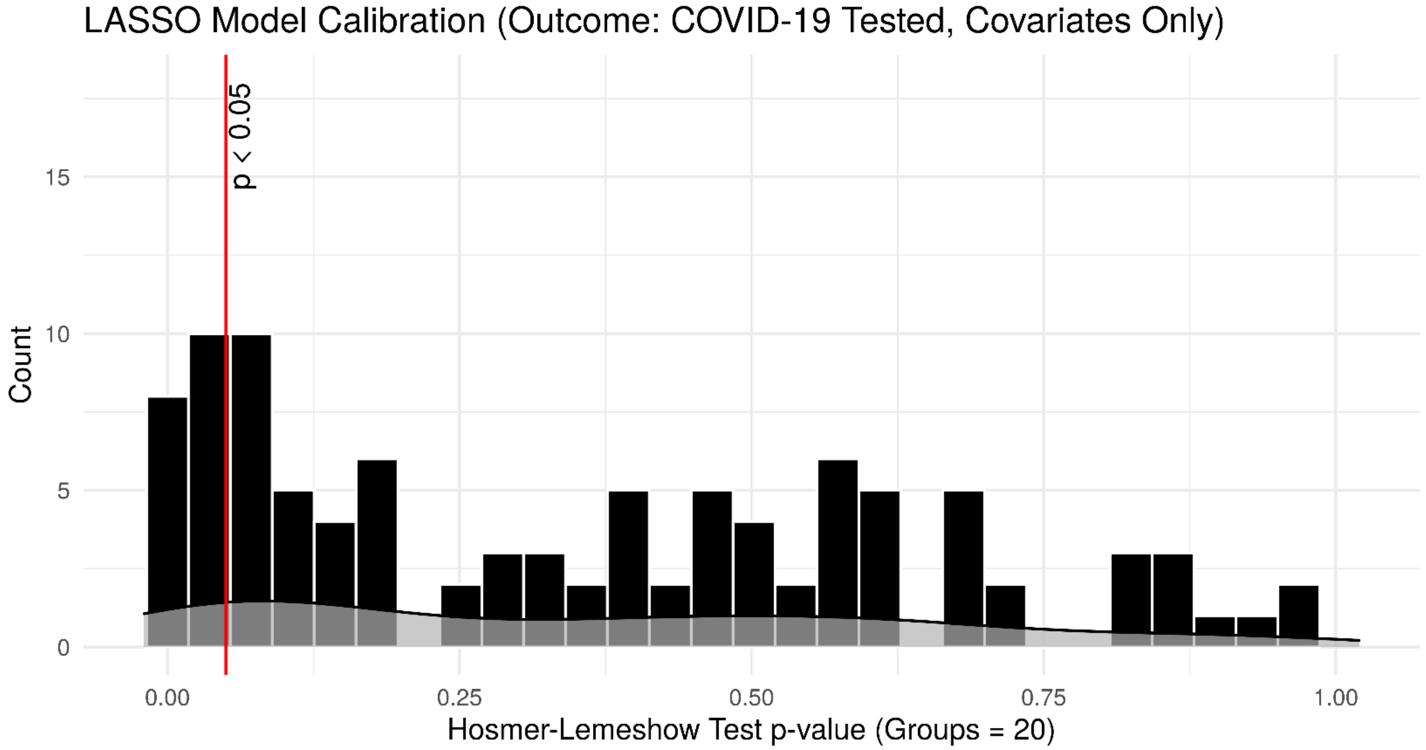

We plotted the p-values from conducting a Hosmer-Lemeshow goodness of fit test on all 100 train/test splits of the model evaluation procedure. Models tending to have poor calibration would show large numbers of p-values below the statistical significance threshold of 0.05.

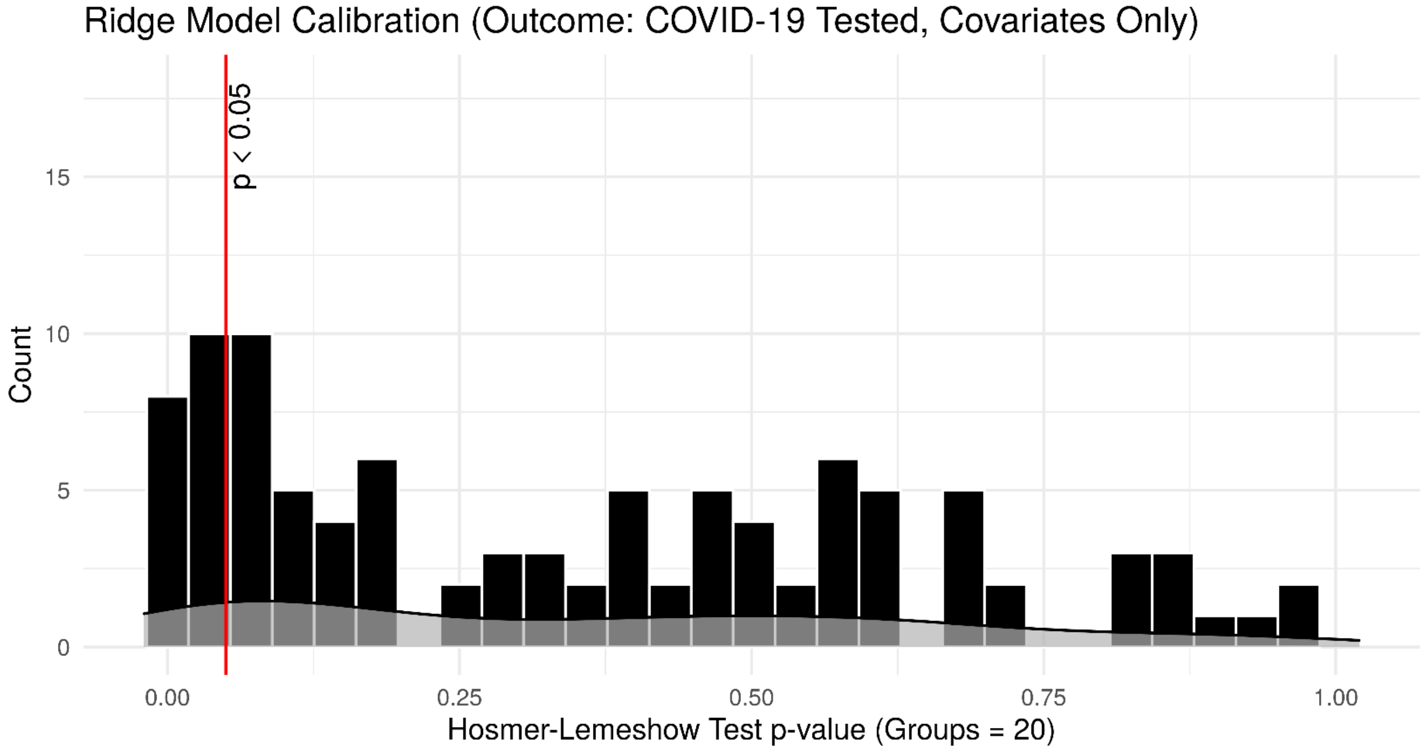

We plotted the p-values from conducting a Hosmer-Lemeshow goodness of fit test on all 100 train/test splits of the model evaluation procedure. Models tending to have poor calibration would show large numbers of p-values below the statistical significance threshold of 0.05.

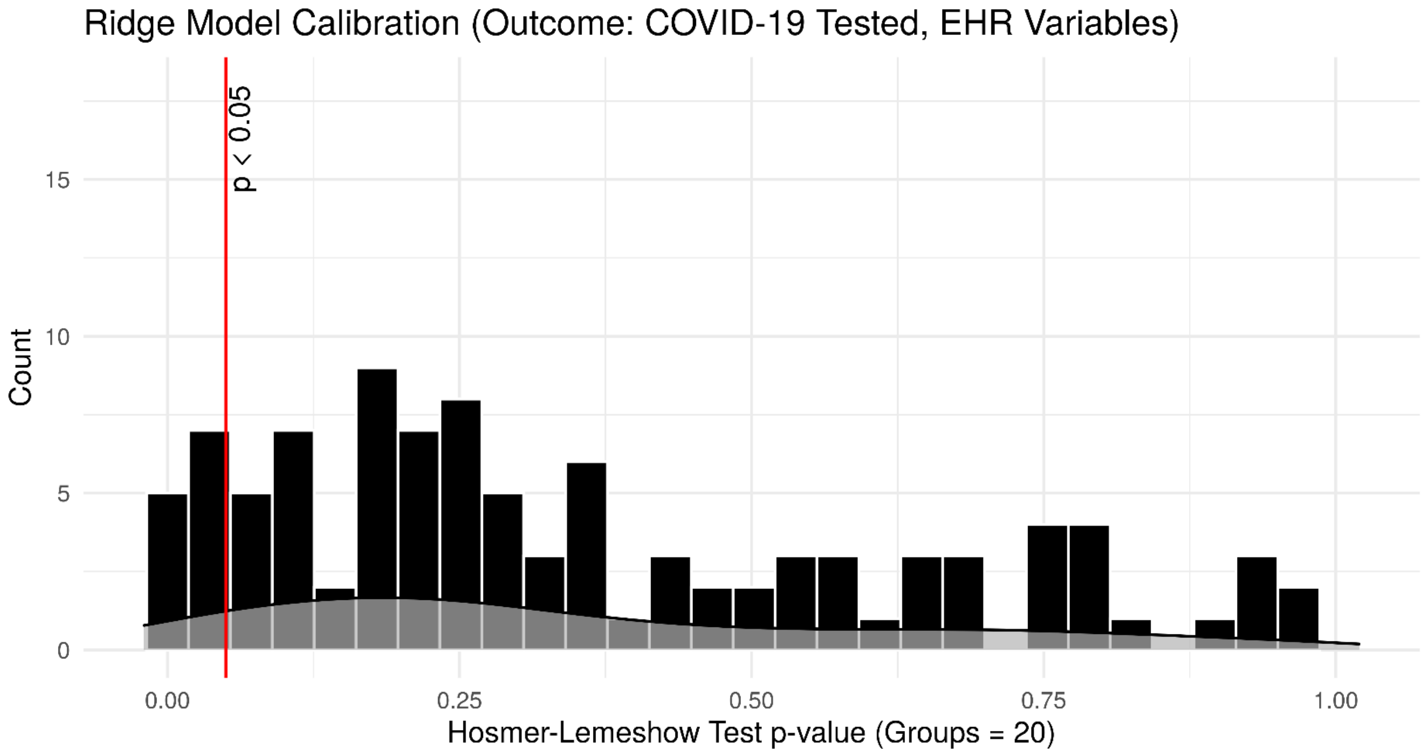

We plotted the p-values from conducting a Hosmer-Lemeshow goodness of fit test on all 100 train/test splits of the model evaluation procedure. Models tending to have poor calibration would show large numbers of p-values below the statistical significance threshold of 0.05.

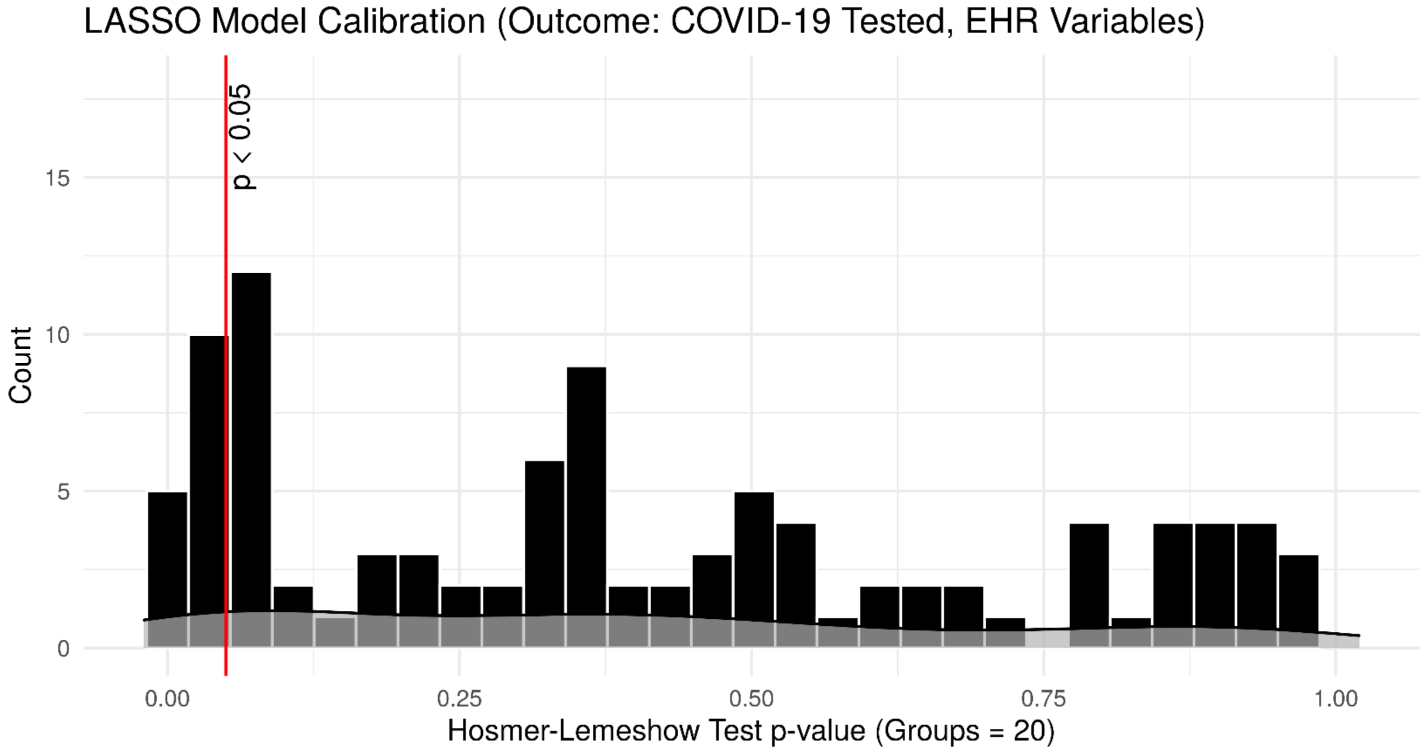

We plotted the p-values from conducting a Hosmer-Lemeshow goodness of fit test on all 100 train/test splits of the model evaluation procedure. Models tending to have poor calibration would show large numbers of p-values below the statistical significance threshold of 0.05.

S15 File (Continued)

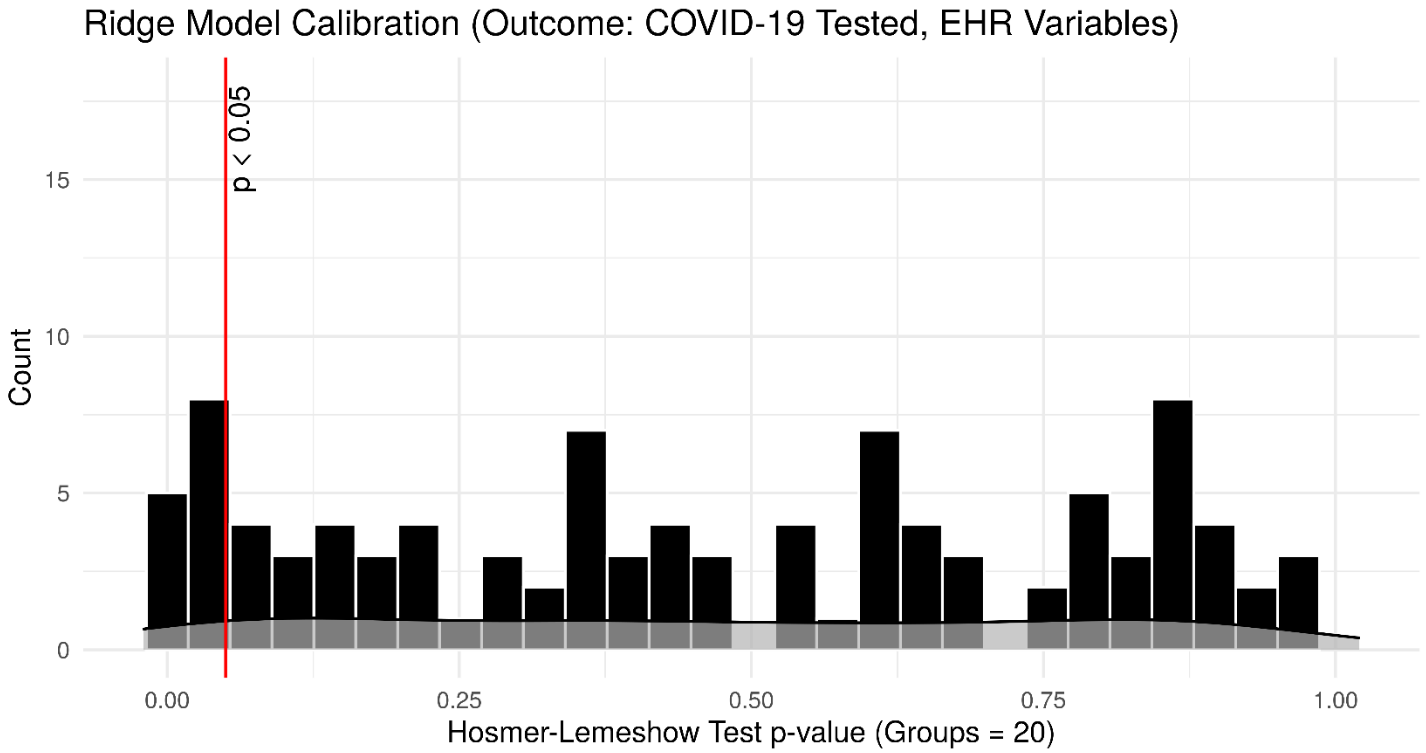

We plotted the p-values from conducting a Hosmer-Lemeshow goodness of fit test on all 100 train/test splits of the model evaluation procedure. Models tending to have poor calibration would show large numbers of p-values below the statistical significance threshold of 0.05.

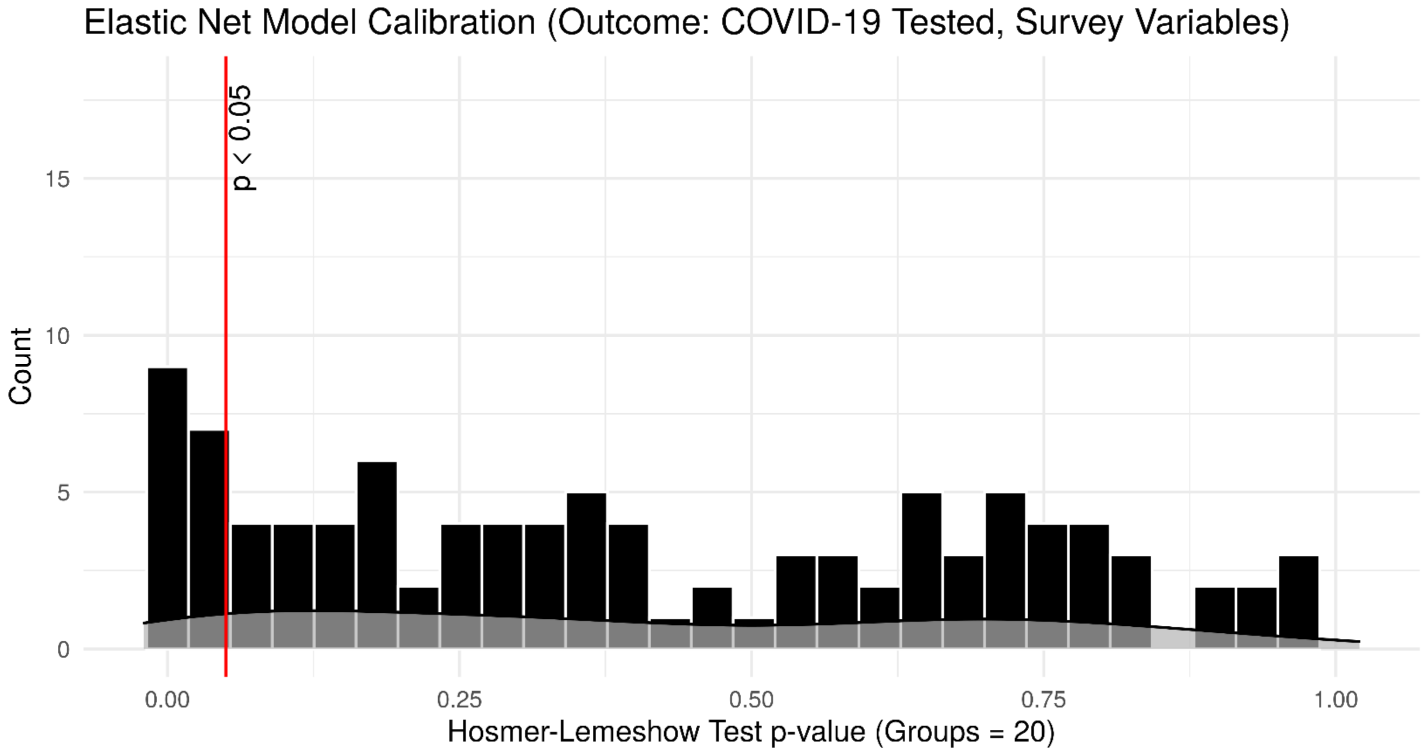

We plotted the p-values from conducting a Hosmer-Lemeshow goodness of fit test on all 100 train/test splits of the model evaluation procedure. Models tending to have poor calibration would show large numbers of p-values below the statistical significance threshold of 0.05.

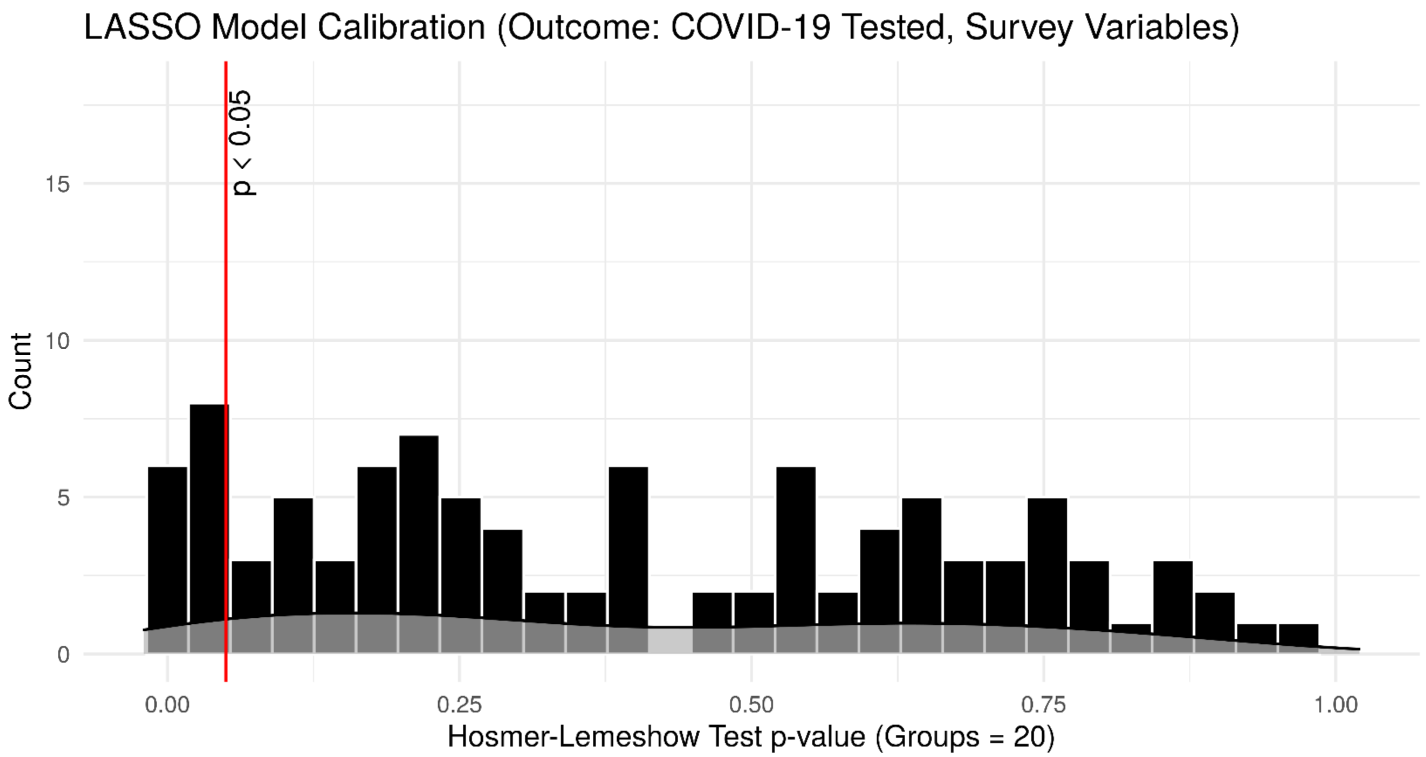

We plotted the p-values from conducting a Hosmer-Lemeshow goodness of fit test on all 100 train/test splits of the model evaluation procedure. Models tending to have poor calibration would show large numbers of p-values below the statistical significance threshold of 0.05.

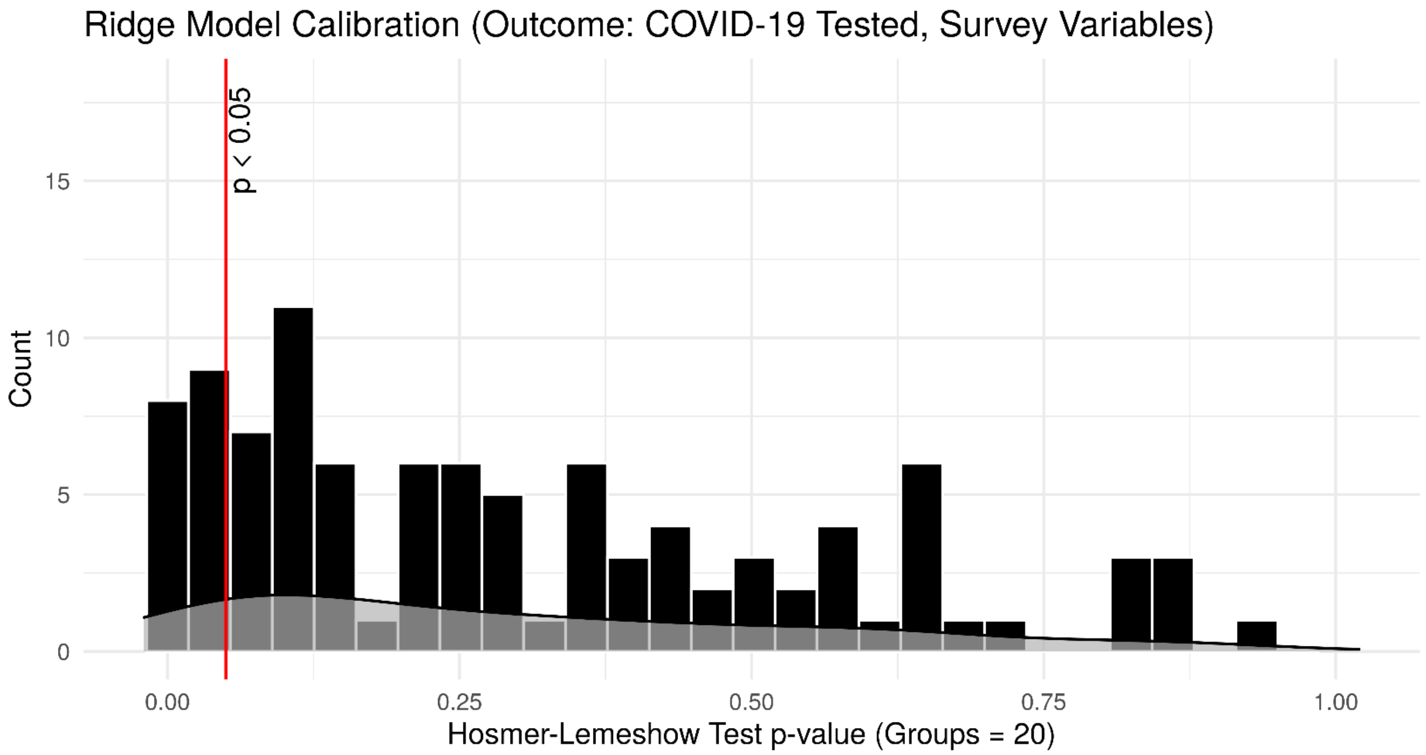

We plotted the p-values from conducting a Hosmer-Lemeshow goodness of fit test on all 100 train/test splits of the model evaluation procedure. Models tending to have poor calibration would show large numbers of p-values below the statistical significance threshold of 0.05.

S14 File. Description and Results of EHR-Based Case-Control Analysis

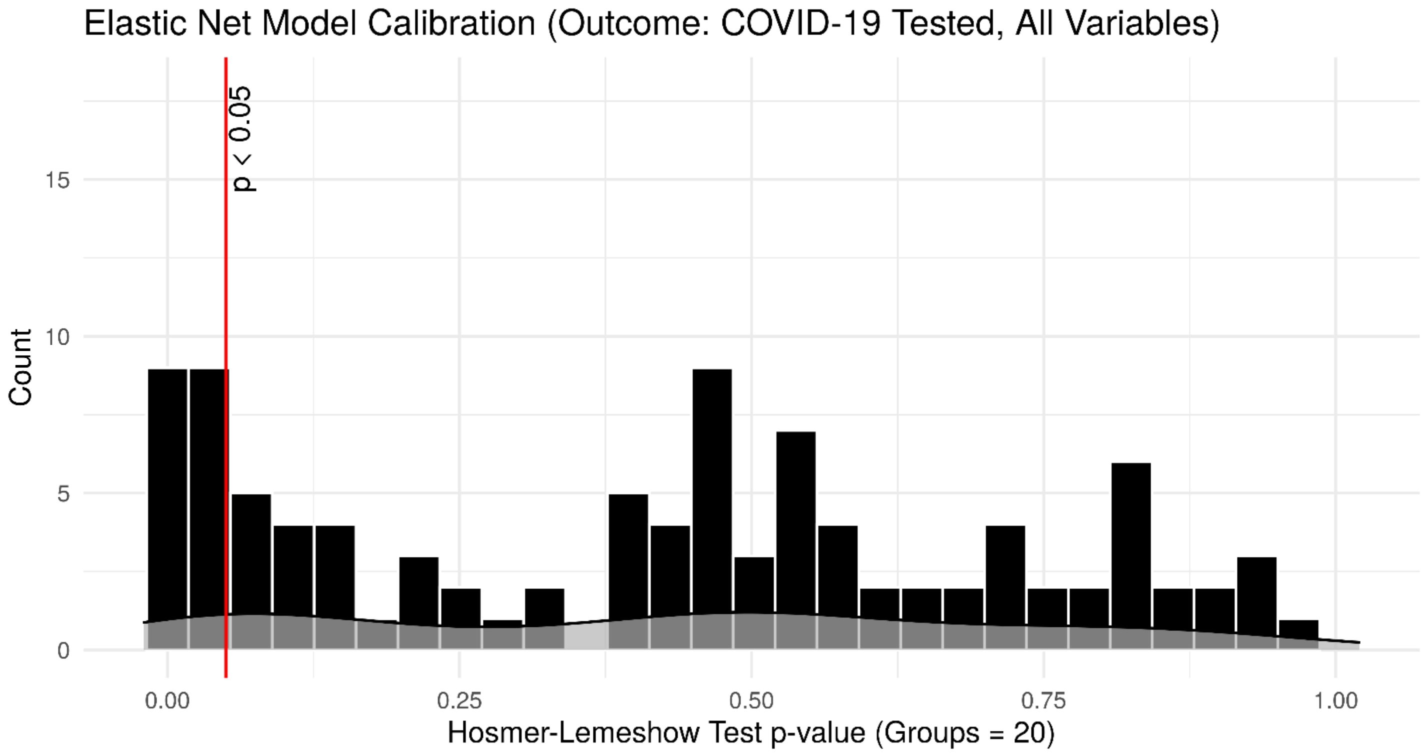

We plotted the p-values from conducting a Hosmer-Lemeshow goodness of fit test on all 100 train/test splits of the model evaluation procedure. Models tending to have poor calibration would show large numbers of p-values below the statistical significance threshold of 0.05.

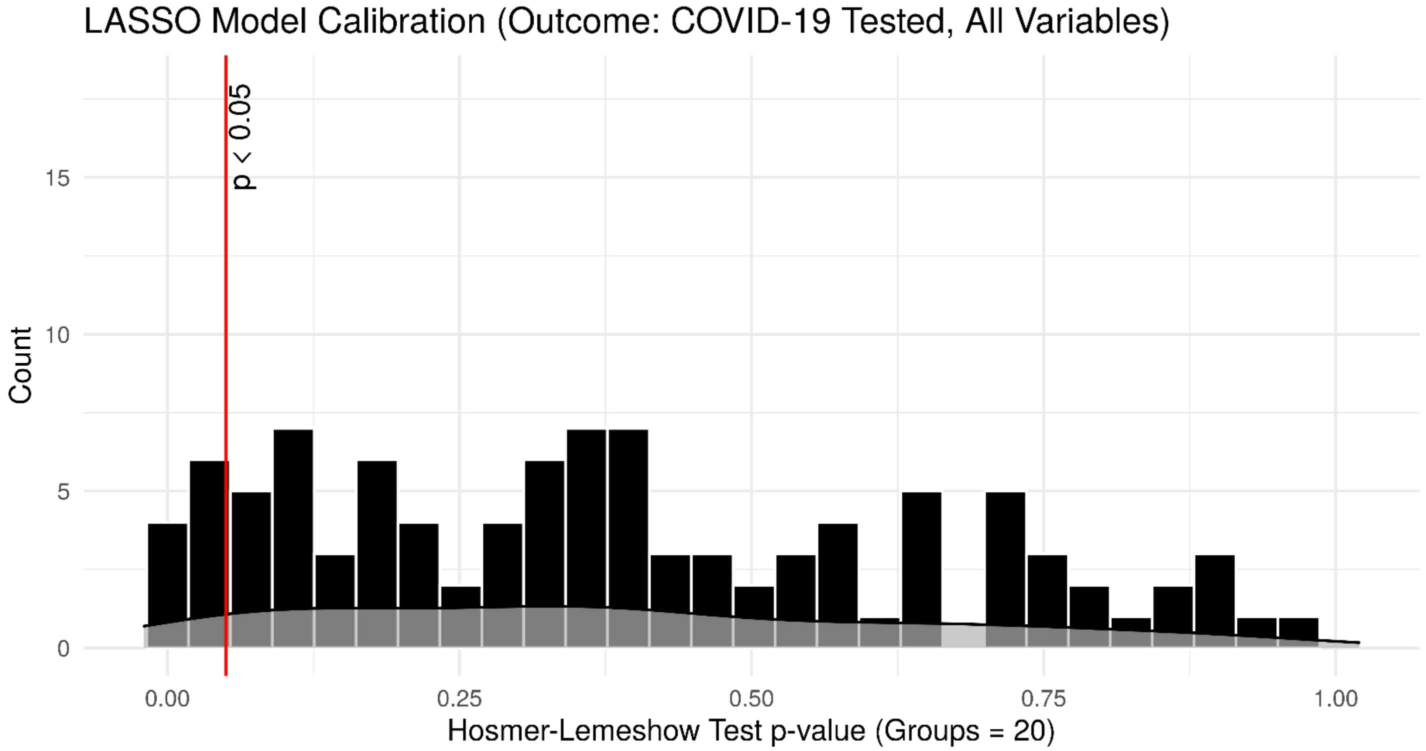

We plotted the p-values from conducting a Hosmer-Lemeshow goodness of fit test on all 100 train/test splits of the model evaluation procedure. Models tending to have poor calibration would show large numbers of p-values below the statistical significance threshold of 0.05.

S14 File. Description and Results of EHR-Based Case-Control Analysis

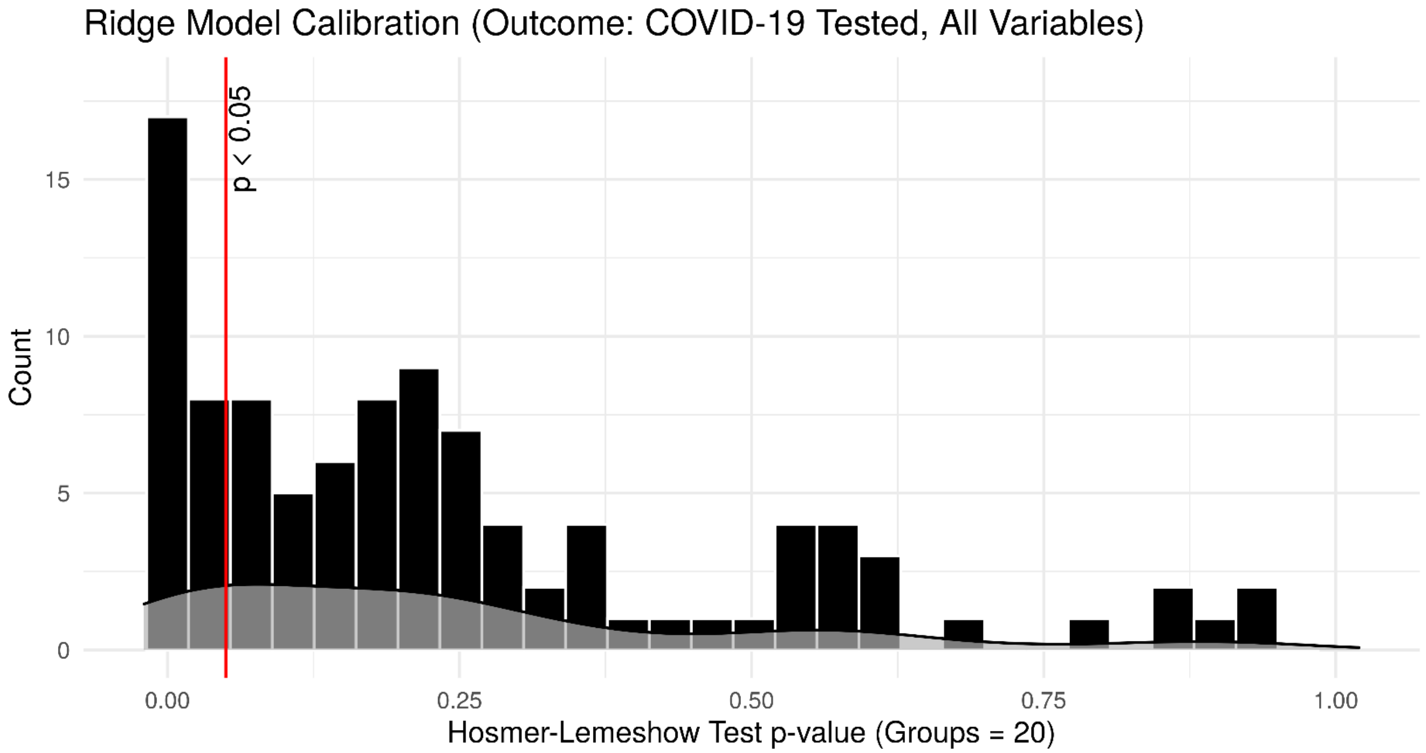

We plotted the p-values from conducting a Hosmer-Lemeshow goodness of fit test on all 100 train/test splits of the model evaluation procedure. Models tending to have poor calibration would show large numbers of p-values below the statistical significance threshold of 0.05.

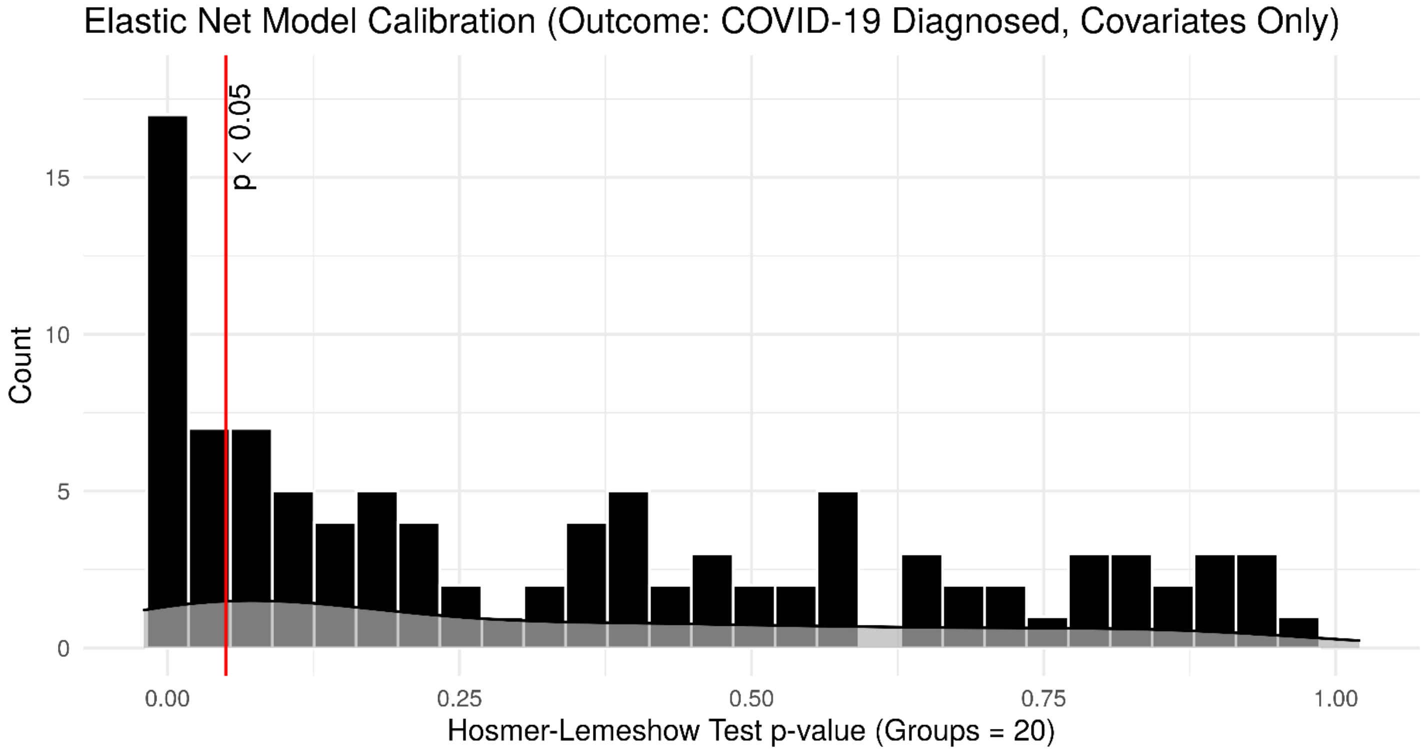

We plotted the p-values from conducting a Hosmer-Lemeshow goodness of fit test on all 100 train/test splits of the model evaluation procedure. Models tending to have poor calibration would show large numbers of p-values below the statistical significance threshold of 0.05.

S14 File. Description and Results of EHR-Based Case-Control Analysis

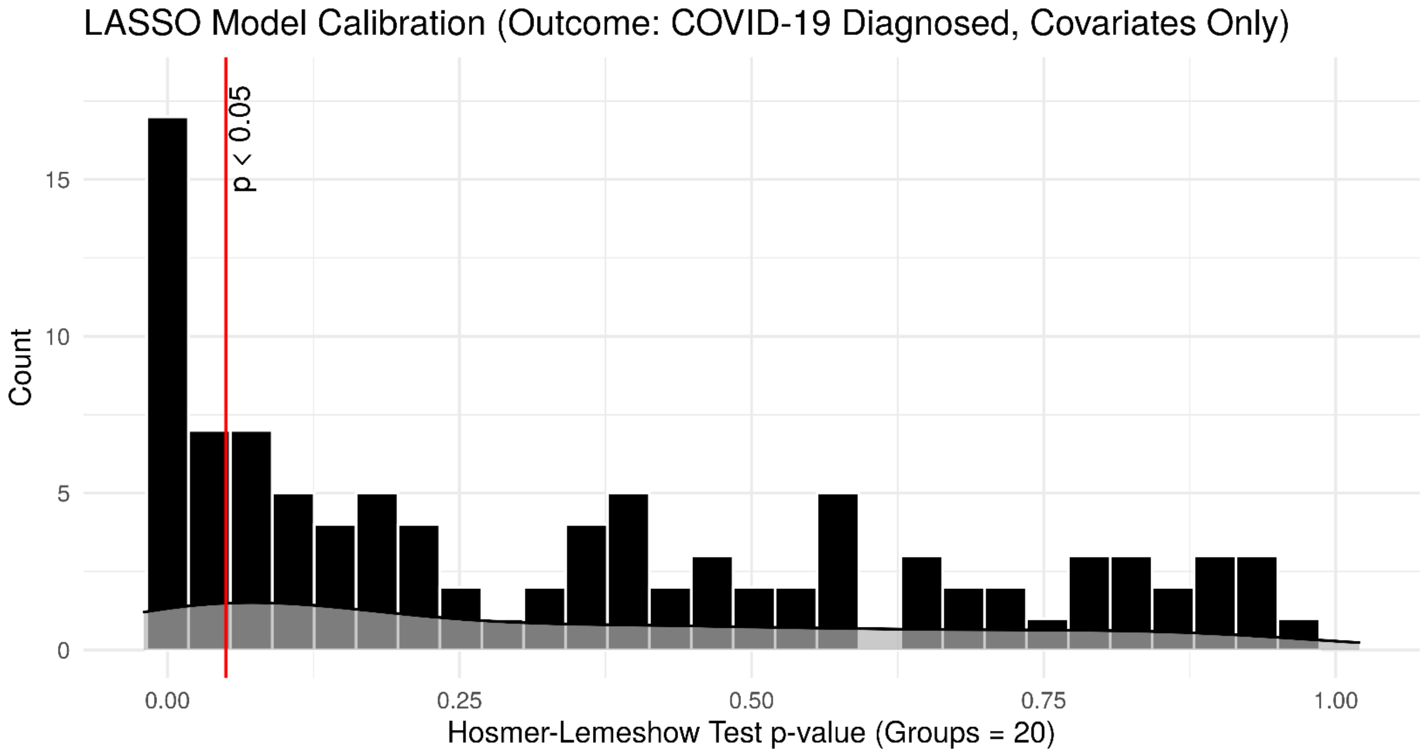

We plotted the p-values from conducting a Hosmer-Lemeshow goodness of fit test on all 100 train/test splits of the model evaluation procedure. Models tending to have poor calibration would show large numbers of p-values below the statistical significance threshold of 0.05.

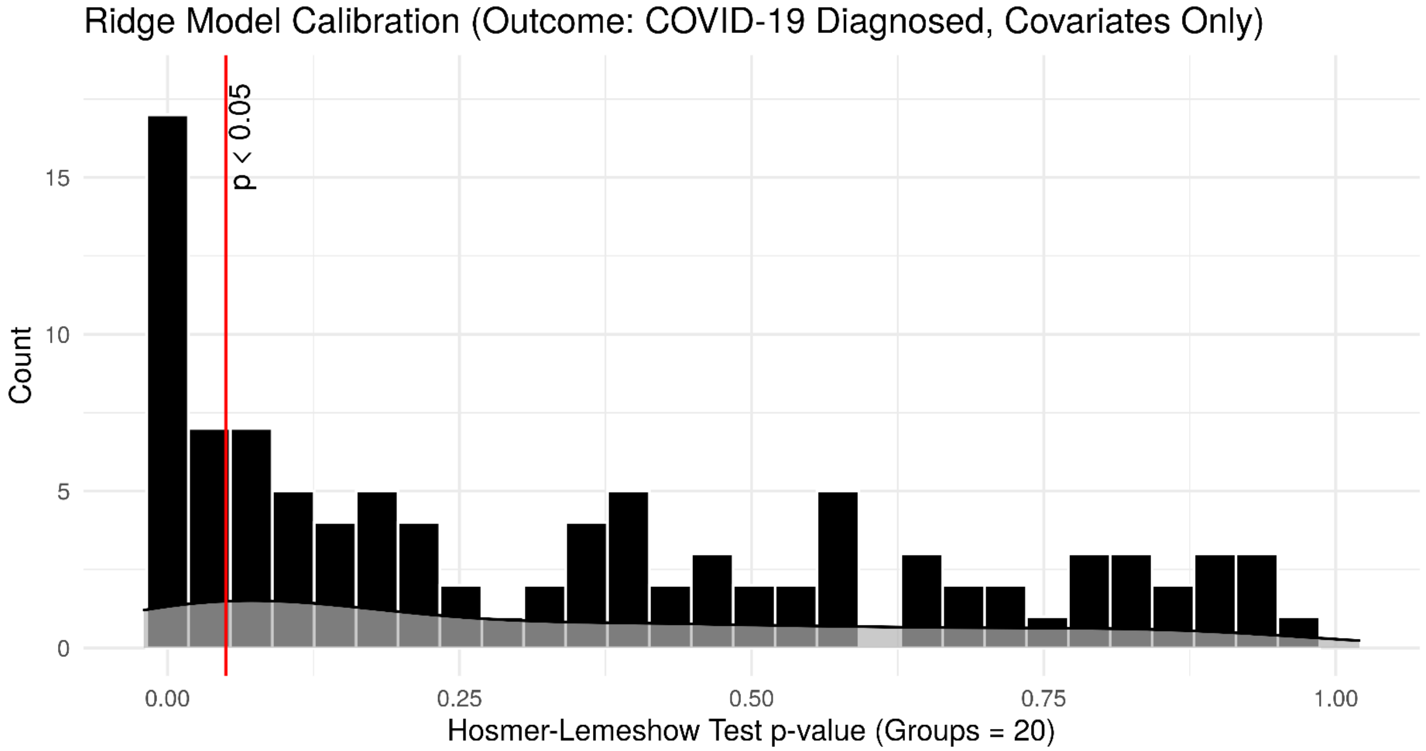

We plotted the p-values from conducting a Hosmer-Lemeshow goodness of fit test on all 100 train/test splits of the model evaluation procedure. Models tending to have poor calibration would show large numbers of p-values below the statistical significance threshold of 0.05.

S14 File. Description and Results of EHR-Based Case-Control Analysis

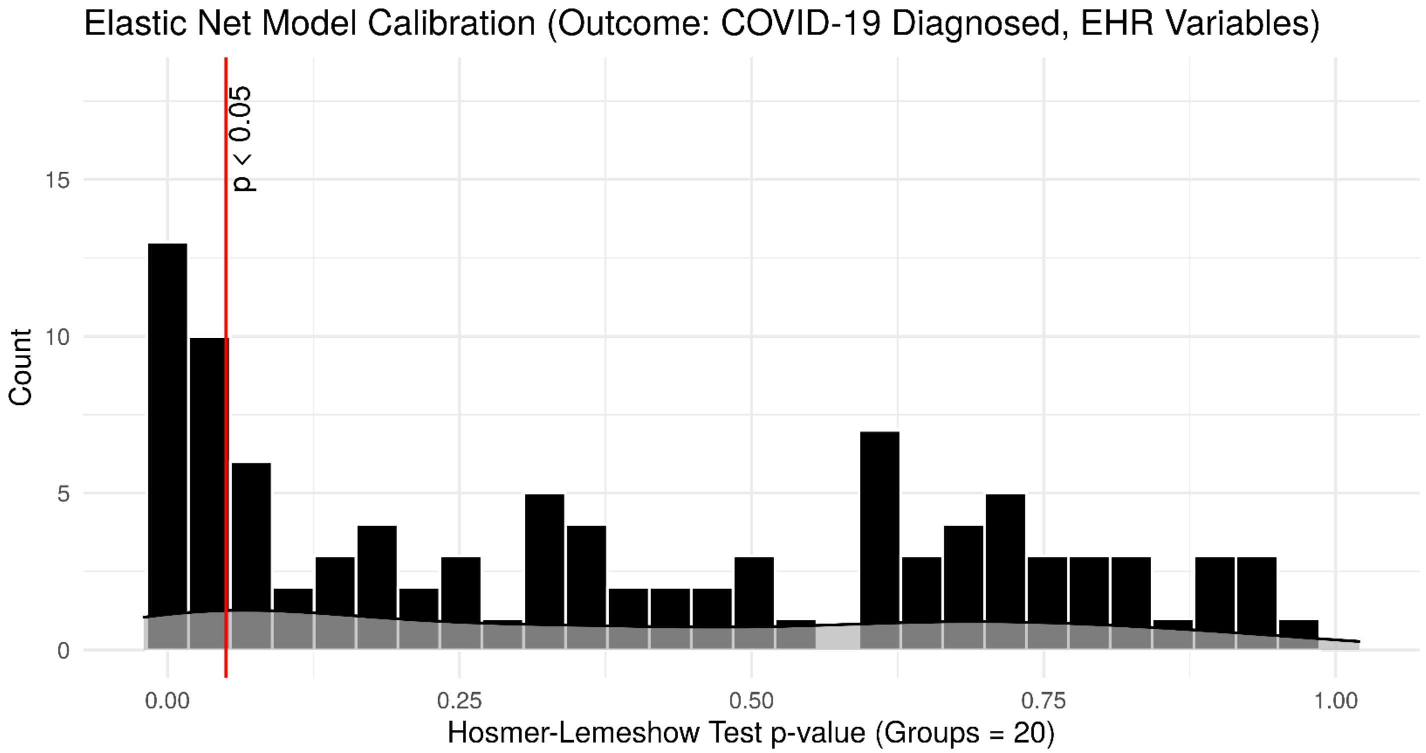

We plotted the p-values from conducting a Hosmer-Lemeshow goodness of fit test on all 100 train/test splits of the model evaluation procedure. Models tending to have poor calibration would show large numbers of p-values below the statistical significance threshold of 0.05.

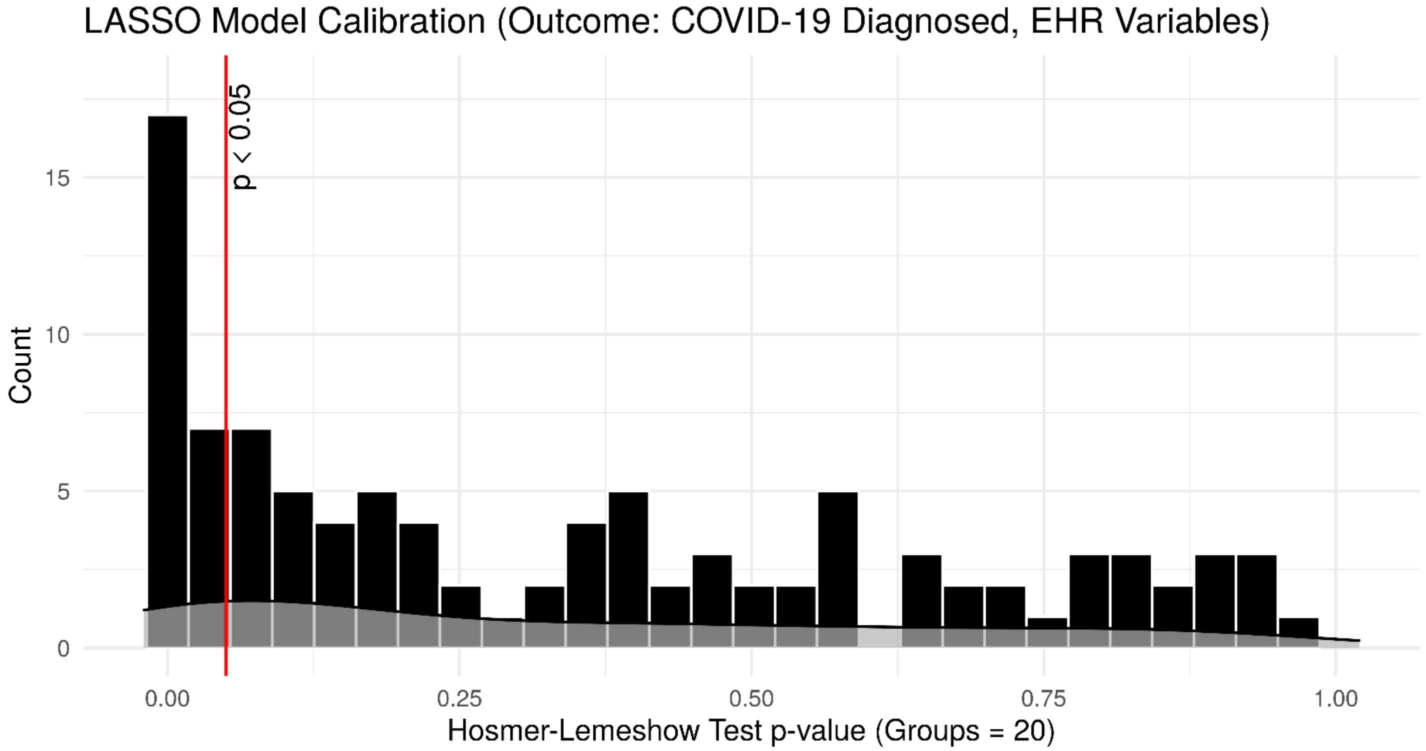

We plotted the p-values from conducting a Hosmer-Lemeshow goodness of fit test on all 100 train/test splits of the model evaluation procedure. Models tending to have poor calibration would show large numbers of p-values below the statistical significance threshold of 0.05.

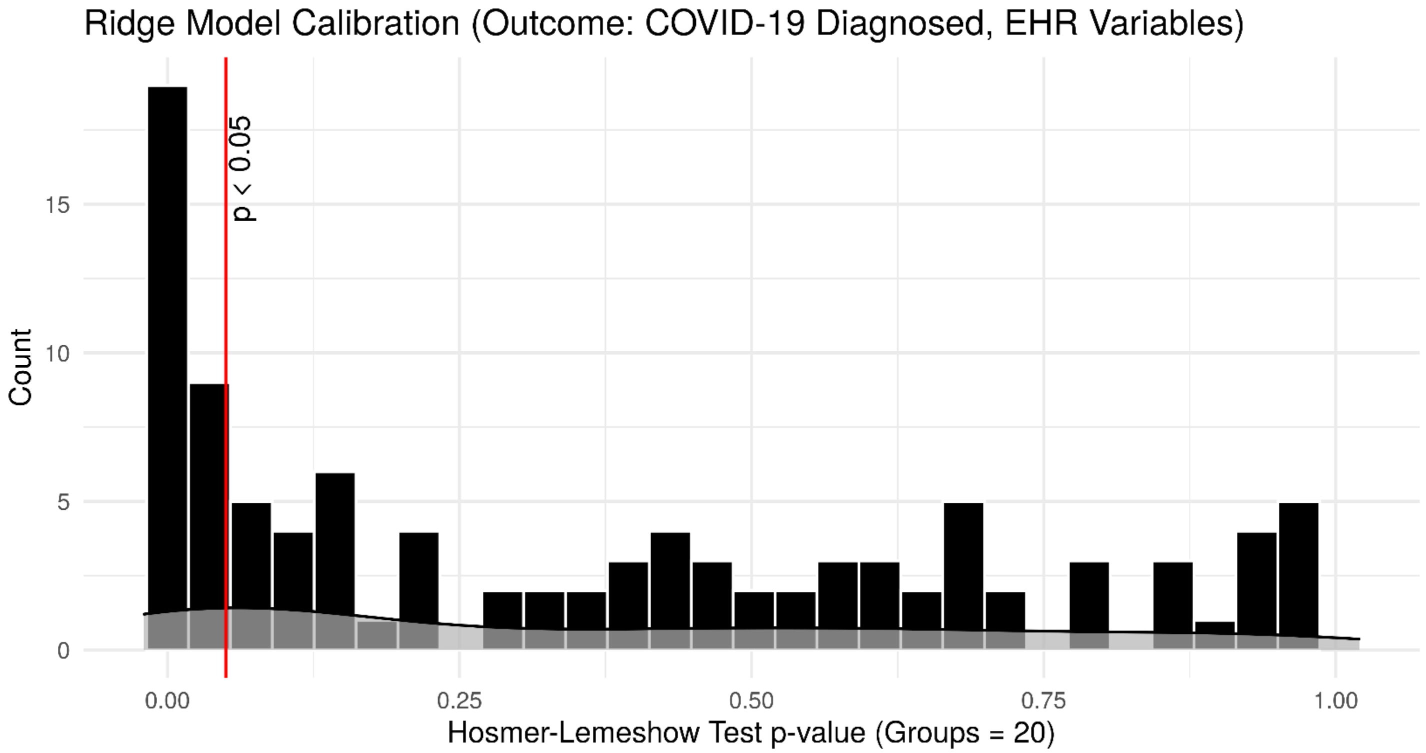

We plotted the p-values from conducting a Hosmer-Lemeshow goodness of fit test on all 100 train/test splits of the model evaluation procedure. Models tending to have poor calibration would show large numbers of p-values below the statistical significance threshold of 0.05.

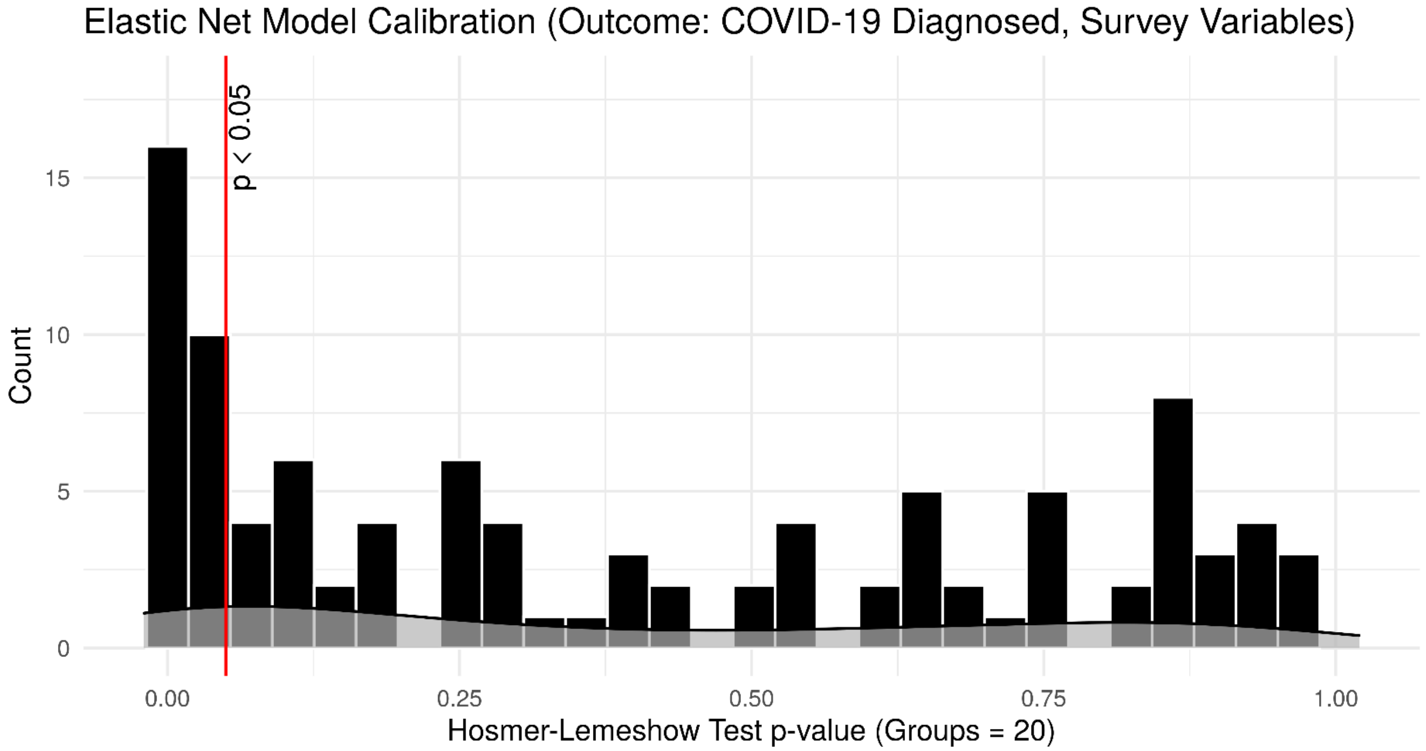

We plotted the p-values from conducting a Hosmer-Lemeshow goodness of fit test on all 100 train/test splits of the model evaluation procedure. Models tending to have poor calibration would show large numbers of p-values below the statistical significance threshold of 0.05.

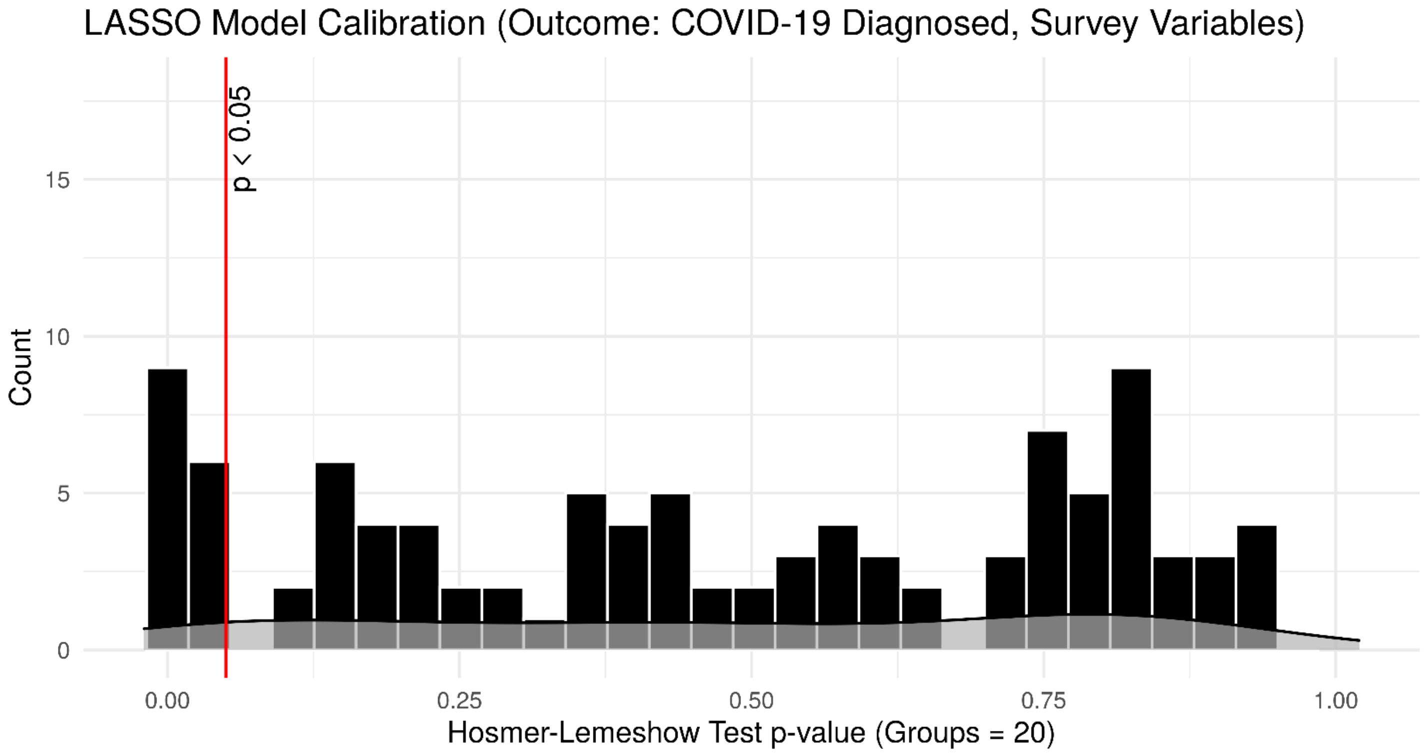

We plotted the p-values from conducting a Hosmer-Lemeshow goodness of fit test on all 100 train/test splits of the model evaluation procedure. Models tending to have poor calibration would show large numbers of p-values below the statistical significance threshold of 0.05.

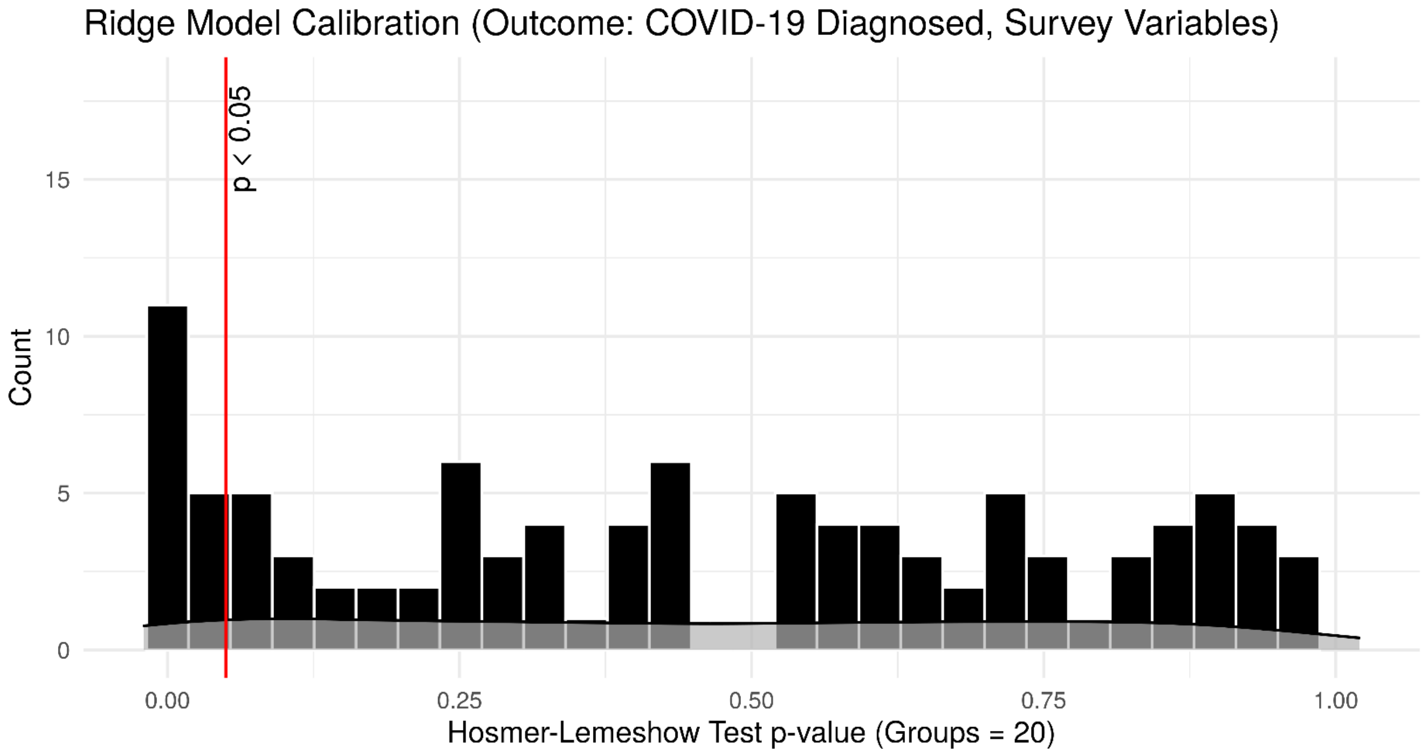

We plotted the p-values from conducting a Hosmer-Lemeshow goodness of fit test on all 100 train/test splits of the model evaluation procedure. Models tending to have poor calibration would show large numbers of p-values below the statistical significance threshold of 0.05.

S14 File. Description and Results of EHR-Based Case-Control Analysis

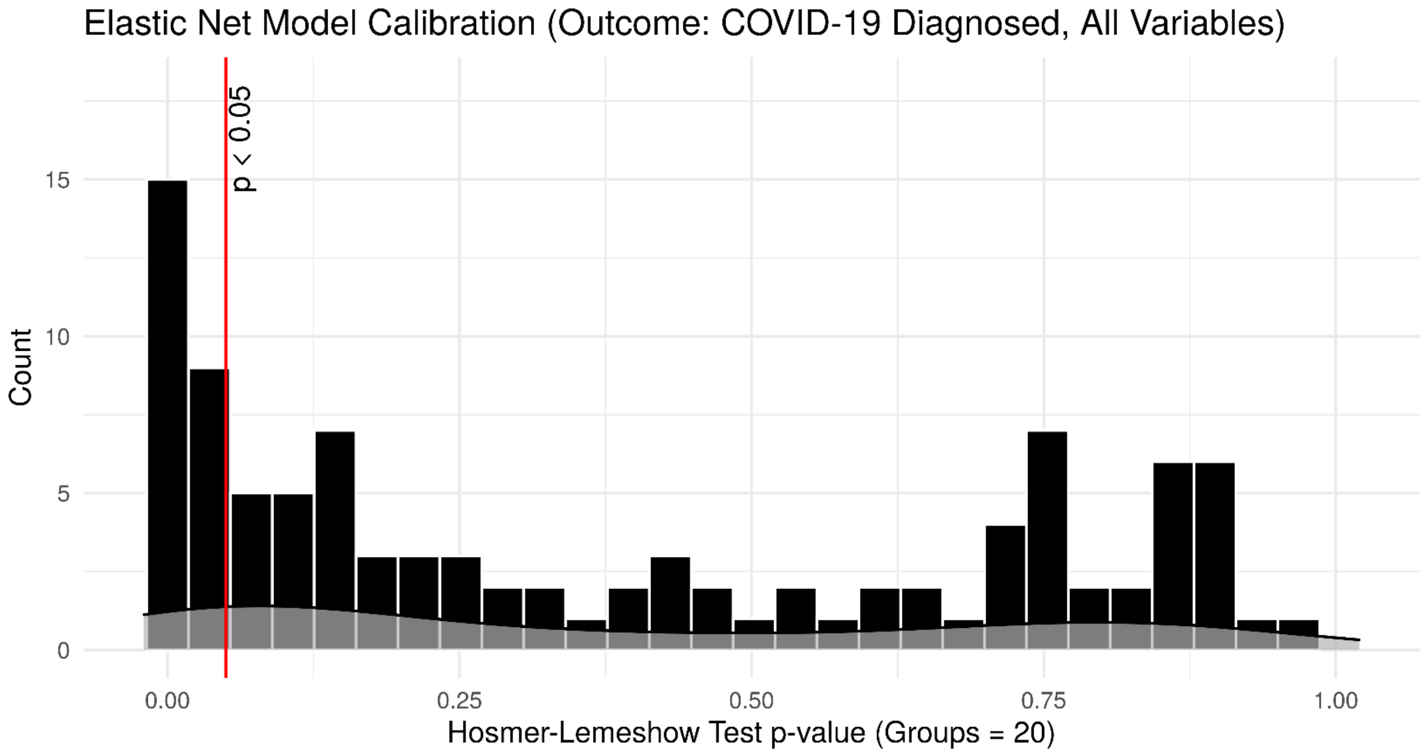

We plotted the p-values from conducting a Hosmer-Lemeshow goodness of fit test on all 100 train/test splits of the model evaluation procedure. Models tending to have poor calibration would show large numbers of p-values below the statistical significance threshold of 0.05.

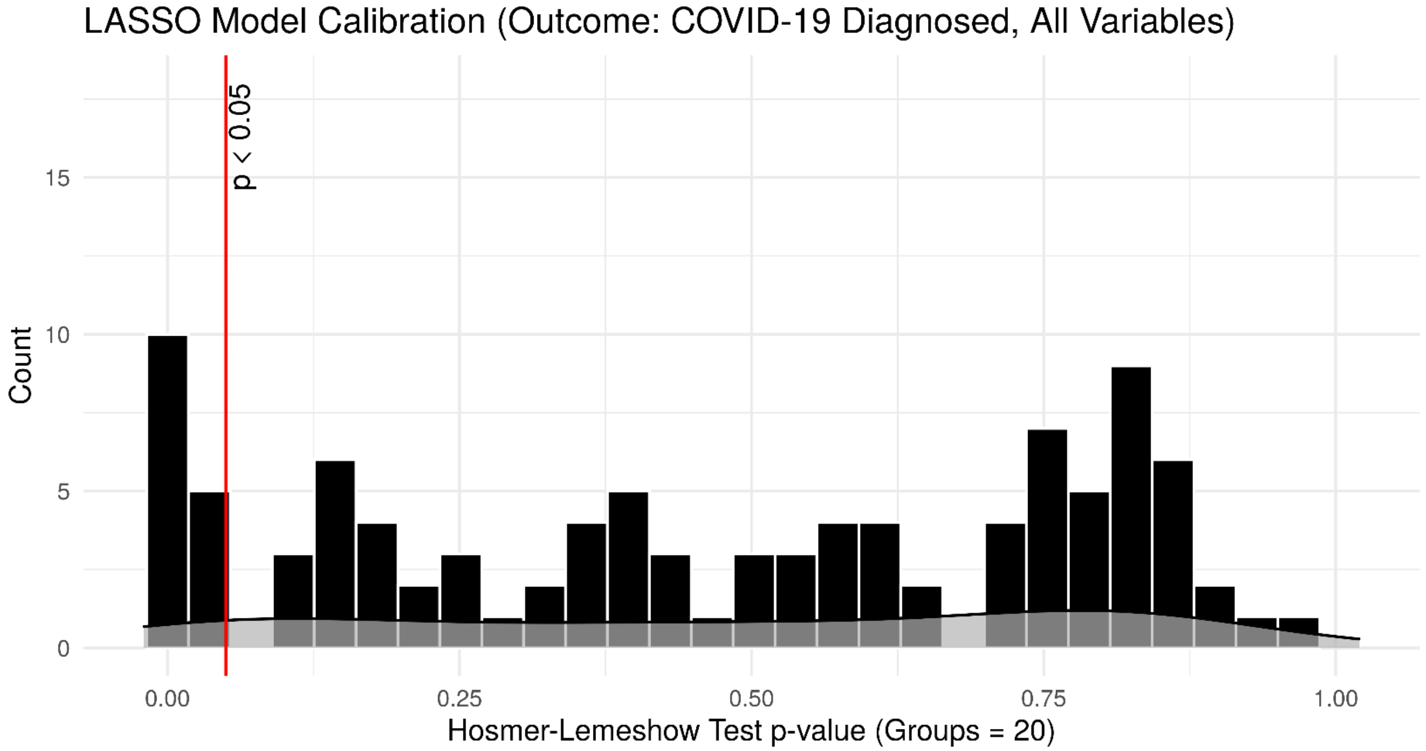

We plotted the p-values from conducting a Hosmer-Lemeshow goodness of fit test on all 100 train/test splits of the model evaluation procedure. Models tending to have poor calibration would show large numbers of p-values below the statistical significance threshold of 0.05.

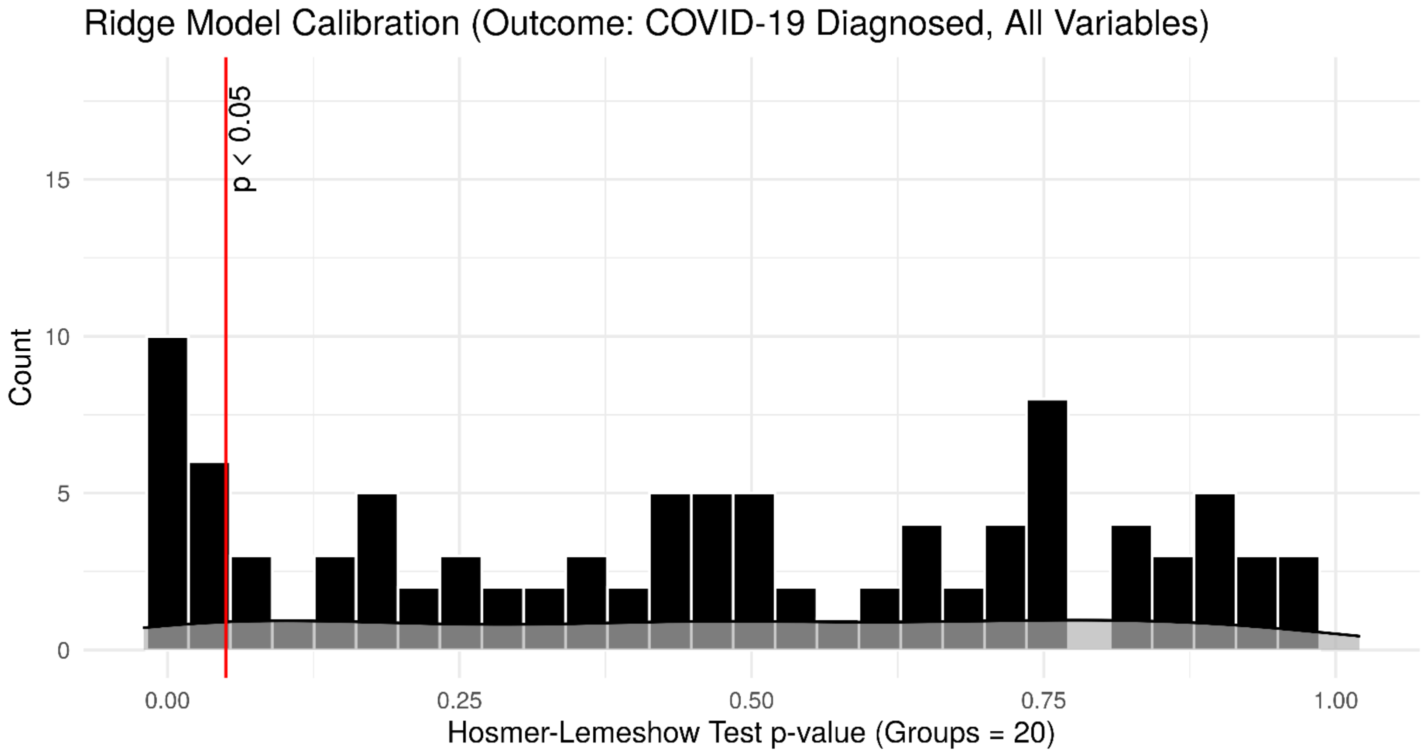

We plotted the p-values from conducting a Hosmer-Lemeshow goodness of fit test on all 100 train/test splits of the model evaluation procedure. Models tending to have poor calibration would show large numbers of p-values below the statistical significance threshold of 0.05.

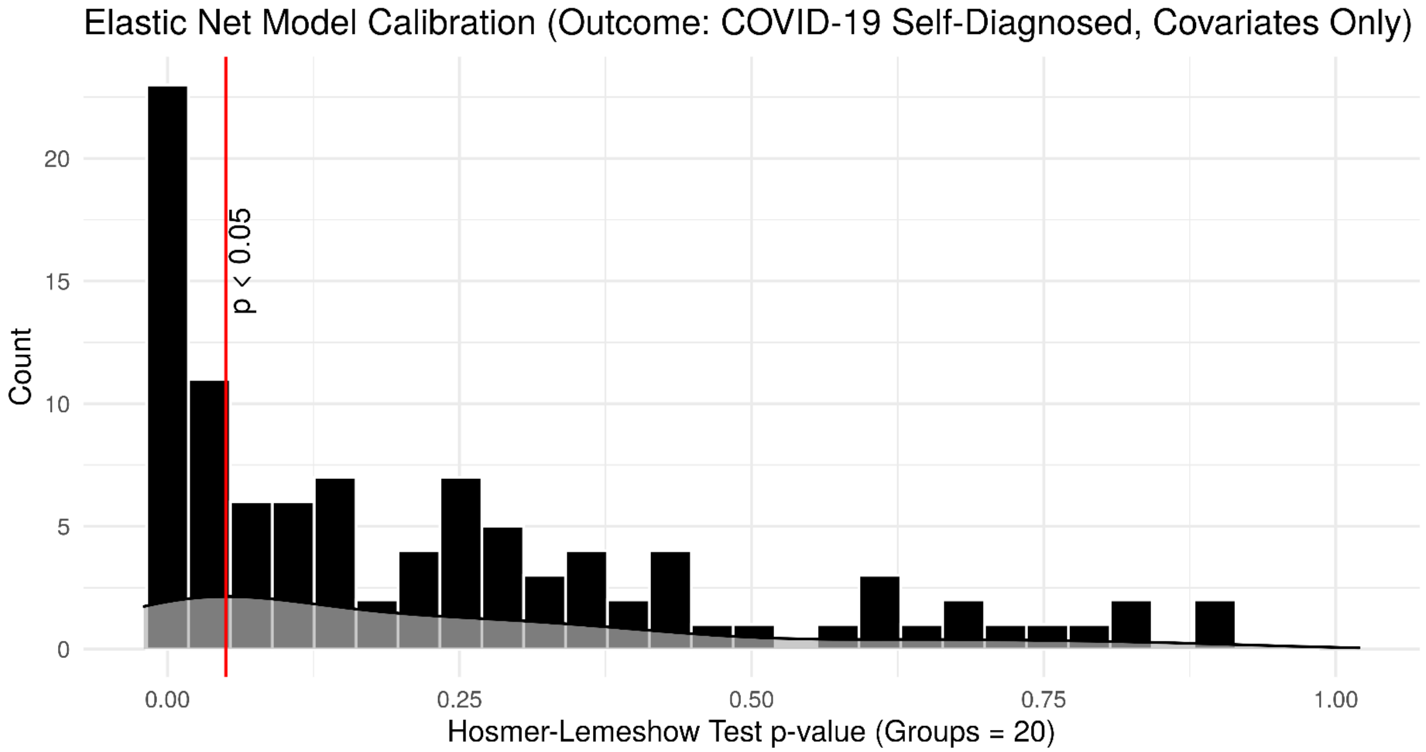

We plotted the p-values from conducting a Hosmer-Lemeshow goodness of fit test on all 100 train/test splits of the model evaluation procedure. Models tending to have poor calibration would show large numbers of p-values below the statistical significance threshold of 0.05.

S14 File. Description and Results of EHR-Based Case-Control Analysis

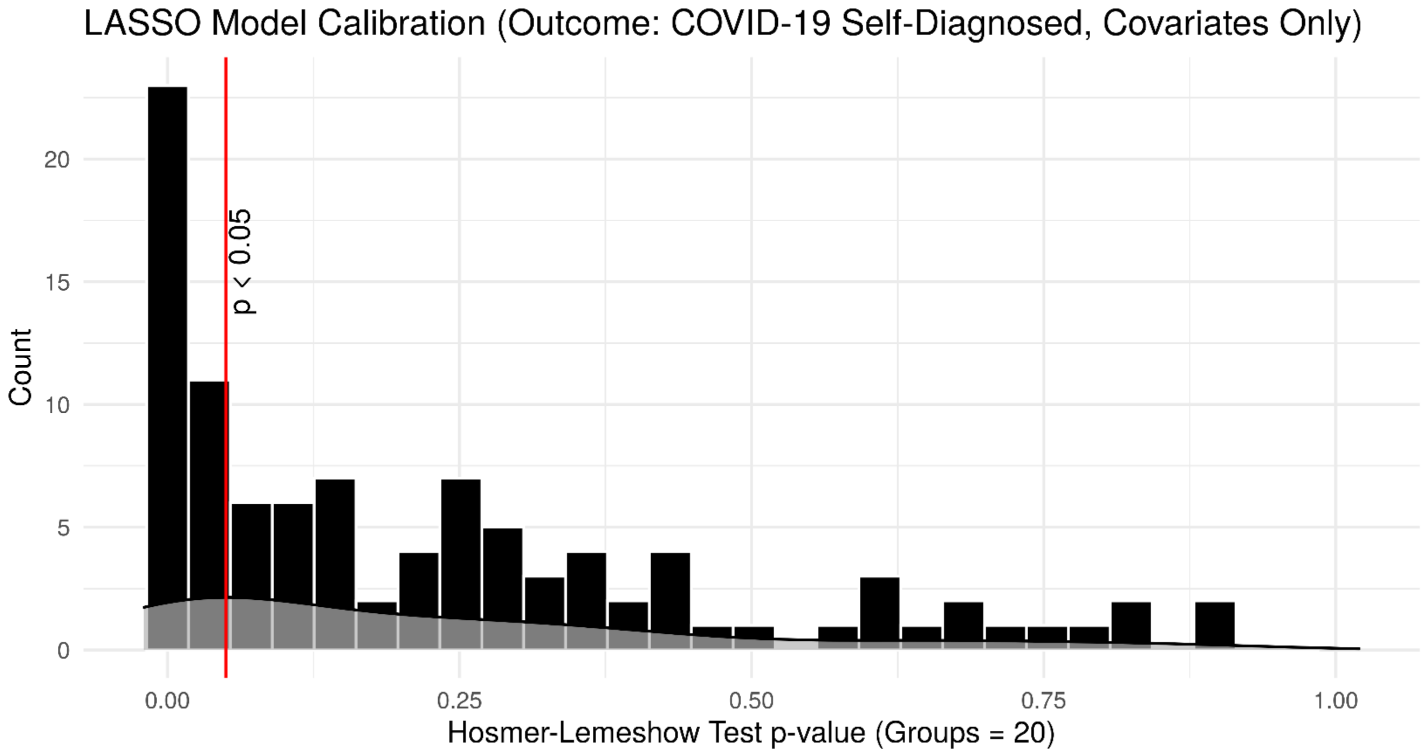

We plotted the p-values from conducting a Hosmer-Lemeshow goodness of fit test on all 100 train/test splits of the model evaluation procedure. Models tending to have poor calibration would show large numbers of p-values below the statistical significance threshold of 0.05.

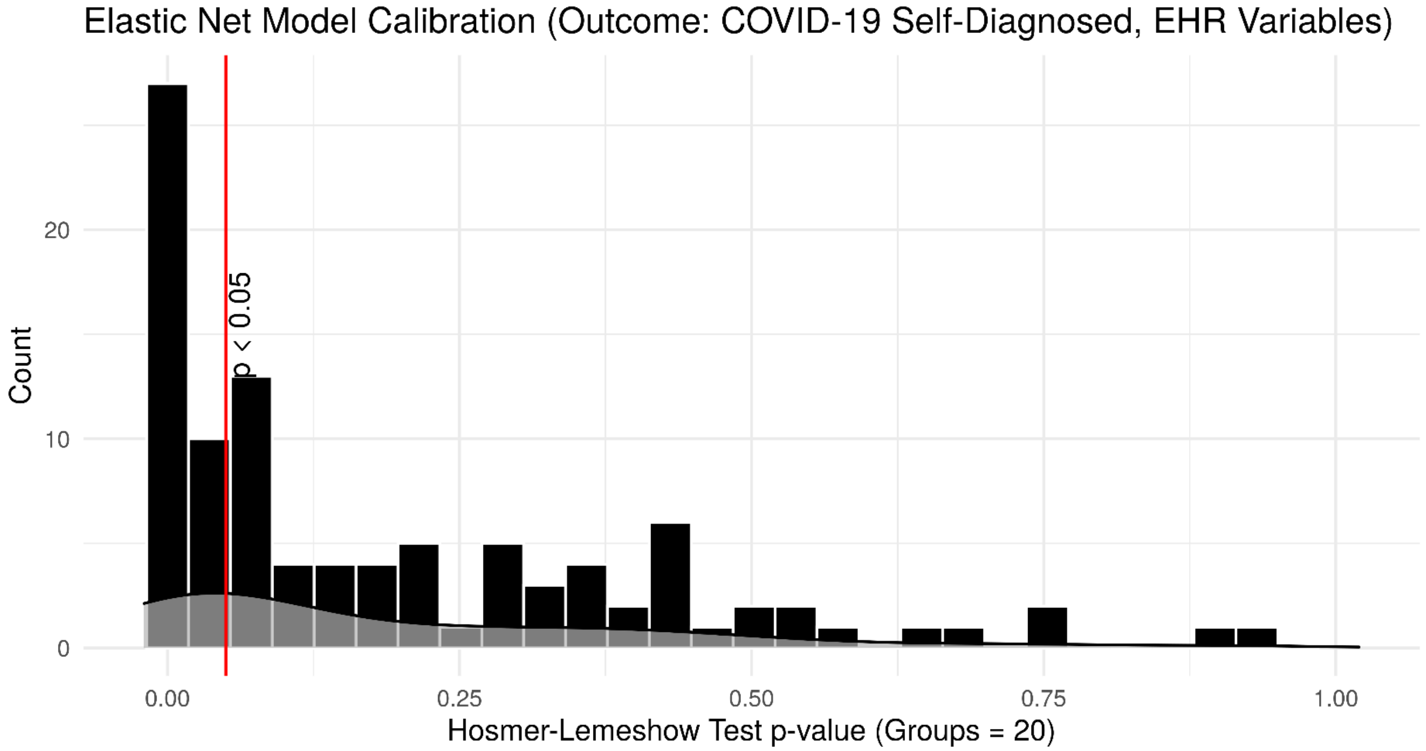

We plotted the p-values from conducting a Hosmer-Lemeshow goodness of fit test on all 100 train/test splits of the model evaluation procedure. Models tending to have poor calibration would show large numbers of p-values below the statistical significance threshold of 0.05.

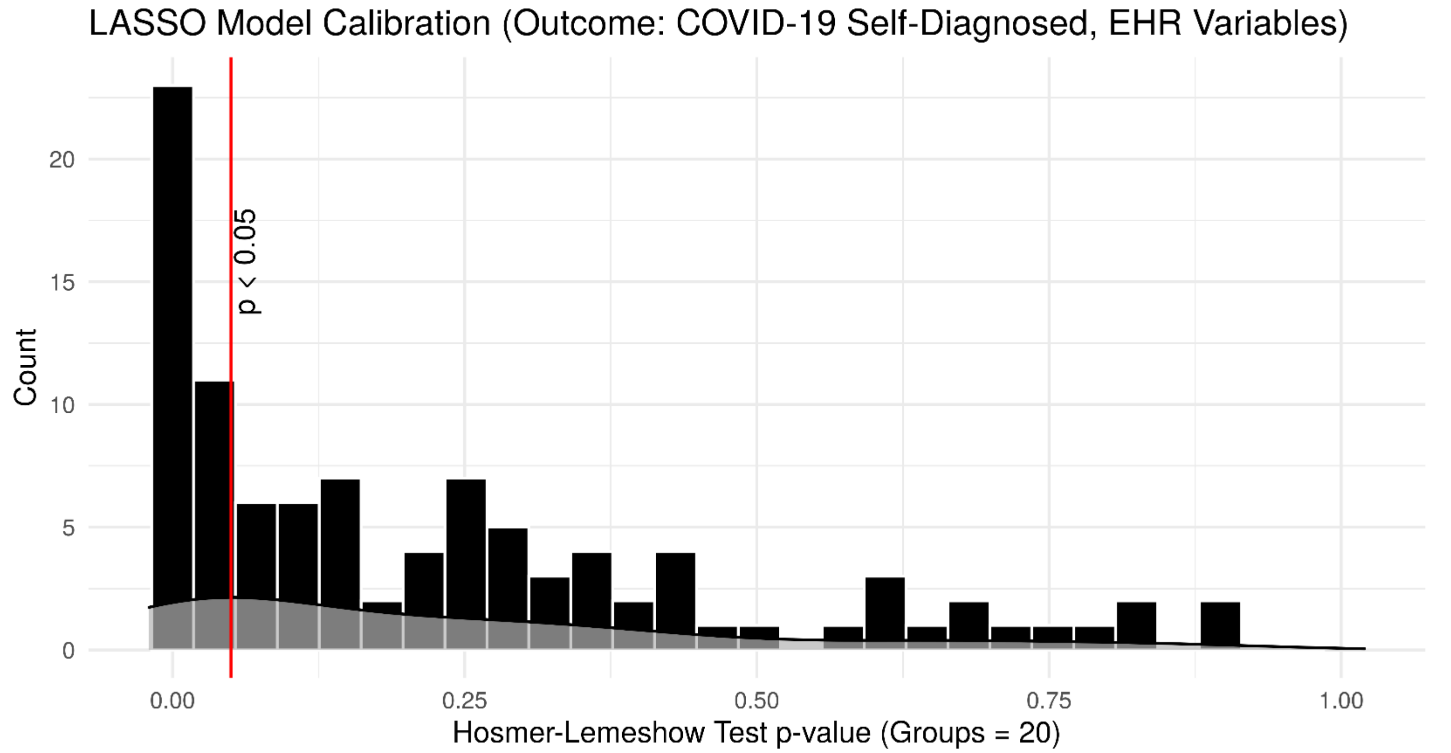

We plotted the p-values from conducting a Hosmer-Lemeshow goodness of fit test on all 100 train/test splits of the model evaluation procedure. Models tending to have poor calibration would show large numbers of p-values below the statistical significance threshold of 0.05.

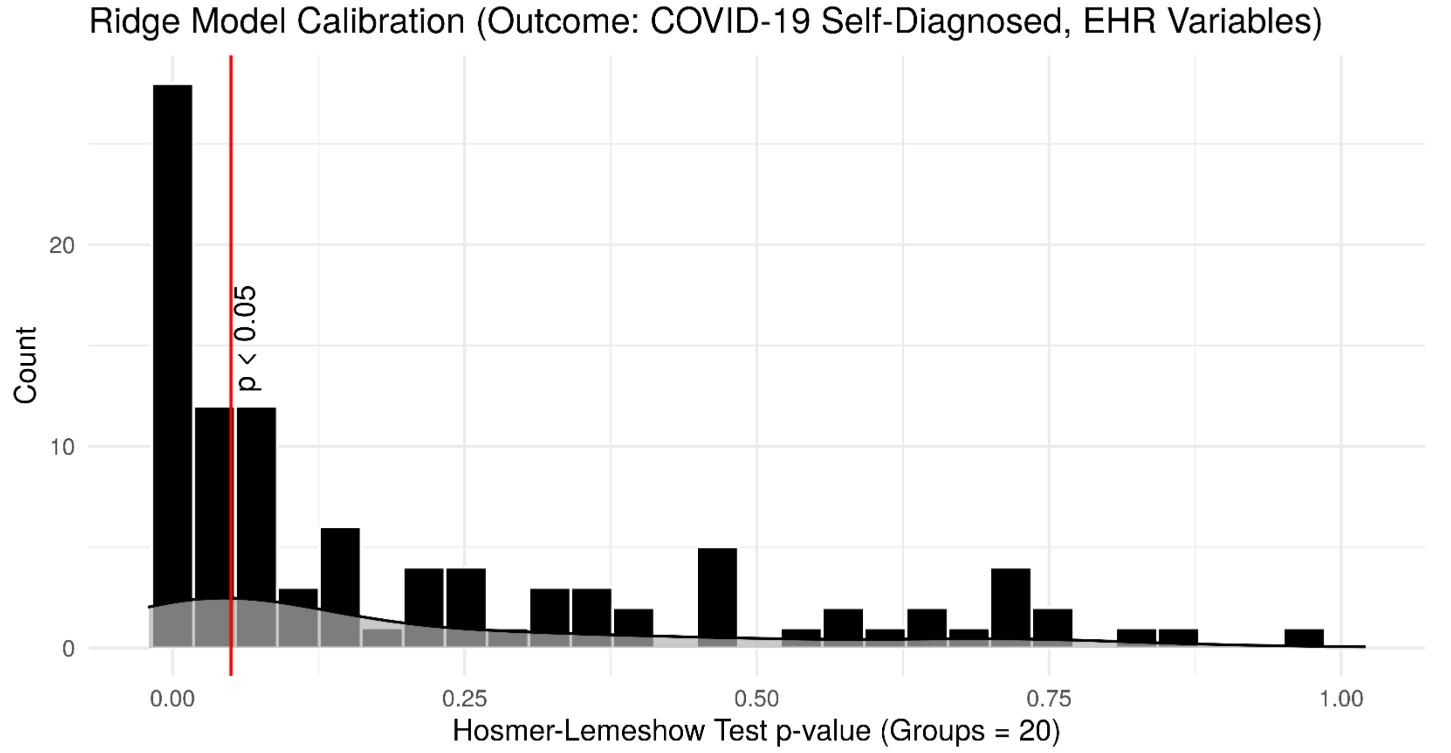

We plotted the p-values from conducting a Hosmer-Lemeshow goodness of fit test on all 100 train/test splits of the model evaluation procedure. Models tending to have poor calibration would show large numbers of p-values below the statistical significance threshold of 0.05.

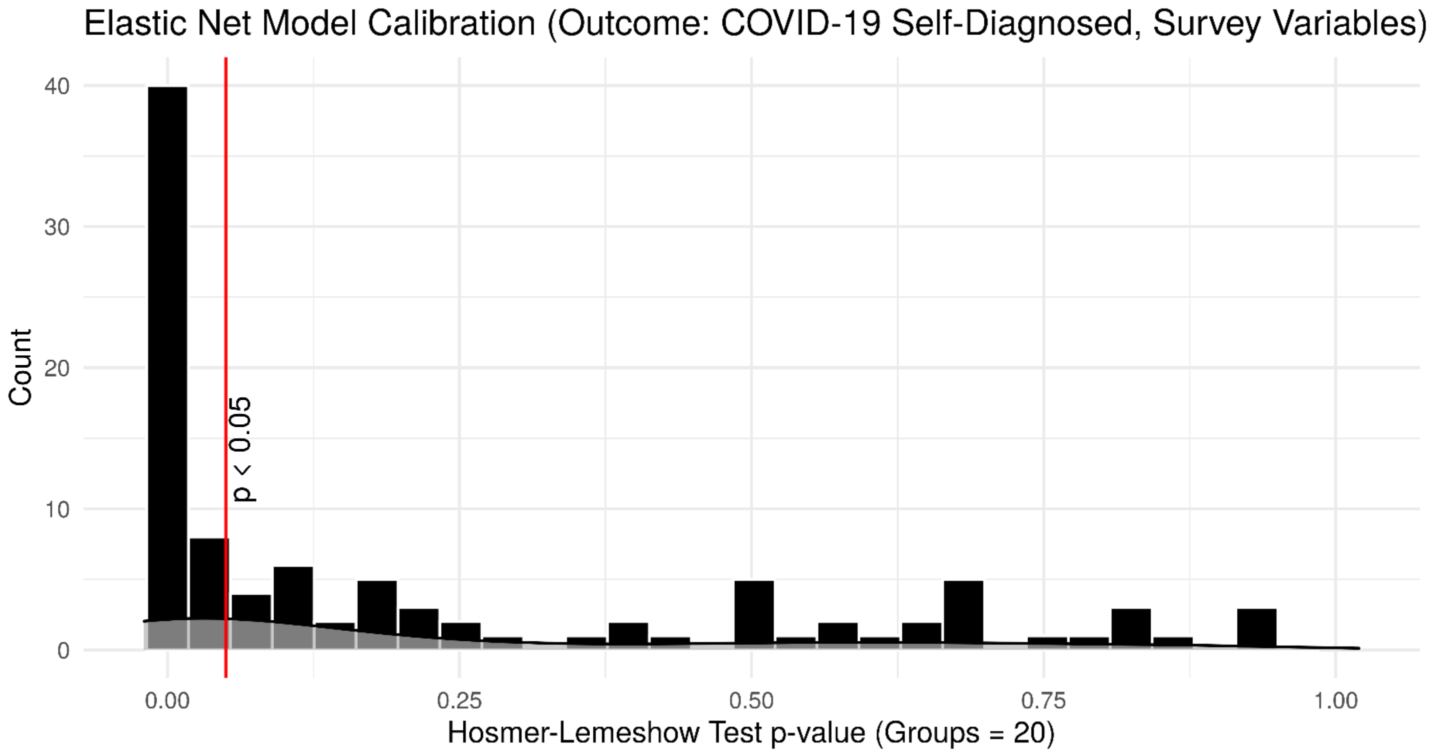

We plotted the p-values from conducting a Hosmer-Lemeshow goodness of fit test on all 100 train/test splits of the model evaluation procedure. Models tending to have poor calibration would show large numbers of p-values below the statistical significance threshold of 0.05.

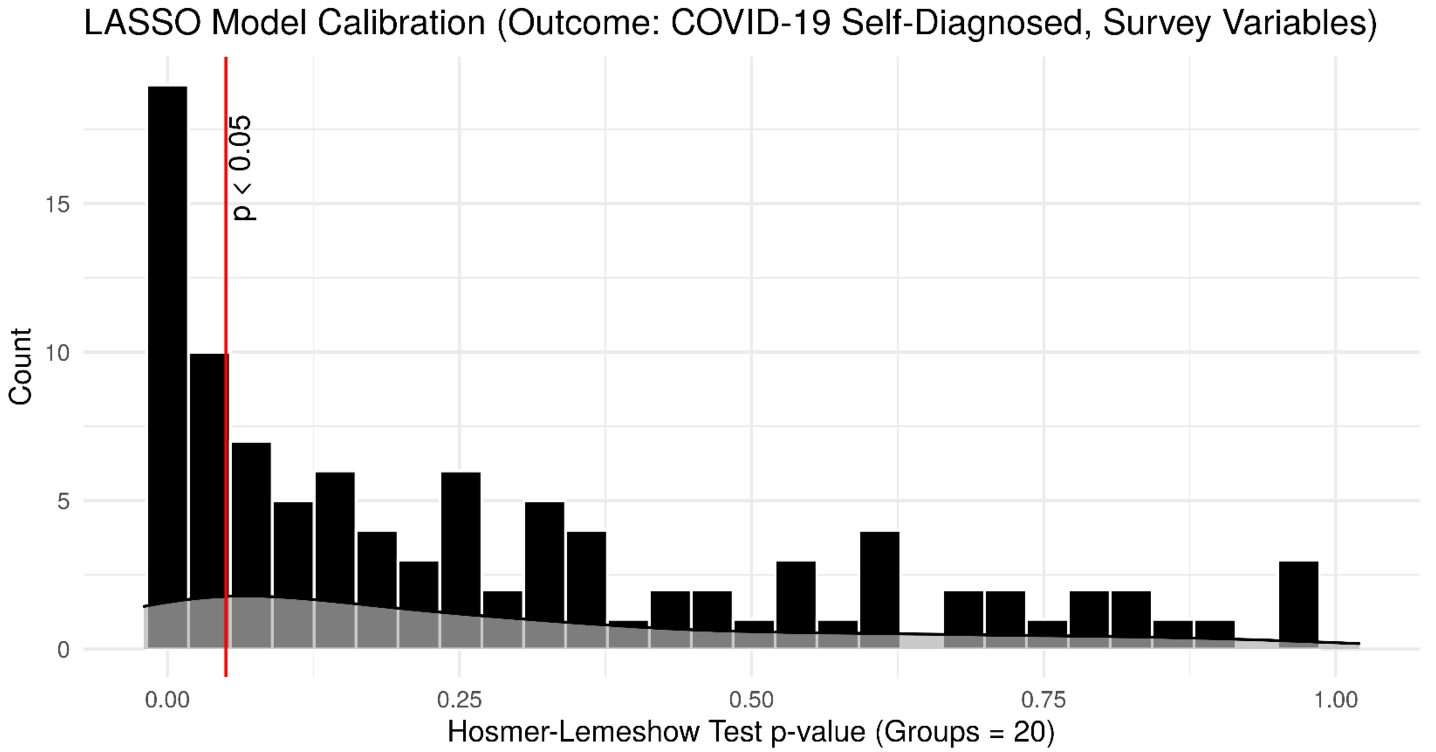

We plotted the p-values from conducting a Hosmer-Lemeshow goodness of fit test on all 100 train/test splits of the model evaluation procedure. Models tending to have poor calibration would show large numbers of p-values below the statistical significance threshold of 0.05.

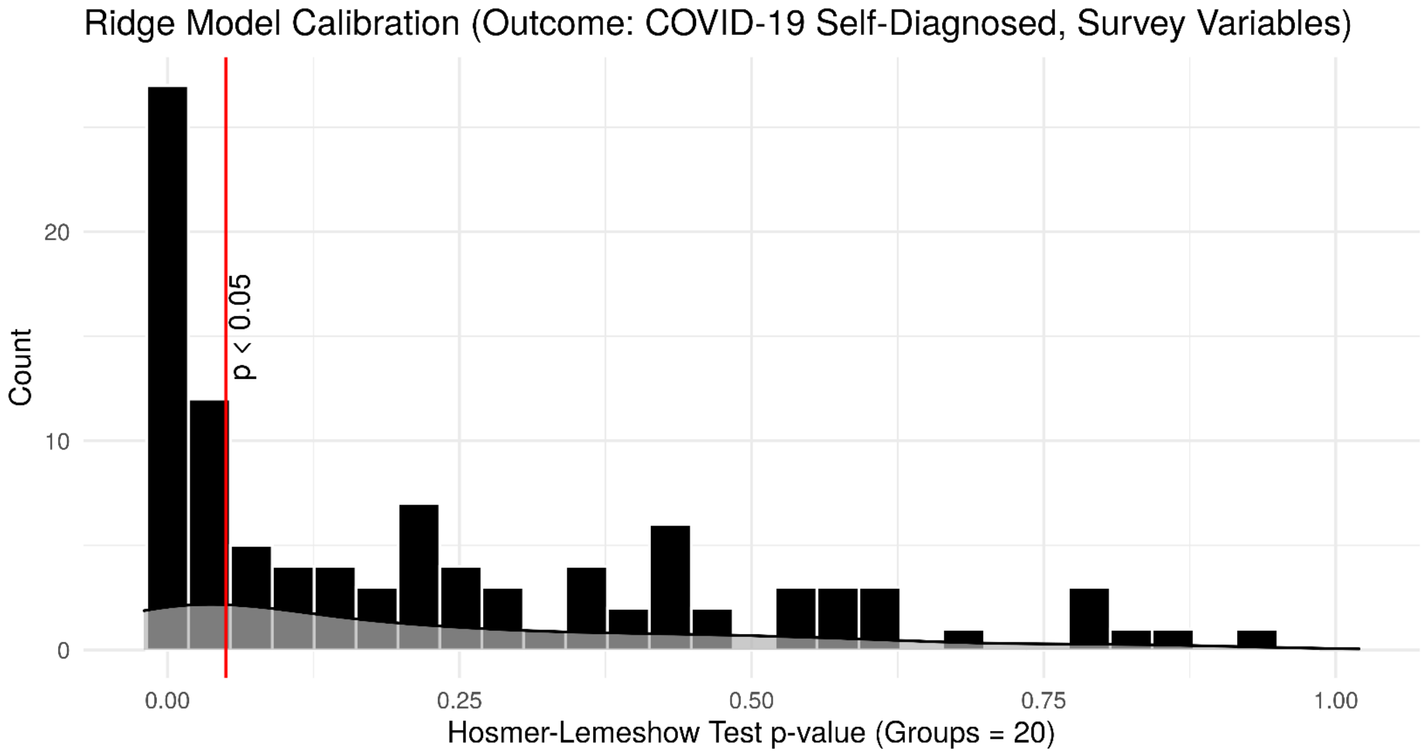

We plotted the p-values from conducting a Hosmer-Lemeshow goodness of fit test on all 100 train/test splits of the model evaluation procedure. Models tending to have poor calibration would show large numbers of p-values below the statistical significance threshold of 0.05.

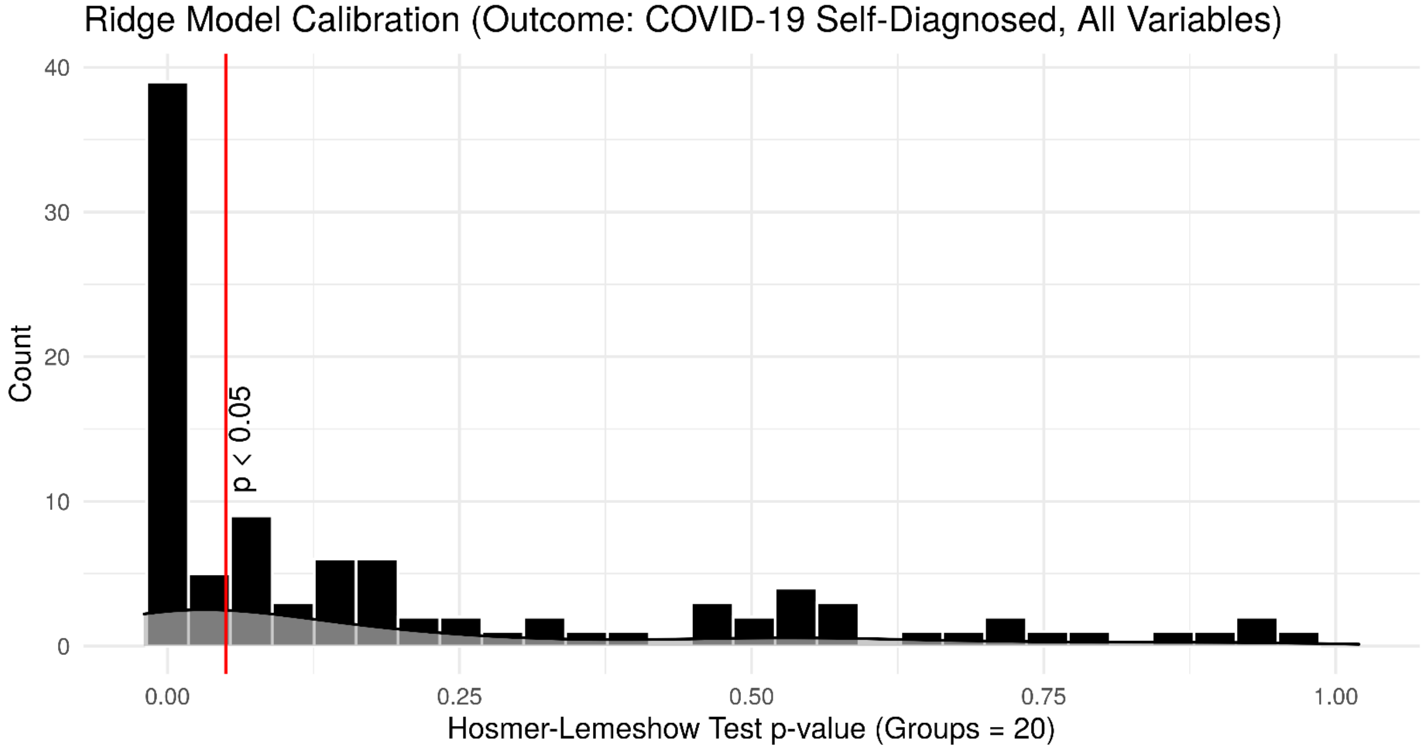

We plotted the p-values from conducting a Hosmer-Lemeshow goodness of fit test on all 100 train/test splits of the model evaluation procedure. Models tending to have poor calibration would show large numbers of p-values below the statistical significance threshold of 0.05.

S14 File. Description and Results of EHR-Based Case-Control Analysis

LASSO Model Calibration (Outcome: COVID-19 Self-Diagnosed, All Variables)

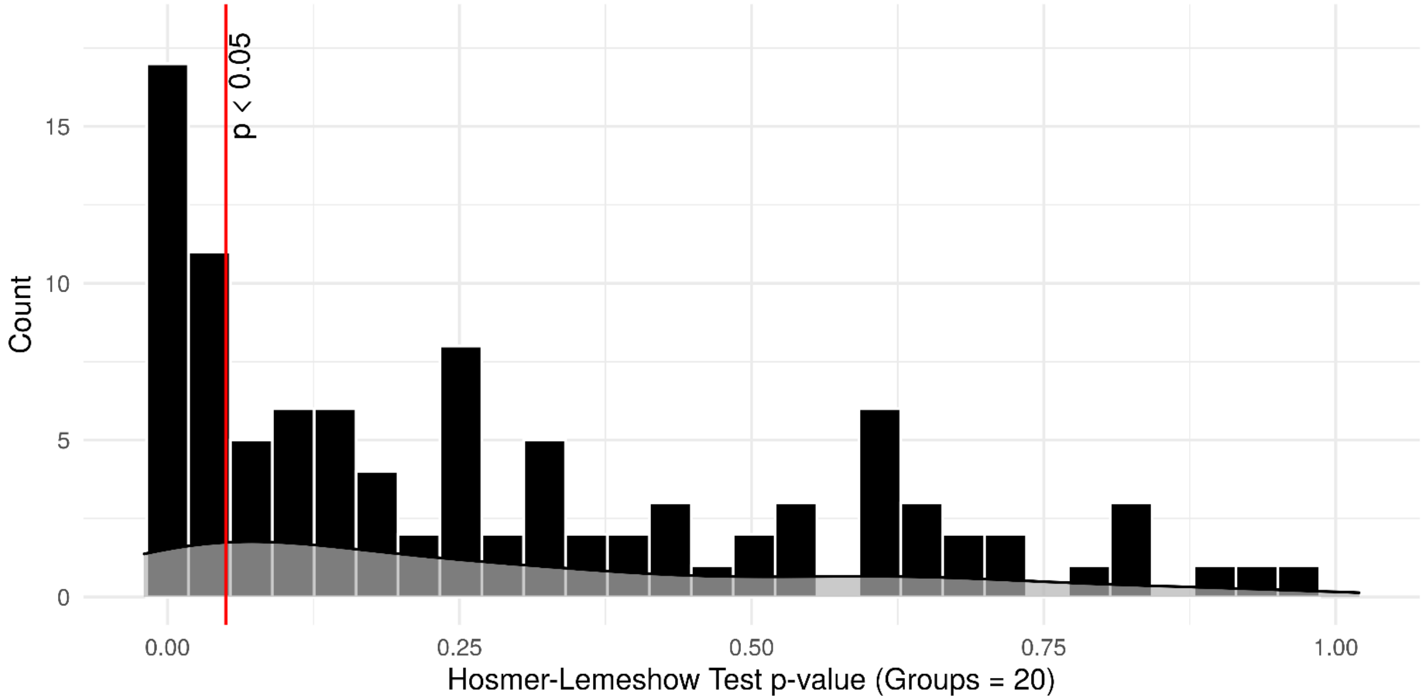

We plotted the p-values from conducting a Hosmer-Lemeshow goodness of fit test on all 100 train/test splits of the model evaluation procedure. Models tending to have poor calibration would show large numbers of p-values below the statistical significance threshold of 0.05.

Ridge Model Calibration (Outcome: COVID-19 Self-Diagnosed, All Variables)

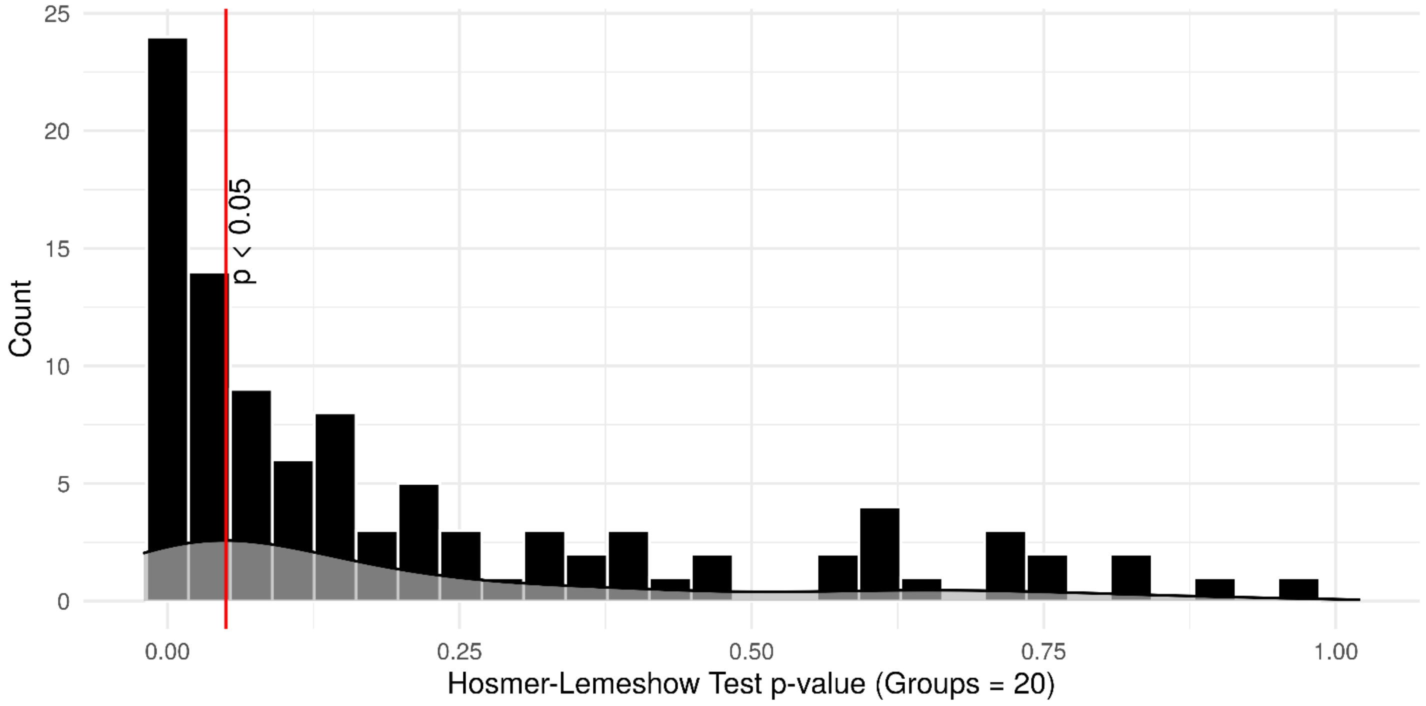

We plotted the p-values from conducting a Hosmer-Lemeshow goodness of fit test on all 100 train/test splits of the model evaluation procedure. Models tending to have poor calibration would show large numbers of p-values below the statistical significance threshold of 0.05.

S15 File. Calibration Plots for Survey-Based Models of COVID-19 Outcomes

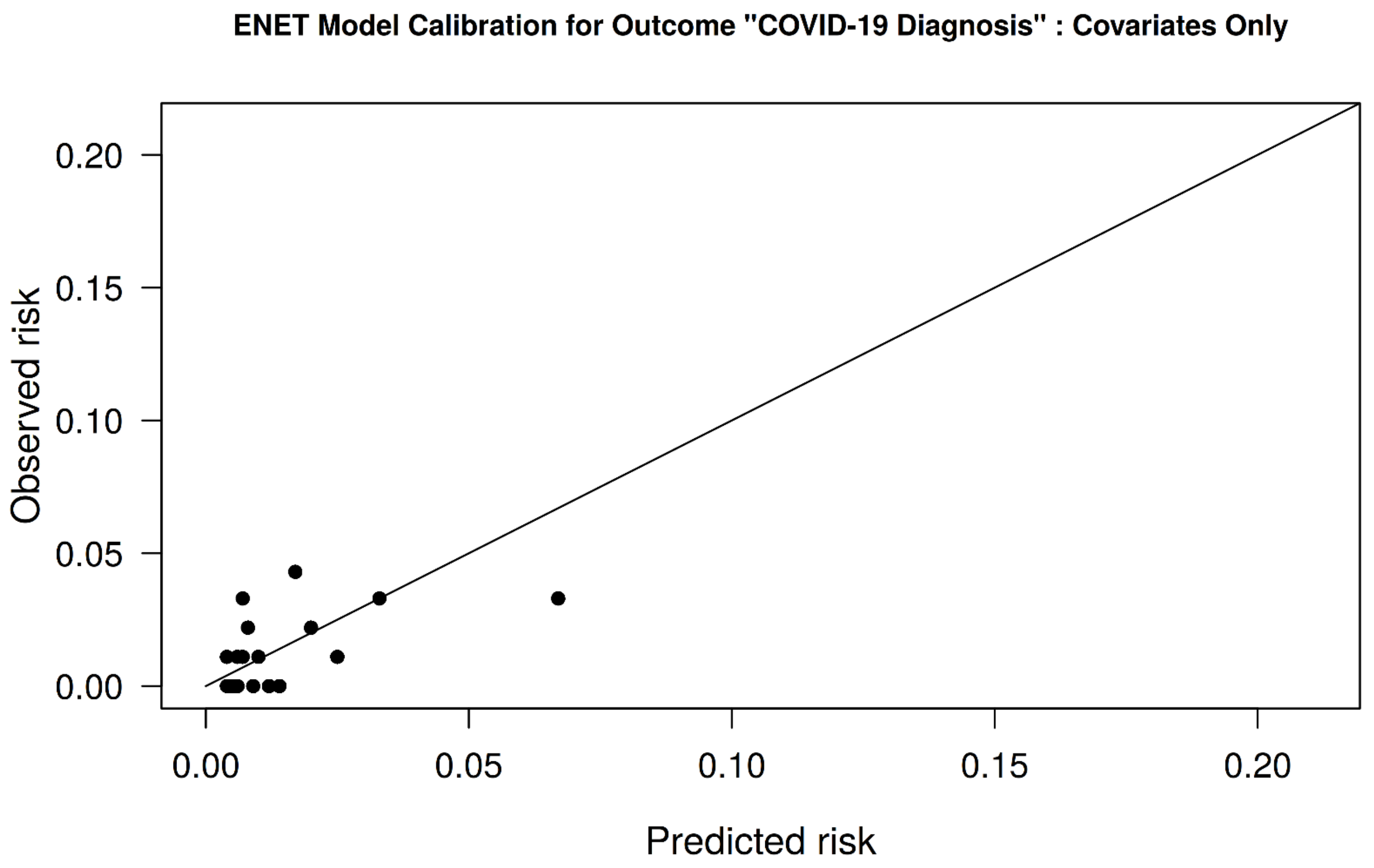

Subjects were split into 20 groups based on their predicted risk, and the observed proportion of COVID-19 tested subject in those groups was plotted against the expected proportion as per the model. The model used for this plot was from the first random 70/30 train-test split out of 100 total.

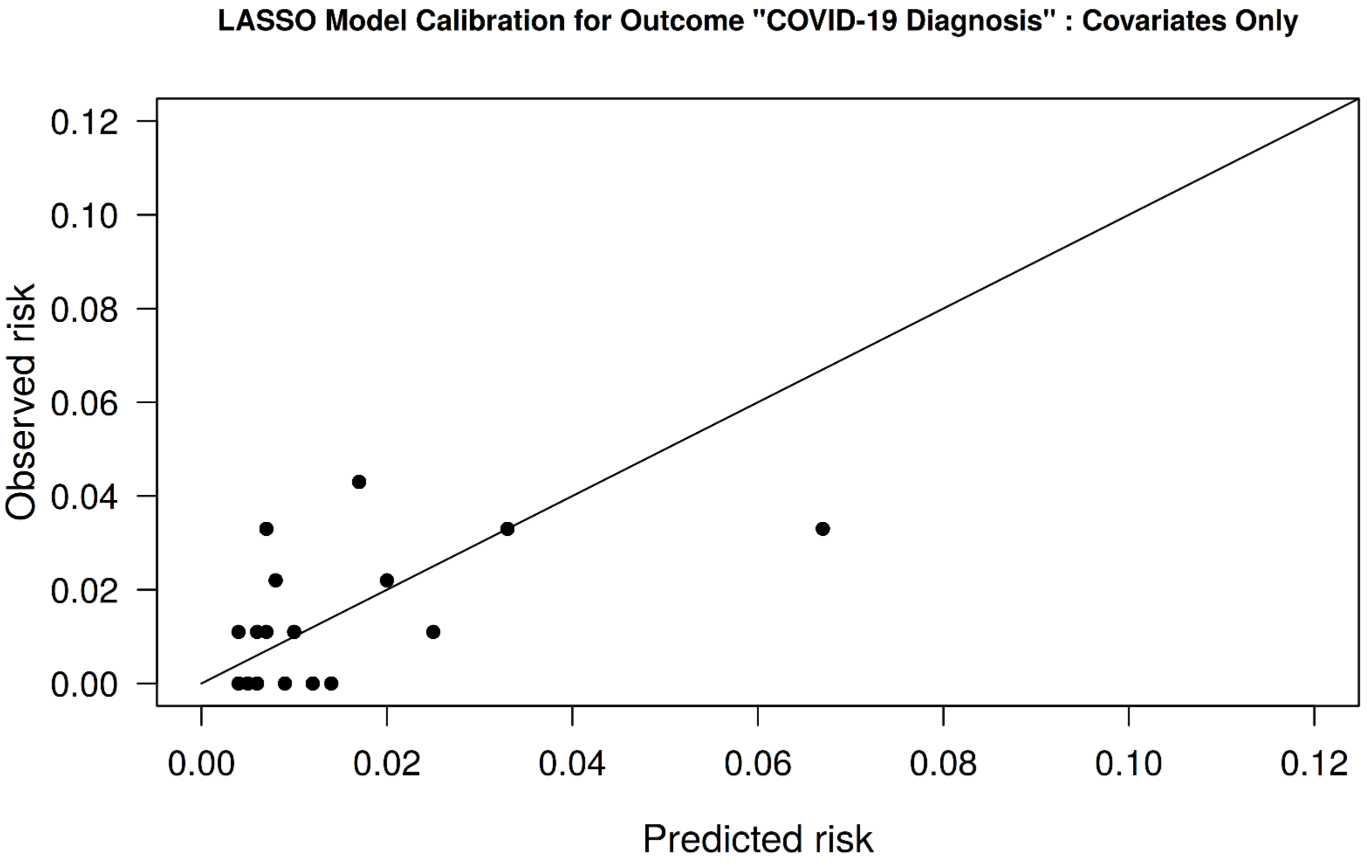

Subjects were split into 20 groups based on their predicted risk, and the observed proportion of COVID-19 tested subject in those groups was plotted against the expected proportion as per the model. The model used for this plot was from the first random 70/30 train-test split out of 100 total.

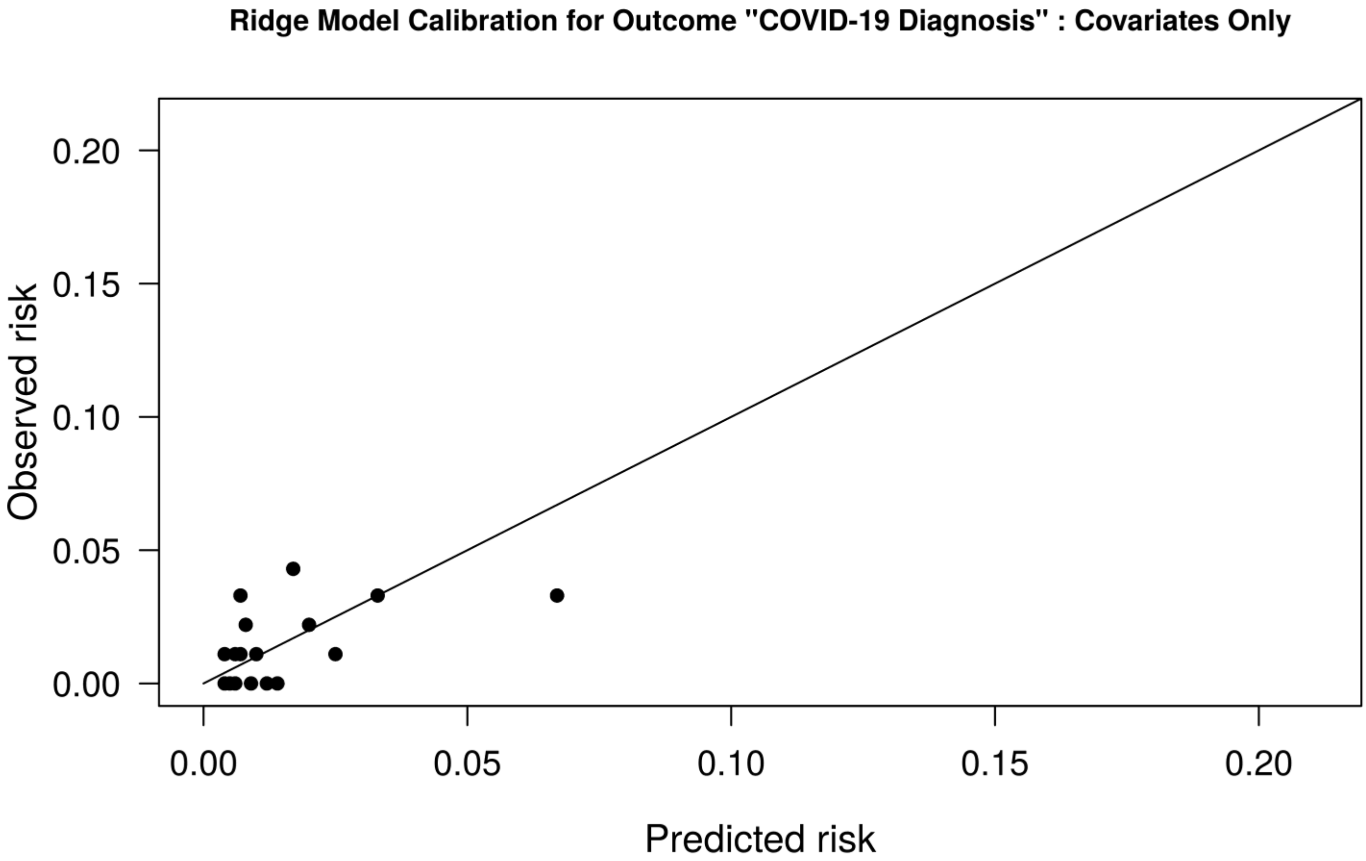

Subjects were split into 20 groups based on their predicted risk, and the observed proportion of COVID-19 tested subject in those groups was plotted against the expected proportion as per the model. The model used for this plot was from the first random 70/30 train-test split out of 100 total.

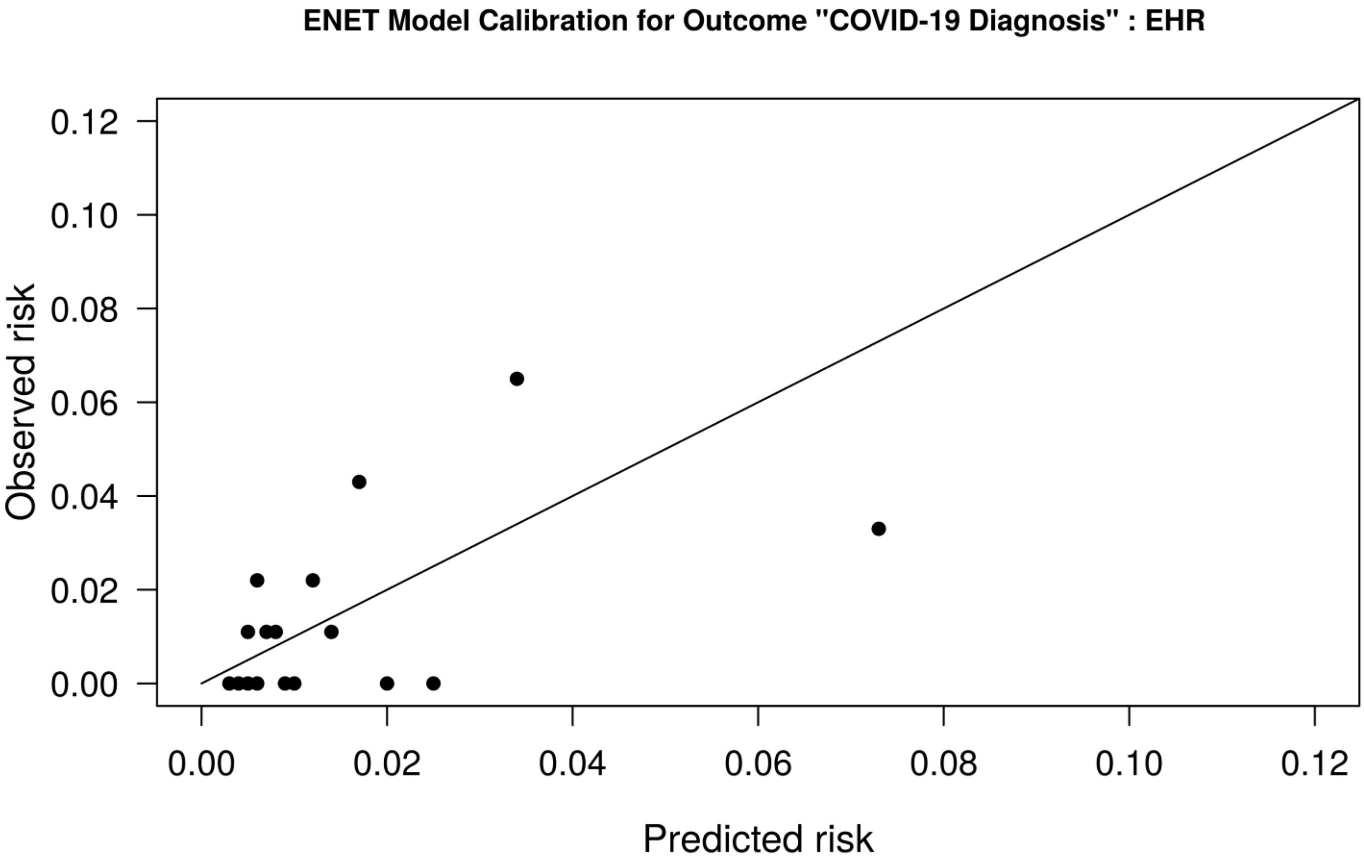

Subjects were split into 20 groups based on their predicted risk, and the observed proportion of COVID-19 tested subject in those groups was plotted against the expected proportion as per the model. The model used for this plot was from the first random 70/30 train-test split out of 100 total.

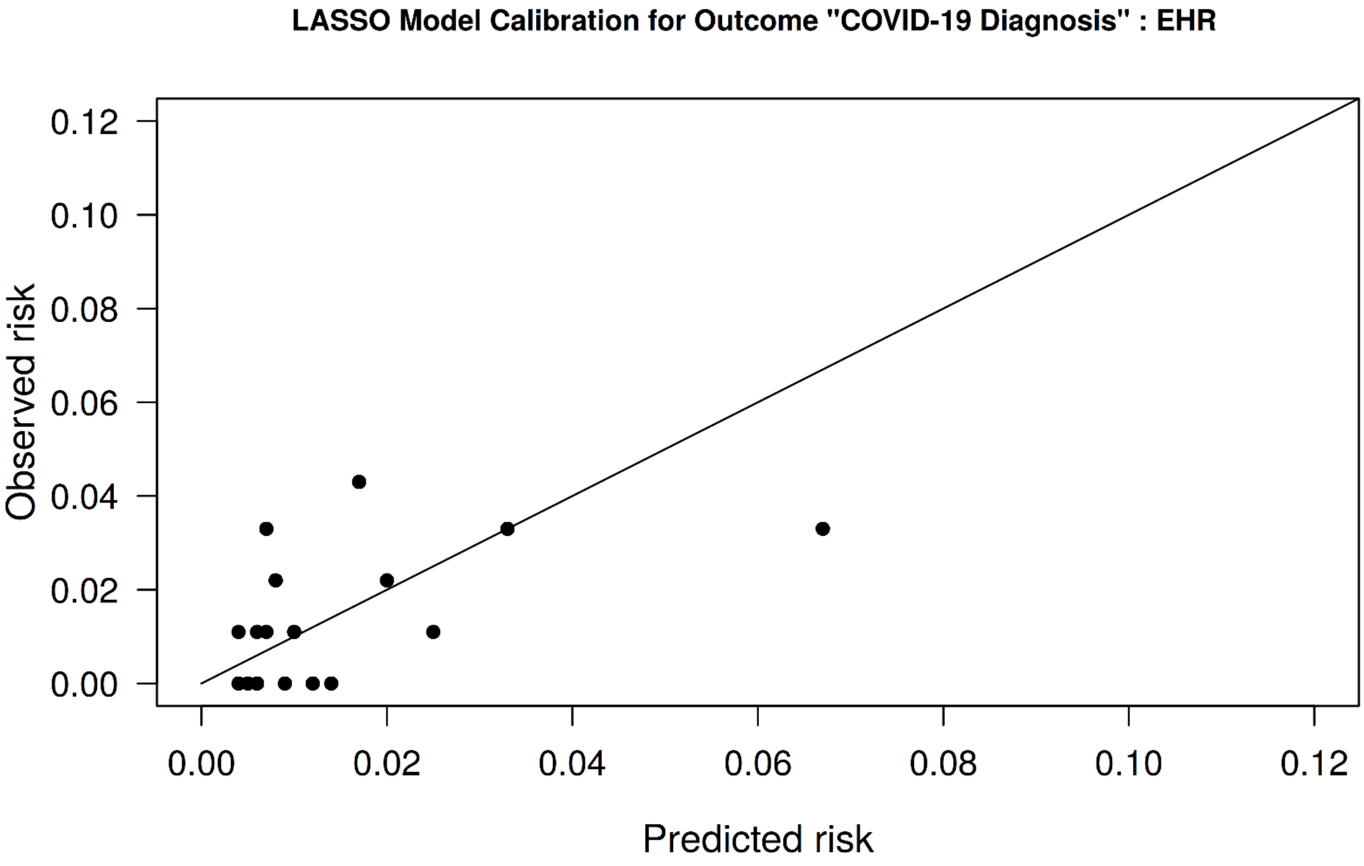

Subjects were split into 20 groups based on their predicted risk, and the observed proportion of COVID-19 tested subject in those groups was plotted against the expected proportion as per the model. The model used for this plot was from the first random 70/30 train-test split out of 100 total.

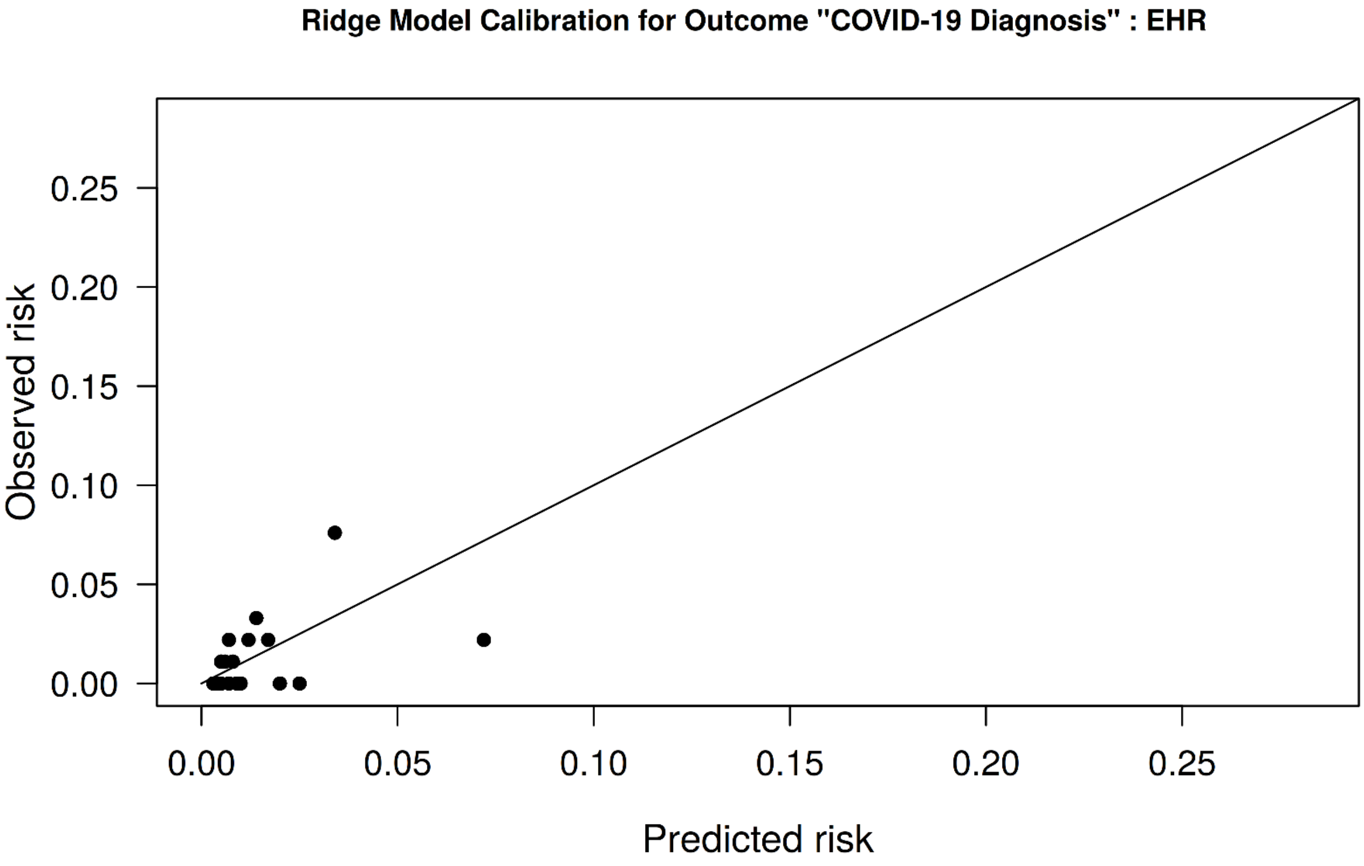

Subjects were split into 20 groups based on their predicted risk, and the observed proportion of COVID-19 tested subject in those groups was plotted against the expected proportion as per the model. The model used for this plot was from the first random 70/30 train-test split out of 100 total.

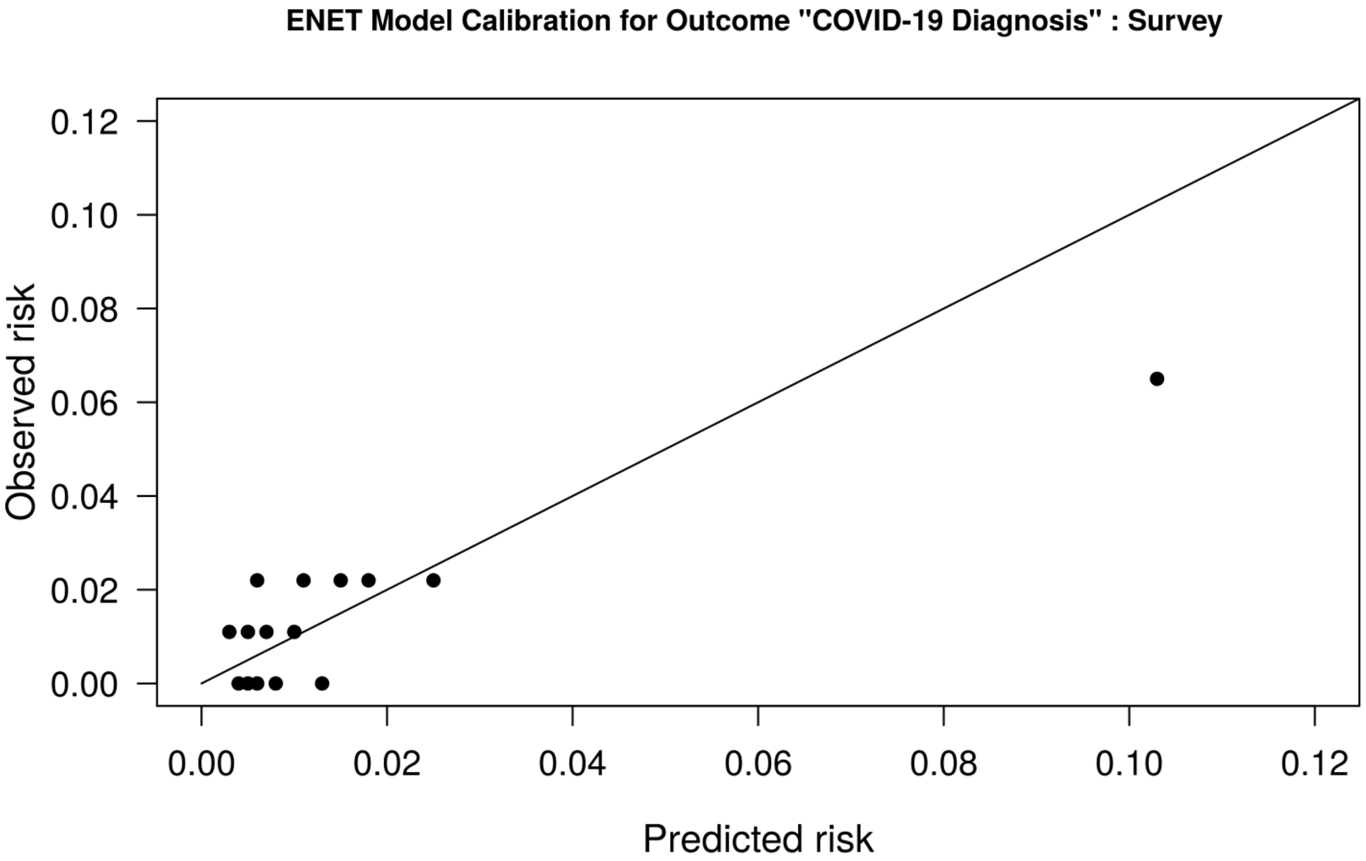

Subjects were split into 20 groups based on their predicted risk, and the observed proportion of COVID-19 tested subject in those groups was plotted against the expected proportion as per the model. The model used for this plot was from the first random 70/30 train-test split out of 100 total.

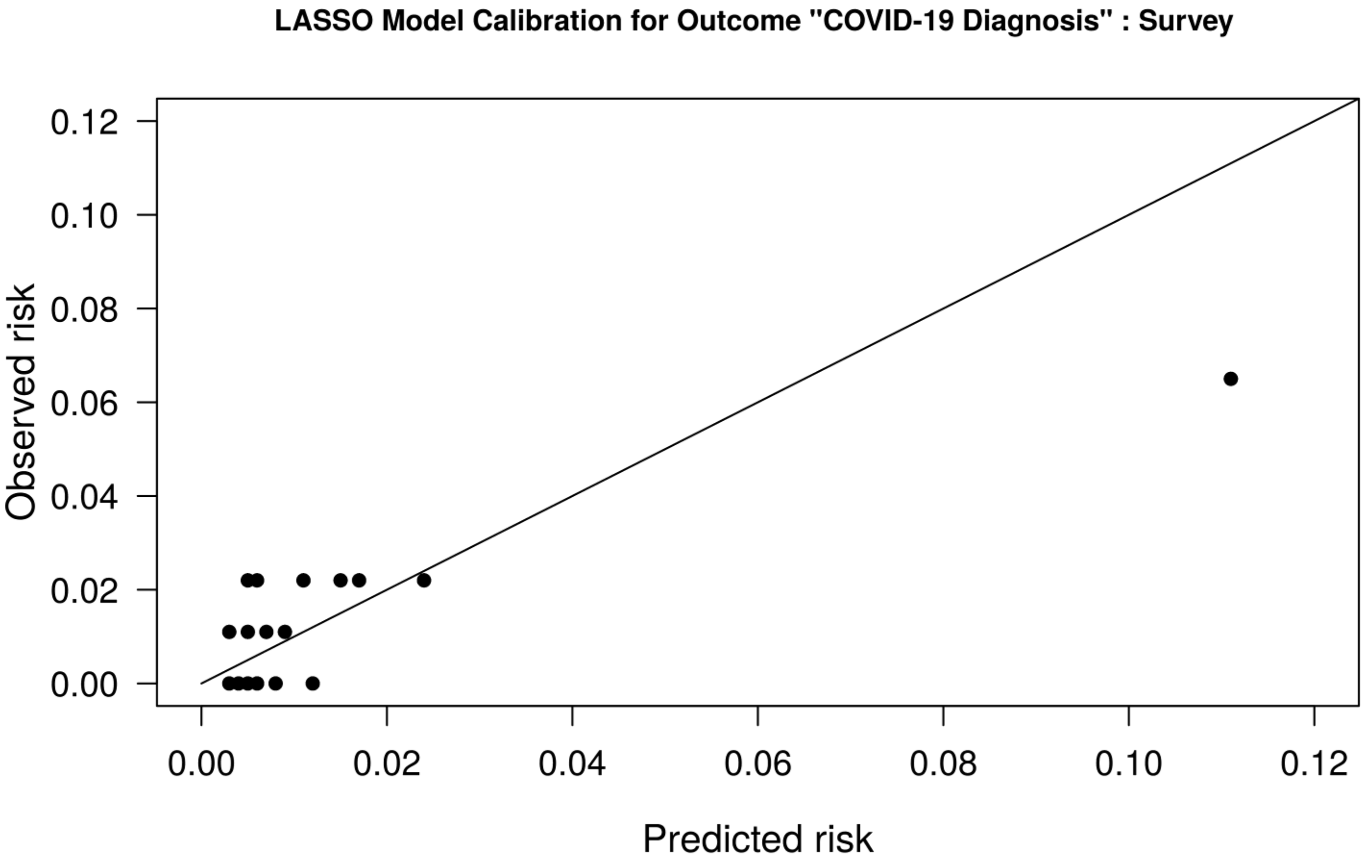

Subjects were split into 20 groups based on their predicted risk, and the observed proportion of COVID-19 tested subject in those groups was plotted against the expected proportion as per the model. The model used for this plot was from the first random 70/30 train-test split out of 100 total.

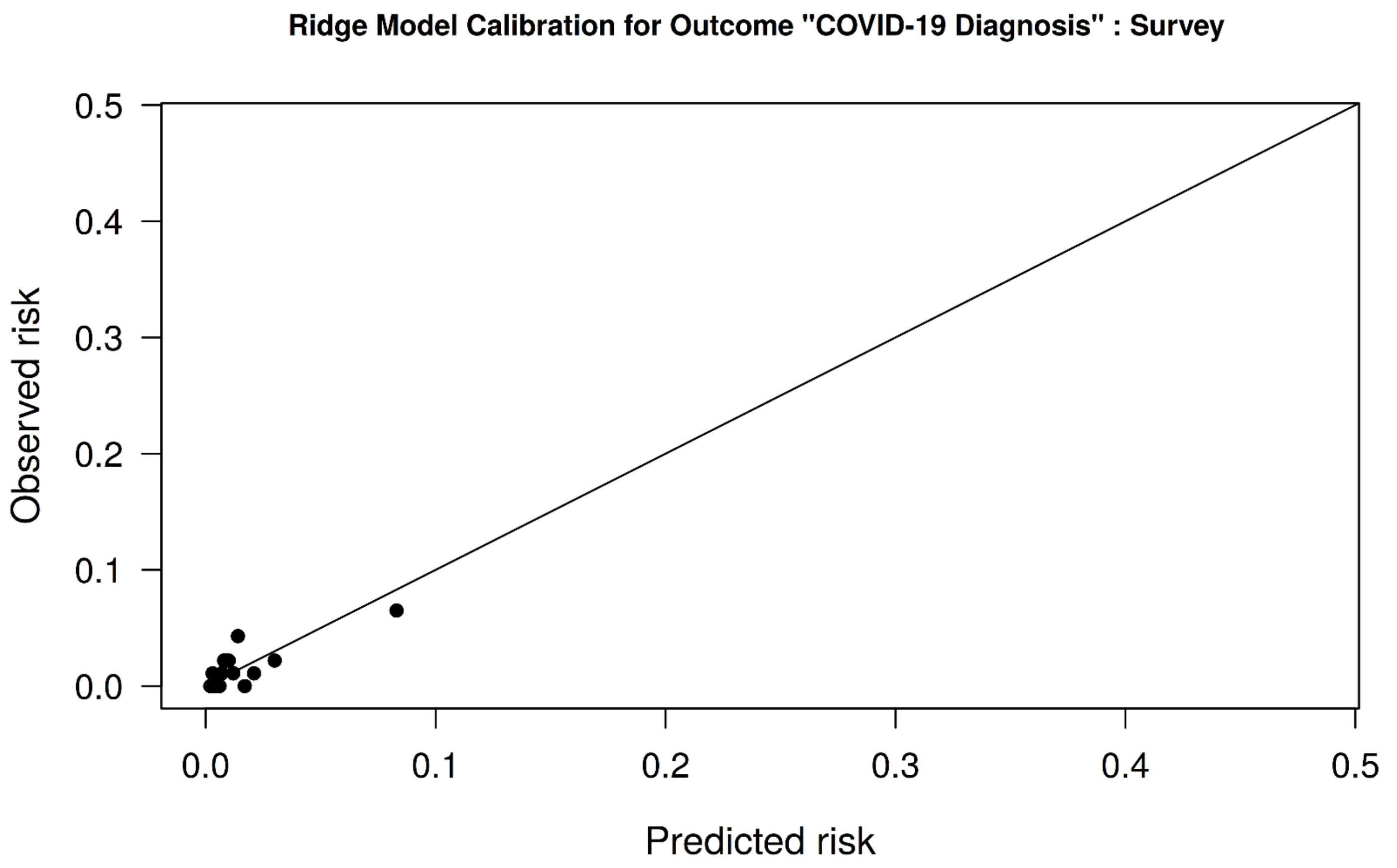

Subjects were split into 20 groups based on their predicted risk, and the observed proportion of COVID-19 tested subject in those groups was plotted against the expected proportion as per the model. The model used for this plot was from the first random 70/30 train-test split out of 100 total.

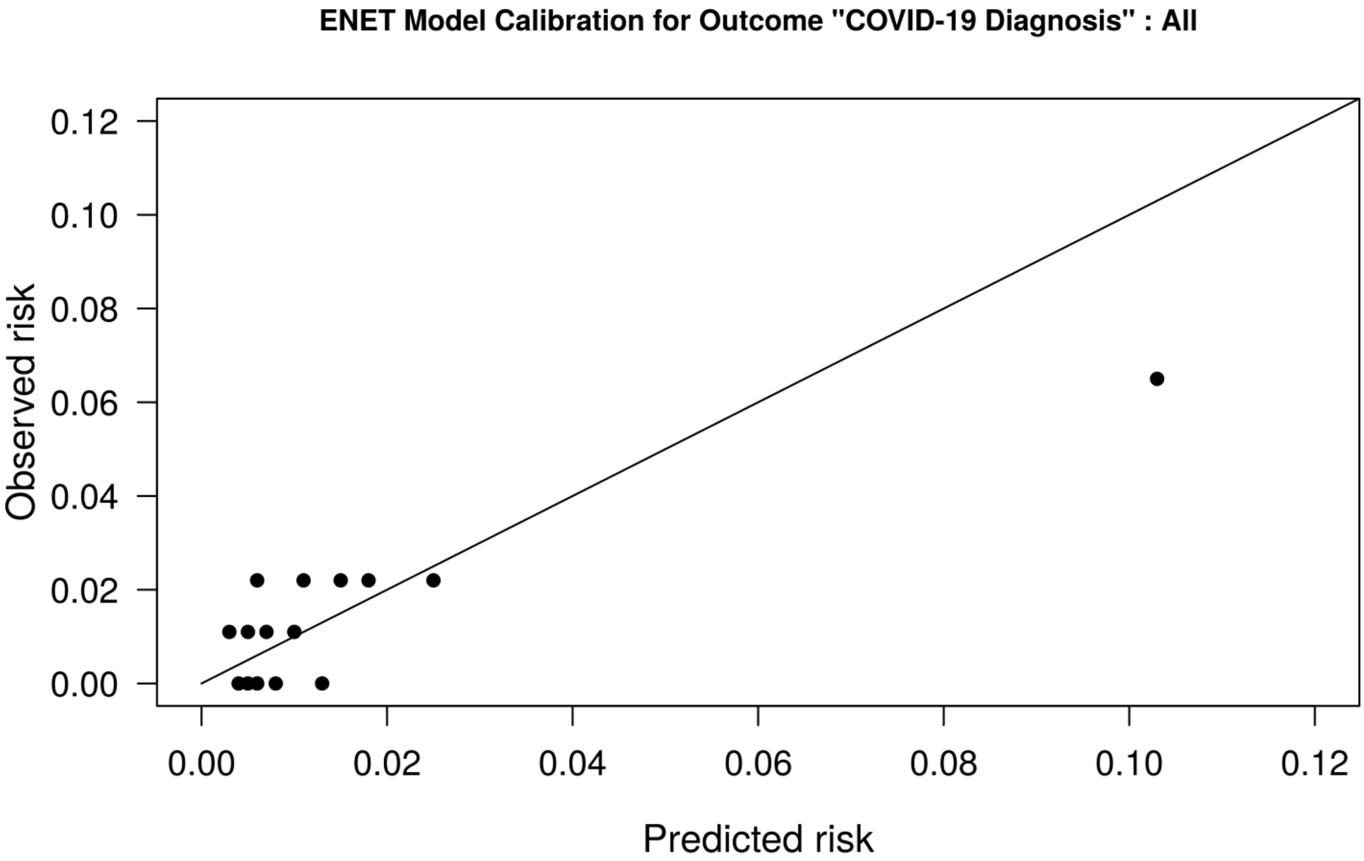

Subjects were split into 20 groups based on their predicted risk, and the observed proportion of COVID-19 tested subject in those groups was plotted against the expected proportion as per the model. The model used for this plot was from the first random 70/30 train-test split out of 100 total.

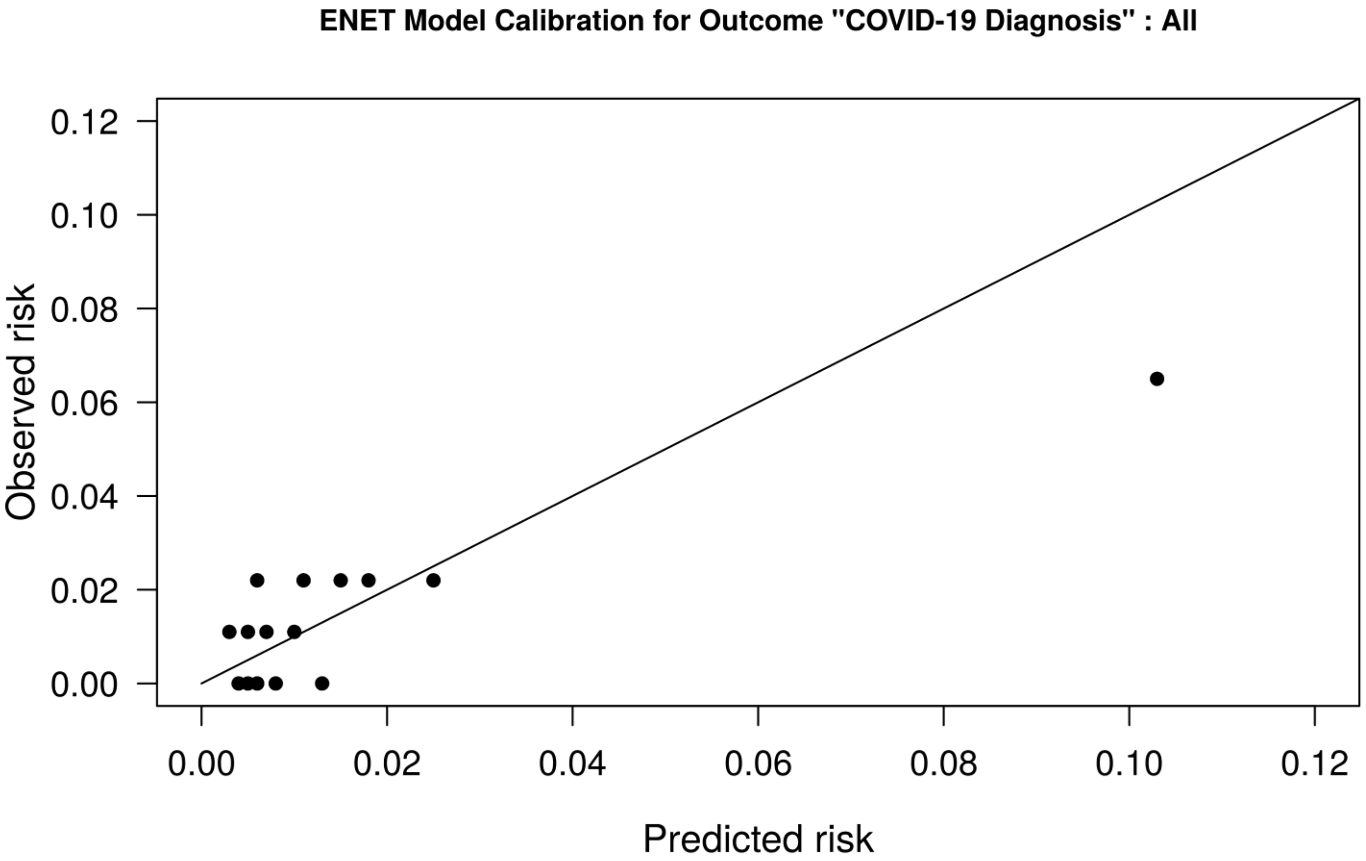

Subjects were split into 20 groups based on their predicted risk, and the observed proportion of COVID-19 tested subject in those groups was plotted against the expected proportion as per the model. The model used for this plot was from the first random 70/30 train-test split out of 100 total.

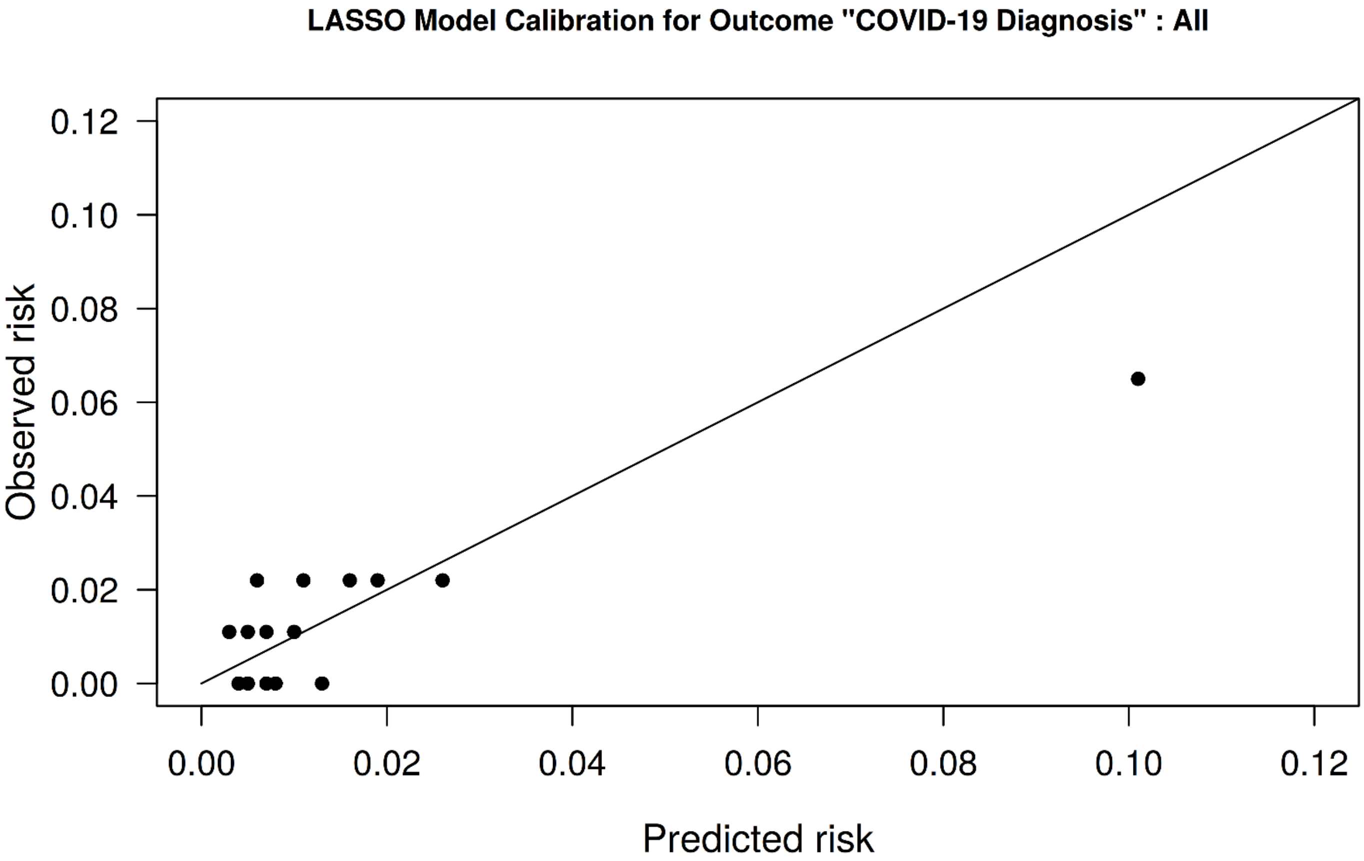

Subjects were split into 20 groups based on their predicted risk, and the observed proportion of COVID-19 tested subject in those groups was plotted against the expected proportion as per the model. The model used for this plot was from the first random 70/30 train-test split out of 100 total.

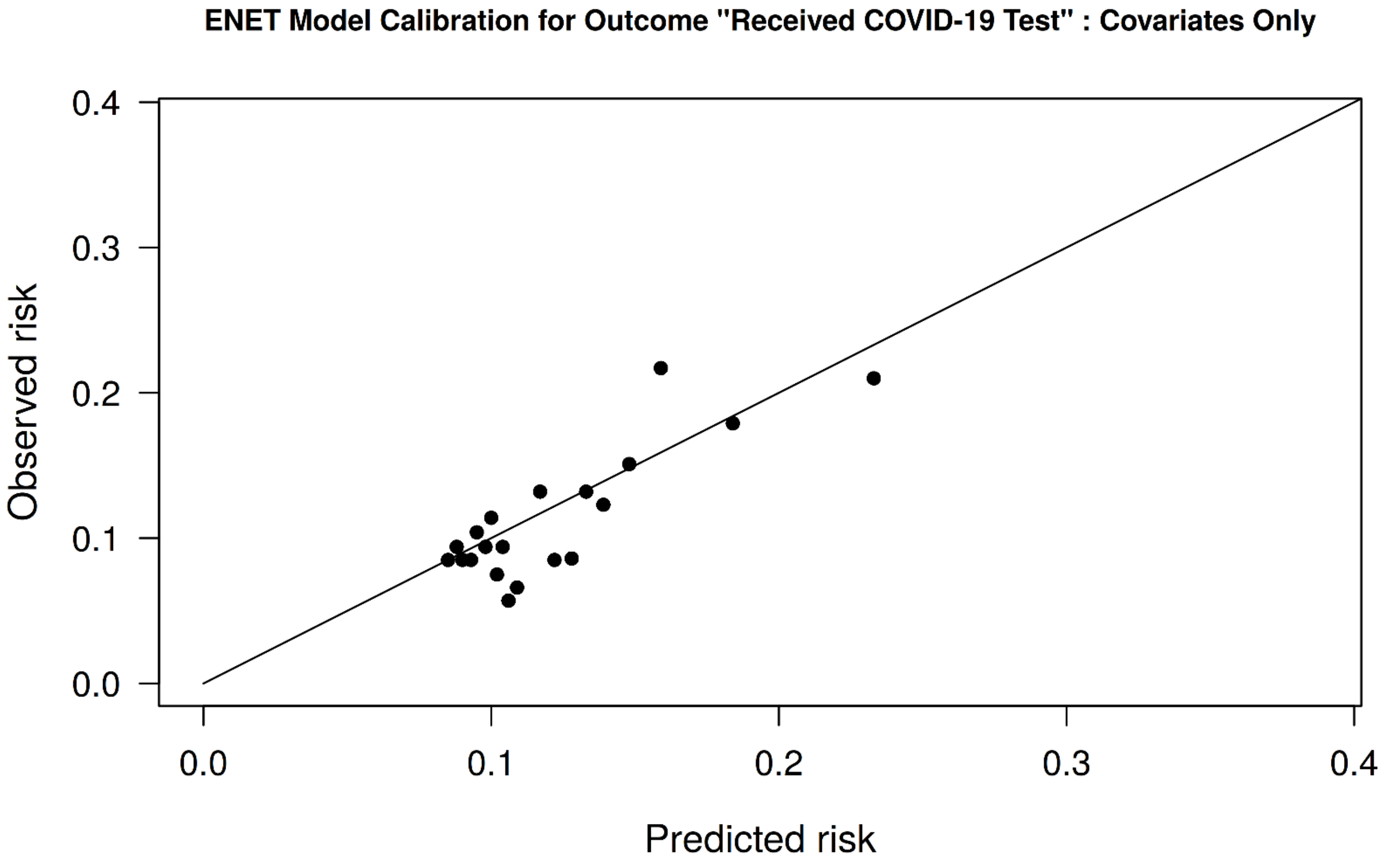

Subjects were split into 20 groups based on their predicted risk, and the observed proportion of COVID-19 tested subject in those groups was plotted against the expected proportion as per the model. The model used for this plot was from the first random 70/30 train-test split out of 100 total.

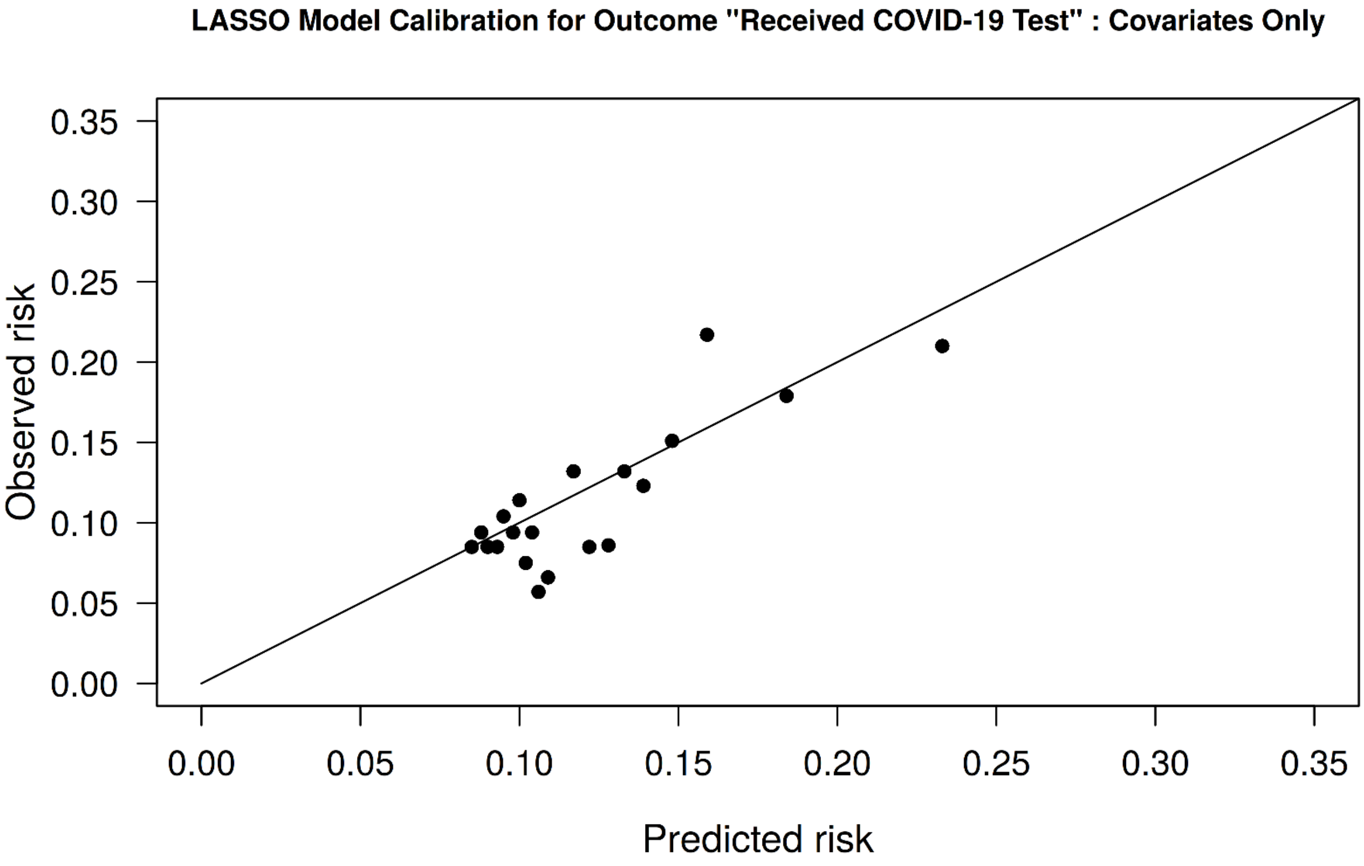

Subjects were split into 20 groups based on their predicted risk, and the observed proportion of COVID-19 tested subject in those groups was plotted against the expected proportion as per the model. The model used for this plot was from the first random 70/30 train-test split out of 100 total.

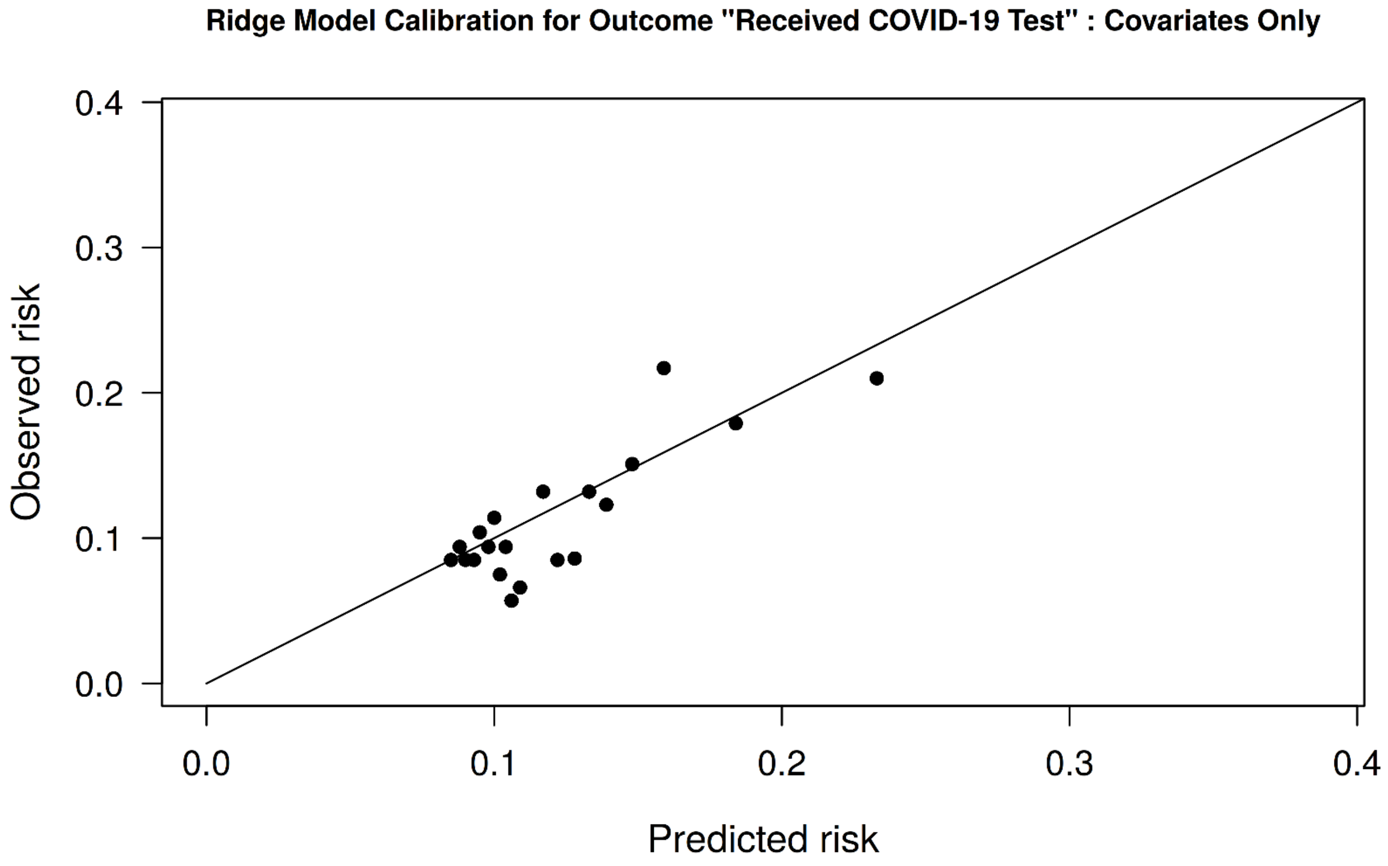

Subjects were split into 20 groups based on their predicted risk, and the observed proportion of COVID-19 tested subject in those groups was plotted against the expected proportion as per the model. The model used for this plot was from the first random 70/30 train-test split out of 100 total.

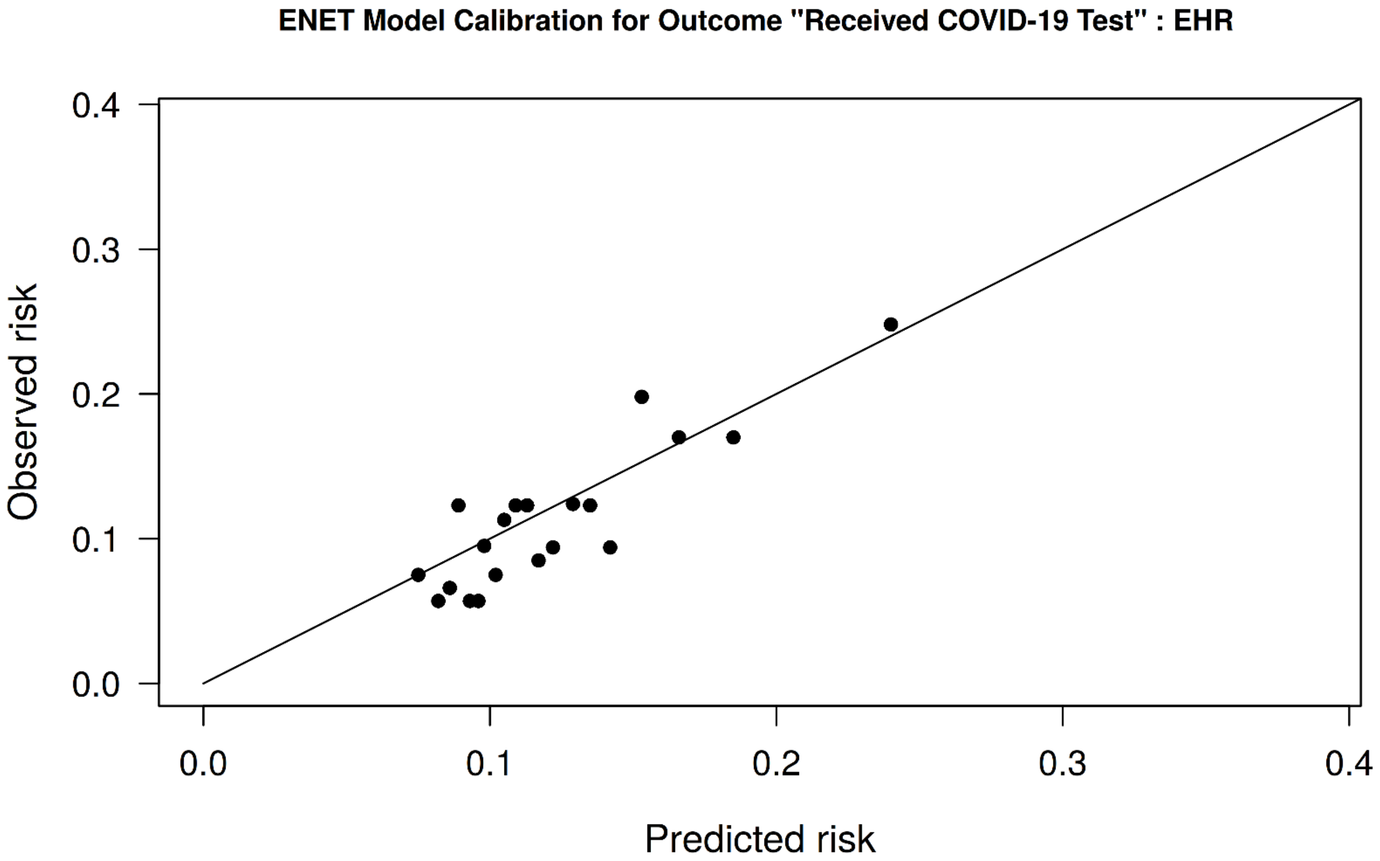

Subjects were split into 20 groups based on their predicted risk, and the observed proportion of COVID-19 tested subject in those groups was plotted against the expected proportion as per the model. The model used for this plot was from the first random 70/30 train-test split out of 100 total.

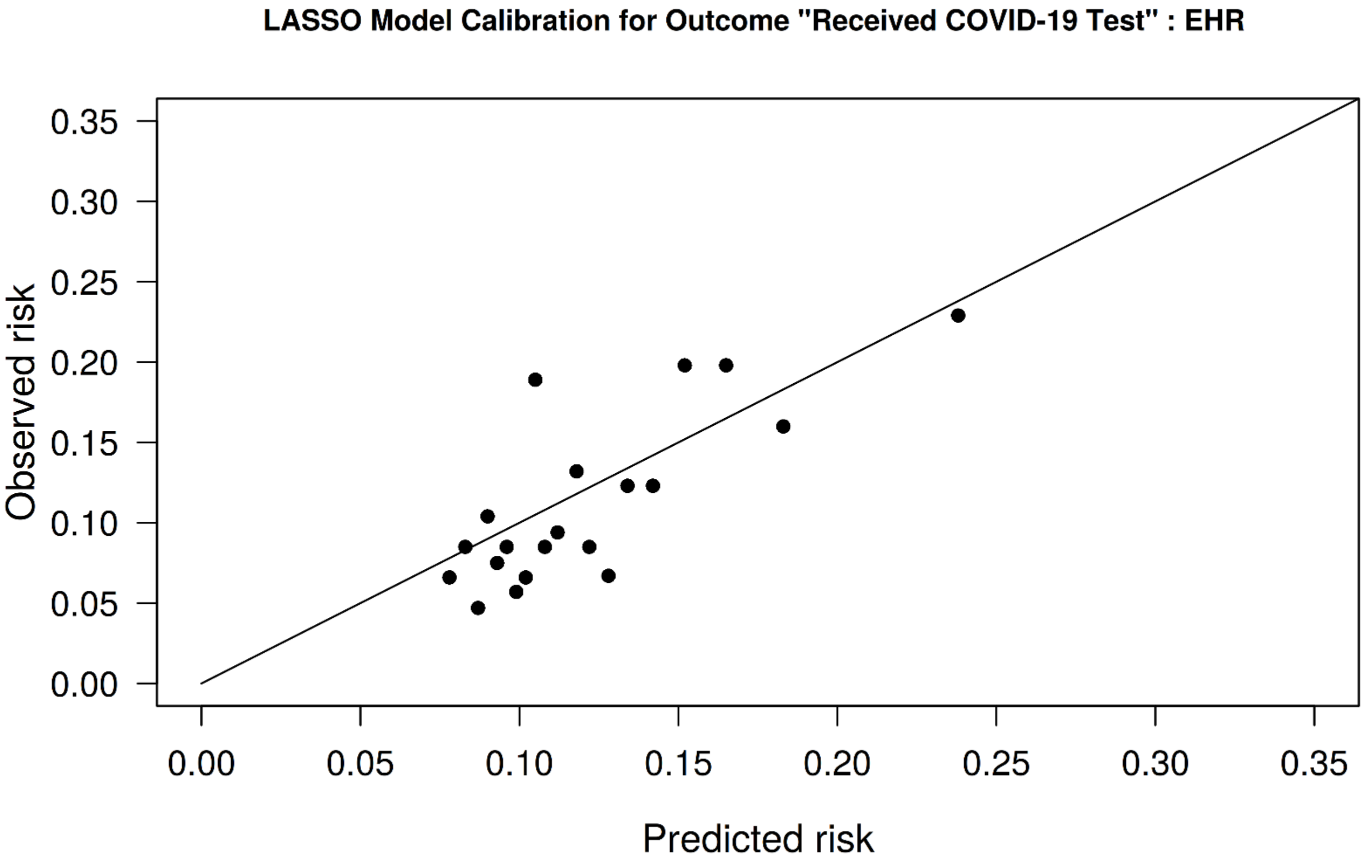

Subjects were split into 20 groups based on their predicted risk, and the observed proportion of COVID-19 tested subject in those groups was plotted against the expected proportion as per the model. The model used for this plot was from the first random 70/30 train-test split out of 100 total.

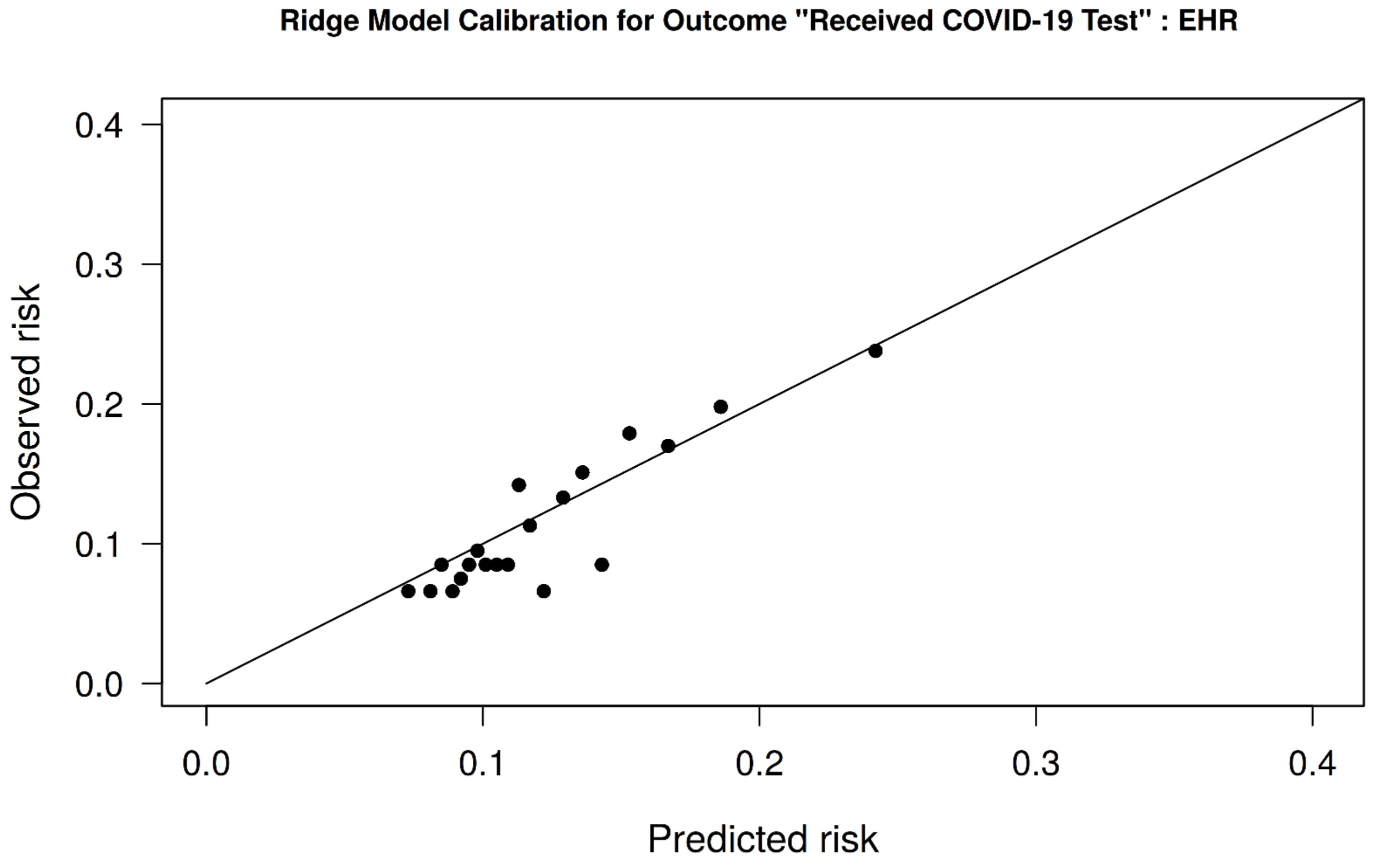

Subjects were split into 20 groups based on their predicted risk, and the observed proportion of COVID-19 tested subject in those groups was plotted against the expected proportion as per the model. The model used for this plot was from the first random 70/30 train-test split out of 100 total.

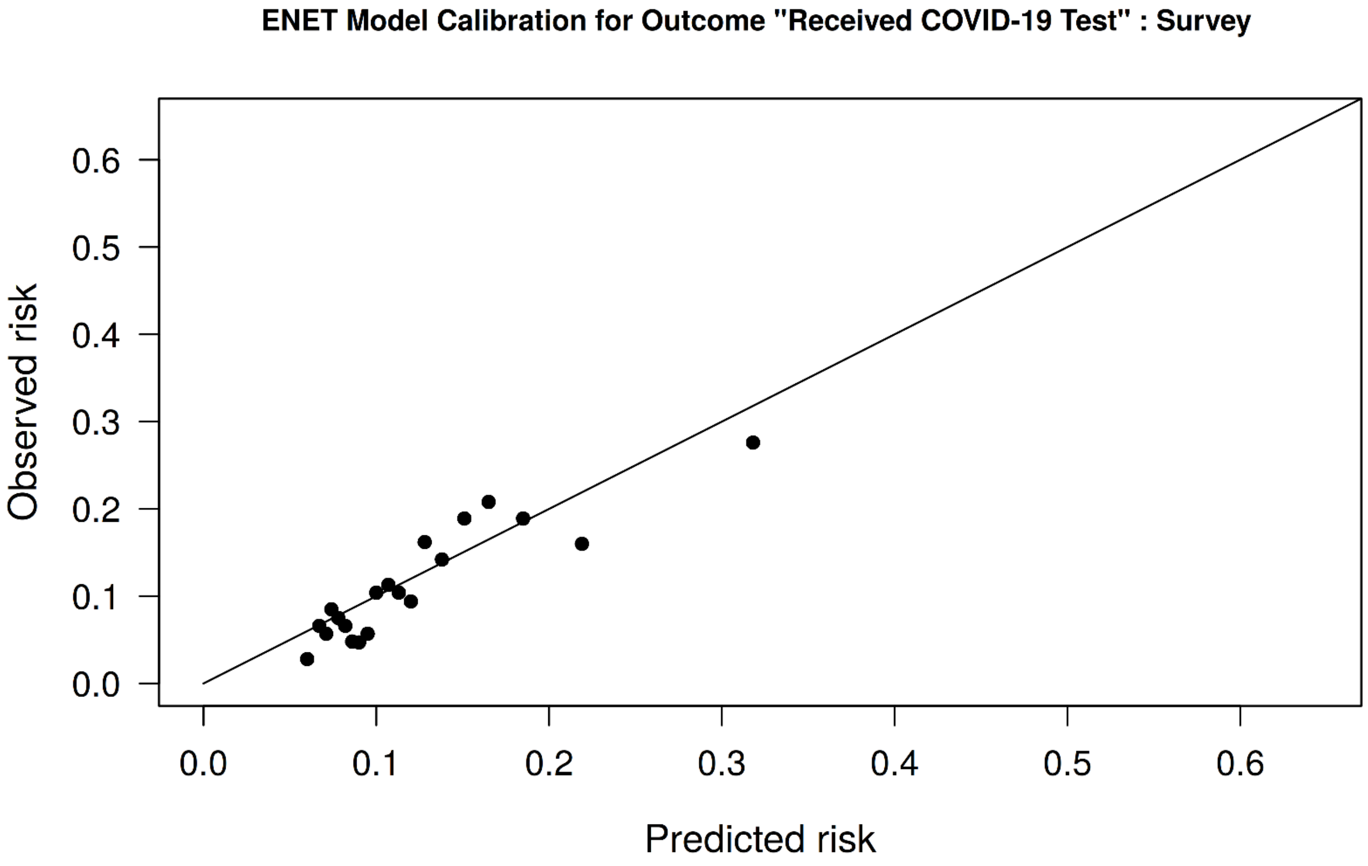

Subjects were split into 20 groups based on their predicted risk, and the observed proportion of COVID-19 tested subject in those groups was plotted against the expected proportion as per the model. The model used for this plot was from the first random 70/30 train-test split out of 100 total.

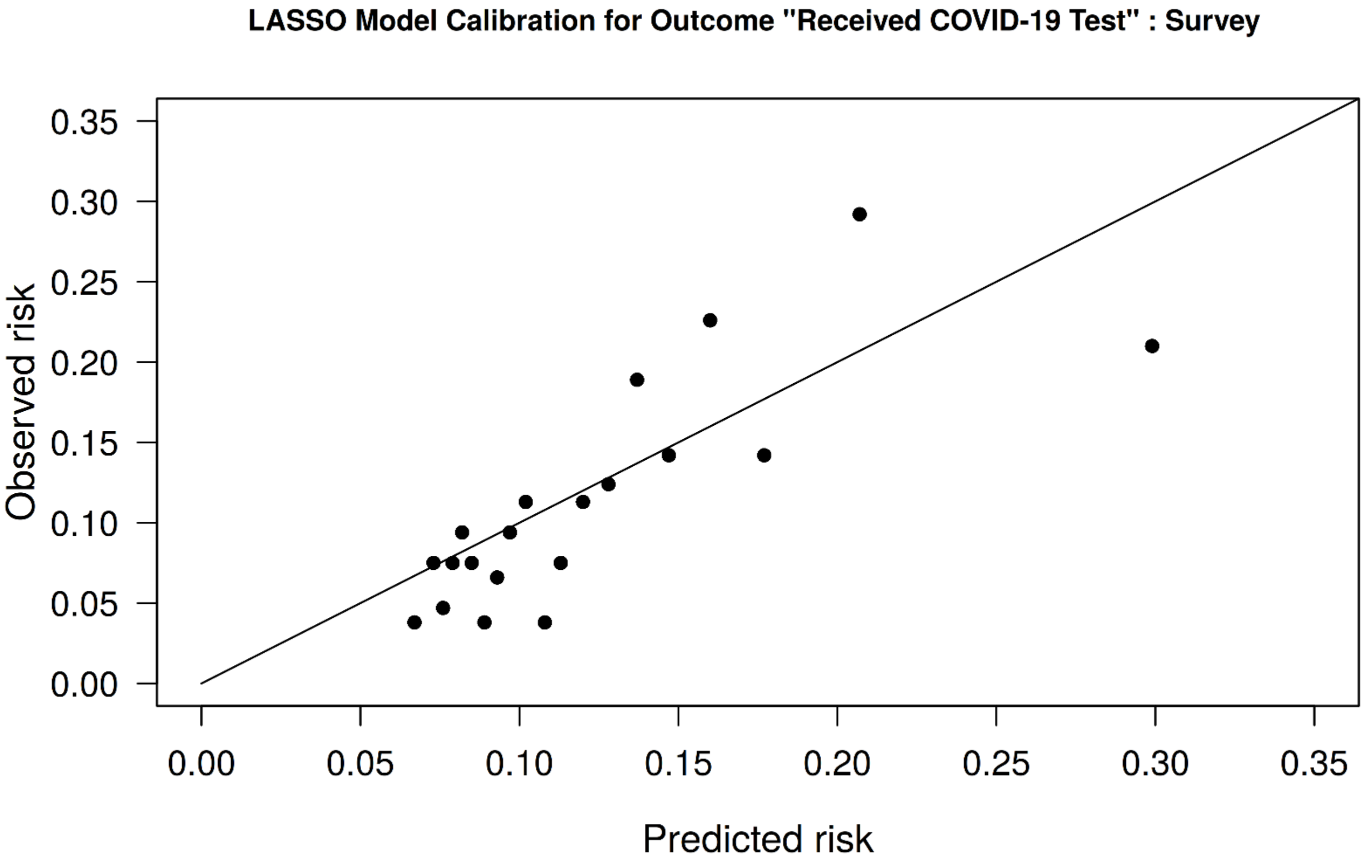

Subjects were split into 20 groups based on their predicted risk, and the observed proportion of COVID-19 tested subject in those groups was plotted against the expected proportion as per the model. The model used for this plot was from the first random 70/30 train-test split out of 100 total.

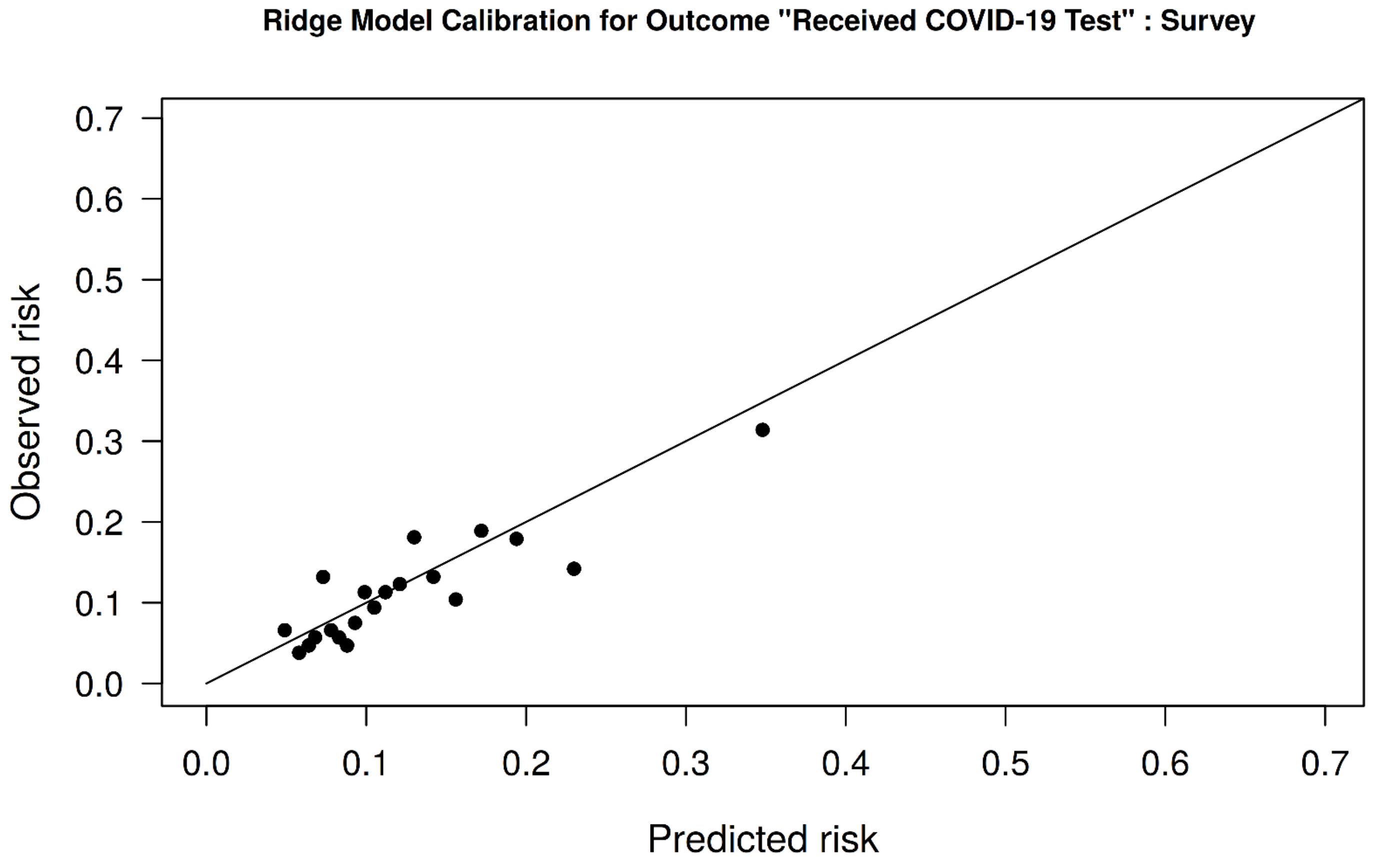

Subjects were split into 20 groups based on their predicted risk, and the observed proportion of COVID-19 tested subject in those groups was plotted against the expected proportion as per the model. The model used for this plot was from the first random 70/30 train-test split out of 100 total.

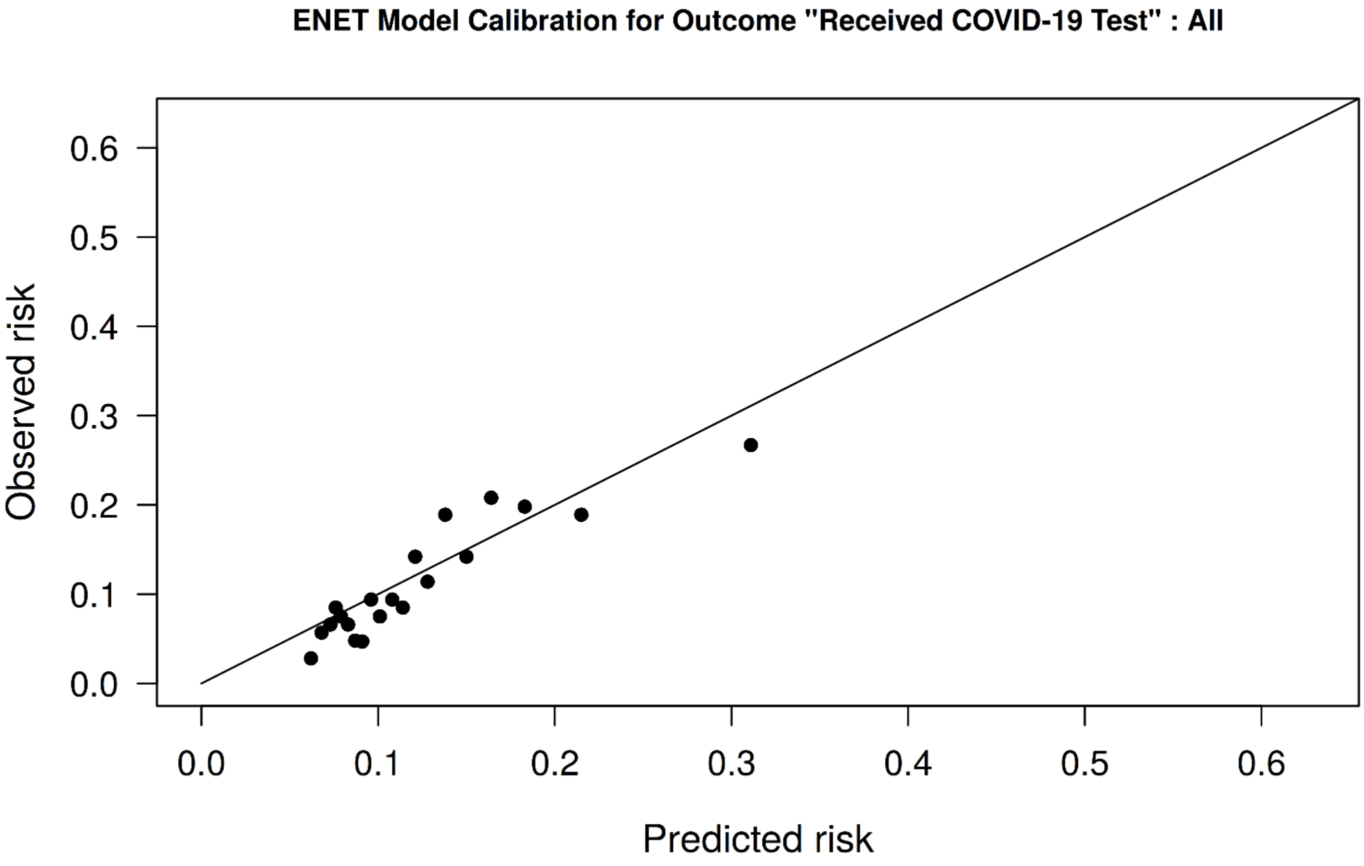

Subjects were split into 20 groups based on their predicted risk, and the observed proportion of COVID-19 tested subject in those groups was plotted against the expected proportion as per the model. The model used for this plot was from the first random 70/30 train-test split out of 100 total.

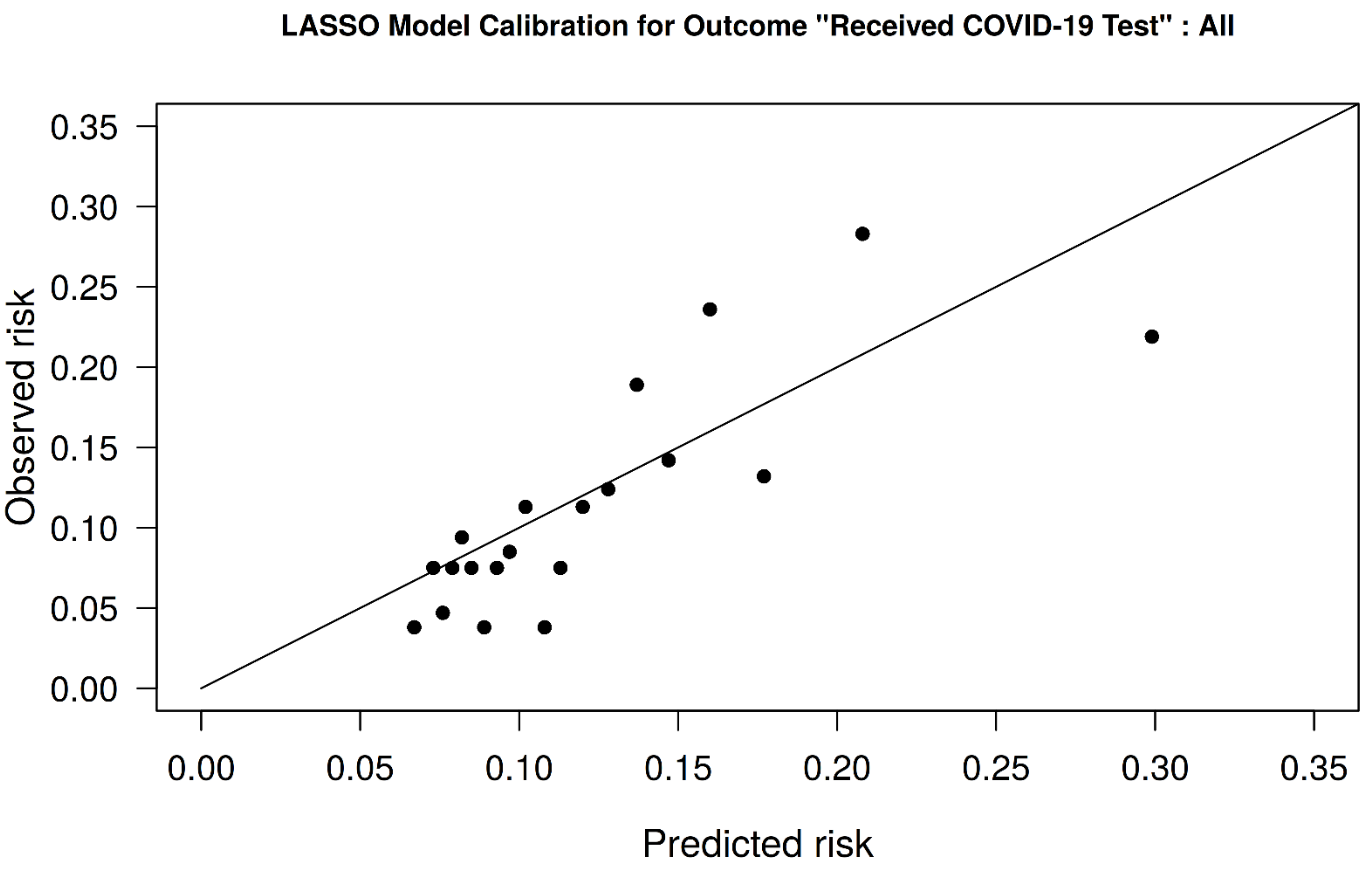

Subjects were split into 20 groups based on their predicted risk, and the observed proportion of COVID-19 tested subject in those groups was plotted against the expected proportion as per the model. The model used for this plot was from the first random 70/30 train-test split out of 100 total.

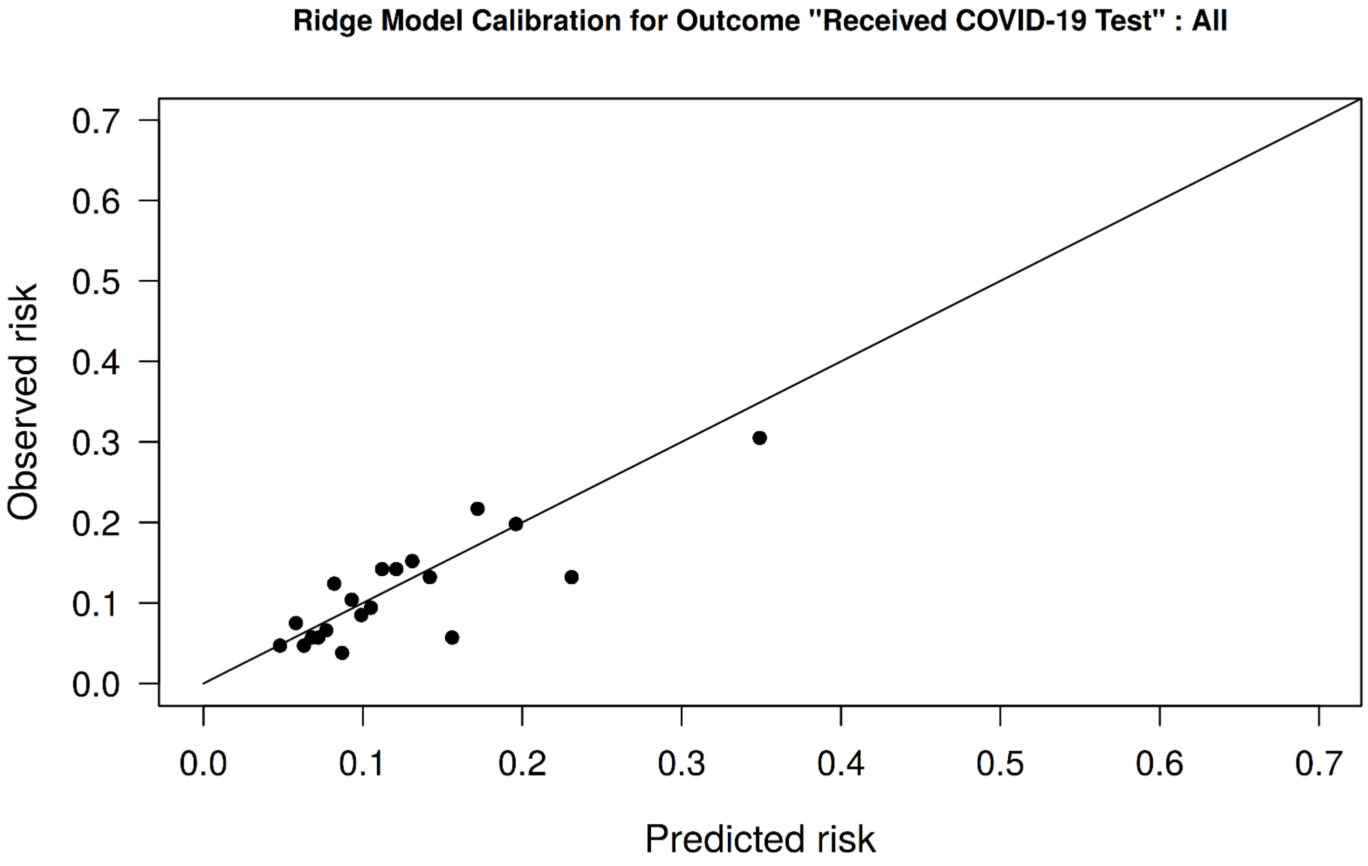

Subjects were split into 20 groups based on their predicted risk, and the observed proportion of COVID-19 tested subject in those groups was plotted against the expected proportion as per the model. The model used for this plot was from the first random 70/30 train-test split out of 100 total.

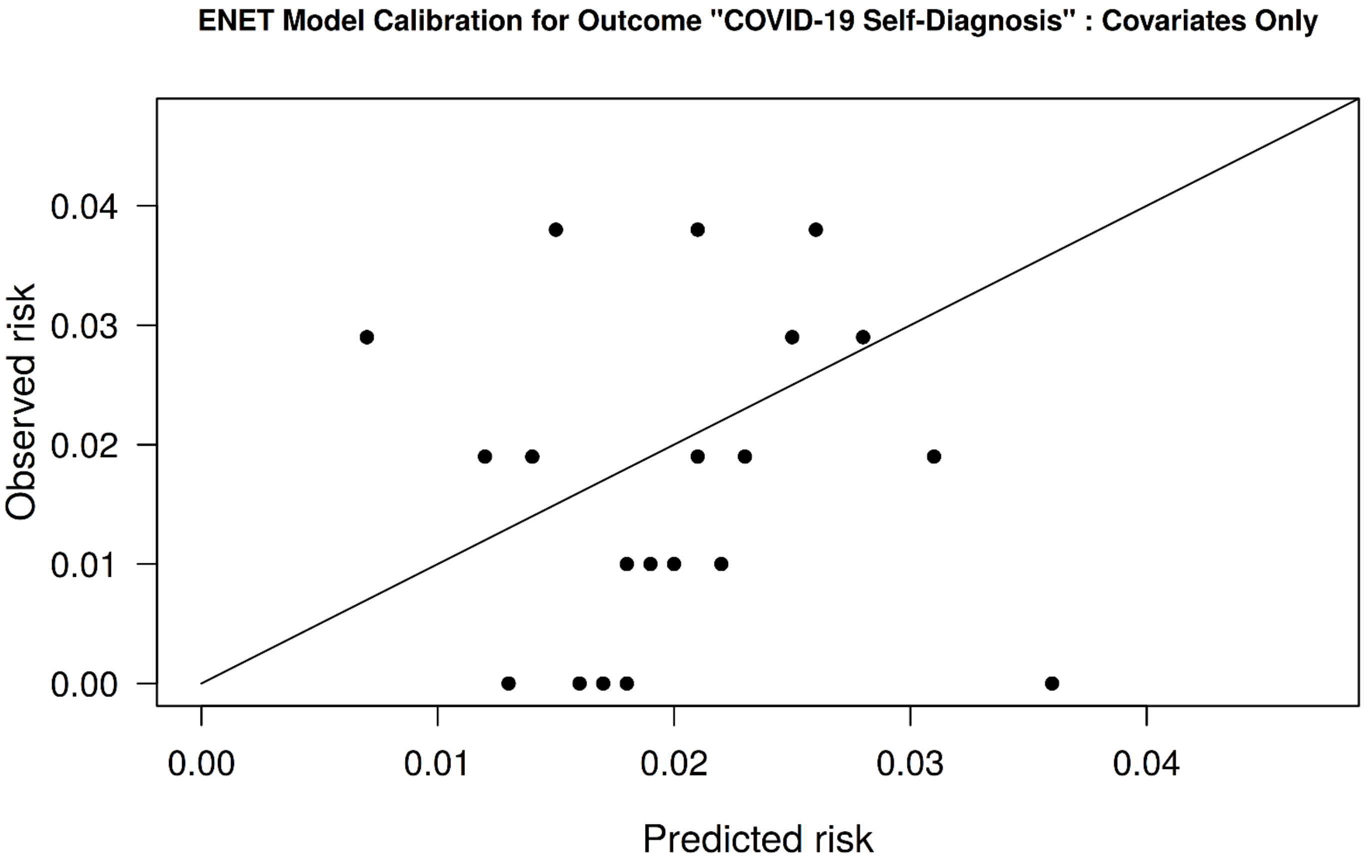

Subjects were split into 20 groups based on their predicted risk, and the observed proportion of COVID-19 tested subject in those groups was plotted against the expected proportion as per the model. The model used for this plot was from the first random 70/30 train-test split out of 100 total.

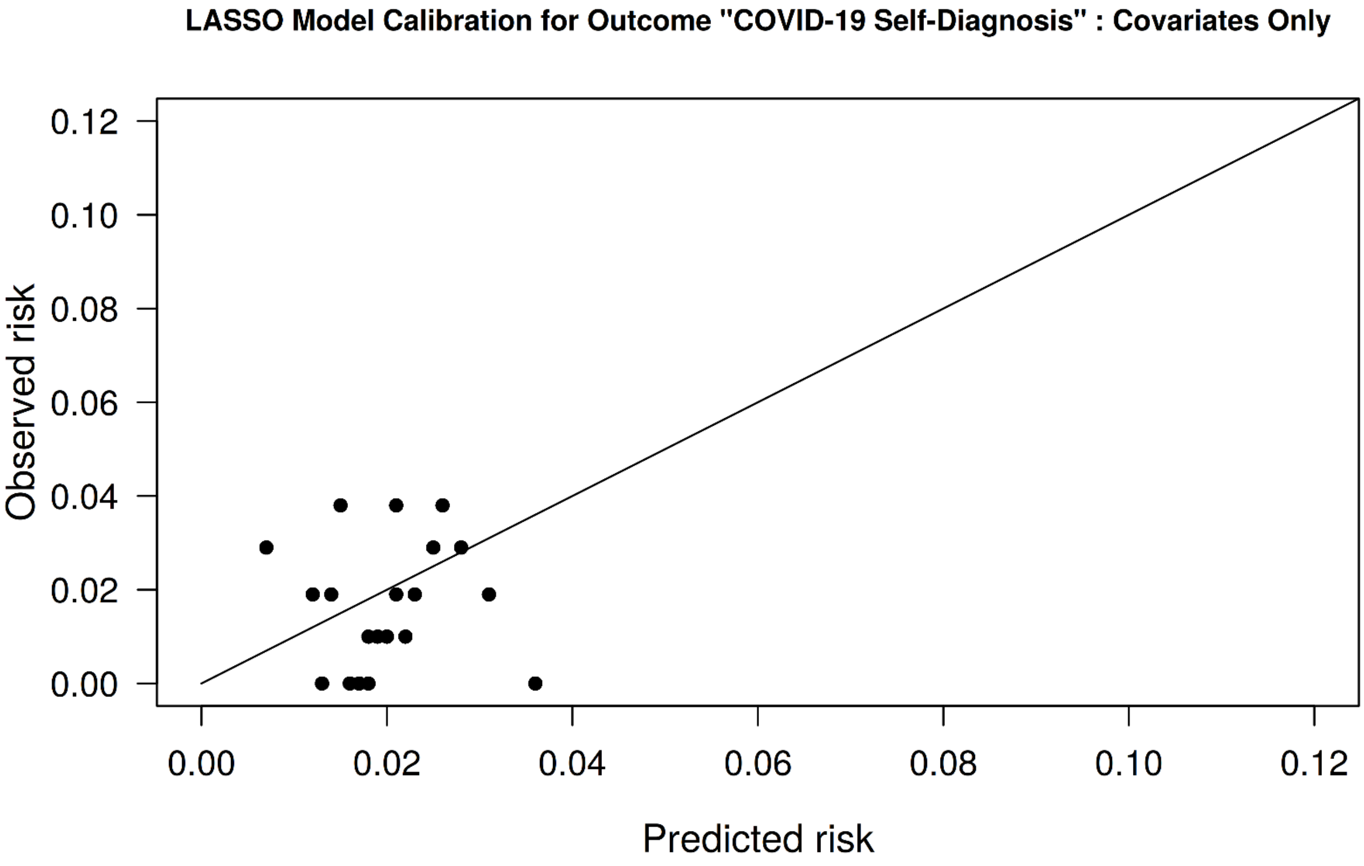

Subjects were split into 20 groups based on their predicted risk, and the observed proportion of COVID-19 tested subject in those groups was plotted against the expected proportion as per the model. The model used for this plot was from the first random 70/30 train-test split out of 100 total.

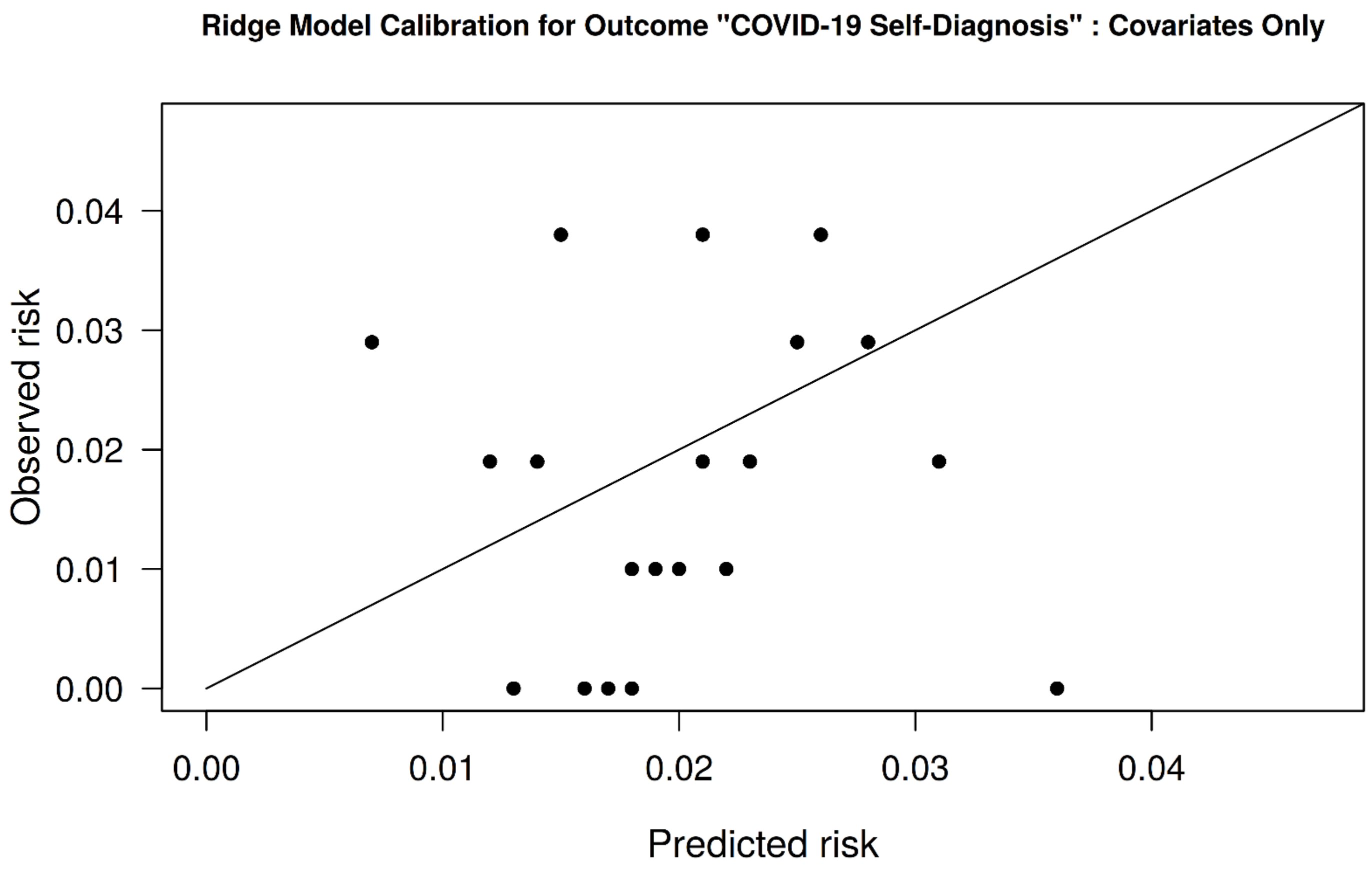

Subjects were split into 20 groups based on their predicted risk, and the observed proportion of COVID-19 tested subject in those groups was plotted against the expected proportion as per the model. The model used for this plot was from the first random 70/30 train-test split out of 100 total.

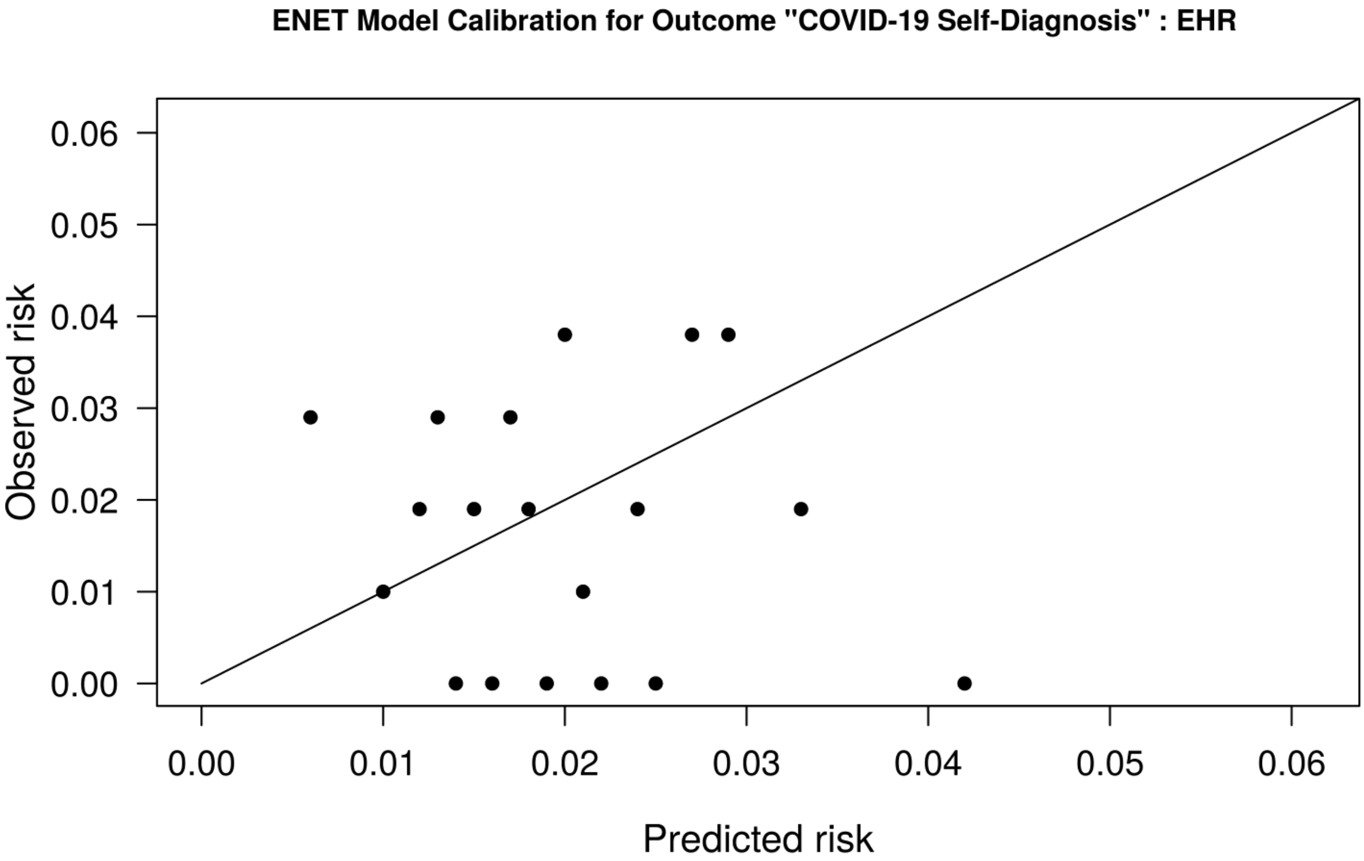

Subjects were split into 20 groups based on their predicted risk, and the observed proportion of COVID-19 tested subject in those groups was plotted against the expected proportion as per the model. The model used for this plot was from the first random 70/30 train-test split out of 100 total.

S15 File (continued)

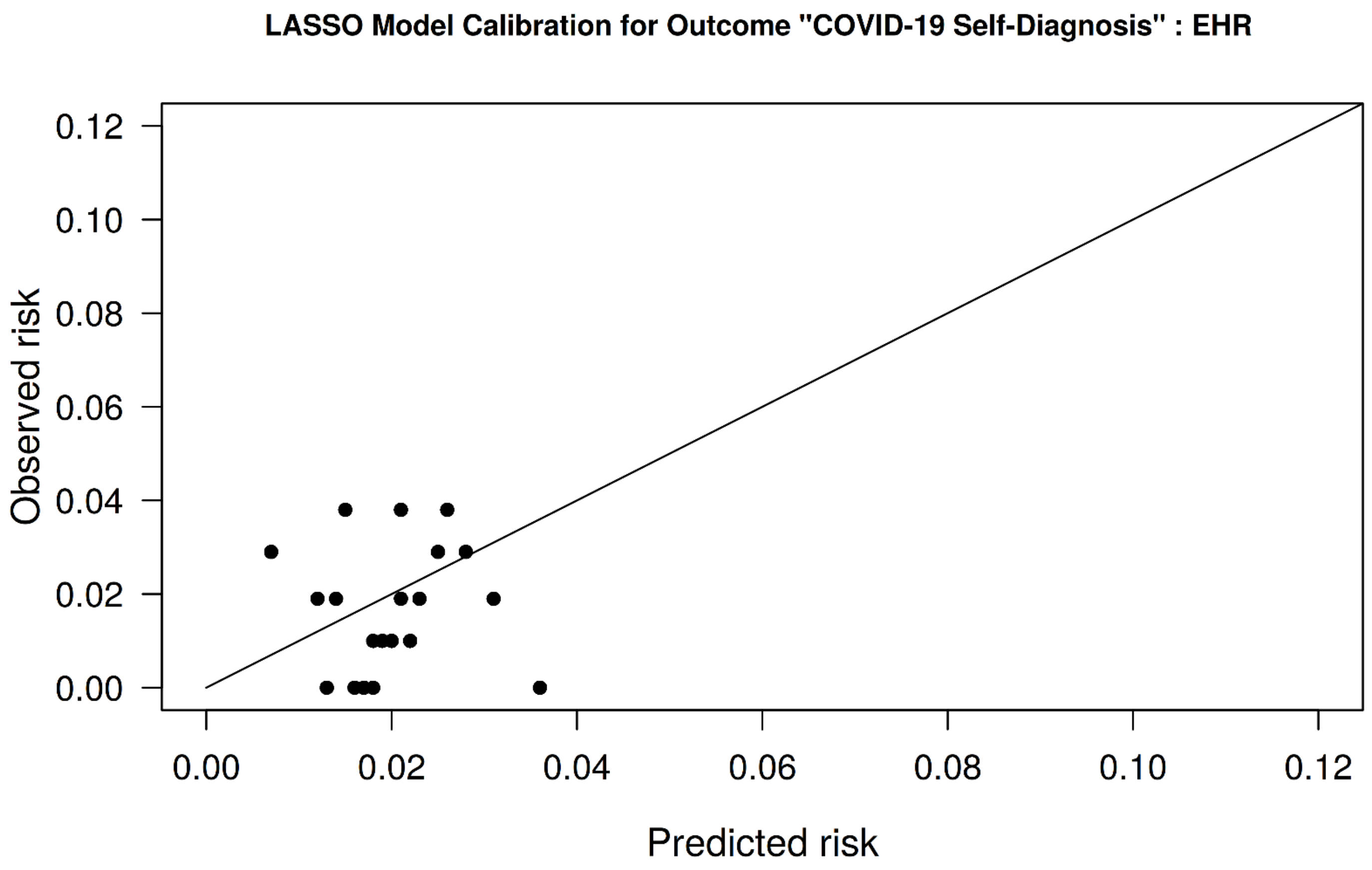

Subjects were split into 20 groups based on their predicted risk, and the observed proportion of COVID-19 tested subject in those groups was plotted against the expected proportion as per the model. The model used for this plot was from the first random 70/30 train-test split out of 100 total.

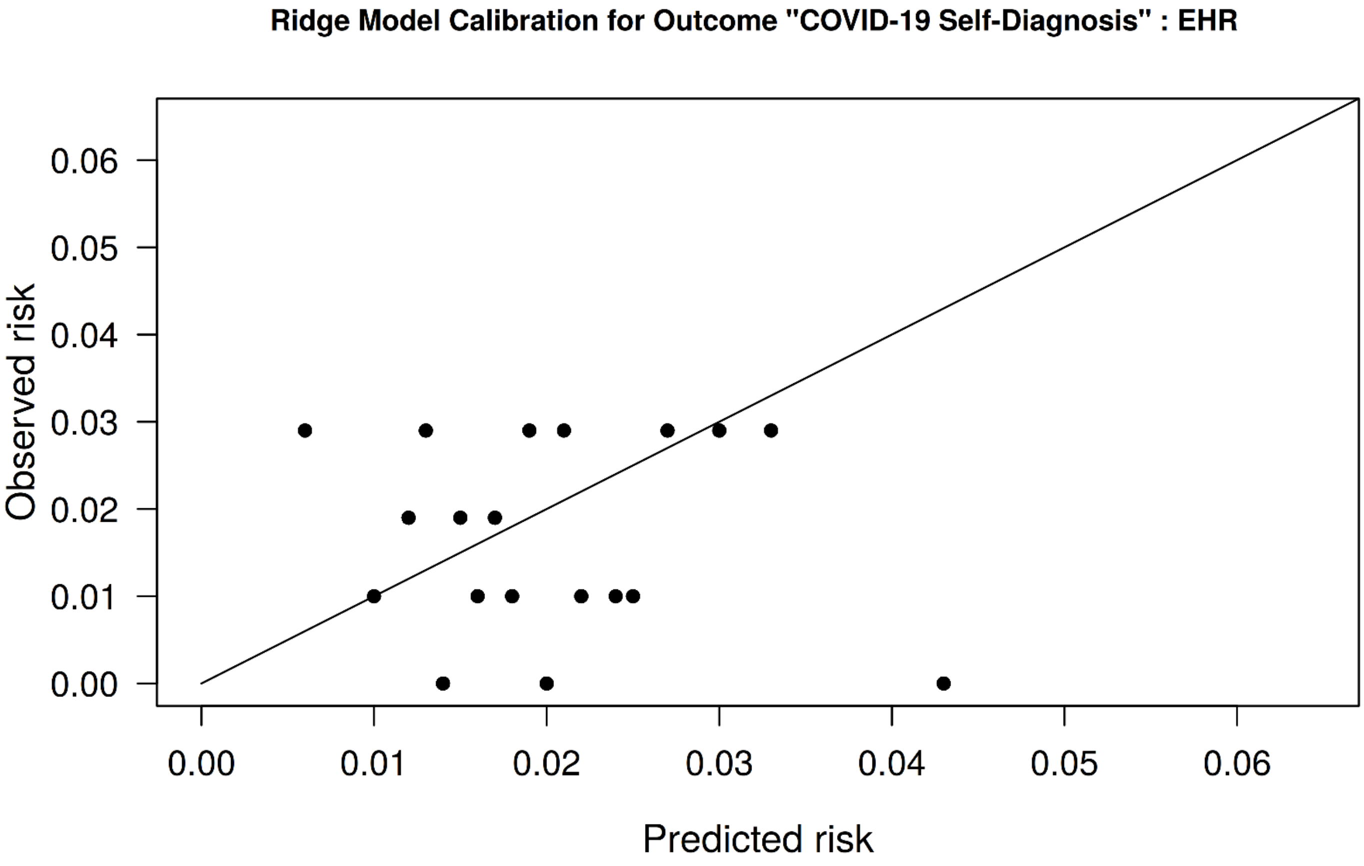

Subjects were split into 20 groups based on their predicted risk, and the observed proportion of COVID-19 tested subject in those groups was plotted against the expected proportion as per the model. The model used for this plot was from the first random 70/30 train-test split out of 100 total.

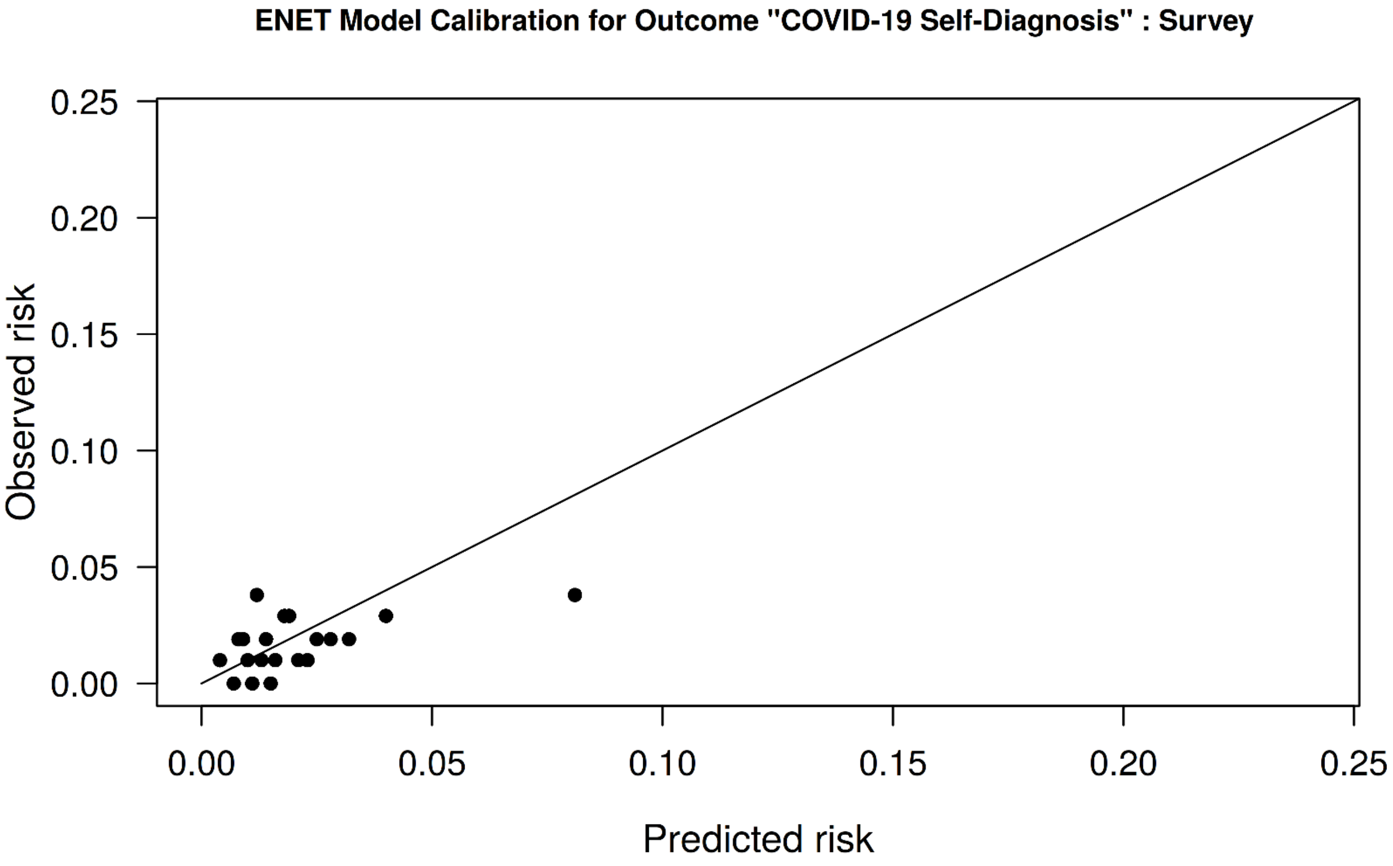

Subjects were split into 20 groups based on their predicted risk, and the observed proportion of COVID-19 tested subject in those groups was plotted against the expected proportion as per the model. The model used for this plot was from the first random 70/30 train-test split out of 100 total.

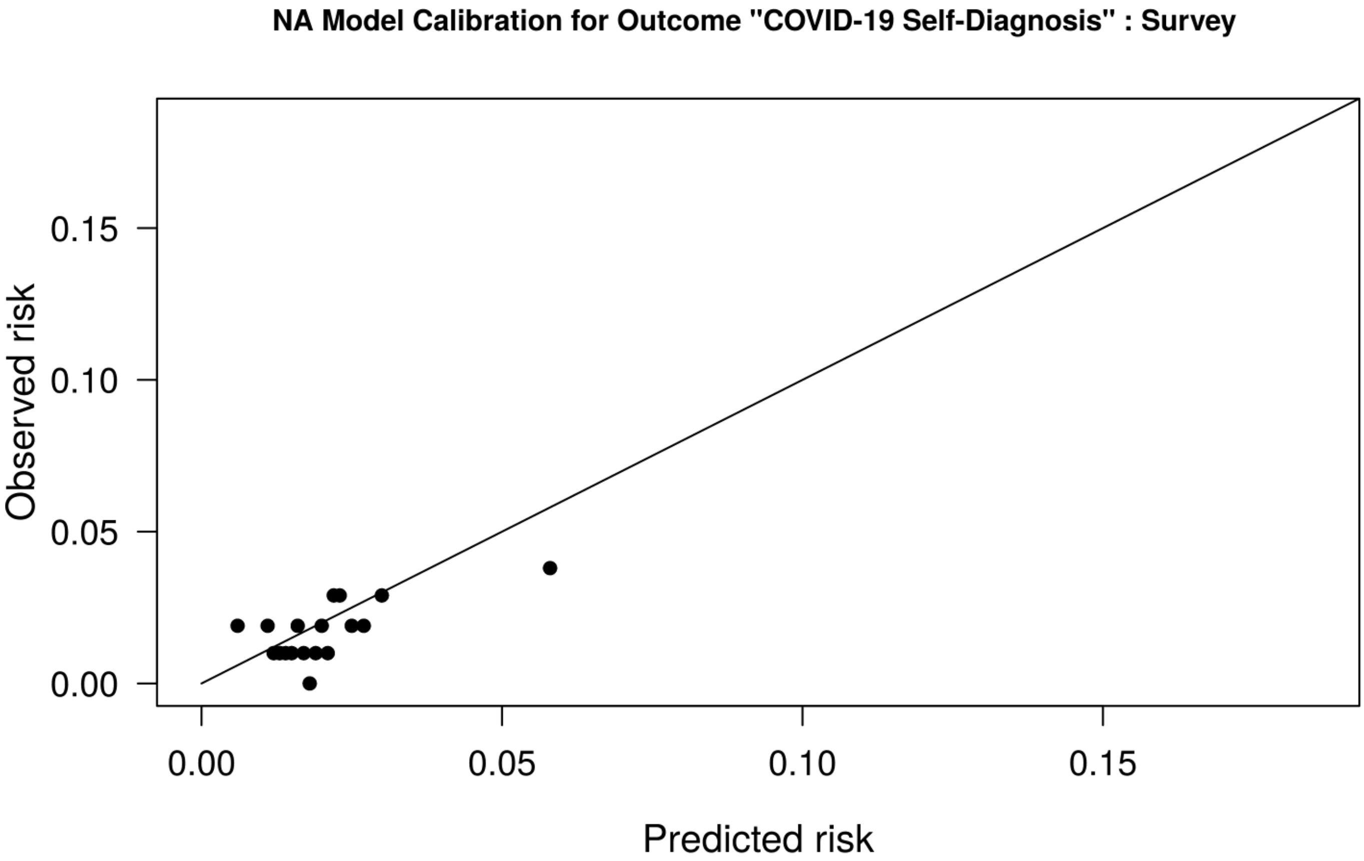

Subjects were split into 20 groups based on their predicted risk, and the observed proportion of COVID-19 tested subject in those groups was plotted against the expected proportion as per the model. The model used for this plot was from the first random 70/30 train-test split out of 100 total.

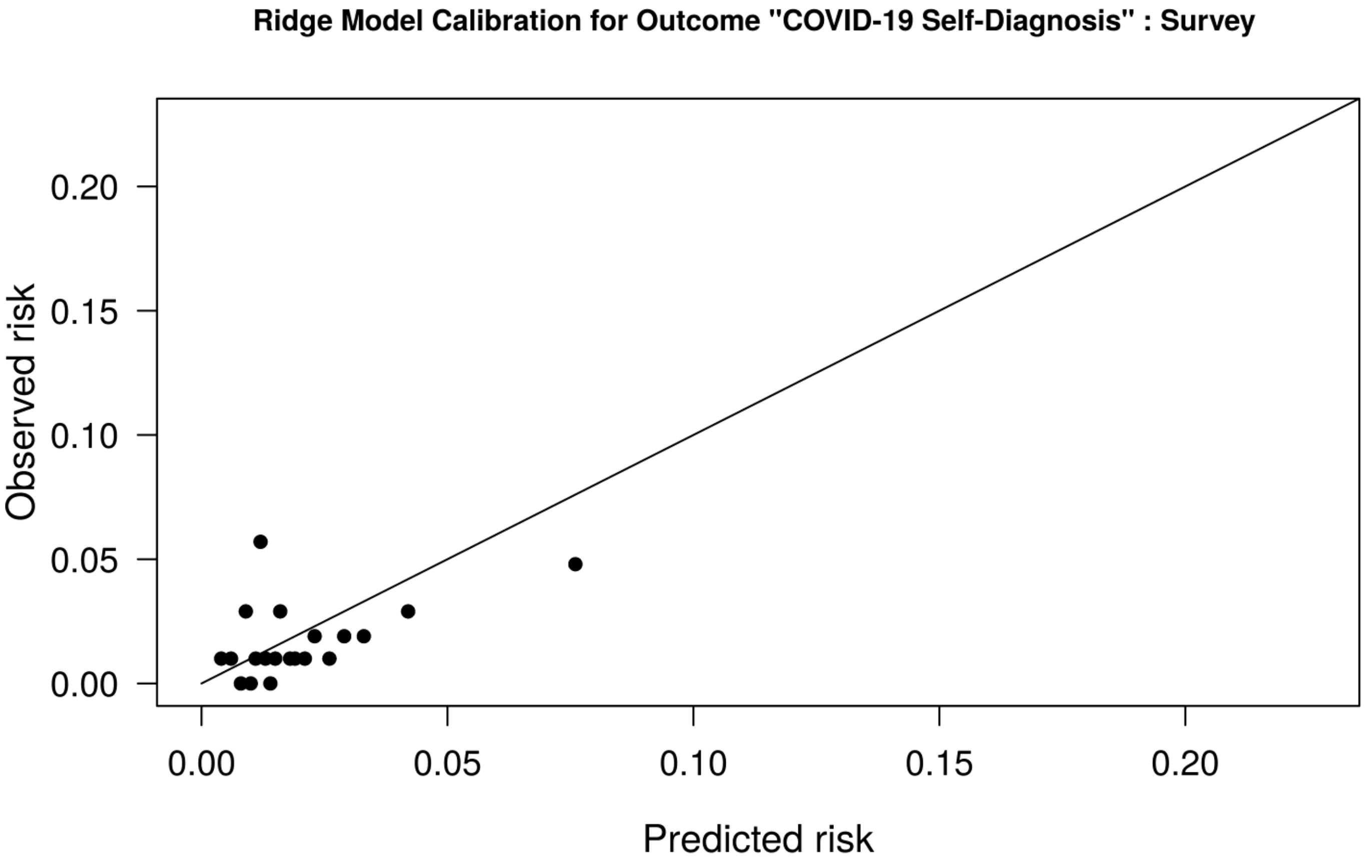

Subjects were split into 20 groups based on their predicted risk, and the observed proportion of COVID-19 tested subject in those groups was plotted against the expected proportion as per the model. The model used for this plot was from the first random 70/30 train-test split out of 100 total.

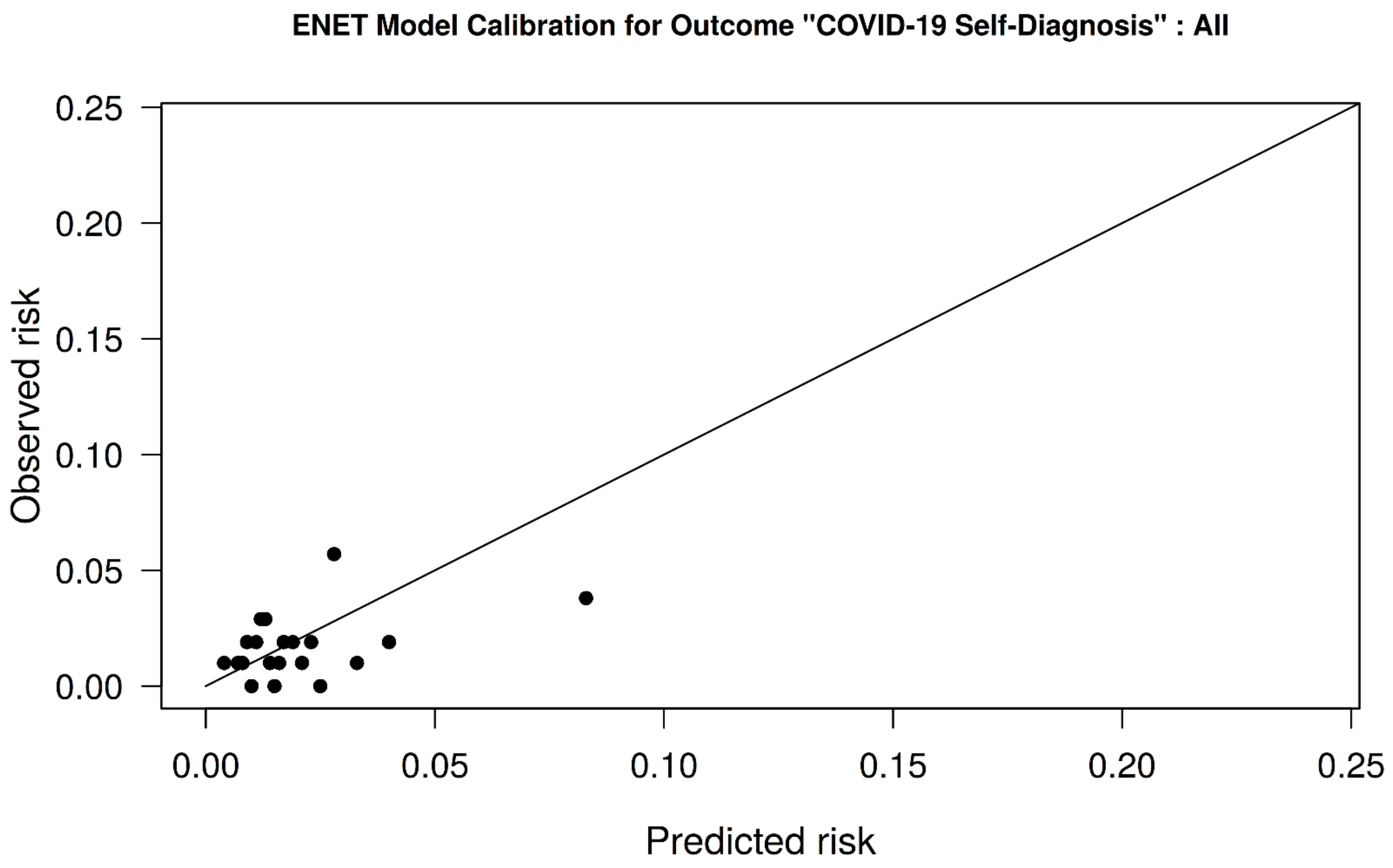

Subjects were split into 20 groups based on their predicted risk, and the observed proportion of COVID-19 tested subject in those groups was plotted against the expected proportion as per the model. The model used for this plot was from the first random 70/30 train-test split out of 100 total.

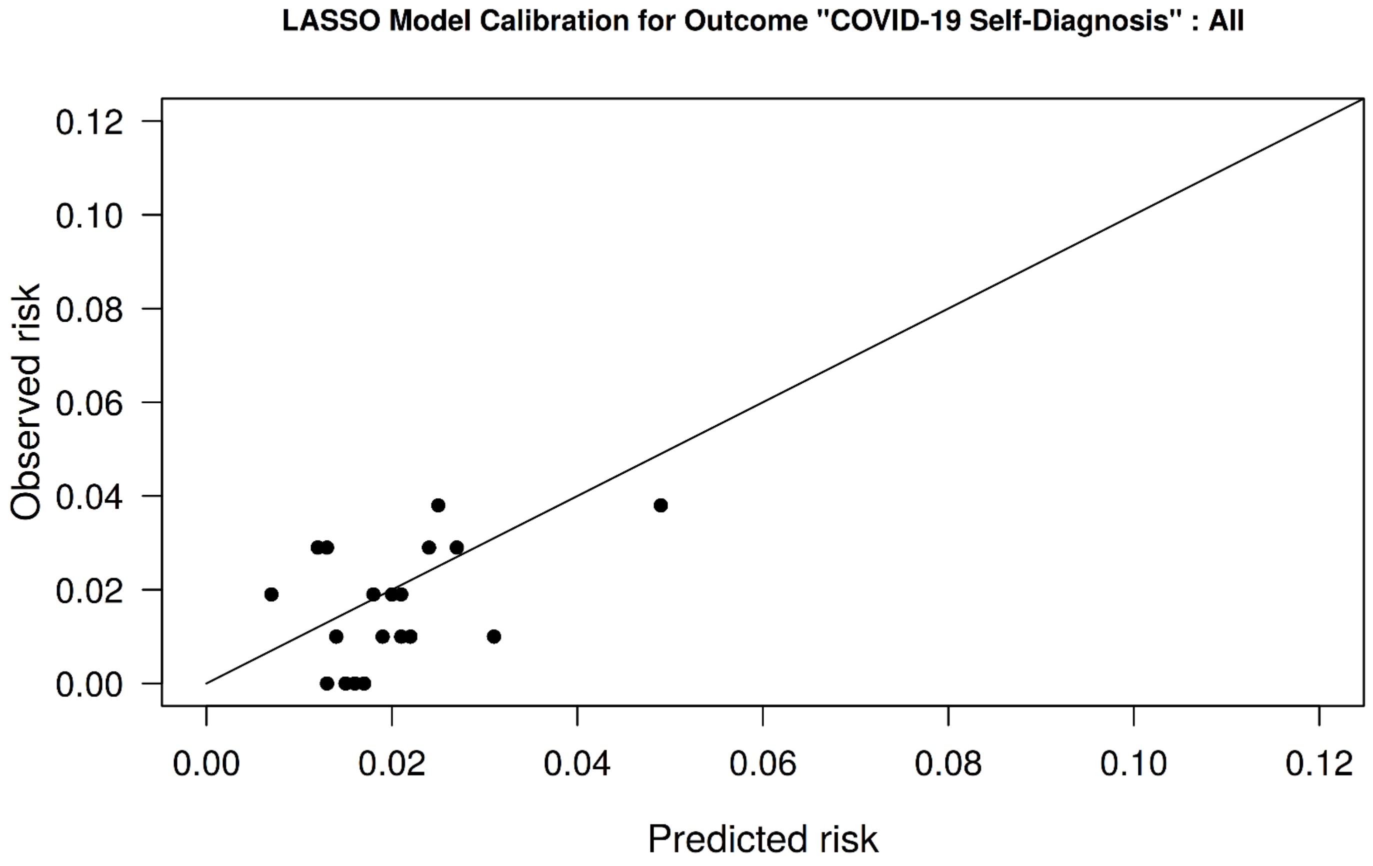

Subjects were split into 20 groups based on their predicted risk, and the observed proportion of COVID-19 tested subject in those groups was plotted against the expected proportion as per the model. The model used for this plot was from the first random 70/30 train-test split out of 100 total.

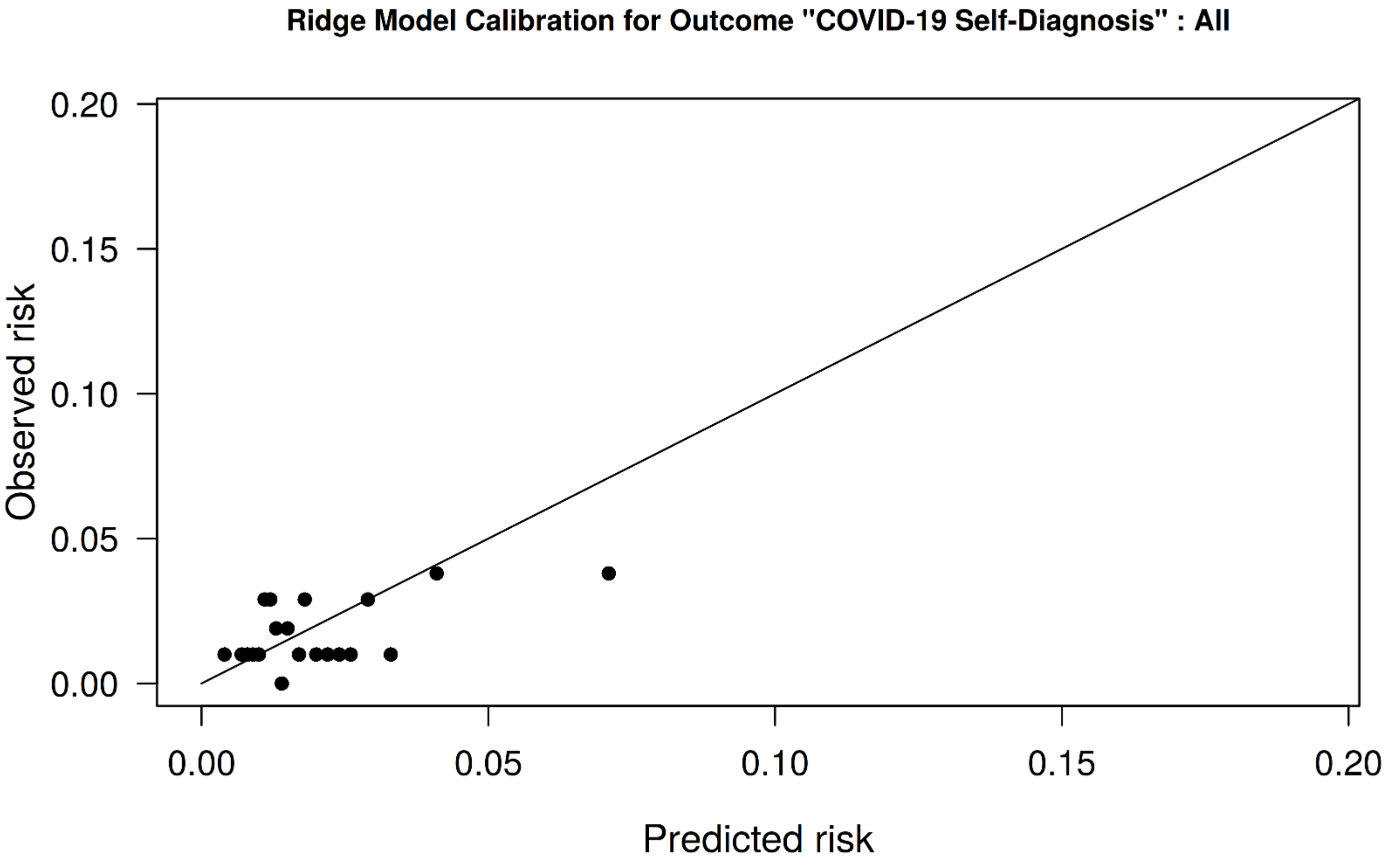

Subjects were split into 20 groups based on their predicted risk, and the observed proportion of COVID-19 tested subject in those groups was plotted against the expected proportion as per the model. The model used for this plot was from the first random 70/30 train-test split out of 100 total.

S16 Table. Comparison of Survey Respondents to Michigan Genomics Initiative and Michigan Medicine

	COVID-19 Survey N = 7,054		Michigan Genomics Initiative N = 82,372		Michigan Medicine N = 3,953,712	
	Number	Percent	Number	Percent	Number	Percent
**Age**						
18-30	379	5.37	8069	9.80	655185	16.57
31-40	689	9.77	8980	10.90	514851	13.02
41-50	907	12.86	11855	14.39	571074	14.44
51-60	1530	21.69	16699	20.27	635522	16.07
61-70	2066	29.29	19523	23.70	587345	14.86
71-80	1284	18.20	12880	15.64	393714	9.96
81-90	190	2.69	4365	5.30	298033	7.54
91+	9	0.13	0	0	297988	7.54
**Sex**						
Male	2831	40.13	37891	45.00	1800540	45.54
Female	4223	59.87	44479	54.00	2102545	53.18
Other	0	0	0	0.00	6406	0.16
Unknown	0	0	2	0.002	44222	1.12
**Race/Ethnicity**						
NHAA	158	2.24	4994	6.06	106744	2.70
NHW	6545	92.78	68341	82.97	950123	24.03
Unknown	90	1.28	3590	4.36	2750156	69.56
Other	261	3.70	5447	6.61	146695	3.71

Michigan Medicine records include patients who received treatment at any point from 01/01/2000 to 07/27/2020 and were over 18 years of age. Note that many self-reported Caucasians did not report an ethnicity, only a race, and therefore the number of unknowns in the Michigan Medicine Race/Ethnicity variable is large. Acronyms: NHAA, Non-Hispanic African American, NHW, Non-Hispanic White
