# Supplementary material for: Assessing the added value of linking electronic health records to improve the prediction of self-reported COVID-19 testing and diagnosis
Source: PLoS One. 2022 Jul 25;17(7):e0269017. doi: 10.1371/journal.pone.0269017 (PMC9312965; doi:10.1371/journal.pone.0269017)
Supplement: S8 Table — The value shown is the proportion of times the variable was chosen in 3,000 fitted models, as models were fit on 1000 train/test splits of 30 multiply imputed datasets (100x30 = 3,000). Only variables with a selection rate over 80% are included. Variable descriptions are available in the supplement (S1 Table). The tested for COVID-19 outcome compares the tested population (1) to those not tested (0). The diagnosed with COVID-19 outcome compares those diagnosed with COVID-19 by a physician or test (1) to those not diagnosed, not tested, and not self-diagnosed (0). The self-diagnosed with COVID-19 outcome compares those who diagnosed themselves with COVID-19 without a test to those who were not self-diagnosed or formally diagnosed (0). All models included the six covariates age, sex, race/ethnicity, body mass index, education level, and essential worker status, which were not selected for or penalized. Data from Michigan Medicine COVID-19 Survey and Michigan Genomics Initiative. Sample size: 6,159–7,054. (PDF) [file pone.0269017.s008.pdf]

S9 Table. LASSO Model Most Selected Variables

**Variable Selection Proportion. Outcome: Received a COVID-19 Test (Self-Reported)**					
EHR-Variable Models		Survey-Variable Models		All-Variable Models	
Kidney disease	0.95	Q17. Ever Hospitalized with infection	1.00	Q17. Ever Hospitalized with infection	1.00
Comorbidity score	0.92	Q36. Household member diagnosed with COVID-19	1.00	Q36. Household member diagnosed with COVID-19	1.00
		Q68.1 Felt fatigued in past week	1.00	Q68.1 Felt fatigued in past week	1.00
		Q147.1 Kidney disease	0.99	Q147.1 Kidney disease	0.99
		Q70.1 Abdomen pain in past 6 months	0.98	Q70.1 Abdomen pain in past 6 months	0.98
		Q146.2 COPD	0.96	Q146.2 COPD	0.96
		Q70.3 Headaches in past 6 months	0.96	Q70.3 Headaches in past 6 months	0.95
		Q125. Cardiovascular condition	0.95	Q125. Cardiovascular condition	0.94
		Q13. Number of times gotten flu in past year	0.94	Q13. Number of times gotten flu in past year	0.94
		Q125.7 Blood clotting disorder	0.91	Q125.7 Blood clotting disorder	0.9
		Q59.1. Police officer lives in home	0.87	Q59.1. Police officer lives in home	0.84
		Q23.3 Concerned about losing job	0.82		
**Variable Selection Proportion. Outcome: Diagnosed with COVID-19 (Self-Reported)**					
EHR-Variable Models		Survey-Variable Models		All-Variable Models	
<No Variables Over 0.8>		Q36. Household member diagnosed with COVID-19	1.00	Q36. Household member diagnosed with COVID-19	1.00
**Variable Selection Proportion. Outcome: Self-Diagnosed with COVID-19 (Self-Reported)**					
EHR-Variable Models		Survey-Variable Models		All-Variable Models	
<No Variables Over 0.8>		Q36. Household member diagnosed with COVID-19	1.00	Q36. Household member diagnosed with COVID-19	1.00

The value shown is the proportion of times the variable was chosen in 3,000 fitted models, as models were fit on 1000 train/test splits of 30 multiply imputed datasets (100x30=3,000). Only variables with a selection rate over 80% are included. Variable descriptions are available in the supplement (S1 Table). The tested for COVID-19 outcome compares the tested population (1) to those not tested (0). The diagnosed with COVID-19 outcome compares those diagnosed with COVID-19 by a physician or test (1) to those not diagnosed, not tested, and not self-diagnosed (0). The self-diagnosed with COVID-19 outcome compares those who diagnosed themselves with COVID-19 without a test to those who were not self-diagnosed or formally diagnosed (0). All models included the six covariates age, sex, race/ethnicity, body mass index, education level, and essential worker status, which were not selected for or penalized. Data from Michigan Medicine COVID-19 Survey and Michigan Genomics Initiative. Sample size: 6,159 – 7,054
