# Supplementary material for: Assessing the added value of linking electronic health records to improve the prediction of self-reported COVID-19 testing and diagnosis
Source: PLoS One. 2022 Jul 25;17(7):e0269017. doi: 10.1371/journal.pone.0269017 (PMC9312965; doi:10.1371/journal.pone.0269017)
Supplement: S9 Table — The value shown is the proportion of times the variable was chosen in 3,000 fitted models, as models were fit on 1000 train/test splits of 30 multiply imputed datasets (100x30 = 3,000). Only variables with a selection rate over 80% are included. Variable descriptions are available in the supplement (S1 Table). The tested for COVID-19 outcome compares the tested population (1) to those not tested (0). The diagnosed with COVID-19 outcome compares those diagnosed with COVID-19 by a physician or test (1) to those not diagnosed, not tested, and not self-diagnosed (0). The self-diagnosed with COVID-19 outcome compares those who diagnosed themselves with COVID-19 without a test to those who were not self-diagnosed or formally diagnosed (0). All models included the six covariates age, sex, race/ethnicity, body mass index, education level, and essential worker status, which were not selected for or penalized. Data from Michigan Medicine COVID-19 Survey and Michigan Genomics Initiative. Sample size: 6,159–7,054. (PDF) [file pone.0269017.s009.pdf]

S10 Table. ENET Model Most-Selected Variables

**Variable Selection Proportion. Outcome: Received a COVID-19 Test (Self-Reported)**					
EHR-Variable Models		Survey-Variable Models		All-Variable Models	
Comorbidity score*	0.99	Q17. Ever Hospitalized with infection	1.00	Q17. Ever Hospitalized with infection	1.00
Kidney disease*	0.98	Q36. Household member diagnosed with COVID-19	1.00	Q36. Household member diagnosed with COVID-19	1.00
Respiratory disease*	0.94	Q147.1 Kidney disease	1.00	Q147.1 Kidney disease	1.00
Liver disease*	0.91	Q68.1 Felt fatigued in past week	1.00	Q68.1 Felt fatigued in past week	1.00
Former smoker*	0.80	Q70.1 Abdomen pain in past 6 months	1.00	Q70.1 Abdomen pain in past 6 months	1.00
		Q70.3 Headaches in past 6 months	1.00	Q70.3 Headaches in past 6 months	0.99
		Q13. No times gotten flu in past year	0.99	Q13. No times gotten flu in past year	0.99
		Q125. Cardiovascular condition	0.98	Q125. Cardiovascular condition	0.98
		Q146.2 COPD	0.98	Q146.2 COPD	0.97
		Q147. Metabolic Condition	0.96	Q125.7 Blood clotting disorder	0.95
		Q125.7 Has cardiovascular condition	0.95	Q147. Metabolic condition	0.94
		Q59.1 Police officer lives in home	0.95	Q59.1 Police officer lives in home	0.94
		Q23.3 Concerned about losing job	0.95	Q114.1 Overall body pain at worst	0.94
		Q71.1 Some difficult doing chores	0.94	Q23.3 Concerned about losing job	0.93
		Q71.1 Much difficulty doing chores	0.94	Q71.1 Much difficulty doing chores	0.93
		Q114.1 Overall body pain at worst	0.94	Q71.1 Some difficulty doing chores	0.93
		Q133.2 Benzodiazepine use has increased	0.88	Q133.2 Benzodiazepine use has increased	0.87
		Q114.2 Overall body pain on average	0.86	Q68.3 Trouble waking up refreshed	0.85
		Q46. Flu shot in past year	0.86	Q77. Poor sleep quality, past 7 days	0.85
		Q77. Poor sleep quality, past 7 days	0.86	Q114.2 Overall body pain, on average	0.85
		Q68.3 Trouble waking up refreshed	0.85	Q133.1 Opioid use has increased	0.83
		Q133.1 Opioid use has increase	0.85	Q46. Flu shot in past year	0.83
		Q36.1. Lives alone	0.83	Q36.1. Lives alone	0.81
		Q68.2 Memory trouble in past week	0.81		
**Variable Selection Proportion. Outcome: Diagnosed with COVID-19 (Self-Reported)**					
EHR-Variable Models		Survey-Variable Models		All-Variable Models	
Liver disease	0.92	Q36. Household member diagnosed with COVID-19	1.00	Q36. Household member diagnosed with COVID-19	1.00
Respiratory disease	0.87	Q85. Relative died from COVID-19	0.85	Q85. Relative died from COVID-19	0.82
		Q70.1 Abdomen pain in past 6 months	0.82	Q81. Relative diagnosed with COVID-19	0.81
		Q81. Relative diagnosed with COVID-19	0.82	Q70.1 Abdomen pain in past 6 months	0.80
**Variable Selection Proportion. Outcome: Self-Diagnosed with COVID-19 (Self-Reported)**					
EHR-Variable Models		Survey-Variable Models		All-Variable Models	
<No Variables Over 0.8>		Q36. Household member diagnosed with COVID-19	1	Q36. Household member diagnosed with COVID-19	1
		Q70.3 Headaches in past 6 months	0.95	Q70.3 Headaches in past 6 months	0.93
		Q81. Relative diagnosed with COVID-19	0.9	Q81. Relative diagnosed with COVID-19	0.89
		Q23.1 Concerned about someone close contracting COVID-19	0.87	Q23.1 Concerned about someone close contracting COVID-19	0.87

S10 Table (continued)

		Q118.5 Sleep habits have improved during pandemic	0.84	Q70.2 Depression in past 6 months	0.83
		Q28.16 Family member has irregular heartbeat	0.83	Q28.16 Family member has irregular heartbeat	0.81
		Q70.2 Depression in past 6 months	0.82	Q118.5 Sleep habits have improved during pandemic	0.81

The value shown is the proportion of times the variable was chosen in 3,000 fitted models, as models were fit on 1000 train/test splits of 30 multiply imputed datasets (100x30=3,000). Only variables with a selection rate over 80% are included. Variable descriptions are available in the supplement (S1 Table). The tested for COVID-19 outcome compares the tested population (1) to those not tested (0). The diagnosed with COVID-19 outcome compares those diagnosed with COVID-19 by a physician or test (1) to those not diagnosed, not tested, and not self-diagnosed (0). The self-diagnosed with COVID-19 outcome compares those who diagnosed themselves with COVID-19 without a test to those who were not self-diagnosed or formally diagnosed (0). All models included the six covariates age, sex, race/ethnicity, body mass index, education level, and essential worker status, which were not selected for or penalized. Data from Michigan Medicine COVID-19 Survey and Michigan Genomics Initiative. Sample size: 6,159 – 7,054
