# Supplementary material for: Assessing the added value of linking electronic health records to improve the prediction of self-reported COVID-19 testing and diagnosis
Source: PLoS One. 2022 Jul 25;17(7):e0269017. doi: 10.1371/journal.pone.0269017 (PMC9312965; doi:10.1371/journal.pone.0269017)
Supplement: S10 Table — Lambda and alpha were selected by five-fold cross-validation on the training set of a single 70/30 train/test split. (PDF) [file pone.0269017.s010.pdf]

S11 Table. Mean Penalties Selected by Elastic Net Regression Models

**Elastic Net Regression Model Penalties – Outcome: Received a COVID-19 Test**						
	Covariates + EHR Variables		Covariates + Survey Variables		All Variables	
Split	Lambda	Alpha	Lambda	Alpha	Lambda	Alpha
1	0.020	0.247	0.051	0.100	0.057	0.107
2	0.013	0.267	0.052	0.107	0.054	0.140
3	0.017	0.100	0.054	0.160	0.052	0.180
4	0.019	0.213	0.052	0.100	0.055	0.100
5	0.022	0.107	0.049	0.160	0.044	0.213
6	0.012	0.273	0.048	0.200	0.047	0.187
7	0.018	0.120	0.054	0.113	0.056	0.107
8	0.020	0.120	0.039	0.300	0.034	0.360
9	0.021	0.120	0.045	0.187	0.045	0.227
10	0.012	0.407	0.042	0.260	0.039	0.313
**Elastic Net Regression Model Penalties – Outcome: Diagnosed with COVID-19**						
	Covariates + EHR Variables		Covariates + Survey Variables		All Variables	
Split	Lambda	Alpha	Lambda	Alpha	Lambda	Alpha
1	0.007	0.100	0.015	0.893	0.015	0.893
2	0.005	0.100	0.017	0.773	0.018	0.793
3	0.019	0.133	0.016	0.840	0.016	0.860
4	0.005	0.100	0.005	0.700	0.005	0.573
5	0.005	0.107	0.005	0.300	0.005	0.293
6	0.006	0.100	0.020	0.300	0.020	0.300
7	0.005	0.100	0.028	0.260	0.005	0.100
8	0.005	0.100	0.037	0.447	0.039	0.400
9	0.006	0.407	0.039	0.333	0.037	0.347
10	0.025	0.167	0.005	0.900	0.005	0.900
**Elastic Net Regression Model Penalties – Outcome: Self-Diagnosed with COVID-19**						
	Covariates + EHR Variables		Covariates + Survey Variables		All Variables	
Split	Lambda	Alpha	Lambda	Alpha	Lambda	Alpha
1	0.007	0.107	0.009	0.153	0.009	0.140
2	0.042	0.107	0.019	0.127	0.020	0.100
3	0.032	0.107	0.005	0.300	0.005	0.293
4	0.066	0.113	0.005	0.627	0.005	0.667
5	0.072	0.133	0.006	0.793	0.005	0.900
6	0.006	0.167	0.006	0.100	0.008	0.160
7	0.020	0.140	0.005	0.500	0.005	0.500
8	0.017	0.100	0.019	0.100	0.019	0.100
9	0.040	0.147	0.006	0.100	0.009	0.100
10	0.064	0.120	0.005	0.420	0.005	0.520

Lambda and alpha shown are the mean across 30 models fit on multiply imputed datasets. Lambda and alpha were selected by five-fold cross-validation on the training set of a single 70/30 train/test split.
