# Supplementary material for: Assessing the added value of linking electronic health records to improve the prediction of self-reported COVID-19 testing and diagnosis
Source: PLoS One. 2022 Jul 25;17(7):e0269017. doi: 10.1371/journal.pone.0269017 (PMC9312965; doi:10.1371/journal.pone.0269017)
Supplement: S11 Table — Lambda and alpha were selected by five-fold cross-validation on the training set of a single 70/30 train/test split. (PDF) [file pone.0269017.s011.pdf]

S12 Table. Mean Penalties Selected by Ridge Regression Models

| **Ridge Regression Model Penalties – Outcome: Received a COVID-19 Test** | | | | | | |
|---|---|---|---|---|---|---|
| | Covariates + EHR Variables | | Covariates + Survey Variables | | All Variables | |
| Split | Lambda | Alpha | Lambda | Alpha | Lambda | Alpha |
| 1 | 0.069 | 0.000 | 0.100 | 0.000 | 0.100 | 0.000 |
| 2 | 0.074 | 0.000 | 0.100 | 0.000 | 0.100 | 0.000 |
| 3 | 0.060 | 0.000 | 0.100 | 0.000 | 0.100 | 0.000 |
| 4 | 0.072 | 0.000 | 0.100 | 0.000 | 0.100 | 0.000 |
| 5 | 0.062 | 0.000 | 0.100 | 0.000 | 0.100 | 0.000 |
| 6 | 0.044 | 0.000 | 0.100 | 0.000 | 0.100 | 0.000 |
| 7 | 0.057 | 0.000 | 0.100 | 0.000 | 0.100 | 0.000 |
| 8 | 0.063 | 0.000 | 0.100 | 0.000 | 0.100 | 0.000 |
| 9 | 0.051 | 0.000 | 0.100 | 0.000 | 0.100 | 0.000 |
| 10 | 0.052 | 0.000 | 0.100 | 0.000 | 0.100 | 0.000 |
| **Ridge Regression Model Penalties – Outcome: Diagnosed with COVID-19** | | | | | | |
| | Covariates + EHR Variables | | Covariates + Survey Variables | | All Variables | |
| Split | Lambda | Alpha | Lambda | Alpha | Lambda | Alpha |
| 1 | 0.019 | 0.000 | 0.061 | 0.000 | 0.056 | 0.000 |
| 2 | 0.006 | 0.000 | 0.100 | 0.000 | 0.099 | 0.000 |
| 3 | 0.061 | 0.000 | 0.098 | 0.000 | 0.100 | 0.000 |
| 4 | 0.005 | 0.000 | 0.075 | 0.000 | 0.068 | 0.000 |
| 5 | 0.010 | 0.000 | 0.036 | 0.000 | 0.035 | 0.000 |
| 6 | 0.013 | 0.000 | 0.100 | 0.000 | 0.099 | 0.000 |
| 7 | 0.005 | 0.000 | 0.023 | 0.000 | 0.015 | 0.000 |
| 8 | 0.016 | 0.000 | 0.099 | 0.000 | 0.089 | 0.000 |
| 9 | 0.034 | 0.000 | 0.098 | 0.000 | 0.100 | 0.000 |
| 10 | 0.064 | 0.000 | 0.039 | 0.000 | 0.044 | 0.000 |
| **Ridge Regression Model Penalties – Outcome: Self-Diagnosed with COVID-19** | | | | | | |
| | Covariates + EHR Variables | | Covariates + Survey Variables | | All Variables | |
| Split | Lambda | Alpha | Lambda | Alpha | Lambda | Alpha |
| 1 | 0.019 | 0.000 | 0.042 | 0.000 | 0.049 | 0.000 |
| 2 | 0.049 | 0.000 | 0.066 | 0.000 | 0.071 | 0.000 |
| 3 | 0.061 | 0.000 | 0.031 | 0.000 | 0.039 | 0.000 |
| 4 | 0.037 | 0.000 | 0.064 | 0.000 | 0.066 | 0.000 |
| 5 | 0.069 | 0.000 | 0.033 | 0.000 | 0.040 | 0.000 |
| 6 | 0.023 | 0.000 | 0.024 | 0.000 | 0.025 | 0.000 |
| 7 | 0.050 | 0.000 | 0.055 | 0.000 | 0.061 | 0.000 |
| 8 | 0.021 | 0.000 | 0.057 | 0.000 | 0.053 | 0.000 |
| 9 | 0.068 | 0.000 | 0.023 | 0.000 | 0.026 | 0.000 |
| 10 | 0.084 | 0.000 | 0.082 | 0.000 | 0.083 | 0.000 |

Lambda and alpha shown are the mean across 30 models fit on multiply imputed datasets. Lambda and alpha were selected by five-fold cross-validation on the training set of a single 70/30 train/test split.
