# Supplementary material for: Assessing the added value of linking electronic health records to improve the prediction of self-reported COVID-19 testing and diagnosis
Source: PLoS One. 2022 Jul 25;17(7):e0269017. doi: 10.1371/journal.pone.0269017 (PMC9312965; doi:10.1371/journal.pone.0269017)
Supplement: S12 Table — Lambda and alpha were selected by five-fold cross-validation on the training set of a single 70/30 train/test split. (PDF) [file pone.0269017.s012.pdf]

S13 Table. Mean Penalties Selected by Lasso Regression Models

**Lasso Regression Model Penalties – Outcome: Received a COVID-19 Test**						
	Covariates + EHR Variables		Covariates + Survey Variables		All Variables	
Split	Lambda	Alpha	Lambda	Alpha	Lambda	Alpha
1	0.005	1.000	0.007	1.000	0.007	1.000
2	0.006	1.000	0.007	1.000	0.008	1.000
3	0.008	1.000	0.008	1.000	0.008	1.000
4	0.008	1.000	0.006	1.000	0.006	1.000
5	0.008	1.000	0.007	1.000	0.007	1.000
6	0.005	1.000	0.007	1.000	0.007	1.000
7	0.008	1.000	0.006	1.000	0.006	1.000
8	0.005	1.000	0.008	1.000	0.008	1.000
9	0.005	1.000	0.006	1.000	0.007	1.000
10	0.009	1.000	0.009	1.000	0.009	1.000
**Lasso Regression Model Penalties – Outcome: Diagnosed with COVID-19**						
	Covariates + EHR Variables		Covariates + Survey Variables		All Variables	
Split	Lambda	Alpha	Lambda	Alpha	Lambda	Alpha
1	0.100	1.000	0.015	1.000	0.015	1.000
2	0.100	1.000	0.015	1.000	0.015	1.000
3	0.100	1.000	0.017	1.000	0.017	1.000
4	0.100	1.000	0.005	1.000	0.005	1.000
5	0.100	1.000	0.010	1.000	0.010	1.000
6	0.100	1.000	0.010	1.000	0.010	1.000
7	0.100	1.000	0.005	1.000	0.005	1.000
8	0.100	1.000	0.020	1.000	0.020	1.000
9	0.100	1.000	0.020	1.000	0.020	1.000
10	0.100	1.000	0.005	1.000	0.005	1.000
**Lasso Regression Model Penalties – Outcome: Self-Diagnosed with COVID-19**						
	Covariates + EHR Variables		Covariates + Survey Variables		All Variables	
Split	Lambda	Alpha	Lambda	Alpha	Lambda	Alpha
1	0.100	1.00	0.006	1.000	0.006	1.000
2	0.100	1.00	0.006	1.000	0.006	1.000
3	0.100	1.00	0.005	1.000	0.005	1.000
4	0.100	1.00	0.008	1.000	0.008	1.000
5	0.100	1.00	0.006	1.000	0.006	1.000
6	0.100	1.00	0.005	1.000	0.005	1.000
7	0.100	1.00	0.007	1.000	0.007	1.000
8	0.100	1.00	0.006	1.000	0.007	1.000
9	0.100	1.00	0.006	1.000	0.006	1.000
10	0.100	1.00	0.007	1.000	0.007	1.000

Lambda and alpha shown are the mean across 30 models fit on multiply imputed datasets. Lambda and alpha were selected by five-fold cross-validation on the training set of a single 70/30 train/test split.
