# Supplementary material for: Assessing the added value of linking electronic health records to improve the prediction of self-reported COVID-19 testing and diagnosis
Source: PLoS One. 2022 Jul 25;17(7):e0269017. doi: 10.1371/journal.pone.0269017 (PMC9312965; doi:10.1371/journal.pone.0269017)
Supplement: S13 Table — Michigan Medicine records include patients who received treatment at any point from 01/01/2000 to 07/27/2020 and were over 18 years of age. Note that many self-reported Caucasians did not report an ethnicity, only a race, and therefore the number of unknowns in the Michigan Medicine Race/Ethnicity variable is large. Acronyms: NHAA, Non-Hispanic African American, NHW, Non-Hispanic White. (PDF) [file pone.0269017.s013.pdf]

S16 Table. Comparison of Survey Respondents to Michigan Genomics Initiative and Michigan Medicine

	COVID-19 Survey N = 7,054		Michigan Genomics Initiative N = 82,372		Michigan Medicine N = 3,953,712	
	Number	Percent	Number	Percent	Number	Percent
**Age**						
18-30	379	5.37	8069	9.80	655185	16.57
31-40	689	9.77	8980	10.90	514851	13.02
41-50	907	12.86	11855	14.39	571074	14.44
51-60	1530	21.69	16699	20.27	635522	16.07
61-70	2066	29.29	19523	23.70	587345	14.86
71-80	1284	18.20	12880	15.64	393714	9.96
81-90	190	2.69	4365	5.30	298033	7.54
91+	9	0.13	0	0	297988	7.54
**Sex**						
Male	2831	40.13	37891	45.00	1800540	45.54
Female	4223	59.87	44479	54.00	2102545	53.18
Other	0	0	0	0.00	6406	0.16
Unknown	0	0	2	0.002	44222	1.12
**Race/Ethnicity**						
NHAA	158	2.24	4994	6.06	106744	2.70
NHW	6545	92.78	68341	82.97	950123	24.03
Unknown	90	1.28	3590	4.36	2750156	69.56
Other	261	3.70	5447	6.61	146695	3.71

Michigan Medicine records include patients who received treatment at any point from 01/01/2000 to 07/27/2020 and were over 18 years of age. Note that many self-reported Caucasians did not report an ethnicity, only a race, and therefore the number of unknowns in the Michigan Medicine Race/Ethnicity variable is large. Acronyms: NHAA, Non-Hispanic African American, NHW, Non-Hispanic White
