# Supplementary material for: Assessing the added value of linking electronic health records to improve the prediction of self-reported COVID-19 testing and diagnosis
Source: PLoS One. 2022 Jul 25;17(7):e0269017. doi: 10.1371/journal.pone.0269017 (PMC9312965; doi:10.1371/journal.pone.0269017)
Supplement: S2 File — (PDF) [file pone.0269017.s015.pdf]

EHR-Based Supplementary Analysis

To see how the 15 EHR-derived variables would perform in the larger cohort that was not surveyed, we ran similar models using COVID-19 testing and diagnosis case-control data from Michigan Medicine. To construct the COVID-19 tested cases, we retrieved data for all 15,929 patients who had obtained a reverse transcription polymerase chain reaction (RT-PCR) test for SARS-CoV-2 at Michigan Medicine between March 10th and June 30th, 2020 (Figure 1). For COVID-19 diagnosed cases, we used the 1,193 who had tested positive, along with another 290 patients who had had COVID-19 per their EHRs but had no test results (this latter group would have included, for example, patients who were treated for COVID-19 at Michigan Medicine but were not tested there). This resulted in a total of 1,483 diagnosed cases for the analysis. Lastly, for controls, we extracted data for 30,000 random patients who were alive, were not in the tested or diagnosed groups, and had an encounter in Michigan Medicine (Inpatient, Outpatient, or Emergency) between April 23, 2012, and June 21, 2020.

Figure 1. Extraction of Michigan Case-Control Records for COVID-19-related Outcomes

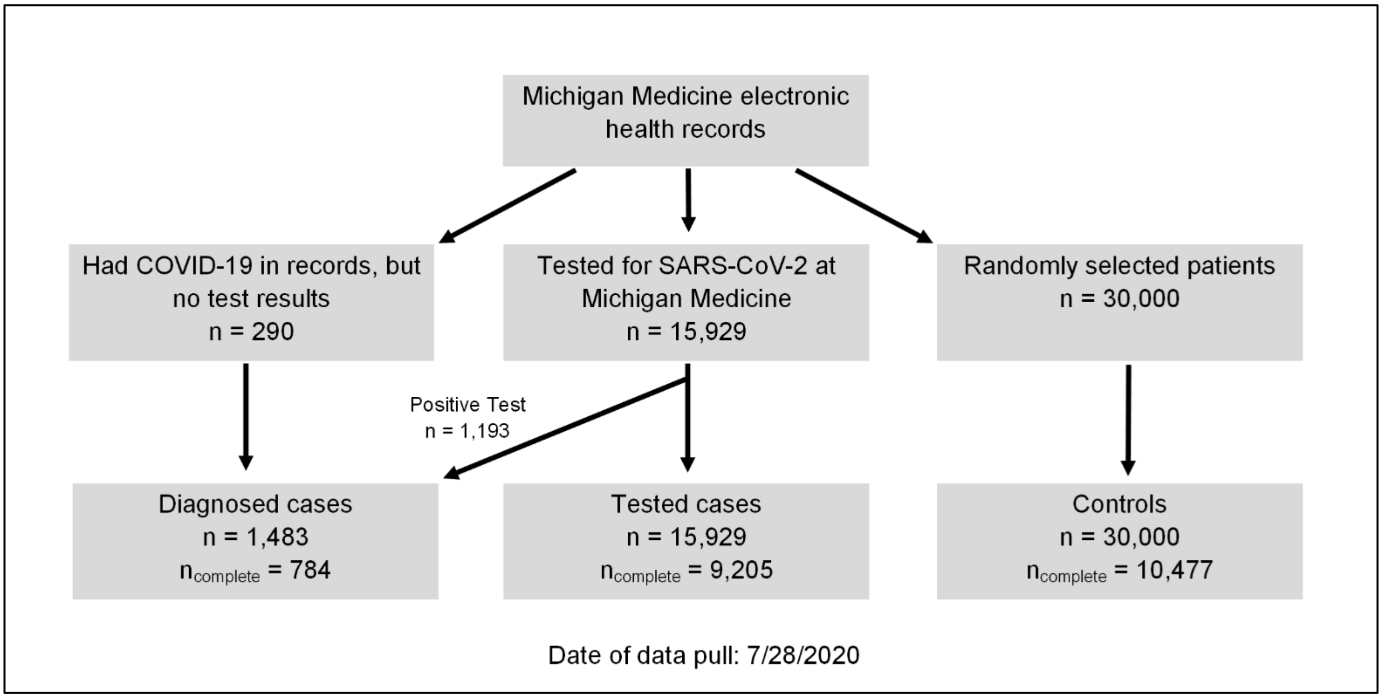

Patients were coded as “1” for the testing models if they were tested for COVID-19 at Michigan Medicine, and “1” for the diagnosis models if they tested positive or were diagnosed with COVID-19. Random controls

were coded as “0”. We used the same covariates as in the survey-based models except for education and essential worker status, which were not available. In addition, we attempted two different constructions of the health condition indicator variables: A *restricted* version, which limited health conditions to pre-existing conditions (those appearing at least 14 days prior to the patient’s first COVID-19 diagnosis or test, if they had one), and an *unrestricted* version, which included health conditions appearing up to *the day of* that patient’s first COVID-19 test or diagnosis (Figure 2A). This way, the EHR-derived variables in the unrestricted data would also be able to capture health conditions that occurred as a result of COVID-19 infection, rather than only those that existed beforehand, as symptoms usually appear within 14 days of infection [25]. Using both datasets separately, we applied similar models to our main analysis: a ridge regression, lasso regression, and elastic net regression (Figure 2B). All models adjusted for the four covariates age, sex, race/ethnicity, and body mass index, which were not selected for or penalized. Tuning parameters were selected using five-fold cross-validation. As in the main analysis, models were evaluated internally by computing the test set AUC of 100 random train/test splits and taking the average of those 100, along with the 2.5^{th} and 97.5^{th} percentiles for an empirical confidence interval. Note that, since the degree of missingness in the data was high, but sample size was large, we elected to conduct a complete case analysis rather than pursuing multiple imputation, our approach for the survey-based analysis described in the main text.

Figure 2. Analysis Overview of Michigan Medicine Case-Control Data

(A) Health condition predictor variables were defined differently for two separate analyses, a restricted version and an unrestricted version. (B) Multivariable logistic models were used to assess how well EHR-derived variables could discriminate COVID-19 cases from controls.

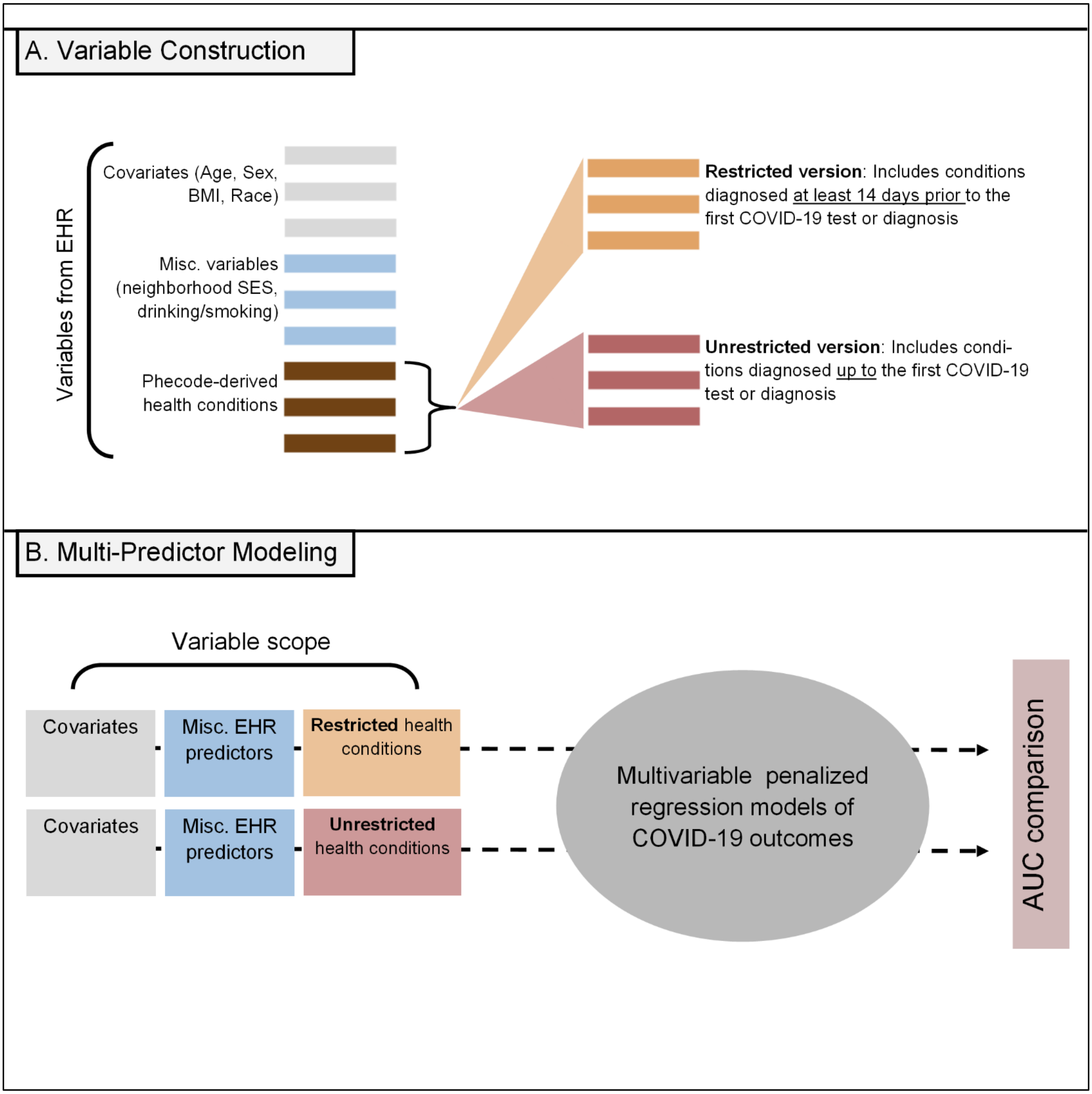

S14 File (continued)

Results for the analysis are shown below in Table 1.

Table 1. Area under the Curve (AUC) for Michigan Medicine Case-Control Analysis of COVID-19 Outcomes

Mean AUC (95% Empirical CI)				
Outcome Variable	Model Type	Covariates Only	Covariates + EHR Variables (Restricted)	Covariates + EHR Variables (Unrestricted)
Tested for COVID-19	Lasso	0.563 (0.551, 0.574)	0.723 (0.712, 0.732)	0.747 (0.737, 0.756)
	Ridge Regression	0.563 (0.551, 0.574)	0.724 (0.713, 0.734)	0.748 (0.737, 0.757)
	Elastic Net	0.563 (0.551, 0.574)	0.724 (0.712, 0.734)	0.748 (0.737, 0.757)
Diagnosed with COVID-19	Lasso	0.671 (0.643, 0.698)	0.759 (0.735, 0.785)	0.79 (0.77, 0.812)
	Ridge Regression	0.671 (0.643, 0.698)	0.774 (0.749, 0.803)	0.803 (0.785, 0.823)
	Elastic net	0.671 (0.643, 0.698)	0.773 (0.748, 0.801)	0.802 (0.784, 0.823)

Mean AUC reflects the average of 100 random training test/splits, with a CI representing the 2.5th and 97.5th percentiles, respectively. Data from Michigan Medicine COVID-19 Cohort. Corresponding models include only EHR-derived predictors and covariates. Sample size: n = 19,682 for testing models, n = 11,261 for diagnosis models.

In the following tables and figures, we provide the selected tuning parameters for the models and plots describing their calibration. For tuning parameters, we present the λ and α selected for the first ten data splits of the procedure. Note that $\lambda_1 = \alpha \times \lambda$ and $\lambda_2 = (1 - \alpha)/2 \times \lambda$. For calibration, the first set of plots show the distribution of Hosmer-Lemeshow test p-values for all 100 training-test splits. A p-value less than 0.05 suggests poor calibration. The second set of plots using only the first training-test split and show the predicted probabilities of each outcome contrasted with the observed proportion of the outcome in a particular prediction range. Results for calibration appeared to vary. For the *Received a COVID-19* outcome, the models with only covariates had poor calibration, but the integration of other predictors, especially with elastic net and ridge regression, led to improvements. Similar trends were observed for the *Diagnosed with COVID-19* outcome, though calibration as a whole seemed to be superior.

S14 File (continued)

**Elastic Net Regression Model Penalties – Outcome: Received a COVID-19 Test**				
	Covariates + EHR Variables (Restricted)		Covariates + EHR Variables (Unrestricted)	
Split	Lambda	Alpha	Lambda	Alpha
1	0.005	0.100	0.005	0.100
2	0.030	0.100	0.03	0.100
3	0.005	0.100	0.005	0.100
4	0.005	0.100	0.005	0.100
5	0.005	0.100	0.005	0.100
6	0.005	0.500	0.005	0.300
7	0.005	0.100	0.005	0.100
8	0.005	0.100	0.005	0.100
9	0.005	0.100	0.005	0.100
10	0.005	0.100	0.005	0.100
**Elastic Net Regression Model Penalties – Outcome: Diagnosed with COVID-19**				
	Covariates + EHR Variables (Restricted)		Covariates + EHR Variables (Unrestricted)	
Split	Lambda	Alpha	Lambda	Alpha
1	0.005	0.100	0.005	0.100
2	0.005	0.100	0.005	0.100
3	0.005	0.100	0.005	0.100
4	0.005	0.100	0.005	0.100
5	0.005	0.100	0.005	0.100
6	0.005	0.100	0.005	0.100
7	0.005	0.100	0.005	0.100
8	0.005	0.100	0.005	0.100
9	0.005	0.100	0.005	0.100
10	0.005	0.100	0.005	0.100

Lambda and alpha were selected by five-fold cross-validation on the training set of a single 70/30 train/test split.

S14 File (continued)

**Ridge Regression Model Penalties – Outcome: Received a COVID-19 Test**				
	Covariates + EHR Variables (Restricted)		Covariates + EHR Variables (Unrestricted)	
Split	Lambda	Alpha	Lambda	Alpha
1	0.01	0.000	0.010	0.000
2	0.04	0.000	0.030	0.000
3	0.01	0.000	0.010	0.000
4	0.01	0.000	0.010	0.000
5	0.01	0.000	0.010	0.000
6	0.015	0.000	0.015	0.000
7	0.01	0.000	0.010	0.000
8	0.01	0.000	0.010	0.000
9	0.01	0.000	0.010	0.000
10	0.01	0.000	0.010	0.000
**Ridge Regression Model Penalties – Outcome: Diagnosed with COVID-19**				
	Covariates + EHR Variables (Restricted)		Covariates + EHR Variables (Unrestricted)	
Split	Lambda	Alpha	Lambda	Alpha
1	0.005	0.000	0.005	0.000
2	0.005	0.000	0.005	0.000
3	0.005	0.000	0.005	0.000
4	0.005	0.000	0.005	0.000
5	0.005	0.000	0.005	0.000
6	0.005	0.000	0.005	0.000
7	0.005	0.000	0.005	0.000
8	0.005	0.000	0.005	0.000
9	0.005	0.000	0.005	0.000
10	0.005	0.000	0.005	0.000

Lambda and alpha were selected by five-fold cross-validation on the training set of a single 70/30 train/test split.

**Lasso Regression Model Penalties – Outcome: Received a COVID-19 Test**				
	Covariates + EHR Variables (Restricted)		Covariates + EHR Variables (Unrestricted)	
Split	Lambda	Alpha	Lambda	Alpha
1	0.005	1.000	0.005	1.000
2	0.005	1.000	0.005	1.000
3	0.005	1.000	0.005	1.000
4	0.005	1.000	0.005	1.000
5	0.005	1.000	0.005	1.000
6	0.005	1.000	0.005	1.000
7	0.005	1.000	0.005	1.000
8	0.005	1.000	0.005	1.000
9	0.005	1.000	0.005	1.000
10	0.005	1.000	0.005	1.000
**Lasso Regression Model Penalties – Outcome: Diagnosed with COVID-19**				
	Covariates + EHR Variables (Restricted)		Covariates + EHR Variables (Unrestricted)	
Split	Lambda	Alpha	Lambda	Alpha
1	0.005	1.000	0.005	1.000
2	0.005	1.000	0.005	1.000
3	0.005	1.000	0.005	1.000
4	0.005	1.000	0.005	1.000
5	0.005	1.000	0.005	1.000
6	0.005	1.000	0.005	1.000
7	0.005	1.000	0.005	1.000
8	0.005	1.000	0.005	1.000
9	0.005	1.000	0.005	1.000
10	0.005	1.000	0.005	1.000

Lambda and alpha were selected by five-fold cross-validation on the training set of a single 70/30 train/test split.

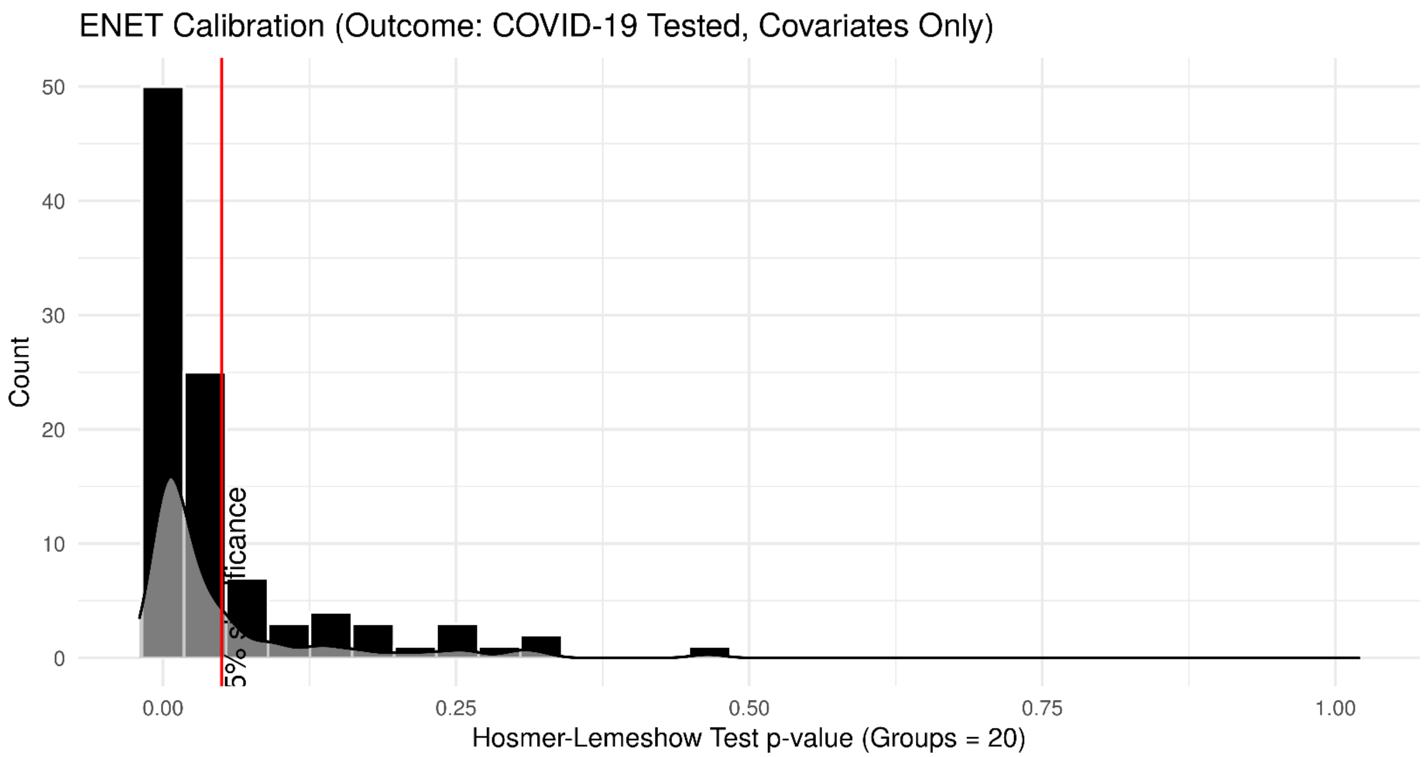

We plotted the p-values from conducting a Hosmer-Lemeshow goodness of fit test on all 100 train/test splits of the model evaluation procedure. Models tending to have poor calibration would show large numbers of p-values below the statistical significance threshold of 0.05.

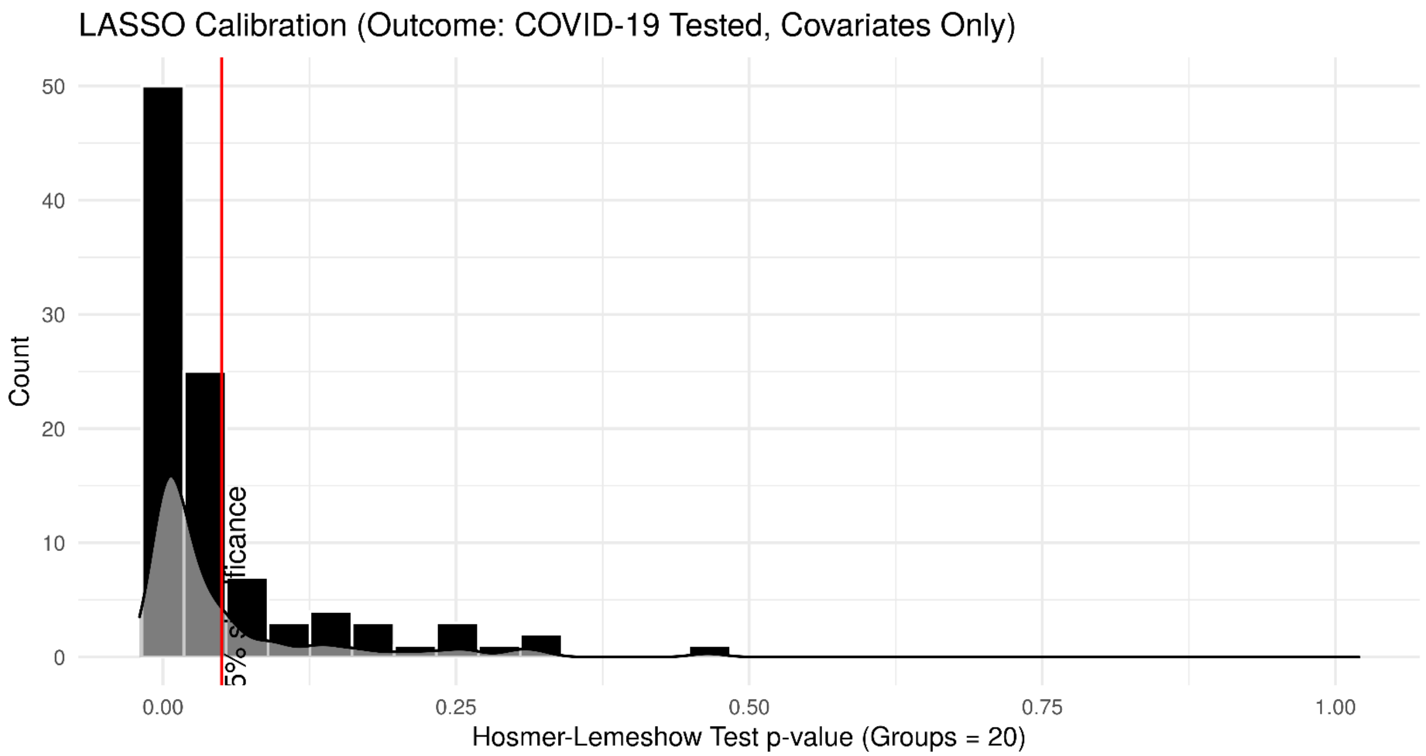

We plotted the p-values from conducting a Hosmer-Lemeshow goodness of fit test on all 100 train/test splits of the model evaluation procedure. Models tending to have poor calibration would show large numbers of p-values below the statistical significance threshold of 0.05.

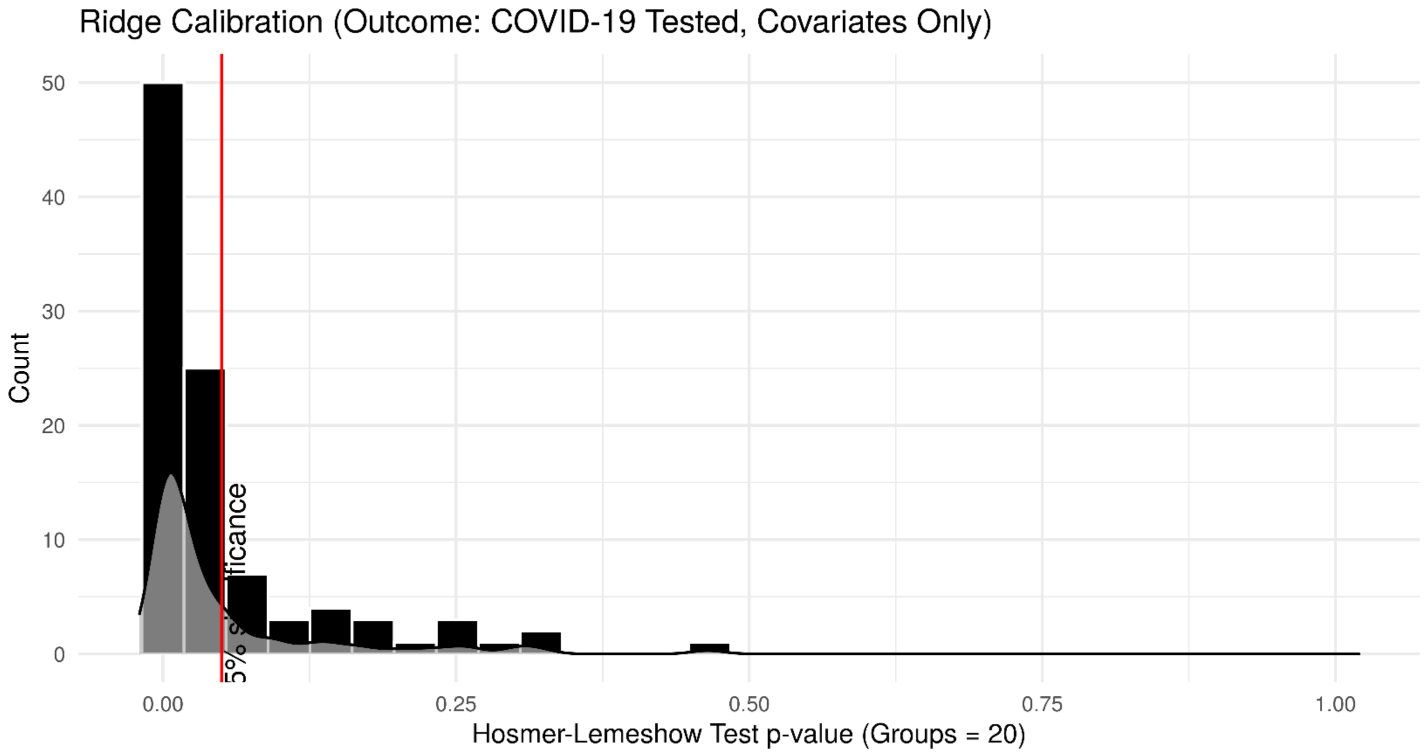

We plotted the p-values from conducting a Hosmer-Lemeshow goodness of fit test on all 100 train/test splits of the model evaluation procedure. Models tending to have poor calibration would show large numbers of p-values below the statistical significance threshold of 0.05.

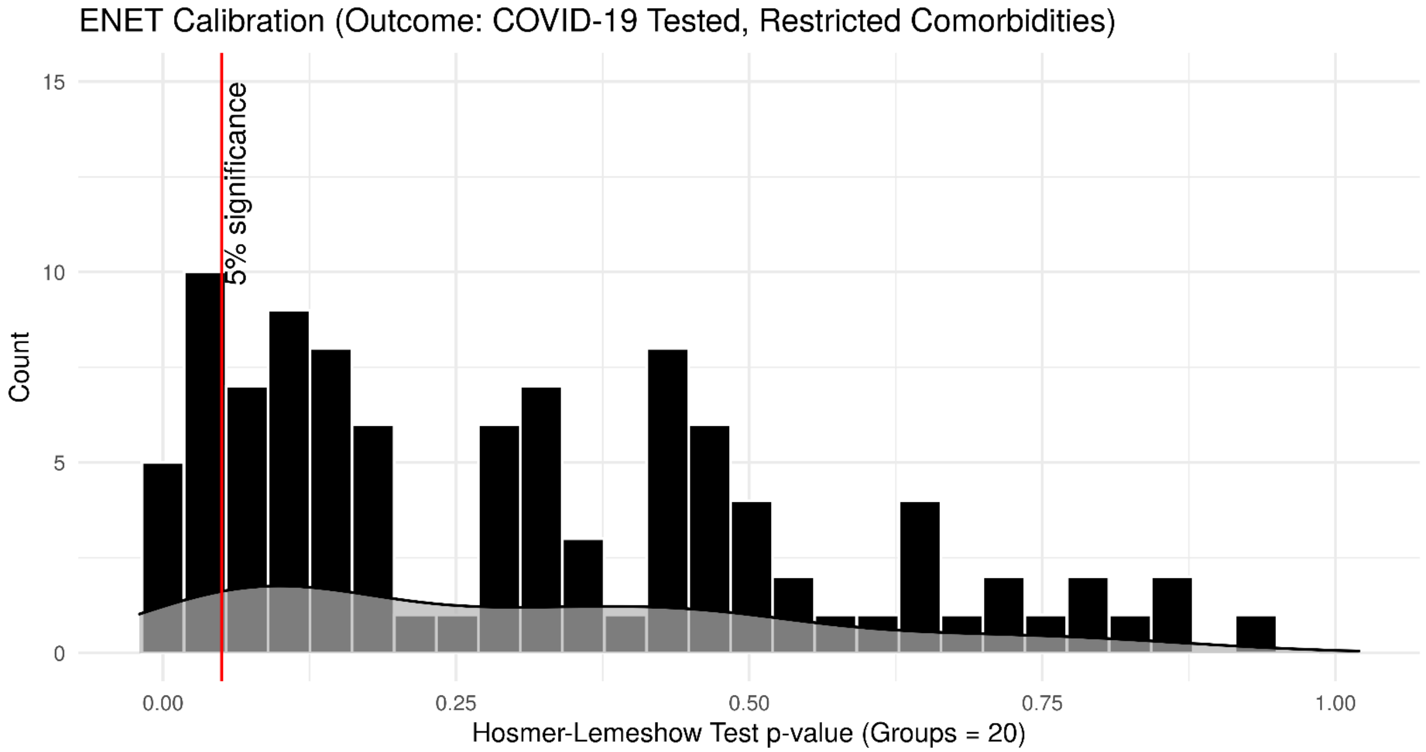

We plotted the p-values from conducting a Hosmer-Lemeshow goodness of fit test on all 100 train/test splits of the model evaluation procedure. Models tending to have poor calibration would show large numbers of p-values below the statistical significance threshold of 0.05.

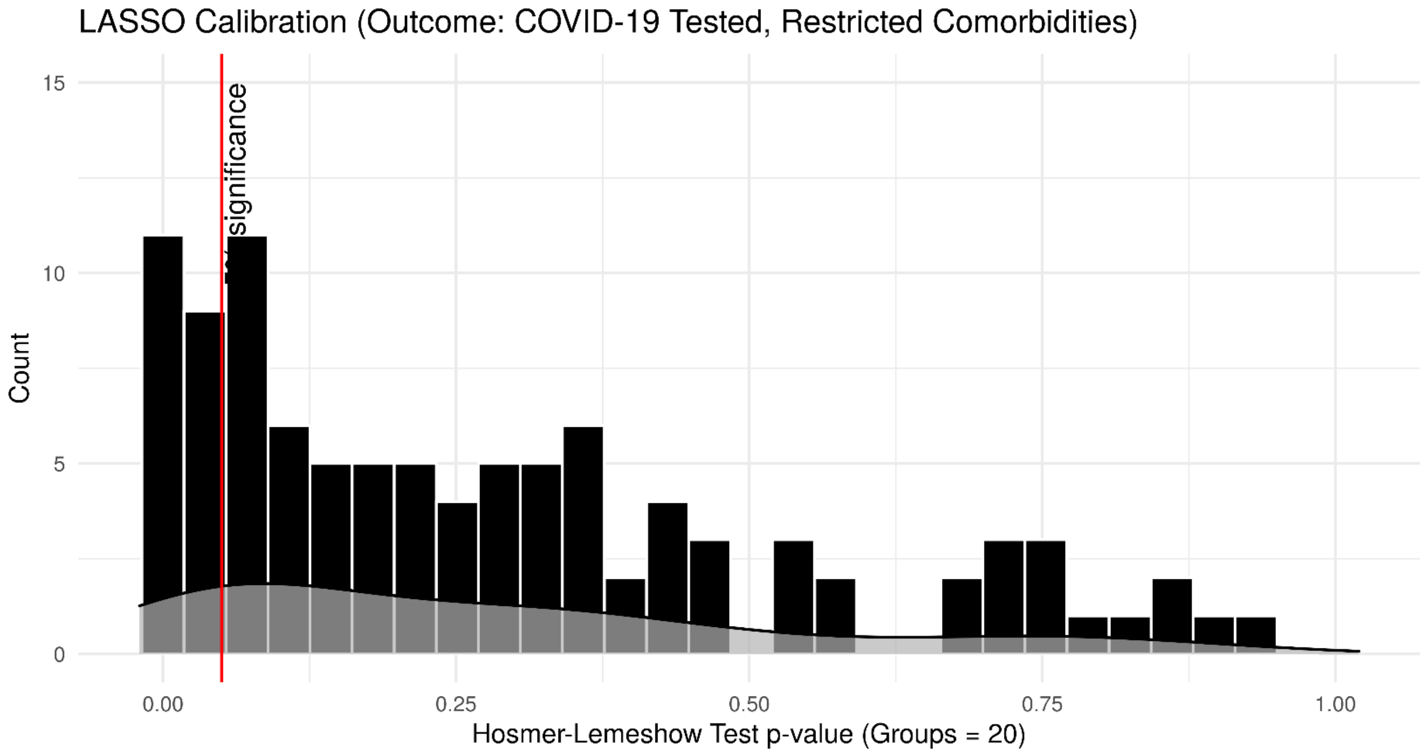

We plotted the p-values from conducting a Hosmer-Lemeshow goodness of fit test on all 100 train/test splits of the model evaluation procedure. Models tending to have poor calibration would show large numbers of p-values below the statistical significance threshold of 0.05.

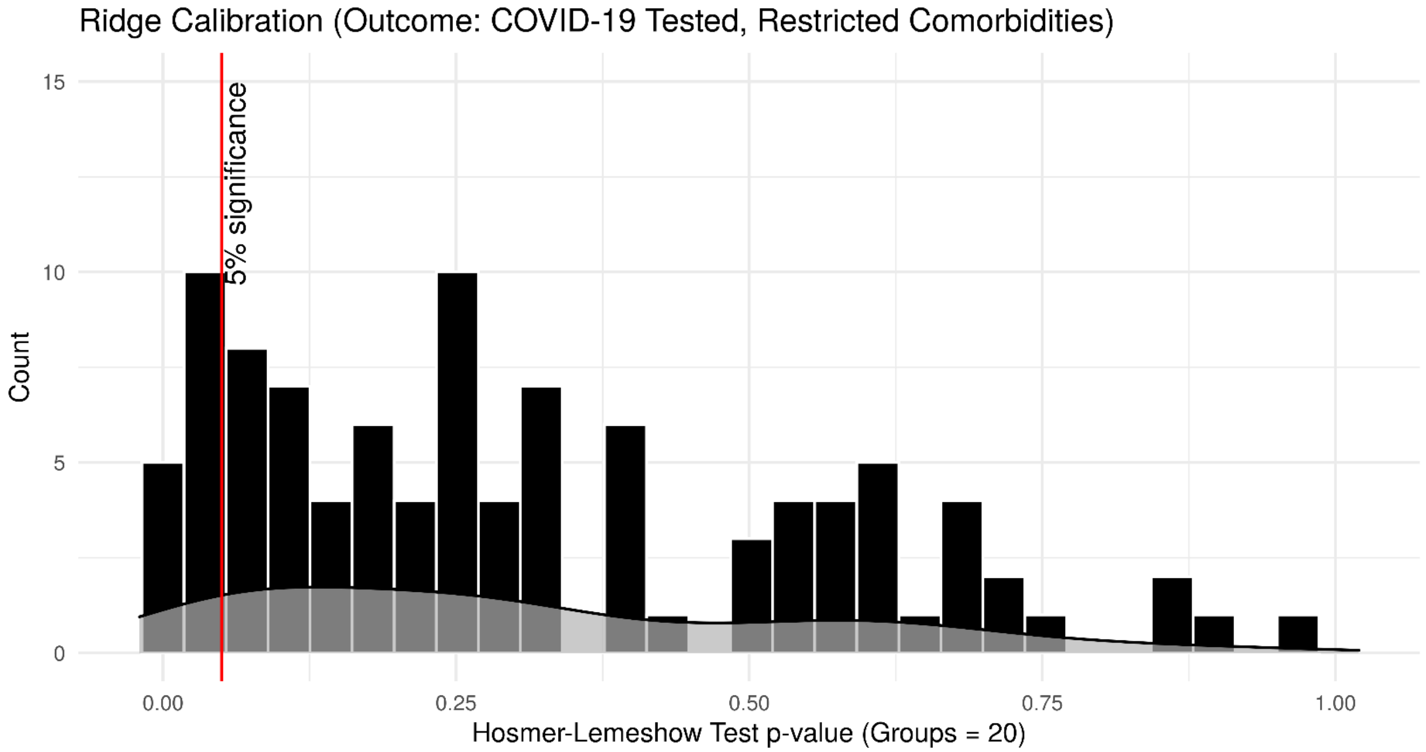

We plotted the p-values from conducting a Hosmer-Lemeshow goodness of fit test on all 100 train/test splits of the model evaluation procedure. Models tending to have poor calibration would show large numbers of p-values below the statistical significance threshold of 0.05.

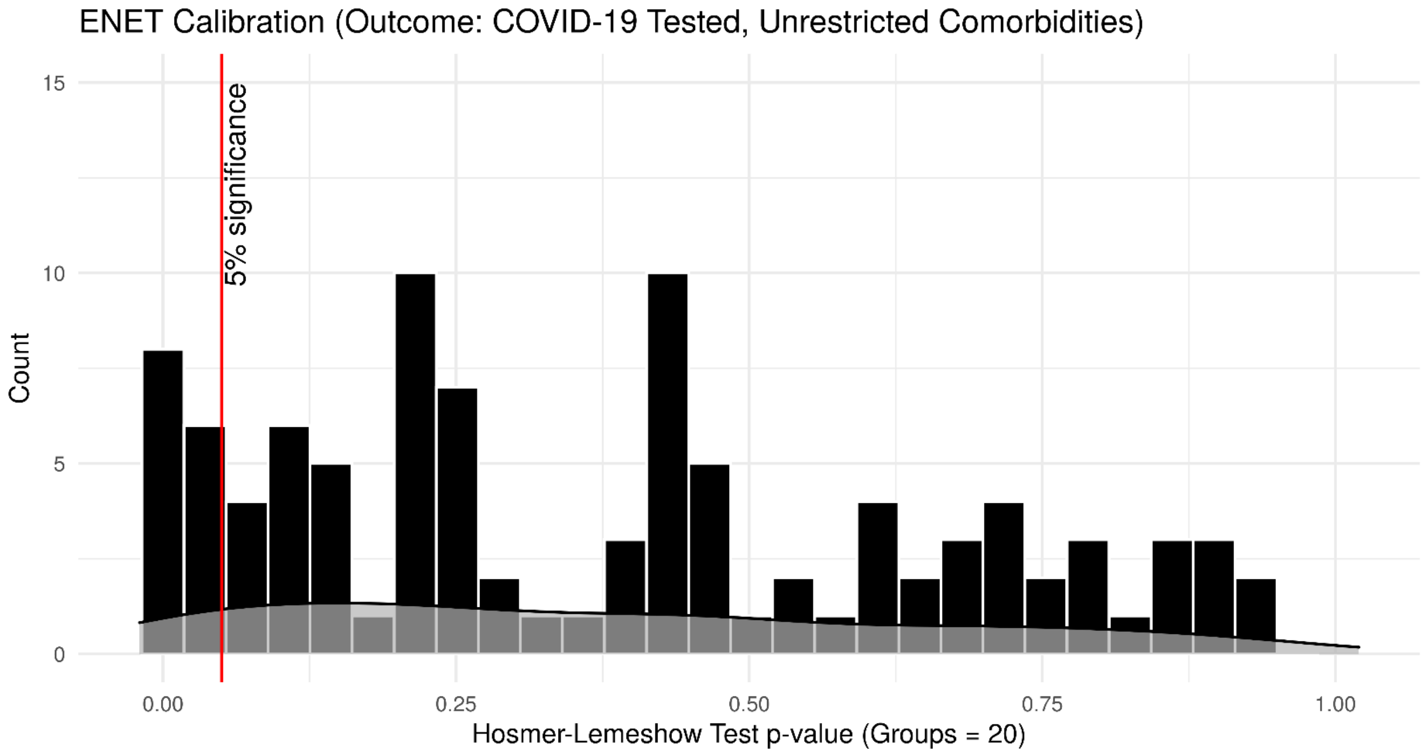

We plotted the p-values from conducting a Hosmer-Lemeshow goodness of fit test on all 100 train/test splits of the model evaluation procedure. Models tending to have poor calibration would show large numbers of p-values below the statistical significance threshold of 0.05.

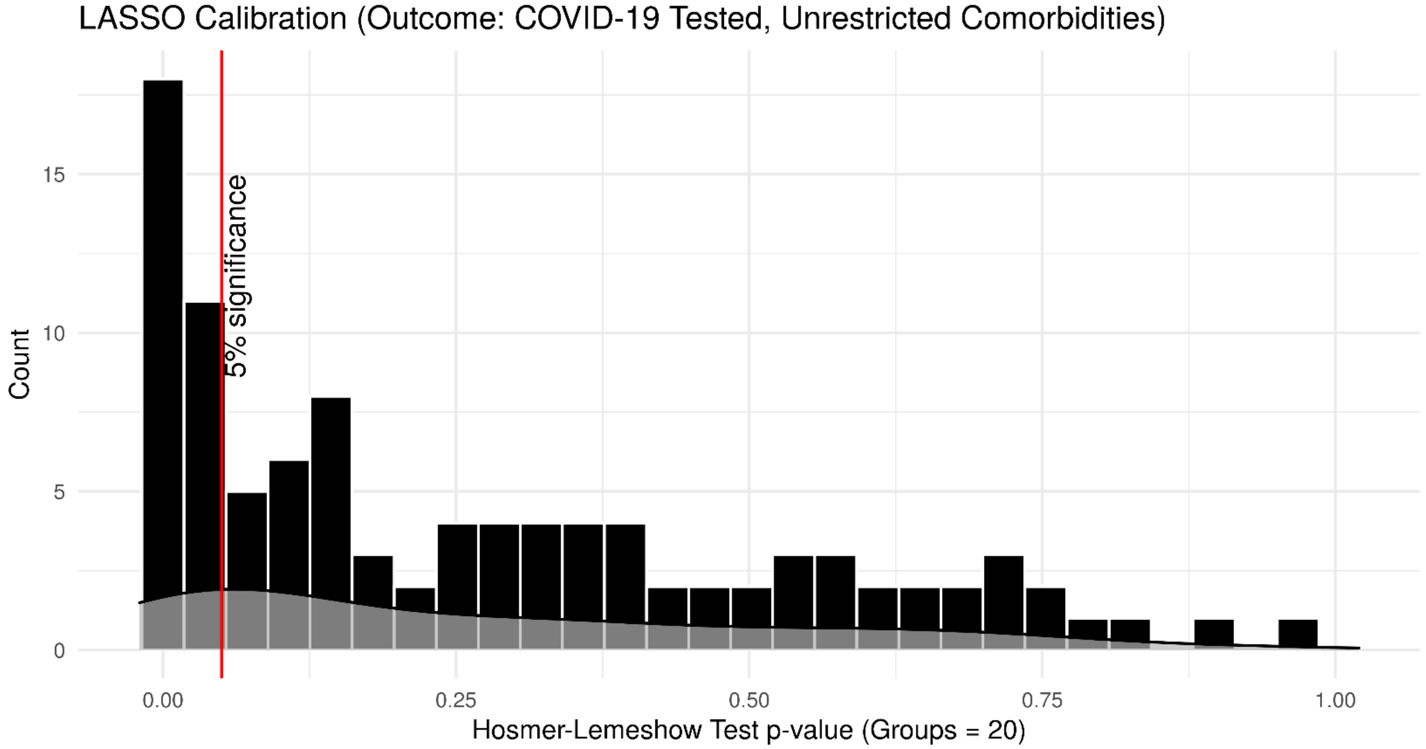

We plotted the p-values from conducting a Hosmer-Lemeshow goodness of fit test on all 100 train/test splits of the model evaluation procedure. Models tending to have poor calibration would show large numbers of p-values below the statistical significance threshold of 0.05.

S14 File (continued)

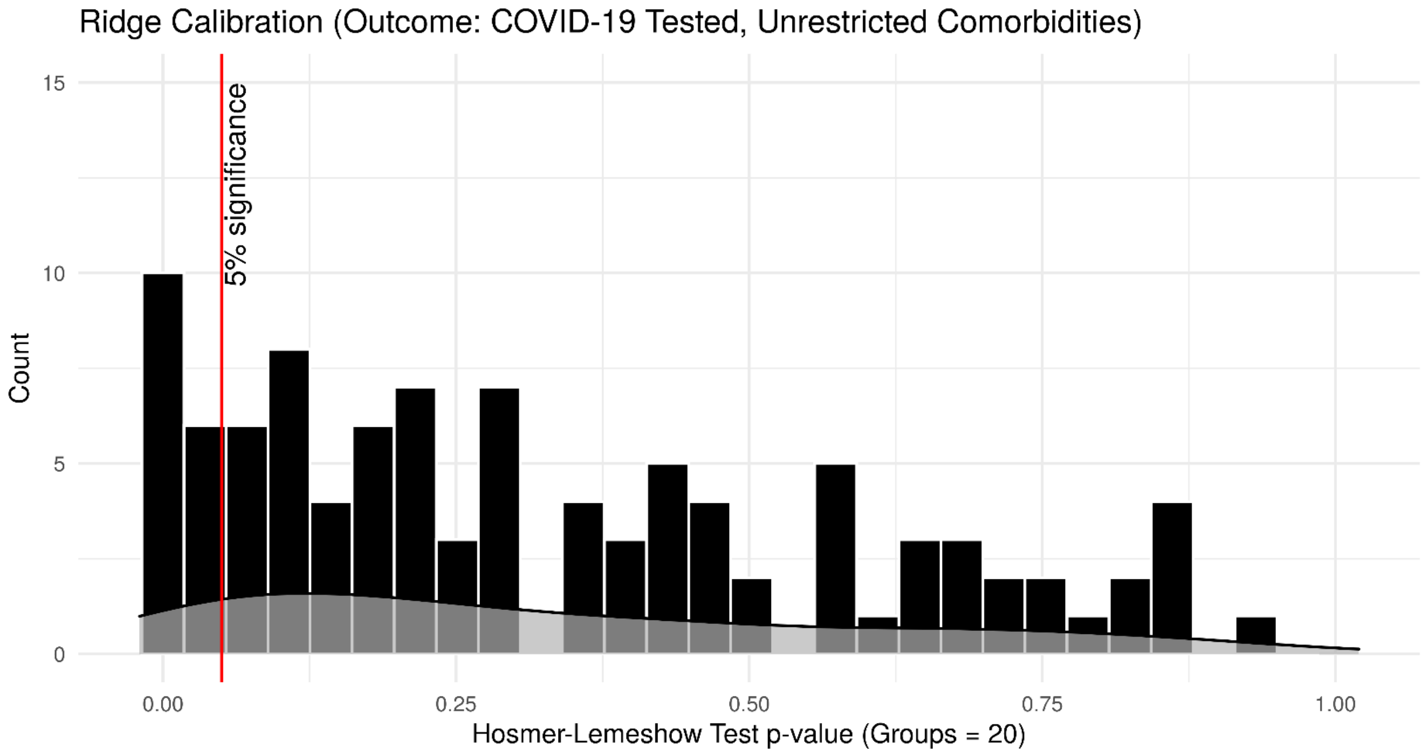

We plotted the p-values from conducting a Hosmer-Lemeshow goodness of fit test on all 100 train/test splits of the model evaluation procedure. Models tending to have poor calibration would show large numbers of p-values below the statistical significance threshold of 0.05.

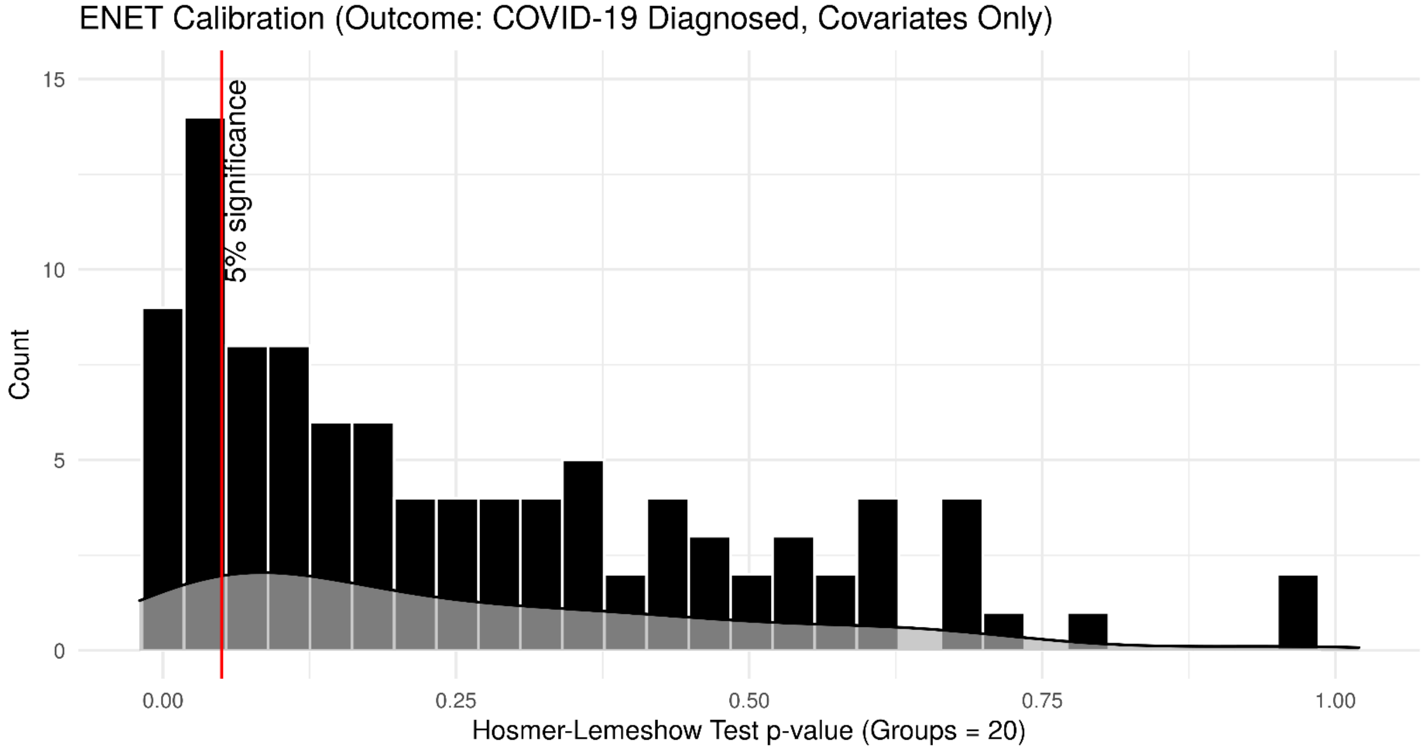

We plotted the p-values from conducting a Hosmer-Lemeshow goodness of fit test on all 100 train/test splits of the model evaluation procedure. Models tending to have poor calibration would show large numbers of p-values below the statistical significance threshold of 0.05.

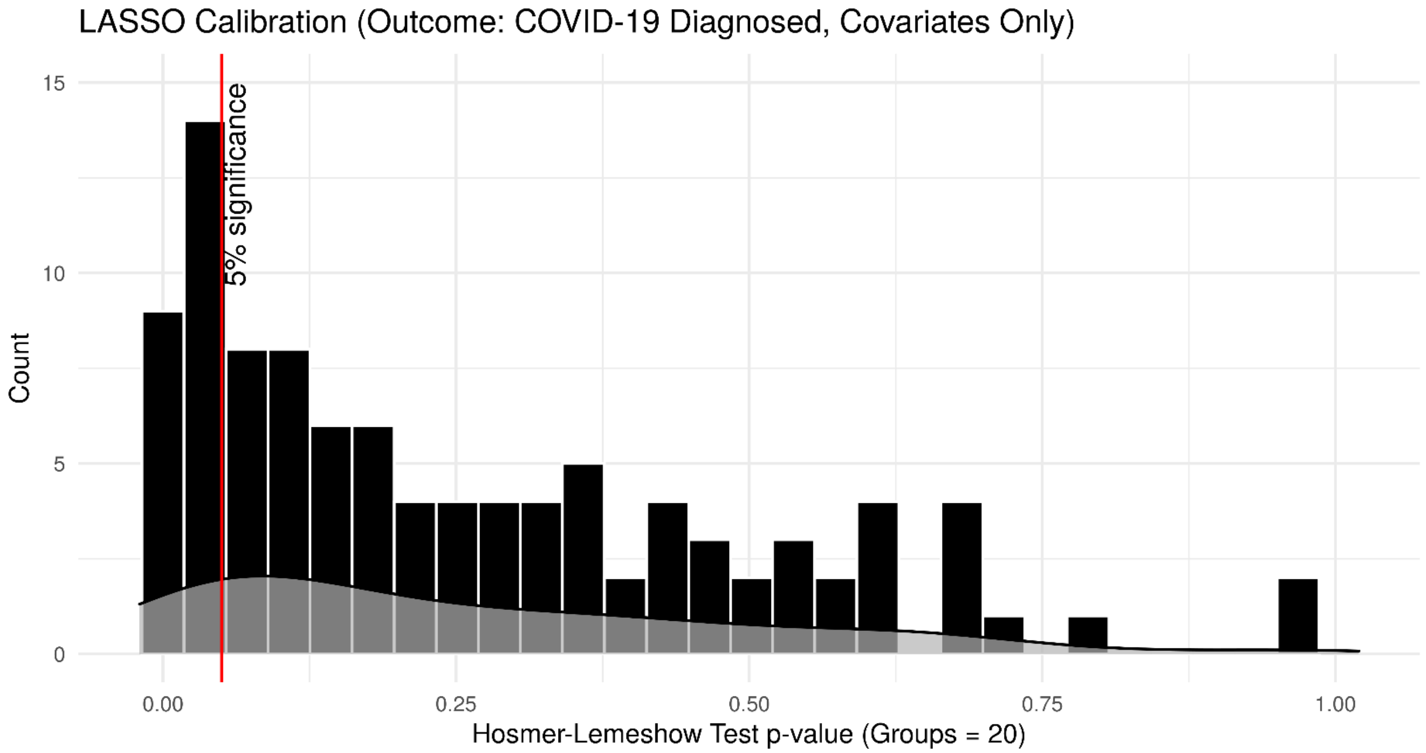

We plotted the p-values from conducting a Hosmer-Lemeshow goodness of fit test on all 100 train/test splits of the model evaluation procedure. Models tending to have poor calibration would show large numbers of p-values below the statistical significance threshold of 0.05.

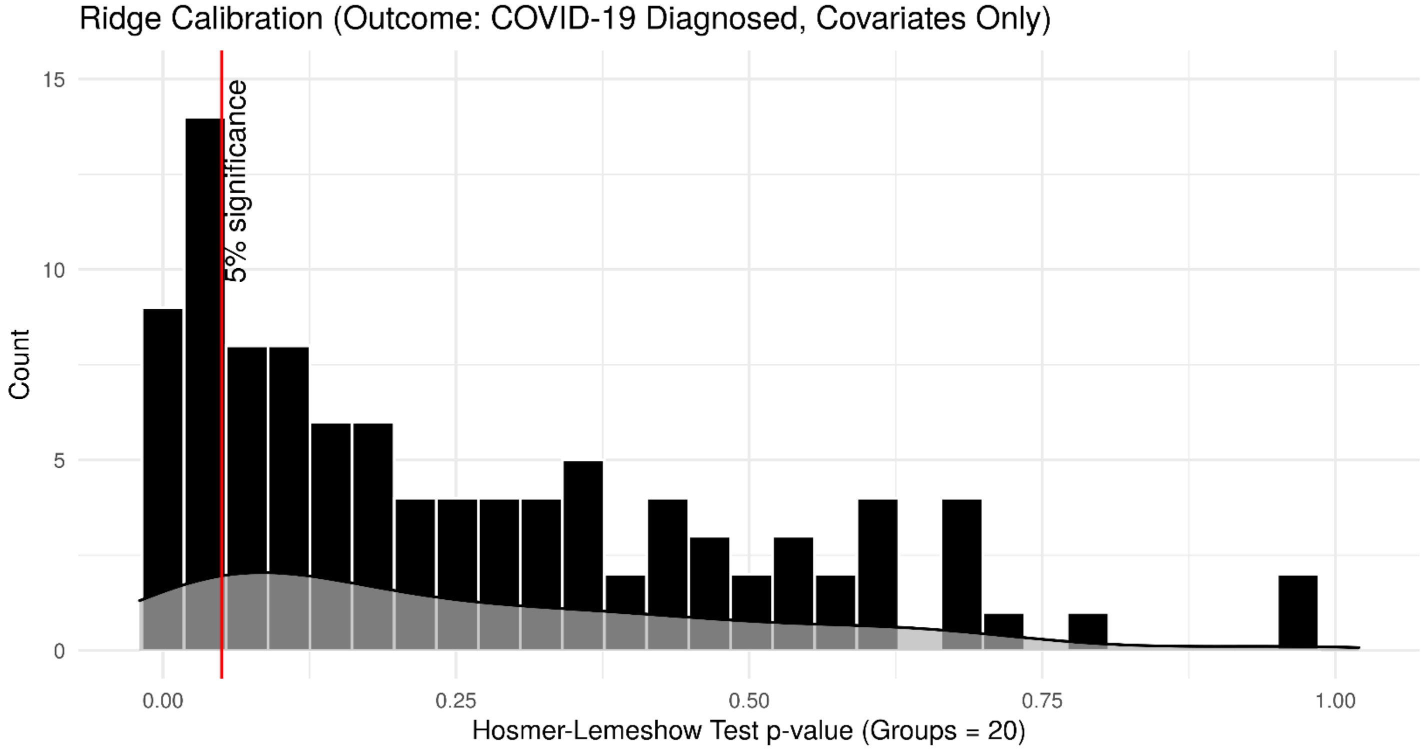

We plotted the p-values from conducting a Hosmer-Lemeshow goodness of fit test on all 100 train/test splits of the model evaluation procedure. Models tending to have poor calibration would show large numbers of p-values below the statistical significance threshold of 0.05.

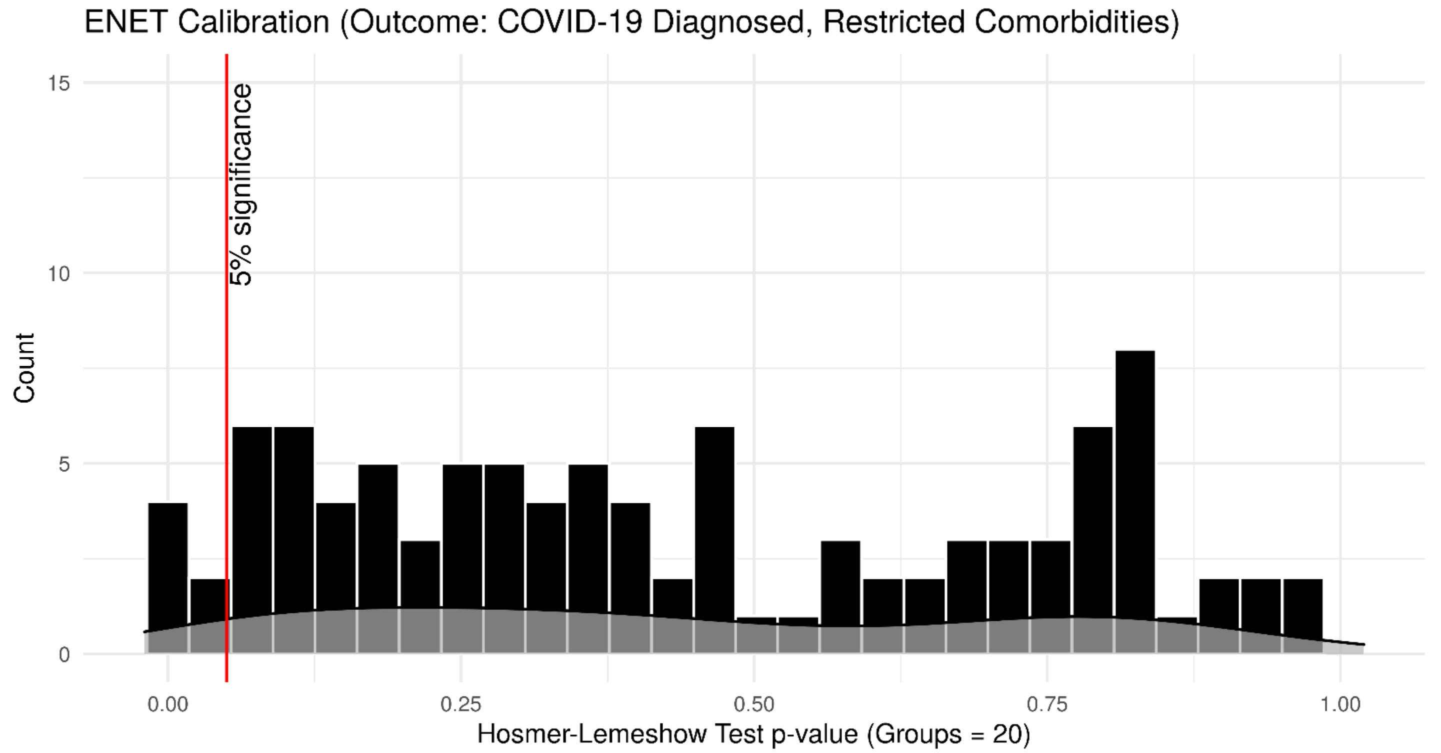

We plotted the p-values from conducting a Hosmer-Lemeshow goodness of fit test on all 100 train/test splits of the model evaluation procedure. Models tending to have poor calibration would show large numbers of p-values below the statistical significance threshold of 0.05.

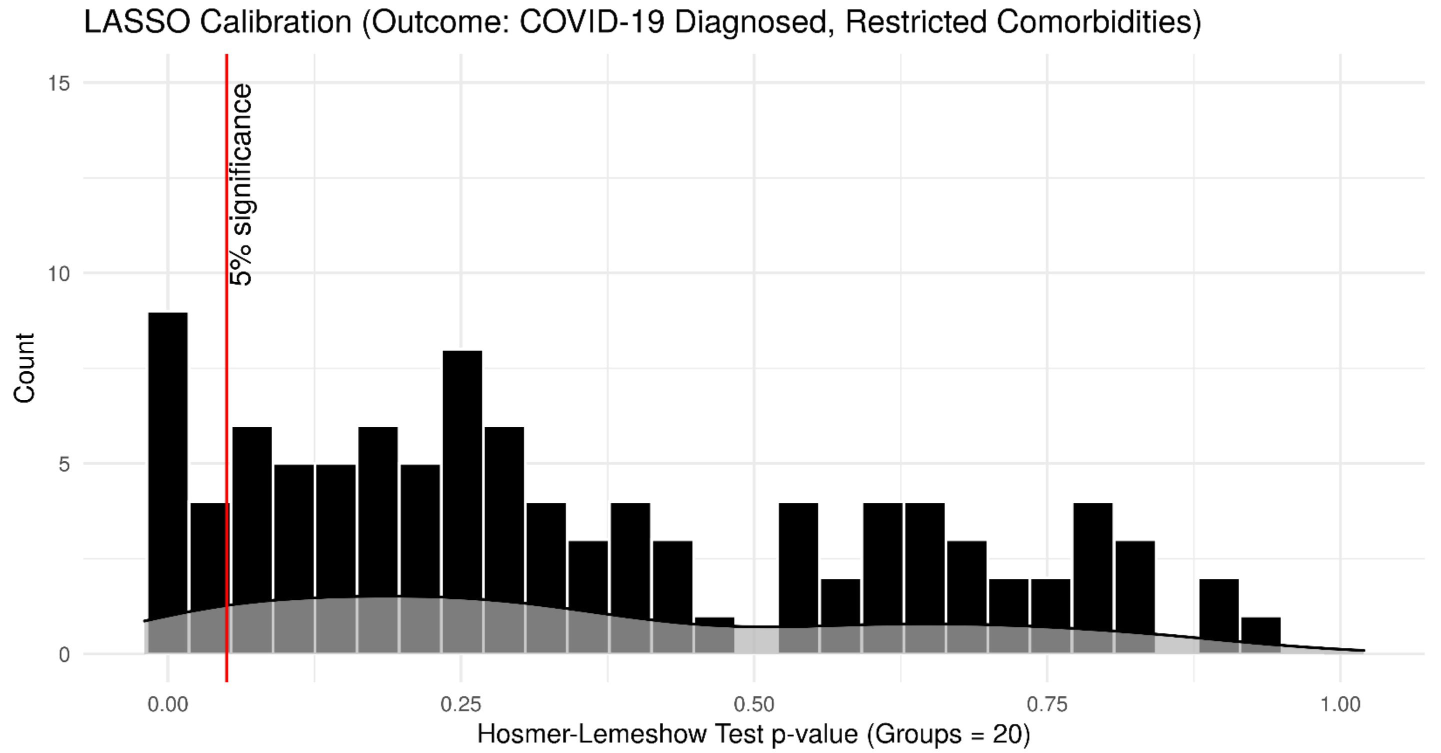

We plotted the p-values from conducting a Hosmer-Lemeshow goodness of fit test on all 100 train/test splits of the model evaluation procedure. Models tending to have poor calibration would show large numbers of p-values below the statistical significance threshold of 0.05.

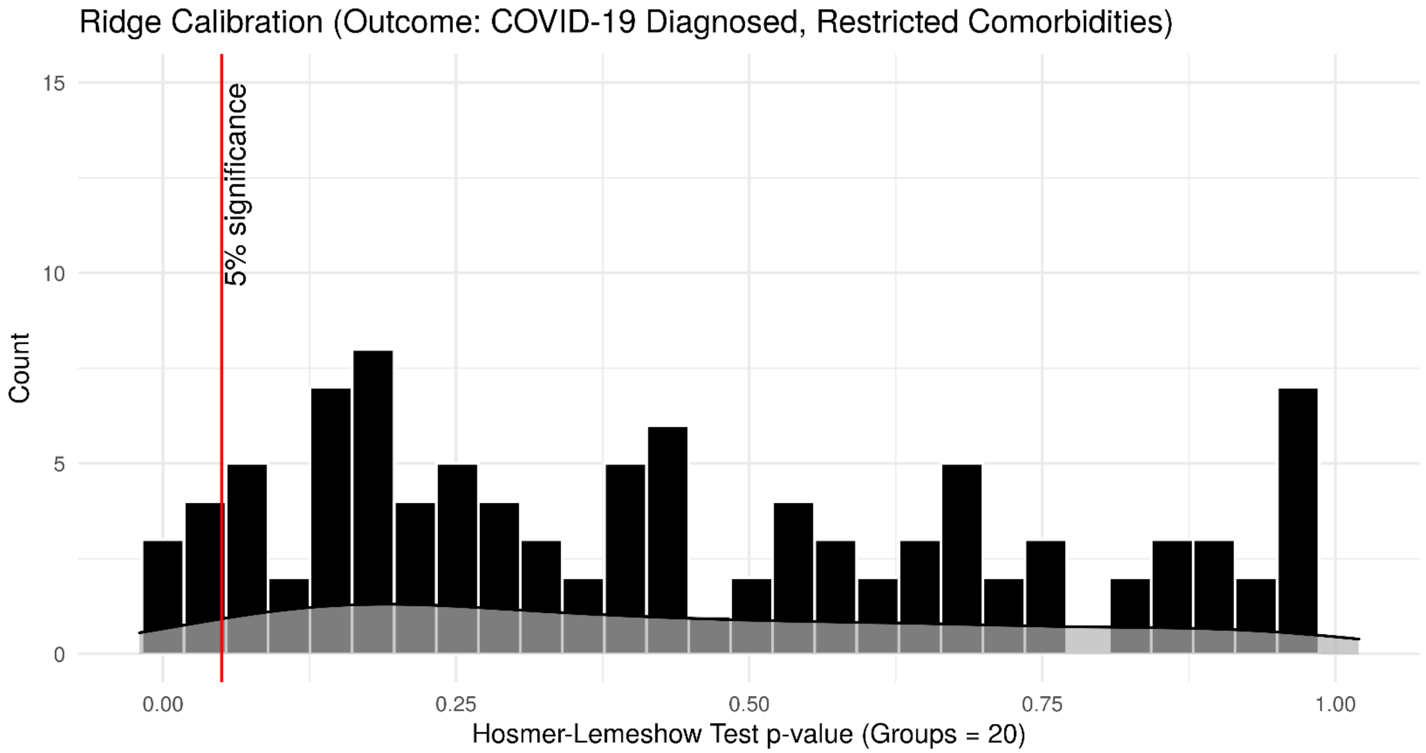

We plotted the p-values from conducting a Hosmer-Lemeshow goodness of fit test on all 100 train/test splits of the model evaluation procedure. Models tending to have poor calibration would show large numbers of p-values below the statistical significance threshold of 0.05.

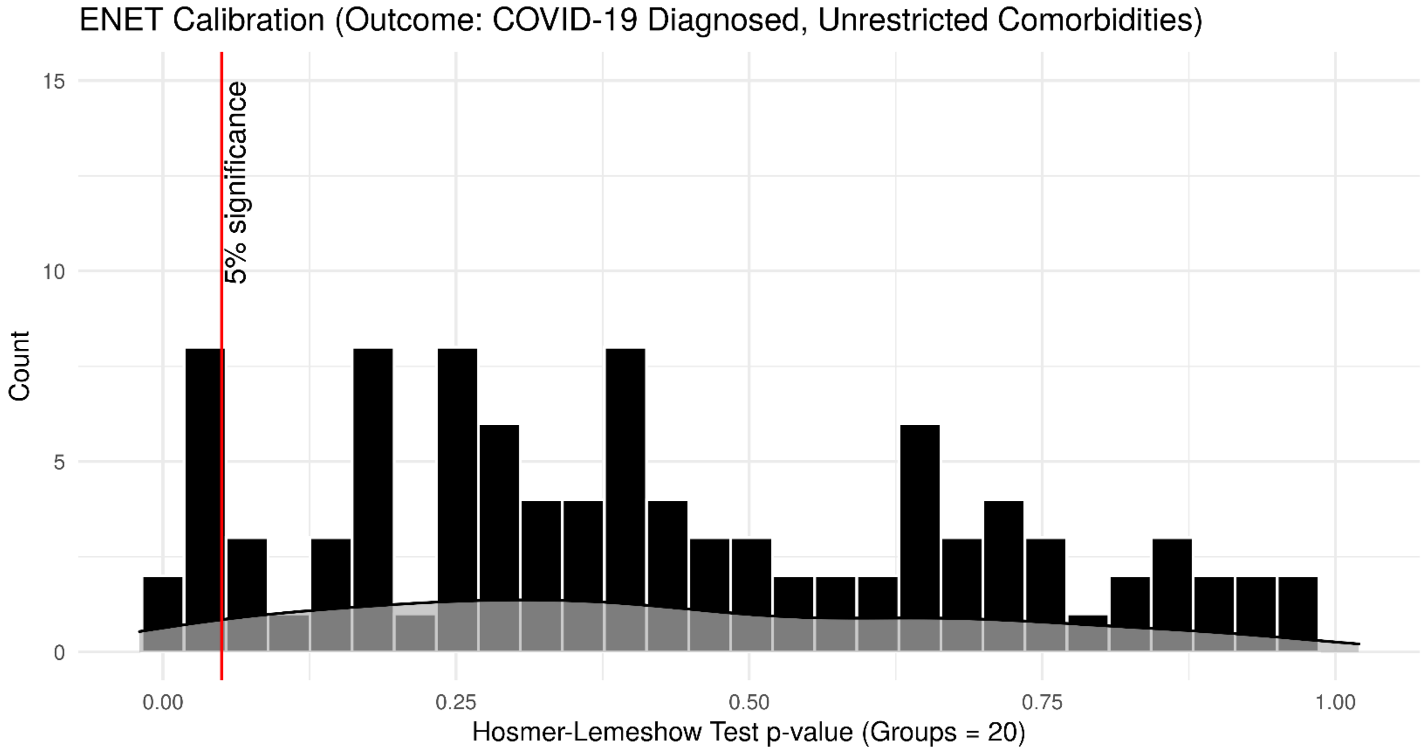

We plotted the p-values from conducting a Hosmer-Lemeshow goodness of fit test on all 100 train/test splits of the model evaluation procedure. Models tending to have poor calibration would show large numbers of p-values below the statistical significance threshold of 0.05.

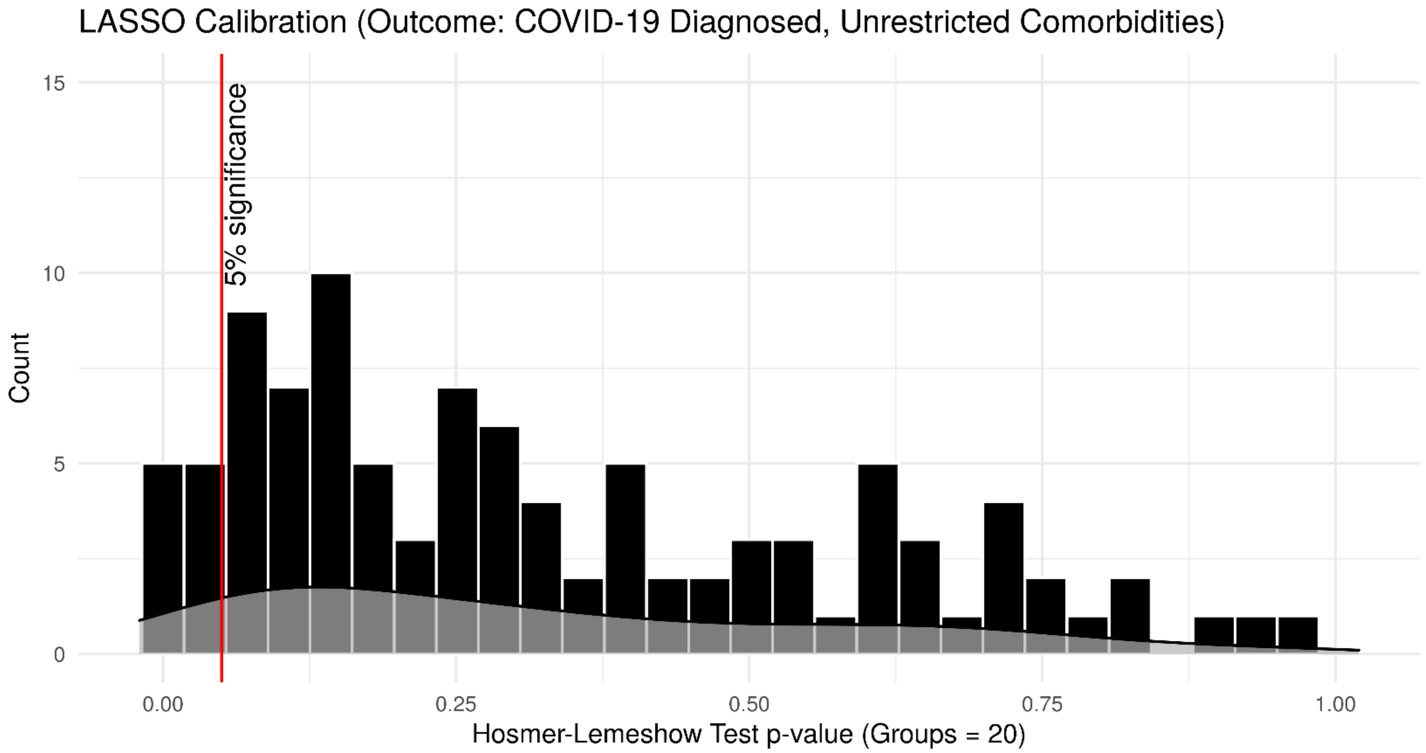

We plotted the p-values from conducting a Hosmer-Lemeshow goodness of fit test on all 100 train/test splits of the model evaluation procedure. Models tending to have poor calibration would show large numbers of p-values below the statistical significance threshold of 0.05.

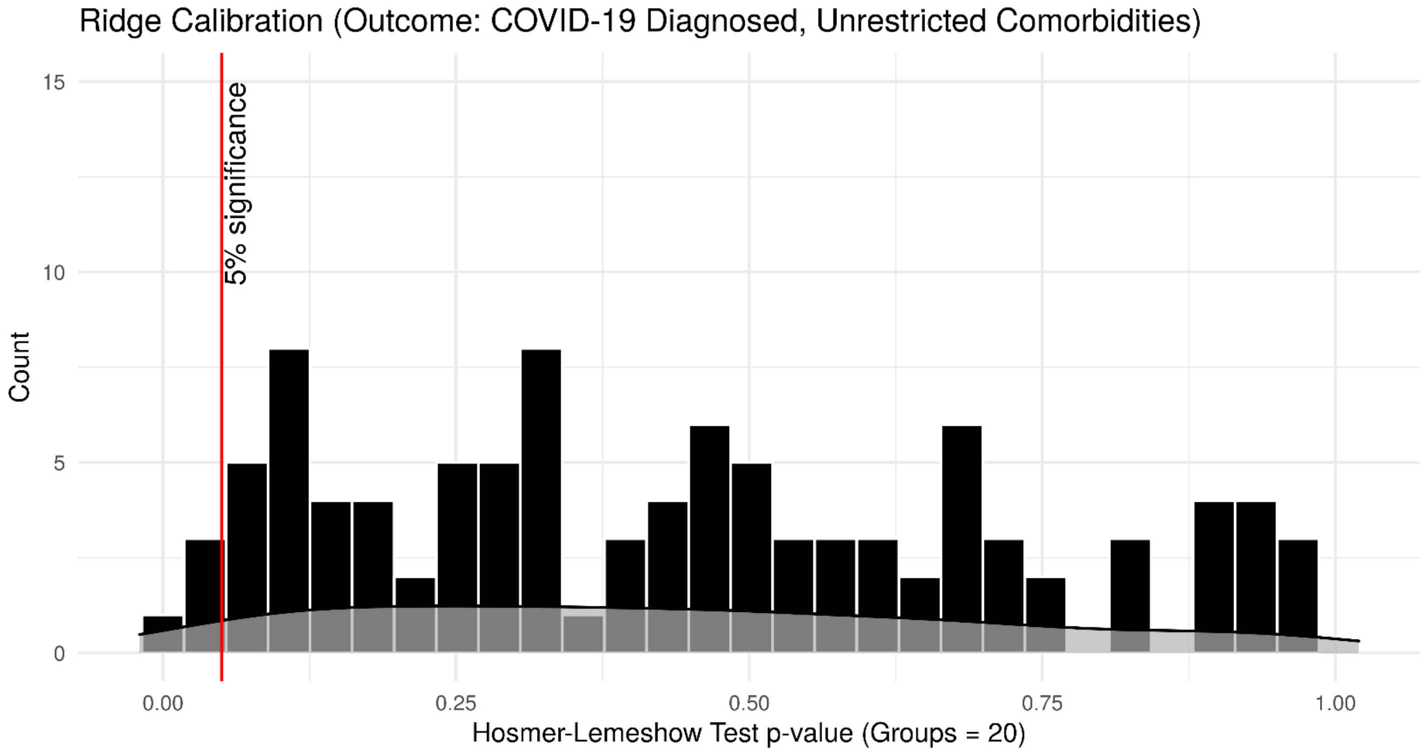

We plotted the p-values from conducting a Hosmer-Lemeshow goodness of fit test on all 100 train/test splits of the model evaluation procedure. Models tending to have poor calibration would show large numbers of p-values below the statistical significance threshold of 0.05.
