# Supplementary material for: Assessing the added value of linking electronic health records to improve the prediction of self-reported COVID-19 testing and diagnosis
Source: PLoS One. 2022 Jul 25;17(7):e0269017. doi: 10.1371/journal.pone.0269017 (PMC9312965; doi:10.1371/journal.pone.0269017)
Supplement: S3 File — (PDF) [file pone.0269017.s016.pdf]

S15 File. Calibration Plots for Survey-Based Models of COVID-19 Outcomes

Calibration

We assessed the calibration of our models with the construction of two plots. The first set of plots show the distribution of Hosmer-Lemeshow test p-values for all 100 training-test splits. A p-value less than 0.05 suggests poor calibration. The second set of plots using only the first training-test split and show the predicted probabilities of each outcome contrasted with the observed proportion of the outcome in a particular prediction range. Models for the outcome *Received a COVID-19 Test* in general appeared to be well-calibrated. The calibration plots from the first train/test split show that the observed risk for groups of respondents was highly similar to the predicted risk, and the Hosmer-Lemeshow p-values from all 100 splits were infrequently statistically significant. Calibration was weaker for the outcome *Diagnosed with COVID-19*, as the relationship between observed risk and predicted risk was less linear, and the distributions of Hosmer-Lemeshow test p-values tended to have spikes below 0.05. Models of the *Self-Diagnosed with COVID-19* outcome showed especially poor calibration per both sets of plots.

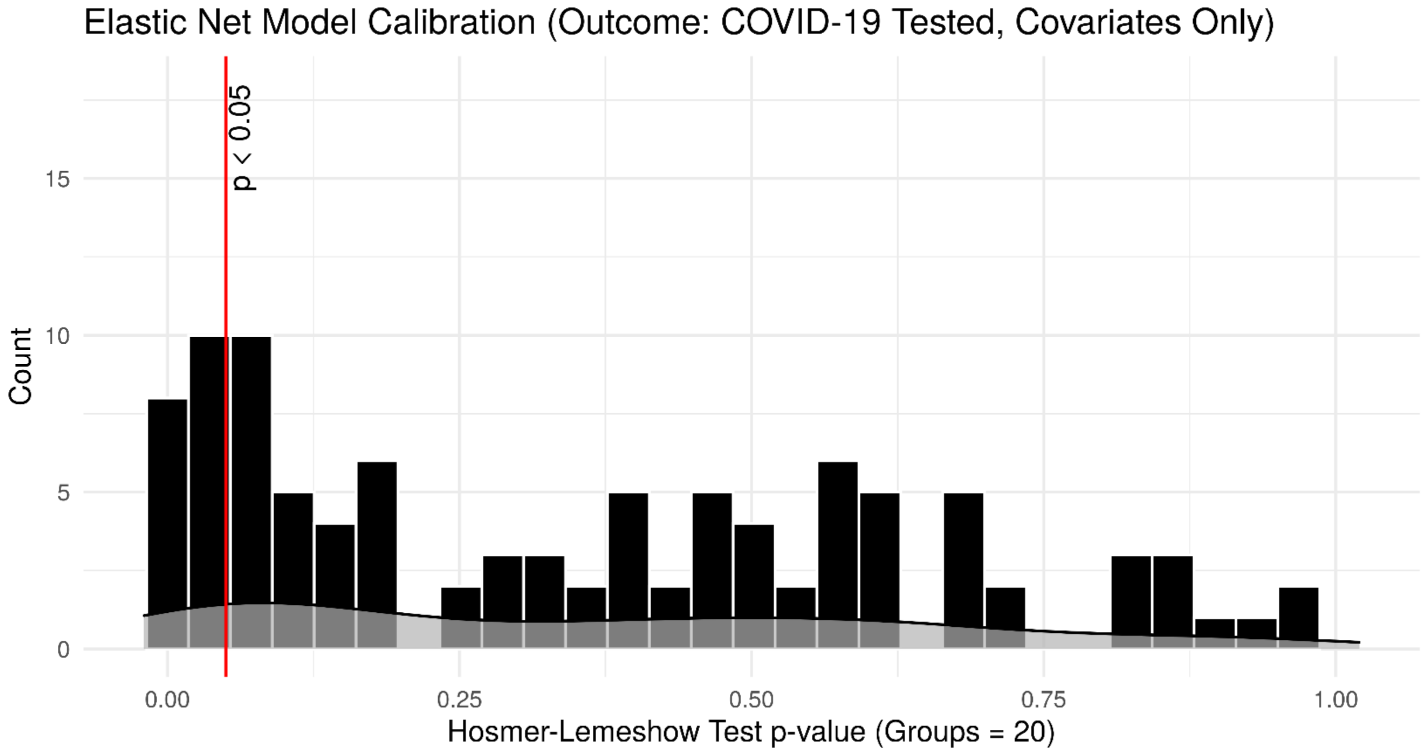

We plotted the p-values from conducting a Hosmer-Lemeshow goodness of fit test on all 100 train/test splits of the model evaluation procedure. Models tending to have poor calibration would show large numbers of p-values below the statistical

significance threshold of 0.05.

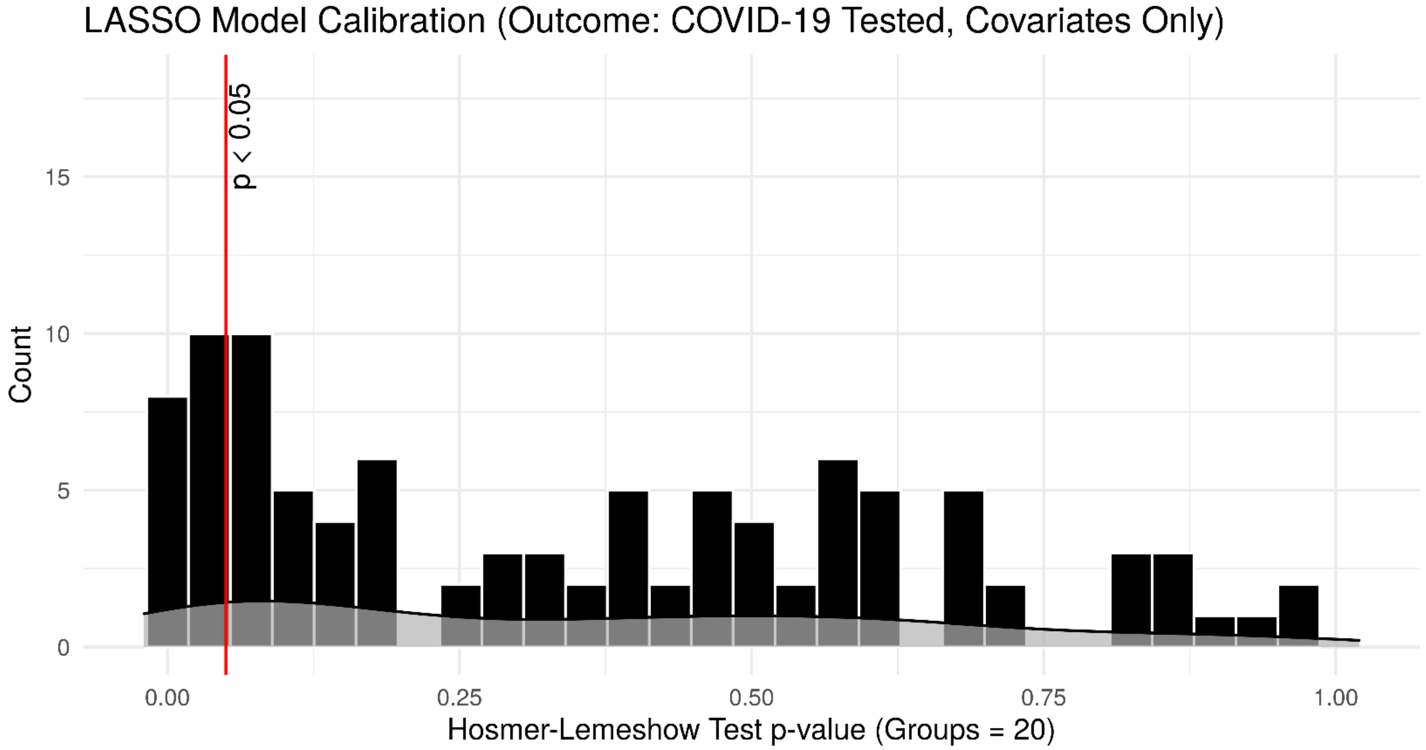

We plotted the p-values from conducting a Hosmer-Lemeshow goodness of fit test on all 100 train/test splits of the model evaluation procedure. Models tending to have poor calibration would show large numbers of p-values below the statistical significance threshold of 0.05.

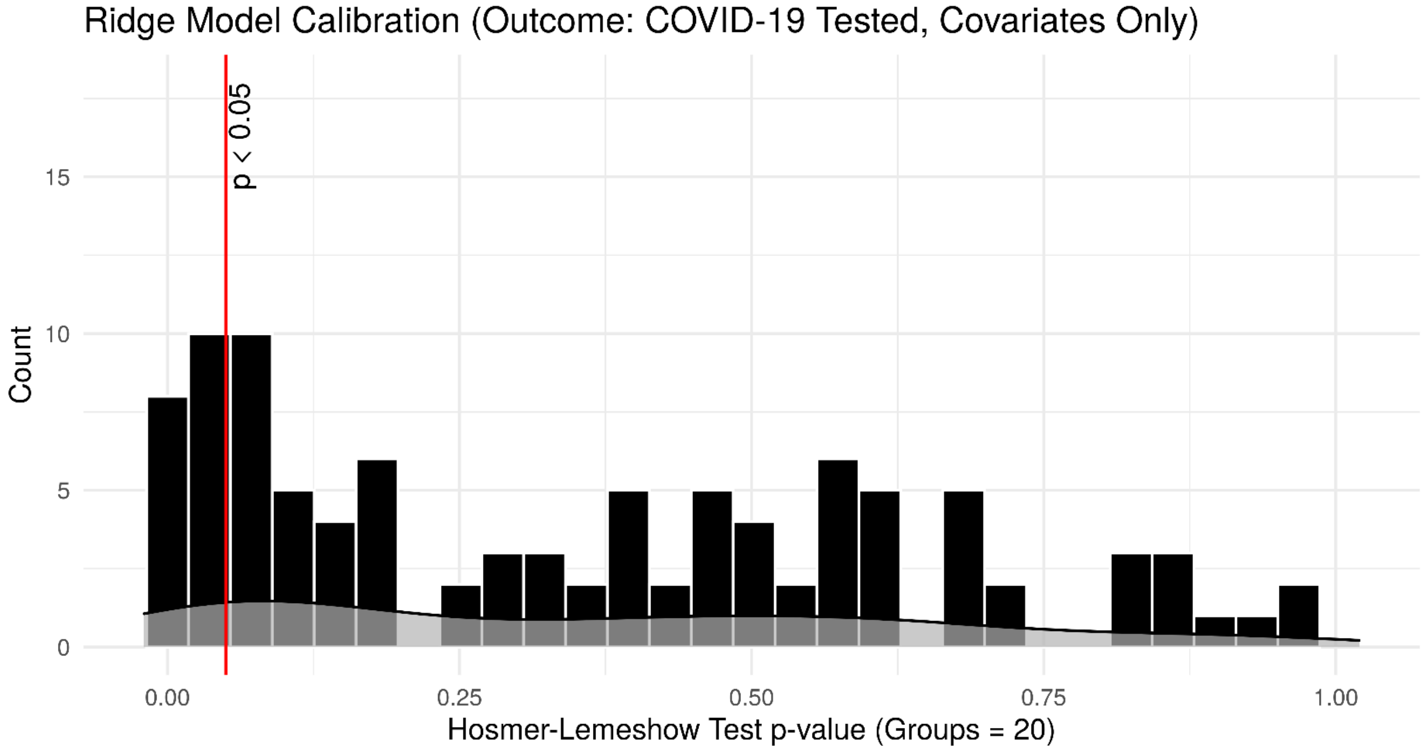

We plotted the p-values from conducting a Hosmer-Lemeshow goodness of fit test on all 100 train/test splits of the model evaluation procedure. Models tending to have poor calibration would show large numbers of p-values below the statistical significance threshold of 0.05.

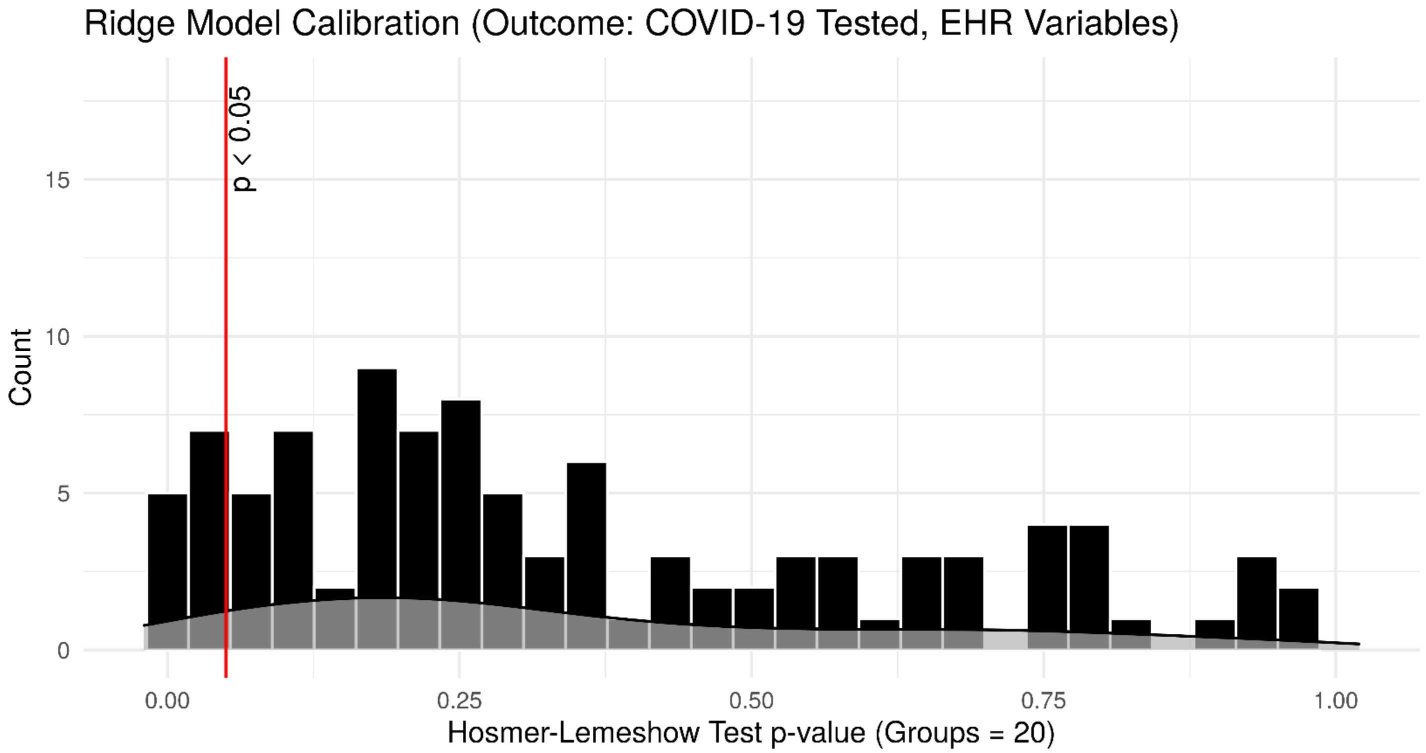

We plotted the p-values from conducting a Hosmer-Lemeshow goodness of fit test on all 100 train/test splits of the model evaluation procedure. Models tending to have poor calibration would show large numbers of p-values below the statistical significance threshold of 0.05.

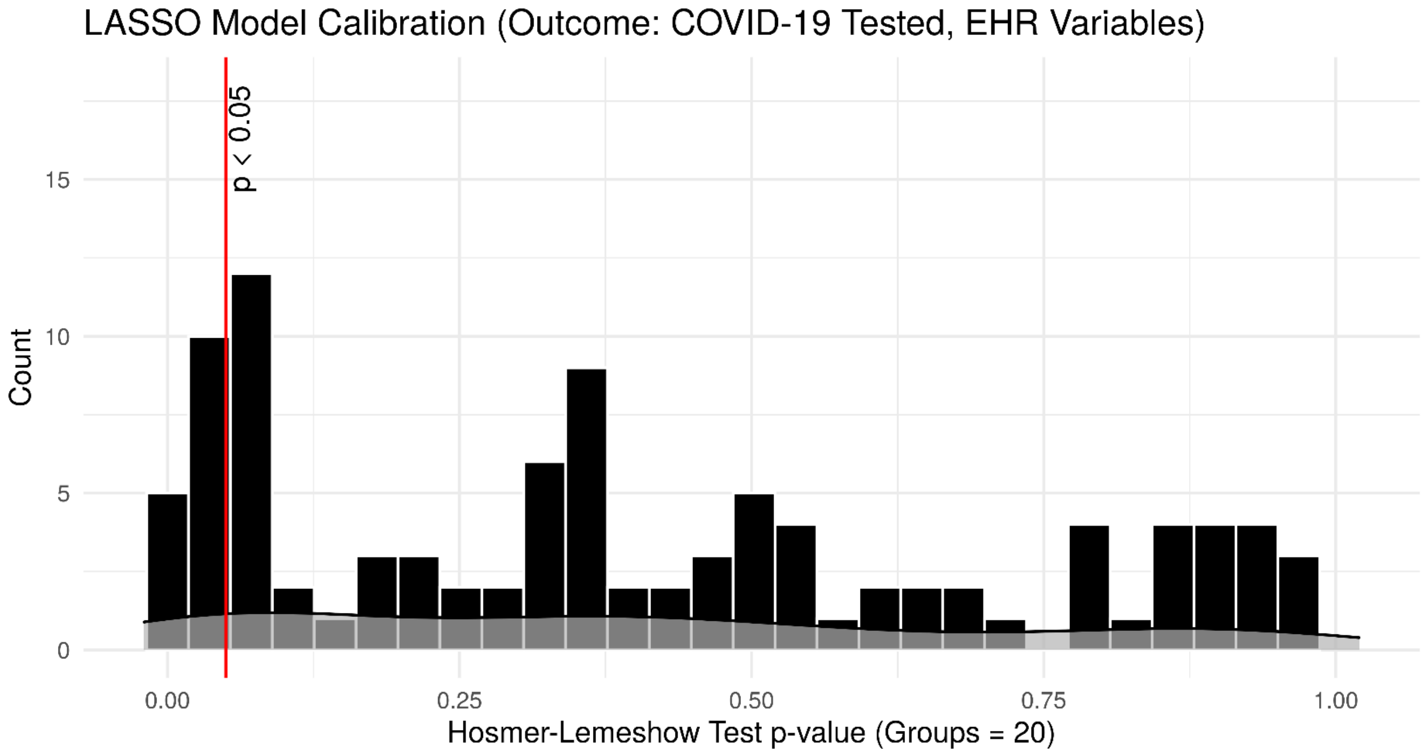

We plotted the p-values from conducting a Hosmer-Lemeshow goodness of fit test on all 100 train/test splits of the model evaluation procedure. Models tending to have poor calibration would show large numbers of p-values below the statistical significance threshold of 0.05.

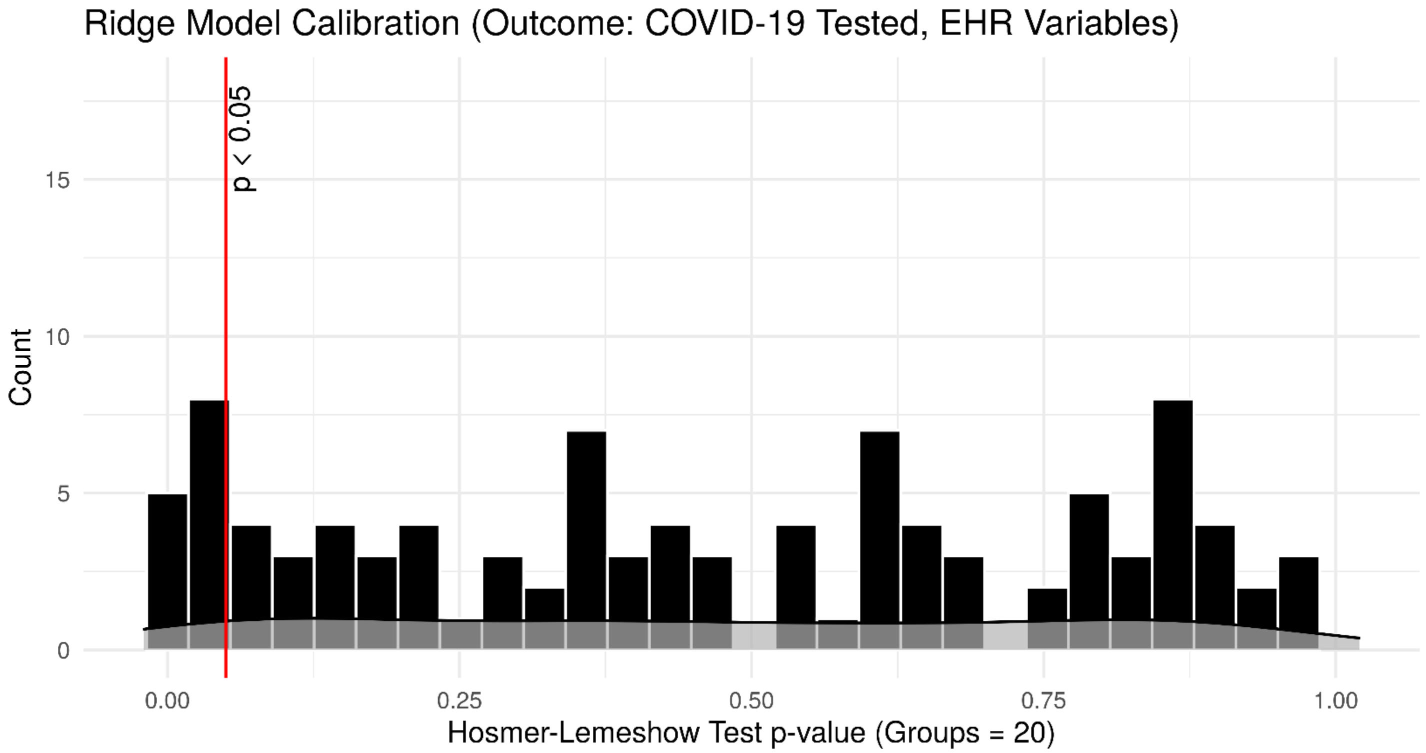

We plotted the p-values from conducting a Hosmer-Lemeshow goodness of fit test on all 100 train/test splits of the model evaluation procedure. Models tending to have poor calibration would show large numbers of p-values below the statistical significance threshold of 0.05.

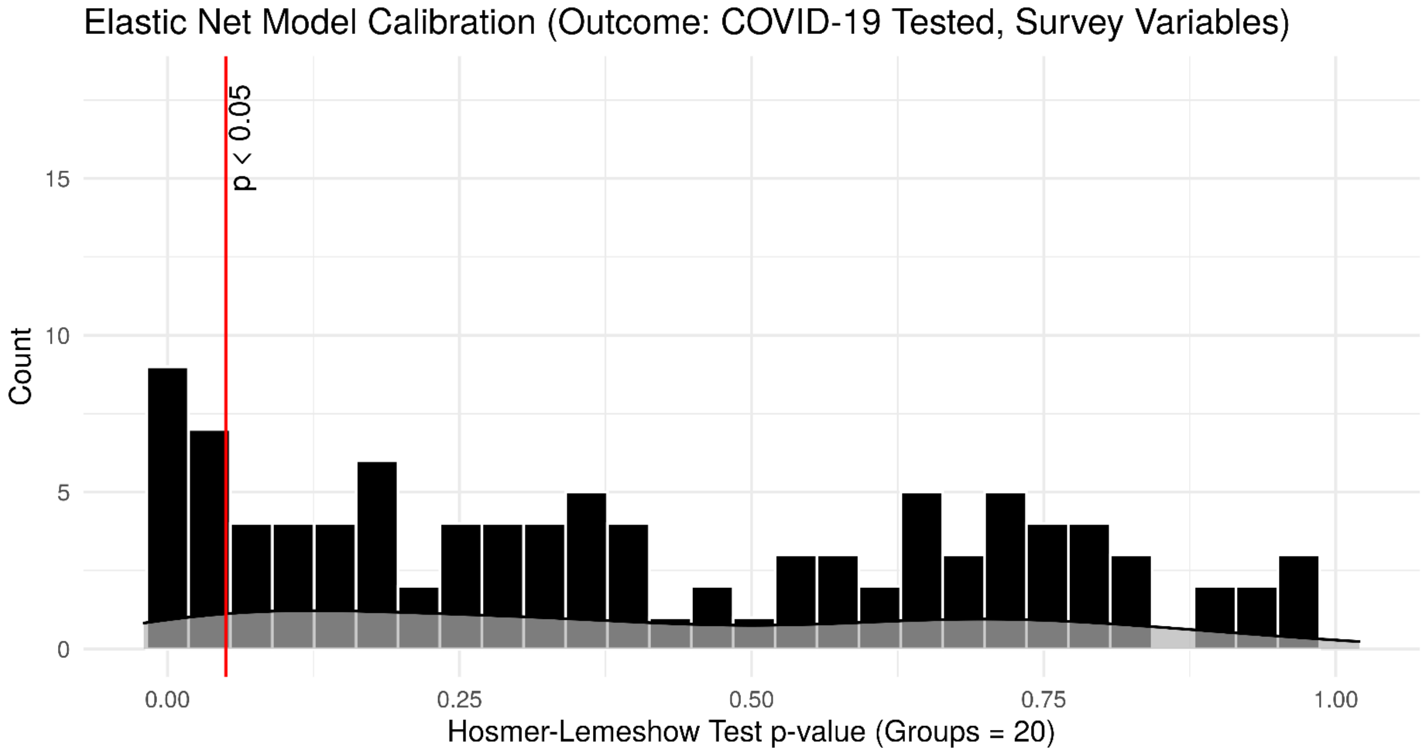

We plotted the p-values from conducting a Hosmer-Lemeshow goodness of fit test on all 100 train/test splits of the model evaluation procedure. Models tending to have poor calibration would show large numbers of p-values below the statistical significance threshold of 0.05.

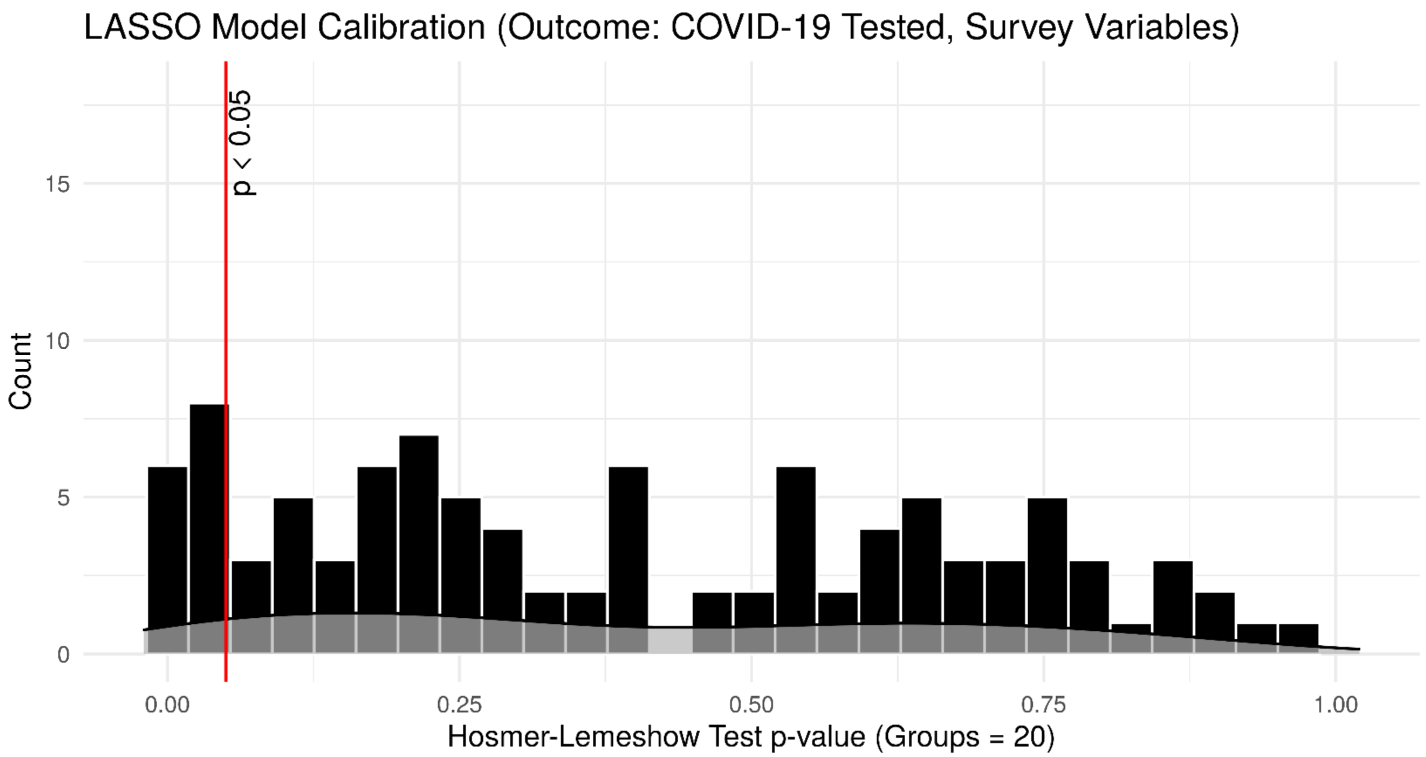

We plotted the p-values from conducting a Hosmer-Lemeshow goodness of fit test on all 100 train/test splits of the model evaluation procedure. Models tending to have poor calibration would show large numbers of p-values below the statistical significance threshold of 0.05.

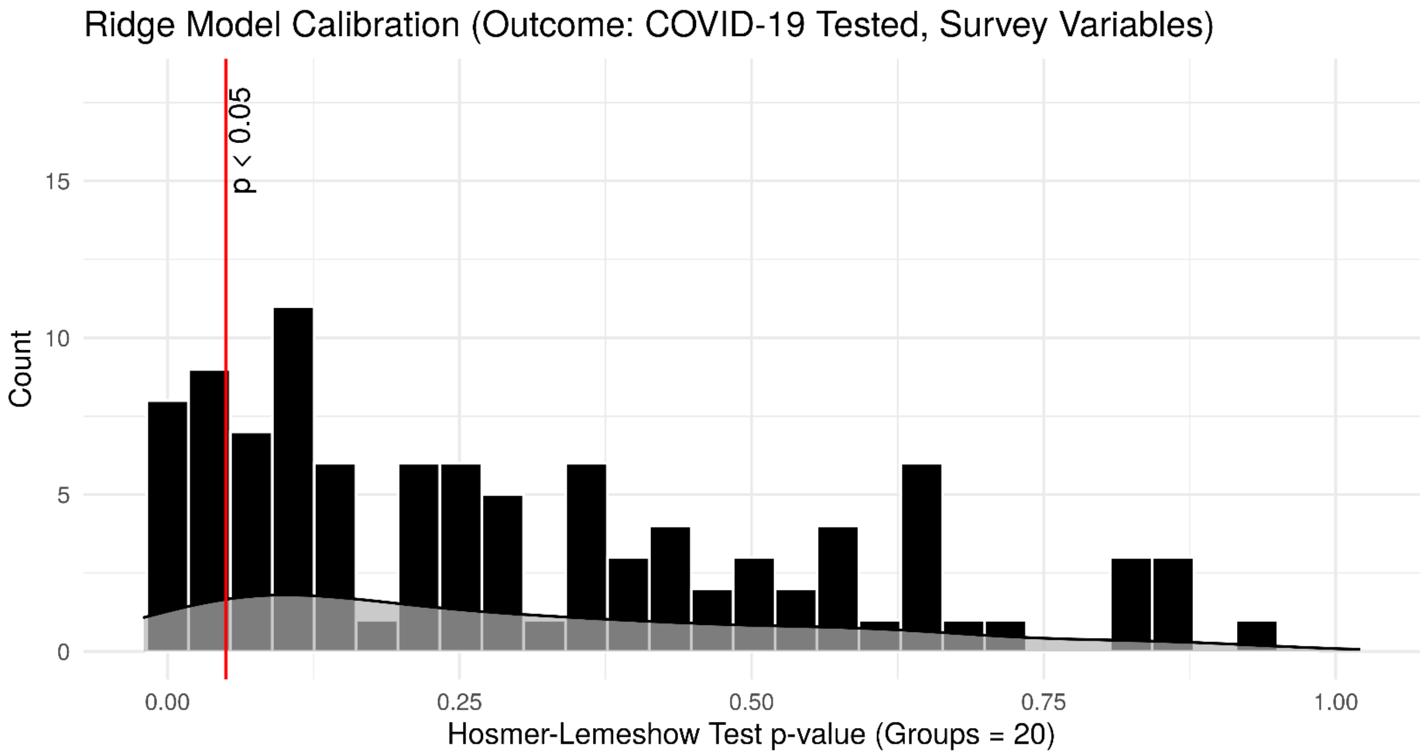

We plotted the p-values from conducting a Hosmer-Lemeshow goodness of fit test on all 100 train/test splits of the model evaluation procedure. Models tending to have poor calibration would show large numbers of p-values below the statistical significance threshold of 0.05.

S14 File. Description and Results of EHR-Based Case-Control Analysis

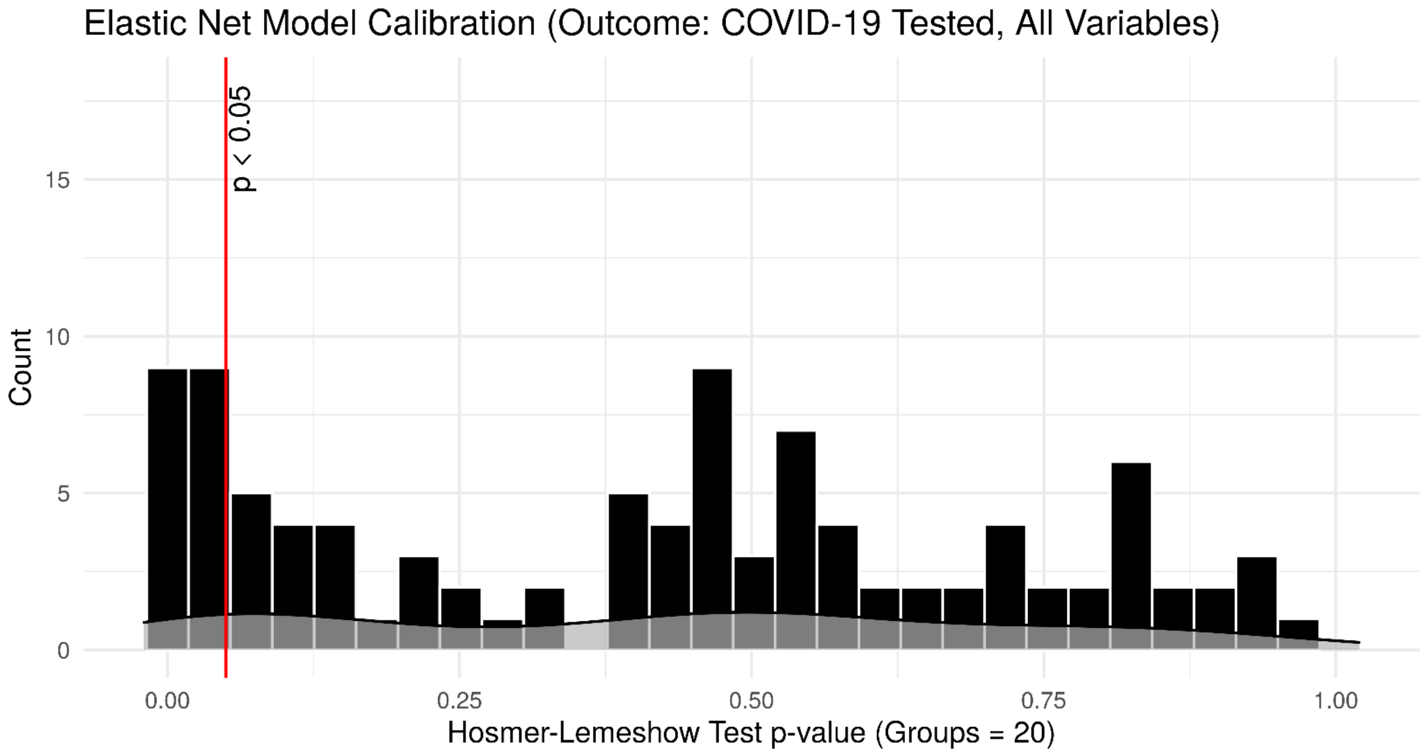

We plotted the p-values from conducting a Hosmer-Lemeshow goodness of fit test on all 100 train/test splits of the model evaluation procedure. Models tending to have poor calibration would show large numbers of p-values below the statistical significance threshold of 0.05.

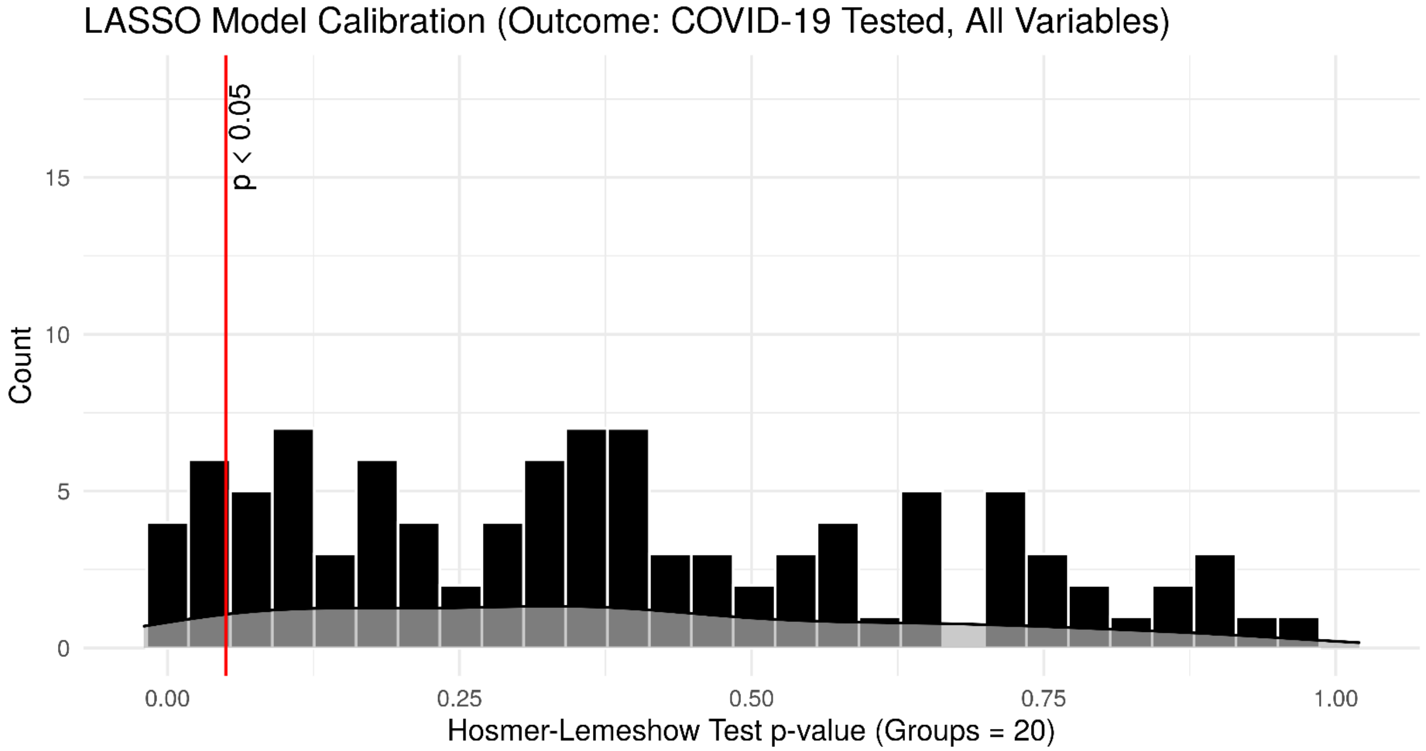

We plotted the p-values from conducting a Hosmer-Lemeshow goodness of fit test on all 100 train/test splits of the model evaluation procedure. Models tending to have poor calibration would show large numbers of p-values below the statistical significance threshold of 0.05.

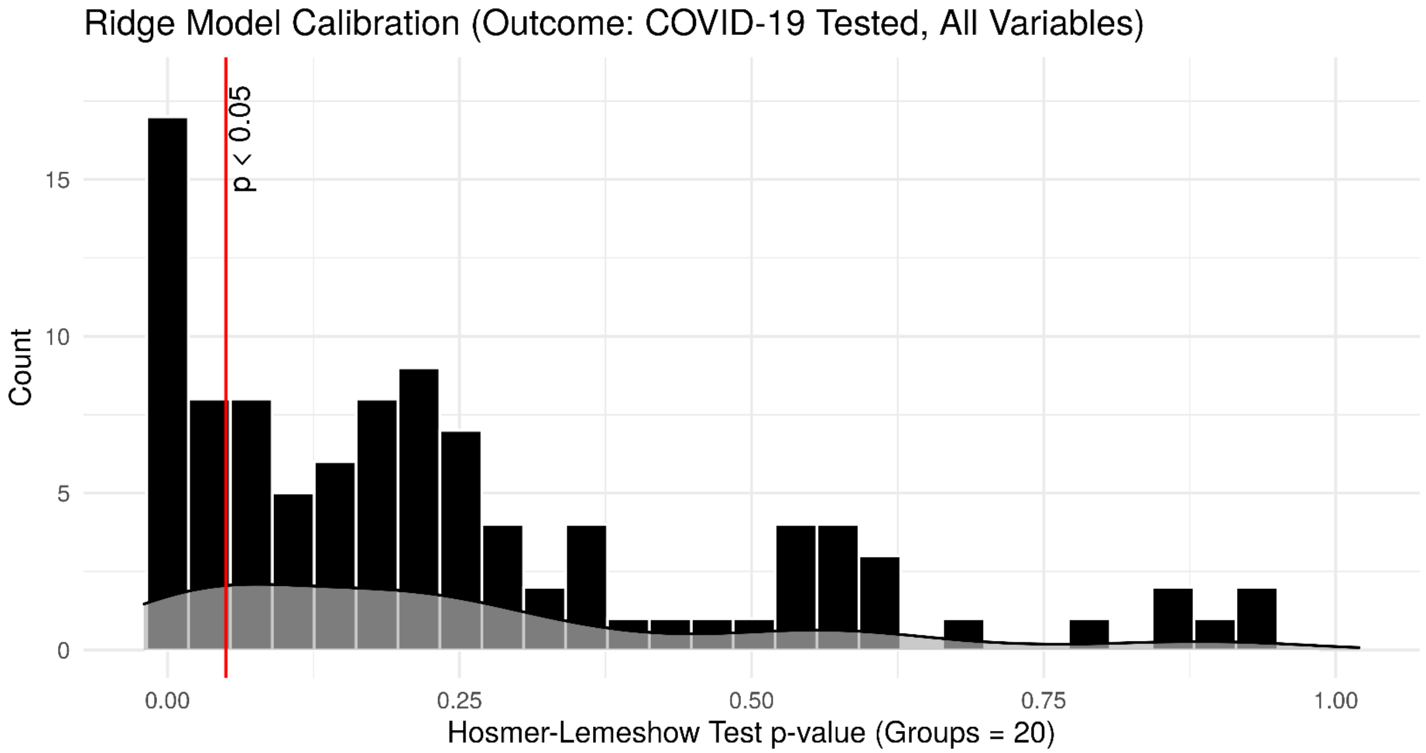

We plotted the p-values from conducting a Hosmer-Lemeshow goodness of fit test on all 100 train/test splits of the model evaluation procedure. Models tending to have poor calibration would show large numbers of p-values below the statistical significance threshold of 0.05.

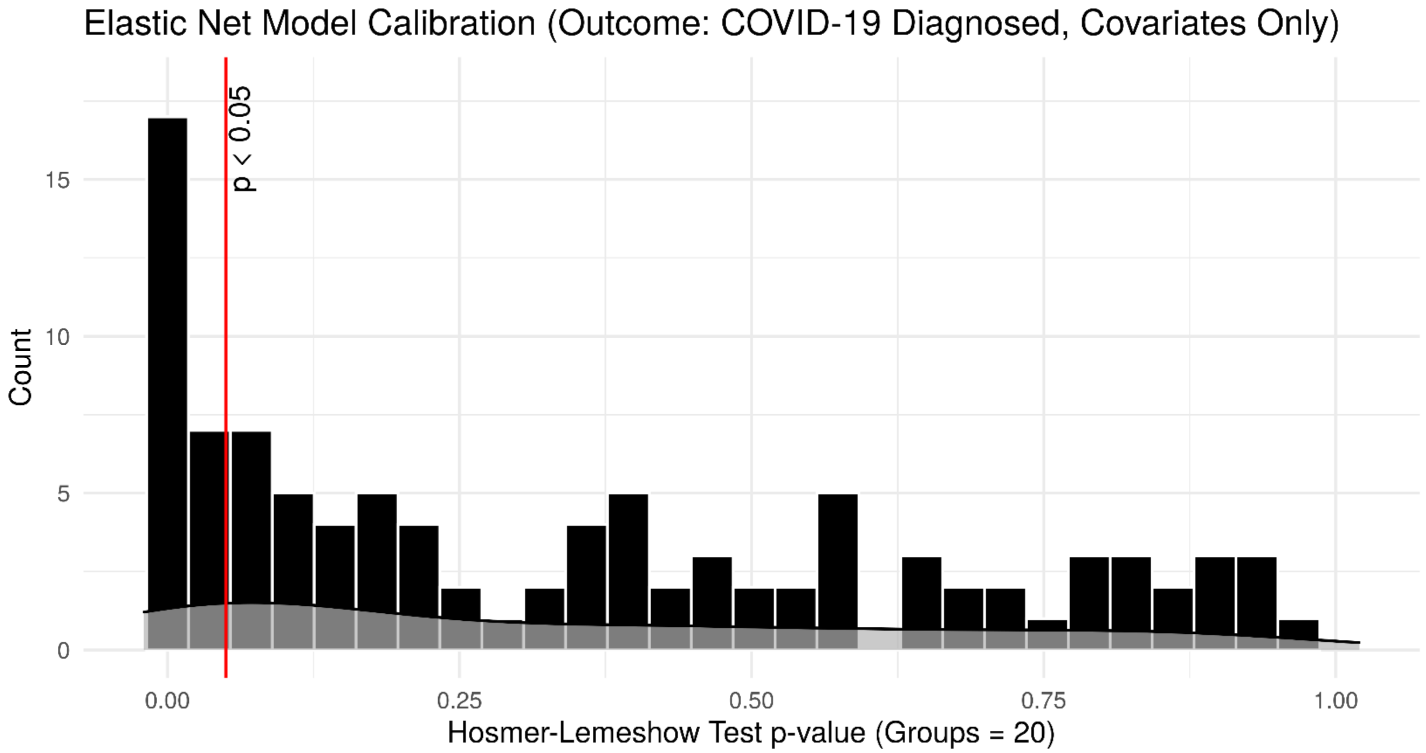

We plotted the p-values from conducting a Hosmer-Lemeshow goodness of fit test on all 100 train/test splits of the model evaluation procedure. Models tending to have poor calibration would show large numbers of p-values below the statistical significance threshold of 0.05.

S14 File. Description and Results of EHR-Based Case-Control Analysis

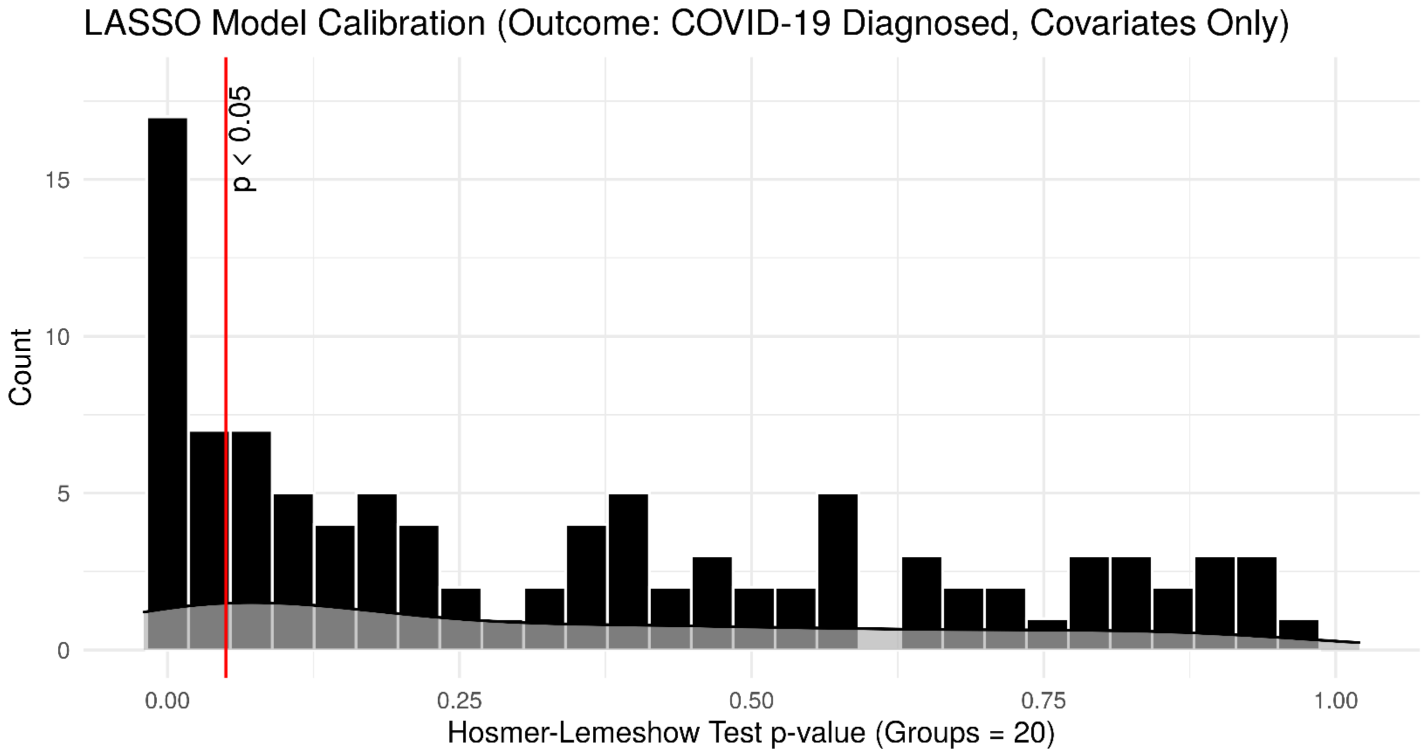

We plotted the p-values from conducting a Hosmer-Lemeshow goodness of fit test on all 100 train/test splits of the model evaluation procedure. Models tending to have poor calibration would show large numbers of p-values below the statistical significance threshold of 0.05.

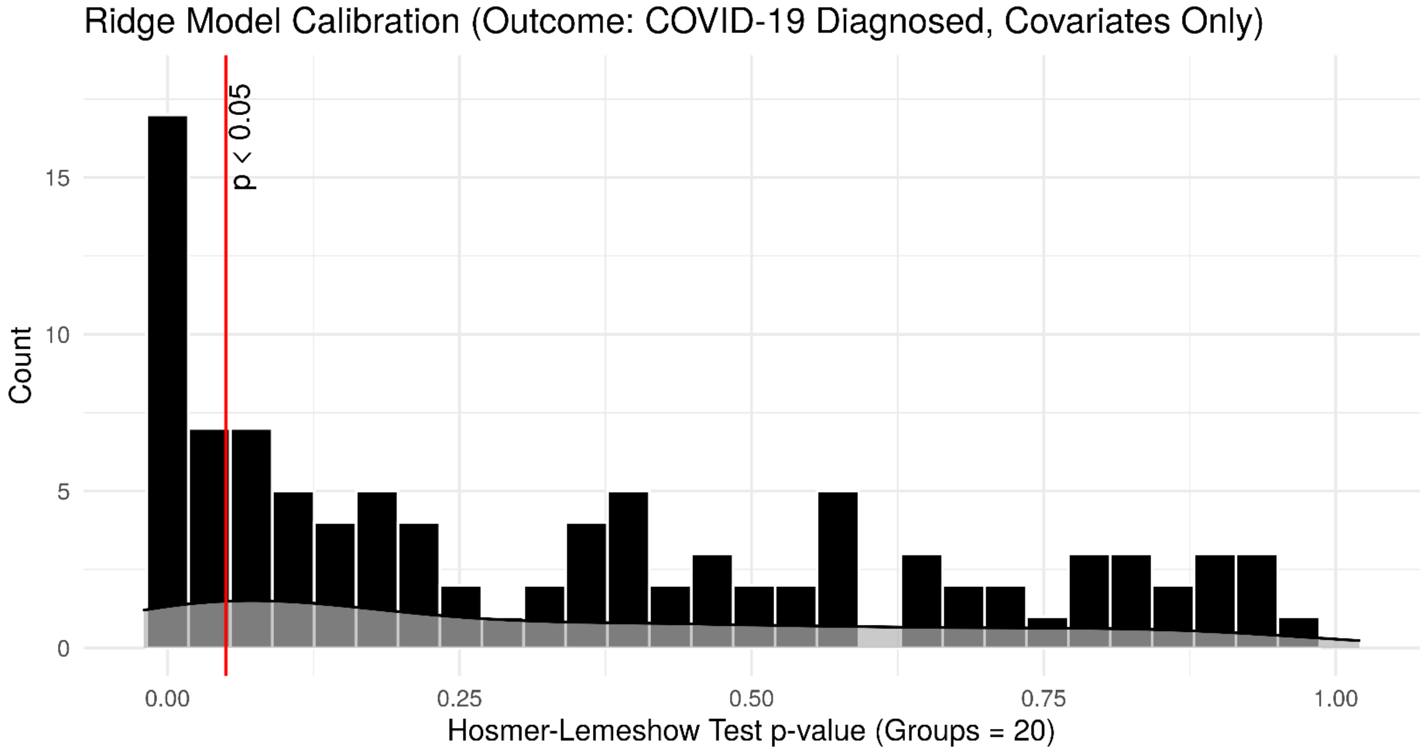

We plotted the p-values from conducting a Hosmer-Lemeshow goodness of fit test on all 100 train/test splits of the model evaluation procedure. Models tending to have poor calibration would show large numbers of p-values below the statistical significance threshold of 0.05.

S14 File. Description and Results of EHR-Based Case-Control Analysis

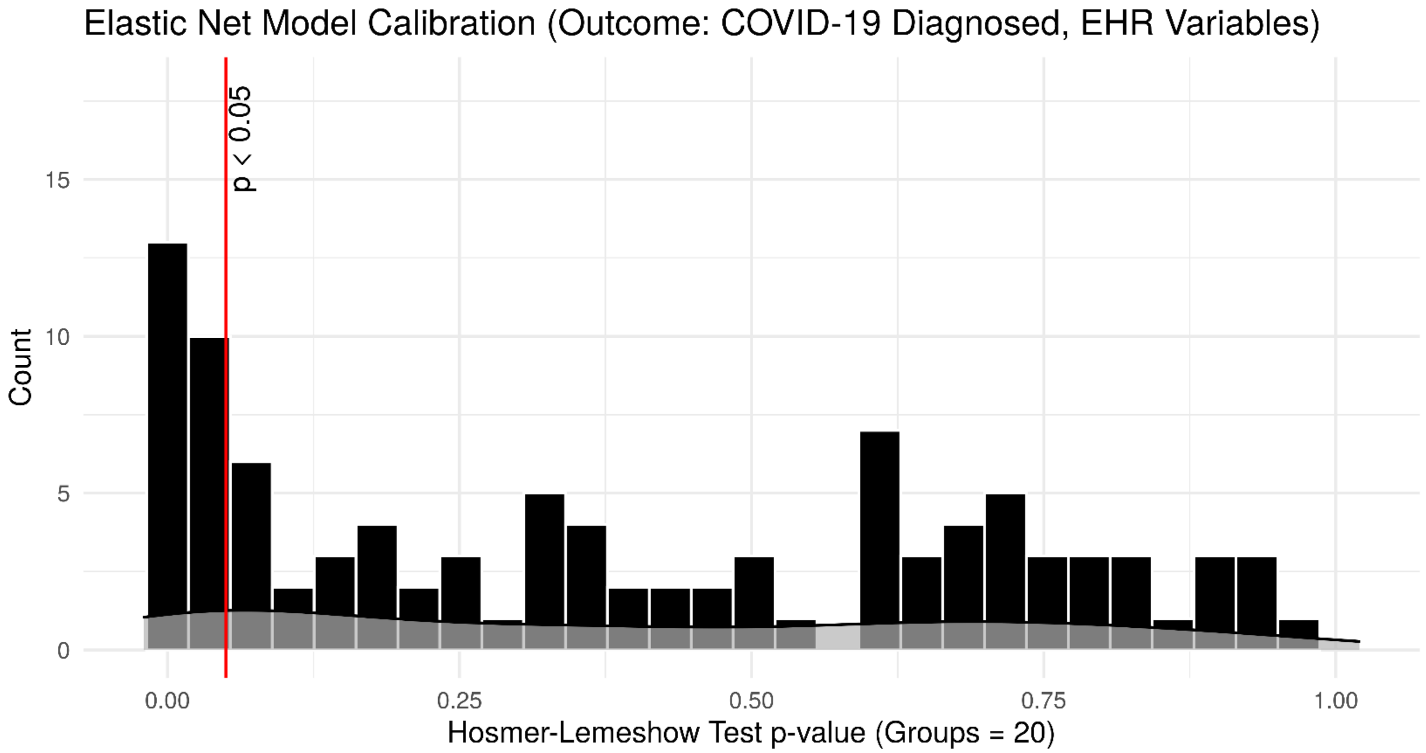

We plotted the p-values from conducting a Hosmer-Lemeshow goodness of fit test on all 100 train/test splits of the model evaluation procedure. Models tending to have poor calibration would show large numbers of p-values below the statistical significance threshold of 0.05.

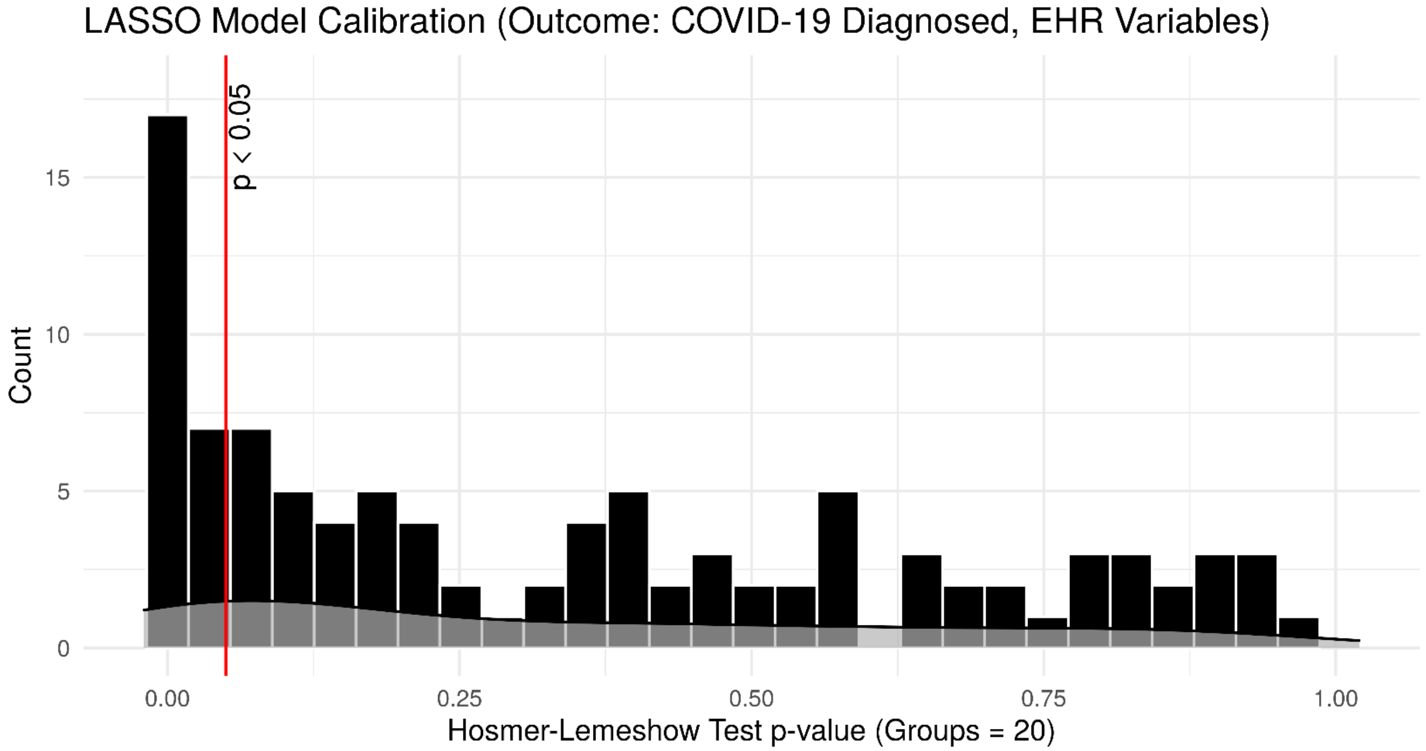

We plotted the p-values from conducting a Hosmer-Lemeshow goodness of fit test on all 100 train/test splits of the model evaluation procedure. Models tending to have poor calibration would show large numbers of p-values below the statistical significance threshold of 0.05.

S14 File. Description and Results of EHR-Based Case-Control Analysis

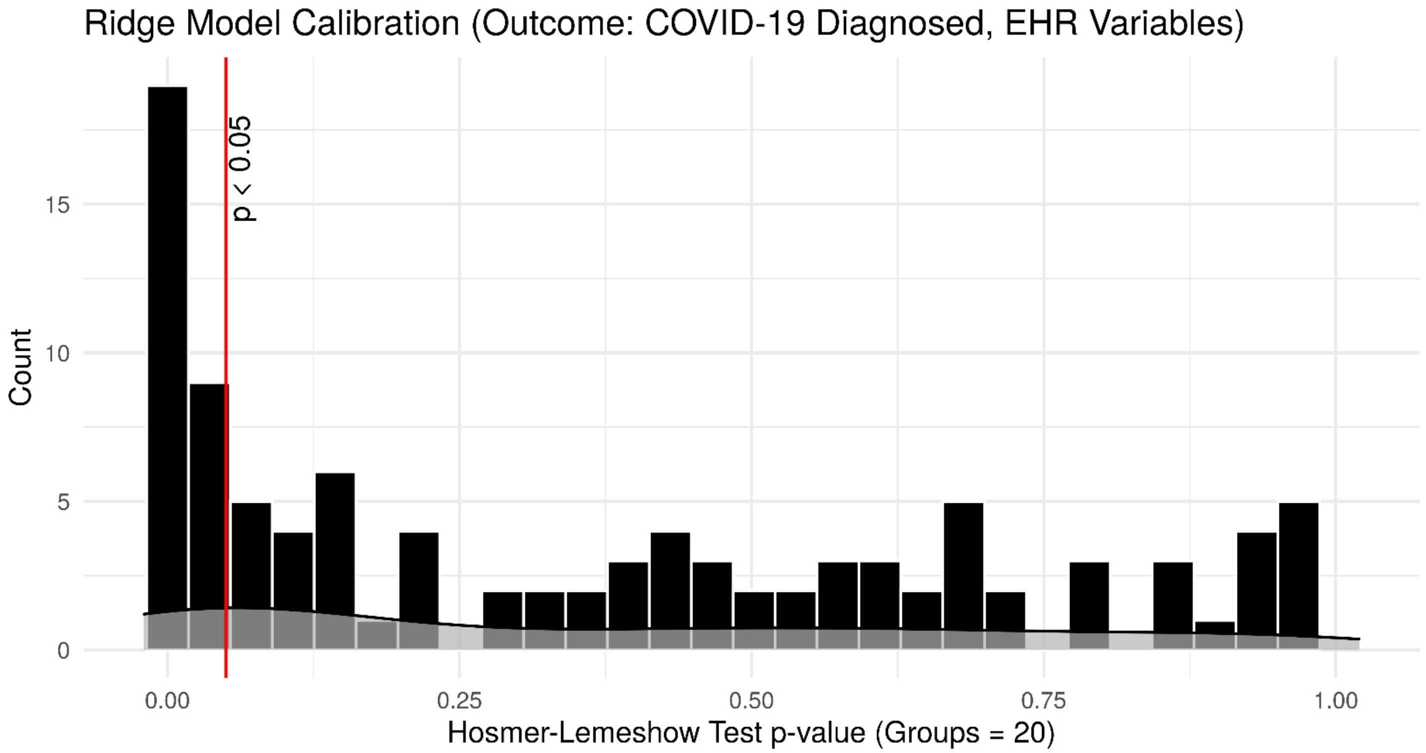

We plotted the p-values from conducting a Hosmer-Lemeshow goodness of fit test on all 100 train/test splits of the model evaluation procedure. Models tending to have poor calibration would show large numbers of p-values below the statistical significance threshold of 0.05.

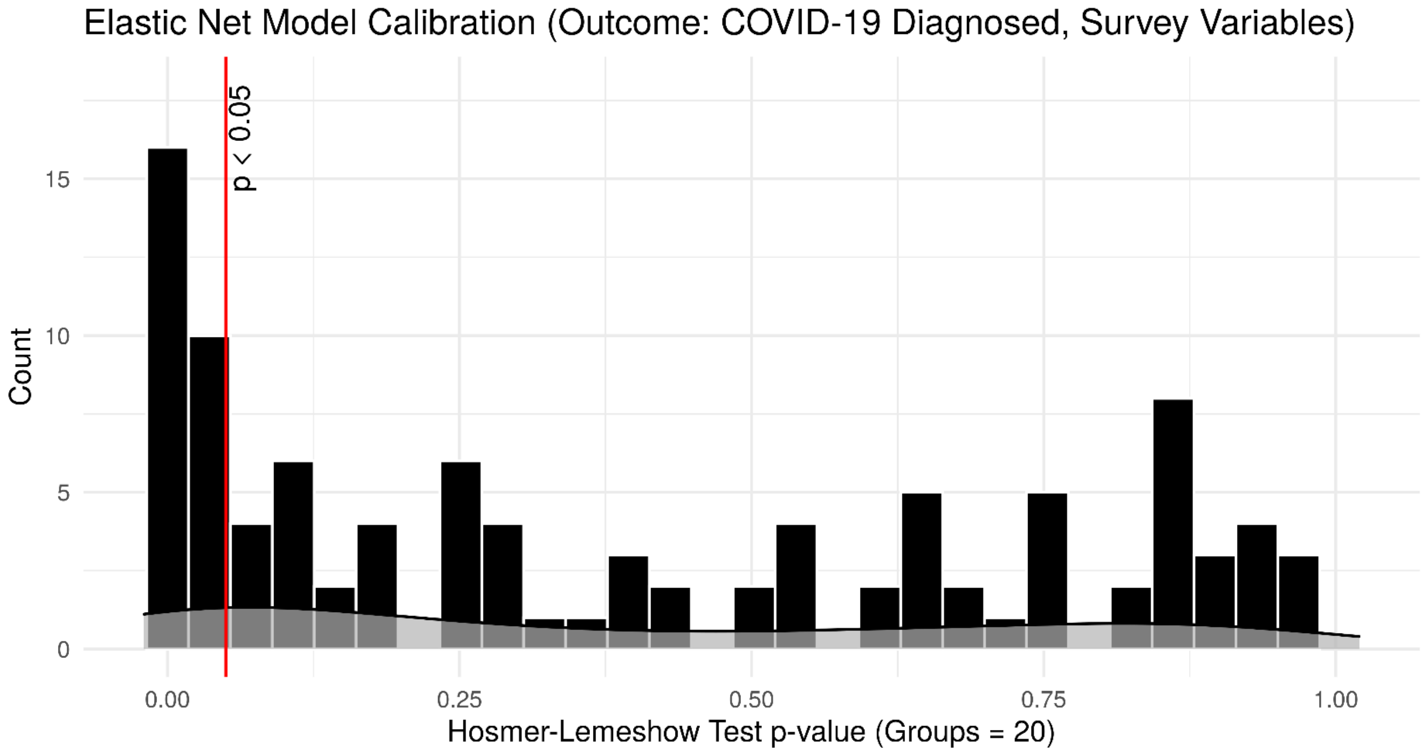

We plotted the p-values from conducting a Hosmer-Lemeshow goodness of fit test on all 100 train/test splits of the model evaluation procedure. Models tending to have poor calibration would show large numbers of p-values below the statistical significance threshold of 0.05.

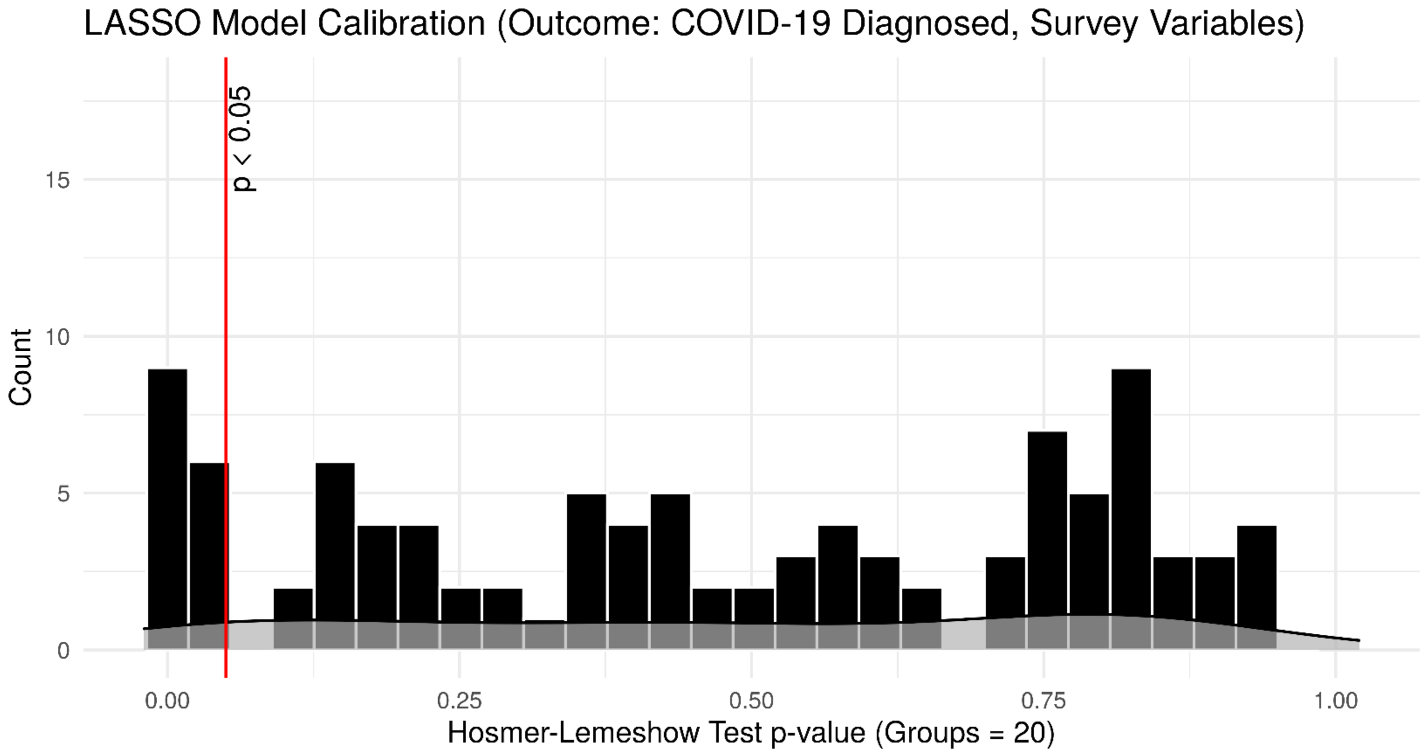

We plotted the p-values from conducting a Hosmer-Lemeshow goodness of fit test on all 100 train/test splits of the model evaluation procedure. Models tending to have poor calibration would show large numbers of p-values below the statistical significance threshold of 0.05.

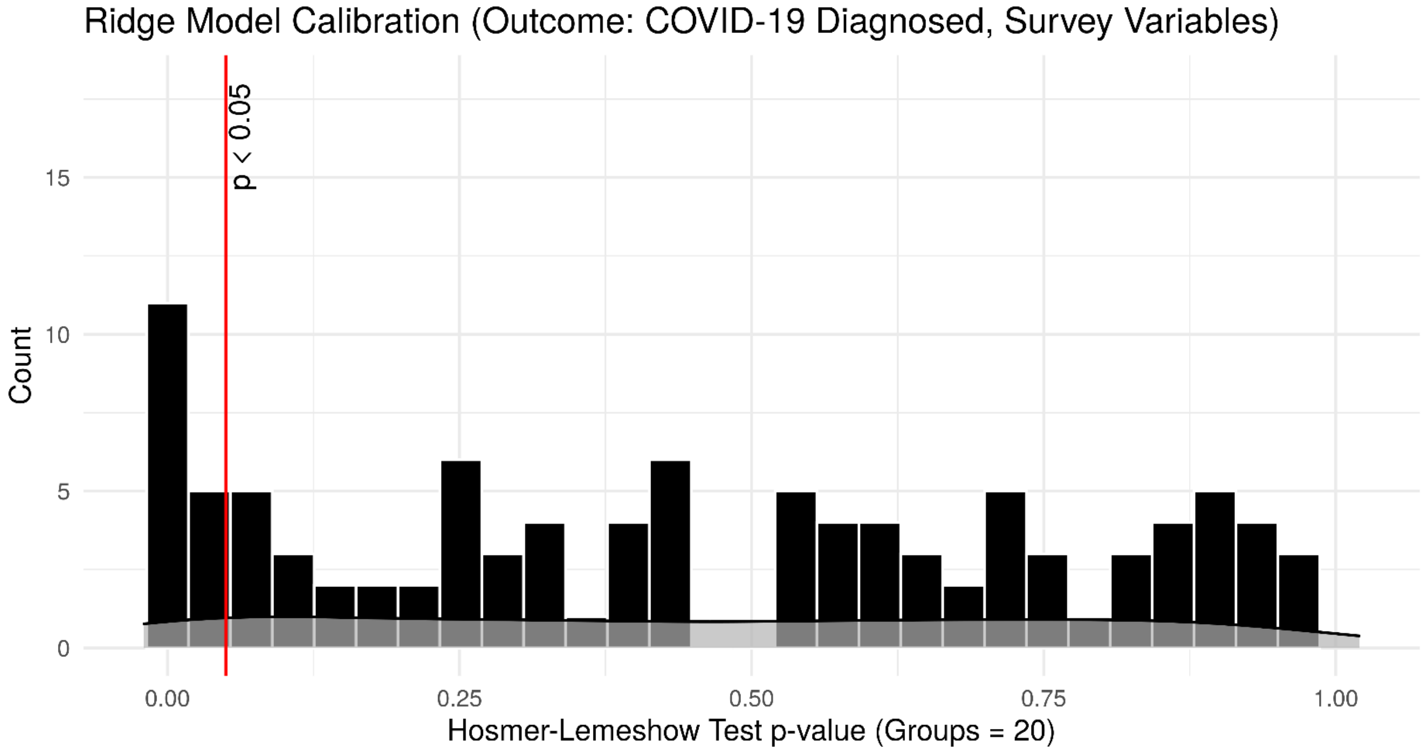

We plotted the p-values from conducting a Hosmer-Lemeshow goodness of fit test on all 100 train/test splits of the model evaluation procedure. Models tending to have poor calibration would show large numbers of p-values below the statistical significance threshold of 0.05.

S14 File. Description and Results of EHR-Based Case-Control Analysis

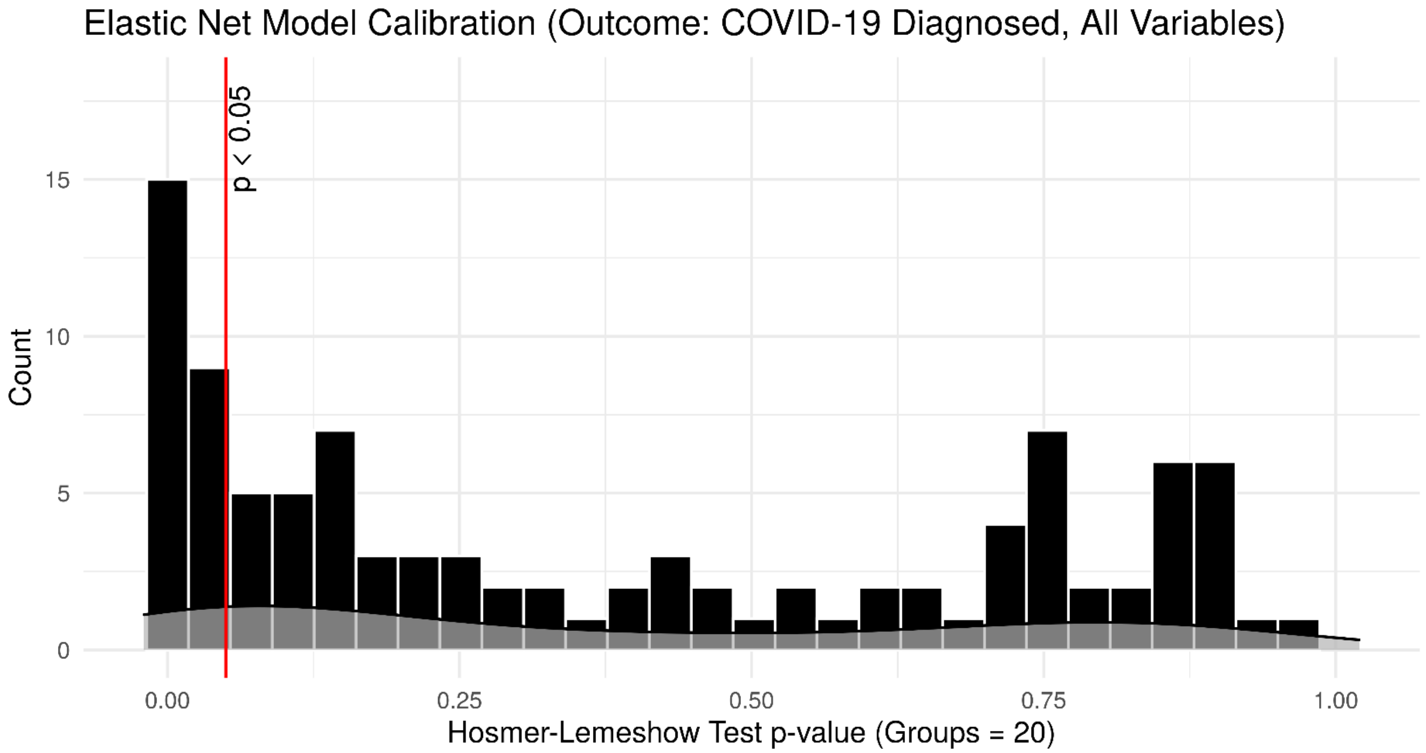

We plotted the p-values from conducting a Hosmer-Lemeshow goodness of fit test on all 100 train/test splits of the model evaluation procedure. Models tending to have poor calibration would show large numbers of p-values below the statistical significance threshold of 0.05.

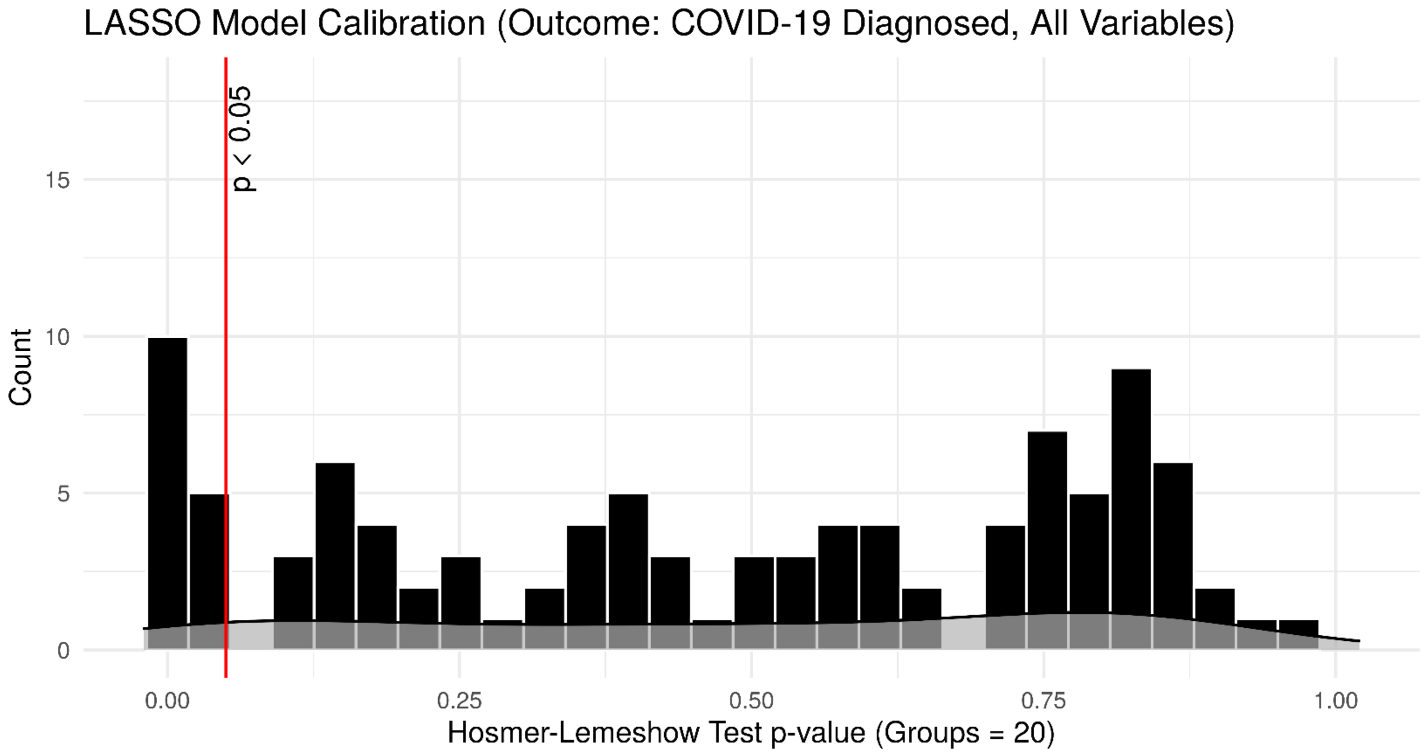

We plotted the p-values from conducting a Hosmer-Lemeshow goodness of fit test on all 100 train/test splits of the model evaluation procedure. Models tending to have poor calibration would show large numbers of p-values below the statistical significance threshold of 0.05.

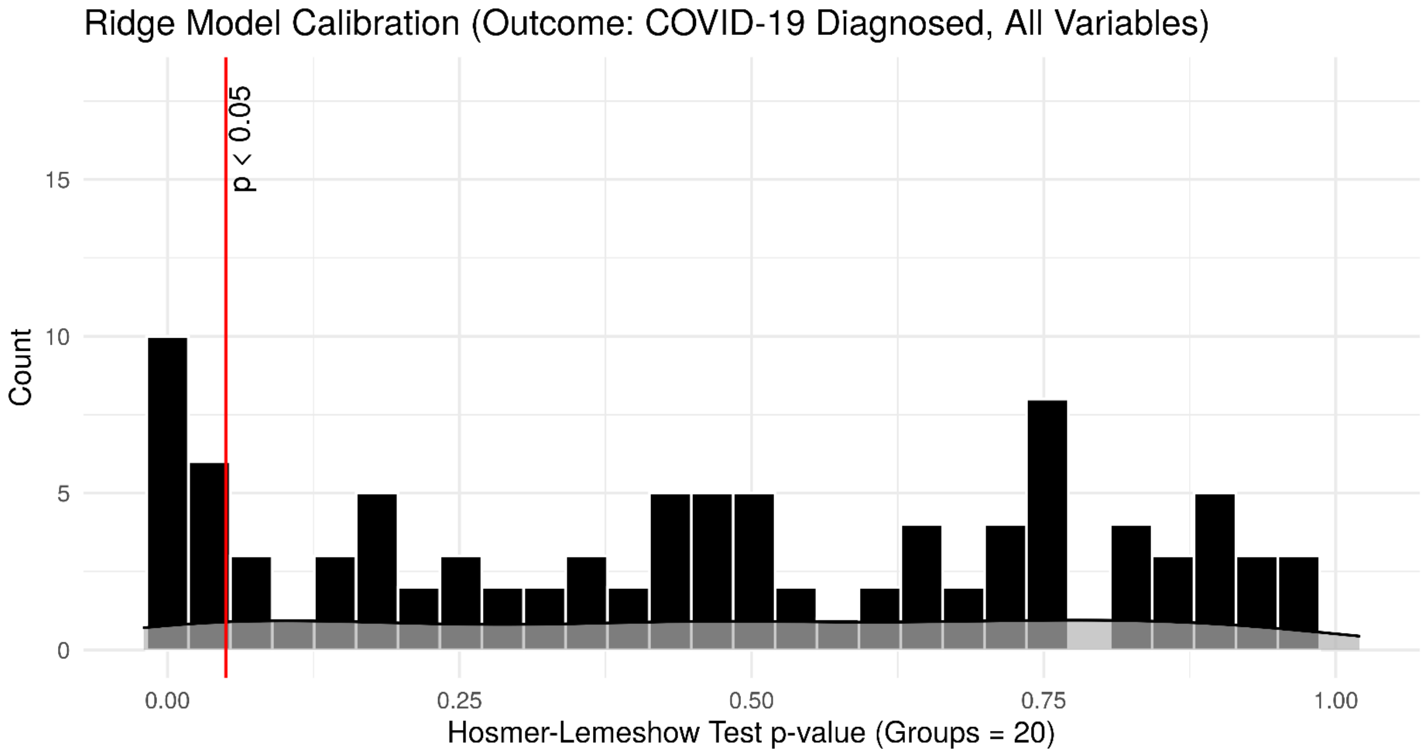

We plotted the p-values from conducting a Hosmer-Lemeshow goodness of fit test on all 100 train/test splits of the model evaluation procedure. Models tending to have poor calibration would show large numbers of p-values below the statistical significance threshold of 0.05.

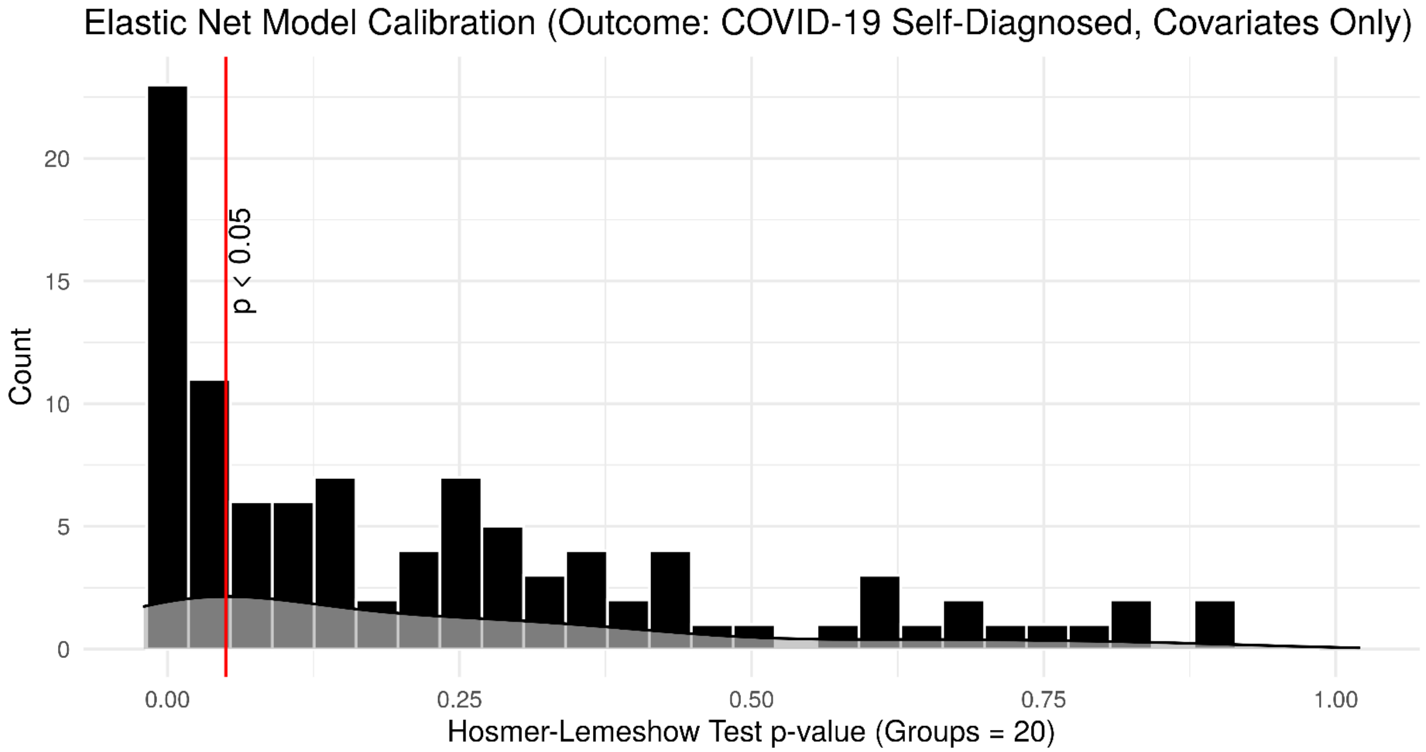

We plotted the p-values from conducting a Hosmer-Lemeshow goodness of fit test on all 100 train/test splits of the model evaluation procedure. Models tending to have poor calibration would show large numbers of p-values below the statistical significance threshold of 0.05.

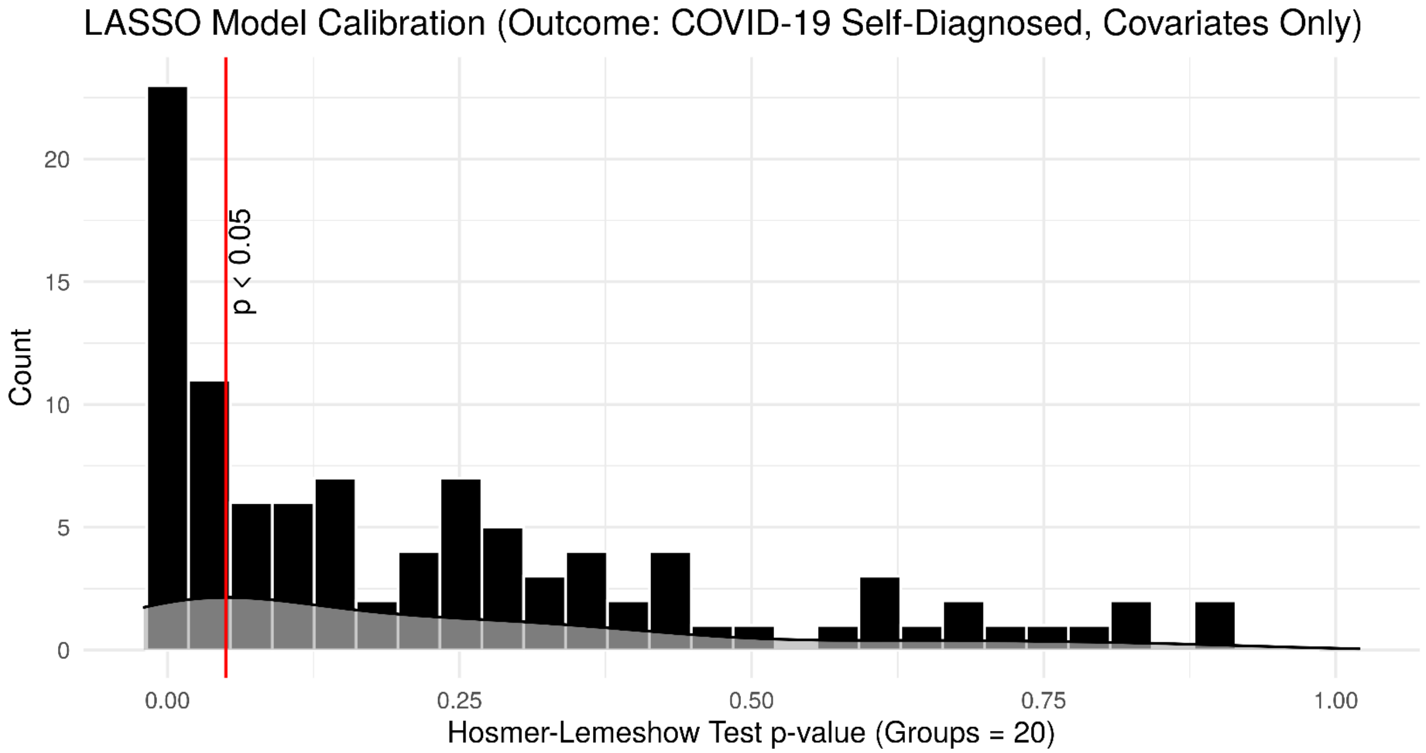

We plotted the p-values from conducting a Hosmer-Lemeshow goodness of fit test on all 100 train/test splits of the model evaluation procedure. Models tending to have poor calibration would show large numbers of p-values below the statistical significance threshold of 0.05.

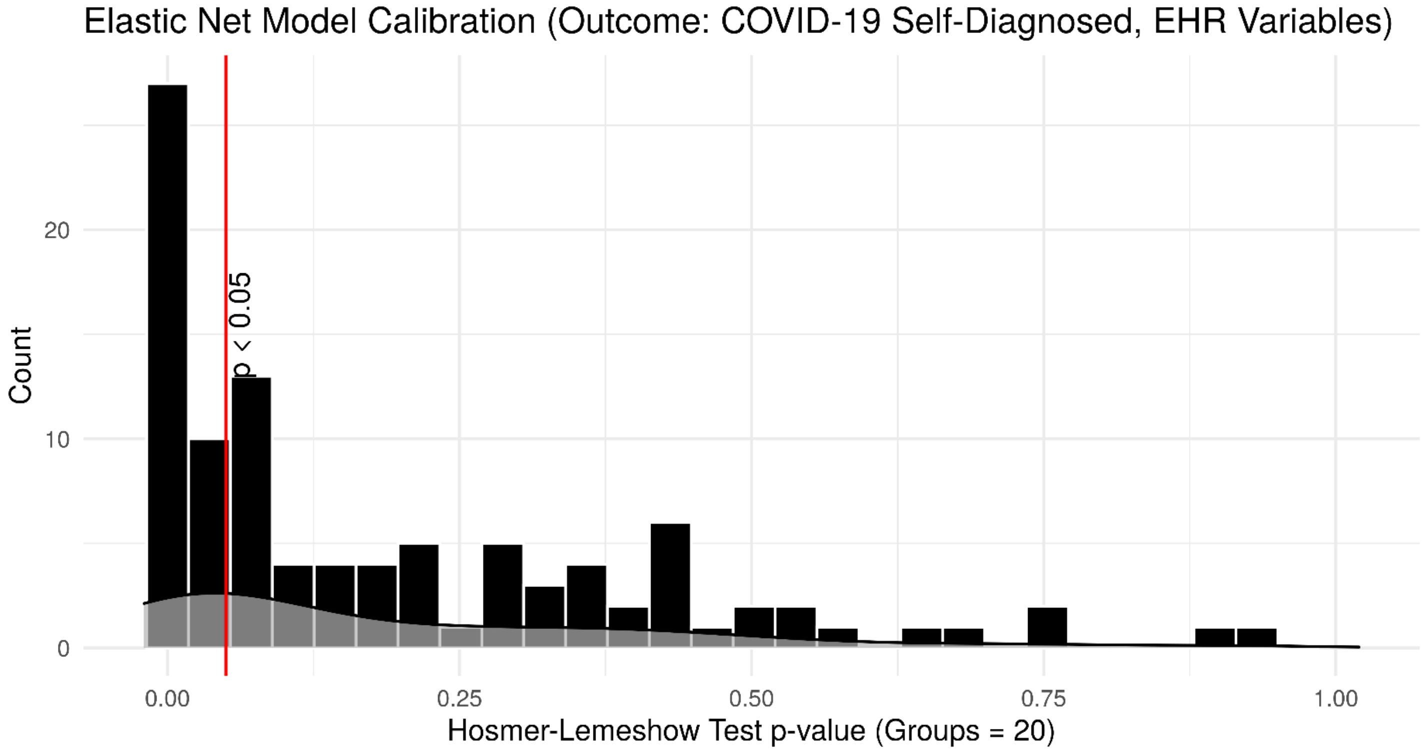

We plotted the p-values from conducting a Hosmer-Lemeshow goodness of fit test on all 100 train/test splits of the model evaluation procedure. Models tending to have poor calibration would show large numbers of p-values below the statistical significance threshold of 0.05.

S14 File. Description and Results of EHR-Based Case-Control Analysis

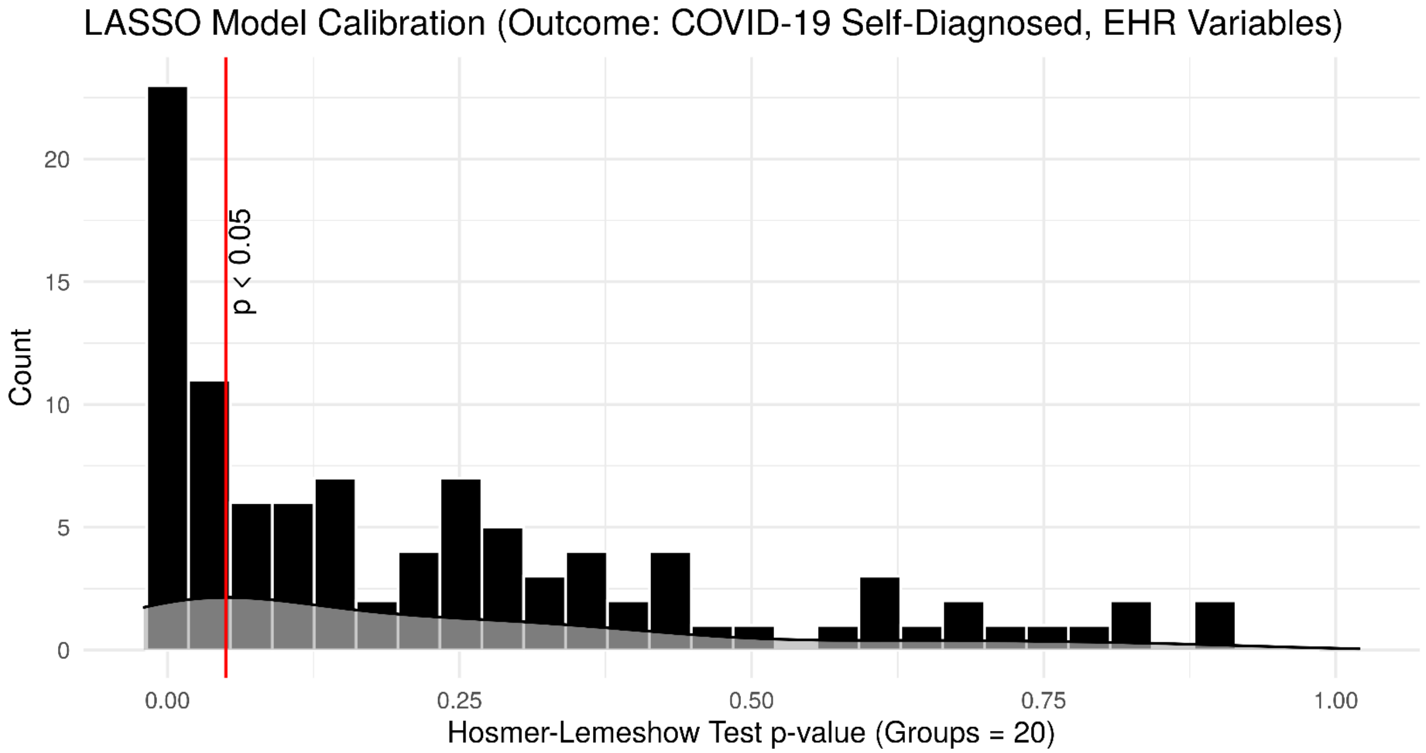

We plotted the p-values from conducting a Hosmer-Lemeshow goodness of fit test on all 100 train/test splits of the model evaluation procedure. Models tending to have poor calibration would show large numbers of p-values below the statistical significance threshold of 0.05.

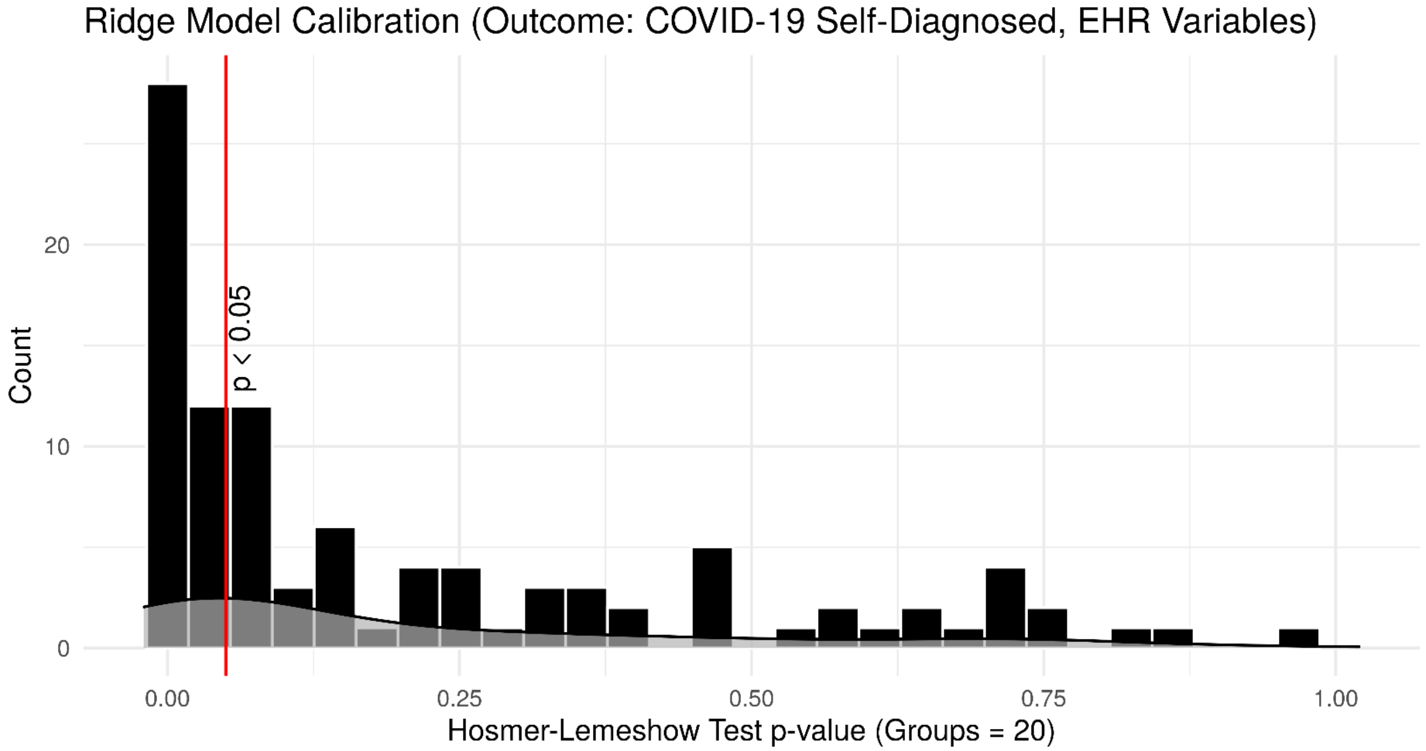

We plotted the p-values from conducting a Hosmer-Lemeshow goodness of fit test on all 100 train/test splits of the model evaluation procedure. Models tending to have poor calibration would show large numbers of p-values below the statistical significance threshold of 0.05.

S14 File. Description and Results of EHR-Based Case-Control Analysis

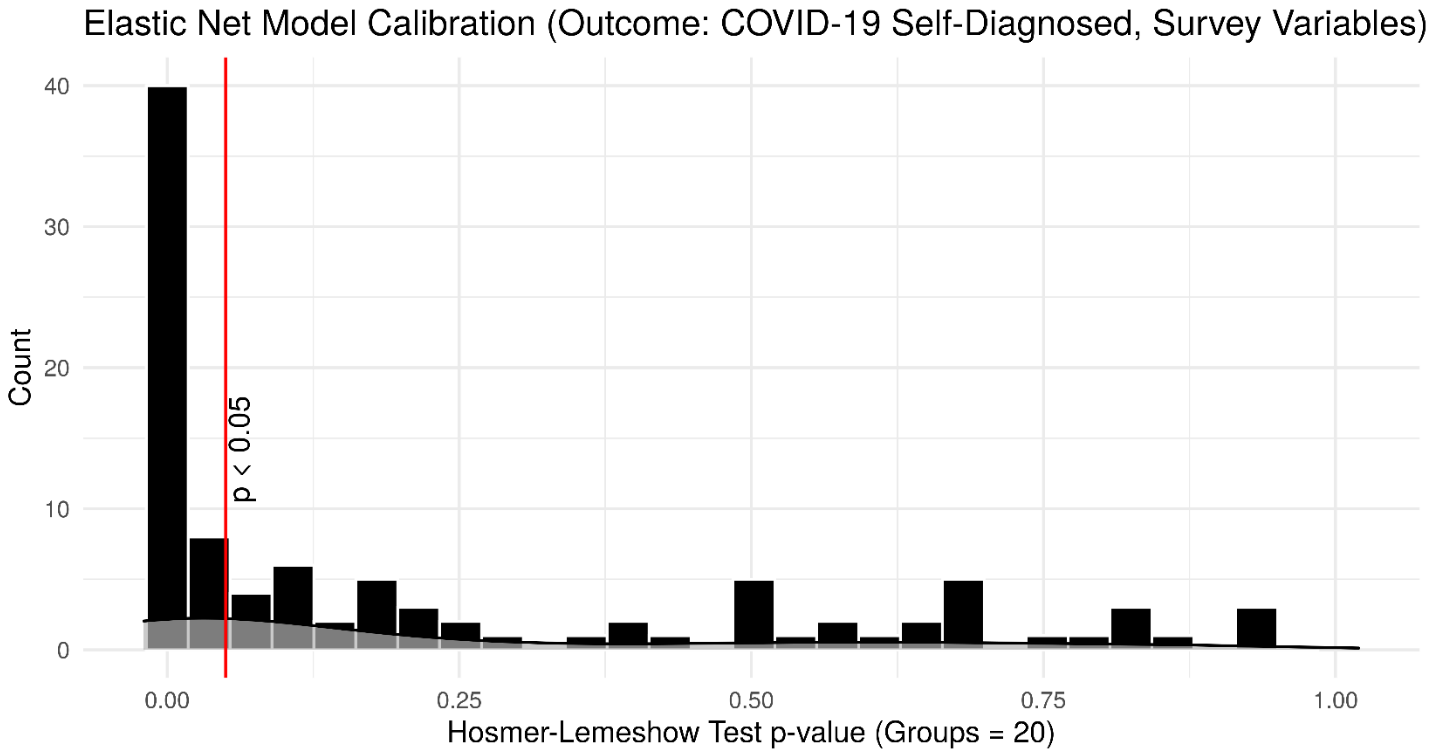

We plotted the p-values from conducting a Hosmer-Lemeshow goodness of fit test on all 100 train/test splits of the model evaluation procedure. Models tending to have poor calibration would show large numbers of p-values below the statistical significance threshold of 0.05.

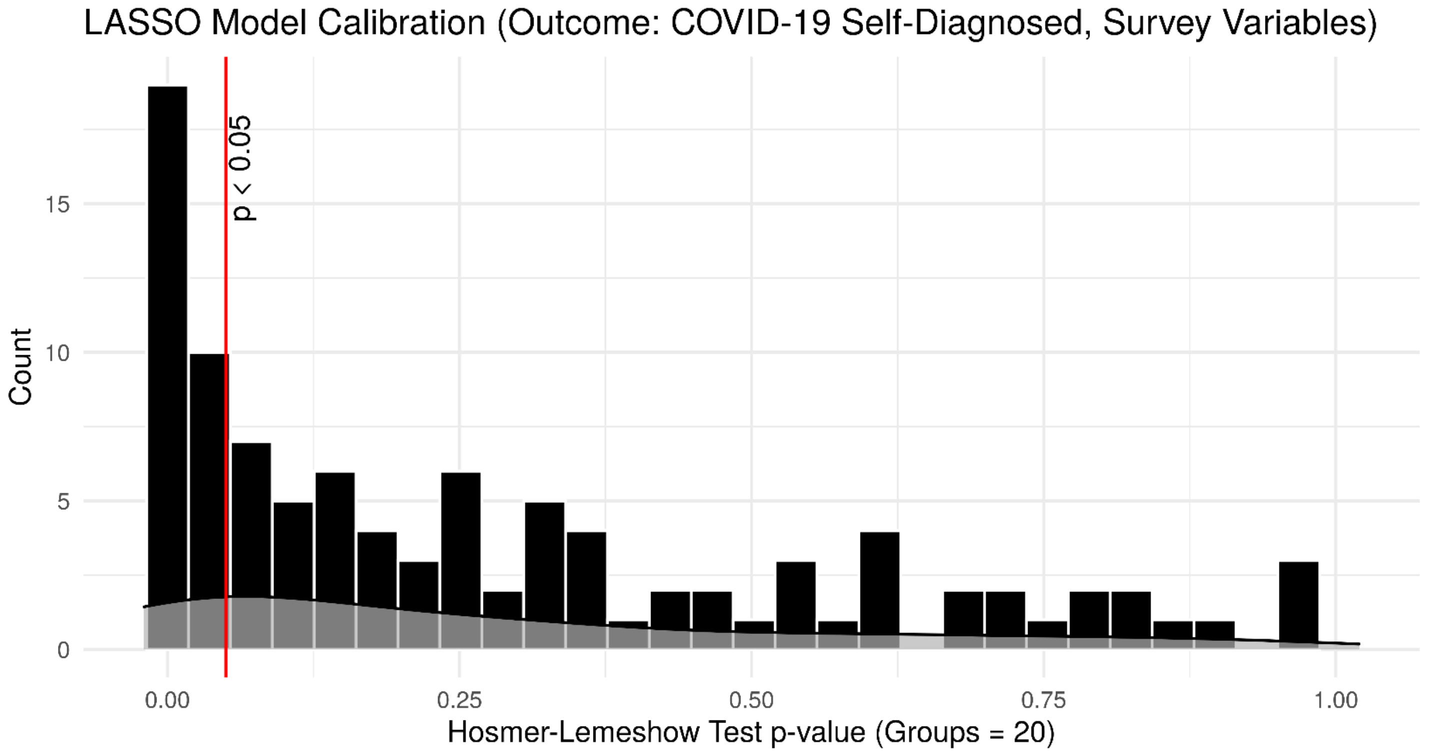

We plotted the p-values from conducting a Hosmer-Lemeshow goodness of fit test on all 100 train/test splits of the model evaluation procedure. Models tending to have poor calibration would show large numbers of p-values below the statistical significance threshold of 0.05.

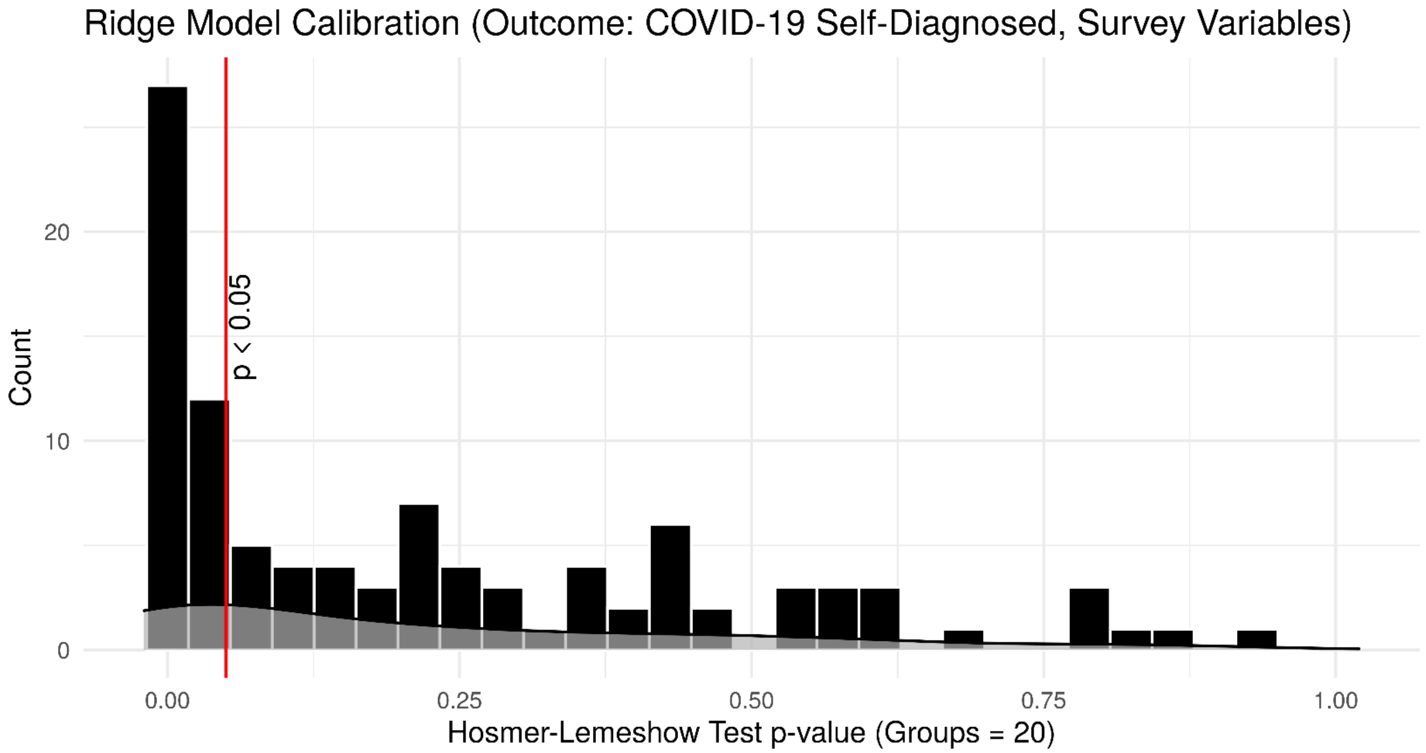

We plotted the p-values from conducting a Hosmer-Lemeshow goodness of fit test on all 100 train/test splits of the model evaluation procedure. Models tending to have poor calibration would show large numbers of p-values below the statistical significance threshold of 0.05.

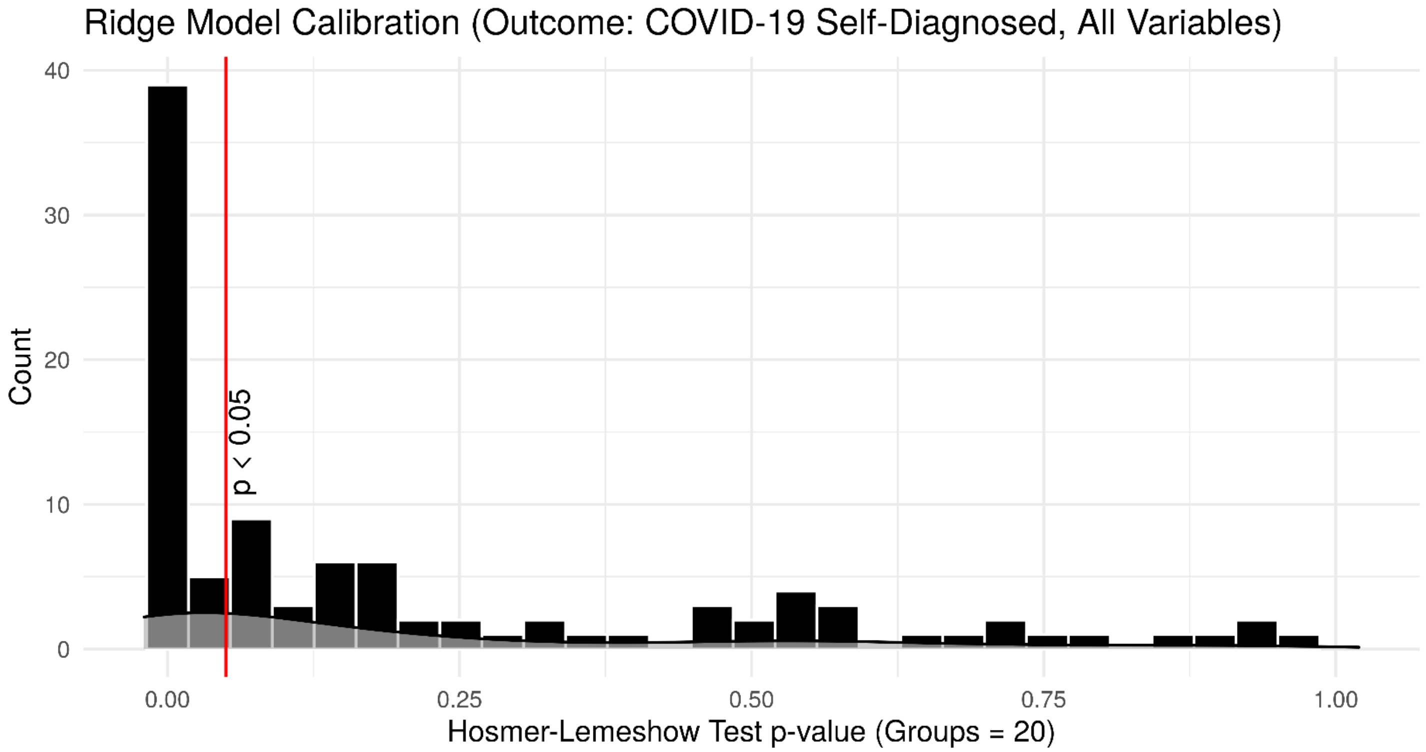

We plotted the p-values from conducting a Hosmer-Lemeshow goodness of fit test on all 100 train/test splits of the model evaluation procedure. Models tending to have poor calibration would show large numbers of p-values below the statistical significance threshold of 0.05.

S14 File. Description and Results of EHR-Based Case-Control Analysis

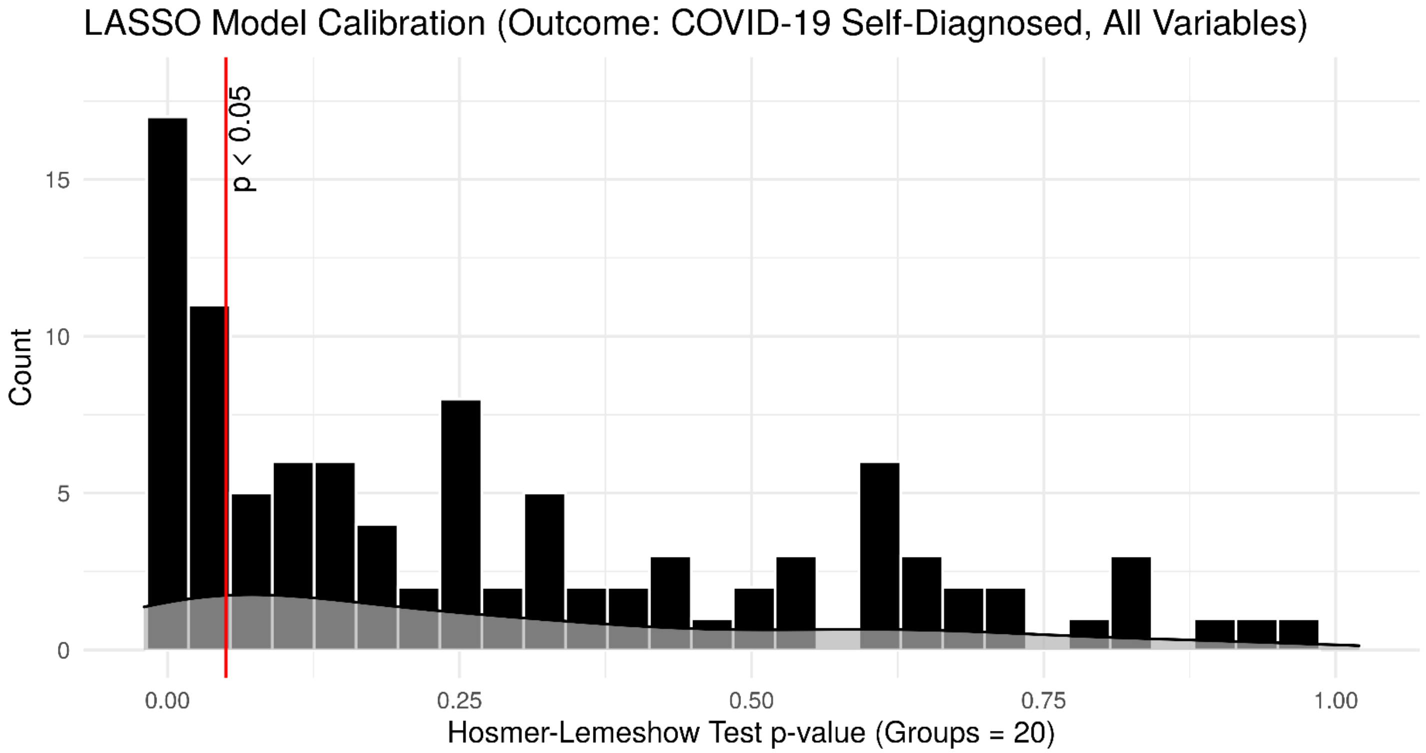

We plotted the p-values from conducting a Hosmer-Lemeshow goodness of fit test on all 100 train/test splits of the model evaluation procedure. Models tending to have poor calibration would show large numbers of p-values below the statistical significance threshold of 0.05.

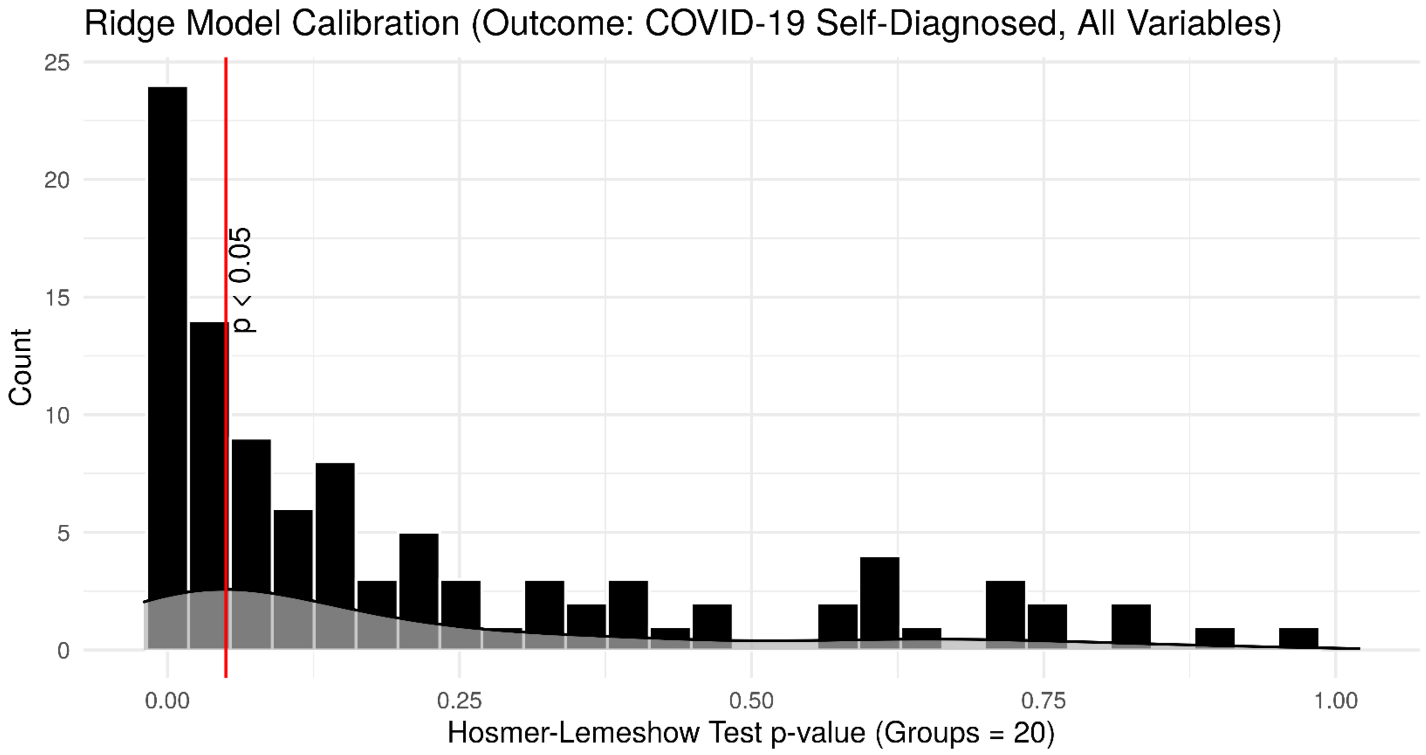

We plotted the p-values from conducting a Hosmer-Lemeshow goodness of fit test on all 100 train/test splits of the model evaluation procedure. Models tending to have poor calibration would show large numbers of p-values below the statistical significance threshold of 0.05.

S15 File. Calibration Plots for Survey-Based Models of COVID-19 Outcomes

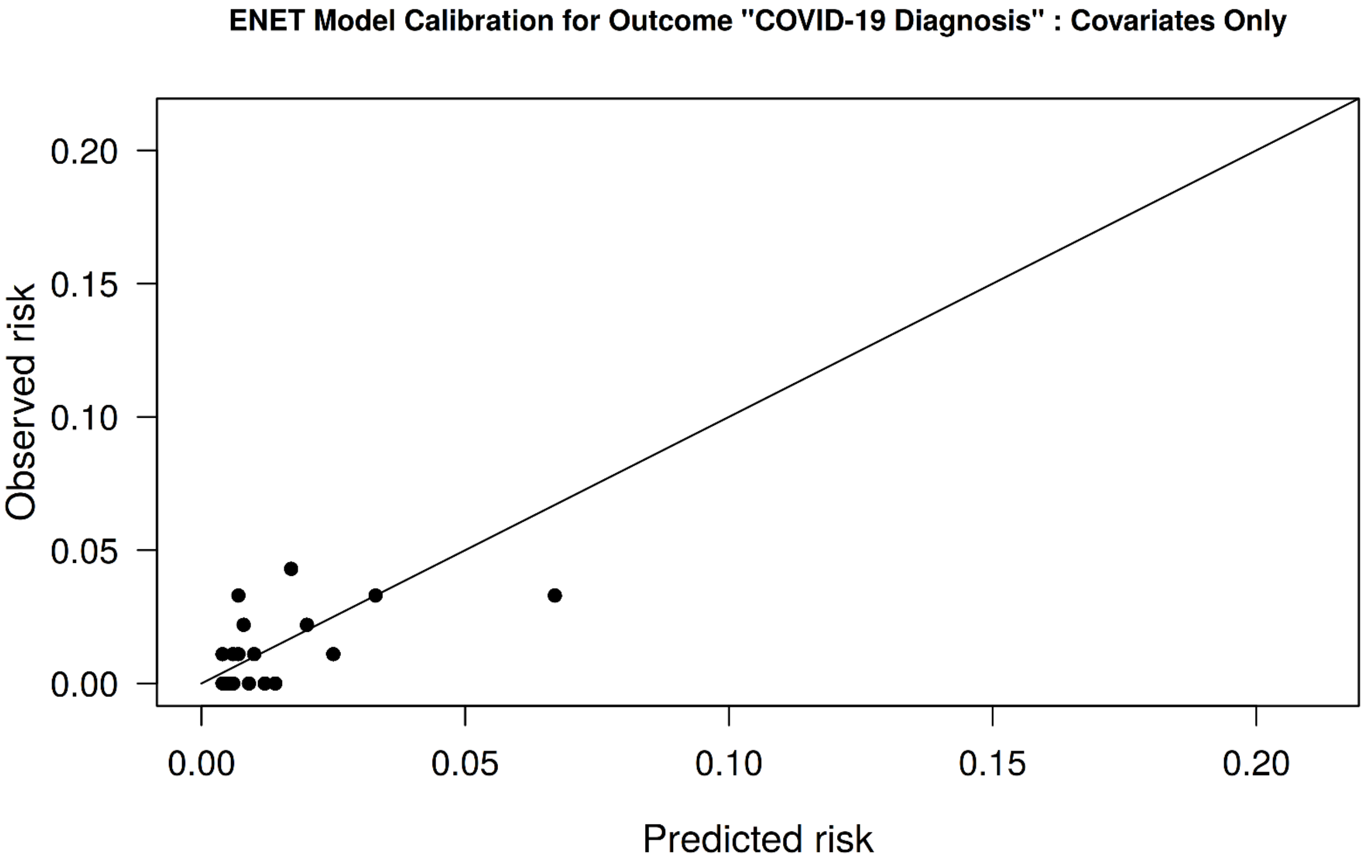

Subjects were split into 20 groups based on their predicted risk, and the observed proportion of COVID-19 tested subject in those groups was plotted against the expected proportion as per the model. The model used for this plot was from the first random 70/30 train-test split out of 100 total.

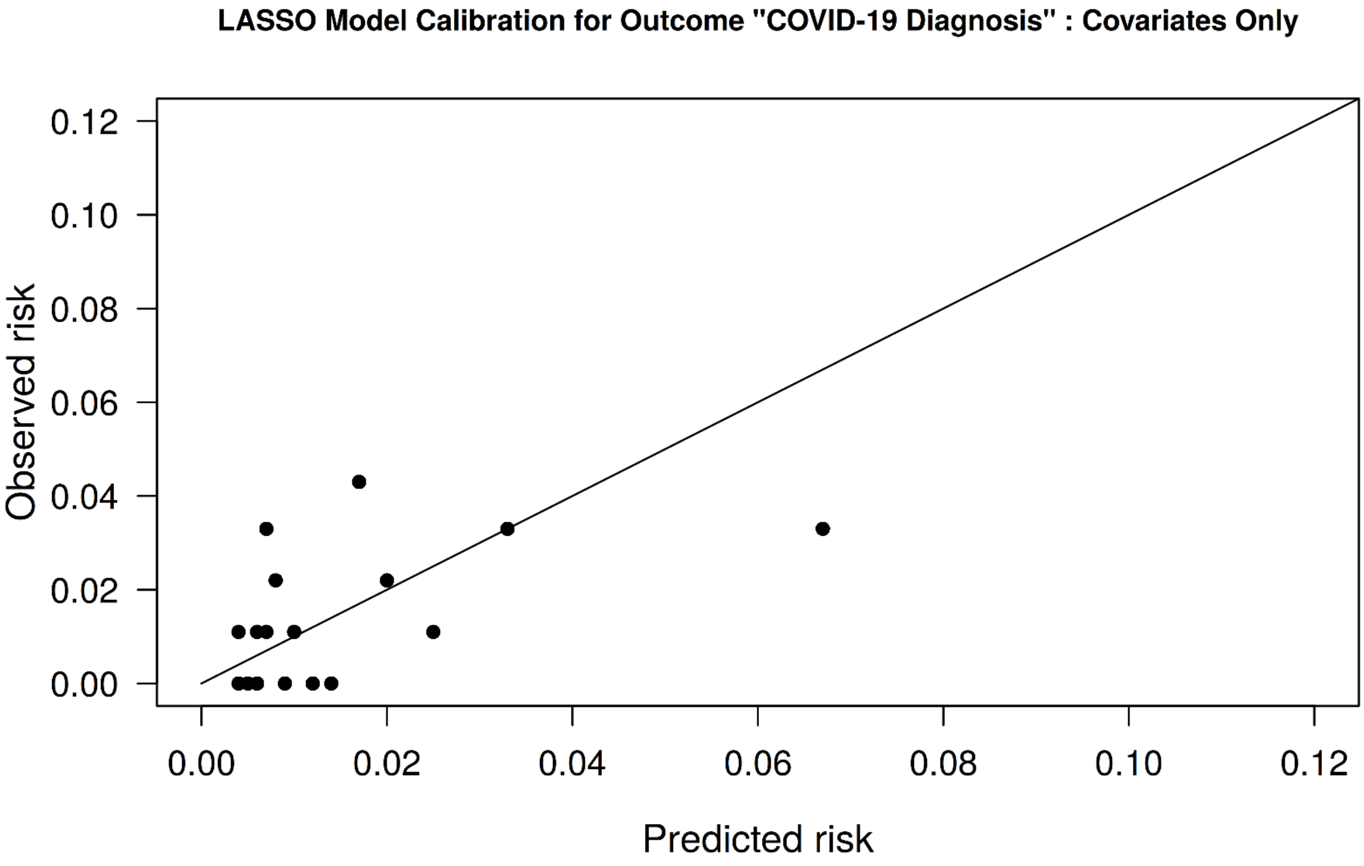

Subjects were split into 20 groups based on their predicted risk, and the observed proportion of COVID-19 tested subject in those groups was plotted against the expected proportion as per the model. The model used for this plot was from the first random 70/30 train-test split out of 100 total.

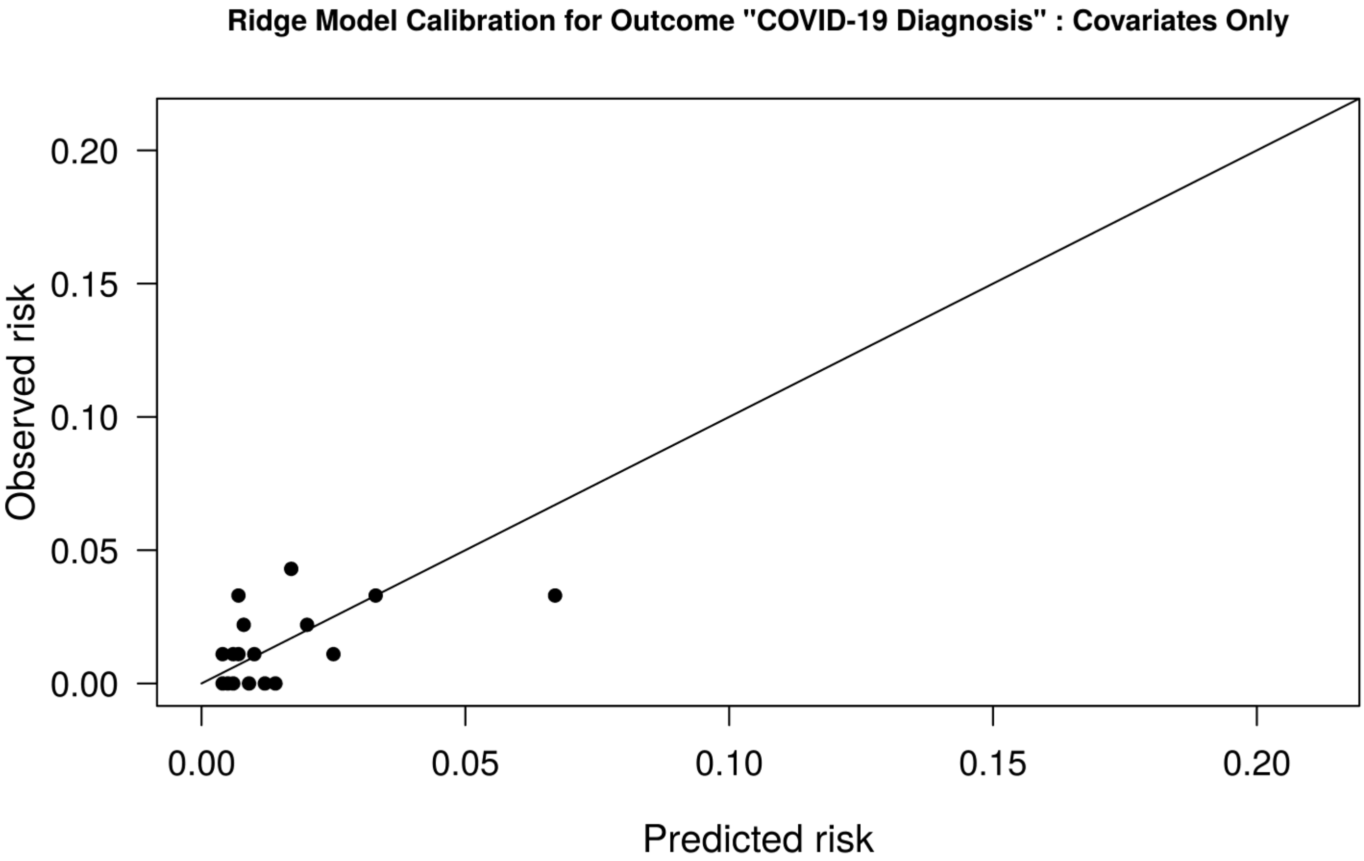

Subjects were split into 20 groups based on their predicted risk, and the observed proportion of COVID-19 tested subject in those groups was plotted against the expected proportion as per the model. The model used for this plot was from the first random 70/30 train-test split out of 100 total.

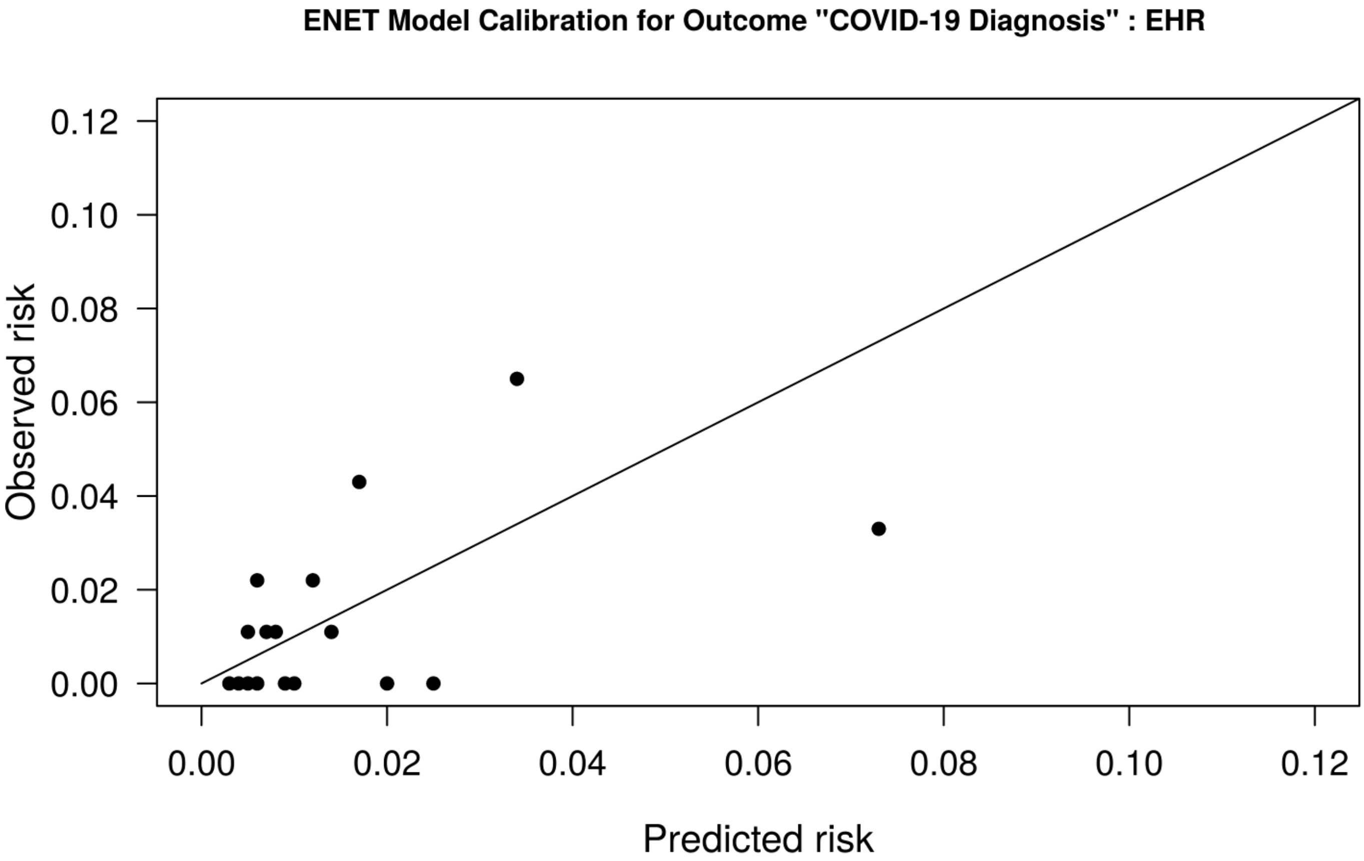

Subjects were split into 20 groups based on their predicted risk, and the observed proportion of COVID-19 tested subject in those groups was plotted against the expected proportion as per the model. The model used for this plot was from the first random 70/30 train-test split out of 100 total.

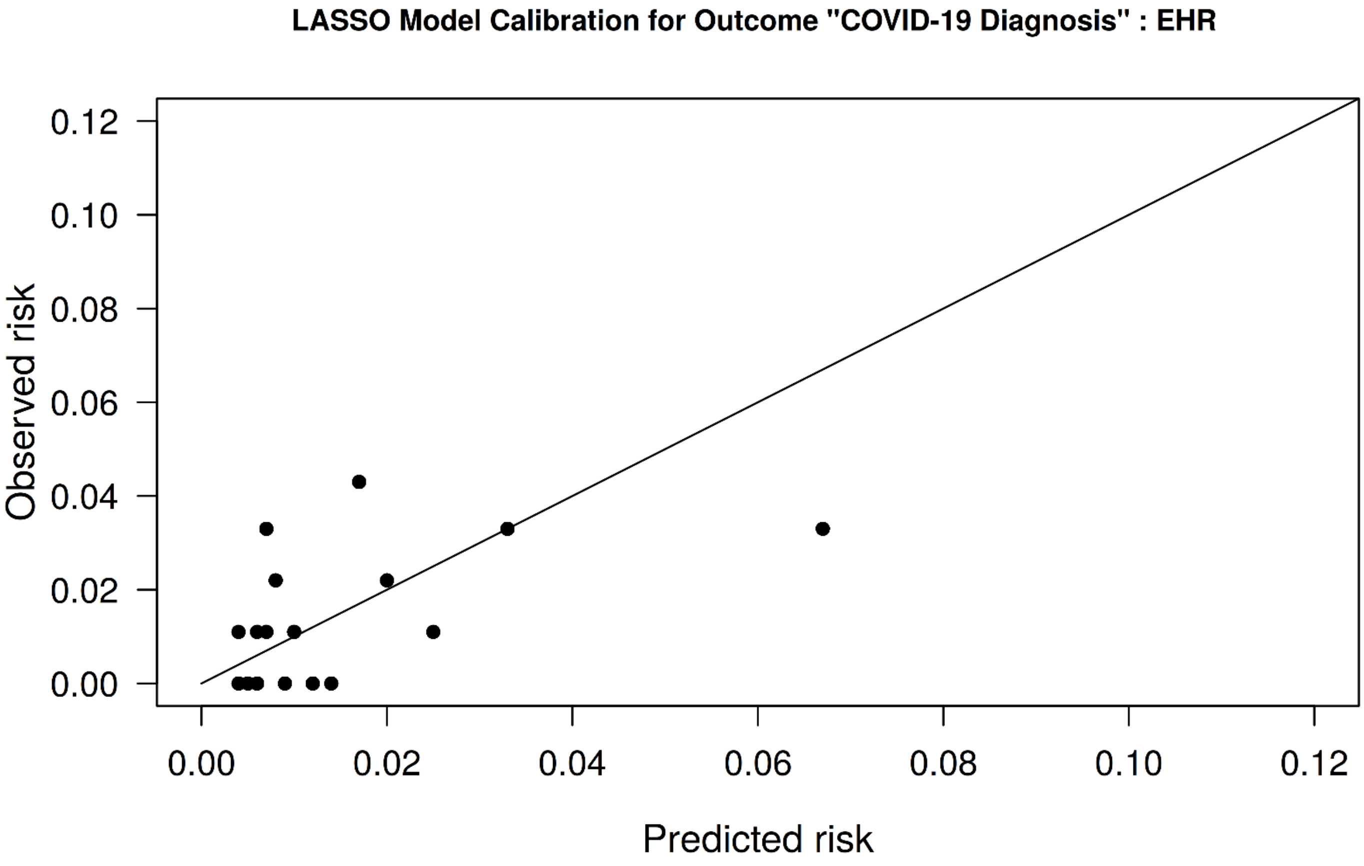

Subjects were split into 20 groups based on their predicted risk, and the observed proportion of COVID-19 tested subject in those groups was plotted against the expected proportion as per the model. The model used for this plot was from the first random 70/30 train-test split out of 100 total.

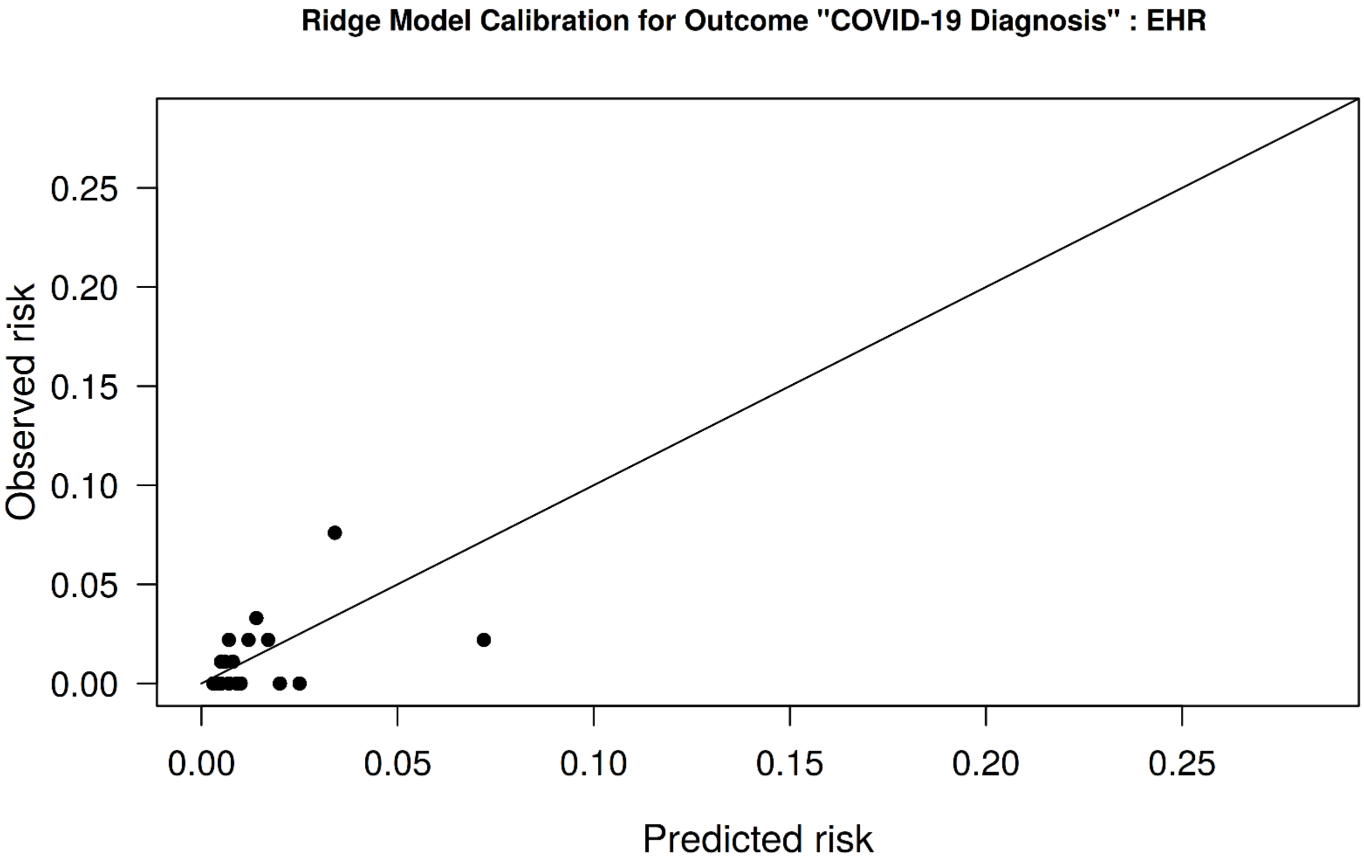

Subjects were split into 20 groups based on their predicted risk, and the observed proportion of COVID-19 tested subject in those groups was plotted against the expected proportion as per the model. The model used for this plot was from the first random 70/30 train-test split out of 100 total.

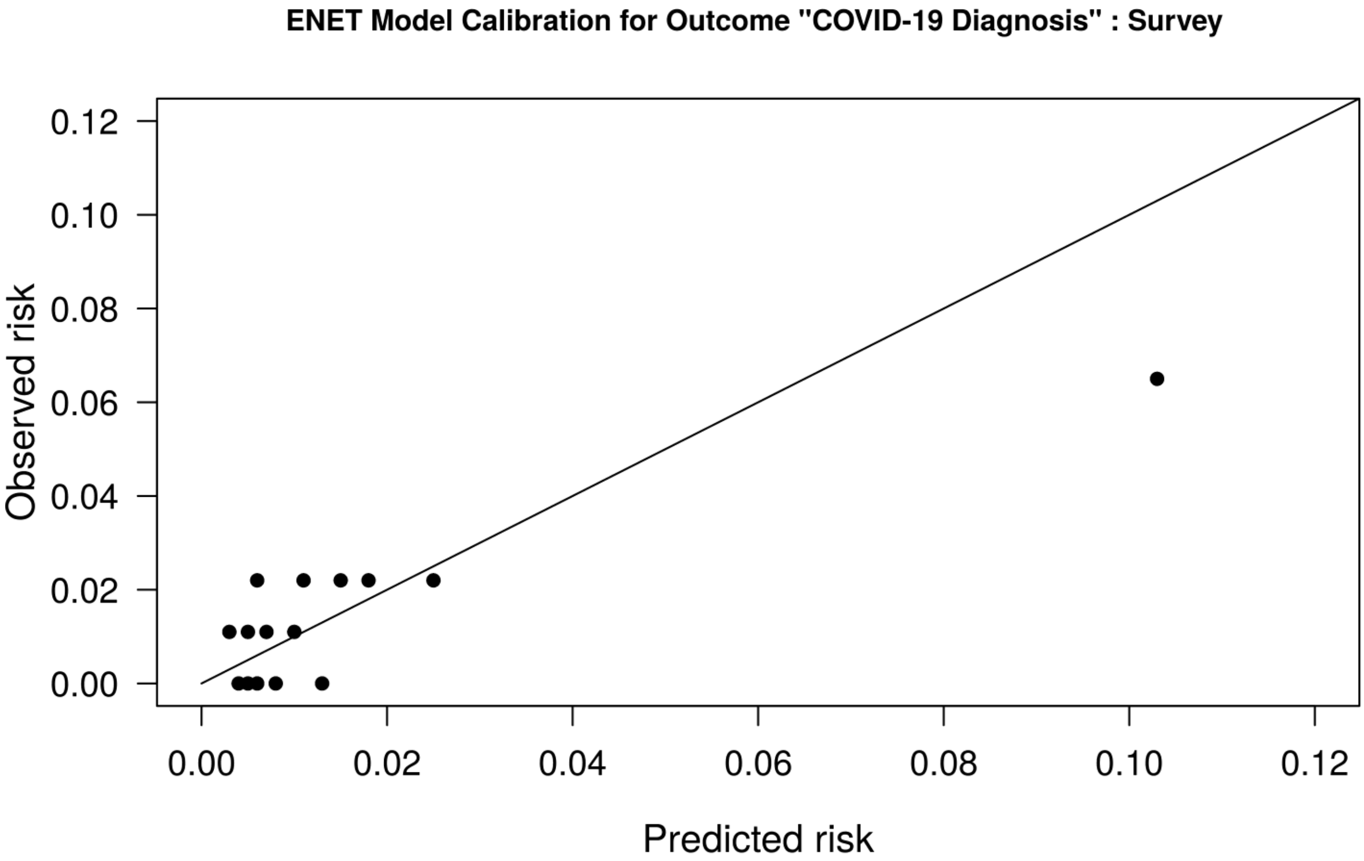

Subjects were split into 20 groups based on their predicted risk, and the observed proportion of COVID-19 tested subject in those groups was plotted against the expected proportion as per the model. The model used for this plot was from the first random 70/30 train-test split out of 100 total.

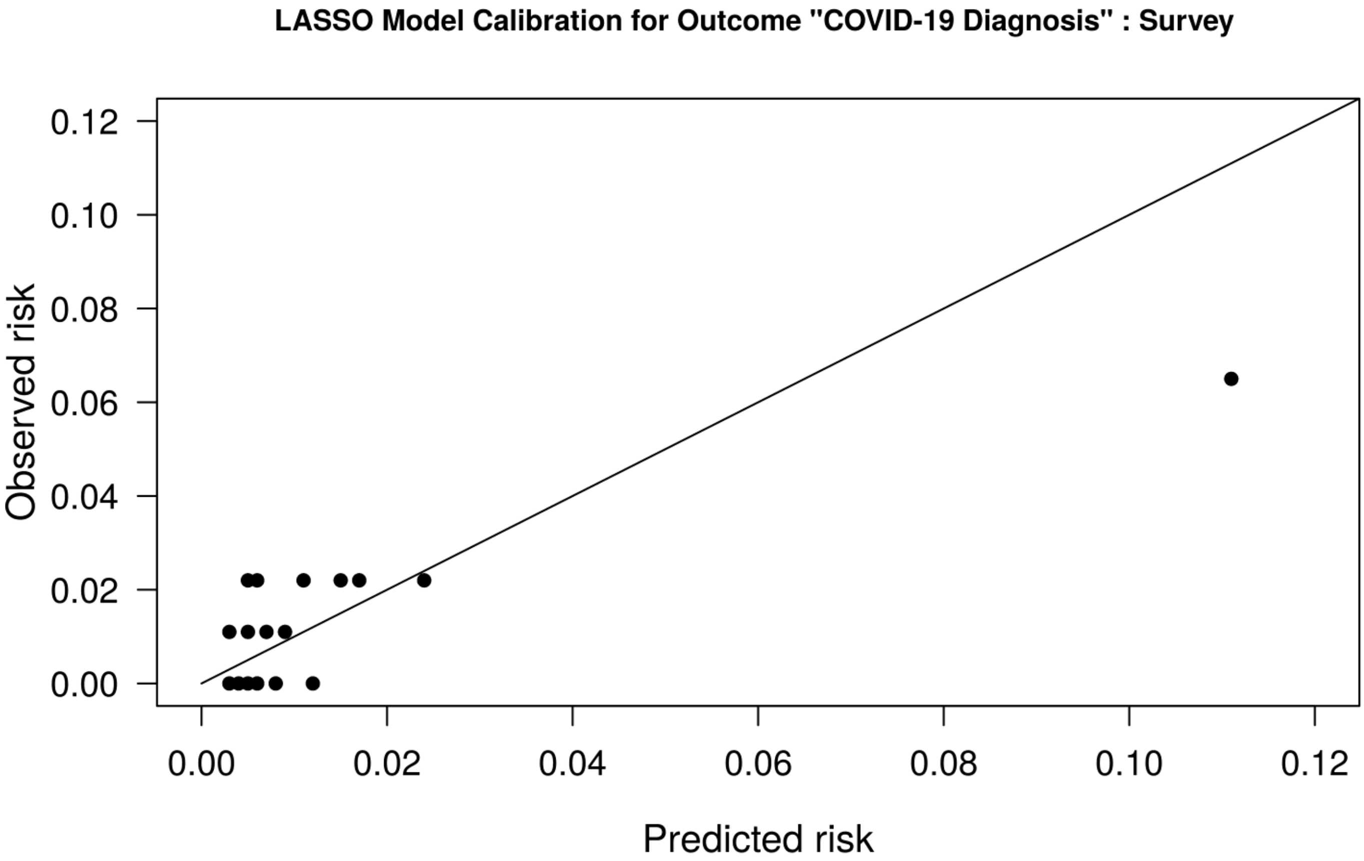

Subjects were split into 20 groups based on their predicted risk, and the observed proportion of COVID-19 tested subject in those groups was plotted against the expected proportion as per the model. The model used for this plot was from the first random 70/30 train-test split out of 100 total.

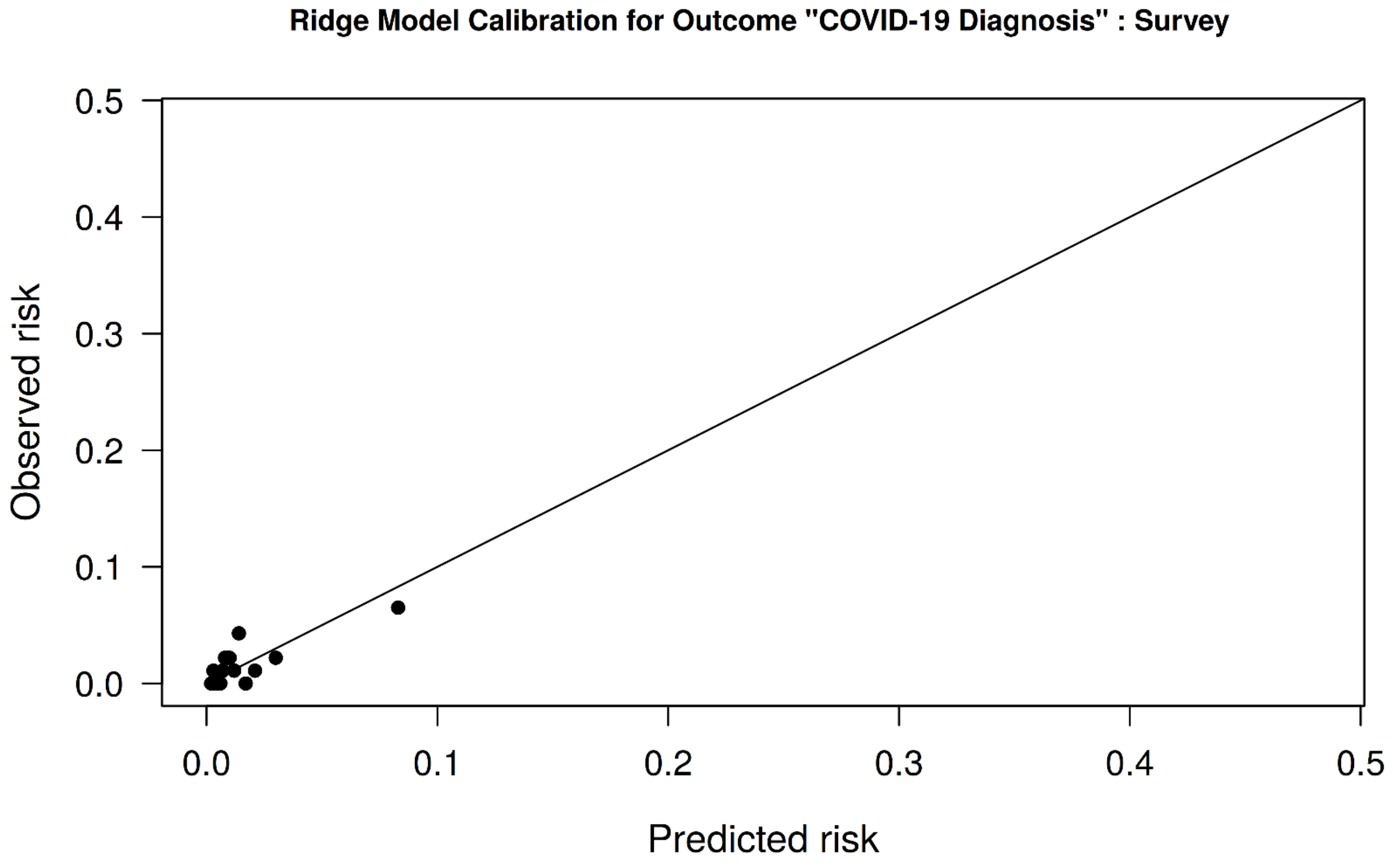

Subjects were split into 20 groups based on their predicted risk, and the observed proportion of COVID-19 tested subject in those groups was plotted against the expected proportion as per the model. The model used for this plot was from the first random 70/30 train-test split out of 100 total.

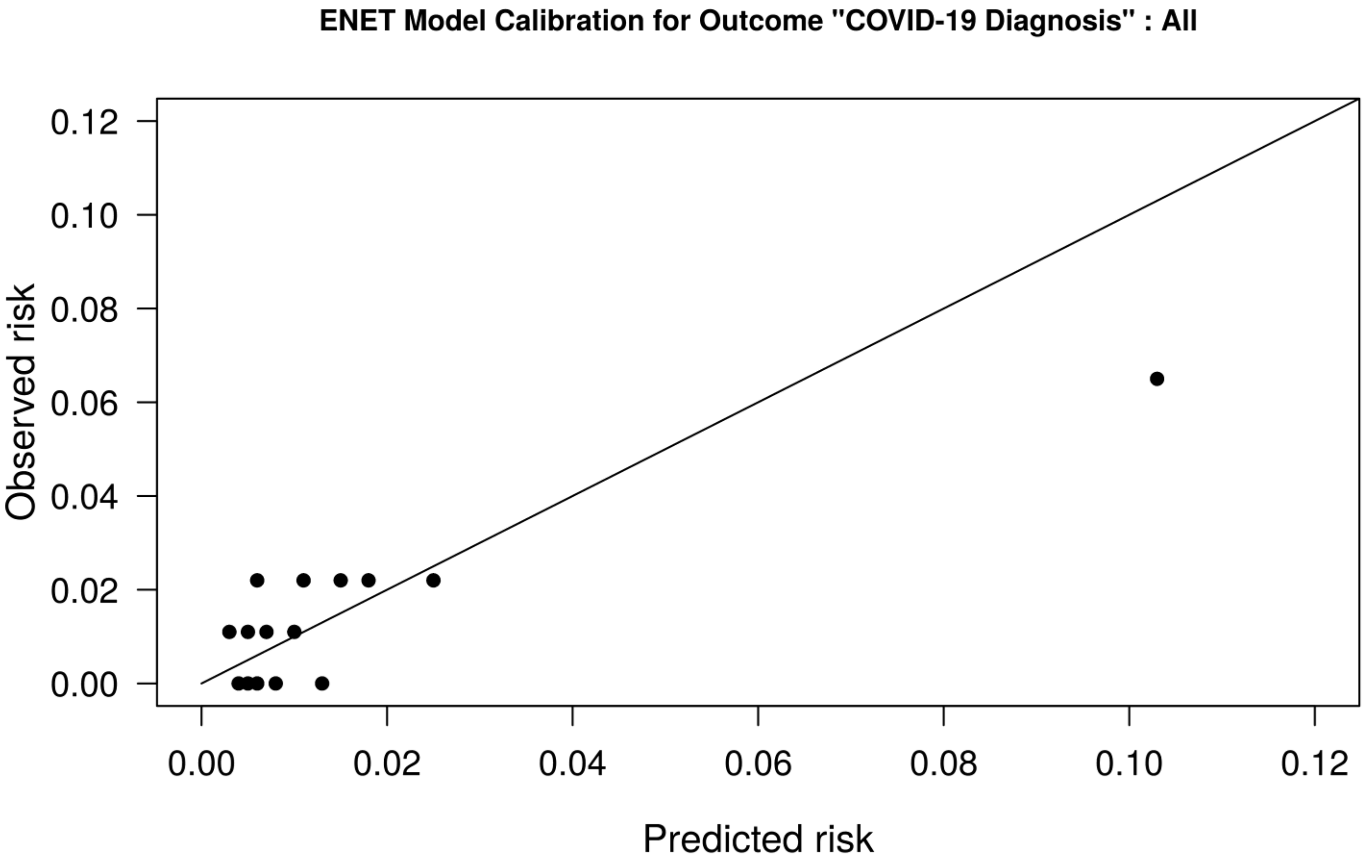

Subjects were split into 20 groups based on their predicted risk, and the observed proportion of COVID-19 tested subject in those groups was plotted against the expected proportion as per the model. The model used for this plot was from the first random 70/30 train-test split out of 100 total.

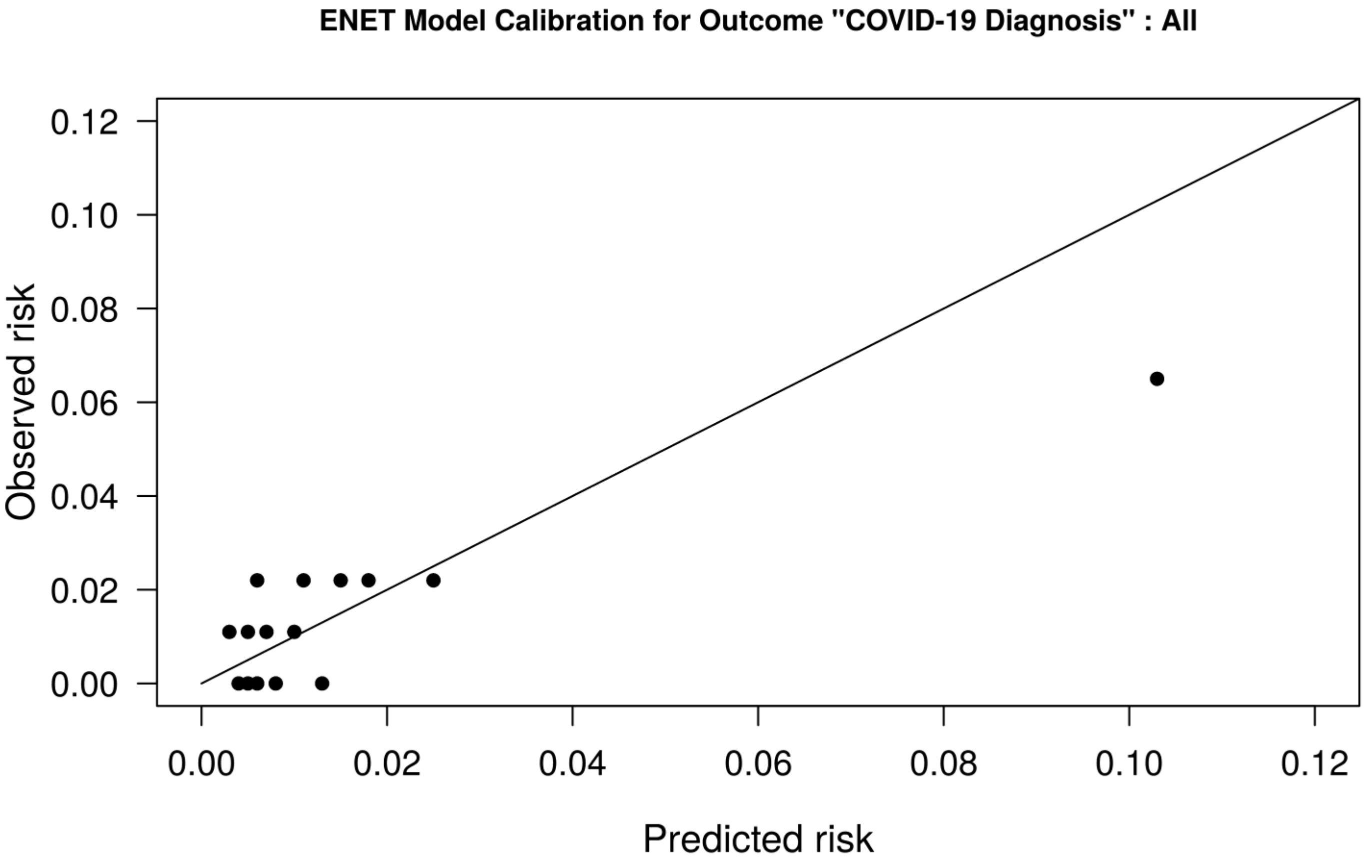

Subjects were split into 20 groups based on their predicted risk, and the observed proportion of COVID-19 tested subject in those groups was plotted against the expected proportion as per the model. The model used for this plot was from the first random 70/30 train-test split out of 100 total.

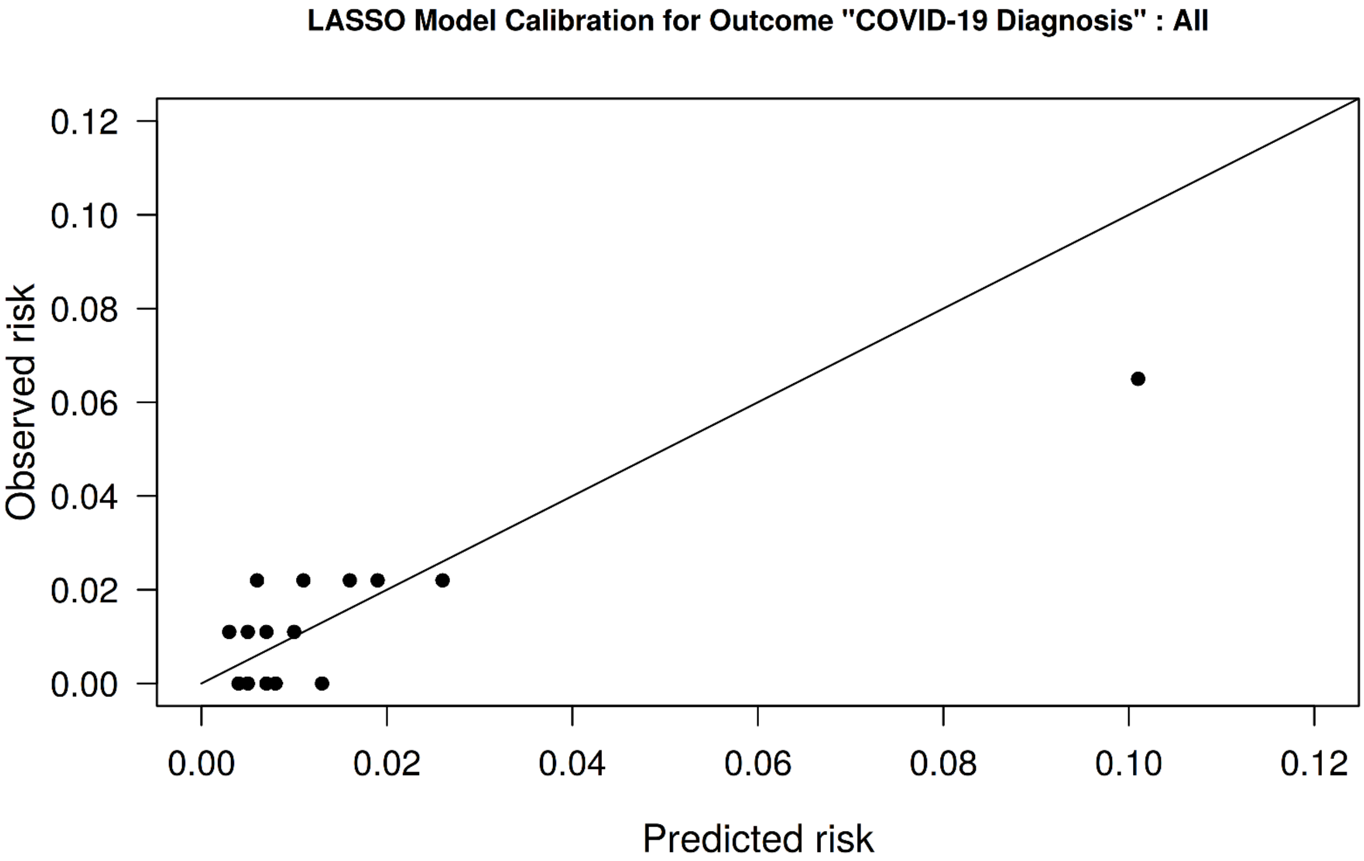

Subjects were split into 20 groups based on their predicted risk, and the observed proportion of COVID-19 tested subject in those groups was plotted against the expected proportion as per the model. The model used for this plot was from the first random 70/30 train-test split out of 100 total.

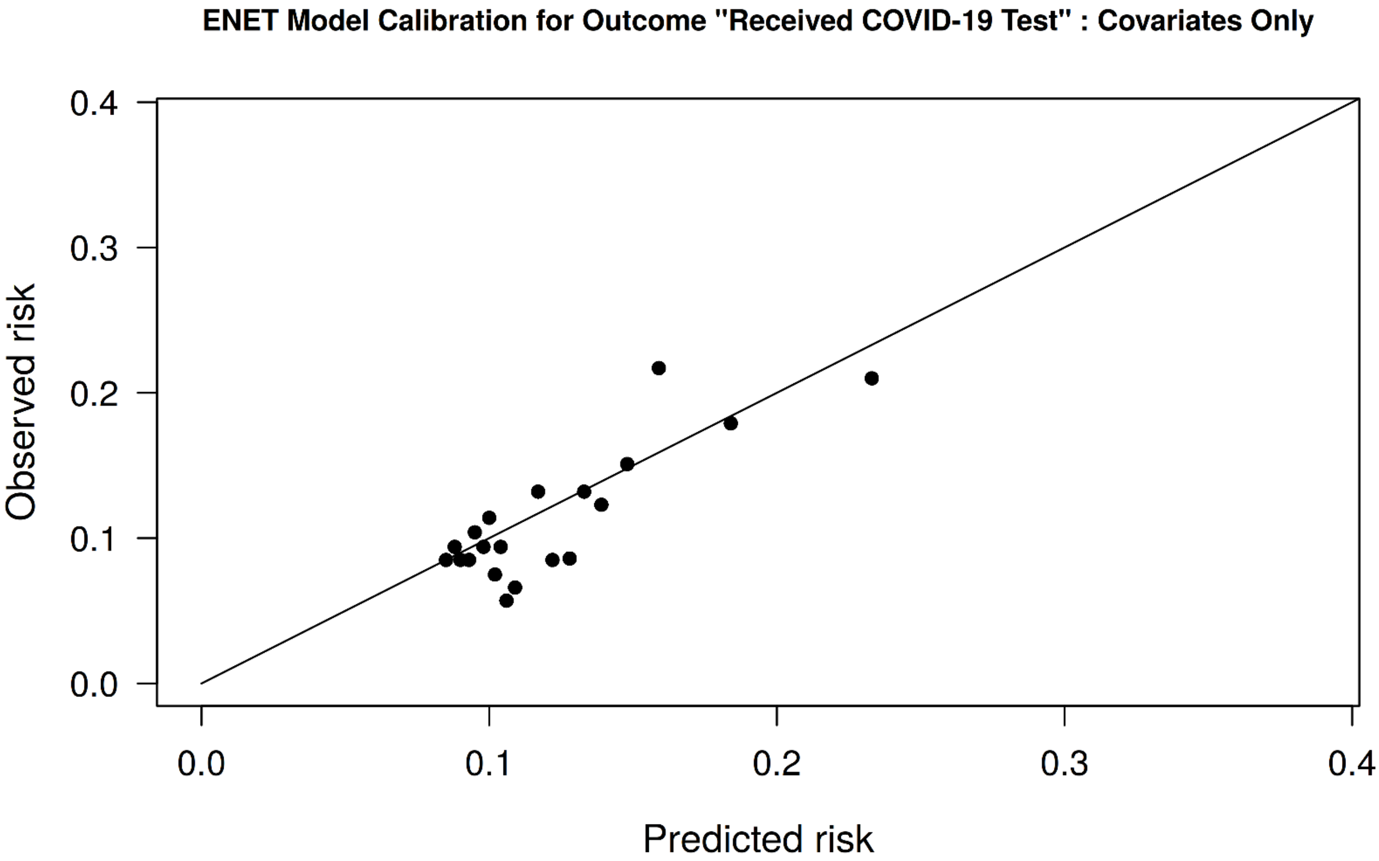

Subjects were split into 20 groups based on their predicted risk, and the observed proportion of COVID-19 tested subject in those groups was plotted against the expected proportion as per the model. The model used for this plot was from the first random 70/30 train-test split out of 100 total.

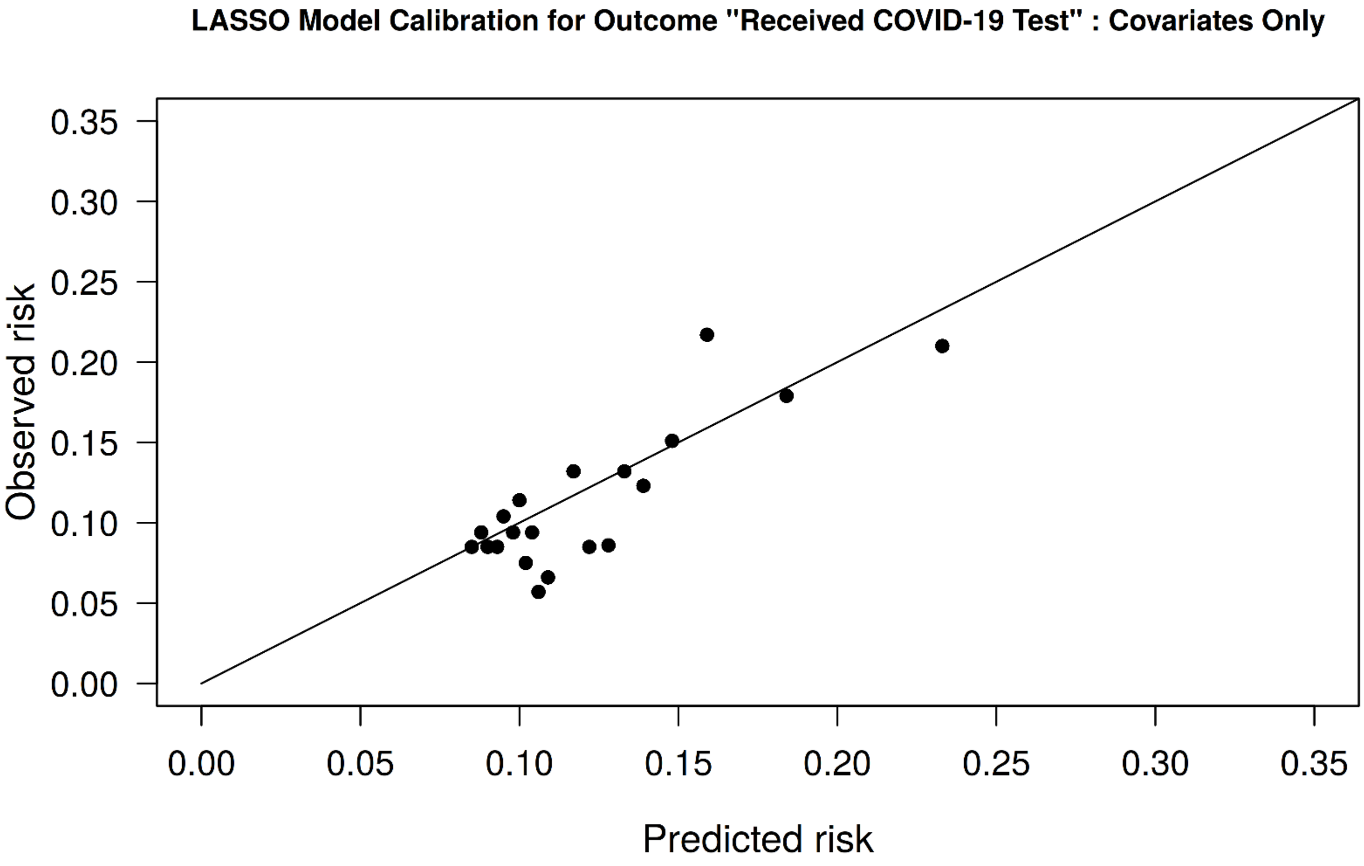

Subjects were split into 20 groups based on their predicted risk, and the observed proportion of COVID-19 tested subject in those groups was plotted against the expected proportion as per the model. The model used for this plot was from the first random 70/30 train-test split out of 100 total.

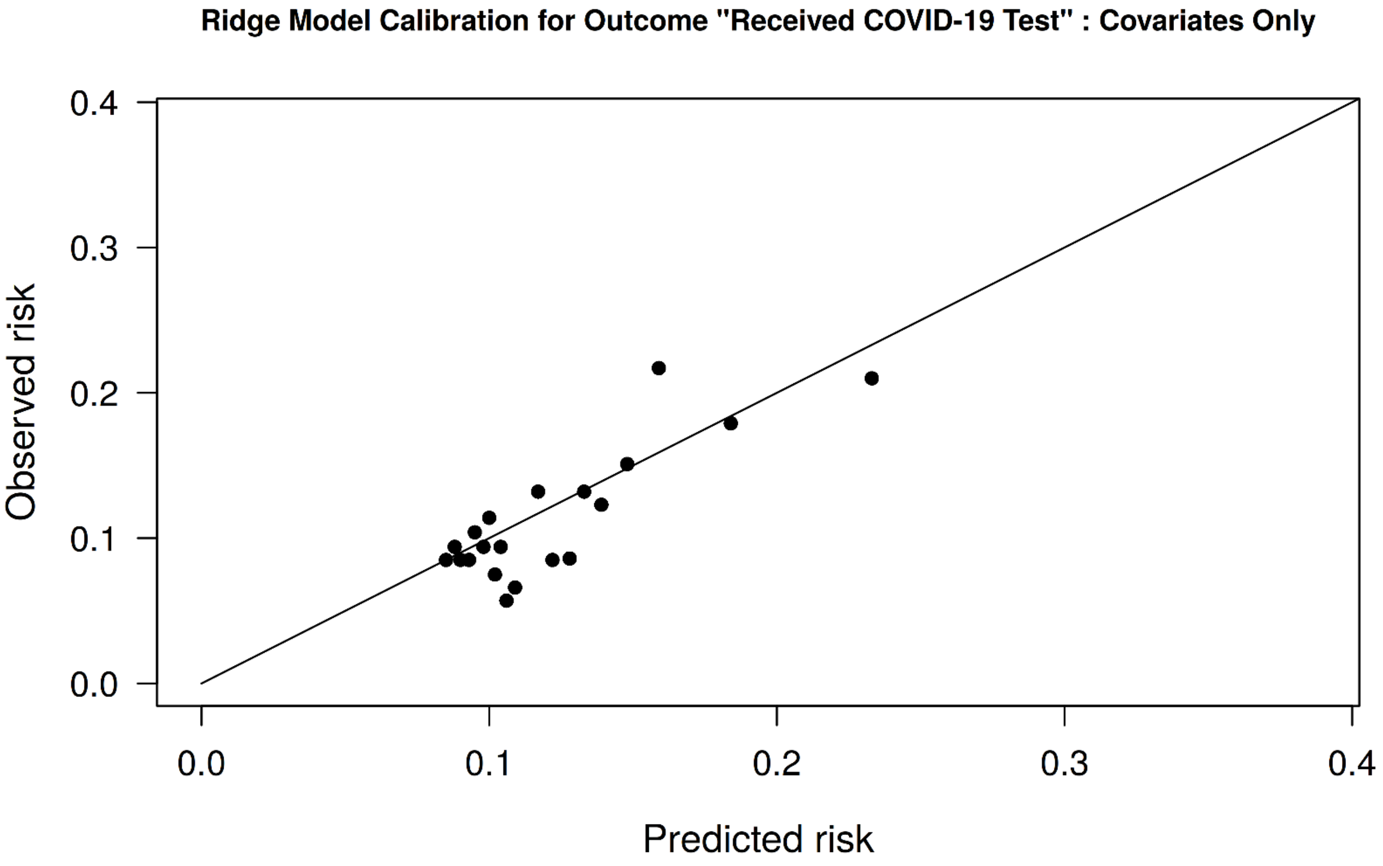

Subjects were split into 20 groups based on their predicted risk, and the observed proportion of COVID-19 tested subject in those groups was plotted against the expected proportion as per the model. The model used for this plot was from the first random 70/30 train-test split out of 100 total.

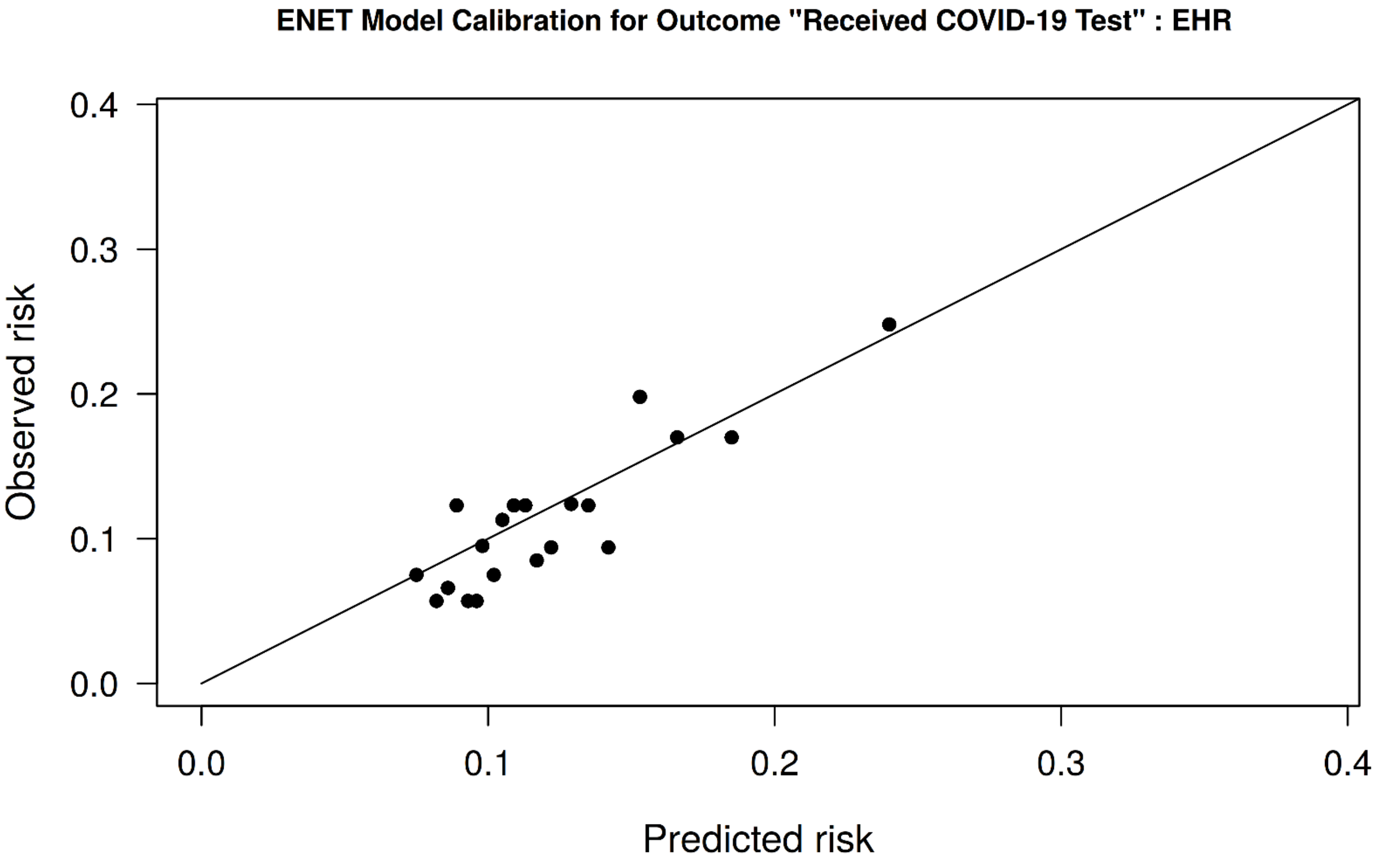

Subjects were split into 20 groups based on their predicted risk, and the observed proportion of COVID-19 tested subject in those groups was plotted against the expected proportion as per the model. The model used for this plot was from the first random 70/30 train-test split out of 100 total.

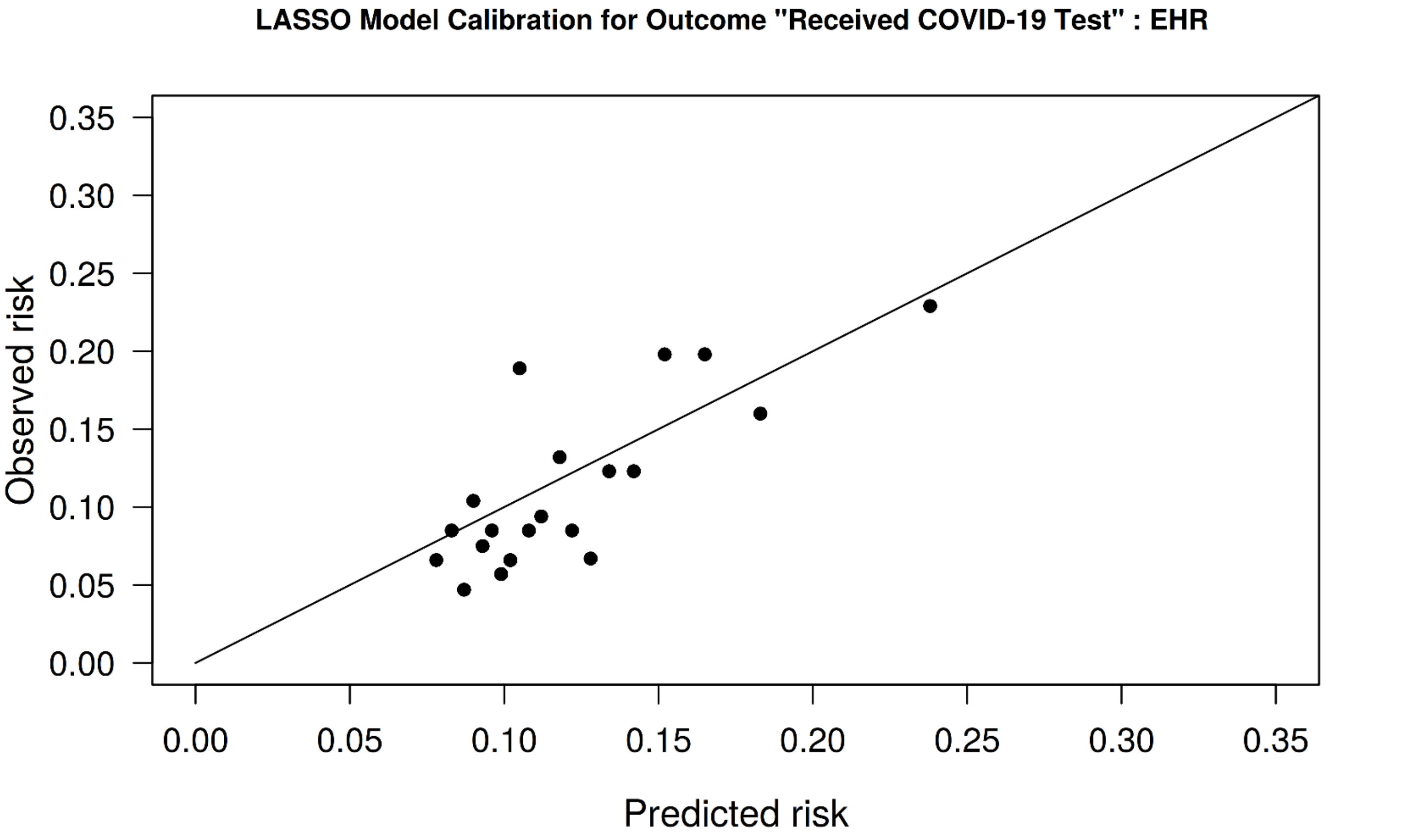

Subjects were split into 20 groups based on their predicted risk, and the observed proportion of COVID-19 tested subject in those groups was plotted against the expected proportion as per the model. The model used for this plot was from the first random 70/30 train-test split out of 100 total.

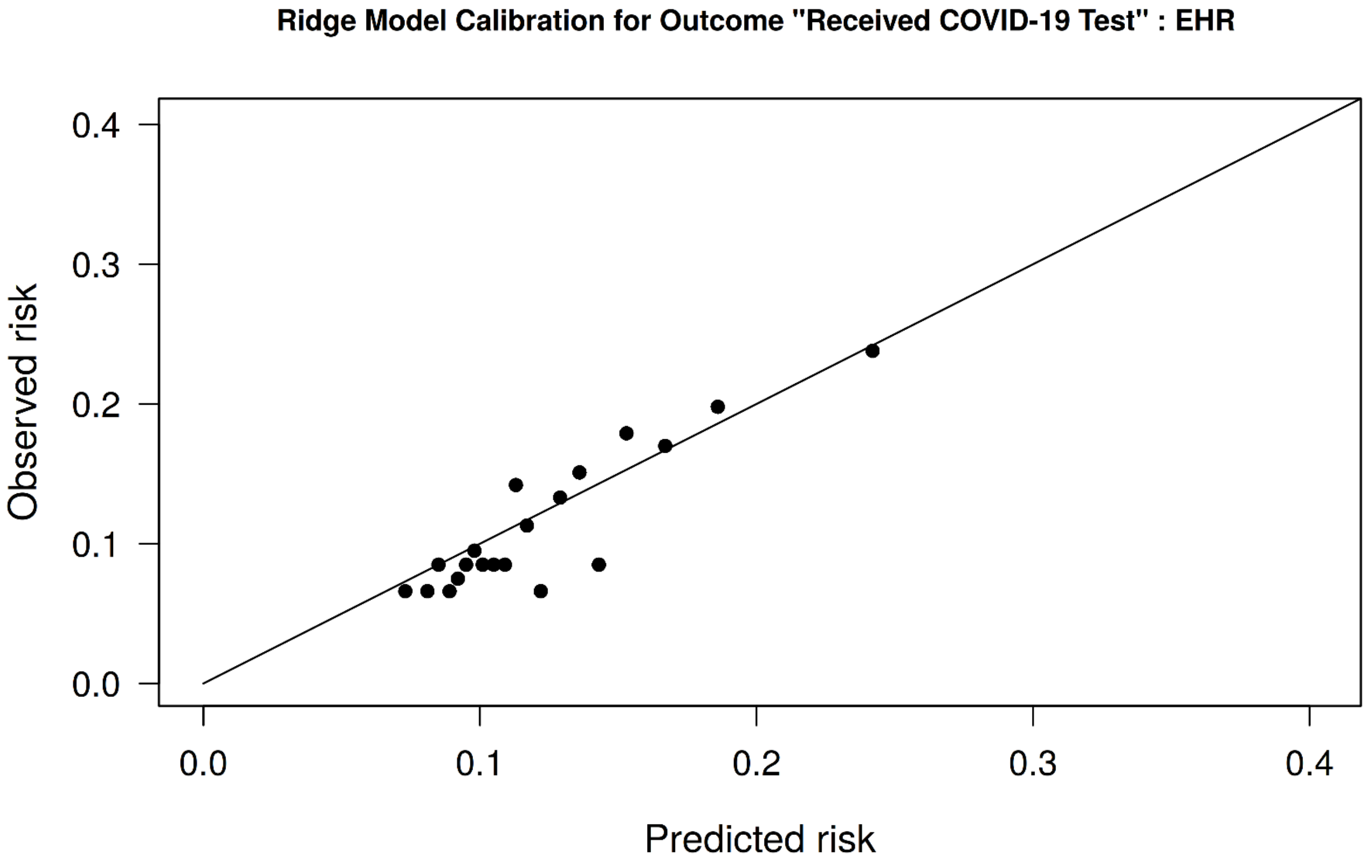

Subjects were split into 20 groups based on their predicted risk, and the observed proportion of COVID-19 tested subject in those groups was plotted against the expected proportion as per the model. The model used for this plot was from the first random 70/30 train-test split out of 100 total.

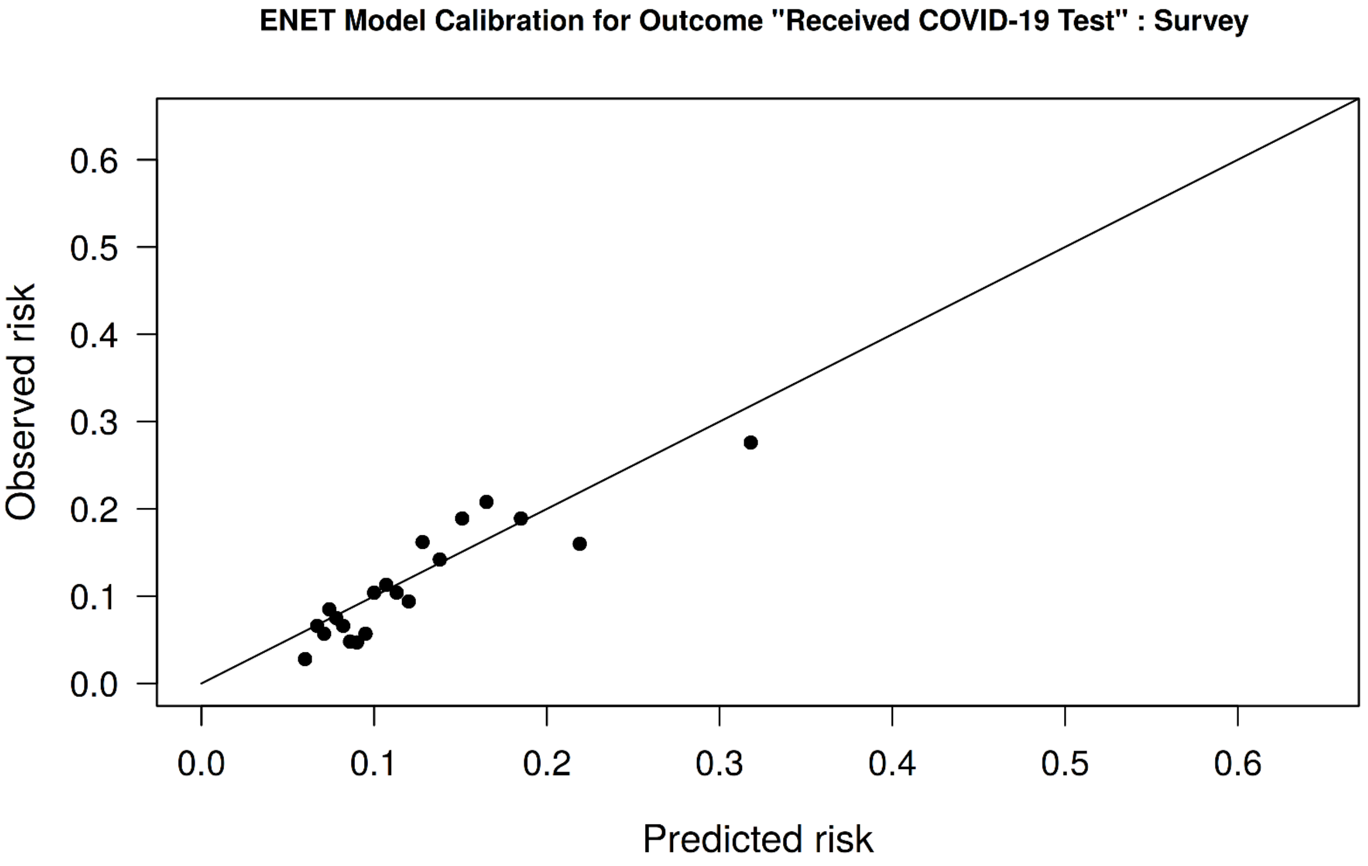

Subjects were split into 20 groups based on their predicted risk, and the observed proportion of COVID-19 tested subject in those groups was plotted against the expected proportion as per the model. The model used for this plot was from the first random 70/30 train-test split out of 100 total.

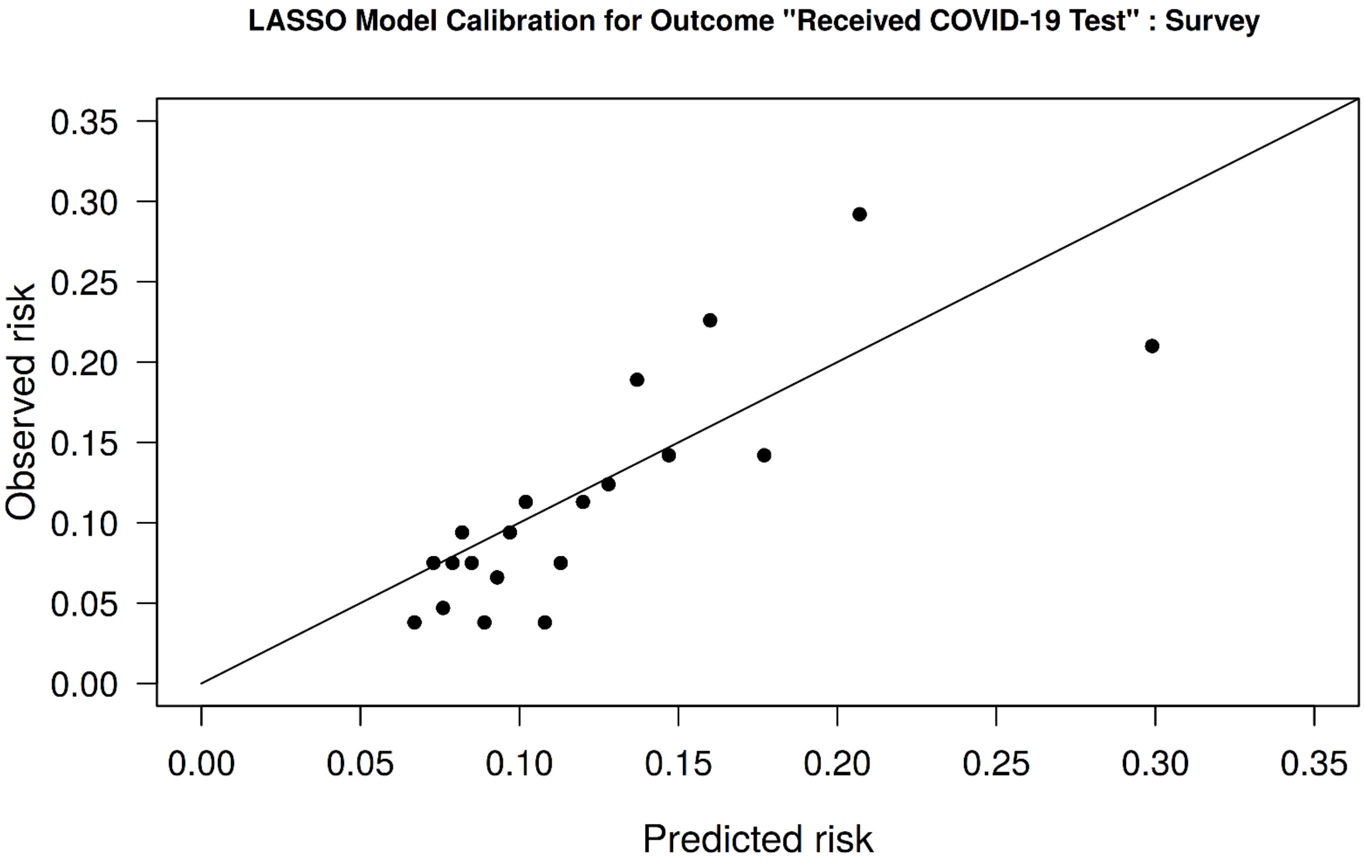

Subjects were split into 20 groups based on their predicted risk, and the observed proportion of COVID-19 tested subject in those groups was plotted against the expected proportion as per the model. The model used for this plot was from the first random 70/30 train-test split out of 100 total.

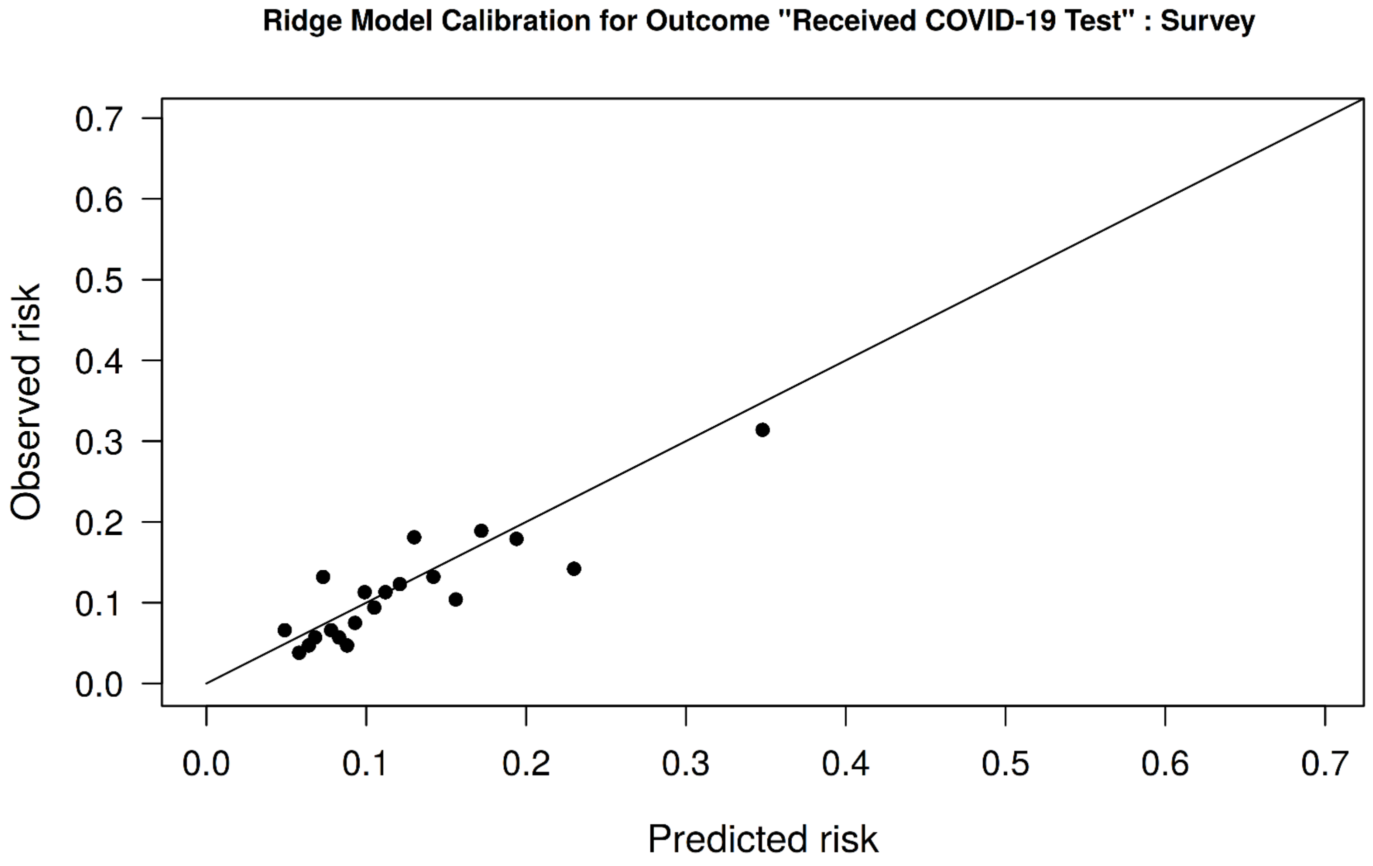

Subjects were split into 20 groups based on their predicted risk, and the observed proportion of COVID-19 tested subject in those groups was plotted against the expected proportion as per the model. The model used for this plot was from the first random 70/30 train-test split out of 100 total.

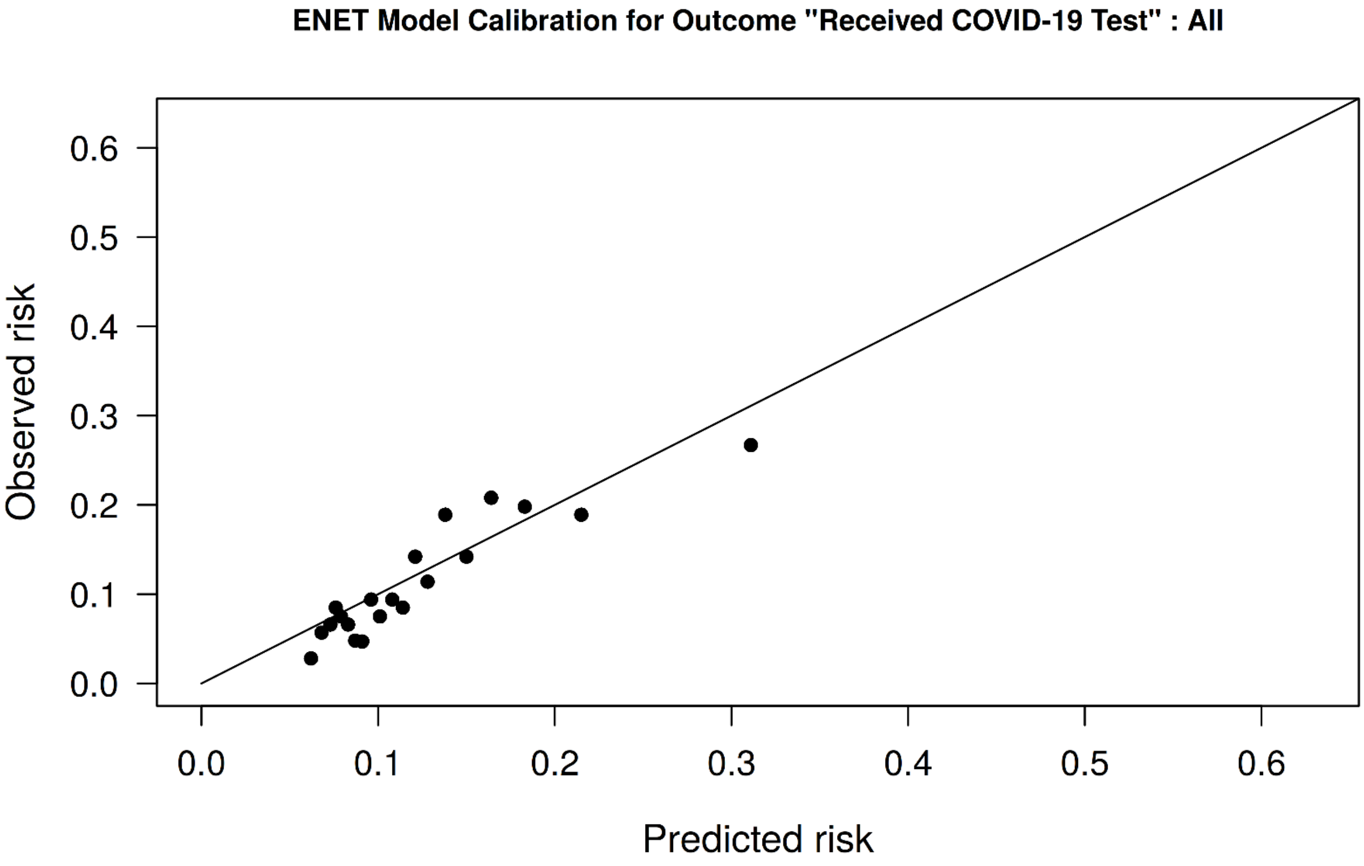

Subjects were split into 20 groups based on their predicted risk, and the observed proportion of COVID-19 tested subject in those groups was plotted against the expected proportion as per the model. The model used for this plot was from the first random 70/30 train-test split out of 100 total.

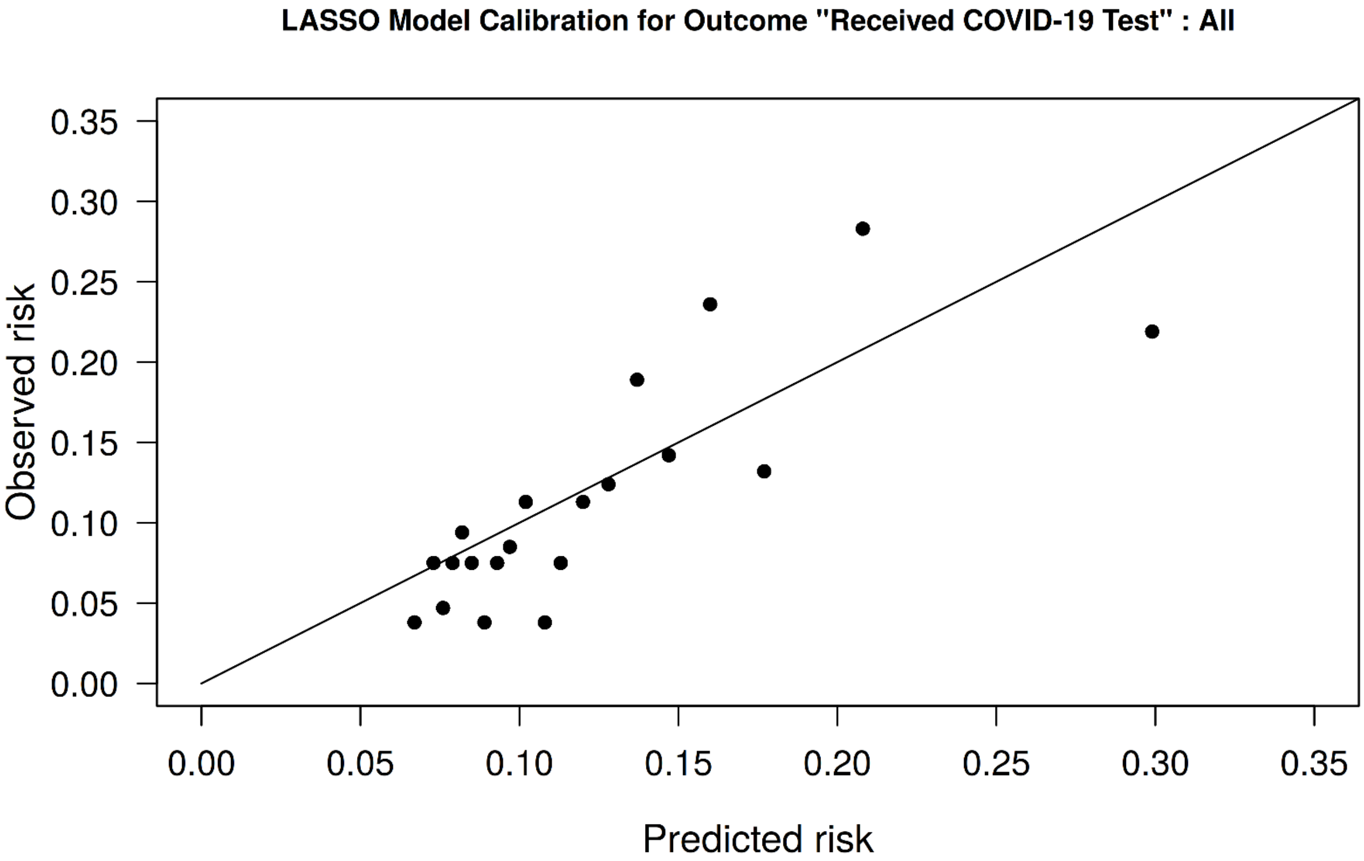

Subjects were split into 20 groups based on their predicted risk, and the observed proportion of COVID-19 tested subject in those groups was plotted against the expected proportion as per the model. The model used for this plot was from the first random 70/30 train-test split out of 100 total.

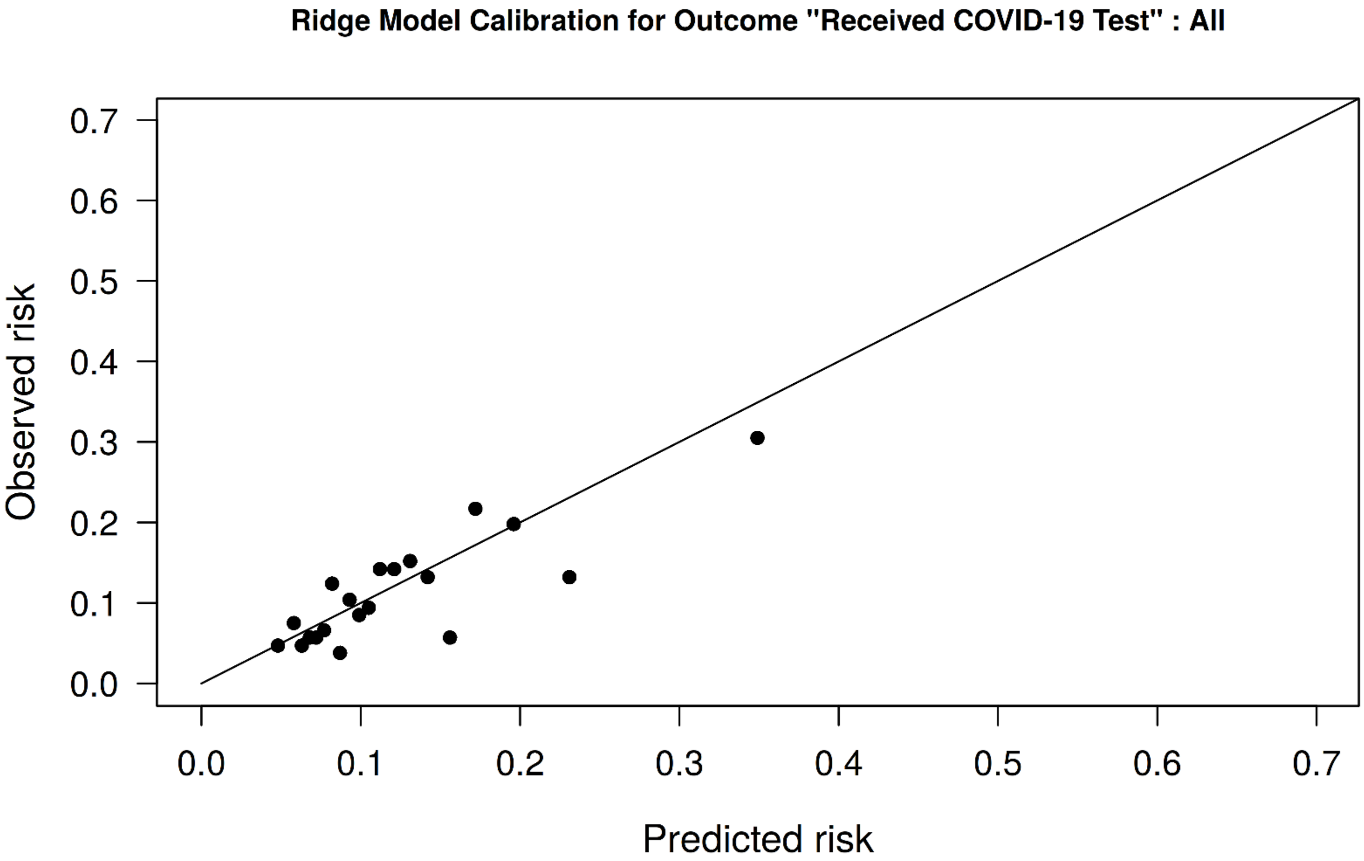

Subjects were split into 20 groups based on their predicted risk, and the observed proportion of COVID-19 tested subject in those groups was plotted against the expected proportion as per the model. The model used for this plot was from the first random 70/30 train-test split out of 100 total.

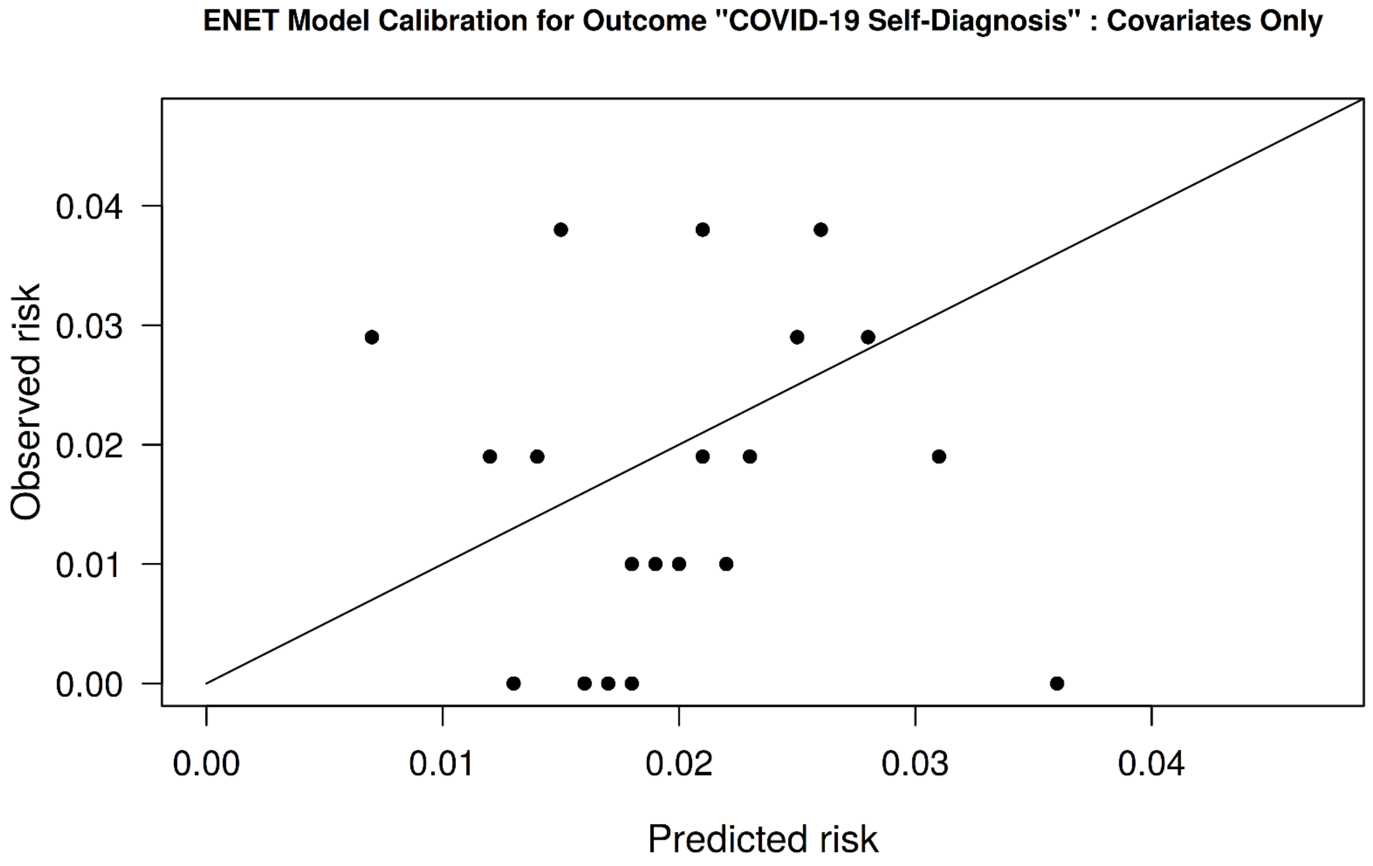

Subjects were split into 20 groups based on their predicted risk, and the observed proportion of COVID-19 tested subject in those groups was plotted against the expected proportion as per the model. The model used for this plot was from the first random 70/30 train-test split out of 100 total.

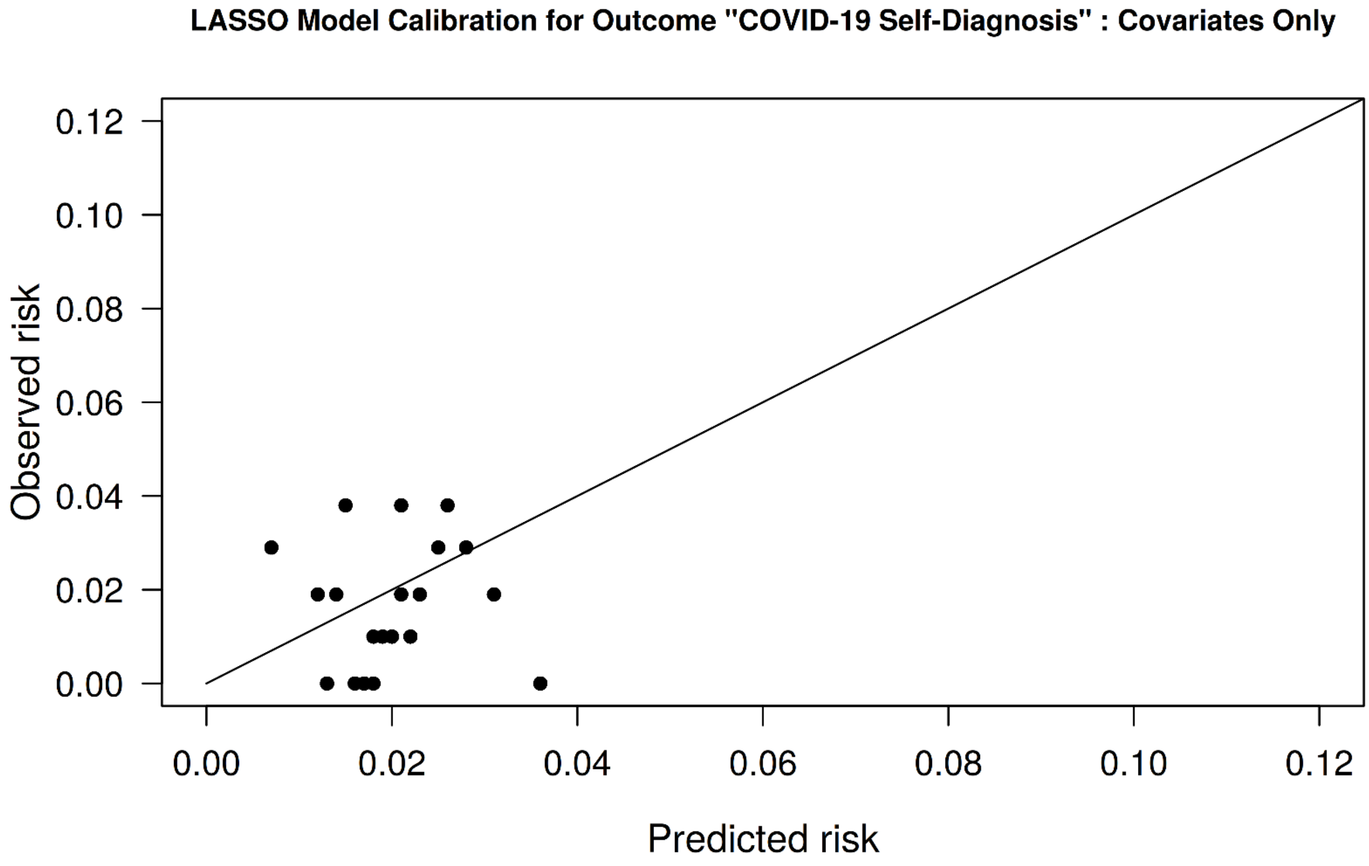

Subjects were split into 20 groups based on their predicted risk, and the observed proportion of COVID-19 tested subject in those groups was plotted against the expected proportion as per the model. The model used for this plot was from the first random 70/30 train-test split out of 100 total.

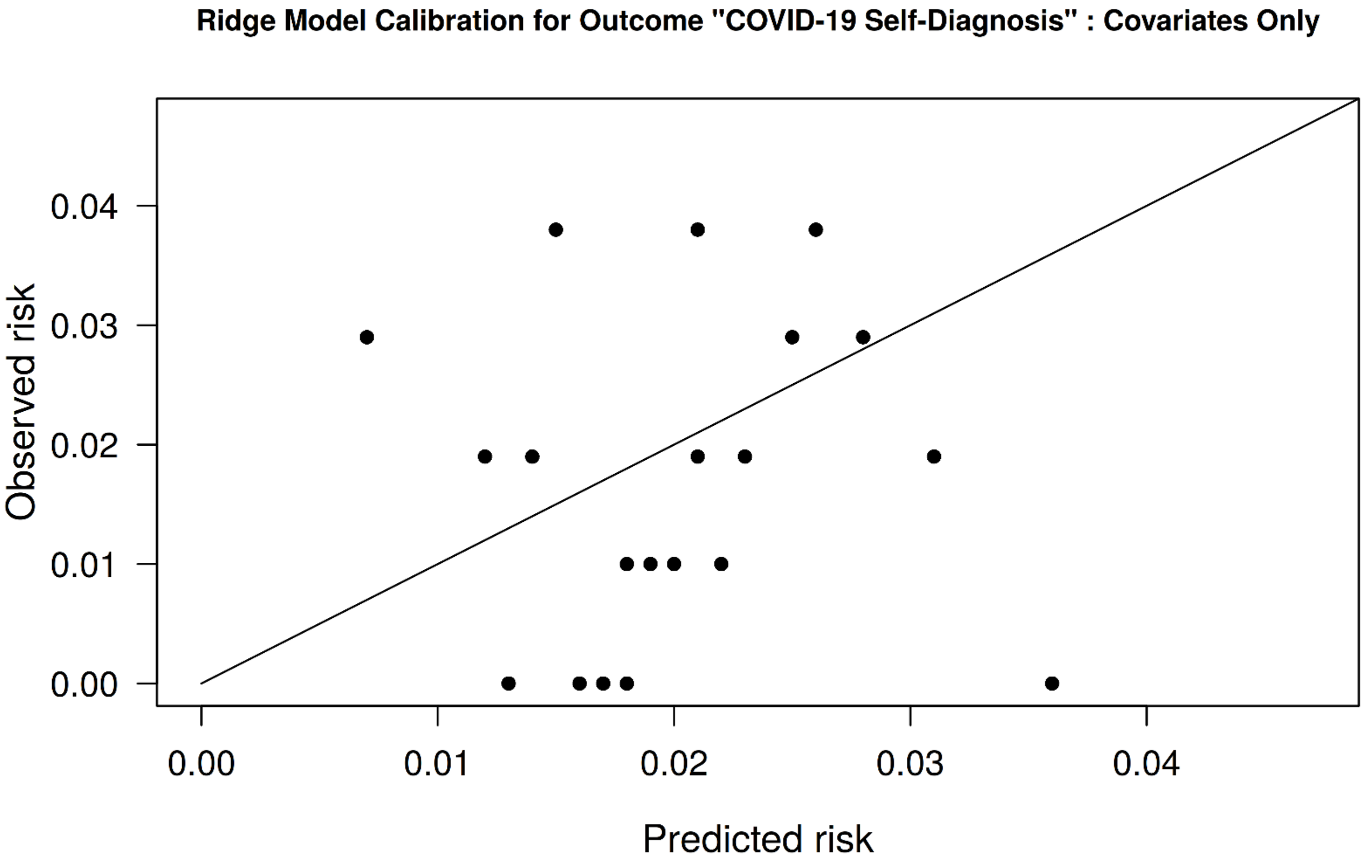

Subjects were split into 20 groups based on their predicted risk, and the observed proportion of COVID-19 tested subject in those groups was plotted against the expected proportion as per the model. The model used for this plot was from the first random 70/30 train-test split out of 100 total.

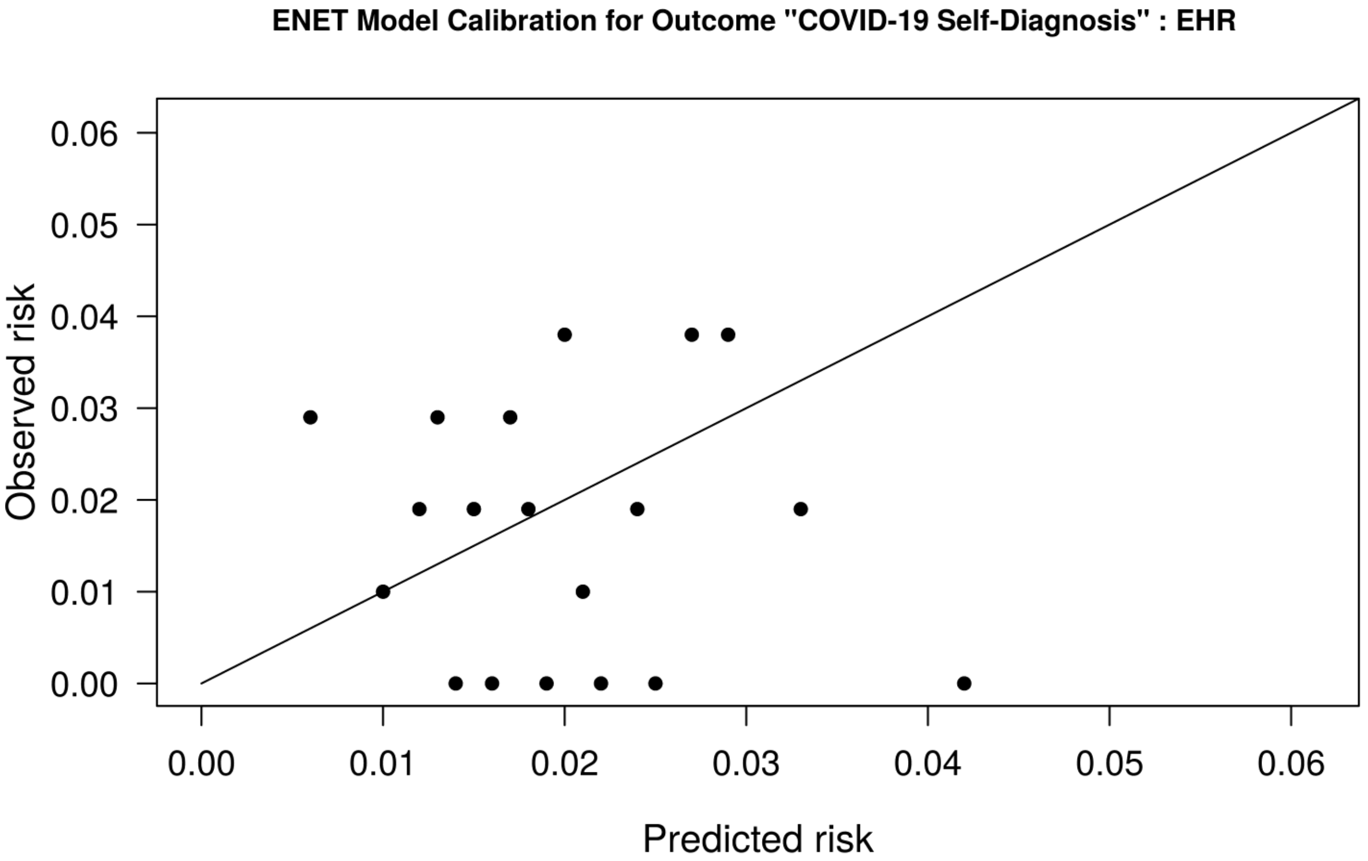

Subjects were split into 20 groups based on their predicted risk, and the observed proportion of COVID-19 tested subject in those groups was plotted against the expected proportion as per the model. The model used for this plot was from the first random 70/30 train-test split out of 100 total.

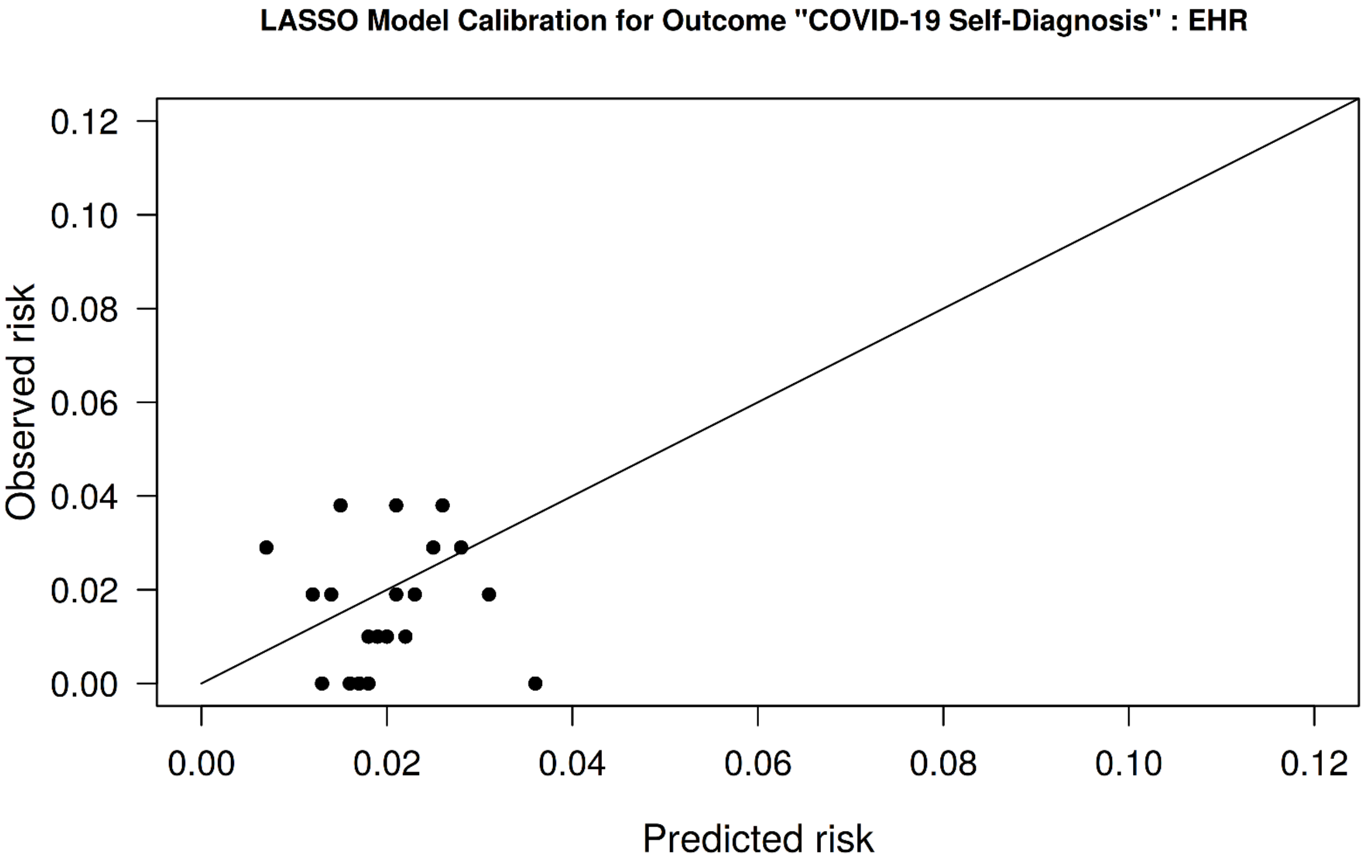

Subjects were split into 20 groups based on their predicted risk, and the observed proportion of COVID-19 tested subject in those groups was plotted against the expected proportion as per the model. The model used for this plot was from the first random 70/30 train-test split out of 100 total.

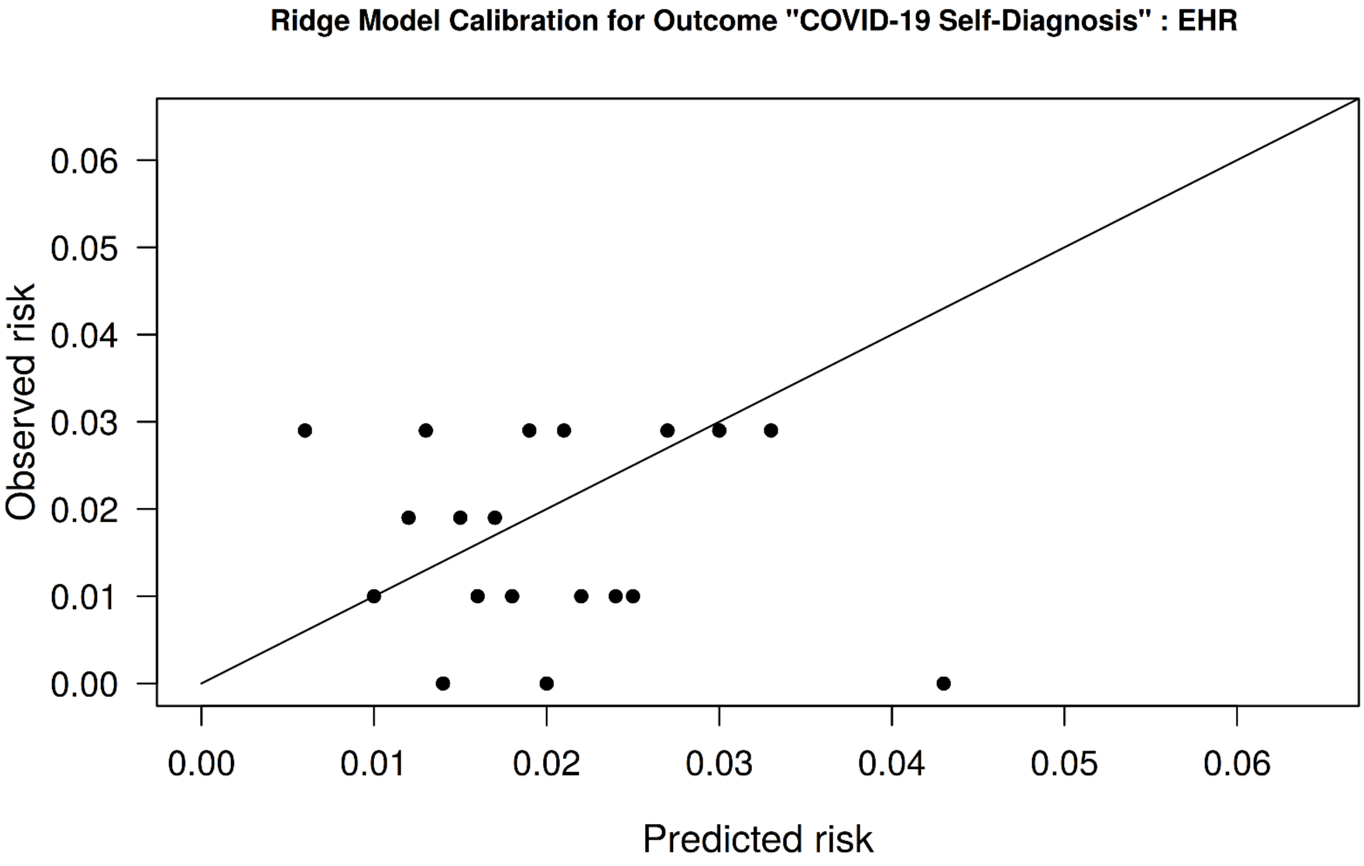

Subjects were split into 20 groups based on their predicted risk, and the observed proportion of COVID-19 tested subject in those groups was plotted against the expected proportion as per the model. The model used for this plot was from the first random 70/30 train-test split out of 100 total.

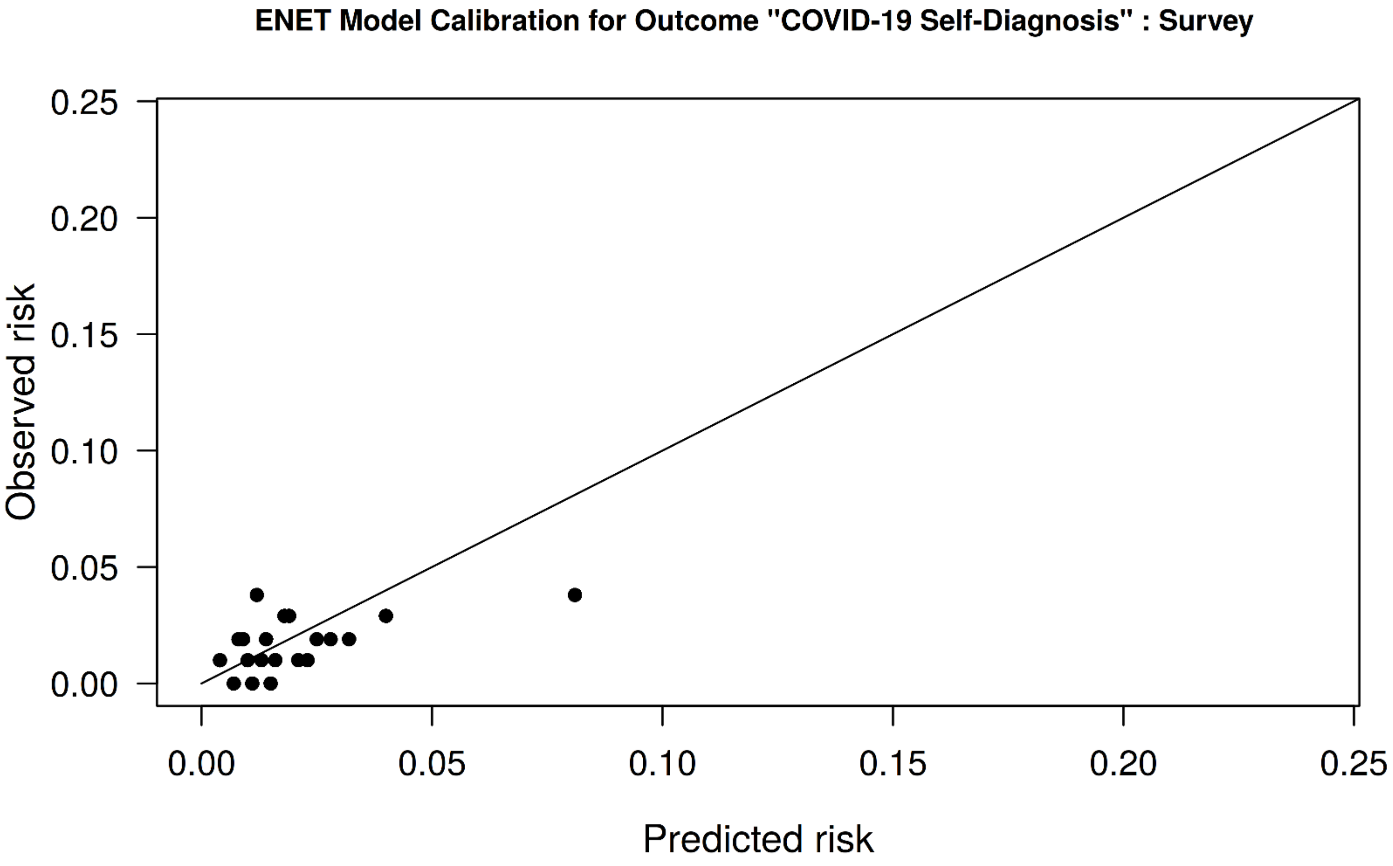

Subjects were split into 20 groups based on their predicted risk, and the observed proportion of COVID-19 tested subject in those groups was plotted against the expected proportion as per the model. The model used for this plot was from the first random 70/30 train-test split out of 100 total.

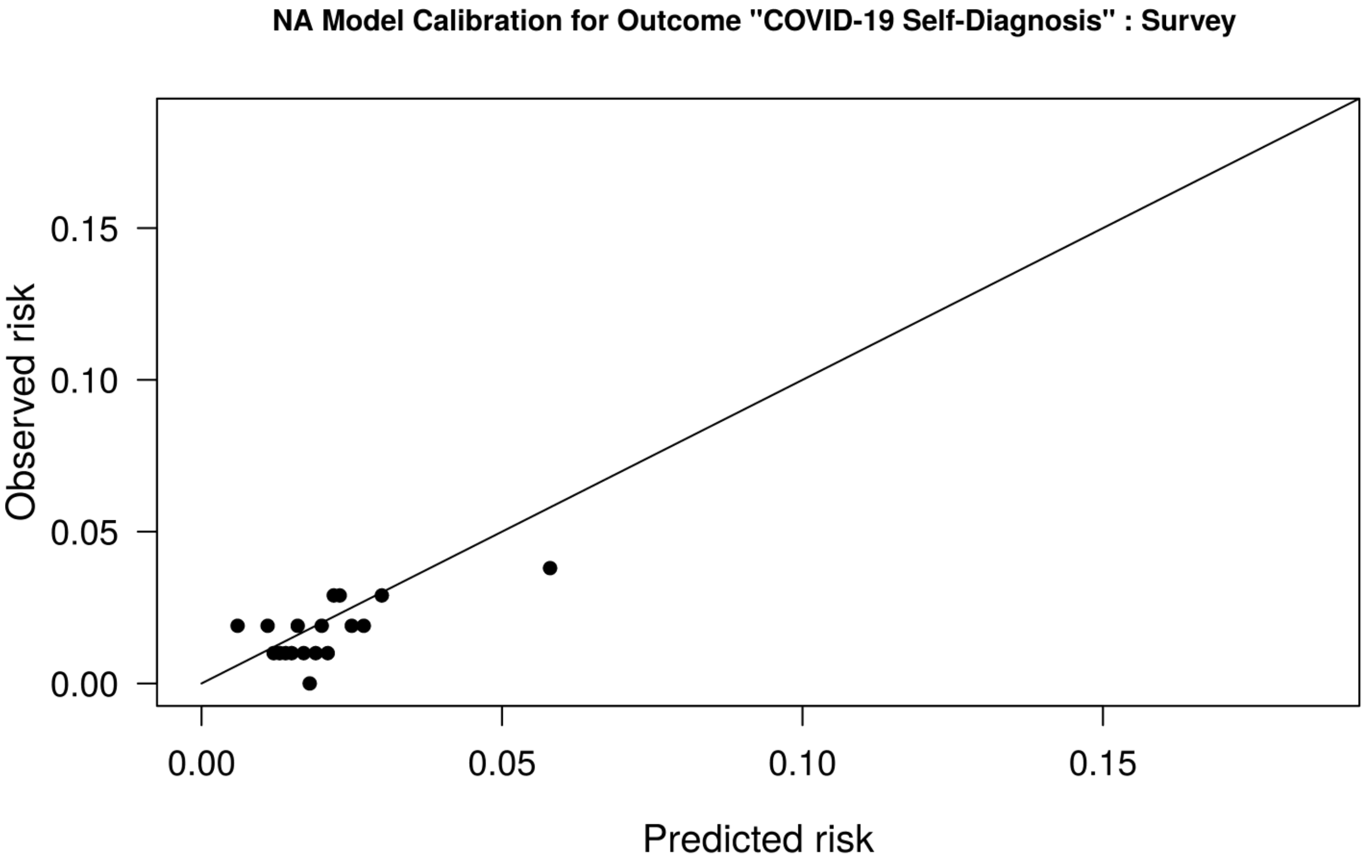

Subjects were split into 20 groups based on their predicted risk, and the observed proportion of COVID-19 tested subject in those groups was plotted against the expected proportion as per the model. The model used for this plot was from the first random 70/30 train-test split out of 100 total.

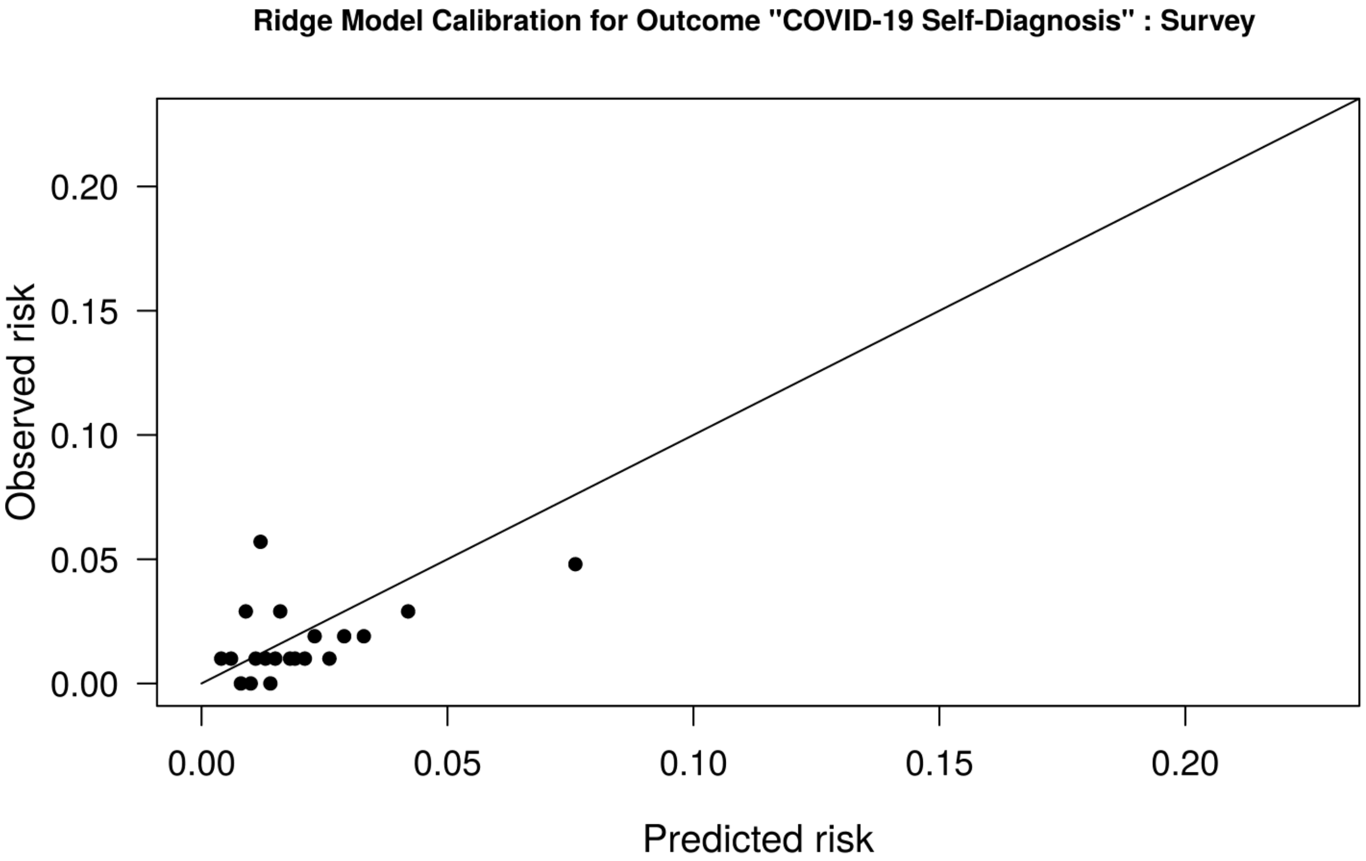

Subjects were split into 20 groups based on their predicted risk, and the observed proportion of COVID-19 tested subject in those groups was plotted against the expected proportion as per the model. The model used for this plot was from the first random 70/30 train-test split out of 100 total.

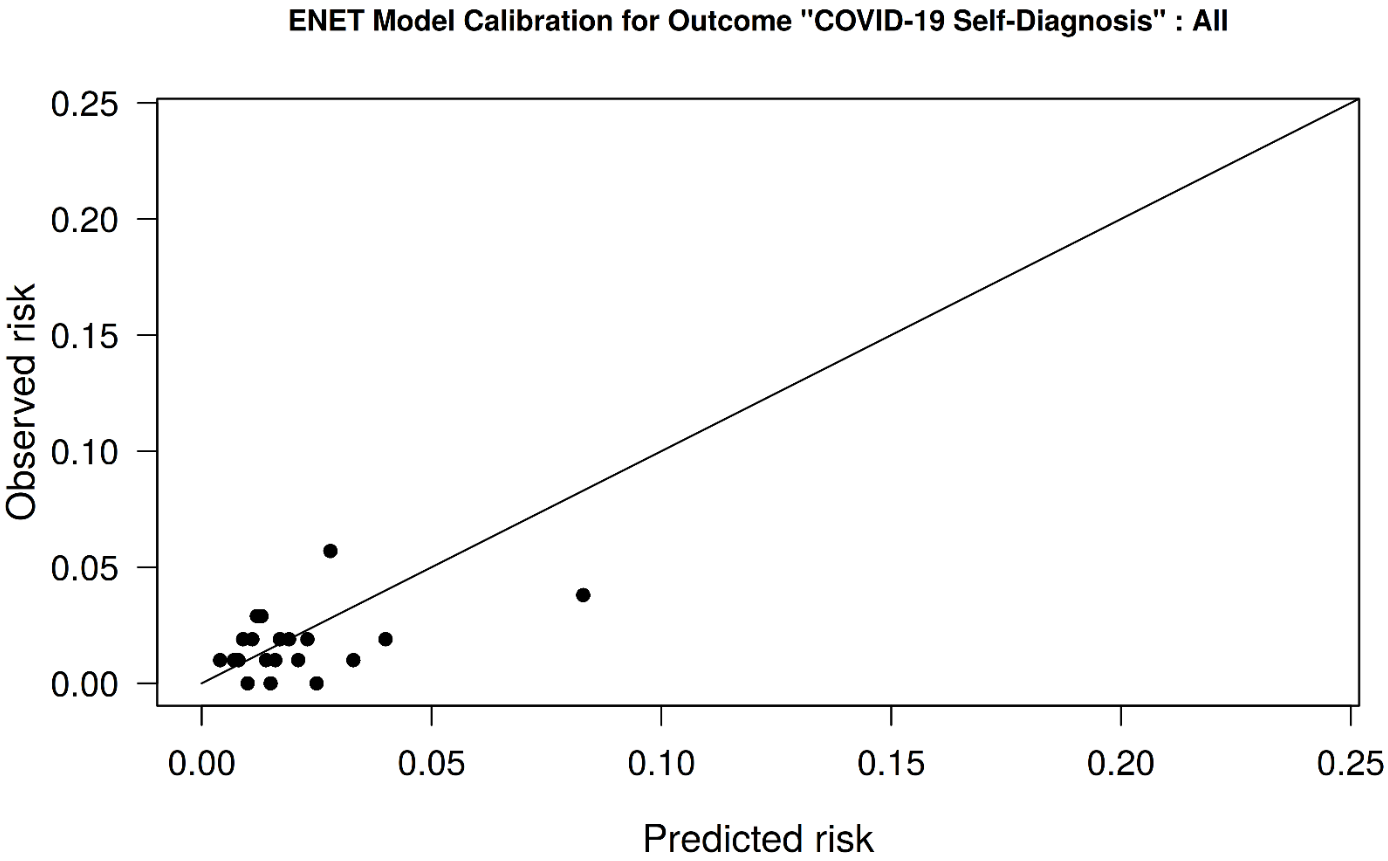

Subjects were split into 20 groups based on their predicted risk, and the observed proportion of COVID-19 tested subject in those groups was plotted against the expected proportion as per the model. The model used for this plot was from the first random 70/30 train-test split out of 100 total.

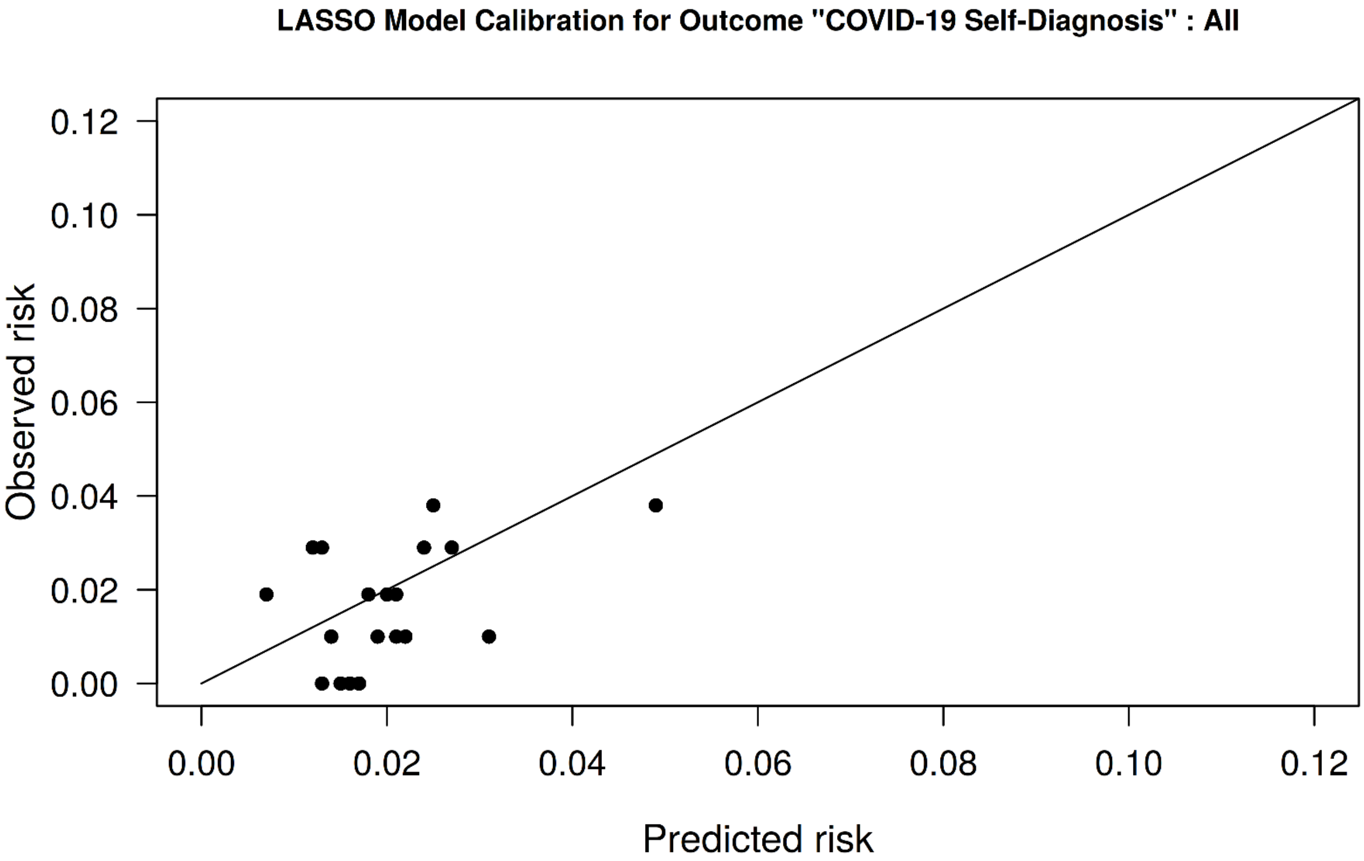

Subjects were split into 20 groups based on their predicted risk, and the observed proportion of COVID-19 tested subject in those groups was plotted against the expected proportion as per the model. The model used for this plot was from the first random 70/30 train-test split out of 100 total.

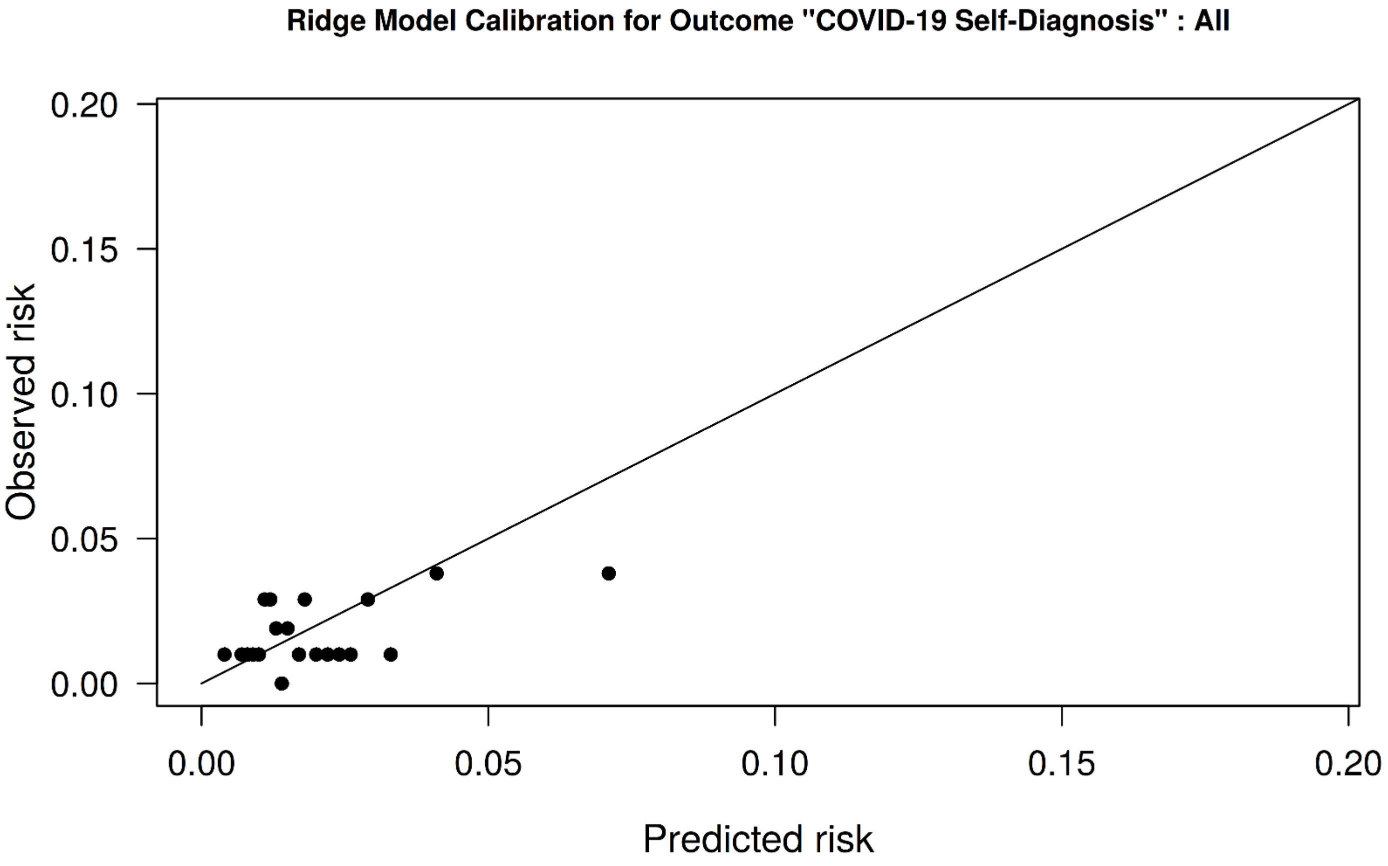

Subjects were split into 20 groups based on their predicted risk, and the observed proportion of COVID-19 tested subject in those groups was plotted against the expected proportion as per the model. The model used for this plot was from the first random 70/30 train-test split out of 100 total.
