# Supplementary material for: Assessing the added value of linking electronic health records to improve the prediction of self-reported COVID-19 testing and diagnosis
Source: PLoS One. 2022 Jul 25;17(7):e0269017. doi: 10.1371/journal.pone.0269017 (PMC9312965; doi:10.1371/journal.pone.0269017)
Supplement: Attachment — Submitted filename: Response_to_reviewers.pdf [file pone.0269017.s017.pdf]

Response to Reviewer Comments

Title: *Assessing the added value of linking Electronic Health Records to improve the prediction of self-reported COVID-19 testing and diagnosis*

Authors: Clark-Boucher, D; Boss, J; Salvatore, M; Smith, JA; Fritsche, LG; Mukherjee, B

Ref: PONE-D-21-35566

We would like to thank the editorial board and the reviewers for their constructive feedback on our previous submission and giving us an opportunity to address the concerns through a revision. Following are itemized responses to the reviewer comments. The original comments are in ***italics***, our responses are in **blue** and the resulting manuscript additions are quoted in **green**. We hope you find the revised manuscript suitable for publication in PLOS One.

Reviewer 1

1. To make the introduction more focused, it may be helpful to clarify that the focus of the study is the prediction of being tested for COVID-19 and being diagnosed with it. At the moment, there is lack of clarity what outcomes are being investigated.

We thank the reviewer for this comment and agree it is important to be clear here. We have edited the introduction section to emphasize that our focus is on prediction of two outcomes (a) being tested for COVID-19 and (b) being diagnosed with the disease. The final paragraph of the Introduction (p. 4) is pasted below. The focus on COVID-19 testing and diagnosis is mentioned in both lines 4 and 7.

"Given the differences between survey data and EHRs, it is natural to question their relative utility in model development for COVID-19-related outcomes of testing and test positivity. However, to our knowledge, a direct comparison of EHR- and survey-derived variables has not been explored on the same population for prediction of these COVID-19-related outcomes. In this study, we explicitly consider whether the addition of EHR variables to survey data can improve prediction models for COVID-19 testing and diagnosis. We combine data from the Michigan Medicine COVID-19 Survey [11] with EHR-derived comorbidities and sociodemographic variables; then, using both pools of variables, we construct prediction models for two outcomes: (1) whether survey respondents have been tested for COVID-19 and (2) whether they have been diagnosed with COVID-19 by either a physician or test, comparing model performance using AUC."

2. Some description of the survey and what questions it entailed, in the methods section would be helpful. There are a lot of references made to it (which is understandable as this is the primary source of data) but not description of it presented. This makes it difficult to evaluate source of data.

We thank the reviewer for this comment, and we agree: It is important for the paper to expand upon the survey questions. We have updated the manuscript in two places: first, in the Data description of the Methods section, and second, in the Outcomes and Variables description. Note that, since there were a diversity of questions, on many topics and in several formats, it would not be feasible to describe the survey in much more detail than this in the main text. As originally submitted, the manuscript's supplement included a list of all variables considered, but it will now also include a link to the survey questions, which were published previously.

The Data section (p. 5) now explains: "Respondents were asked, among other topics, about their living situation, professional work, socioeconomic status, physical health, mental health, drug use, medication use, changes in behavior due to the pandemic, perceptions of pandemic-related restrictions, and efforts to avoid contracting COVID-19. Most questions were multiple choice, though some involved a typed-in numerical response (e.g., height and weight), and many questions were either binary or used a Likert scale. A full version of the survey with branching logic was published by Wu and Hornsby et al. 2020 and is available online via the following URL: https://doi.org/10.1371/journal.pone.0246447.s004 [11]."

The Outcomes and Variables section (pp. 6-7) explains: "We constructed prediction models for two outcomes of interest: first, whether an individual was tested for COVID-19, and second, whether they were diagnosed as COVID-19 positive (by either a test or a physician) or not. Both outcomes were determined by the first two questions of the survey, which asked, (1) "Were you diagnosed with COVID-19?" and (2) "Were you tested for COVID-19 at any point in time? If so, where?" Whether each respondent was diagnosed or tested only once or multiple times was not recorded, nor was information on the specific type of test."

3. Could the authors explain the reasons for excluding variables with missing data rather than imputing the missingness? It seems a wasteful approach that is likely to lead to biased estimates.

Thank you for this suggestion. We agree that imputation would help to make our results more rigorous, and we have decided to update our procedure to incorporate multiply imputed datasets as suggested. As described in the methods section, we used multiple imputation with M = 30, and combined results across datasets using Rubin's rules. Our updated model-fitting procedure which incorporates the multiply imputed datasets is described in response to Reviewer 1, Comment 6.

The Methods section of the manuscript (p. 7) now explains: "Missing data were handled with multiple imputation [26]. Under a missing at random assumption, we used predictive mean matching to create 30 multiply imputed datasets. In accordance with imputation guidelines [27,28], the number 30 was chosen to roughly coincide with proportion of incomplete cases in the full dataset—in our case, 29.0%."

The results of our single-predictor and multi-predictor models are included in the following table and figure. We updated the text of the Results section to reflect these changes (pp.12-13).

Fig 4. Firth-corrected Odds Ratios for Survey-Reported COVID-19 Outcomes, Adjusted for Covariates

All odds ratios used Firth correction, were adjusted for age, sex, race/ethnicity, education, and essential worker status, and were combined across 30 multiply imputed datasets using Rubin's Rules. Significance was determined using $\alpha = 0.05$ for covariates and $\alpha = 0.05/184 \approx 2.72 \times 10^{-4}$ for predictors. For brevity, predictors are included in the figure only if they are statistically significant for at least one outcome.

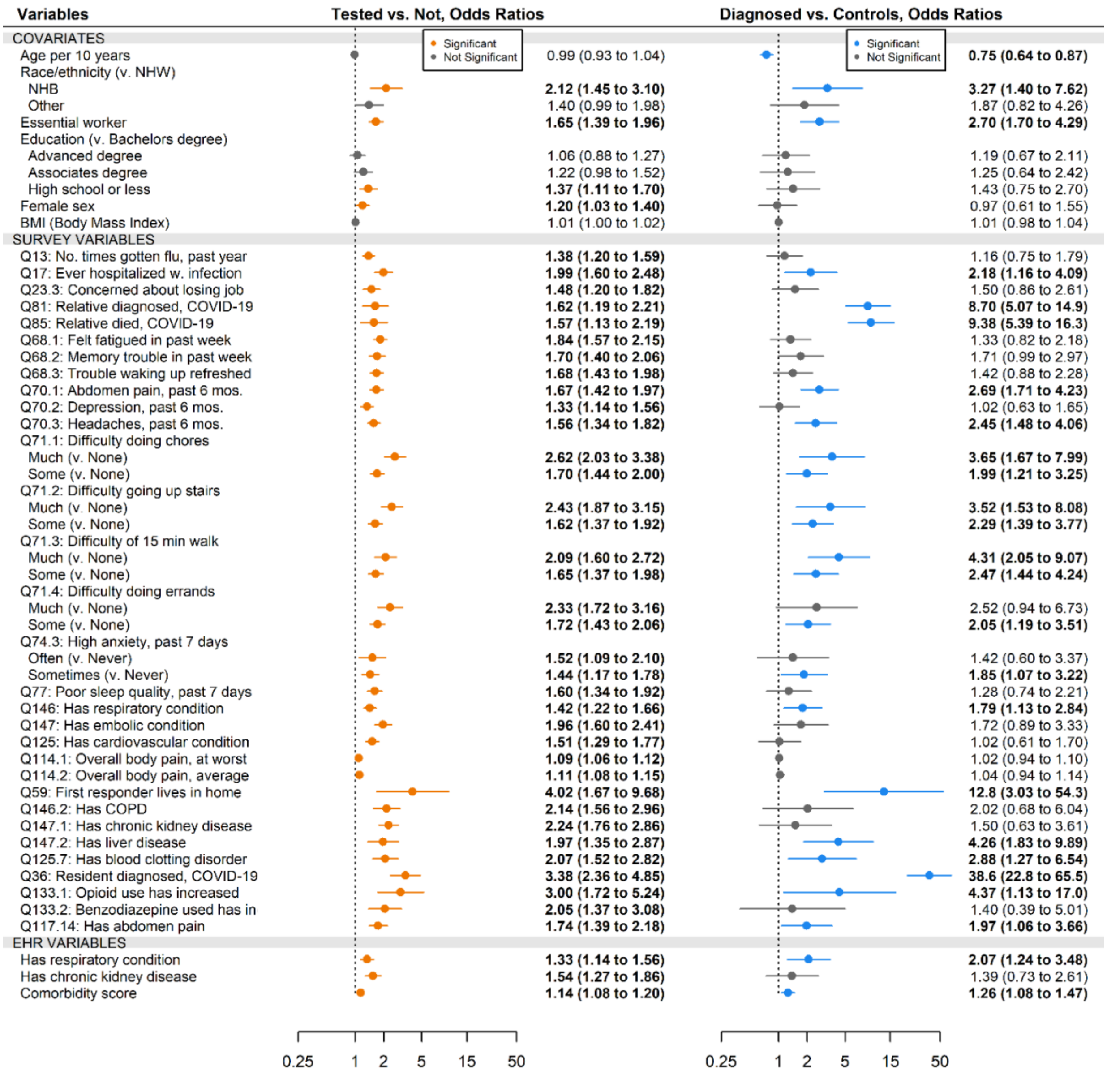

Table 2. Area under the Curve (AUC) and the corresponding 95% CI for the two COVID-19 related outcome prediction Models.

Mean AUC (95% Empirical CI)*					
Outcome Variable	Model Type	Covariates Only	Covariates + EHR Variables	Covariates + Survey Variables	All Variables
Tested for COVID-19	Lasso	0.582<br>(0.552, 0.609)	0.593<br>(0.569, 0.617)	0.646<br>(0.621, 0.676)	0.645<br>(0.619, 0.674)
	Ridge Regression	0.582<br>(0.552, 0.609)	0.597<br>(0.57, 0.623)	0.641<br>(0.618, 0.67)	0.639<br>(0.616, 0.668)
	Elastic Net	0.582<br>(0.552, 0.609)	0.595<br>(0.569, 0.62)	0.649<br>(0.624, 0.678)	0.648<br>(0.624, 0.676)
Diagnosed with COVID-19	Lasso	0.694<br>(0.599, 0.774)	0.694<br>(0.599, 0.774)	0.798<br>(0.718, 0.885)	0.798<br>(0.718, 0.885)
	Ridge Regression	0.694<br>(0.599, 0.774)	0.713<br>(0.615, 0.793)	0.812<br>(0.741, 0.885)	0.821<br>(0.743, 0.887)
	Elastic net	0.694<br>(0.599, 0.774)	0.709<br>(0.612, 0.788)	0.802<br>(0.728, 0.878)	0.804<br>(0.724, 0.88)

*Mean AUC reflects the average of 100 random training test/splits, with a CI representing the 2.5th and 97.5th percentiles, respectively. Each the 100 AUCs was the result of pooling of 30 imputed datasets. The tested for COVID-19 outcome compares the tested population (1) to those not tested (0). The diagnosed with COVID-19 outcome compares those diagnosed with COVID-19 by a physician or test (1) to those not diagnosed, not tested, and not self-diagnosed (0). Data from Michigan Medicine COVID-19 Survey and Michigan Genomics Initiative. Sample size: n = 7,054 for testing outcome models, n = 6,159 for diagnosis outcome models.

4. From the description of the statistical approaches, it would appear that the authors used the same variables as covariates and predictors. If this is so, please clarify how it may be possible for the same variables to be both predictors and covariates?

We thank the reviewer for this point and apologize for the confusion. We have chosen to include age, sex, BMI, essential worker status, and education level as covariates because they are basic characteristics which have already been established as predictors of our outcomes of interest. We adjust for these factors in the single-predictor models and the multi-predictor models because we are only interested in associations and predictive performance after conditioning on them. These covariates also function as a base set of variables to which we can compare the survey-variable and EHR-variable models. We agree that the wording in the manuscript is confusing, so we have eliminated the phrasing "established predictors" and instead refer to this set of variables only as "covariates."

The Methods section (p. 7) now states: We also considered the six variables age, sex, race/ethnicity, body mass index (BMI), education, and essential worker status as covariates, all of which have been shown to be associated with COVID-19 testing, diagnosis, or severity. All prediction models contain this base set of covariates. All selections of additional predictors are carried out conditional on the covariates.

5. For each penalised regressions, please describe how the shrinkage penalty was selected and present these penalties for each approach.

We apologize for this omission and agree that it is important to include these details in the methods section. The Statistical Analysis section (p.8) now clarifies, "The tuning parameters λ_1 and λ_2 were selected by five-fold cross-validation."

Below, we present the λ and α selected for the first ten data splits of the procedure, each averaged over all 30 imputed datasets. Note that $\lambda_1 = \alpha \times \lambda$ and $\lambda_2 = (1 - \alpha)/2 \times \lambda$. To reduce the number of tables and figures in this document, we show only the results of the elastic net models.

Table R1.1. Mean Selected ENET Penalty. Outcome: COVID-19 Testing. Variables: Covariates + EHR

Random Split	Lambda	Alpha
1	0.012	0.247
2	0.013	0.267
3	0.017	0.100
4	0.019	0.213
5	0.022	0.107
6	0.0122	0.273
7	0.018	0.120
8	0.020	0.120
9	0.021	0.120
10	0.012	0.407

Lambda and alpha shown are the mean across 30 models fit on multiply imputed datasets. Lambda and alpha were selected by five-fold cross-validation on the training set of a single 70/30 train/test split.

Table R1.2. Mean Selected ENET Penalty. Outcome: COVID-19 Testing. Variables: Covariates + Survey

Random Split	Lambda	Alpha
1	0.051	0.100
2	0.052	0.107
3	0.054	0.160
4	0.052	0.100
5	0.049	0.160
6	0.048	0.200
7	0.054	0.113
8	0.039	0.300
9	0.045	0.187
10	0.042	0.260

Lambda and alpha shown are the mean across 30 models fit on multiply imputed datasets Lambda and alpha were selected by five-fold cross-validation on the training set of a single 70/30 train/test split.

Table R1.3. Mean Selected ENET Penalty. Outcome: COVID-19 Testing. Variables: All

Random Split	Lambda	Alpha
1	0.057	0.107
2	0.054	0.140
3	0.052	0.180
4	0.055	0.100
5	0.044	0.213
6	0.047	0.187
7	0.056	0.107
8	0.034	0.360
9	0.045	0.227
10	0.039	0.313

Lambda and alpha shown are the mean across 30 models fit on multiply imputed datasets Lambda and alpha were selected by five-fold cross-validation on the training set of a single 70/30 train/test split.

Table R1.4. Mean Selected ENET Penalty. Outcome: COVID-19 Diagnosis. Variables: Covariates + EHR

Random Split	Lambda	Alpha
1	0.007	0.100
2	0.005	0.100
3	0.019	0.133
4	0.005	0.100
5	0.005	0.107
6	0.006	0.100
7	0.005	0.100
8	0.005	0.100
9	0.006	0.407
10	0.025	0.167

Lambda and alpha shown are the mean across 30 models fit on multiply imputed datasets Lambda and alpha were selected by five-fold cross-validation on the training set of a single 70/30 train/test split.

Table. Mean Selected ENET Penalty. Outcome: COVID-19 Diagnosis. Variables: Covariates + Survey

Random Split	Lambda	Alpha
1	0.015	0.893
2	0.017	0.773
3	0.016	0.840
4	0.005	0.700
5	0.005	0.300
6	0.020	0.300
7	0.028	0.260
8	0.037	0.447
9	0.039	0.333
10	0.005	0.900

Lambda and alpha shown are the mean across 30 models fit on multiply imputed datasets Lambda and alpha were selected by five-fold cross-validation on the training set of a single 70/30 train/test split.

Table R1.5. Mean Selected ENET Penalty. Outcome: COVID-19 Diagnosis. Variables: All

Random Split	Lambda	Alpha
1	0.015	0.893
2	0.017	0.773
3	0.016	0.840
4	0.005	0.700
5	0.005	0.300
6	0.020	0.300
7	0.028	0.260
8	0.037	0.447
9	0.039	0.333
10	0.005	0.900

Lambda and alpha shown are the mean across 30 models fit on multiply imputed datasets Lambda and alpha were selected by five-fold cross-validation on the training set of a single 70/30 train/test split.

6. Please note that 70/30 train-test split for internal validation is not a recommended approach as it is likely to lead to biased estimates. A more accurate approach to internal validation is optimism correction. Please see references below.

Thank you for this suggestion. We agree that it is important to perform internal validation in a fair manner. However, we believe there are limitations to and challenges for implementing the optimism correction in this particular situation which have led us to maintain our original approach. First, since we now face the computational burden of working with 30 multiply imputed datasets, the optimism correction approach is not directly applicable. The suggested approach would involve fitting models on datasets which have not only been multiply imputed, but repeatedly bootstrap-sampled. If the number of bootstrap samples were quite large—as it would need to be—the computational burden of the approach would be substantial. This could also be problematic statistically, as the distributional assumptions of optimism correction may not be valid in the case of combining results across bootstrap samples and the dependence induced by multiple imputation. Second, the optimism-corrected estimates of performance may be more encouraging than our current train-test approach. At each stage of the optimism correction procedure, the model is fitted on a bootstrap sample and evaluated on the original sample. Since large numbers of datapoints would be present in both samples, the training and evaluation sets could have high degree of overlap, biasing the results in favor of over-optimism. In contrast, by splitting the data into a train/test split, there are no datapoints which are simultaneously trained and tested on. Doing this 100 times and taking the average, in the opinion of the authors, is a more feasible and conservative approach at evaluating predictive performance. We have made a note of this issue in the Discussion (p. XX): "Though our study evaluated prediction performance of the models by repeated data-splitting of the sample, alternative methods to avoid over-fitting such as the optimism correction [REF] are also logical choices for an honest assessment of prediction. However, data-splitting has the advantage of simplicity, especially in the case of handling multiply imputed datasets, and the procedure should be sufficient for the sake of comparing relative predictive performance across multiple sets of variables on the same sample."

Our manuscript has been updated with additional text on how we conducted the data splitting approach in cohesion with the multiply imputed datasets. The text (p. X) now reads: "We evaluated the models internally using the Area Under the Curve (AUC). Each of the 30 imputed datasets was divided 100 times into a 70-30 train-test split. At each split, the model was fitted on each of the 30 imputed training sets and evaluated on the corresponding test set, and the 30 AUCs were pooled into a single value representing

performance on that split. The mean of the 100 pooled AUCs was taken as the estimated AUC of the model, and the 2.5th and 97.5% percentiles were used to create an empirical 95% confidence interval."

For ease of understanding, a schematic of our model fitting process is included in the following figure.

Fig 3. Internal Validation by Repeated 70:30 Train:Test Data Splitting on Multiply Imputed Datasets.

We valuated each model internally using data-splitting. For each data split, we pooled the results of the 30 imputed datasets into a single AUC using Rubin's Rules. The resulting 100 pooled AUCs were used to compute an empirical mean and confidence interval.

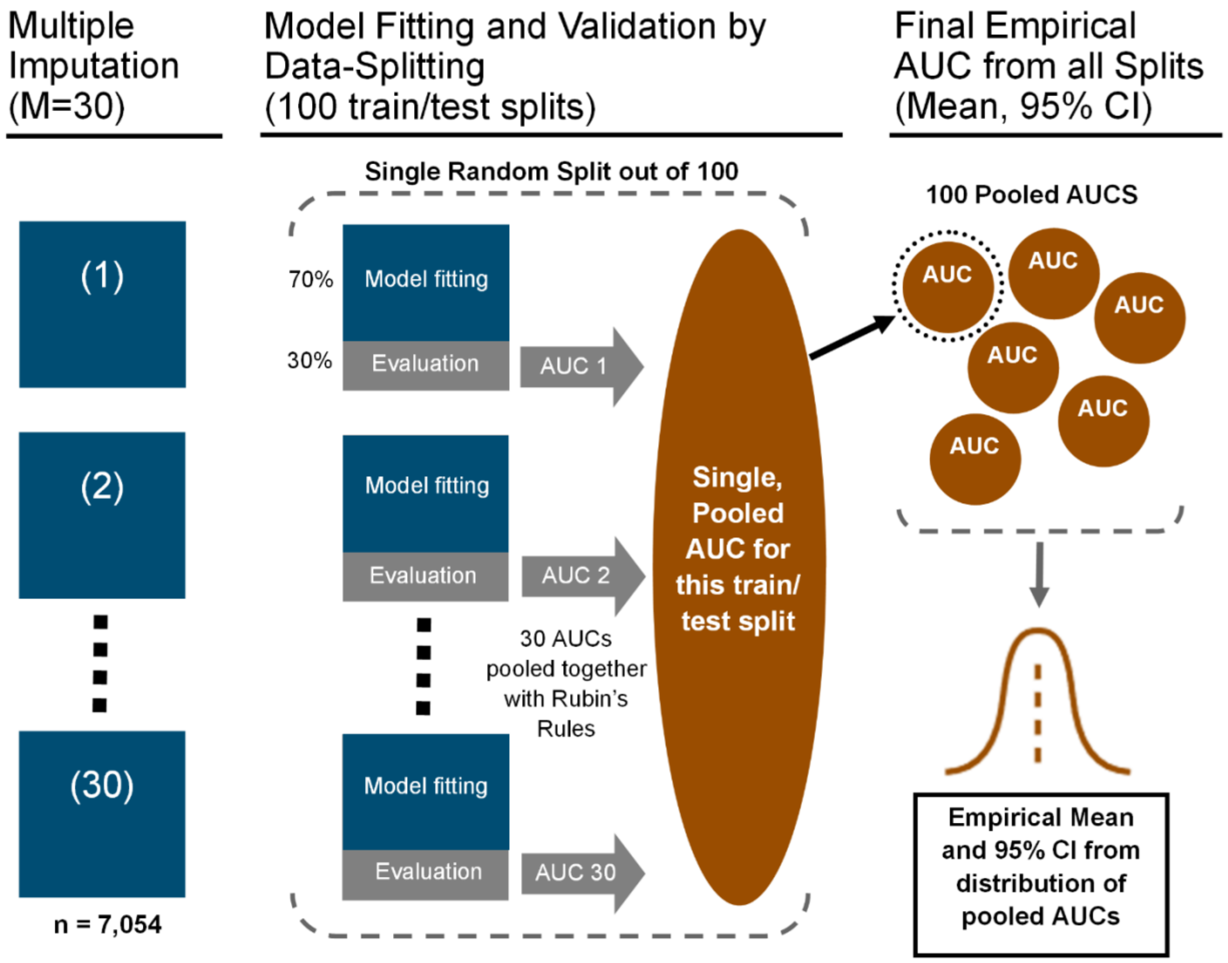

7. According to recent guidelines on how to develop and evaluate predictions models, as part of models' evaluation, it is essential to provide calibration and calibration in the large.

Thank you for your comment. We agree that omitting calibration was an important gap in the paper. We have updated the supplements to include, for each model, (1) a calibration plot for the first random train/test split, and (2) a distribution of Hosmer-Lemeshow Test p-values for each of the train/test splits.

We added the following to Methods section (p. 10): "We assessed the calibration of each model by producing calibration plots corresponding to the first split, and by plotting a distribution of the Hosmer-Lemeshow test p-values of all 100 splits [37]."

In the supplements (S15 File) we discuss the results of these plots: "Models for the outcome *Received a COVID-19 Test* in general appeared to be well-calibrated. The calibration plots from the first train/test split show that the observed risk for groups of respondents was highly similar to the predicted risk, and the Hosmer-Lemeshow p-values from all 100 splits were infrequently statistically significant. Calibration was weaker for the outcome *Diagnosed with COVID-19*, as the relationship between observed risk and predicted risk was less linear, and the distributions of Hosmer-Lemeshow test p-values tended to have spikes below 0.05."

We include one of each of these plots as an example below, while the remainder are available in the supplements (S15 File).

Figure R1.1. ENET Model Calibration for Outcome “Received COVID-19 Test”: Covariates Only
Subjects were split into 20 groups based on their predicted risk, and the observed proportion of COVID-19 tested subject in those groups was plotted against the expected proportion as per the model. The model used for this plot was from the first random 70/30 train-test split out of 100 total.

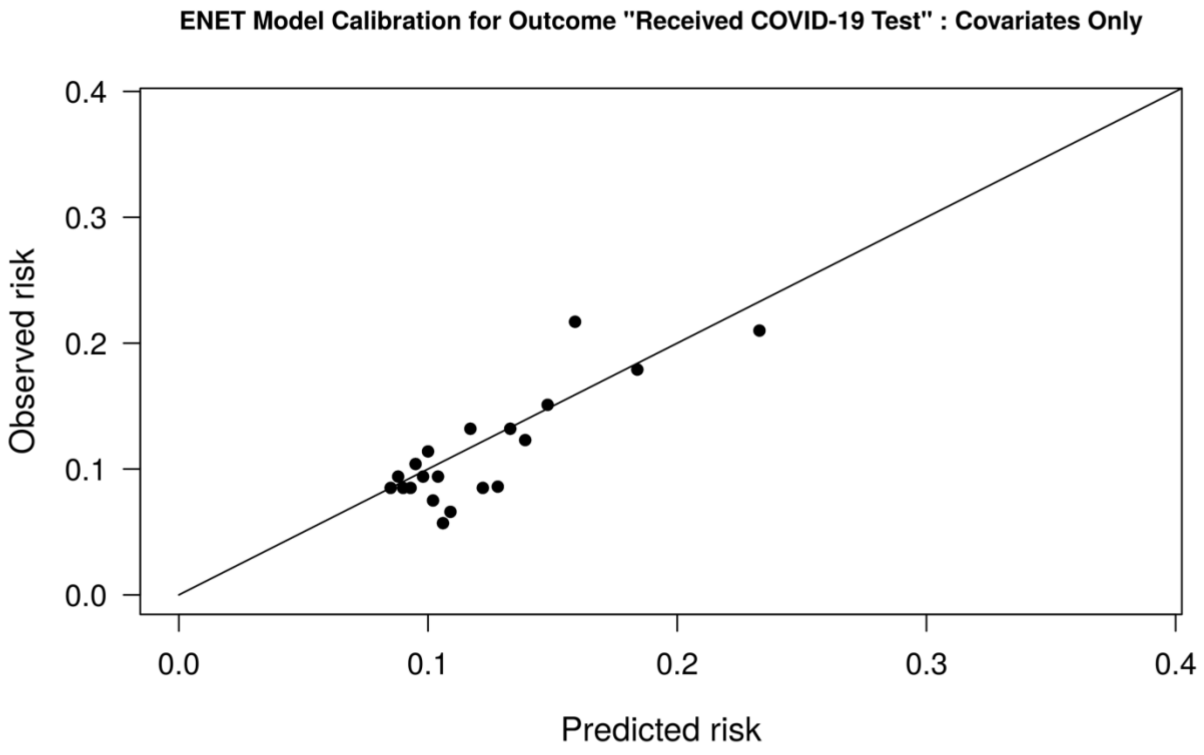

Figure R1.2. ENET Model Hosmer-Lemeshow Tests for Outcome “Received COVID-19 Test”: Covariates Only

We plotted the p-values from conducting a Hosmer-Lemeshow goodness of fit test on all 100 train/test splits of the model evaluation procedure. Models tending to have poor calibration would show large numbers of p-values below the statistical significance threshold of 0.05.

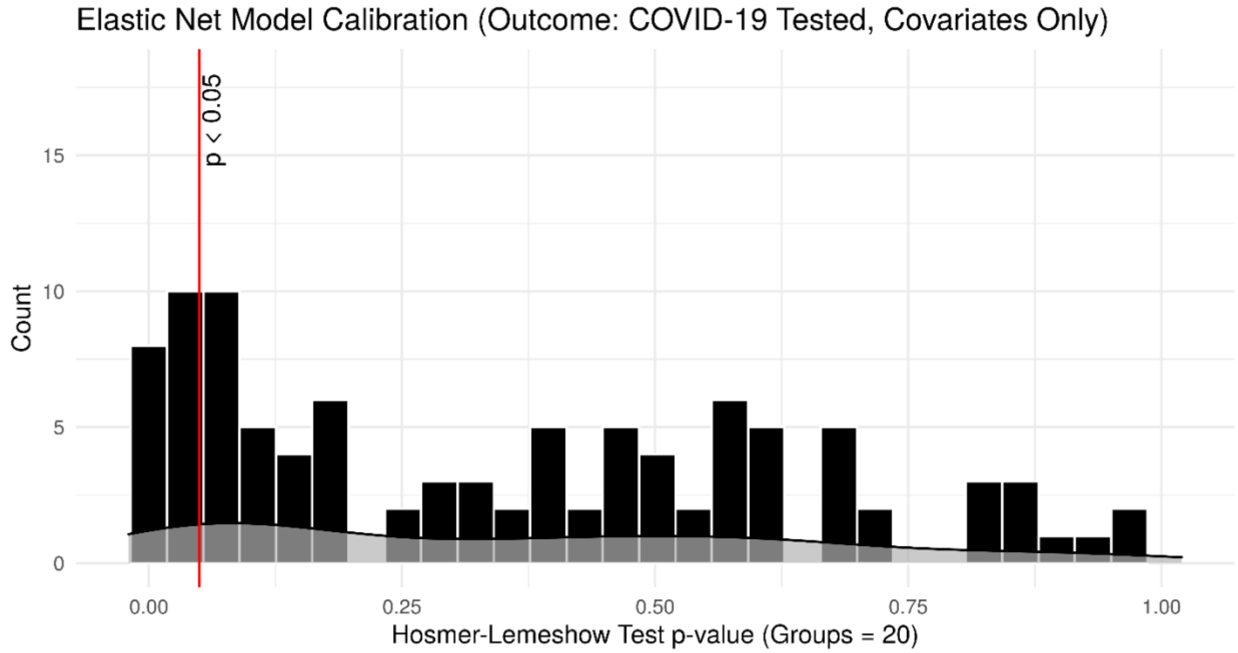

8. Similarly, according to the guidelines, it is essential to provide sample calculation for prediction models, which was not done here.

We thank the reviewer for raising this important point. The resources provided by the reviewer were helpful in reviewing this topic. However, there do not appear to be clear guidelines in the literature that would apply to our specific dataset. Riley et al. 2020 offers a formula for sample size calculation, but it applies only to datasets with 30 features or fewer. In general, after reviewing the literature, there appears not to be a gold standard for sample size calculation when the number of features is large. However, van Smeden et al. 2016 show that penalization methods, such as Firth correction, can improve the separability issues that occur when the number of events is small relative to the number of features. All regression models presented in our manuscript are penalized by either Firth correction, the ridge penalty, or the lasso penalty, all of which perform some degree of shrinkage on the model coefficients. The authors acknowledge that, given the small number of COVID-19 diagnosed cases in the dataset and the large number of features, our sample size may be limited for producing an implementable model for predicting COVID-19-related outcomes. However, to do so is not the major goal of the paper. Rather, in comparing variables from surveys and electronic health records, we effectively *condition* on sample size. Our project asks the question, "Given a particular sample, will the addition of EHR variables improve prediction on the same individuals?" If the sample size was ultra large (millions of records) we may not be able to distinguish the relative contribution of the two sets of predictors but we cannot comment broadly or more generally based on a single dataset anyway unless extensive simulation studies are carried out for various effect sizes and sample sizes. In response to your comment, we have added a note in the discussion to address the small number of events in our sample relative to the number of features.

References:
van Smeden, Maarten et al. "No rationale for 1 variable per 10 events criterion for binary logistic regression analysis." BMC medical research methodology vol. 16,1 163. 24 Nov. 2016, doi:10.1186/s12874-016-0267-3

Riley, R.D., et al., Calculating the sample size required for developing a clinical prediction model. Bmj, 2020. 368: p. m441.

The limitations portion of the Discussion (p. 22)now includes the sentence: “Third, the small number of COVID-19 diagnosed cases in the data (78), relative to the number of features explored (up to 164), could have led to instability in the estimated model coefficients or performance. However, these concerns are lessened, though not eliminated entirely, by the fact that we applied shrinkage to the models via penalization. Moreover, the relative comparison between EHR- and survey-derived variables for prediction can be thought of as conditioning on a given sample, so the relative comparison is still of value despite this limitation of the data. A proper quantification that is broadly generalizable would require simulation studies with various effect sizes and sample sizes that is beyond the scope of the current work.”

9. From the description of the statistical approaches, it appears that the Bonferroni adjustments were applied to all variables. Does it include those variables that were included in the penalised models? If so, this is incorrect as penalised models already correct for collaterality by utilising a penalty.

We thank the reviewer for this astute comment and apologize for the confusion. Bonferroni correction was applied to the p-values produced in the single-predictor screening portion of the analysis but was not used in the penalized multi-predictor prediction models. The final goal was prediction with a joint model, and we know that statistically insignificant predictors may add to the overall prediction. The multivariate model estimates were simply solutions to the penalized objective function after conditioning on the covariates. Although the single-predictor models employ Firth correction—technically a penalty—it is nevertheless important to adjust for multiple testing when presenting p-values and confidence intervals to multiple marginal association hypotheses, as we do in the forest plot, hence the need for Bonferroni correction.

References:

• Altman, D.G., et al., Prognosis and prognostic research: validating a prognostic model. Bmj, 2009. 338: p. b605.

• Steyerberg, E., Clinical Prediction Models. A practical approach to development, validation, and updating. Second Edition ed. 2019: Springer Nature Switzerland.

• Riley, R.D., et al., Calculating the sample size required for developing a clinical prediction model. Bmj, 2020. 368: p. m441.

• Collins, G.S., et al., Transparent reporting of a multivariable prediction model for individual prognosis or diagnosis (TRIPOD): the TRIPOD statement. Bjog, 2015. 122(3): p. 434-43.

• Harrell, F.E., Jr., K.L. Lee, and D.B. Mark, Multivariable prognostic models: issues in developing models, evaluating assumptions and adequacy, and measuring and reducing errors. Stat Med, 1996. 15(4): p. 361-87.

• Hui Zou, T.H., Regularization and variable selection via the elastic net. Journal of the Royal Statistical Society, 2005(67): p. 301-320.

• Riley, R.D., et al., External validation of clinical prediction models using big datasets from e-health records or IPD meta-analysis: opportunities and challenges. Bmj, 2016. 353: p. i3140.

• Moons, K.G., et al., Using the outcome for imputation of missing predictor values was preferred. J Clin Epidemiol, 2006. 59(10): p. 1092-101.

Reviewer #2

Excellent MS. Sound study design and methodology. Clear discussion of findings and fair assessment of weaknesses, particularly with respect to limited generalizability of findings due to low survey response rate (16.7%).

We thank the reviewer for this comment. Positive feedback is greatly appreciated.

Reviewer #3

Research idea: The research idea is novel and relevant to current pandemic circumstances. The authors have been able to use data linkage to explore gaps in assessing risk of SARS-Cov-2 diagnosis and where it can be improved upon.

Problem Statement: The problem statement is well defined in the introduction. The research gap has been well articulated.

Case definition: The case definition utilised standardised measures (i.e RT-PCR for case diagnosis).

Outcomes definition: The outcomes are also defined.

Feature reduction techniques & Bias control: Efforts to control for multicollinearity using Ridge regression is applauded. The authors also utilised Elastic Net to combine regularisation of Ridge & lasso regression. Other feature reduction techniques are noted. There has been significant efforts to control for bias in this study.

Discussion: In the discussion, the authors make a good case for how how covid outcomes from EHR data should be interpreted. The gaps not addressed by this research has been acknowledged.

Recommendations: Recommendations for collection of more data points in order to accurately assess Covid risk is in order.

Review summary: The authors utilised technically rigorous methods to address a time-relevant research question. Kudos.

Thank you for your comments. We appreciate the positive feedback.

<u>Reviewer 4</u>

Minor comments

1. I suggest that the authors state the reason for choosing the Michigan Genomics Initiative over other initiatives for this particular project.

Thank you for raising this point. Of the original 8,422 respondents to the survey conducted by Wu and Hornsby et al., data were available on 7,054 participants of the Michigan Genomics Initiative. Our group did not have approval to access the individual level data of participants of the other repositories included in the survey. We have updated the Data portion of the Methods section (p. 5) with the line, "Respondents to the survey who had participated in other repositories besides MGI were not considered, as we did not have approval to access the individual level data of these participants."

2. I suggest that the data flow for survey-based and EHR-based analysis (fig 1) includes the dates in which the survey and the data pull were performed.

Thank you for your comment. We agree this detail is important and we have added the dates to the figure (Figure 1), shown below.

Figure 1. Processing of COVID-19 Survey Data and Michigan Genomics Initiative EHRs.

For the survey-based analysis, respondents from the COVID-19 survey were limited to those with relevant EHR from MGI. The desired EHR variables were then merged with the survey data for those patients.

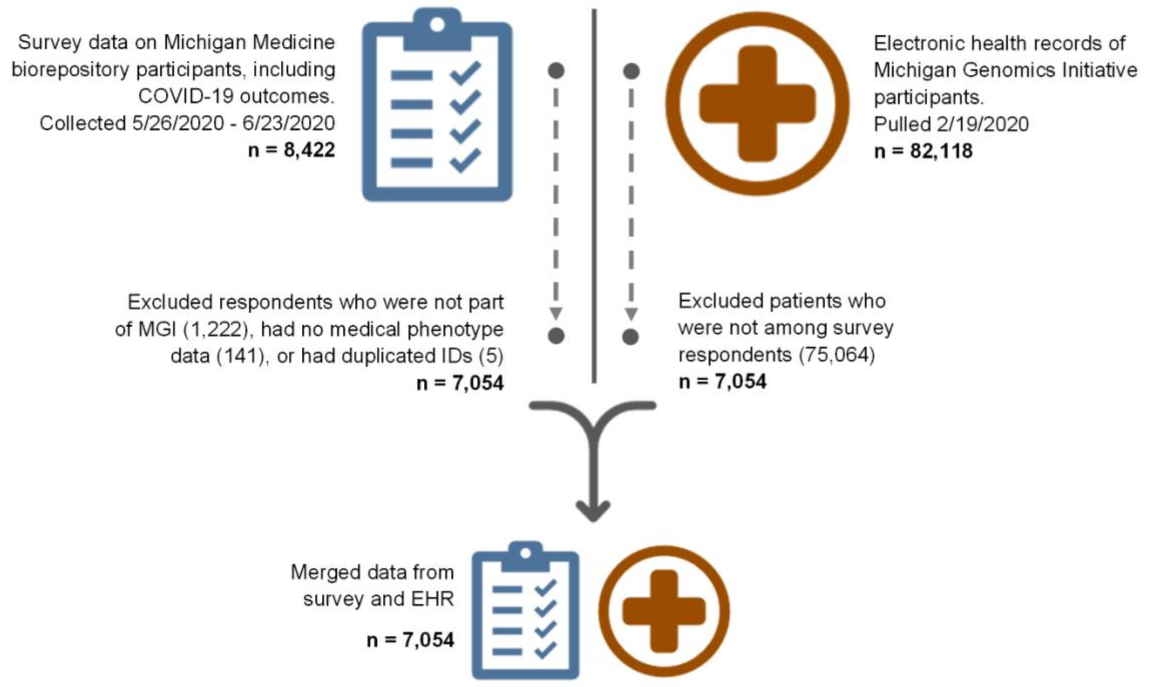

S14 Figure 1. Data Processing for Michigan Medicine EHR Case Control Analyses

For the EHR-only analysis, patients tested for COVID-19 or diagnosed with COVID-19 were extracted from Michigan Medicine records to form the cases, and 30,000 randomly chosen, untested patients were incorporated as controls.

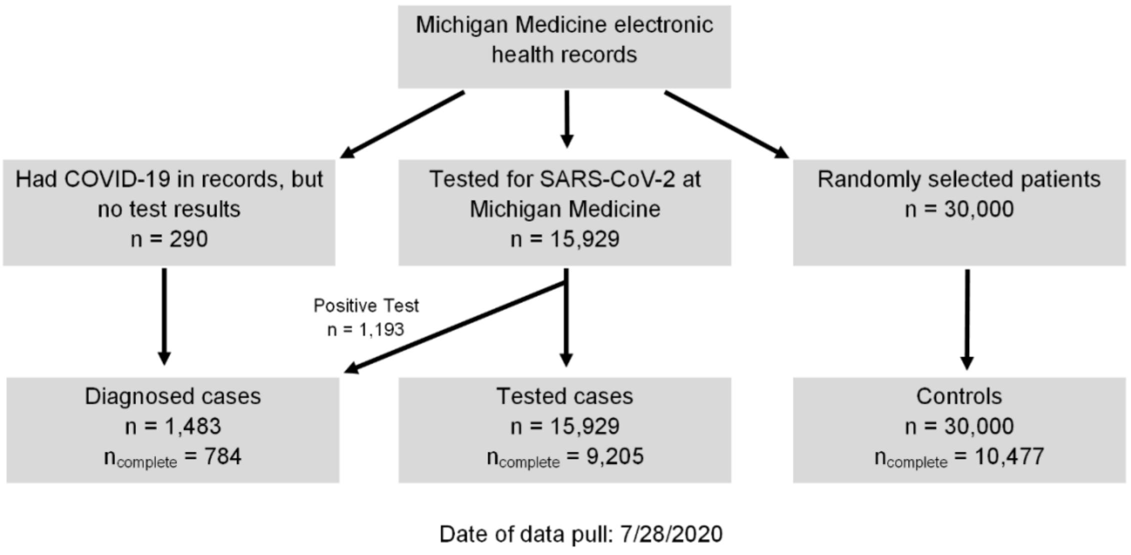

3. The dates of the data survey and the EHR analyses are not overlapping. It would be important to explain why and if that can lead to any different results.

Thank you for raising this important point. For the main analysis, linking survey data with EHRs, the EHRs we used had been pulled on February 19, 2020. This was the most recent data pull prior to the start of the pandemic in Michigan, which is a preferable choice because we would like the EHR-derived variables to be strictly pre-pandemic. This way, medical conditions which could have been caused by COVID-19 infection would not be used as predictors of COVID-19-related outcomes. We explain this decision in the manuscript (p. X) when we write "This date was chosen to precede the initial COVID-19 outbreak in Michigan, which began in March of 2020 [2], so that the data would include medical conditions that existed strictly before COVID-19 infection as opposed to those that may have been due to infection."

If the question is aimed at the EHR-only analysis, which used EHRs from Michigan Medicine, the cases include those tested for COVID-19 or diagnosed with COVID-19 per Michigan Medicine records between March 10th and June 30th of 2020. These dates coincide nicely with the survey, which was active from May 26 to June 23rd of 2020, so the EHR-only and survey-based analyses do assess COVID-19 tests and diagnoses which occurred in a similar time span—the first four months of the pandemic in Michigan. In response to your suggestion, we updated the manuscript to explain: "These dates were chosen to align approximately with the timeline of the COVID-19 Survey, so that both analyses would capture COVID-19-related outcomes which had occurred in the same window: namely, the first four months of the pandemic in Michigan." We would note, though, that the EHR-only and survey-based analyses are not directly comparable, since they represent different populations, and we have made the choice to move the EHR-only analysis to the supplement in response to the comments of another reviewer.

4. In the "Outcomes and variables" of the methods section, the definition of "tested for COVID" and "diagnosed with COVID" should be clearly stated and the questions used in the survey transcribed. It is not clear if the test performed was a PCR or an antigen test. In addition, it is not stated if any given person was tested more than once and how this data is handled.

We thank the reviewer for this comment, which raises an important point. We have made the following change to the "Outcomes and Variables" opening paragraph. Details about the specific coding of these variables are included shortly after, in the "Statistical Analysis" section (p. X), because they are relevant to the logistic regression.

"We constructed prediction models for two outcomes of interest: first, whether an individual was tested for COVID-19, and second, whether they were diagnosed as COVID-19 positive (by either a test or a physician) or not. Both outcomes were determined with the first two questions of the survey, which asked (1) "Were you diagnosed with COVID-19?" and (2) "Were you tested for COVID-19 at any point in time? If so, where?" Whether each respondent was diagnosed or tested only once or multiple times was not recorded, nor was information about the specific type of test it was."

5. The participation rate in the survey is extremely low, which can lead to a significant (2 level) selection bias. It would be interesting to compare these individuals with the ones who have not participated in the original Michigan Medicine Repository and not with the ones that, having participated in this initiative, have not answered this particular survey.

Thank you for this important point. There are several stages of inclusion in the analytic sample. Comparing with Michigan Medicine, we see the survey population was older, more females and had more complete data on race/ethnicity. This leads to issues with generalizability of the prediction models to MM more broadly or to the population of the state of Michigan.

We have added to the Discussion (p. X) to address this concern: "Advanced tools such as inverse probability weighting, which can help account for some of these biases, were beyond the scope of the current work, as our comparison between survey- and EHR-derived variables is still fair on a fixed pool of respondents."

Table R4.1. Demographic Statistics of COVID-19 Survey Respondents, Michigan Genomics Initiative Participants, and Michigan Medicine Repository

	COVID-19 Survey n = 7,054		Michigan Genomics Initiative n = 82,372		Michigan Medicine n = 3,953,712	
	Number	Percent	Number	Percent	Number	Percent
**Age**						
18-30	379	5.37	8069	9.80	655185	16.57
31-40	689	9.77	8980	10.90	514851	13.02
41-50	907	12.86	11855	14.39	571074	14.44
51-60	1530	21.69	16699	20.27	635522	16.07
61-70	2066	29.29	19523	23.70	587345	14.86
71-80	1284	18.20	12880	15.64	393714	9.96
81+	199	2.83	4365	5.30	596021	15.08
**Sex**						
Male	2831	40.13	37891	45.00	1800540	45.54
Female	4223	59.87	44479	54.00	2102545	53.18
Other	0	0	0	0.00	6406	0.16
Unknown	0	0	2	0.002	44222	1.12
**Race/Ethnicity**						
NHAA	158	2.24	4994	6.06	106744	2.70
NHW	6545	92.78	68341	82.97	950123	24.03
Unknown	90	1.28	3590	4.36	2750156	69.56
Other	261	3.70	5447	6.61	146695	3.71

Michigan Medicine records include patients who received treatment at any point from 01/01/2000 to 07/27/2020 and were over 18 years of age. Note that many self-reported Caucasians did not report an ethnicity, only a race, and therefore the number of unknowns in the Michigan Medicine Race/Ethnicity variable is large. Acronyms: NHAA, Non-Hispanic African American, NHW, Non-Hispanic White

Reviewer 5

Major Comments

1. I feel that some context is missing from the introduction. While the predictors contained in various data sources are discussed at length, and this is certainly an important driver of predictive model performance, a quantitative overview of existing predictive models' is missing. A brief brief comparison of models trained on EHR and survey data would help motivate your research question.

Thank you for this comment. We agree that some background on predictive models was lacking from the introduction section. The section has been modified so that paragraphs 3 and 4 consecutively cover EHR-based and survey-based analysis, and so that both paragraphs include quantitative details on existing models.

The text of the Introduction (pp. 3-4) now reads:

"Predictive models of COVID-19-related outcomes have tended to rely on either electronic health records (EHRs) or survey data. In optimal cases, EHRs can paint a detailed picture of a patient's medical history, providing demographic information, anthropometrics, and longitudinal disease and procedure codes. Vaid et al. used EHRs to predict mortality and critical events among COVID-19 patients with Areas Under the Receiver Operating Characteristic curve (AUCs) of 0.79 to 0.89 [14]. Feng et al. used EHRs, specifically lab results, to predict suspected COVID-19 pneumonia with an AUC of 0.94 among on hospital admittees [15]. Using EHR-derived comorbidities and social variables, Hippisley-Cox et al. built a model explaining 74.1% of the variation in time to death among adults hospitalized for COVID-19 post COVID-19 vaccination [16].

Though EHRs have their advantages—such as costs, detail, and sample size—they also have limitations. Indeed, what EHRs offer in depth, they sometimes lack in depth, as non-medical data are typically either limited or unavailable [17,18]. Survey data, in contrast, can be more specific to the topic of interest. Recent studies have used email correspondence, mobile applications, and other digital survey tools to study COVID-19 status in relation to exposure, occupation, social habits, and many other non-medical variables [10,11,19,20]. With survey data on 3,829 mobile-phone application users, Allen et al. obtained an AUC of 0.79 for predicting positive COVID-19 test results [10]. Surveys may also be able to identify COVID-19 cases with greater success, as EHRs tend to be system- or hospital-specific and may miss out on COVID-19 cases that were diagnosed or treated at offsite locations."

2. Am I understanding correctly that the single-predictor models were trained on the entire survey data, and that their test results were used for variable selection in the multi-parameter models trained on subsets of the same data? If this is the case, then observations in the test sets were used to select the predictors present in the training sets. The empirical mean AUCs are likely over-optimistic for these models. The initial filtering step should be performed, wherever the survey predictors are used, as part of the model fitting procedure to avoid this issue. See Ambroise and McLachlan [2002] for a discussion on this topic. Alternatively, you could avoid this feature selection procedure altogether when training the multivariate models by relying solely on penalized logistic regressions models. The single-predictor models could then be reported as a stand-alone analysis of marginal predictor importance (adjusting for established covariates).

We thank the reviewer for raising this point, which was indeed an oversight and an error in our methodology. In response to the comments of another reviewer, we have modified our analysis to incorporate multiple imputation, so the single-predictor models—fit on the whole data—now have no bearing on the variables or observations which are included in the multi-predictor models. These stages of the analysis are now completely independent, so there should no longer be any issues of data leakage. Thank you again for your careful reading and attention to details in catching this mistake.

3. Instead of using the results of a forward selection procedure trained on the full dataset to gain insight on the features selected by the models in the training sets, why not report the proportion of times features were selected when fitting the models to the training sets? This is straightforward to compute using the forward selection, LASSO, and/or elastic-net models.

Thank you for your comment. We agree this would be a superior approach. Further, for a variety of reasons, such as computational burden, we have elected to remove forward selection from the paper altogether. Instead, we now report the variables that were selected at least 80% of the time for the lasso and elastic net models. The number reported is the proportion of times the variable was selected over all 100 train/test splits on all 30 multiply imputed datasets—3,000 models total.

The updated manuscript text (pp. 14-15) reads:

"Lastly, to illustrate some of the variables these models have chosen, we report the variables selected at least 80% of the time from the elastic net models for both outcome variables. Results for the lasso regression models are included in the supplements (S9 Table). Note that, in the present context, it would be inappropriate to provide estimated model coefficients, as they would be adjusted for different sets of selected variables and therefore have different interpretations. For predicting self-reported COVID-19 testing (Table 3), the EHR-variable models chose comorbidity score 99% of the time, a kidney disease indicator variable 98% of the time, and a respiratory disease indicator 94% of the time. In the survey-variable models, several predictors had extremely high selection rates, such as whether a person had ever been hospitalized with a viral infection (Q17, 1.00) and whether a member of their household (besides themselves) had been diagnosed with COVID-19 (Q36, 1.00). The all-variable model results look similar to the survey-variable model results, and no EHR-derived variables were selected more than 80% of the time.

For predicting self-reported COVID-19 diagnoses (Table 4), the most frequent predictor chosen in the EHR-variable models was an indicator for liver disease (0.92), the only predictor to be chosen more than 90% of the time. In the survey-variable models, the most selected variable again was whether a member of the respondent's household had been diagnosed with COVID-19 (Q36, 1.00), though similar variables—such as having had a relative diagnosed with COVID-19 (Q81, 0.82)—were popular as well. The all-variable models produced similar results to the survey-variable models, and again no EHR-derived variables were selected more than 80% of the time. Overall, selection rates tended be lower for the COVID-19 diagnosis outcome than the COVID-19 testing outcome (Table 3), a sign that the former has fewer strong predictors and requires sparser models."

Table R5.1. Lasso Regression Variable Selection in Models of "Test for COVID-19" Outcome

Proportion of Times Selected					
EHR Variables Models		Survey Variable Models		All Variable Models	
Kidney disease	0.95	Q17. Ever Hospitalized with infection	1.00	Q17. Ever Hospitalized with infection	1.00
Comorbidity score	0.92	Q36. Household member diagnosed with COVID-19	1.00	Q36. Household member diagnosed with COVID-19	1.00
		Q68.1 Felt fatigued in past week	1.00	Q68.1 Felt fatigued in past week	1.00
		Q147.1 Kidney disease	0.99	Q147.1 Kidney disease	0.99
		Q70.1 Abdomen pain in past 6 months	0.98	Q70.1 Abdomen pain in past 6 months	0.98
		Q146.2 COPD	0.96	Q146.2 COPD	0.96
		Q70.3 Headaches in past 6 months	0.96	Q70.3 Headaches in past 6 months	0.95
		Q125. Cardiovascular condition	0.95	Q125. Cardiovascular condition	0.94
		Q13. Number of times gotten flu in past year	0.94	Q13. Number of times gotten flu in past year	0.94
		Q125.7 Blood clotting disorder	0.91	Q125.7 Blood clotting disorder	0.9
		Q59.1. Police officer lives in home	0.87	Q59.1. Police officer lives in home	0.84
		Q23.3 Concerned about losing job	0.82		

The value shown is the proportion of times the variable was chosen in 3,000 fitted models, as models were fit on 100 train/test splits of 30 multiply imputed datasets (100x30=3,000). Only variables with a selection rate over 80% are included. Variable descriptions are available in the supplement (S1 Table). The tested for COVID-19 outcome compares the tested population (1) to those not tested (0). All models included the six covariates age, sex, race/ethnicity, body mass index, education level, and essential worker status, which were not selected for or penalized. Data from Michigan Medicine COVID-19 Survey and Michigan Genomics Initiative. Sample size: 7,054

Table R5.2. Elastic Net Regression Variable Selection in Models of "Test for COVID-19" Outcome

Proportion of Times Selected					
EHR Variables Models		Survey Variable Models		All Variable Models	
Comorbidity score*	0.99	Q17. Ever Hospitalized with infection	1.00	Q17. Ever Hospitalized with infection	1.00
Kidney disease*	0.98	Q36. Household member diagnosed with COVID-19	1.00	Q36. Household member diagnosed with COVID-19	1.00
Respiratory disease*	0.94	Q147.1 Kidney disease	1.00	Q147.1 Kidney disease	1.00
Liver disease*	0.91	Q68.1 Felt fatigued in past week	1.00	Q68.1 Felt fatigued in past week	1.00
Former smoker*	0.80	Q70.1 Abdomen pain in past 6 months	1.00	Q70.1 Abdomen pain in past 6 months	1.00
		Q70.3 Headaches in past 6 months	1.00	Q70.3 Headaches in past 6 months	0.99
		Q13. No times gotten flu in past year	0.99	Q13. No times gotten flu in past year	0.99
		Q125. Cardiovascular condition	0.98	Q125. Cardiovascular condition	0.98
		Q146.2 COPD	0.98	Q146.2 COPD	0.97
		Q147. Metabolic Condition	0.96	Q125.7 Blood clotting disorder	0.95
		Q125.7 Has cardiovascular condition	0.95	Q147. Metabolic condition	0.94
		Q59.1 Police officer lives in home	0.95	Q59.1 Police officer lives in home	0.94
		Q23.3 Concerned about losing job	0.95	Q114.1 Overall body pain at worst	0.94
		Q71.1 Some difficult doing chores	0.94	Q23.3 Concerned about losing job	0.93
		Q71.1 Much difficulty doing chores	0.94	Q71.1 Much difficulty doing chores	0.93
		Q114.1 Overall body pain at worst	0.94	Q71.1 Some difficulty doing chores	0.93
		Q133.2 Benzodiazepine use has increased	0.88	Q133.2 Benzodiazepine use has increased	0.87

		Q114.2 Overall body pain on average	0.86	Q68.3 Trouble waking up refreshed	0.85
		Q46. Flu shot in past year	0.86	Q77. Poor sleep quality, past 7 days	0.85
		Q77. Poor sleep quality, past 7 days	0.86	Q114.2 Overall body pain, on average	0.85
		Q68.3 Trouble waking up refreshed	0.85	Q133.1 Opioid use has increased	0.83
		Q133.1 Opioid use has increase	0.85	Q46. Flu shot in past year	0.83
		Q36.1. Lives alone	0.83	Q36.1. Lives alone	0.81
		Q68.2 Memory trouble in past week	0.81		

The value shown is the proportion of times the variable was chosen in 3,000 fitted models, as models were fit on 100 train/test splits of 30 multiply imputed datasets (100x30=3,000). Only variables with a selection rate over 80% are included. Variable descriptions are available in the supplement (S1 Table). The tested for COVID-19 outcome compares the tested population (1) to those not tested (0). All models included the six covariates age, sex, race/ethnicity, body mass index, education level, and essential worker status, which were not selected for or penalized. Data from Michigan Medicine COVID-19 Survey and Michigan Genomics Initiative. Sample size: 7,054

Table R5.3. Lasso Regression Variable Selection in Models of “Diagnosed with COVID-19” Outcome

Proportion of Times Selected					
EHR Variables Models		Survey Variable Models		All Variable Models	
<No Variables>		Q36. Household member diagnosed with COVID-19	1.00	Q36. Household member diagnosed with COVID-19	1.00

The value shown is the proportion of times the variable was chosen in 3,000 fitted models, as models were fit on 1000 train/test splits of 30 multiply imputed datasets (100x30=3,000). Only variables with a selection rate over 80% are included. Variable descriptions are available in the supplement (S1 Table). The diagnosed with COVID-19 outcome compares those diagnosed with COVID-19 by a physician or test (1) to those not diagnosed, not tested, and not self-diagnosed (0). All models included the six covariates age, sex, race/ethnicity, body mass index, education level, and essential worker status, which were not selected for or penalized. Data from Michigan Medicine COVID-19 Survey and Michigan Genomics Initiative. Sample size: 6,159

Table R5.4. Elastic Net Regression Variable Selection in Models of "Diagnosed with COVID-19" Outcome

Proportion of Times Selected					
EHR Variables Models		Survey Variable Models		All Variable Models	
Liver disease	0.92	Q36. Household member diagnosed with COVID-19	1.00	Q36. Household member diagnosed with COVID-19	1.00
Respiratory disease	0.87	Q85. Relative died from COVID-19	0.85	Q85. Relative died from COVID-19	0.82
		Q70.1 Abdomen pain in past 6 months	0.82	Q81. Relative diagnosed with COVID-19	0.81
		Q81. Relative diagnosed with COVID-19	0.82	Q70.1 Abdomen pain in past 6 months	0.80

The value shown is the proportion of times the variable was chosen in 3,000 fitted models, as models were fit on 1000 train/test splits of 30 multiply imputed datasets (100x30=3,000). Only variables with a selection rate over 80% are included. Variable descriptions are available in the supplement (S1 Table). The diagnosed with COVID-19 outcome compares those diagnosed with COVID-19 by a physician or test (1) to those not diagnosed, not tested, and not self-diagnosed (0). All models included the six covariates age, sex, race/ethnicity, body mass index, education level, and essential worker status, which were not selected for or penalized. Data from Michigan Medicine COVID-19 Survey and Michigan Genomics Initiative. Sample size: 6,159

4. Related to the above: Absolute coefficient estimates produced by stepwise selection procedures tend to be upwards biased. Does the Firth-correction counteract this? If so, it might be helpful to state it.

Thank you for this remark. Firth bias correction is largely intended to address separation issues, specifically, when the sample size is small or there are a small number of events of one category. The upward bias of forward selection, however, is due to a variety of factors, so we would not expect that Firth correction counteracts this issue completely. However, there may be some benefit. Note that, for computational reasons, we have chosen to omit forward selection from our multivariable models now that we are doing imputation. Forward selection is time consuming for such a large number of features, and it is less appropriate for variable selection problems than more modern tools such as lasso. For the single-predictor models, where Firth correction is utilized, we can observe the effects of shrinkage explicitly. For instance, the Firth-corrected odds ratio of being an essential worker for the COVID-19 diagnosis outcome is 2.70, adjusting for BMI, age, race/ethnicity, sex, and education level. If we fit the same model without Firth correction, and extract the coefficient of this variable, the result is 2.72, slightly further from the null.

Wang, Xuefeng. “Firth logistic regression for rare variant association tests.” Frontiers in genetics vol. 5 187. 19 Jun. 2014, doi:10.3389/fgene.2014.00187

5. I think that more discussion is needed to motivate the relevance of the EHR-based analysis to your aim of evaluating whether survey data should be augmented with EHR data. As you mention, the underlying populations for these analyses differ, and so the various models' predictive performances can't be compared equitably. The message of the paper might be made clearer by moving this analysis to the supplement, and only briefly mentioning in the main paper that, although these EHR-based predictors don't appreciably improve the performance of models trained on survey data, they still have value in EHR-based case-control studies.

Thank you for your comment. We agree that the EHR-only analysis with Michigan Medicine data is better suited for the supplementary materials. We have modified the Methods portion of the paper to focus only on the survey-based analysis until a section at the very end, which addressed the EHR-only analysis as a supplementary analysis.

"As a supplementary analysis, we explored how these same 15 EHR-derived variables would perform for prediction on the greater set of EHR data that was available. We used Michigan Medicine EHRs to construct cases and controls for both outcome variables. For the COVID-19 tested cases, we retrieved data for all 15,929 patients who had obtained a reverse transcription polymerase chain reaction (RT-PCR) test for SARS-CoV-2 at Michigan Medicine between March 10th and June 30th, 2020. For COVID-19 diagnosed cases, we used the 1,193 who had tested positive, along with another 290 patients who had had COVID-19 per their EHRs but had no test results (this latter group would have included, for example, patients who were treated for COVID-19 at Michigan Medicine but were not tested there). This resulted in a total of 1,483 diagnosed cases for the analysis. Lastly, for controls, we extracted data for 30,000 random patients who were alive, were not in the tested or diagnosed groups, and had an encounter in Michigan Medicine (Inpatient, Outpatient, or Emergency) between April 23, 2012, and June 21, 2020. We used this data to fit similar models as described above and evaluated performance using AUC. A detailed description of our procedure and results is included in the supplement."

Minor Comments

1. By no fault of the authors, the first paragraph of the introduction is (unfortunately) outdated.

Thank you for this comment. Yes, unfortunately that number is outdated, as is our focus in the Introduction section on the Delta variant, which is no longer the most prominent. The new section (p.3) reads:

"Although some countries have been successful in suppressing COVID-19 cases, the United States remains a hotbed, with a still-growing count of over 78 million cases and 900 thousand deaths as of February 22nd, 2022 [1]. Over 2 million people have been infected in the State of Michigan alone, resulting in more than 31,000 deaths [2]. Even as the initial pandemic has waned in some locations, novel SARS-CoV-2 variants such as B.1.617.2 ("delta"), and more recently B.1.1.529 ("omicron"), have continued to spark new waves of cases worldwide [3–8]"

2. Five-fold cross-validation is mentioned on line 343, yet I don't see it mentioned elsewhere. It seems that Monte Carlo cross-validation was used instead to assess model performance. Was five-fold cross-validation used to select the hyperparameters of the penalized logistic regression models on the training sets?

Thank you for pointing this out. This was an error in the text and we apologize for the mistake. The text has been updated to say "data-splitting" instead of "five-fold cross-validation".

References

Christophe Ambroise and Geoffrey J. McLachlan. Selection bias in gene extraction on the basis of microarray gene-expression data. Proceedings of the National Academy of Sciences, 99(10):6562–6566, 2002. ISSN 0027-8424. doi: 10.1073/pnas.102102699. URL https://www.pnas.org/content/99/10/6562.
