# Supplementary material for: Assessing the added value of linking electronic health records to improve the prediction of self-reported COVID-19 testing and diagnosis
Source: PLoS One. 2022 Jul 25;17(7):e0269017. doi: 10.1371/journal.pone.0269017 (PMC9312965; doi:10.1371/journal.pone.0269017)
Supplement: Attachment — Submitted filename: Response_to_reviewers.pdf [file pone.0269017.s018.pdf]

Response to Reviewer Comments

Title: *Assessing the added value of linking Electronic Health Records to improve the prediction of self-reported COVID-19 testing and diagnosis*

Authors: Clark-Boucher, D; Boss, J; Salvatore, M; Smith, JA; Fritsche, LG; Mukherjee, B

Ref: PONE-D-21-35566

We would like to thank the editorial board and the reviewers for their constructive feedback on our resubmission and giving us an opportunity to address the concerns through a second revision. Following are itemized responses to the reviewer comments. The original comments are in ***italics***, our responses are in **blue** and the resulting manuscript additions are quoted in **green**. We hope you find the revised manuscript suitable for publication in PLOS One.

Reviewer 5

1. Consider adding slightly more context to results of predictive models given in introduction (e.g. population information).

Thank you for raising this important point. We agree that this portion of the Introduction section could be more detailed, and we have added information that includes the sample size and population for each study. See below.

Vaid et al. used EHRs to predict mortality and critical events on a sample of 4,098 COVID-19 patients in New York City, USA, attaining Areas Under the Receiver Operating Characteristic curve (AUCs) of 0.79 to 0.89 [14]. Feng et al. used EHRs, specifically lab results, to predict suspected COVID-19 pneumonia with an AUC of 0.94 among 32 hospital admittees in Beijing, China [15]. Using EHR-derived comorbidities and social variables, Hippisley-Cox et al. built a model explaining 74.1% of the variation in time to death among 626,656 adults hospitalized for COVID-19 post COVID-19 vaccination in the United Kingdom [16]."

"With survey data on 3,829 adult mobile-phone application users across the United States, Allen et al. obtained an AUC of 0.79 for predicting positive COVID-19 test results using pre-test data only [10]."

We have also added information on our own study's population of interest and sample size later in the introduction section: "On a sample of 7,054 participants of Michigan Medicine biorepositories, we combine data from the Michigan Medicine COVID-19 Survey [11] with EHR-derived comorbidities and

sociodemographic variables; then, using both pools of variables, we construct prediction models for two outcomes: (1) whether survey respondents have been tested for COVID-19 and (2) whether they have been diagnosed with COVID-19 by either a physician or test, comparing model performance using AUC."

2. A potential limitation of using LASSO or elastic net regression for variable selection is that they are only guaranteed to asymptotically select the truly important features under stringent conditions. In particular, this metric of variable importance could be misleading if certain features are highly correlated. Have you checked the estimated correlation matrices of the variables used in your models? Otherwise, this might be worth mentioning in the discussion.

Thank you for your comment. We agree that these are limitations to LASSO and elastic net regression in finite sample size situations. In our case, with such a large number of features, there are bound to be correlations as you have described. The Spearman correlations among variables in our maximal dataset ranged from -0.87 to 0.94, with a median of 0.012 and an interquartile range from -0.01 to 0.04, suggesting that most variables were minimally correlated but a few very highly correlated. As we mention in the Methods section, elastic net tends to perform better than LASSO when predictors are correlated; specifically, LASSO will generally only pick one of two highly correlated features, whereas elastic net may be able to incorporate both simultaneously. In response to your comment, we have added the following point to our Discussion section: "There are also possible limitations to our specific choice of models. Variable selection approaches such as LASSO and elastic net can have difficulty choosing the optimal model under certain conditions: for instance, when predictors are correlated. For this reason, the proportion of times each variable was selected may not always be reflective of the true variable importance."

3. A limitation of the multi-predictor benchmark is the use of penalized logistic regression models. It is unlikely that parametric models are capable of capturing the true complexity of the relationships under study. Determining whether the conclusions of this study hold true when using more flexible predictive models could make for an interesting follow-up analysis.

Thank you for mentioning this limitation. We agree that exposure-outcome associations can be highly complex and that linear models may be limited for describing those associations. Experimenting with more advanced modeling tools like random forest, neural networks and ensemble methods like the super learner is a critical path for future research, though it is the beyond the scope this study. We believe that for our purposes, generalized linear models as a base comparison across the survey and EHR variables are

adequate, because despite their relative simplicity, they still can provide a fair comparison between variables across different data sources. In response to your comment, we have updated the Discussion section with the following explanation: "Moreover, parametric generalized linear models in general may struggle to capture the true nature of exposure-outcome associations, especially when those associations are complex and involves non-linearity and interactions. Future work could expand upon our findings by applying machine learning approaches that are more flexible than logistic regression, such as, for example random forests or neural networks as well as ensemble methods like the super learner [48]."

References (numbers extracted from revised manuscript)

10. Allen WE, Altae-Tran H, Briggs J, Jin X, McGee G, Shi A, et al. Population-scale longitudinal mapping of COVID-19 symptoms, behaviour and testing. Nat Hum Behav. 2020;4: 972–982. doi:10.1038/s41562-020-00944-2

11. Wu KHH, Hornsby WE, Klunder B, Krause A, Driscoll A, Kulka J, et al. Exposure and risk factors for COVID-19 and the impact of staying home on Michigan residents. PLoS One. 2021;16. doi:10.1371/journal.pone.0246447

14. Vaid A, Somani S, Russak AJ, De Freitas JK, Chaudhry FF, Paranjpe I, et al. Machine Learning to Predict Mortality and Critical Events in a Cohort of Patients With COVID-19 in New York City: Model Development and Validation. J Med Internet Res. 2020;22: e24018–e24018. doi:10.2196/24018

15. Feng C, Wang L, Chen X, Zhai Y, Zhu F, Chen H, et al. A novel artificial intelligence-assisted triage tool to aid in the diagnosis of suspected COVID-19 pneumonia cases in fever clinics. Ann Transl Med Vol 9, No 3 (February 2021) Ann Transl Med. 2021. Available: https://atm.amegroups.com/article/view/60789

16. Hippisley-Cox J, Coupland CA, Mehta N, Keogh RH, Diaz-Ordaz K, Khunti K, et al. Risk prediction of covid-19 related death and hospital admission in adults after covid-19 vaccination: national prospective cohort study. BMJ. 2021;374: n2244. doi:10.1136/bmj.n2244

48. Hastie T, Tibshirani R, Friedman J. The Elements of Statistical Learning. 2nd ed. Springer US; 2009.
